# Versatile, Modular, and General Strategy for the Synthesis of α-Amino Carbonyls

**DOI:** 10.1021/jacs.4c09434

**Published:** 2024-08-24

**Authors:** Jianzhong Liu, Matthew J. Gaunt

**Affiliations:** Yusuf Hamied Department of Chemistry, University of Cambridge, Lensfield Road, Cambridge CB2 1EW, U.K.

## Abstract

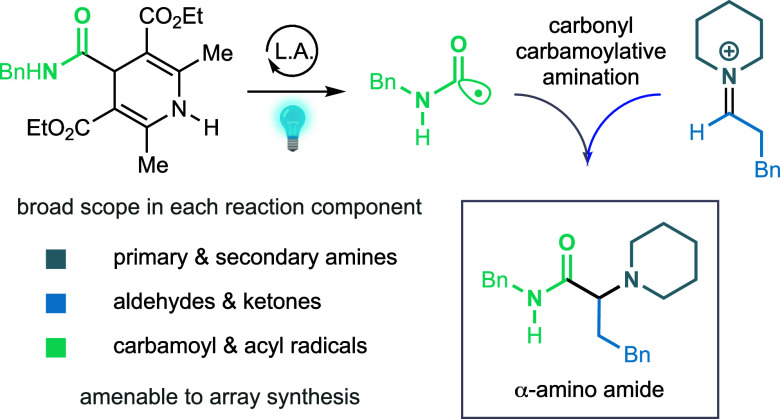

Modulating the basicity of alkylamines is a crucial factor in drug design. Consequently, alkylamines with a proximal amide, ester, or ketone have become privileged features in many pharmaceutical candidates. The impact of α-amino carbonyls has made the development of new methods for their preparation a continuous challenge in synthesis. Here, we describe a practical strategy that provides a modular and programmable synthesis of a wide range of α-amino carbonyls. The generality of this process is made possible by an extremely mild method to generate carbamoyl radicals, proceeding via a Lewis acid-visible-light-mediated Norrish type-I fragmentation of a tailored carboxamide reagent and intercepted through addition to in situ generated unbiased imines. Aside from the reaction’s broad scope in each component, its capacity to draw on plentiful and diversely populated amine and carbonyl feedstocks is showcased through a two-dimensional array synthesis that is used to construct a library of novel, assay-ready, α-amino amides.

## Introduction

Tuning the basicity of alkylamines is an important aspect in the design of biologically active molecules as it regulates their ionization state under physiological conditions and influences factors such as lipophilicity, solubility, metabolism, and interference with the hERG ion channel and targeted receptors, among others. As a result, an alkylamine’s bioavailability and cell permeability can be enhanced while off-target interactions can often be perturbed.^1−3^ One way to reduce an alkylamine’s basicity is through the incorporation of a carbonyl group adjacent to the nitrogen atom. Accordingly, alkylamines with a proximal amide, ester, or ketones have become privileged features in pharmaceutical candidates,^4−6^ and new methods for their preparation represent a constant goal for chemical synthesis,^7,8^ particularly when applied to the assembly of diverse libraries of α-amino carbonyl-derived pharmaceutical candidates.^9−11^

**Figure 1:**
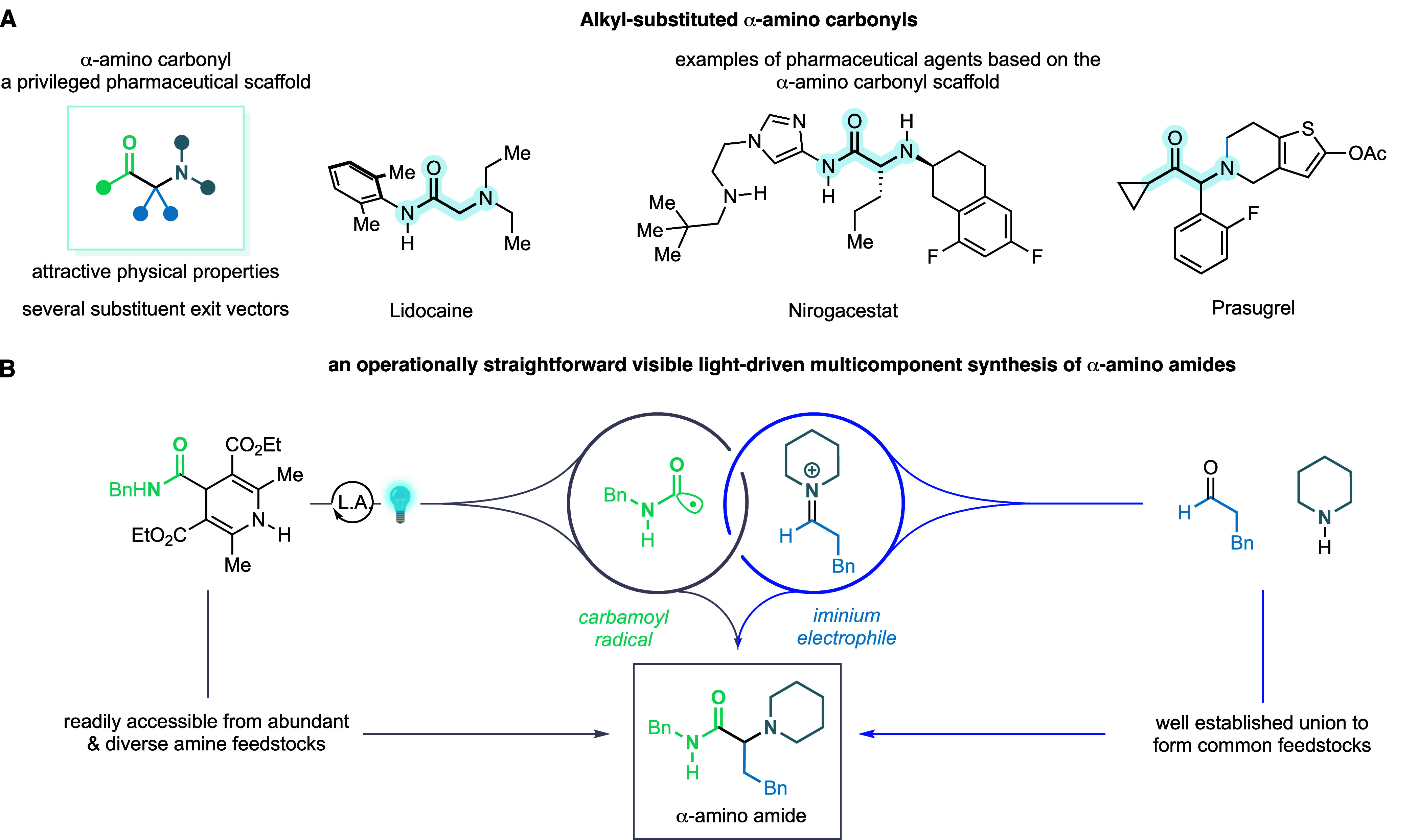
Reaction background and design. (A) α-Amino carbonyl motif and its prevalence in biologically active molecules. (B) Design plan for a carbonyl acylative amination reaction.

A substantial research effort has been directed toward the synthesis of pharmaceutically relevant α-amino carbonyls (Figure 1a).^7,8^ Beyond strategies that manipulate α-amino acids,^12^ methods exploiting the functionalization of enolates, enols, and enamines with appropriate electrophiles have also become commonplace.^8,13,14^ Most of these processes, however, require multiple steps and use of bespoke electrophiles or are limited by the type of carbonyl compound, meaning that a general platform for α-amino carbonyls remains elusive. Through an alternative approach involving acyl anion equivalents, multicomponent transformations have become established via the addition of cyanide (Strecker reaction) and isonitriles (Ugi reaction) to imines to form α-cyano amines and α-amido amides, respectively.^15,16^ Accordingly, the Strecker and Ugi reactions received extensive attention in the synthetic community, resulting in 1000s of primary research articles on the topics. Their extensive investigation has also meant that such multicomponent transformations have become attractive platforms for the preparation of libraries of potential pharmaceutical hit compounds via array synthesis^17^—a reaction matrix where discrete products are prepared in a spatially encoded fashion, ready for assay.^18,19^ Despite both the Strecker and Ugi reactions enjoying broad utility and uptake, they display several limitations. The Strecker reaction must navigate the use of toxic cyanide salts, and the α-cyano amine products require further synthetic manipulations to access the target α-amino carbonyl. The Ugi reaction requires the use of toxic isonitriles, which are often unstable, frequently difficult to synthesize, and not widely available, and the process is restricted to the synthesis of α-amino secondary amide derivatives. The realization of a new multicomponent reaction for the synthesis of (Csp^3^)-rich α-amino carbonyls that displays all the positive attributes of the Ugi and Strecker reactions but few of their disadvantages remains an important challenge to chemical synthesis. We reasoned that a new process should assemble the α-amino amide framework in a single step and draw from readily available and substantially populated classes of C(sp^3^)-rich feedstocks; not contain any residual activating or protecting groups in the products; proceed with near equimolar stoichiometry of reagents; and be amenable to array-type library synthesis. Recently, our laboratory established a versatile tertiary alkylamine synthesis platform called carbonyl alkylative amination, wherein a visible-light and silane-mediated activation mode can generate alkyl radicals under mild conditions and orchestrate their addition to in situ generated all-alkyl substituted iminium ions.^20,21^ Set against the challenges of a modular, practical, and general strategy for the synthesis of α-amino amides, we speculated that visible-light-mediated addition of a carbamoyl radical to an in situ generated iminium ion, unbiased by its substituents, might offer a comparably broad reactivity to the venerable Ugi multicomponent coupling.

Carbamoyl radicals are moderately nucleophilic open shell intermediates whose polarity would be matched to the electrophilic iminium ion acceptor, providing the basis for an effective coupling.^22^ The generation of carbamoyl radicals has, though, frequently involved the use highly functionalized and poorly tractable precursors under nonideal reaction conditions,^23−28^ which has precluded their wider use. The advent of visible-light photochemistry has rendered several more convenient carbamoyl-radical precursors^29−31^ and expanded the scope of accessible chemistries of this generally underexplored species. We speculated that an activation mode for carbamoyl radical formation based solely on excitation by visible-light would lay the foundation for an operationally straightforward and modular synthesis of α-amino amides. Accordingly, this ideal could be realized through visible-light-driven formation of carbamoyl radical and 1,2-addition to an all-alkyl iminium ion, which is formed in situ from an aldehyde or ketone and primary or secondary amine (Figure 1B). Such a transformation could overcome the limitations of other methods for α-amino amide synthesis, presenting a convenient and modular method to prepare a highly desirable class of C(sp^3^)-rich amine scaffold that is present in many pharmaceutical agents and could be of great utility in the discovery of new pharmaceutical agents.^32,33^

## Results and Discussion

**Figure 2:**
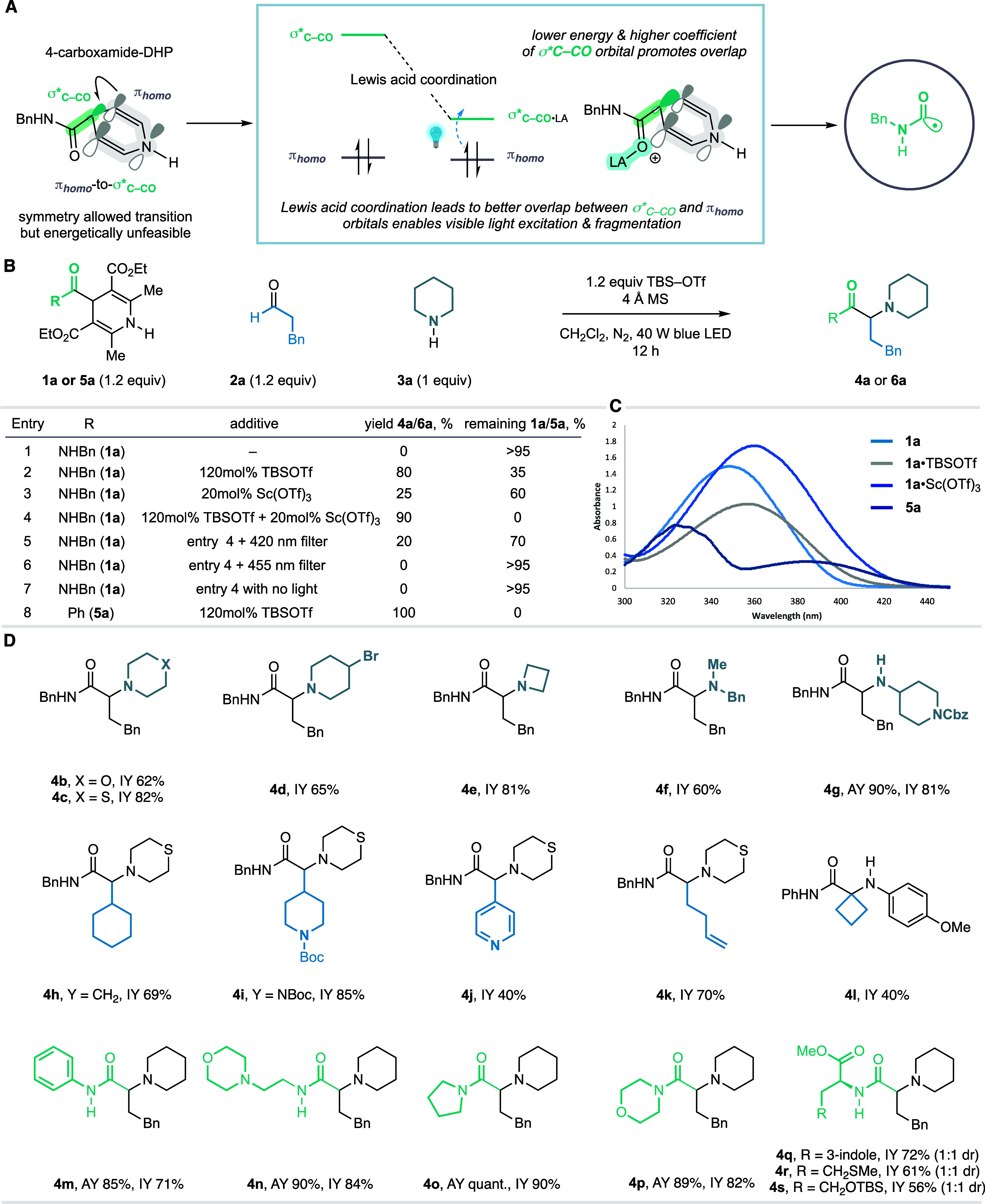
Reaction optimization and scope for carbamoyl reagent. (A) Lewis acid activation of 4-carboxamide DHPs to facilitate visible-light excitation and Norrish type-I fragmentation to a carbamoyl radical. (B) Selected optimization data for the modular synthesis of α-amino amides. (C) UV/vis spectra to evidence the activation of 4-carboxamide-DHPs via Lewis acid coordination. (D) Preliminary scope assessment of the α-amino amide synthesis process with respect to amine, aldehyde, and carbamoyl radical fragment. AY, assay yield; IY, yield of isolated product.

In considering a convenient source of carbamoyl radical, our attention focused on 4-carboxamide-1,4-dihydropyridines (DHPs) because they can be accessed by amide bond formation from the corresponding 4-carboxy-DHP,^34^ which exploits the modularity offered by the diverse amine feedstock pool. While 4-carboxamide-DHPs have been used as precursors to carbamoyl radicals,^35−37^ the action of a visible-light-mediated photocatalyst or formation of an electron–donor–acceptor complex with a reagent^38^ is generally required for their activation. On the basis that selective homolytic bond scission to the carbamoyl radical would occur via Norrish type-I fragmentation,^39,40^ a symmetry-allowed excitation between the π_HOMO_ of the DHP unit and σ_C–CO_^*^ orbital could be driven by visible-light irradiation (Figure 2A). To drive activation of the 4-carboxamide-1,4-DHPs solely under visible-light irradiation, we speculated that the energy of the σ_C–CO_^*^ orbital in 4-carboxamide-DHP reagents could be lowered by Lewis acid coordination to the carbonyl motif of the amide, bringing it closer in energy to the DHP-π_HOMO_ orbital, as well as polarizing the C–CO bond such that the coefficient of the σ_C–CO_^*^ orbital is increased at the C-4 position. This would lead to better overlap and enablement of visible-light excitation to the electronic configuration required for Norrish type-I fragmentation to the carbamoyl radical.

A series of preliminary experiments were carried out to probe a Lewis acid activation mode for visible-light-mediated carbamoyl radical formation. Irradiating a dichloromethane solution of 4-carboxamide-1,4-DHP **1a** in combination with aldehyde **2a**, amine **3a**, and 4 Å molecular sieves—the components required to form the iminium ion acceptor in situ—showed no conversion to the desired α-amino amide **4a** (Figure 2B, entry 1). As expected, the use of photocatalysts {Ir[dF(CF_3_)ppy]_2_(dtbpy)PF_6_ and 4CzIPN} led to an intractable reaction mixture, underlining their incompatibility with the reaction’s sensitive iminium and enamine intermediates. The impact of Brønsted and Lewis acid additives was assessed, and a significant increase in reactivity was observed through the consumption of **1a** and the formation of α-amino amide **4a**. The addition of 1.2 equiv of TBS-OTf led to the most dramatic improvement and produced an assay yield for **4a** of 80% with 35% of **1a** remaining (entries 2,3). Notably, these reaction conditions are effective at near equimolar stoichiometries of the reaction components. Alongside its role in activating the 4-carboxamide-DHP, TBS-OTf facilitates a high concentration of iminium ion, and it is possible that the triflate counterion may also enhance its electrophilic reactivity. A preliminary kinetic assessment revealed a zero-order dependence on aldehyde, amine, and TBS-OTf, and a first-order dependence on **1a**. This suggests that the homolysis of **1a** is rate limiting; a rate constant for the reaction was determined to approximately 1.8 × 10^–5^ S^–1^. While the data show a critical role of TBS-OTf in promoting the desired reaction and supporting its role as an activator for the carboxamide group, a reaction in the presence of Sc(OTf)_3_ showed an almost 4-fold acceleration in reaction rate (see Supporting Information, Figures S35 and S36), which could be explained by its action as a more effective Lewis acid for the activation of **1a**. The reaction also worked well with a preformed iminium ion in the absence of 4 Å molecular sieves (not shown). Therefore, optimal reaction conditions involved the use of 20 mol % Sc(OTf)_3_ in combination with TBS-OTf, which resulted in a quantitative assay yield of **4a** (90% yield of isolated product) and **1a** was completely consumed (entry 4). Further support for the impact of Lewis acid additives was gained from the examination of the UV/vis spectra of **1a** when complexed with TBS-OTf or Sc(OTf)_3_. In both cases, a clear bathochromic shift is observed compared to the parent compound, which also supports the energetic proximity of the σ_C–CO_^*^ and π_homo_ orbitals (Figure 2C). Analysis of the ^1^H NMR spectrum of a 1:1 mixture of 4-carboxamide-DHP **1a** and TBS-OTf showed a clear downfield shift of both amide N–H and the DHP N–H signals, although the shift was more substantial for the amide signal. While this suggests that coordination to the amide is most likely the predominant interaction, we cannot rule out that such a downfield shift in the amide N–H is not due to a coordination to the esters of the DHP motif, which could also explain the observed bathochromic shift in the UV/vis spectrum. No reaction was observed in the absence of light or using a blue-LED fitted with a 455 nm filter, and almost all **1a** was recovered; with a 420 nm filter, 20% of **4a** was formed and 70% of **1a** remained (entries 5–7). These experiments are consistent with the bathochromic shift of the tail wavelength to 440 nm when **1a** is coordinated to a Lewis acid. We draw an important comparison to the seminal work of Melchiorre and co-workers, who elegantly showed that visible-light irradiation alone could convert 4-benzoyl DHP **5a**, a related class of ketone-containing precursors, to its corresponding acyl radical, which was intercepted through an organocatalytic Stetter-type reaction with cinnamaldehydes as well as other transformations.^41,42^ With 4-benzoyl DHP **5a**, the inherently lower energy of the ketone σ_C–CO_^*^ orbital and the impact of a higher polarization in the C–COPh bond would facilitate more facile visible-light-mediated fragmentation. Consequently, **5a** was also adaptable for the synthesis of the corresponding α-amino ketones; irradiation of dichloromethane solution of **5a**, **2a**, amine **3a**, TBS-OTf, and 4 Å molecular sieves led to quantitative and extremely rapid conversion (<2 min) to the desired α-amino ketone **6a**, substantially expanding the potential scope of our general strategy to different classes of α-amino carbonyls and beyond the reach of classical Ugi and Strecker processes (entry 8).

A preliminary scope for the reaction was explored by varying the amine component in combination with **1a** and aldehyde **2a** (Figure 2D). The reaction worked well with cyclic and acyclic secondary amines (**4a–f**) and primary amines (**4g**, **S1**) to produce good yields of the desired α-amino amide products. Several classes of substituted aldehydes also worked well in the reaction with substrates containing α-branched, heterocyclic, and linear substituents, successfully forming the desired products (**4h–k**). Surprisingly, benzaldehydes performed poorly in the reaction (see Supporting Information for further details), although a reaction with 4-pyridaldehyde produced synthetically useable **4j**. However, cyclobutanone was a competent substrate and produced the fully substituted α,α′-disubstituted amino amide **4l** in synthetically useable yield. A series of 4-carboxamide-DHPs, formed in one step by coupling of the corresponding acid with an amine, performed well on reaction with aldehyde **2a** and piperidine **3a**; cyclic secondary amides, anilides, and primary amides derivatives were converted smoothly to the corresponding α-amino amides (**4m–p**). 4-Carboxamide-DHP reagents derived from α-amino acids could also be efficiently utilized, providing direct access to an unusual class of non-natural dipeptides (**4q–s**).

The synthesis of C(sp^3^)-rich α-amino amides, presented here, is distinct from an insightful related transformation reported by von Wagelin and co-workers that involved the photocatalyzed addition of carbamoyl radicals to N-aryl benzaldimines.^35^ While an important demonstration of the utility of carbamoyl radicals, N-aryl benzaldimines are a class of imines whose reactivity toward radical addition is augmented by both their aniline and benzaldehyde components through activation of the carbon–nitrogen double bond and stabilization of the resulting aminyl radical adduct.^43^ Importantly, no examples of reactions with a combination of alkylamines and alkyl-substituted aldehydes or ketones were reported. Our process utilizes unbiased amine and aldehyde components, the coupling of which with the carbamoyl radical leads to the C(sp^3^)-rich α-amino carbonyls demanded by the quest for complex molecules with higher levels of saturated carbon framework. Indeed, our own work in the field of visible-light-mediated amine synthesis clearly shows the challenges associated with the synthesis of all-alkyl imines and iminiums and the reactivity challenges associated with them compared to reactions with bespoke N-aryl benzaldimines.^20,21,44^

**Figure 3:**
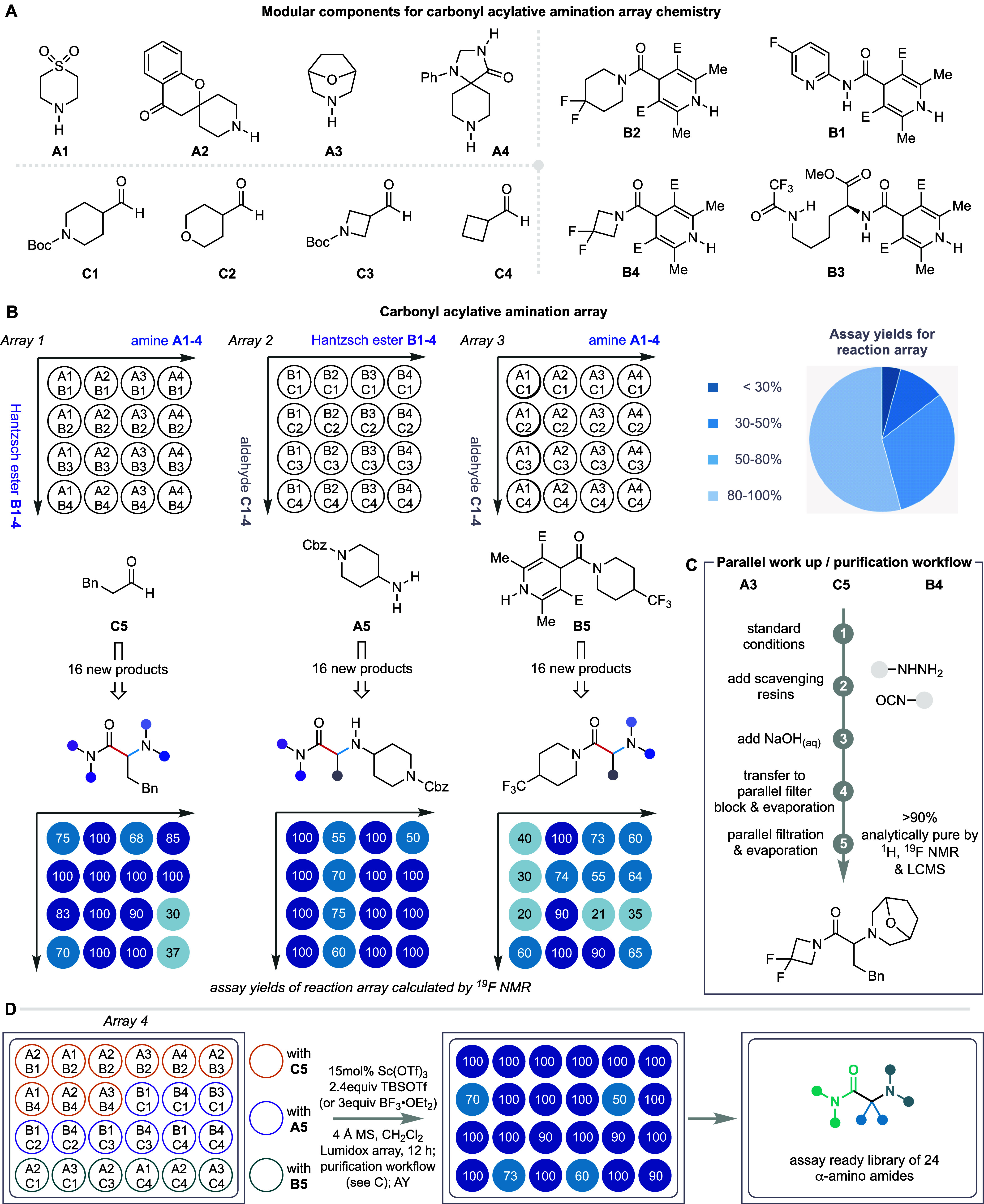
Array development. (A) Components for reaction array. (B) 2D-parallel reaction arrays. (C) Purification workflow for α-amino amide synthesis. (D) Preparation of 24-compound array library that is “ready for assay”. AY, assay yield; IY, yield of isolated product.

The potential efficacy of this method lies in exploiting its operationally straightforward and modular nature to rapidly generate libraries of α-amino amides from extensive classes of abundant and diversely populated amine and carbonyl building blocks. When conducted in a systematic and parallel fashion, the new process could be amenable to array chemistry applications.^17^ As a proof of concept, a parallel two-dimensional array for the synthesis of α-library of α-amino amides was designed, wherein each component could be systematically varied (Figure 3A). To assess the reaction outcome, a fluorine atom was included in each 4-carboxamide-DHP so that a quantitative assay yield of the product could be calculated by ^19^F NMR analysis. Three arrays were designed based on a systematic variation of the three components to produce 48 new α-amino amides. Accordingly, array 1 assessed four amines with four 4-carboxamide-DHPs, while the same aldehyde was retained throughout; array 2 deployed four aldehydes and four 4-carboxamide-DHPs while the amine was kept constant; and array 3 utilized a single 4-carboxamide-DHP while implementing four amines and four aldehydes. Each reaction array was irradiated with a blue LED light source (Lumidox II, 445 nm 96-well LED Array), under the standard conditions for 20 h; filtration and evaporation of solvent provided a crude reaction mixture that could be quantified by ^19^F NMR using trifluorotoluene as an internal standard (Figure 3B). Across all the reaction arrays conducted, we observed the formation of product, in most cases with high assay yields (see pie chart, Figure 3B, and Supporting Information, Figure S39), which underlines the generality and robustness of the carbonyl acylative amination reaction.

As well as high yields, a successful reaction array also requires high product purity such that libraries can be advanced directly to assay without the need for the chromatographic purification of a large number of compounds.^45,46^ In addition to the α-amino amide product, this process also generates a pyridine derivative formed from the radical donor, as well as residual starting materials; interestingly, other byproducts were rarely observed. After assessing several workup and scavenging strategies, a successful workflow involved the “in well” treatment of the reaction mixture with scavenging Amberlyst resins decorated with isocyanate (for amine)^47^ and hydrazine (for carbonyl)^48^ functionalities and agitation for 4 h. Next, 2 M NaOH was added to the crude mixture and stirred for 16 h, followed by transfer to a filtration block to remove the resins (Figure 3C). After evaporation of the eluant (removing methanol), followed by reloading the sample onto the plate filled with silica and MgSO_4_, filtration provided α-amino amides with a purity (determined by LCMS, ^19^F NMR, and ^1^H NMR analysis) comparable to that obtained from chromatography. With this protocol, one round of array reactions was performed on a 24-well plate, which encompassed various combinations of amines, aldehydes, and reagents sampled from across the original three arrays. The optimized workup protocol was applied to this reaction plate to generate a library of high-purity α-amino amides (Figure 3D and see Supporting Information, Figure S43).

**Figure 4:**
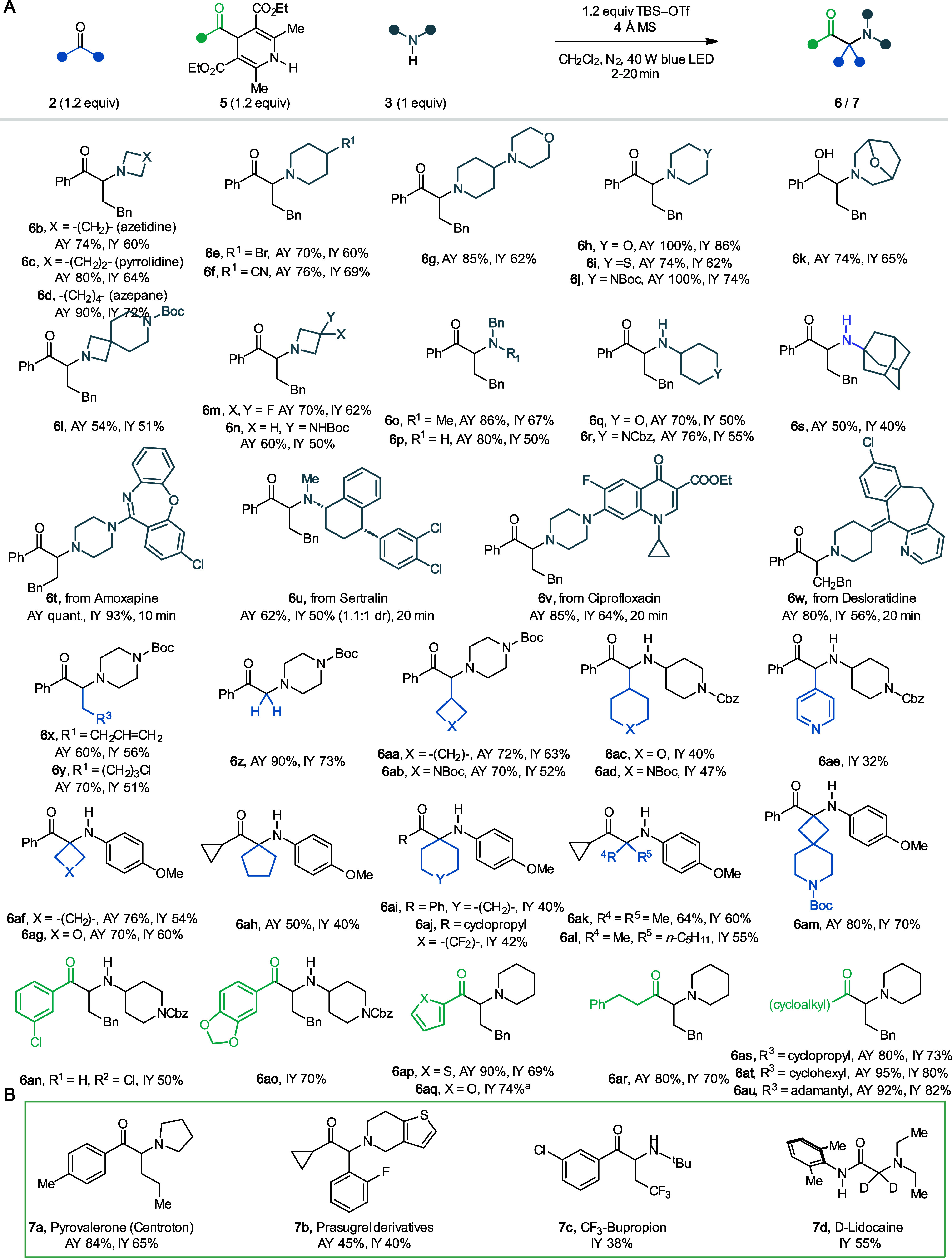
Reaction scope for the ketone reagent. (A) Scope of α-amino ketone synthesis in terms of amine, carbamoyl, and carbonyl component. (B) One-step synthesis of α-amino amide and α-amino ketone pharmaceuticals and selected analogues. AY, assay yield; IY, yield of isolated product.

To further expand the functional space accessible with this modular reaction, the synthetic utility of the 4-keto-DHPs was assessed as reagents for the synthesis of α-amino ketones. Like α-amino amides, the corresponding ketones are privileged structural features and frequently appear in pharmaceutical agents.^4−6^ As expected, the 4-keto-DHP reagents were far more reactive than their corresponding carboxamide derivatives and did not require the addition of Sc(OTf)_3_ to engineer good reactivity. The reaction displayed a remarkably broad scope in each component, and a selected set of examples is shown in Figure 4 (see Supporting Information, Figure S44 for additional examples). Many classes of secondary amine produced good yields of the desired α-amino ketone products (**6a–o**), whose structures are frequently encountered in the pharmaceutical and agrochemical candidates.^49^ Primary alkylamines were good substrates in the reaction, with benzylamines, α-branched amines, and bulky alkylamines performing well (**6p–s**). The tolerance of the reaction to a range of functional groups was exemplified by its successful deployment using several pharmaceutical agents, all of which produced the corresponding complex amine products in good yields (**6t–w**).

Several classes of substituted aldehydes also worked well in the reaction with substrates containing linear, branched, and saturated heterocyclic substituents, all producing good yields of the α-amino ketone products with either secondary or primary amine coupling partners (**6x–6ae**). A reaction using formaldehyde produced the unsubstituted α-amino ketone product in an excellent yield (**6z**). At the opposite end of the steric spectrum, ketones were also good substrates when used in combination with anilines; cyclic and acyclic ketones were equally effective (**6af–6am**). Although the yields are slightly lower when using ketones compared to aldehydes, their deployment provides direct access to highly functional variants of fully substituted α-amino carbonyls that are not always straightforward to prepare by other methods. A range of aryl, heteroaryl, and alkyl-substituted 4-keto-DHPs produced the corresponding α-amino ketones in good yields (**6an-a**).

Given that the amino carbonyl motif is present in several marketed pharmaceuticals that treat disorders ranging from obesity and fatigue to stroke and heart disease, we tested whether this multicomponent reaction could generate these drug molecules in a single step. Several α-amino ketone pharmaceuticals could be assembled in good yield after very short reaction times (Figure 4, **7a–d**).^6,50,51^ The modularity of the process means that changing one of the components in the reaction leads directly to the synthesis of closely related analogues, which we demonstrated through the synthesis of an analogue of bupropion (**7c**).^52^ Deployment of *d*_2_-formaldehyde in combination with an appropriate 4-carboxamide-DHP and diethylamine enabled the single-step synthesis of a deuterated analogue of lidocaine (**7d**).^53^

## Conclusions

These carbonyl carbamoylative amination and carbonyl acylative amination reactions represent a practical and general alternative to the venerable Ugi and Strecker reactions and a straightforward means of producing a wide spectrum of functionally and structurally diverse α-amino carbonyls. The development of robust modular methods for the synthesis of complex C(sp^3^)-rich amines remains an underappreciated challenge. Taken together with our work on the related carbonyl alkylative amination process,^20,21^ the work presented here substantially adds to this broad amine synthesis platform and streamlines the synthesis of these important structures, which is likely to be a great utility in the quest for new bioactive molecule.

## Supporting Information Available

Materials and methods; optimization studies; experimental procedures; mechanistic studies and ^1^H NMR, ^13^C NMR and ^19^F NMR spectra; and HRMS, IR, and UV–vis data (PDF)
