# Supplementary material for: Versatile, Modular, and General Strategy for the Synthesis of α-Amino Carbonyls
Source: J Am Chem Soc. 2024 Aug 24;146(35):24699–707. doi: 10.1021/jacs.4c09434 (PMC11378281; doi:10.1021/jacs.4c09434)
Supplement: Supplementary file 1 — ja4c09434_si_001.pdf [file ja4c09434_si_001.pdf]

Supplementary Materials for

A versatile, modular and general strategy for the synthesis of a-amino carbonyls

Jianzhong Liu, Matthew J. Gaunt*

Correspondence to: mjg32@cam.ac.uk

This pdf file includes.

Materials and Methods 3
  Reaction Setup 4
  Safety Considerations 4
  General Procedure for Carbonyl Acylative Amination (CAcylA) 4
  Useful information related to the Carbonyl Acylative Amination (CAcylA) 5
Lewis-acid activation strategy inspired by the molecular orbital interaction of Hantzsch ester (DHP) reagent 7
  Molecular orbital identification of Hantzsch ester (DHP) 7
  Molecular orbital analysis for Lewis-acid-coordination enabled visible-light photo-excitation and Norrish-type fragmentation. 8
  Initial evaluations to test molecular orbital hypothesis for the Lewis-acid activation strategy 9
  UV-visible light absorption study to testify the Lewis-acid activation system 10
Reaction optimization and controls 13
Synthesis of Hantzsch ester reagent 17
Synthesis of starting material for the study 23
Mechanistic studies 24
  Light on-off experiments 24
  Radical clock experiments 25
  Acyl radical trapping experiments 27
  UV-visible light absorption studies of Carbonyl Acylative Amination (CAcylA) for ketone-DHP reagent 28
  Kinetic studies of Carbonyl Acylative Amination (CAcylA) 30
  The kinetic comparison between TBS-OTf and TBS-OTf/Sc(OTf)$_3$ activation of Carbonyl Acylative Amination (CAcylA) 41
Parallel two-dimensional array for the synthesis of a library of alfa-amino carbonyls 42
  Plate set-up and initial reactivity evaluation by ^{19}F-NMR: 42
  Reaction set-up and work-up for parallel-array applications 45

General workflow for array applications 46
Extended supplementary substrates 49
References 50
Carbonyl Acylative Amination products 51
^{1}H and ^{13}C NMR Spectra Data 51
^{19}F-NMR to calculate the assay yield ....
Spectra of the array library 101

Materials and Methods

All reactions were run under an inert atmosphere (N_2) unless otherwise stated, with oven-dried glassware, using standard techniques. Anhydrous solvents were obtained from solvent stills (Et_2O was distilled from sodium triphenylmethane ketyl; THF from $LiAlH_4$; MeCN, dichloromethane, hexane, and toluene from CaH_2). Commercial anhydrous dichloromethane was used for this study. Powdered 4Å molecular sieves (MS) were activated prior to use by prolonged heating (250 °C) under high-vacuum (<1 mbar) and stored in a round-bottomed flask under N_2. Similar yields were obtained using commercially available activated powdered 4Å MS. Aldehydes were used as supplied if sufficiently pure, otherwise they were purified either by distillation or flash column chromatography and used immediately (hydrocinnamaldehyde was used after distillation as the standard aldehyde). Amines were used as supplied if sufficiently pure, otherwise they were purified either by distillation or flash column chromatography and used immediately (piperidine was used after distillation as the standard amine). Formaldehyde solution (37% in H_2O with 5-10% MeOH as stabiliser) and Boron trifluoride diethyl etherate ($BF_3.Et_2O$) was purchased from Sigma Aldrich and used as supplied. TBSOTf and TMSOTf were purchased from Fluorochem and used as supplied. All other commercial reagents were used as supplied unless otherwise stated.

Irradiation of the reaction mixture was achieved using a 40 W Kessil A160WE LED – Tuna blue aquarium light (setup: max blue, max intensity) and Lumidox® II, 445 nm 96-well LED Array. UV-Vis analysis was performed on a Shimadzu UV-1800 spectrophotometer.

Analytical thin-layer chromatography (TLC) was performed on Merck Kieselgel 60 F254 0.20 mm precoated, glass backed silica gel plates. Visualization of the developed chromatogram was performed by UV absorbance (λ_{max} = 254 nm), and/or by aqueous $KMnO_4$. Flash column chromatography was performed using silica gel (Merck Geduran Si 60 [40-63 µm]) with the indicated solvent system.

Nuclear magnetic resonance (NMR) spectra were recorded on a Bruker DPX 400 or DPX 500 spectrometer with cryoprobe. Chemical shifts (δ) for ^1H NMR spectra are recorded in ppm from Me_4Si with the solvent resonance as the internal standard ($CDCl_3$ = 7.26 ppm, DMSO-d_6 = 2.50, C_6D_6 = 7.16 ppm, CD_3OD = 3.31 ppm). Data is reported as follows: chemical shift [integration, multiplicity (s = singlet, d = doublet, t = triplet, q = quartet, quint = quintet, sext = sextet, spt = septet, m = multiplet, br = broad), coupling constant and molecular assignment]. ^{13}C NMR spectra are reported in ppm from Me_4Si with the solvent resonance as the internal standard ($CDCl_3$ = 77.00 ppm, DMSO-d_6 = 39.52, C_6D_6 = 128.06 ppm, CD_3OD = 49.00 ppm). ^{19}F NMR spectra are reported in ppm from $CFCl_3$ and are uncorrected. ^{35}P NMR spectra are reported in ppm from 85% H_3PO_4 and are uncorrected.

Infrared spectra (FT-IR) were recorded using a Perkin-Elmer Paragon 1000 Fourier transform Spectrometer equipped with ATR and analyzed as thin films, with absorption maxima (ν_{max}) being quoted in wavenumbers (cm^{-1}) and characteristic peaks being defined (s = singlet, d = doublet, t = triplet, q = quartet, quint = quintet,

sext = sextet, spt = septet, m = multiplet, br = broad). High Resolution Mass spectrometry (HRMS) was carried out by the ESPRC Mass Spectrometry Service at the University of Swansea using an LTQ Orbitrap XL spectrometer with positive ion nano-electrospray. Melting points (m.p.) were recorded using a Gallenkamp melting point apparatus and are reported uncorrected.

Reaction Setup

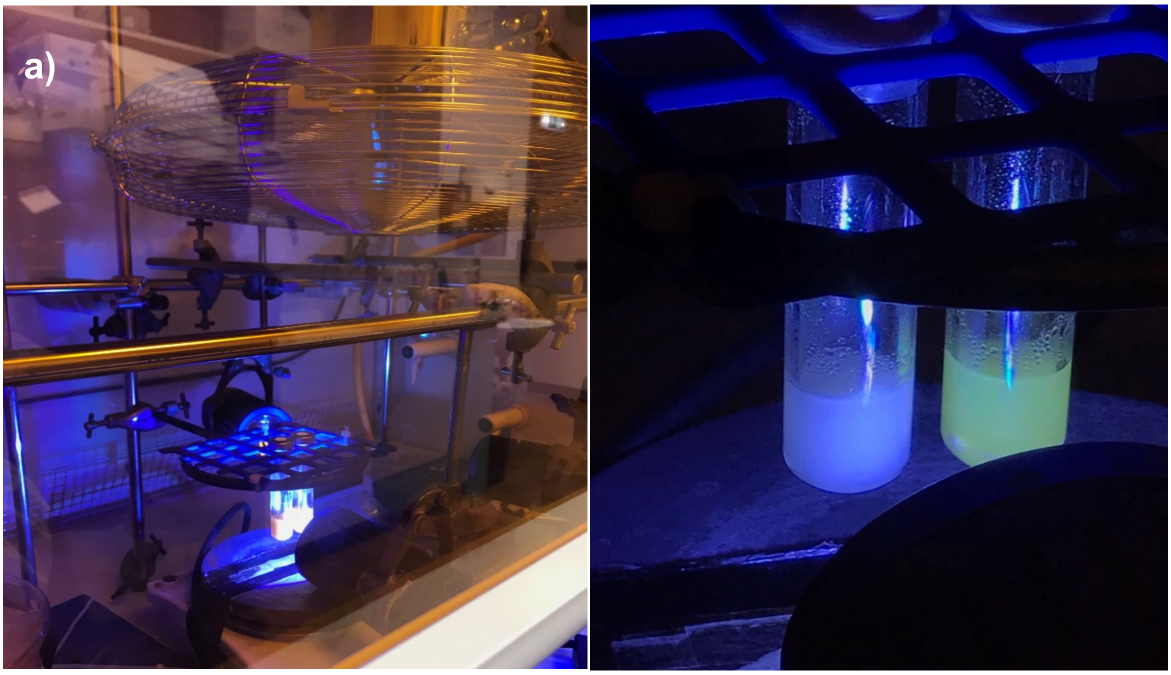

Fig. S1. a) General reaction setup (0.1 mmol). b) In the case of aldehyde substrates with the Benzoyl-DHP reagent in the Carbonyl Acylative Amination (CACA) chemistry, note the instantaneous color change (5 min – 10 min) of the reaction from yellow to white, indicating completion of the reaction.

Safety Considerations

Although little is known about the long-term exposure to short wavelength LED light (400–500 nm), it is reasonable to implement all available safety precautions when conducting experiments with high power LEDs. All reactions should be conducted with suitable shielding and bend a simple yellow screen filter. UV and blue light blocking safety glasses (e.g., SCT-Orange, typically blocking wavelengths < 500 nm) should be worn at all times when working with strong blue LED lights.

General Procedure for Carbonyl Acylative Amination (CAcylA)

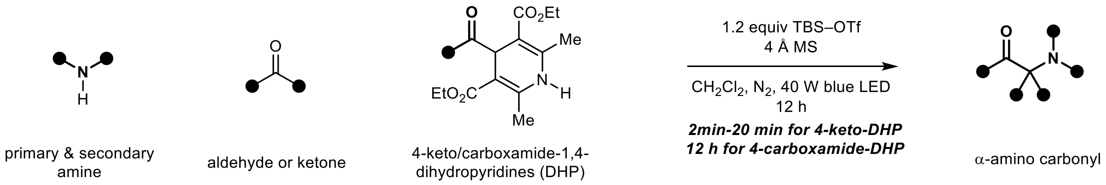

General procedure A: An oven dried vial (Biotage Microwave Process Vial 10–20 mL,

equipped with gas-tight septum) was charged with a stir bar, 4Å MS (200 mg) and DHP-reagent (1.2 equiv.). The vial was sealed, evacuated, and backfilled three times with 1 atm of N_2. Dry dichloromethane (2 mL, 0.05 M) was added followed by the addition of amine (0.1 mmol, 1 equiv.), aldehyde (1.2 equiv.). The mixture was continued to stir for 1 min and then TBSOTf (1.2 equiv, in the case of secondary amine) or $BF_3.Et_2O$ (1.5 equiv, in the case of primary amine) was added using a microsyringe. If the amine or aldehyde was solid, it was added along with the DHP-reagent first and then sealed and backfilled with N_2. Then the reaction mixture was irradiated using a 40 W blue LED lamp (Kessil A160WE Tuna Blue) with vigorous stirring for 2-20 min (4-keto-DHP) or 12 h (4-carboxamide-DHP) at room temperature, the Kessil lamp was positioned 5 cm from the vial along with a desk-fan for cooling. After that, the reaction mixture was filtered and transferred into a 50 mL round bottom flask. Dichloromethane (15 mL) was added, and the solution was neutralized upon stirring with sat. $NaHCO_3$ (aq.) (10 mL) for 30 minutes. The resulting solution was transferred to a separating funnel and organic layer was separated by dichloromethane (15 mL x 3). The organic phase was dried over anhydrous $MgSO_4$, and the solvent was removed in vacuo. The crude material was purified by flash column chromatography.

Procedure B:

In the case of ketone coupling partners, perfomed as general procedure A but using ketone (2.0 equiv) and prolonging the reaction time to 7 h.

Procedure C:

In the case of amine hydrochloride substrates, performed as general procedure A but adding Et_3N (1.0 equiv.) to the reaction system.

Procedure D:

In the cases of 4-carbamoyl DHP reagent, the combination of $Sc(OTf)_3$ (20mol%) with TBSOTf (1.2 equiv) or $BF_3.Et_2O$ (1.5 equiv) resulted in high transformation and full consumption of reagents. It has been employed in the parallel array evaluation.

Useful information related to the Carbonyl Acylative Amination (CAcylA)

➢ ***Purification of Hantzsch ester (HEH)***

The purifty of Hantzsch ester reagent is very important to the high performance and reproducibility of the reaction. Before crystallization, lower and varable yield was obtained. (E.g., for the standard coupling of piperdine, hydrocinnamaldehyde and

benzoyl-HEH, 50-70% yield was obtained). However, after crystallization of benzoyl-hantzsch ester reagent using DCM and *n*-Hexane, quantitative yield of product was obtained in 5 minutes).

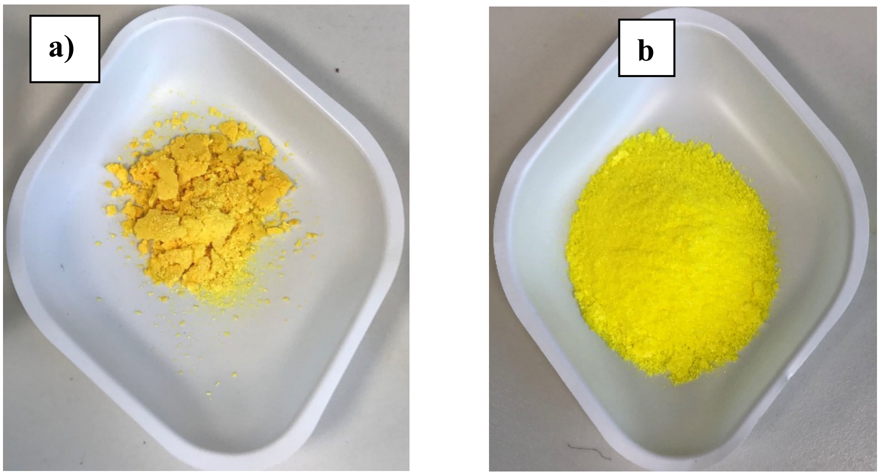

Fig. S2. a) Benzoyl-Hantzsch ester reagent (before crystallization, lumpy solid). b) Benzoyl-Hantzsch ester reagent (after crystallization, granular crystals).

➢ ***Choice of amine source***

It was noted that the amine hydrochloride salt was detrimental to the yield. It was much better to use free amine as the substrates. If not applicable, additional triethylamine (1.0 equiv) was added to neutralize the external acid.

➢ ***Pre-mixing the components***

It has been observed that thoroughly stirring the mixture prior to introducing the activators is crucial. Otherwise, the outcomes could become unpredictable with mess reaction systems.

➢ ***Reagent and solvent used in the chemistry***

Compared with the drying solvent, the normal solvent of dichloromethane diminished the yield slightly (about 20% lower in yield for the standard reactions). 4A powdered molecular sieves are the best option for the process, other types of molecular sieves such as 5A powdered, 3A powdered, and molecular sieves of beads are less efficient. Without molecular sieves and under air conditions, the yields were decreased slightly as well (about 30% and 10% lower in yield respectively for the standard reactions), indicating that the acylative amination process was not that sensentive to ambient environment. But only trace amount of product was isolated without TBSOTf (e.g., for the standard coupling of piperdine, hydrocinnamaldehyde and benzoyl-HEH, 15% yield of product was detected).

➢ *Stoichiometry of the reaction*

Although 1:1.2:1.2 stoichiometry (amine/aldehyde or ketone/reagent) is sufficient for the success of the transformation, 1:2:1.2 stoichiometry improved the yield slightly where α-branching of the amine or aldehyde was present (e.g., for the late-stage functionalization of complex drug moleclues).

➢ *The stability of Hantzsch ester reagent*

The freshly synthesized Hantzsch ester reagent could be stored in the fridge (at -5 °C) under dark for months without detectable deterioration.

➢ *Reaction performance of primary amines and secondary amines subcomponent*

Competitive experiments showed that secondary amines are more reactive than primary amines in this acylative amination transformation (shown below).

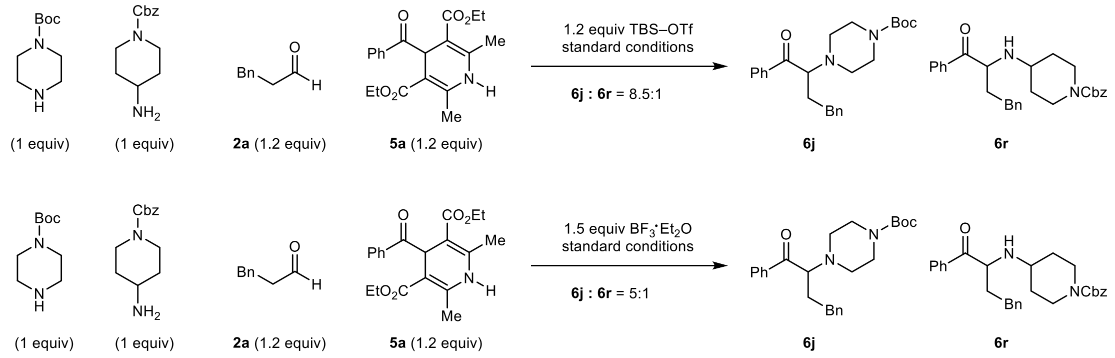

Fig. S3. Competitive experiments of acylative amination process regarding primary and secondary amines.

➢ *The isolation issue of the amines*

It was noted that with almost every example, isolated yields were 10–20% lower than assay yields. This is an issue of recovery from silica gel chromatography and is commonly encountered with the basic aliphatic alkylamines.

Lewis-acid activation strategy inspired by the molecular orbital interaction of Hantzsch ester (DHP) reagent

Molecular orbital identification of Hantzsch ester (DHP)

Firstly, the molecular orbital diagram of Hantzsch ester (DHP) π-system was identified based on Hückel theory[50] (Fig. S4). The frontier orbital would the none-bonding orbital

(HOMO) and ψ_5 orbital (LUMO). Superficially, it appears that the photo-excitation and fragmentation involves the electronic-excitation from the HOMO to the LUMO ($\pi \rightarrow \pi^*$) of the conjugated 1,4-dihydropyridines (DHP) system of Hantzsch ester. Subsequently, there is an orbital interaction between the LUMO of DHP and the σ^* bond of the 4-C–CO bond, through which the energy is favored, however the symmetry of the interaction is wrong[51,52] (Fig. S5).

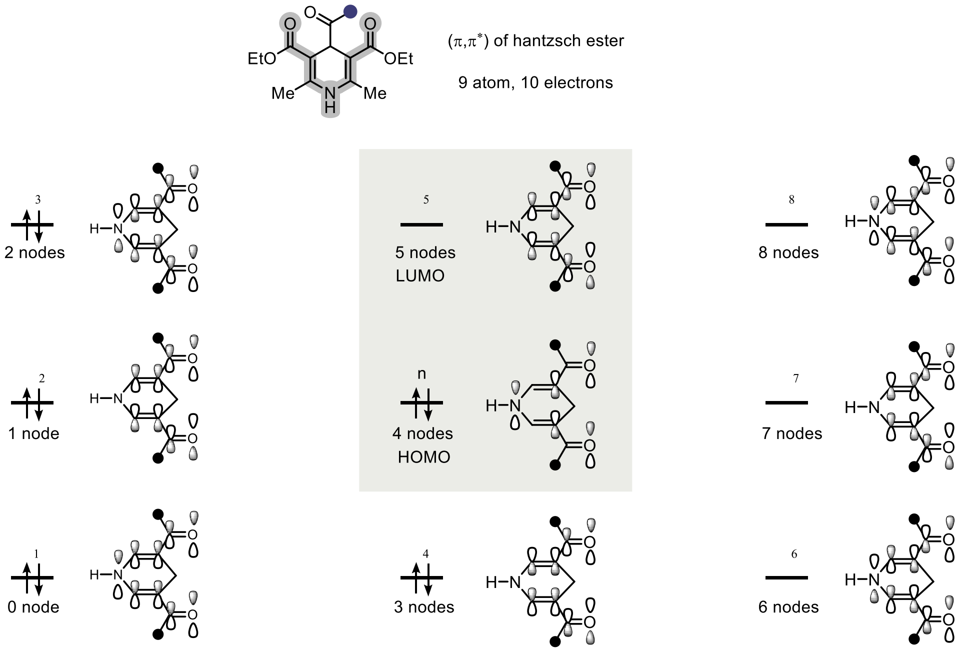

Fig. S4. Hückel molecular orbital diagram of Hantzsch ester (DHP) π-system.

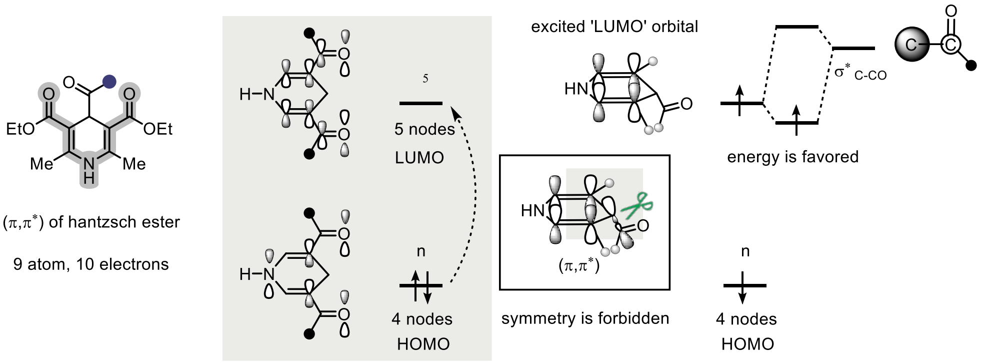

Fig. S5. Molecular orbital interation involved in the photo-excitation and Norrish-type fragmentation of DHP

<u>Molecular orbital analysis for Lewis-acid-coordination enabled visible-light photo-excitation and Norrish-type fragmentation.</u>

The above result provides insight into the fundamental molecular orbital interactions to be considered during the photoexciation and subsequent Norrish-type framenation process of DHP. Accordingly, the interaction would be the direct electronic excitation from HOMO of the DHP to the σ^* bond of the 4-C–CO bond ($\pi \rightarrow \sigma^*$) to obey the correct symmetry of molecular orbital interactions, thereby facilitating the C-C bond cleavage. This interaction would be energy-favored by lowering the energy of the σ^* C-C bond

via Lewis-acid coordination to the carbonyl motif of the amide (Fig. S6). Moreover, the lewis-coordination make the C-C(CO) bond more polarized to contribute higher coefficient at the cleavaged carbon atom in the σ* C-C(CO) bond, thus enbling stronger orbital interation. Additionally, the molecular orbital diagram provides insight that the ketone-reagent would be easier to undergo fragmentation due to the lower energy and polarized atomic orbital coefficient of the σ* C-C(CO) bond, probably even without activation, compared to σ* C-C(CONR).

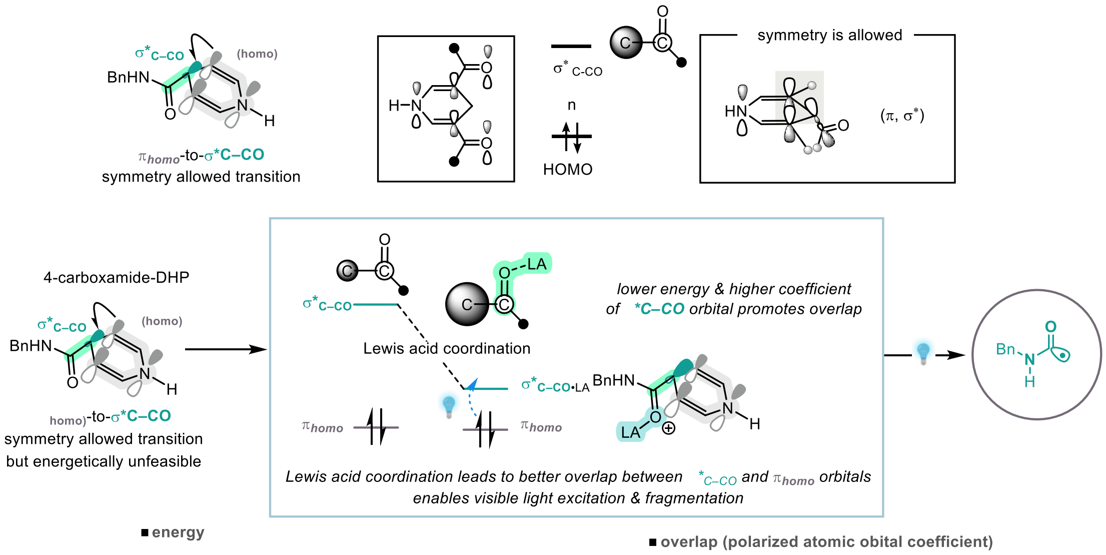

Fig. S6. The hypothesis of Lewis-acid-enabled activation of photo-excitation and Norrish-type fragmentation of DHP

Previous literature showed that the carbamoyl radical generation from carboxyamide-Hantzsch ester reagent requires the use of photocatalyst. However, photo-redox cycle may not be compatible with the presence of reactive iminium ions, which are not only readily reducible but can also form their easily oxidized tautomeric enamines. Based on the hypothesis, a new photocatalyst free process needs to be developed based solely on excitation by visible light.

<u>Initial evaluations to test molecular orbital hypothesis for the Lewis-acid activation strategy</u>

To test our hypothesis, initial experiments were conducted (Table S1). No reactivity was observed without activation of the Hantzsch ester (DHP). Intractable reaction mixture was provided under the normally applied photocatlyst activation system, although the Hantzsch ester (DHP) was fully consumed. Intrestingly, the impact of Brønsted and Lewis acid additions was assessed and a significant increase in reactivity was observed through the consumption of **1a** and the formation of α-amino amide **4a**. The results correlated well with our proposed Lewis-acid activation strategy above mentioned.

Table S1: Initial evaluation of carbonyl acylative amination to test molecular orbital hypothesis.

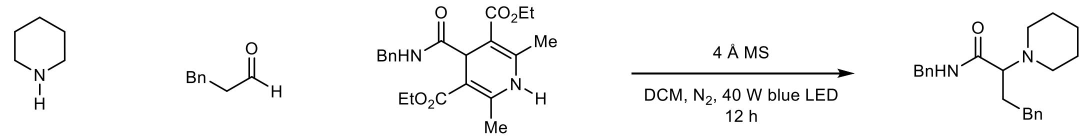

3a (1 equiv) **2a** (1.2 equiv) **1a** (1.2 equiv) **4a**

entry	viriations	Yield	Reagent
1	none	trace	>95%
2	(Ir[dF(CF_3)ppy]$_2$(dtbpy))PF_6 (2 mol%)	10%, new polar spot	0
3	4CzIPN	15%, new polar spot	0
4	TBSOTf (1.2 equiv)	80%, clean system	35%
5	Sc(OTf)$_3$ (20 mol%)	25%, clean system	60%
6	TBSOTf (1.2 equiv) + Sc(OTf)$_3$ (20 mol%)	90% (quant.), clean system	0

UV-visible light absorption study to testify the Lewis-acid activation system

UV-Vis analysis was performed on a Shimadzu UV-1800 spectrophotometer. Experiments were recorded using a quartz cell containing 3 mL of the indicated solution. The UV samples were prepared in DCM (20 mL) as the following concentrations respectively (the solution was stirred vigorously for 20 min before UV-study): Sample **1a** (0.25 mmol/L); **1a**:Sc(OTf)$_3$ (0.25 mmol/L: 0.25mmol/L); **1a**:BF_3.Et_2O (0.25 mmol/L: 0.5mmol/L); **1a**:TBSOTf (0.25 mmol/L: 0.25mmol/L). The UV-visible absorption study indicated that clear bathochromic shift is observed when compared to the parent compound, which also supports the energetic proximity of the σ^* C–CO and HOMO π-orbitals (Fig. S7 and S8). The the tail wavelength absorption of 4-carboxamide-DHP reagent **1a**, 350 nm (max), was considered at 417 nm (Fig. S7), UV-absorption study shows Bathochromic shift via Lewis acid coordination. The tail-wavelength of bathochromic shift, 360 nm (max), was 440 nm.

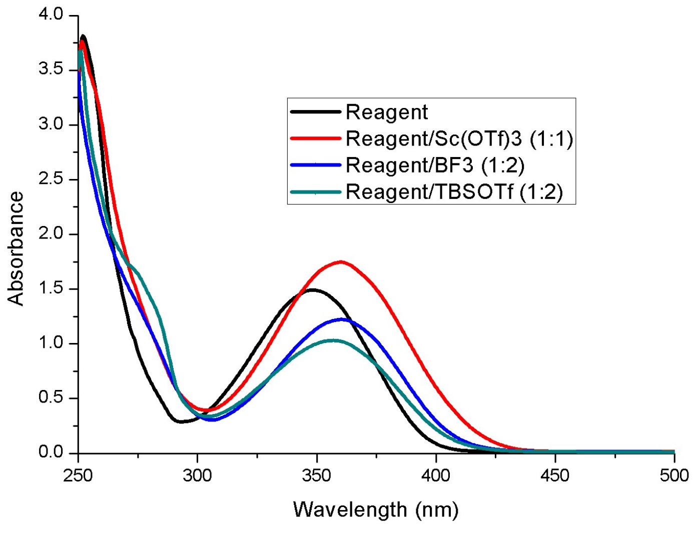

Fig. S7. UV/vis absorption spectra of **1a** complexed TBS-OTf, $BF_3.Et_2O$ and $Sc(OTf)_3$

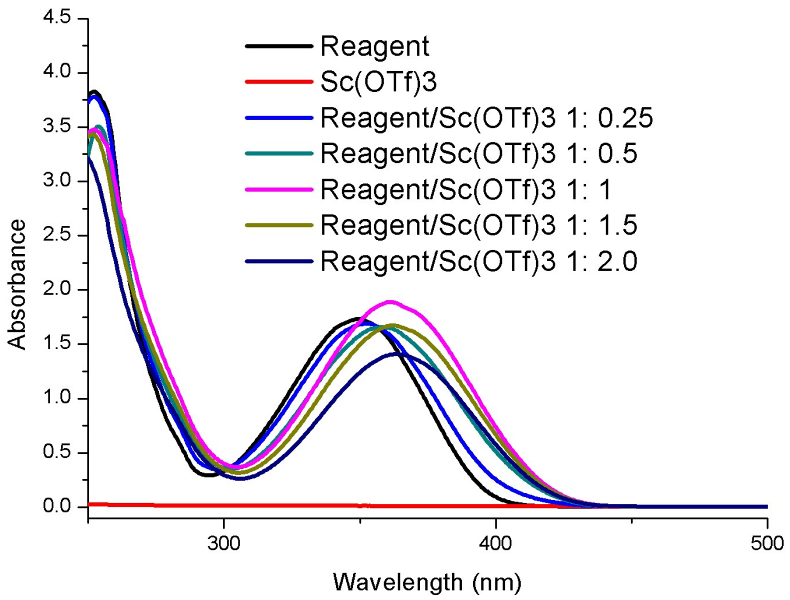

Fig. S8. Detailed information of UV/vis absorption spectra of **1a** complexed $Sc(OTf)_3$

To further vertify the above phenomena, the reaction was conducted under standard conditions with indicated filter. No reaction was observed in the absence of light or using a blue-LED fitted with 455 nm filter, and almost all **1a** was recovered; with a 420 nm filter, 20% of **4a** was formed and 70% of **1a** remained (Table S2). These experiments are consistent with the bathochromic shift of the tail wavelength to 440 nm when **1a** is coordinated to a Lewis acid.

Table S2: Initial light-filtering experiments to evaluate carbonyl acylative amination reactivity to test molecular orbital hypothesis.

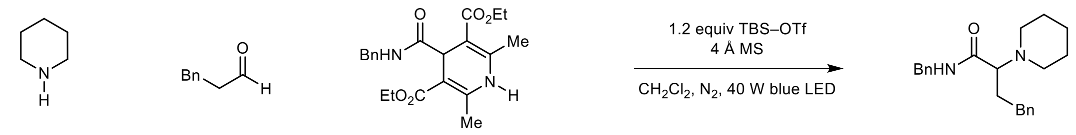

3a (1 equiv) **2a** (1.2 equiv) **1a** (1.2 equiv) **4a**

reactions	product	reagent recovery
, 455 nm filter,12 h	trace	95%
420 nm filter,12 h	20%	70%
without light	0%	>95%

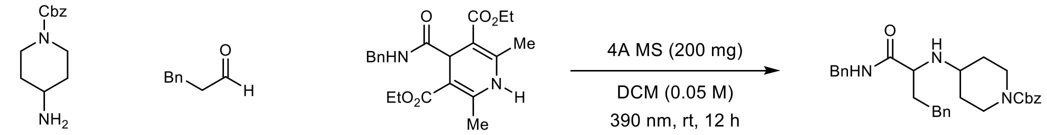

(1 equiv) **2a** (1.2 equiv) **1a** (1.2 equiv) **4g**

$BF_3 \cdot Et_2O$ (1.5 equiv), IY: 85% no activator: 58%

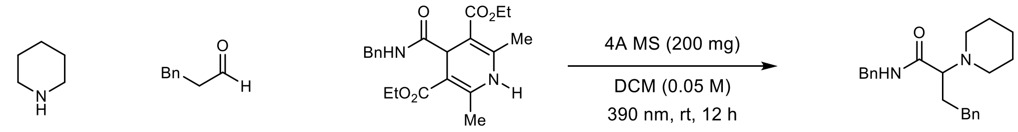

(1 equiv) **2a** (1.2 equiv) **1a** (1.2 equiv) **4a**

TBSOTf (1.2 equiv): 85% no activator: 10%

Reaction optimization and controls

Table S3: Solvents screening of carbonyl acylative amination for carboxyamide Hantzsch ester reagent

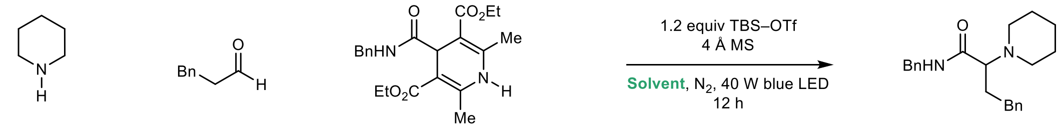

3a (1 equiv) **2a** (1.2 equiv) **1a** (1.2 equiv) **4a**

entry	Solvent	Yield[a]	Reagent[a]
**1**	**DCM**	**90%**	**15%**
2	CH_3CN	trace	100%
3	$PhCF_3$	12%	95%
4	DMF	10%	100%
5	DMSO	15%	80%
6	THF	trace	100%
7	TFE	trace	110%

a) yield was determined by ^{1}H NMR using 1,1,2,2-tetrachloroethane as internal standard.

Table S4: activator screening of carbonyl acylative amination for carboxyamide Hantzsch ester reagent

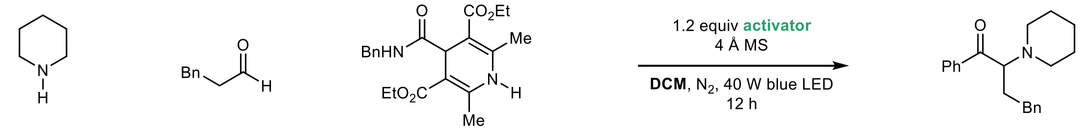

3a (1 equiv) **2a** (1.2 equiv) **1a** (1.2 equiv) **4a**

entry	Activator	Yield[a]	Reagent[a]
**1**	**TBSOTf**	**92%**	**15%**
2	TMSOTf	70%	26%
3	TMSCl	trace	90%
4	$BF_3{\cdot}Et_2O$	75%	6%
5	TfOH	36%	52%
6	TFA	trace	72%
7	$MeSO_3H$	trace	100%
8	Propionic acid	trace	110%
9	HCl	trace	110%
10	none	trace	100%

a) yield was determined by ^{1}H NMR using 1,1,2,2-tetrachloroethane as internal standard.

Table S5: time screening of carbonyl acylative amination for carboxyamide Hantzsch ester reagent

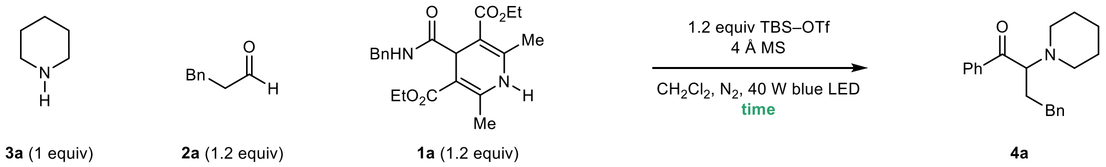

entry	time	product [b]	reagent recovery [a]
1	2 h	27%	78%
2	4 h	43%	50%
3	12 h	92%	15%

a) yield was determined by ^{1}H NMR using 1,1,2,2-tetrachloroethane as internal standard.

Table S6: Activator screening of the carbonyl acylative amination for secondary amines

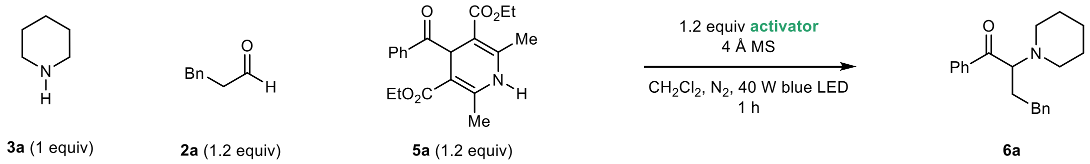

entry	Activator	Yield[a]
1	TBSOTf	72%
2	TMSOTf	70%
3	TMSCl	trace
4	$BF_3 \cdot Et_2O$	40%
5	TfOH	60%
6	TFA	trace
7	AcOH	trace
8	Propionic acid	trace
9	HCl	trace

a) yield was determined by ^{1}H NMR using 1,1,2,2-tetrachloroethane as internal standard.

Table S7: Hantzsch ester reagent purity evaluation

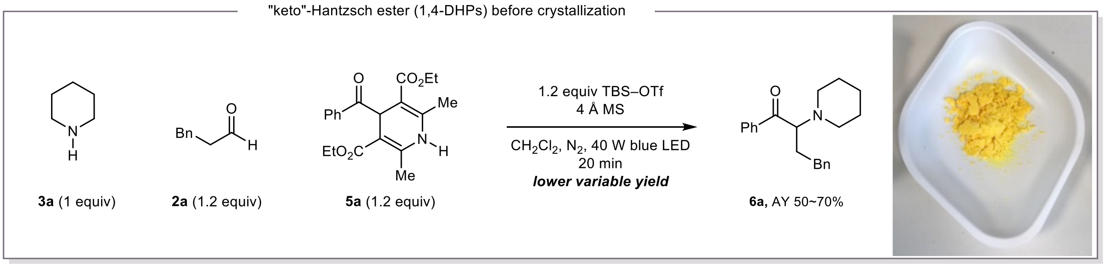

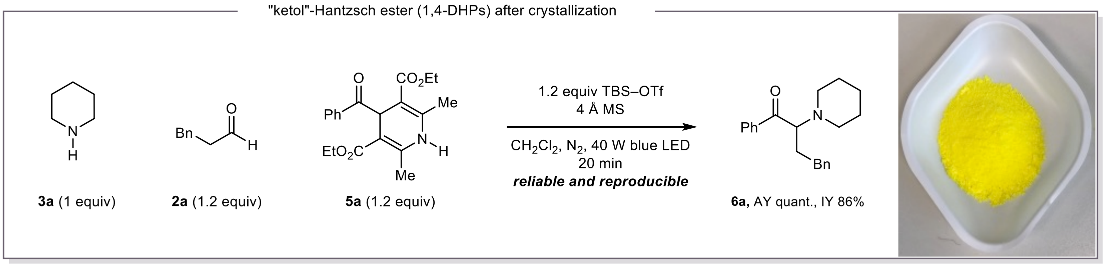

Table S8: Conditions screening of carbonyl acylative amination for primary amines

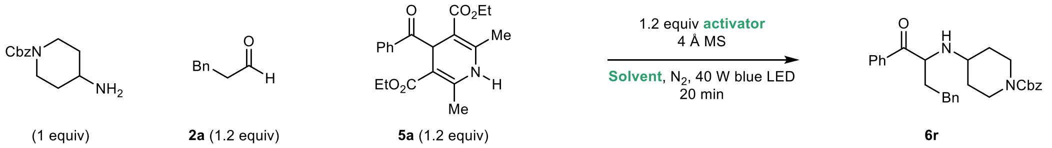

entry	Solvent	Activator	Yield[a]
1	DCM	TMSOTf	60%
2	ACN	TMSOTf	53%
3	PhCF3	TMSOTf	57%
4	Toluene	TMSOTf	36%
5	MTBE	TMSOTf	trace
6	THF	TMSOTf	trace
7	DCM/TFE	TMSOTf	56%
8	DCM/HFIP	TMSOTf	60%
9	DCM	TBSOT	60%
10	DCM	TMSCl	20%
11	DCM	$BF_3 \cdot Et_2O$	76%

a) yield was determined by ^1H NMR using 1,1,2,2-tetrachloroethane as internal standard.

Table S9: Controlling experiments (0.1 mmol scale)

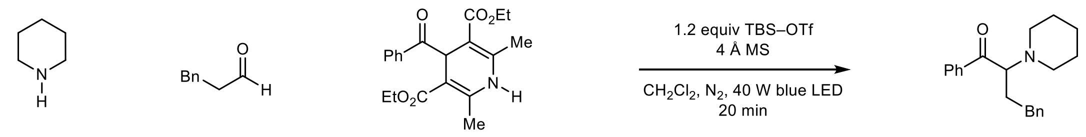

3a (1 equiv) **2a** (1.2 equiv) **5a** (1.2 equiv) **6a**

entry	variation from standard conditions	Product[a]	Reagent recovery[a]
1	no light	0%	98%
2	No TBSOTf	15%	0%
3	Commom DCM	76%	0%
4	Under air	89%	0%
5	no MS	70%	0%
6	Standard conditions	quant.	0%

a) yield was determined by ^{1}H NMR using 1,1,2,2-tetrachloroethane as internal standard.

Fig. S9. Reaction profile of the carbonyl acylative amination (0.1 mmol scale)

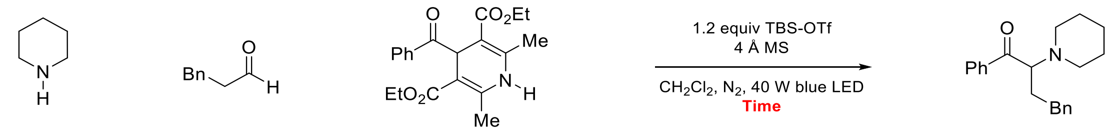

3a (1 equiv) **2a** (1.2 equiv) **5a** (1.2 equiv) **6a**

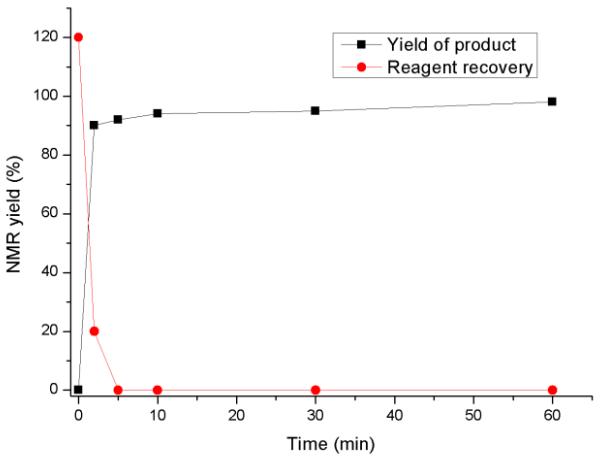

Table S10: Light intensity experiments

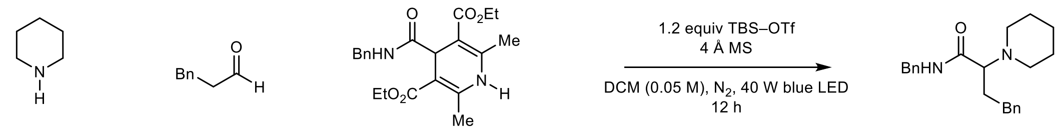

3a (1 equiv) **2a** (1.2 equiv) **1a** (1.2 equiv) **4a**

entry	light intensity	Yield[a]	Reagent[a]
1	25%	50%	50%
2	50%	70%	35%
3	75%	80%	30%
4	100%	80%	30%

Synthesis of Hantzsch ester reagent

➢ **The synthesis of keto-Hantzsch ester reagent (DHP)**

Glyoxal hydrates for the synthesis of reagent were purchased from Acros Organics, and other glyoxal hydrates were prepared using literature protocols via the Riley oxidation) and were used directly for the next step. Reagents of **5a, 5an-5ar** and **5at-5av** were known compounds and synthesized as the following modified procedure according to reported literature. Data were consistent with the literature[53,54].

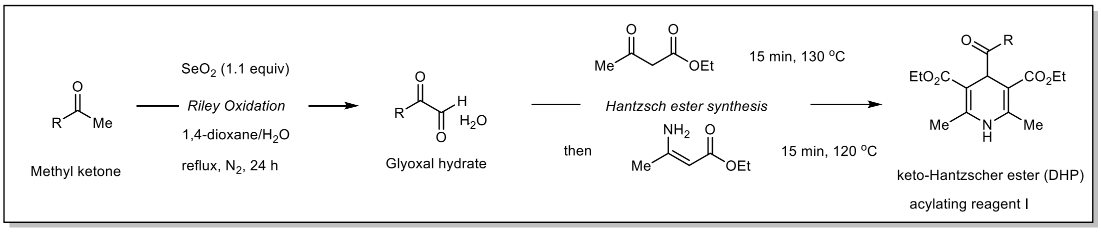

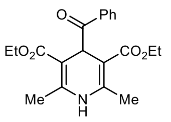

5a

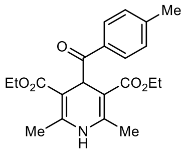

5an

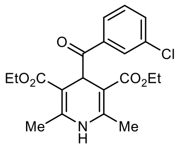

5ao

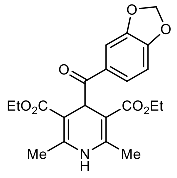

5ap

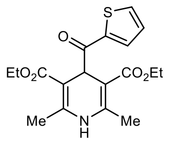

5aq

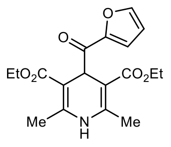

5ar

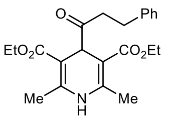

5as

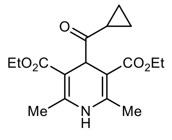

5at

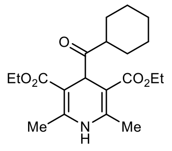

5au

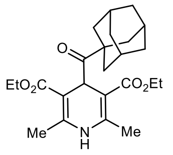

5av

General Procedure for the Preparation of Glyoxal Hydrates via the Riley Oxidation

According to the literature, to a 250 mL round bottom flask, equipped with a magnetic stirrer and a reflux condenser, SeO_2 (1.2 equiv.) was added, followed by 1,4-dioxane/water (5 vol., 10:1 mixture) and the ketone (1.0 equiv.). The reaction mixture was refluxed under argon for 24 h and then cooled to ambient temperature. The suspension was filtered through a plug of celite, and the solvent was removed by rotary evaporator. The residue was dried under high vacuum.

General Procedure for acyl-Hantzsch ester synthesis

According to reported procedure, ethyl acetoacetate (1.0 equiv.) and the glyoxals or glyoxal hydrates (1.0 equiv.) synthesized through the above process were added to a 50 mL round bottom flask equipped with a magnetic stirrer. The mixture was heated to 130 °C and kept under stirring for 15 min, until the condensation reaction was completed (as monitored by TLC analysis). The solution was then cooled to 80 °C. Ethyl 3-aminocrotonate (1.0 equiv.) was slowly added, then the mixture was heated at 120 °C for 15 min and monitored by TLC. After completion, the solution was slowly cooled to ambient temperature and then purified on silica gel (petroleum ether/ethyl acetate, gradient from 3:1 to 1:1), the product was recrystallized from dichloromethane/*n*-Hexane (v/v, 1:10).

Diethyl 2,6-dimethyl-4-(3-phenylpropanoyl)-1,4-dihydropyridine-3,5-dicarboxylate (5as)

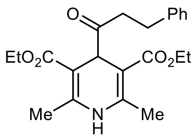

According to the above procedure, the 2-oxo-4-phenylbutanal monohydrate was prepared via riley oxidation from 4-phenylbutan-2-one and followed by the Hantzsch ester synthesis procedure to provide the reagent as pale-yellow solid (2.0 g scale, 10% yield). ^{1}H NMR ($CDCl_3$, 400 MHz): δ 7.28 – 7.24 (m, 2H), 7.18 (td, *J* = 6.2, 1.6 Hz, 3H), 6.04 (s, 1H), 4.83 (s, 1H), 4.18 (qd, *J* = 7.1, 1.1 Hz, 4H), 3.00 (dd, *J* = 8.0, 6.6 Hz, 2H), 2.87 (t, *J* = 7.5 Hz, 2H), 2.29 (s, 6H), 1.28 (t, *J* = 7.1 Hz, 6H). ^{13}C NMR ($CDCl_3$, 100 MHz): δ 210.31, 166.98, 146.16, 141.55, 128.29, 128.26, 125.80, 99.07, 60.09, 46.47, 42.16, 29.60, 19.51, 14.36. HRMS *m/z* (ESI) calcd for $C_{22}H_{28}NO_5$ $(M + H)^+$, 386.1967, found 386.1962. IR Qmax/cm-1 (film): 700, 772, 1044, 1105, 1171, 1231, 1253, 1285, 1369, 1445, 1598, 1720.

➢ The synthesis of 4-Carboxamide-Hantzsch ester reagent (DHP)

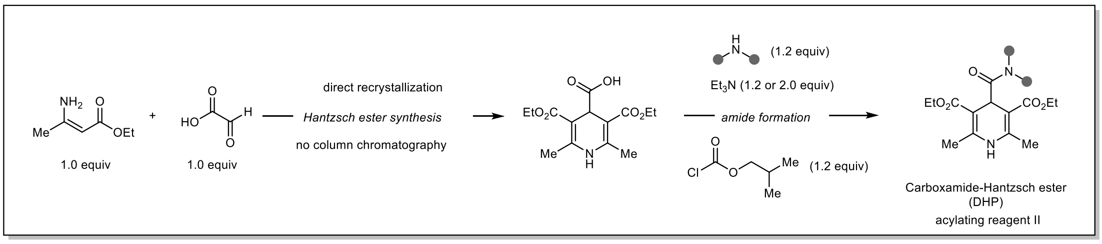

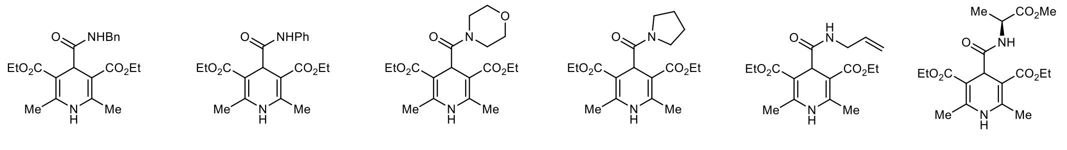

1a **1m** **1p** **1o** **1-s3** **1-s4**

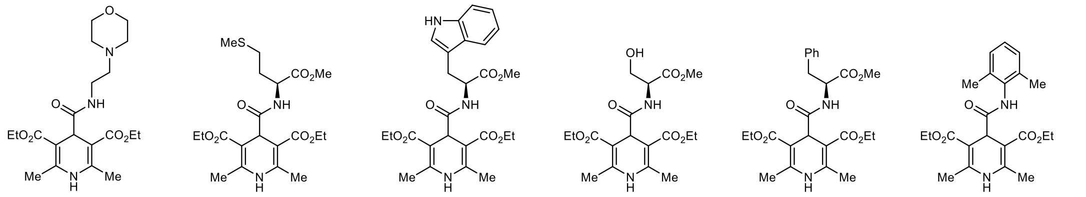

1n **1r** **1q** **1s** **1-s5** **1-7e**

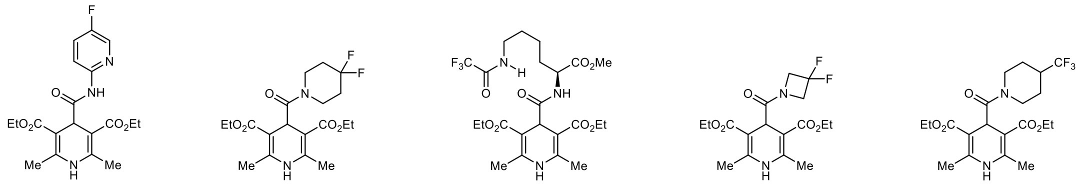

B1 **B2** **B3** **B4** **B5**

General Procedure for 3,5-diethoxycarbonyl-3,6-dimethyl-1,4-dihydroisonicotinic acid synthesis

According to the reported literature[55], glyoxylic acid (10 g, 108 mmol, 1 equiv.) was added portionwise at 0 °C to a solution of ethyl-3-aminocrotonate (27.4 mL, 216 mmol, 2.0 equiv.) in 50 mL of glacial acetic acid. The reaction mixture rapidly became yellow, and a precipitate deposited. After the heat generation had stopped, the reaction mixture was left stirring overnight at room temperature. The solid was collected by filtration and washed with acetic acid and water. The solids were dried overnight under reduced pressure to obtain the pure acid as a white powder. Yield:

50 % (16 g). The spectrum data was in accordance with the literature.

General Procedure for Carbamoyl-Hantzsch ester synthesis

According to the reported literature[56], to a round bottom flask was added carboxylic acid (1 equiv.) in DCM (0.2 M) followed by the addition of triethylamine (1.2 equiv. or 2 equiv. when amine hydrochloride salts are used). The resulting yellow solution was cooled down to 0 °C and isobutylchloroformate (1.2 equiv.) was then added dropwise. After 10 minutes, the mixture was allowed to warm up to ambient temperature and stirred for 20 minutes. Next, the (primary) amine (1.2 equiv.) was added and the resulting solution stirred at ambient temperature for another 60 minutes (overnight for secondary amines). After completion of the reaction, the solution was diluted with DCM, washed with sat. $NaHCO_3$ and water. The organic layers were combined, dried ($MgSO_4$) and concentrated. The remaining residue was purified by flash column chromatography or recrystallized from diethyl ether to provide the desired carbamoyl-Hantzsch ester. All reagents, with the except reagents of **1-**7e, were known compound and synthesized using the procedure described above. The spectrum data was consistent with literature.

Diethyl 4-((2,6-dimethylphenyl)carbamoyl)-2,6-dimethyl-1,4-dihydropyridine-3,5-di carboxylate (1-7e)

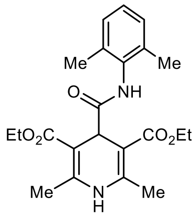

According to the above procedure, the reaction of 2,6-dimethylaniline, carboxylic acid, triethylamine, isobutylchloroformate in DCM provided the desired reagnt as white solid, recrystallization from Et_2O (2.0 g scale, 90% yield). m.p. 240 – 245 °C. ^1H NMR ($CDCl_3$, 400 MHz): δ 8.18 (s, 1H), 8.05 (s, 1H), 7.12-7.04 (m, 3H), 4.88 (s, 1H), 4.47 – 4.13 (m, 4H), 2.17 (d, J = 1.9 Hz, 12H), 1.33 (t, J = 7.1 Hz, 6H). ^{13}C NMR ($CDCl_3$, 100 MHz): δ 173.20, 168.20, 147.84, 135.46, 134.28, 127.93, 126.90, 97.65, 60.19, 41.57, 18.97, 18.17, 14.50; HRMS m/z (ESI) calcd for $C_{22}H_{29}N_2O_5$ $(M + H)^+$, 401.2076, found 401.2072. IR Qmax/cm-1 (film): 1096, 1118, 1213, 1263, 1310, 1326, 1369, 1495, 1659, 1700.

Diethyl 4-((5-fluoropyridin-2-yl)carbamoyl)-2,6-dimethyl-1,4-dihydropyridine-3,5-dicarboxylate (B1)

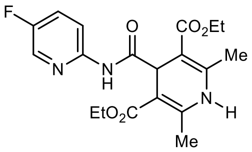

According to the above procedure, the reaction of 5-fluoropyridin-2-amine, carboxylic acid, triethylamine, isobutylchloroformate in DCM provided the desired reagnt **B1** as white solid, (500 mg scale, 40% yield). m.p. 160 – 170 °C. **^{1}H NMR (CDCl$_3$, 400 MHz)**: δ 9.26 (s, 1H), 8.29 – 8.14 (m, 2H), 7.39 (ddd, J = 9.0, 7.7, 3.0 Hz, 1H), 6.44 (s, 1H), 4.77 (s, 1H), 4.34 – 4.18 (m, 4H), 2.34 (s, 6H), 1.32 (t, J = 7.1 Hz, 6H); **^{13}C NMR (CDCl$_3$, 100 MHz)**: δ 172.25, 167.47, 146.80, 135.44 (d, *J* = 25.1Hz), 124.80 (d, *J* = 19.3Hz), 114.25 (d, *J* = 4.1Hz), 98.29, 60.55, 42.64, 19.62, 14.36; **^{19}F NMR (376 MHz, CDCl$_3$)**: δ -134.25; HRMS *m/z* (ESI) calcd for $C_{19}H_{23}N_3O_5F$ (M + H)$^+$, 392.1616, found 392.1618. IR Qmax/cm-1 (film): 1022, 1050, 1096, 1119, 1218, 1265, 1305, 1390, 1470, 1521, 1676, 2980, 3230, 3306.

Diethyl 4-(4,4-difluoropiperidine-1-carbonyl)-2,6-dimethyl-1,4-dihydropyridine-3,5-dicarboxylate: (B2)

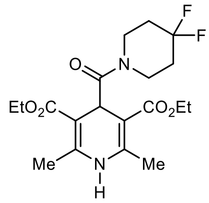

According to the above procedure, the reaction of 4,4-difluoropiperidine hydrochloride salt, carboxylic acid, triethylamine, isobutylchloroformate in DCM provided the desired reagnt as white solid, (1.0 g scale, 50% yield). m.p. 220 – 230 °C. **^{1}H NMR (CDCl$_3$, 400 MHz)**: δ 9.26 (s, 1H), 8.29 – 8.14 (m, 2H), 7.39 (ddd, J = 9.0, 7.7, 3.0 Hz, 1H), 6.44 (s, 1H), 4.77 (s, 1H), 4.34 – 4.18 (m, 4H), 2.34 (s, 6H), 1.32 (t, J = 7.1 Hz, 6H); **^{13}C NMR (CDCl$_3$, 100 MHz)**: δ 172.25, 167.47, 146.80, 135.44 (d, *J* = 25.1Hz), 124.80 (d, *J* = 19.3Hz), 114.25 (d, *J* = 4.1Hz), 98.29, 60.55, 42.64, 19.62, 14.36; **^{19}F NMR (376 MHz, CDCl$_3$)**: δ -134.25; HRMS *m/z* (ESI) calcd for $C_{19}H_{23}N_3O_5F$ (M + H)$^+$, 392.1616, found 392.1618. IR Qmax/cm-1 (film): 1022, 1050, 1096, 1119, 1218, 1265, 1305, 1390, 1470, 1521, 1676, 2980, 3230, 3306.

Diethyl (S)-4-((1-methoxy-1-oxo-6-(2,2,2-trifluoroacetamido)hexan-2-yl)carbamoyl)-2,6-dimethyl-1,4-dihydropyridine-3,5-dicarboxylate: (B3)

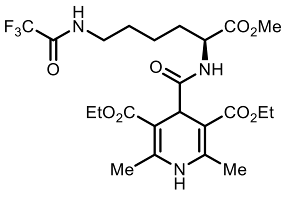

According to the above procedure, the reaction of methyl N^2-(tert-butoxycarbonyl)-N^6-(2,2,2-trifluoroacetyl)-L-lysinate, carboxylic acid, triethylamine, isobutylchloroformate in DCM provided the desired reagnt as yellow foam, (900 mg scale, 45% yield). **^{1}H NMR (CDCl$_3$, 400 MHz** δ 7.83 (s, 1H), 7.38 (t, J = 5.9 Hz, 1H), 7.15 (d, J = 8.3 Hz, 1H), 4.61 (s, 1H), 4.49 (tt, J = 6.3, 3.2 Hz, 1H), 4.28 – 4.11 (m, 4H), 3.68 (s,

3H), 3.39-3.21 (m, 2H), 2.21 (s, 3H), 2.12 (s, 3H), 1.94 – 1.81 (m, 1H), 1.73 – 1.63 (m, 1H), 1.62-1.52 (m, 2H), 1.41-1.32 (m, 2H), 1.31 – 1.24 (m, 6H); **^{13}C NMR ($CDCl_3$, 100 MHz)**: δ 174.39, 172.24, 167.81, 167.77, 157.31 (q, *J* = 36.6Hz), 147.47, 147.22, 115.82 (q, *J* = 285.9Hz), 97.81, 97.31, 60.24, 60.12, 52.16, 51.76, 41.63, 39.48, 31.56, 30.79, 27.82, 22.12, 18.77, 18.74, 14.22, 14.20; **^{19}F NMR (376 MHz, $CDCl_3$)**: δ -76.77; HRMS *m/z* (ESI) calcd for $C_{23}H_{33}N_3O_8F_3$ $(M + H)^+$, 536.2214, found 536.2213. IR Qmax/cm-1 (film): 1027, 1051, 1065, 1075, 1213, 1382, 1393, 1404, 1495, 1663, 1706, 2900, 2987, 3661, 3675, 3686.

Diethyl 4-(3,3-difluoroazetidine-1-carbonyl)-2,6-dimethyl-1,4-dihydropyridine-3,5-dicarboxylate: (B4)

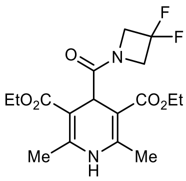

According to the above procedure, the reaction of 3,3-difluoroazetidine hydrochloride salt, carboxylic acid, triethylamine, isobutylchloroformate in DCM provided the desired reagnt as white solid, recrystallization from Et_2O (1.0 g scale, 90% yield). m.p. 170 – 180 °C. **^{1}H NMR ($CDCl_3$, 400 MHz** δ 8.06 (s, 1H), 4.96 (t, J = 12.1 Hz, 2H), 4.56 (d, J = 1.4 Hz, 1H), 4.29 (t, J = 12.0 Hz, 2H), 4.22 (qd, J = 7.1, 1.5 Hz, 4H), 2.21 (s, 6H), 1.32 (td, J = 7.1, 1.5 Hz, 6H); **^{13}C NMR ($CDCl_3$, 100 MHz)**: δ 175.08, 167.29, 147.88, 115.42 (t, *J* = 216.8Hz), 97.73, 62.73(t, *J* = 22.8Hz), 60.11, 59.78 (t, *J* = 22.5Hz), 37.98, 19.31, 14.54; **^{13}F NMR (376 MHz, $CDCl_3$)**: δ -102.40; HRMS *m/z* (ESI) calcd for $C_{17}H_{23}N_2O_5F_2$ $(M + H)^+$, 373.1570, found 373.1566. IR Qmax/cm-1 (film): 1024, 1115, 1208, 1221, 1295, 1304, 1326, 1345, 1370, 1458, 1503, 1632, 1696, 2982, 3096, 3291.

Diethyl 2,6-dimethyl-4-(4-(trifluoromethyl)piperidine-1-carbonyl)-1,4-dihydropyridine-3,5-dicarboxylate: (B5)

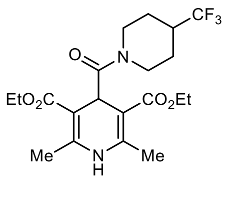

According to the above procedure, the reaction of 4-(trifluoromethyl)piperidine hydrochloride salt, carboxylic acid, triethylamine, isobutylchloroformate in DCM provided the desired reagnt as white solid, PE/Acetone = 3:1, (1.0 g scale, 55% yield). m.p. 200 – 210 °C. **^{1}H NMR ($CDCl_3$, 400 MHz** δ 7.55 (s, 1H), 5.11 (s, 1H), 4.78 (dd, J = 29.0, 13.5 Hz, 2H), 4.32 – 4.10 (m, 4H), 3.10 (t, J = 12.9 Hz, 1H), 2.53 (t, J = 13.1 Hz, 1H), 2.25 (2, 6H), 1.95 (dd, J = 31.1, 13.1 Hz, 2H), 1.78-1.68 (m, 2H), 1.55-1.41 (m, 1H), 1.30 (t, J = 7.2 Hz, 6H); **^{13}C NMR (100 MHz, CDCl3)** δ 174.38, 167.58, 167.47, 147.85, 147.44,

128.23 (q, J = 221.2Hz), 98.83, 98.70, 77.25, 77.00, 76.75, 60.00, 59.81, 45.67, 41.22, 40.65 (q, J = 21.6Hz), 36.56, 25.24, 24.39, 19.48, 19.31, 14.57, 14.5; **^{19}F NMR (376 MHz, $CDCl_3$)**:) δ -74.81; HRMS *m/z* (ESI) calcd for $C_{20}H_{28}N_2O_5F_3$ $(M + H)^+$, 433.1947, found 433.1945. IR Qmax/cm-1 (film): 1007, 1022, 1082, 1148, 1203, 1257, 1306, 1334, 1448, 1499, 1616, 1647, 1677, 2980, 3093, 3220, 3289.

Synthesis of starting material for the study

methyl *N*^{2}-(tert-butoxycarbonyl)-*N*^{6}-(2,2,2-trifluoroacetyl)-L-lysinate

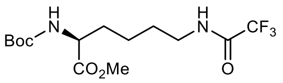

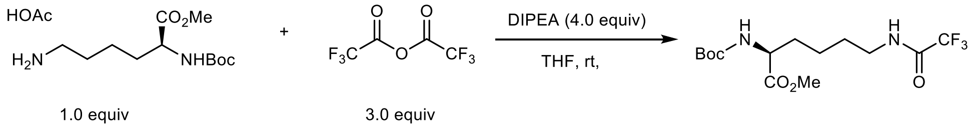

To a solution of Boc-Lys-OMe·AcOH (640 mg, 2.0 mmol) in THF (15 mL) and DIPEA (1.4 mL, 8.0 mmol) was added trifluoroacetic acid anhydride (834 uL, 6.0 mmol) dropwise at 0°C. The solution was warmed to RT and stirred for 5 h. The solvent was removed under vacuum and the mixture was diluted with water and extracted with DCM. The organic layers were washed with water and then combined, dried over Na_2SO_4 and concentrated under reduced pressure. Purification of the crude product by flash chromatography on silica gel with EtOAc/petroleum ether (1:1.5) gave product as a yellow oil (0.7 g, 98%).**^{1}H NMR (400 MHz, $CDCl_3$)**: δ 7.00 (s, 1H), 5.15 (d, J = 8.4 Hz, 1H), 4.27 (q, J = 7.5 Hz, 1H), 3.73 (s, 3H), 3.34 (q, J = 6.8 Hz, 2H), 1.89 – 1.75 (m, 1H), 1.70-1.56 (m, 3H), 1.43 (s, 9H), 1.40 – 1.36 (m, 2H); **^{13}C NMR (101 MHz, $CDCl_3$)**: δ 173.04, 157.33 (q, J = 36.5 Hz) 155.49, 115.81 (q, J = 285.8 Hz), 79.99, 52.96, 52.26, 39.50, 32.32, 28.16, 28.11, 22.34; **^{19}F NMR (376 MHz, CDCl3)**: δ -76.86. The spectrum datas was accordance with the literature[57].

Methyl *N*^{6}-(2,2,2-trifluoroacetyl)-L-lysinate (TFA salt)

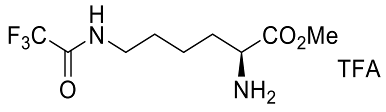

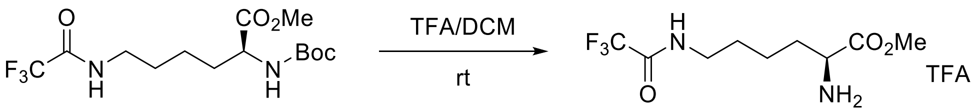

A solution of methyl *N*^{2}-(tert-butoxycarbonyl)-*N*^{6}-(2,2,2-trifluoroacetyl)-L-lysinate (680 mg, 1.9 mmol) in DCM/TFA (40ml/10ml) was stirred at rt for 18 h. Removal of the volatile components under reduced pressure yielded the corresponding amine products quantitatively as their TFA salts. It was used directly without further purification. **^{1}H NMR (400 MHz, CD_3OD)**: δ 4.06 (t, J = 6.4 Hz, 1H), 3.86 (s, 3H), 3.34 –

3.30 (m, 2H), 2.06 – 1.85 (m, 2H), 1.65 (p, J = 7.3 Hz, 2H), 1.58 – 1.39 (m, 2H); **^{13}C NMR (101 MHz, CD_3OD)**: δ 170.91, 160.92 (q, *J* = 38Hz), 159.11 (q, *J* = 36Hz), 118.77 (q, *J* = 36Hz), 117.55 (q, *J* = 284.8Hz), 117.13 (q, *J* = 286.1Hz), 53.81, 53.62, 40.08, 31.06, 29.34, 23.12; **^{19}F NMR (376 MHz, CD_3OD)**: δ -78.38, -78.50. The spectrum datas were accordance with the literature[57].

Mechanistic studies

Light on-off experiments

Considering the click-like characteristcs of acyl-Hantzsch ester involved in the acylation process, we conducted the light on-off reaction using carbamoyl-hantzsch ester reagent.

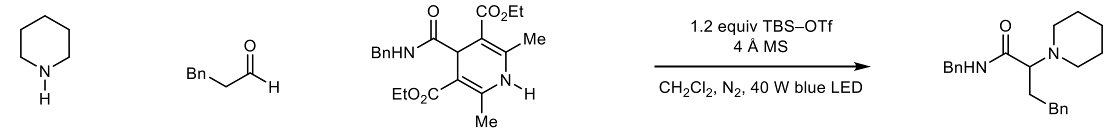

3a (1 equiv) **2a** (1.2 equiv) **1a** (1.2 equiv) **4a**

The model reaction of piperdine (**3a)**, hydrocinnamaldehyde **(2a)** and benzyl-carboxamide-Hantzsch ester (**1a**) using TBSOTf as activator was conducted under standard conditions on 0.1 mmol scale across 4 hours upon intervals of 1 hour, four separated reactions were performed as indicated light excitation conditions.

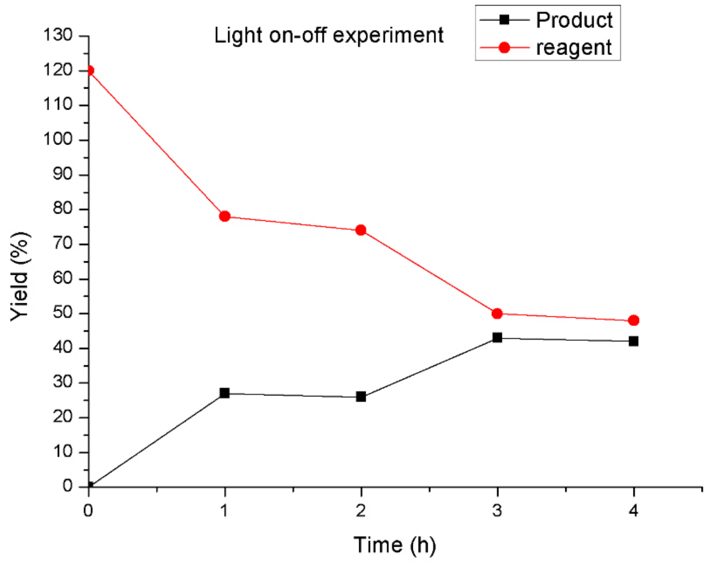

Fig. S10. Light on-off experiments: a) yield was determined by 1H NMR using 1,1,2,2-tetrachloroethane as internal standard; b) isolated yield.

The above results (Fig. S10) showed that the reaction was shut off without the light excitation, and restarted the process after irradiation, indicating that the light is

indispensable for the success of this chemistry. To further verify this conclusion, another variation of light on-off experiments was performed with longer dark (11 hours) conditions (Fig. S11), which demonstrated again that light was requisite to this process. Collectively, these results show that radical chain propagation, if present, is not the major pathway.

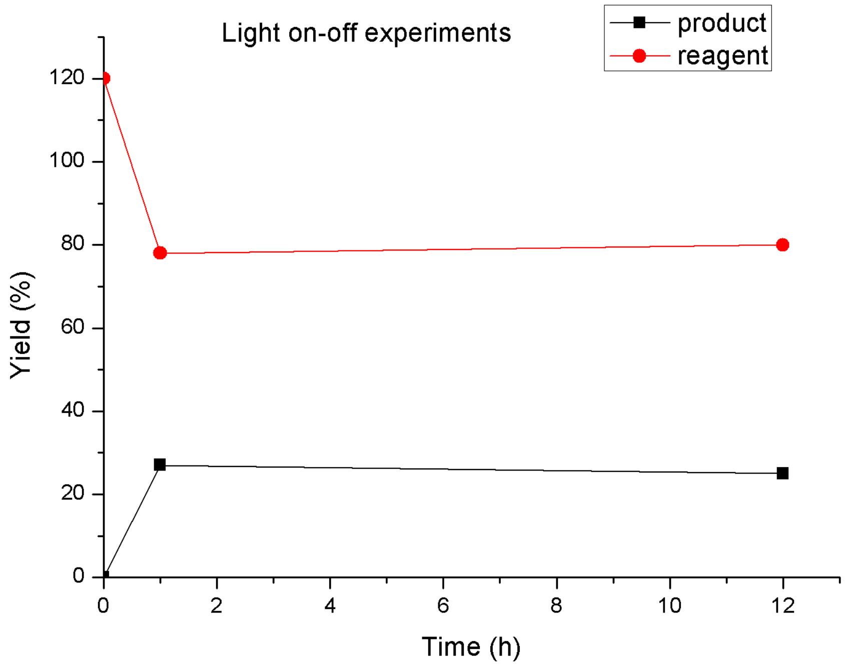

Fig. S11. Light on-off experiments: a) yield was determined by 1H NMR using 1,1,2,2-tetrachloroethane as internal standard; b) isolated yield.

Radical clock experiments

Aminium radical cation formation

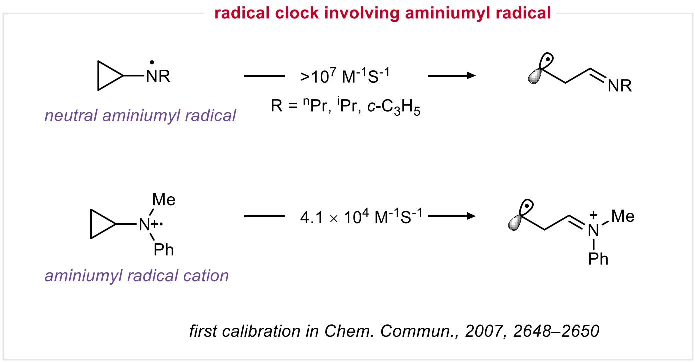

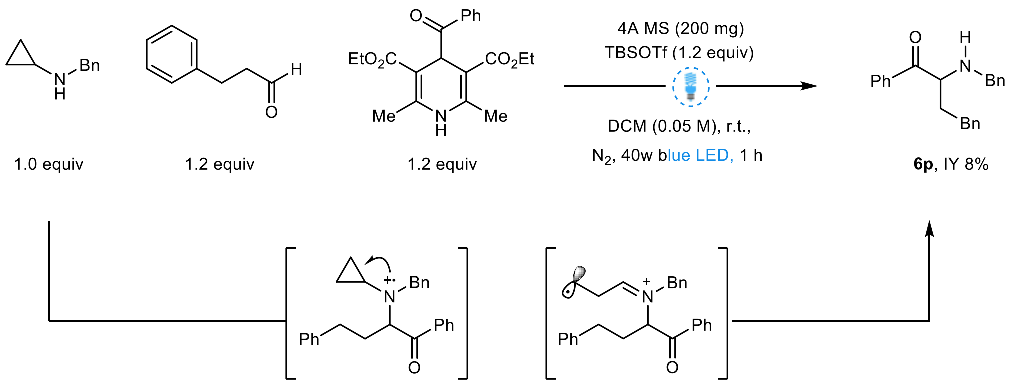

2-(benzylamino)-1,4-diphenylbutan-1-one (6p)

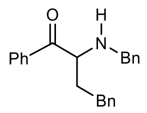

According to general procedure A, the reaction of N-benzylcyclopropanamine (14.7 mg, 0.1 mmol), hydrocinnamaldehyde **2a** (16 uL, 0.12 mmol), diethyl 4-benzoyl-2,6-dimethyl-1,4-dihydropyridine-3,5-dicarboxylate **5a** (44 mg, 0.12 mmol), 4 Å molecular sieve (200 mg), TBSOTf (28 uL, 0.12 mmol) in DCM (2 mL). The crude reaction was purified by flash column chromatography to provide the ring opening product **6p** (3 mg, 8%), indicating the aminium radical cation formation. **^{1}H NMR (400 MHz, $CDCl_3$)**: δ 7.72 – 7.67 (m, 2H), 7.61 – 7.53 (m, 1H), 7.43 – 7.20 (m, 10H), 7.11 – 7.05 (m, 2H), 4.17 (dd, J = 8.8, 3.5 Hz, 1H), 3.88 (d, J = 12.9 Hz, 1H), 3.58 (d, J = 12.9 Hz, 1H), 3.01 – 2.73 (m, 2H), 2.22 (brs, 1H), 2.07 – 1.89 (m, 1H), 1.79-1.70 (m, 1H); **^{13}C NMR (101 MHz, $CDCl_3$)**: δ 203.45, 141.48, 140.30, 135.72, 133.26, 128.73, 128.67, 128.56, 128.37, 128.16, 127.03, 125.95, 60.46, 52.35, 35.71, 32.14; HRMS m/z (ESI) calcd for $C_{23}H_{24}NO$ $(M + H)^+$, 330.1858, found 330.1856. IR Qmax/cm-1 (film): 697, 715, 749, 1027, 1057, 1066, 1249, 1381, 1393, 1452, 1495, 1680, 2900.

α-amino radical formation (EDA complex)

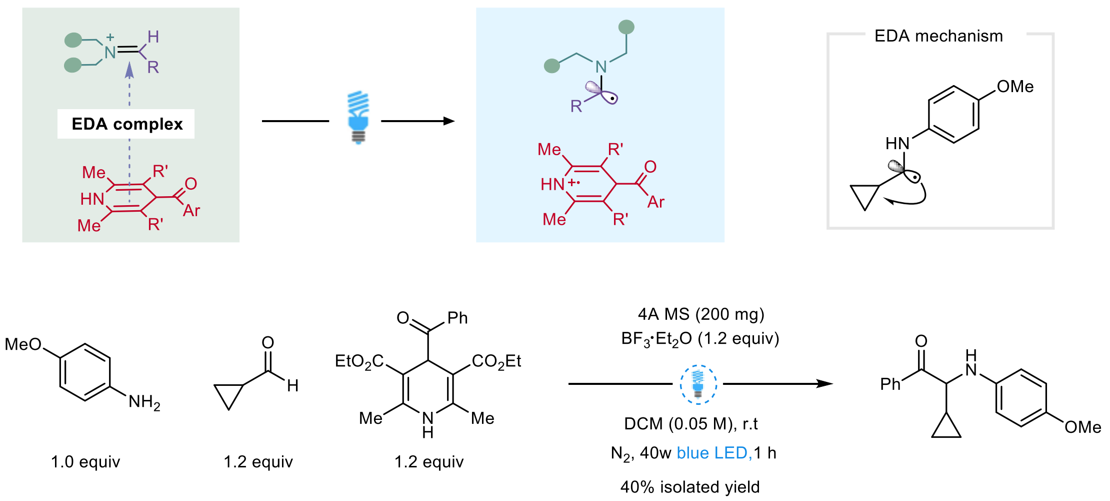

2-cyclopropyl-2-((4-methoxyphenyl)amino)-1-phenylethan-1-one

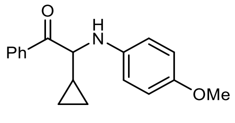

According to general procedure A, the reaction of 4-methoxyaniline (12.3 mg, 0.1 mmol), cyclopropanecarbaldehyde (8.4 mg, 0.12 mmol), diethyl 4-benzoyl-2,6-dimethyl-1,4-dihydropyridine-3,5-dicarboxylate (44 mg, 0.12 mmol), 4 Å molecular sieve (200 mg), $BF_3.Et_2O$ (19 uL, 0.15 mmol) in DCM (2 mL). The crude reaction was purified by flash column chromatography (petroleum ether/acetone = 10 :1) to provide the ring remaining product (11.5 mg, 41%), potentially ruling out the EDA complex formation.**^{1}H NMR (400 MHz, CDCl$_3$)**: δ 8.06 (d, *J* = 6.9 Hz, 2H), 7.66 – 7.59 (m, 1H), 7.53 (t, *J* = 7.6 Hz, 2H), 6.78 (d, *J* = 8.9 Hz, 2H), 6.68 (d, *J* = 8.9 Hz, 2H), 4.83 (d, *J* = 5.6 Hz, 1H), 4.46 (brs, 1H), 3.75 (s, 3H), 1.25 – 1.17 (m, 1H), 0.53-0.47 (m, 2H), 0.45 – 0.31 (m, 2H); **^{13}C NMR (101 MHz, CDCl$_3$)**: δ 199.94, 152.44, 141.89, 135.60, 133.42, 128.76, 128.55, 114.93, 114.86, 60.23, 55.75, 14.02, 3.03, 1.48; HRMS *m/z* (ESI) calcd for $C_{18}H_{20}NO_2$ (M + H)$^+$, 282.1494, found 282.1493. IR Qmax/cm-1 (film): 693, 820, 1036, 1237, 1447, 1511, 1682, 2831, 2929, 3004, 3375.

Acyl radical trapping experiments

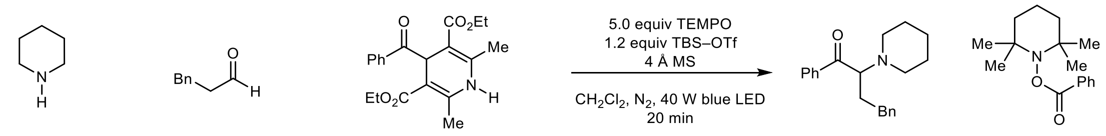

3a (1 equiv) **2a** (1.2 equiv) **5a** (1.2 equiv) **6a**, 0% 61%

2,2,6,6-tetramethylpiperidin-1-yl benzoate

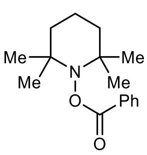

According to general procedure A, the reaction of piperidine **3a** (10 uL, 0.1 mmol), hydrocinnamaldehyde **2a** (16 uL, 0.12 mmol), diethyl 4-benzoyl-2,6-dimethyl-1,4-dihydropyridine-3,5-dicarboxylate **5a** (44 mg, 0.12 mmol), TEMPO (78.2 mg, 0.5 mmol), 4 Å molecular sieve (200 mg), TBSOTf (28 uL, 0.12 mmol) in DCM (2 mL). The carbonyl acylated amination was inhibited and the crude reaction was purified by flash column chromatography to provide the 2,2,6,6-tetramethylpiperidin-1-yl benzoate (16 mg, 61%).**^{1}H NMR (400 MHz, CDCl$_3$)**: δ 8.12 (d, *J* = 7.1 Hz, 2H), 7.61 (t, *J* = 7.4 Hz, 1H), 7.50 (t, *J* = 7.6 Hz, 2H), 1.88 – 1.71 (m, 3H), 1.67 – 1.60 (m, 2H), 1.50 (dt, *J* = 10.1, 3.7 Hz, 1H), 1.32 (s, 6H), 1.16 (s, 6H); **^{13}C NMR (101 MHz, CDCl$_3$)**: δ 165.99, 132.46, 129.36, 129.18, 128.07, 60.01, 38.68, 31.59, 20.47, 16.63; HRMS *m/z* (ESI) calcd for $C_{16}H_{24}NO_2$ (M + H)$^+$, 262.1807, found 262.1808. IR Qmax/cm-1 (film): 719, 1026, 1046,

1062, 1083, 1238, 1252, 1452, 1740, 2940, 2973.

UV-visible light absorption studies of Carbonyl Acylative Amination (CAcylA) for ketone-DHP reagent

As shown below (Table S10), the reaction was conducted under standard conditions with indicated filter. The light-filter experiments using 455 nm filter indicated that the reaction works for Acyl-HEH variation, albeit in low efficiency. Moreover, the reaction works well for Acyl-HEH reagent with 420 nm filter.

Table S10: Light-filtering experiments to evaluate carbonyl acylative amination reactivity.

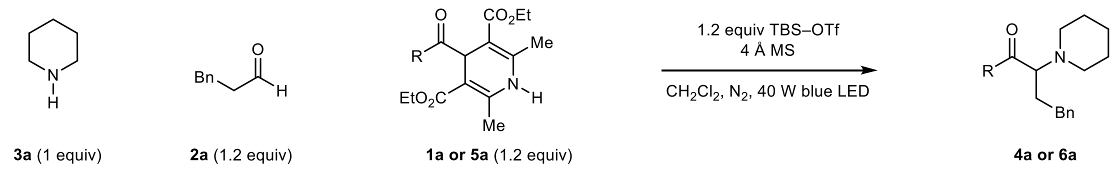

reactions	product	reagent recovery
R= Ph, 455 nm filter, 10 min	24%	43%
R = Ph, 420 nm filter, 10 min	82%	0
without light	0%	>95%

To clarify the above phenomena, a series of UV-vis light experiments involving all combinations of reagents have been conducted. UV-Vis analysis was performed on a Shimadzu UV-1800 spectrophotometer. Experiments were recorded using a quartz cell containing 3 mL of the indicated solution. First, the UV-vis light absorption experiments of the Hantzsch ester reagents have been conducted. As shown in Fig. S12, the tail wavelength absorption of Acyl-HEH reagent, 384 nm (max), was considered at 460 nm. So, it could explain well the result of poor yield using 455 nm filter and good yield with 420 nm filters. Entry 4 and entry 5 have already been explained in Table S2, here is the whole comparison table.

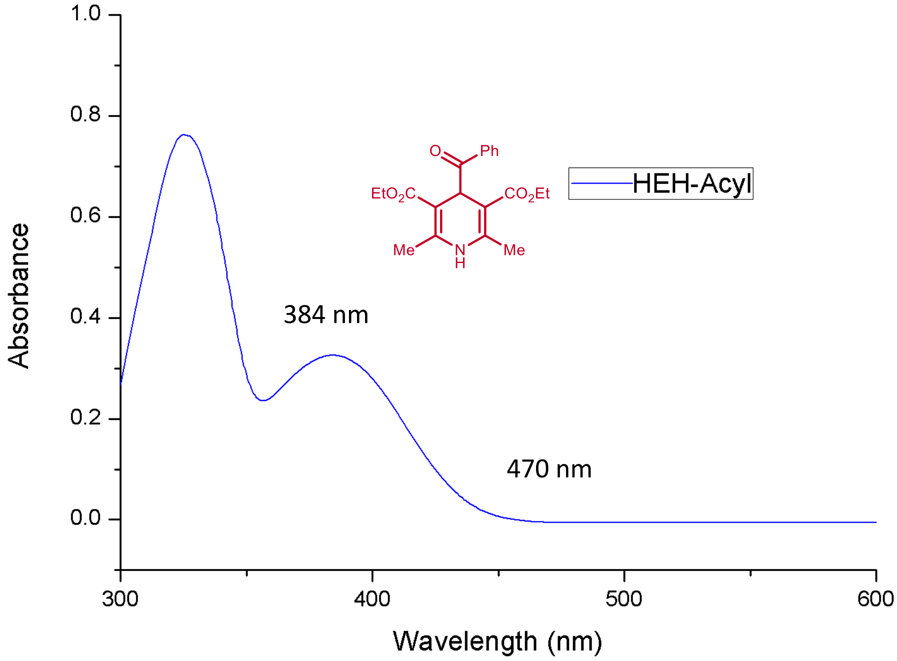

Fig. S12. UV-visble absorption spectrum of Acyl-HEH reagent.

To elucidate whether EDA complex was formed in this process, UV-vis light absorption experiments of the reaction system were conducted. As shown in Fig. S13 and S14, the imnium (piperidine, hydrocinnamaldehyde and TBSOTf, 1:1.2:1 ratio) and enamine (piperidine, hydrocinnamaldehyde; 1:1.2 ratio) combined with the reagent were conducted respectively. However, new band and wavelength shift was not observed, ruling out the formation of EDA complex in the reaction.

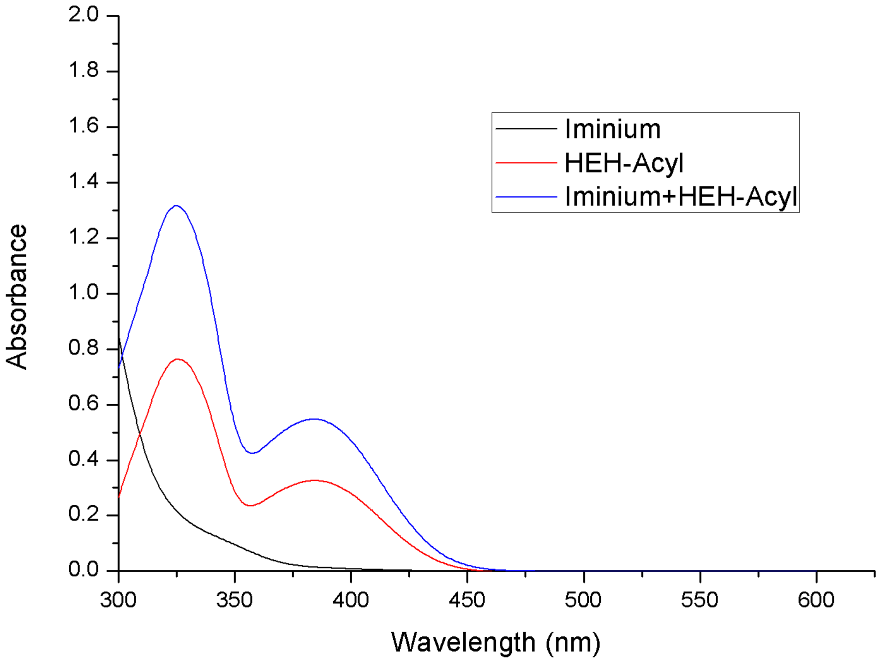

Fig. S13. UV-visble absorption spectrum of iminium, Acyl-HEH reagent and the mixture of iminium and

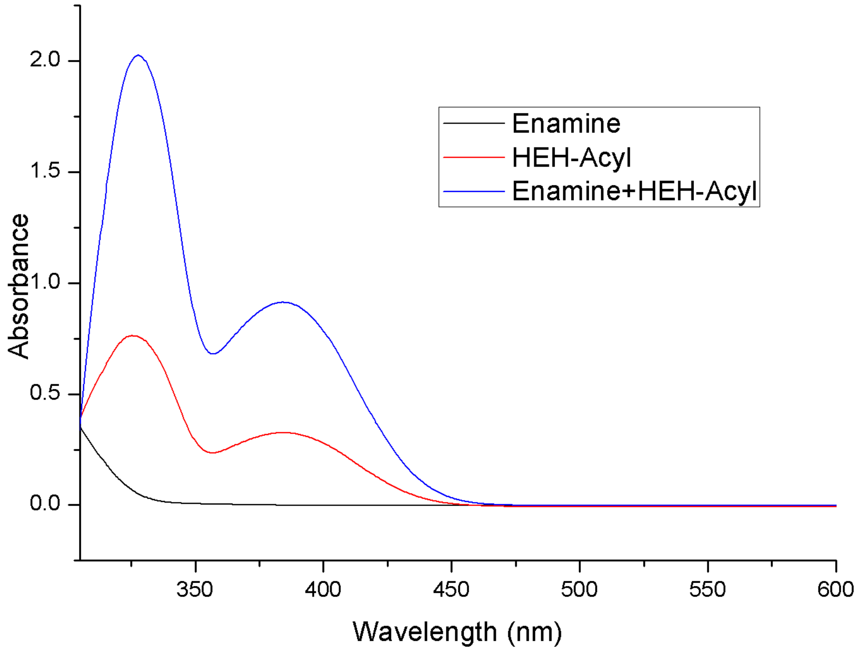

Acyl-HEH reagent

Fig. S14. UV-visble absorption spectrum of enamine, Acyl-HEH reagent and the mixture of enamine and Acyl-HEH reagent reagent.

All the above UV-Vis spectrum experiments should explain the light-dependent character of this chemistry. The capacity of Carbonyl Acylative Amination reactivity is attributed to the direct light absorption of the Hantzsch ester reagent for the polarity-well-matched acyl-radical coupling with iminium, achieving the exquisite selectivity and reactivity control of radical species.

Kinetic studies of Carbonyl Acylative Amination (CAcylA)

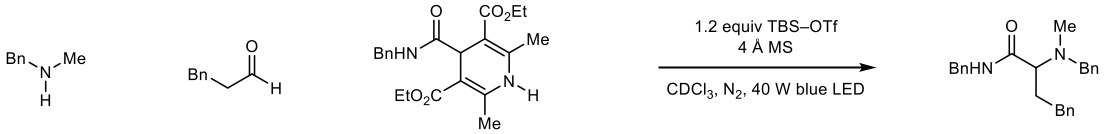

1f(1 equiv) **2a** (1.2 equiv) **1a** (1.2 equiv) **4f**

All samples and reactions for the kinetic experiments were collected and conducted according to the following procedures. An oven dried vial (Biotage Microwave Process Vial 10–20 mL, equipped with gas-tight septum) was charged with a stir bar, 4Å MS (100 mg) and HEH reagent. The vial was sealed, evacuated, and backfilled three times with 1 atm of N_2. $CDCl_3$ (1 mL) was added followed by the addition of amine, aldehyde, TBSOTf and 1,1,2,2-tetrachloroethane (internal standard) using a microsyringe. The Kessil lamp was positioned 5 cm from the vial along with a desk-fan for cooling. The reaction mixture was irradiated using a 40 W blue LED lamp (Kessil A160WE Tuna Blue) with vigorous stirring at room temperature. A t_0 aliquot was then taken and the tube was sealed and continued to stir under light. For example: the mixture was then taken

out of the photo fumehood, and a 30 μL aliquot was removed from the mixture. The tube was resealed and returned to photoexcitation. The aliquots were analyzed by ^1H NMR spectroscopy and concentrations were determined by integration relative to the 1,1,2,2-tetrachloroethane internal standard. Initial reaction rates were subsequently determined by measuring product formation at low conversion.

Reaction rate order in amine (Fig.s S15-S19): zero order

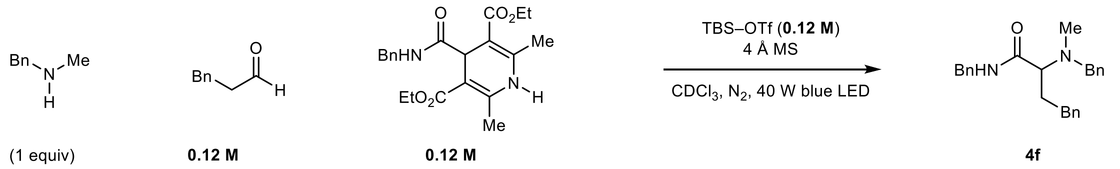

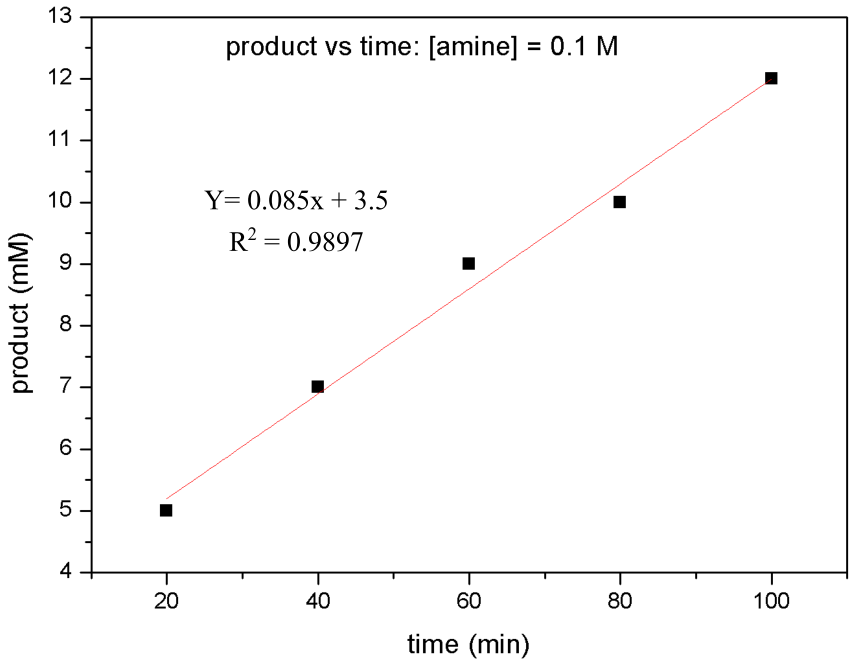

Fig. S15. Rate of product formation as a function of time when [amine] = 0.10 M.

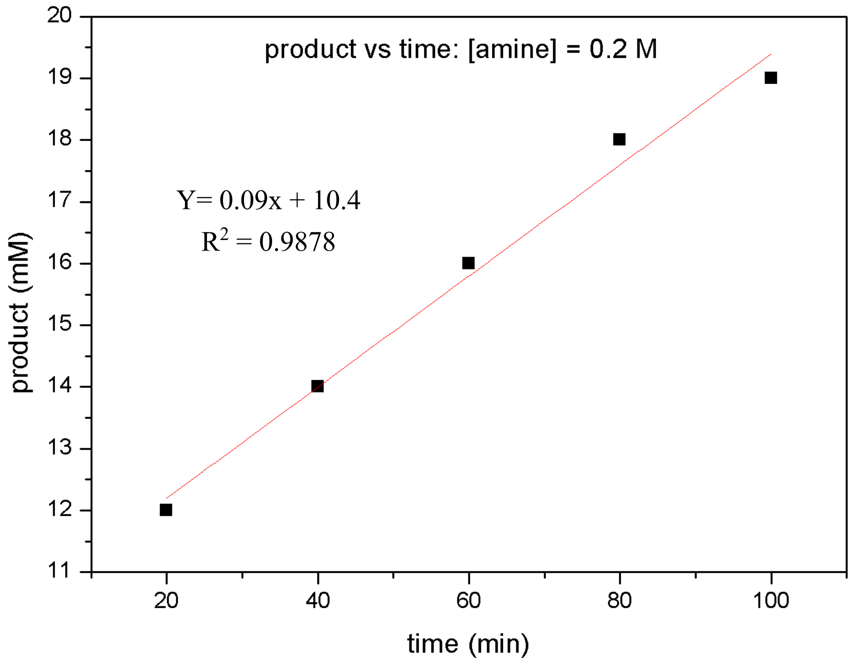

Fig. S16. Rate of product formation as a function of time when [amine] = 0.20 M.

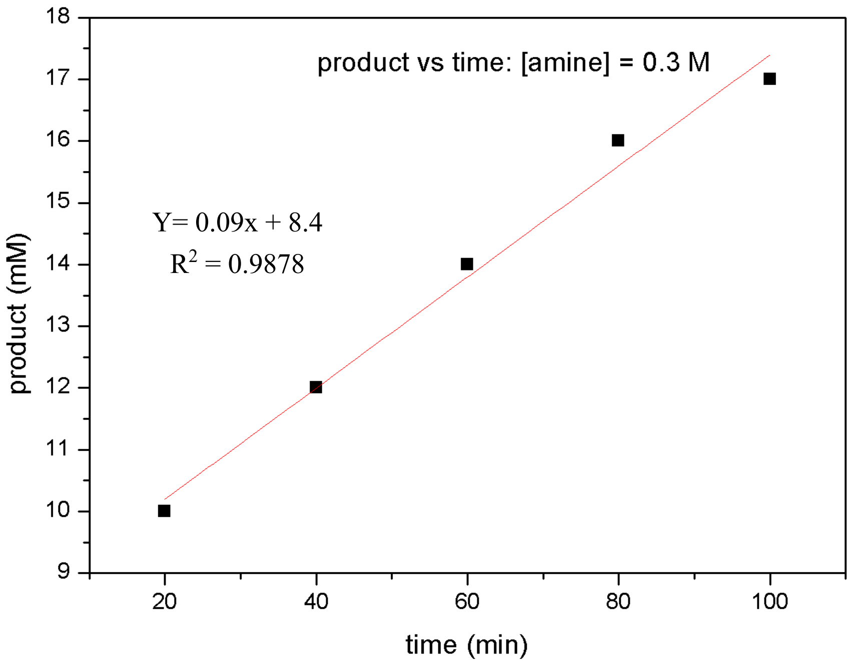

Fig. S17. Rate of product formation as a function of time when [amine] = 0.30 M.

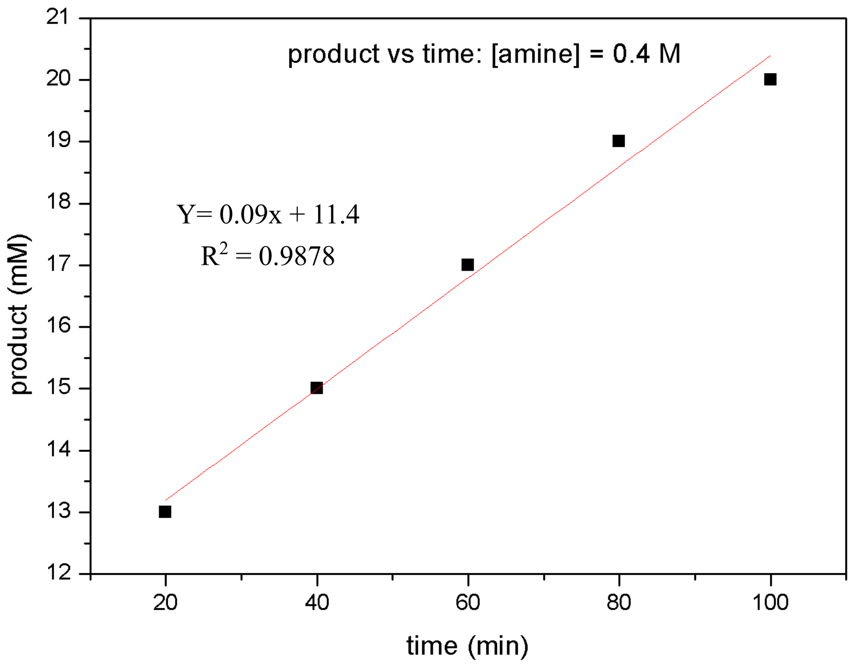

Fig. S18. Rate of product formation as a function of time when [amine] = 0.40 M.

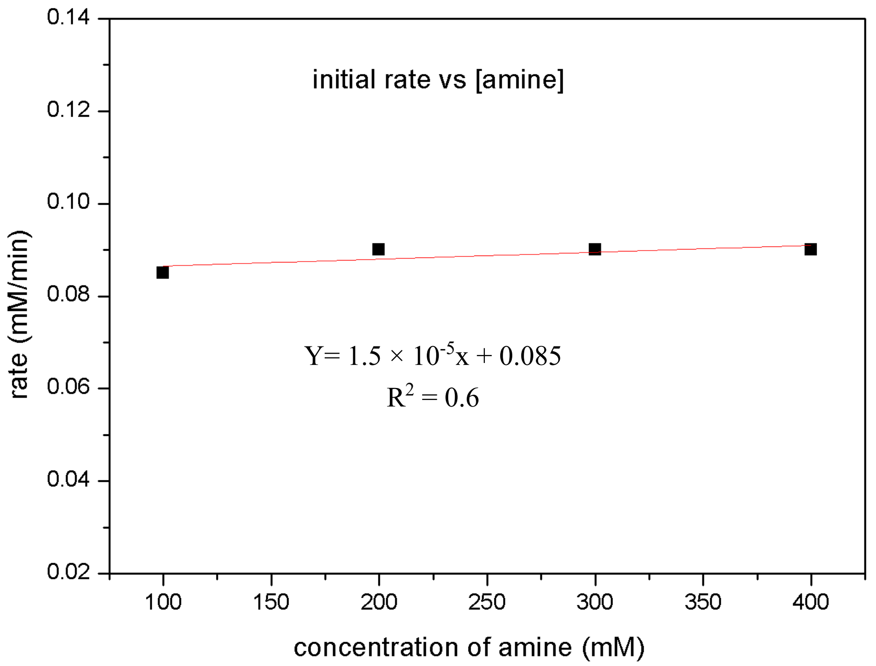

Fig. S19. Initial rate regarding the amine

Reaction rate order in aldehyde (Fig.s S20-S24): zero order

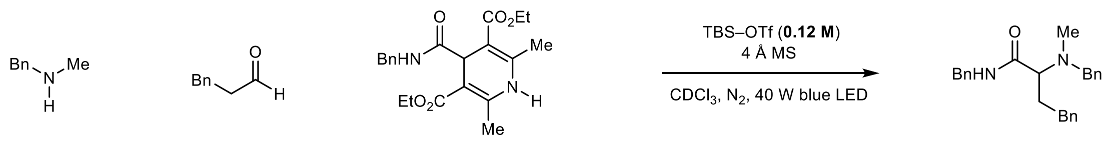

0.1 M **0.12 M** **4f**

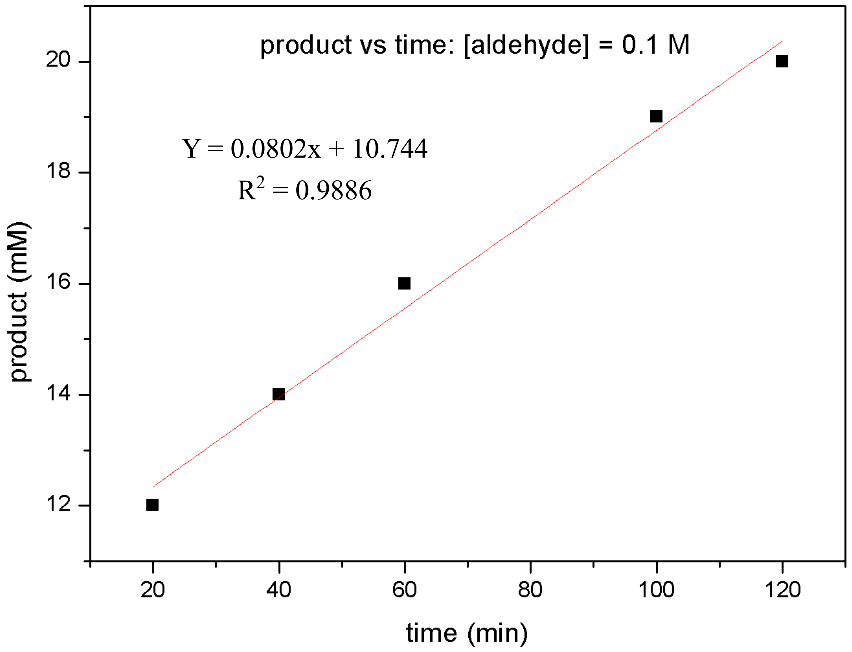

Fig. S20. Rate of product formation as a function of time when [aldehyde] = 0.10 M.

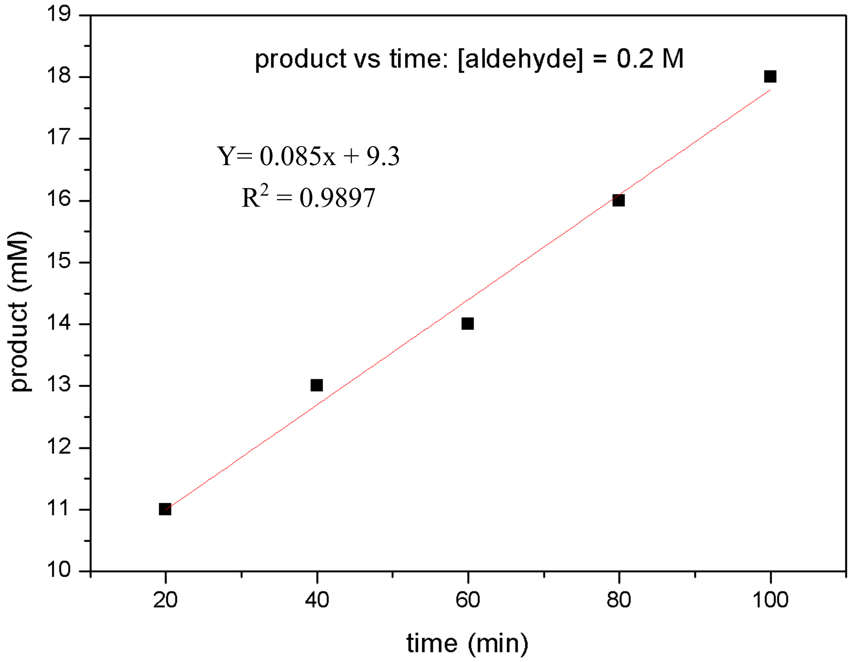

Fig. S21. Rate of product formation as a function of time when [aldehyde] = 0.20 M.

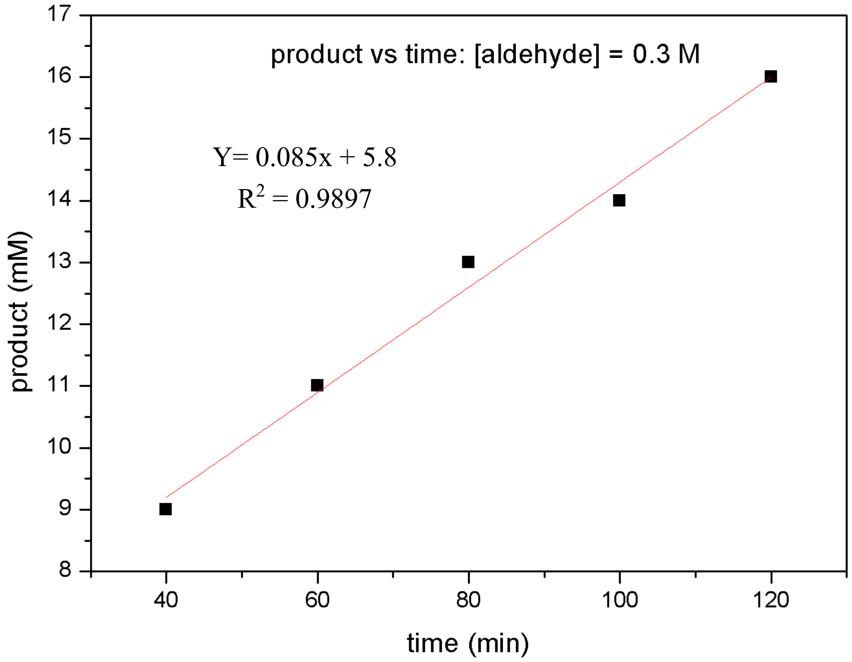

Fig. S22. Rate of product formation as a function of time when [aldehyde] = 0.30 M.

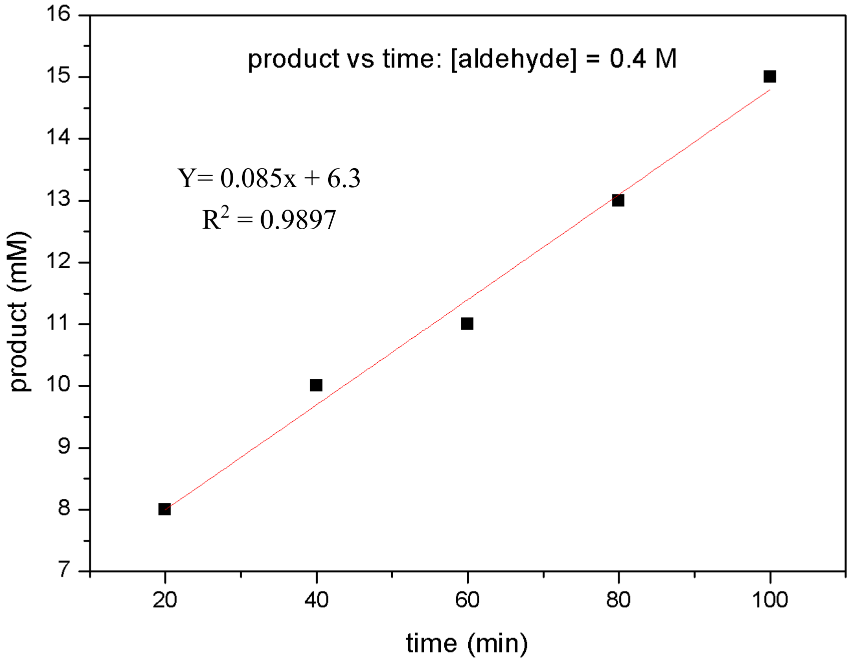

Fig. S23. Rate of product formation as a function of time when [aldehyde] = 0.40 M.

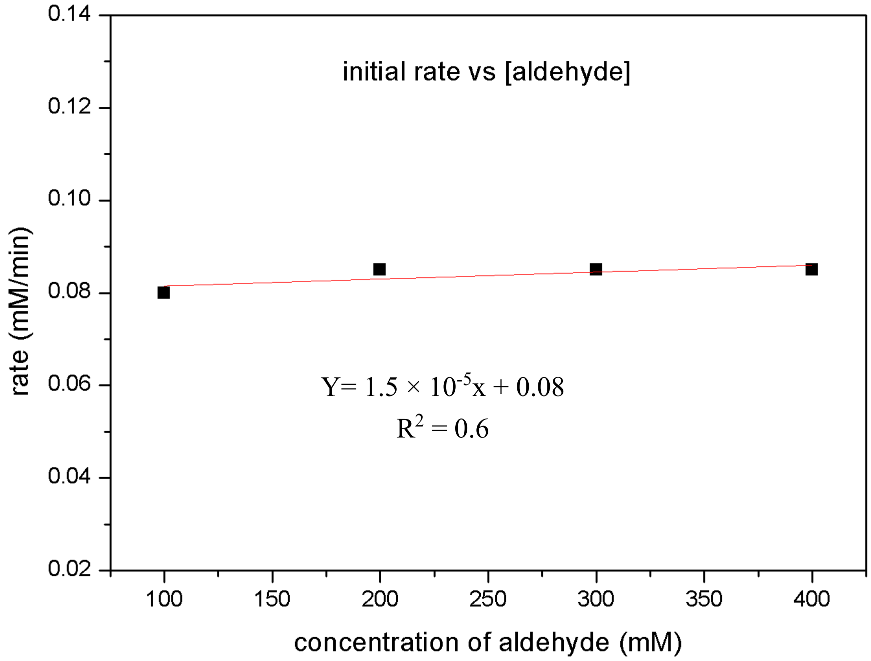

Fig. S24. Initial rate regarding the aldehyde.

Order in Hantzsch ester reagent (Fig.s S25-S29): first order

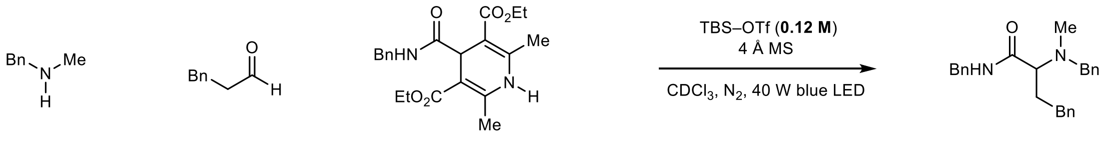

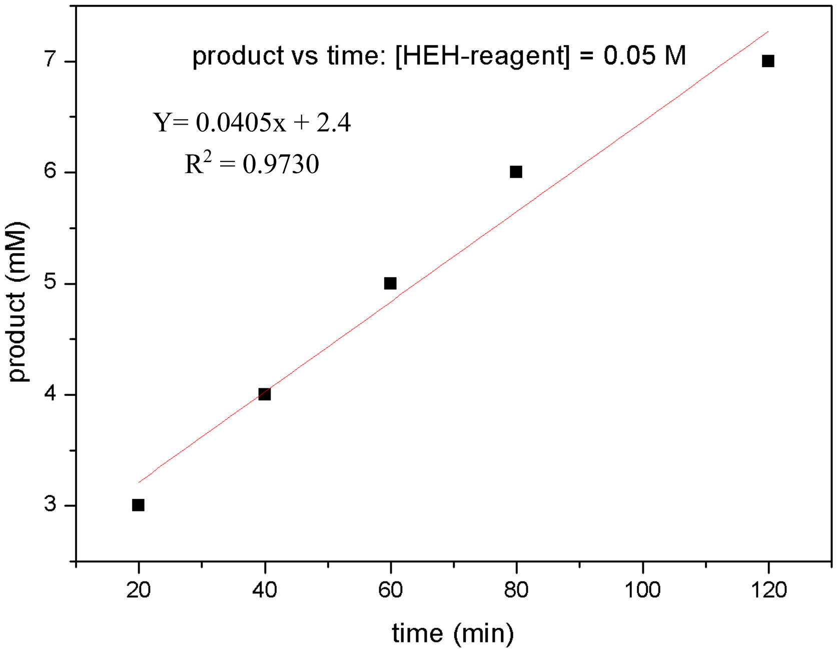

Fig. S25. Rate of product formation as a function of time when [HEH-reagent] = 0.05 M.

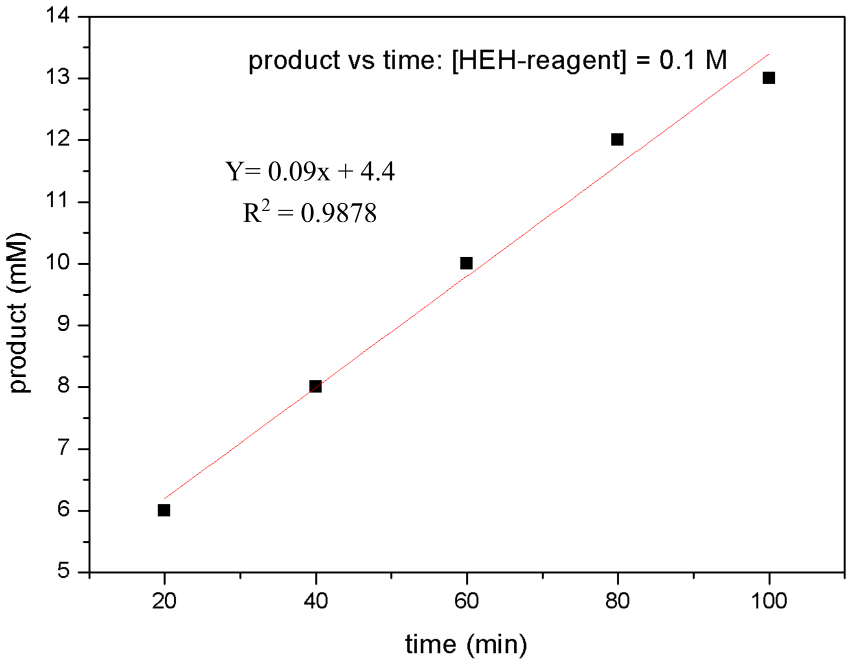

Fig. S26. Rate of product formation as a function of time when [HEH-reagent] = 0.10 M.

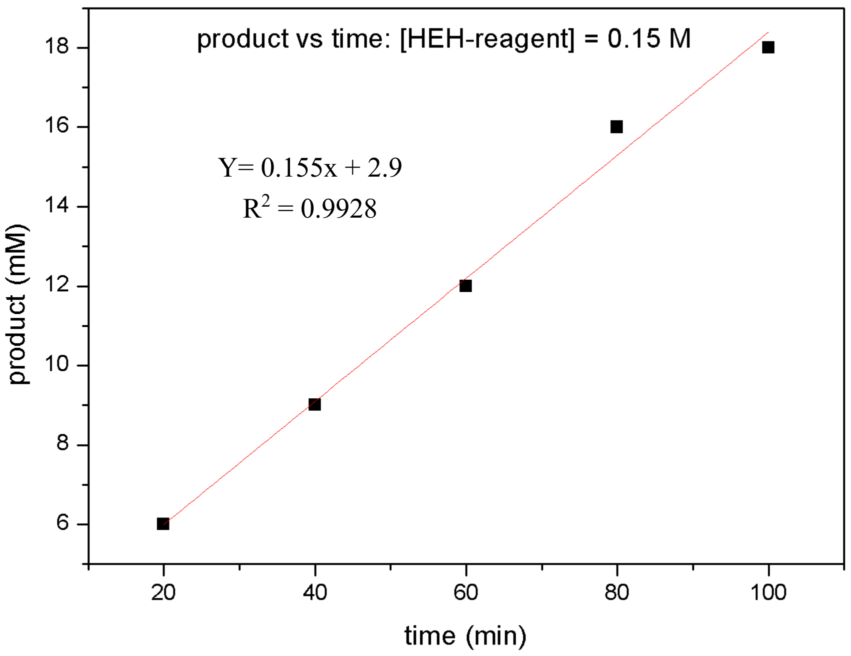

Fig. S27. Rate of product formation as a function of time when [HEH-reagent] = 0.15 M.

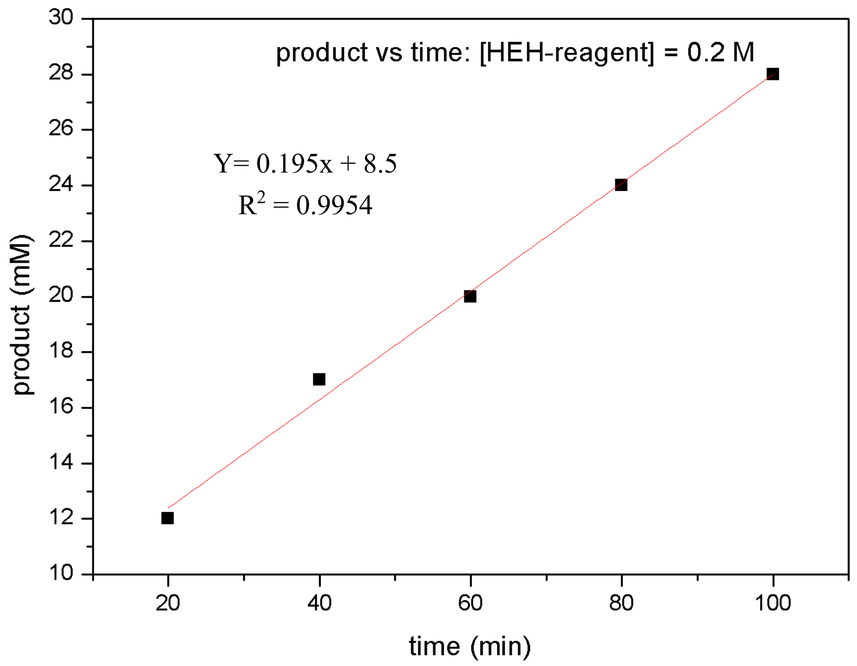

Fig. S28. Rate of product formation as a function of time when [HEH-reagent] = 0.20 M.

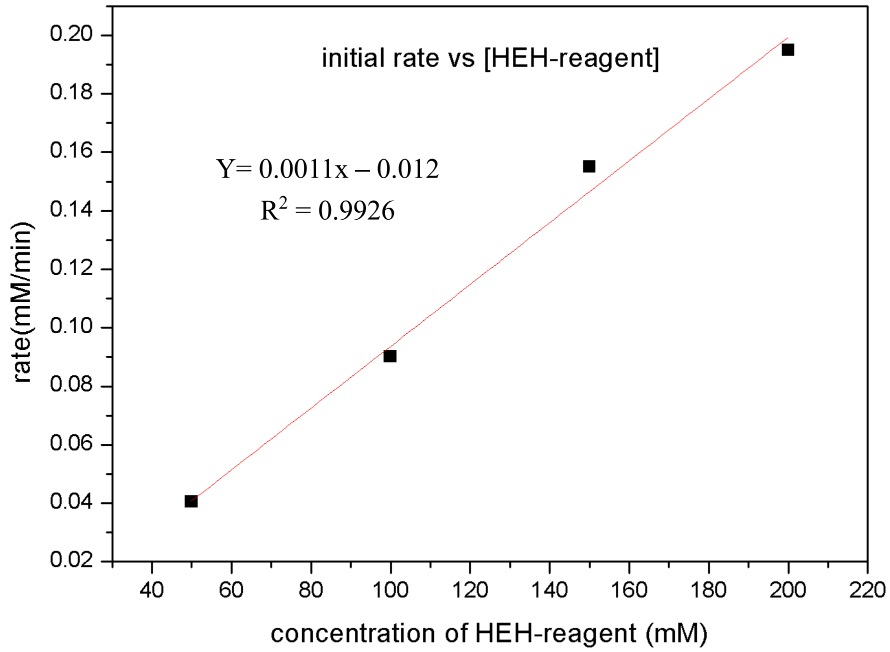

Fig. S29. Initial rate regarding the HEH-reagent.

Reaction rate order in TBSOTf (Fig.s S30-S34): zero order

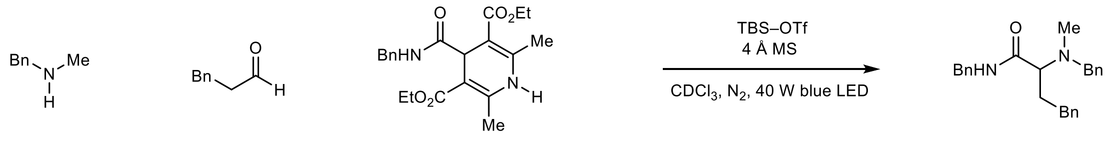

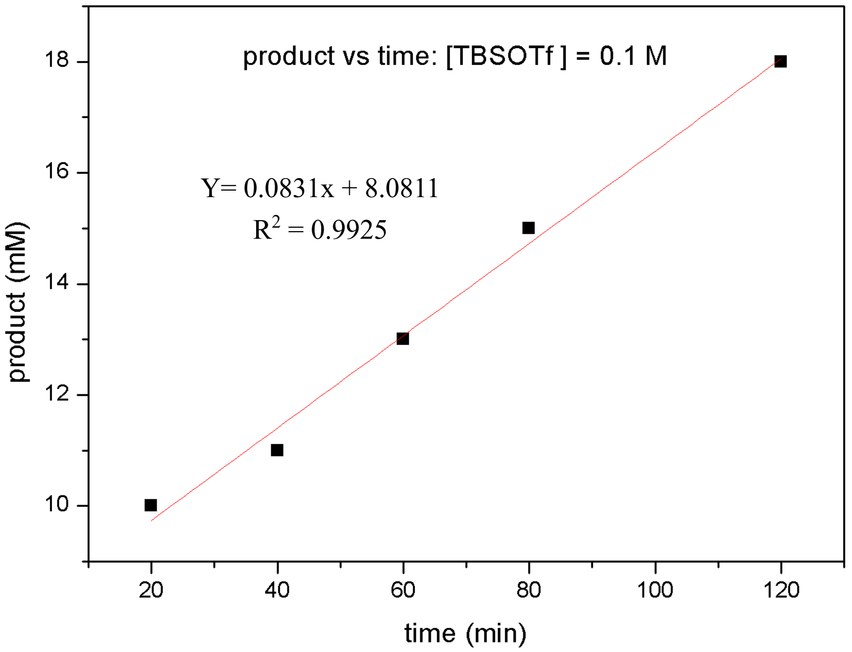

Fig. S30. Rate of product formation as a function of time when [TBSOTf] = 0.10 M.

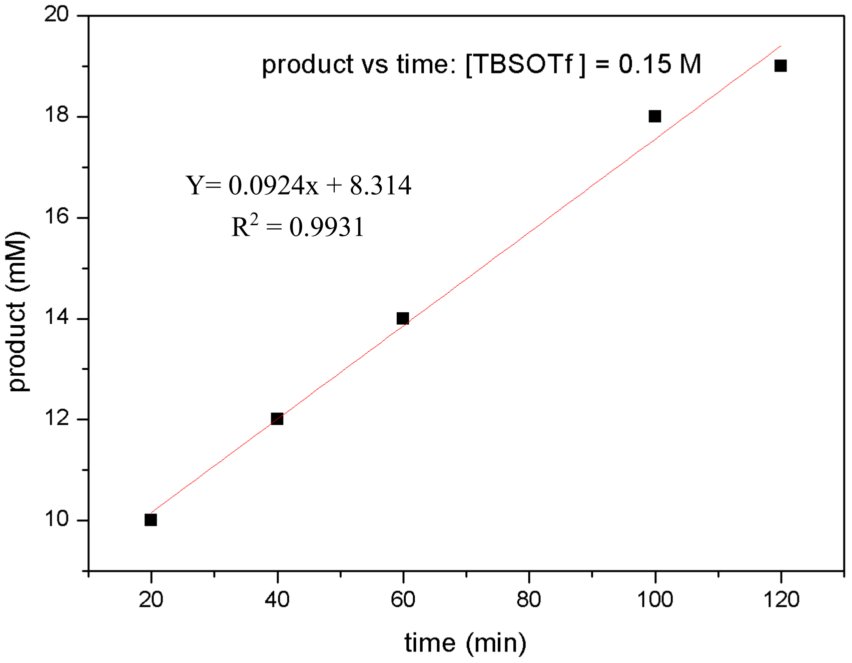

Fig. S31. Rate of product formation as a function of time when [TBSOTf] = 0.15 M.

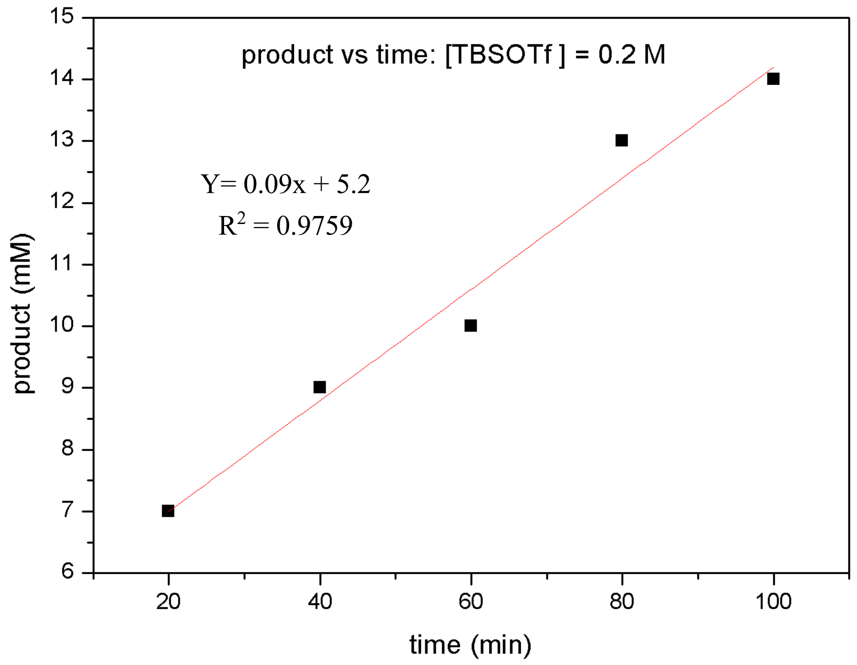

Fig. S32. Rate of product formation as a function of time when [TBSOTf] = 0.20 M.

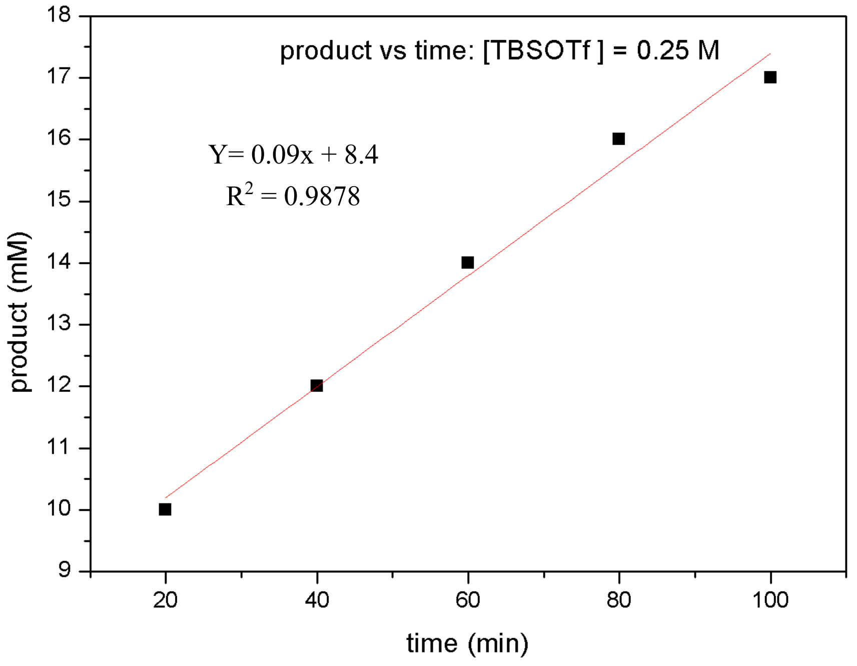

Fig. S33. Rate of product formation as a function of time when [TBSOTf] = 0.25 M.

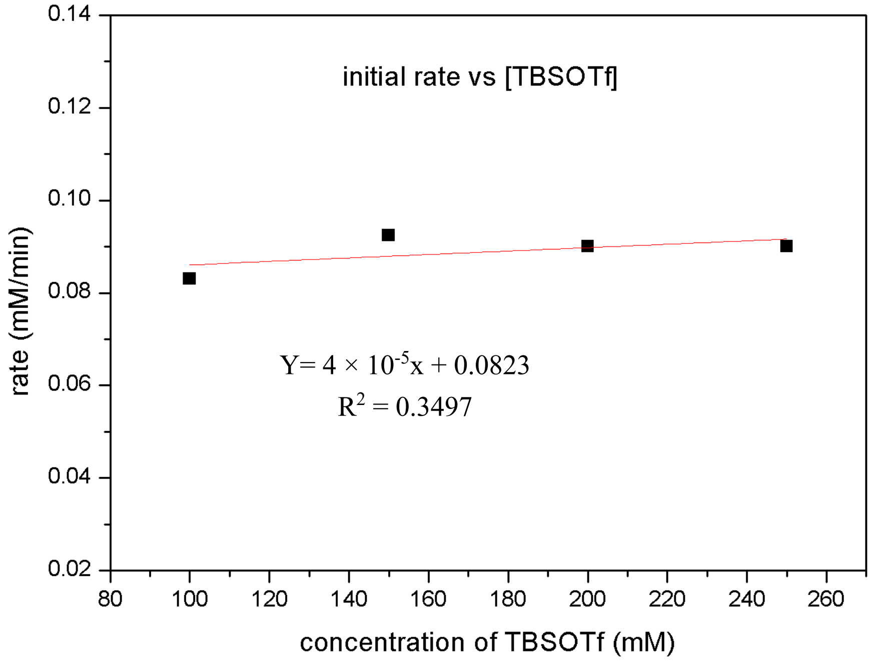

Fig. S34. Initial rate regarding the TBSOTf

The kinetic comparison between TBS-OTf and TBS-OTf/Sc(OTf)$_3$ activation of Carbonyl Acylative Amination (CAcylA)

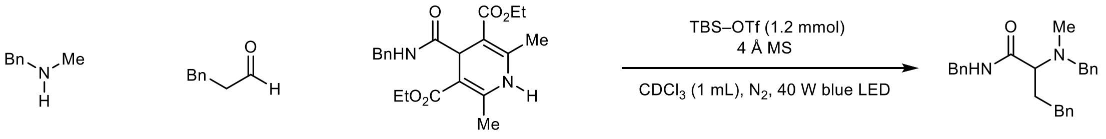

0.1 mmol **0.12 mmol** **0.12 mmol** **4f**

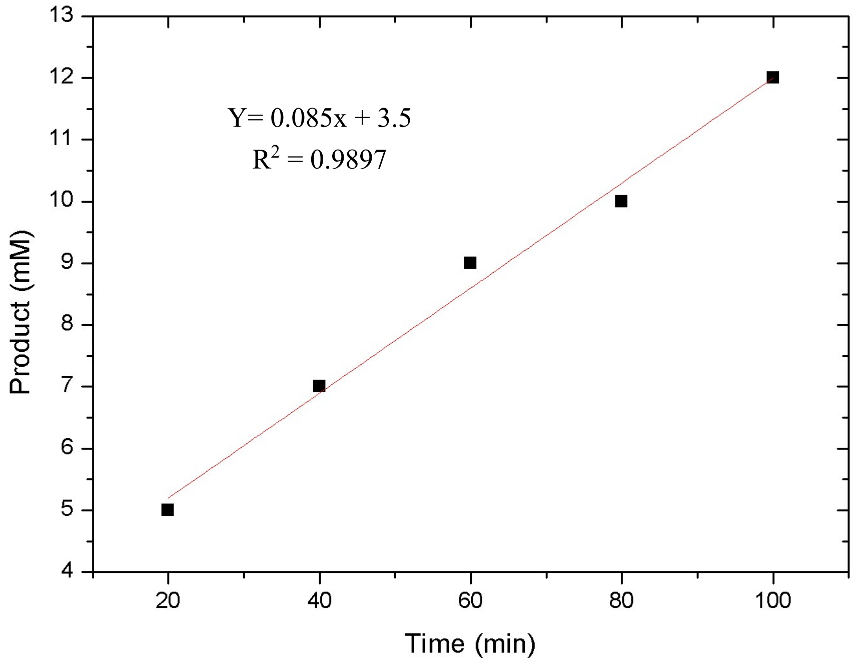

Fig. S35. Initial rate under conditions without Sc: 1.4× 10^{-6}mol/L/S^{-1}

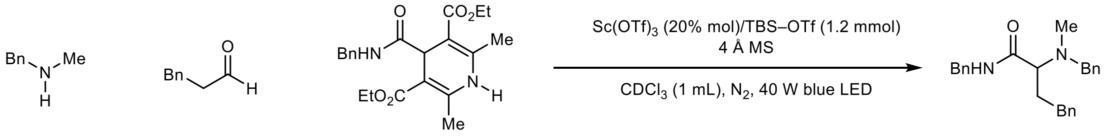

0.1 mmol **0.12 mmol** **0.12 mmol** **8f**

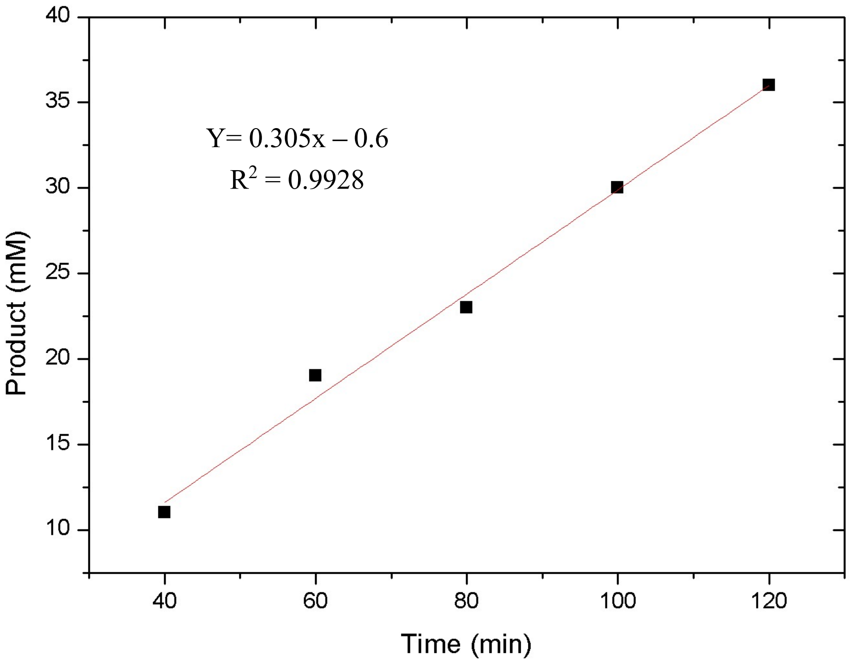

Fig. S36. Initial rate regarding the Sc and TBSOTf: 5.1× 10^{-6}mol/L/S^{-1}

The result clearly showed that reaction rate under TBS-OTf and $Sc(OTf)_3$ combination (5.1 × 10^{-6} mol/L/S^{-1}) is almost four-folds of magnititude than the soly TBS-OTf conditions (1.4× 10^{-6}mol/L/S^{-1}) (Figs. S35, S36).

Parallel two-dimensional array for the synthesis of a library of alfa-amino carbonyls

<u>Plate set-up and initial reactivity evaluation by ^{19}F-NMR:</u>

The initial array plate spaning 64 pharma-related fragments was designed to evaluate the carbonyl acylative amination (CAcylA) reactivity wherein each of the amine, carbonyl and carboxamide radical components could be systematically varied. Each of the 4-carboxamoyl dihydropyridines contained a fluorine atom so that a quantitative assay yield of products could be calculated through ^{19}F NMR analysis. All the reactions were conducted as general procedure A in 50 umol scale using the 4 mL micro-vial equipped with septa-lined screw cap under the excitation of lumidox LED Array (Lumidox® II, 445 nm 96-well LED Array, 12 W of total power, 125 mw per well), except that the aldehyde (2.0 equiv), TBSOTf (2.4 equiv) and $BF_3.Et_2O$ (3.0 equiv) was used instead. After reactions, the mixture was filtered through amberlyst to neutralize the acid for directly ^{19}F NMR experiments. The mass of the products was detected by LCMS. All the yield was calculated through ^{19}F NMR analysis using trifluorotoluene as standard internals. (Figs. S37, S38, S39).

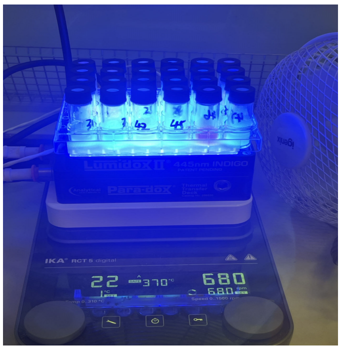

Fig. S37. Array plate set-up.

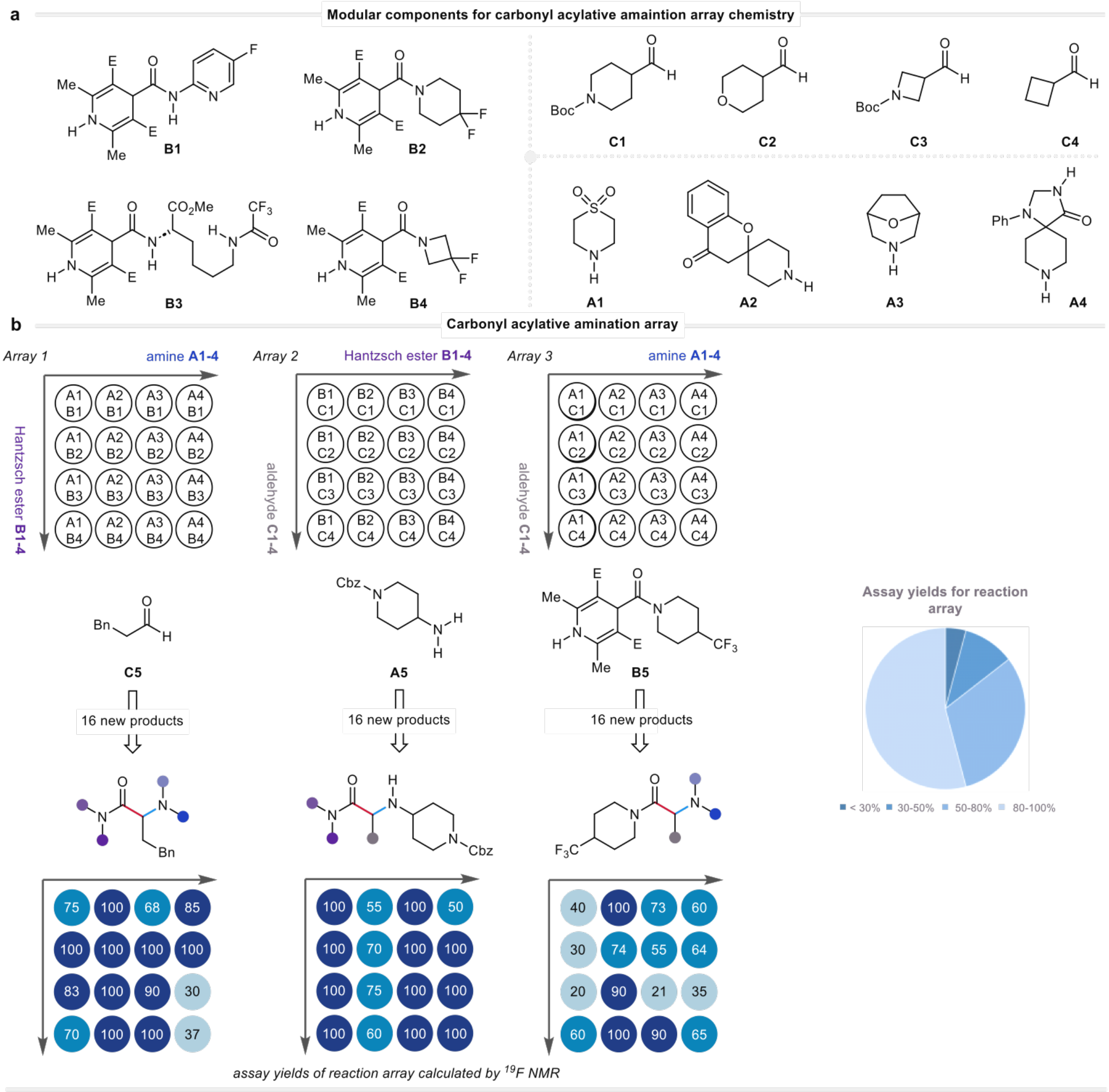

Fig. S38. Array design and result read-out.

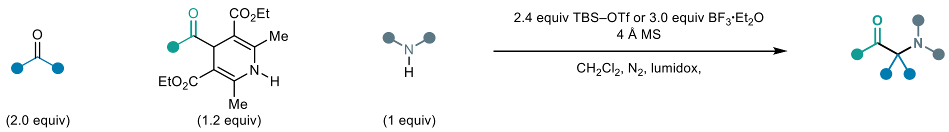

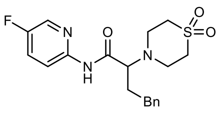

array 1, AY 75%

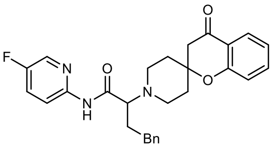

array 2. AY quant.

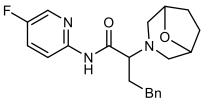

array 3, AY 68%

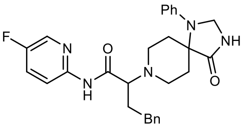

array 4, AY 85%

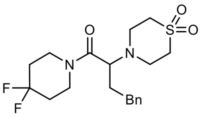

array 5, AY quant.

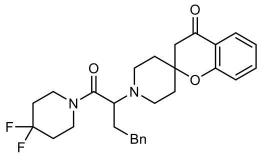

array 6, AY quant.

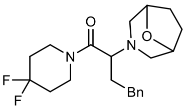

array 7, AY quant.

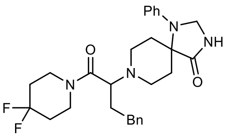

array 8, AY quant.

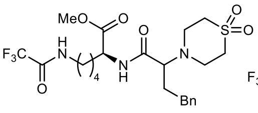

array 9, AY 93%

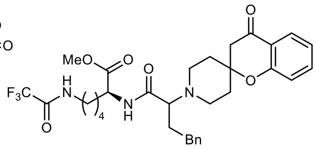

array 10, AY quant.

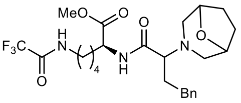

array 11, AY 90%

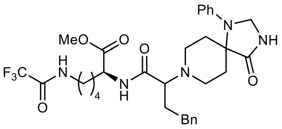

array 12, AY 30%

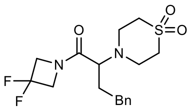

array 13, AY 70%

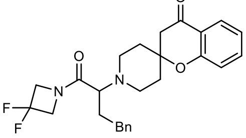

array 14, AY quant.

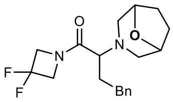

array 15, AY quant.

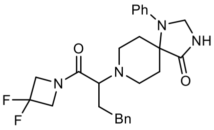

array 16, AY 37%

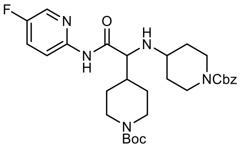

array 17, AY quant.

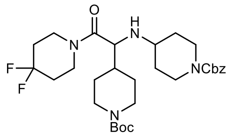

array 18. AY 55%

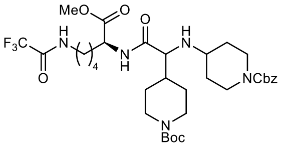

array 19, AY quant.

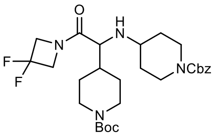

array 20, AY 50%

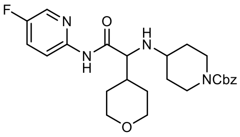

array 21, AY quant.

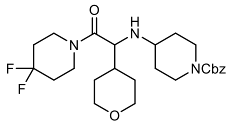

array 22, AY 70%

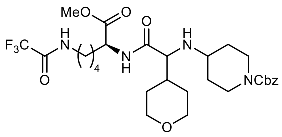

array 23,AY quant.

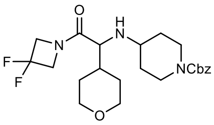

array 24, AY quant.

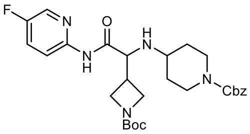

array 25, AY quant.

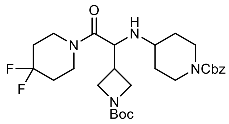

array 26, 75%

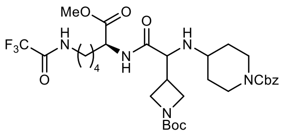

array 27, AY quant.

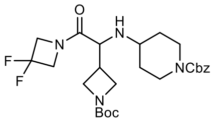

array 28, AY quant.

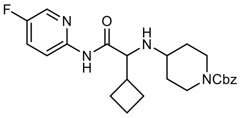

array 29, AY quant.

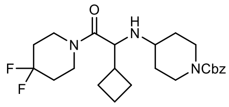

array 30, AY 60%

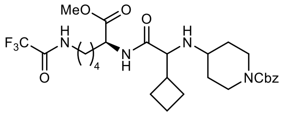

array 31, AY quant.

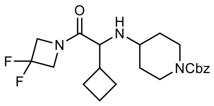

array 32, AY quant.

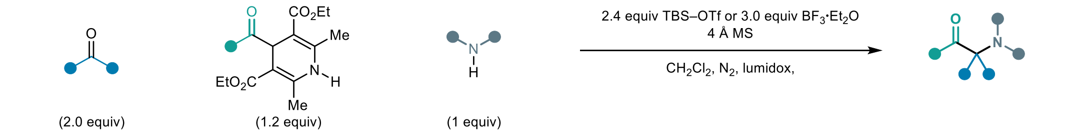

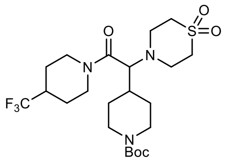

array 33, AY 40%

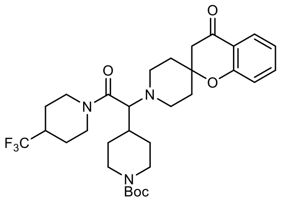

array 34. AYquant.

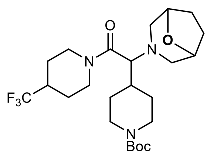

array 35, AY 73%

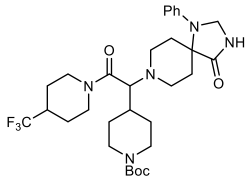

array 36, AY 60%

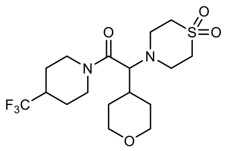

array 37, AY 30%.

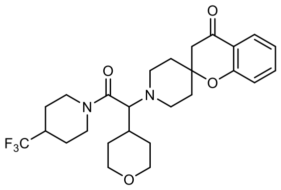

array 38, AY 74%

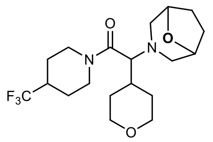

array 39,AY 55%

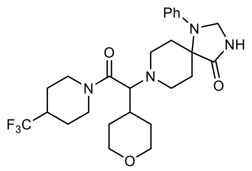

array 40, AY 64%

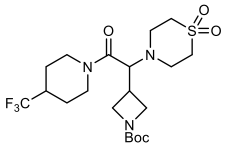

array 41, AY 40%

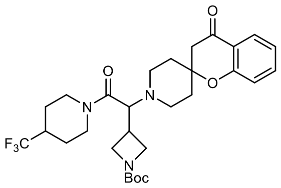

array 42, 90%

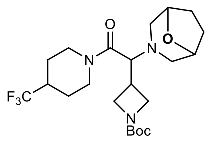

array 43, AY 21%

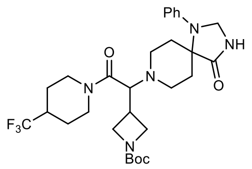

array 44, AY 35%

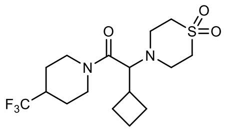

array 45, AY 60%

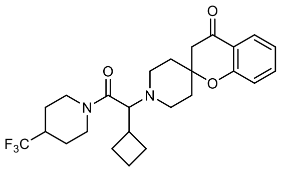

array 46, AY quant.

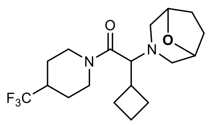

array 47, AY 90%

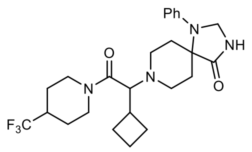

array 48, AY 65%

Fig. S39. 48-Array compound library

<u>Reaction set-up and work-up for parallel-array applications</u>

Encouraged by the above results, we reasoned whether this chemistry can be transferred to array process to provide purified products to construct a library of alpha-amino carbonyls. After extensive exploration, the recovered aldehyde and amine were removed by commercially available amberlyst resins scavengers, and the starting material of hantzsch ester reagent could be fully consumed by adding the Sc(OTf)3 in the reaction and the amount of aldehyde could be decreased to 1.5 equivalent under this modified conditions. After reaction, 60 mg aldehyde scavenger and 50 mg amine scavenger were added respectively and continued stirring for 4 hs. Then MeOH (1.5 mL) and aq. NaOH (2 M, 400 uL) were added (hydrolysis and removal of pyridine sideproduct from DHP reagent), and the mixture was vigorously stirred at room temperature overninght (Fig. S40).

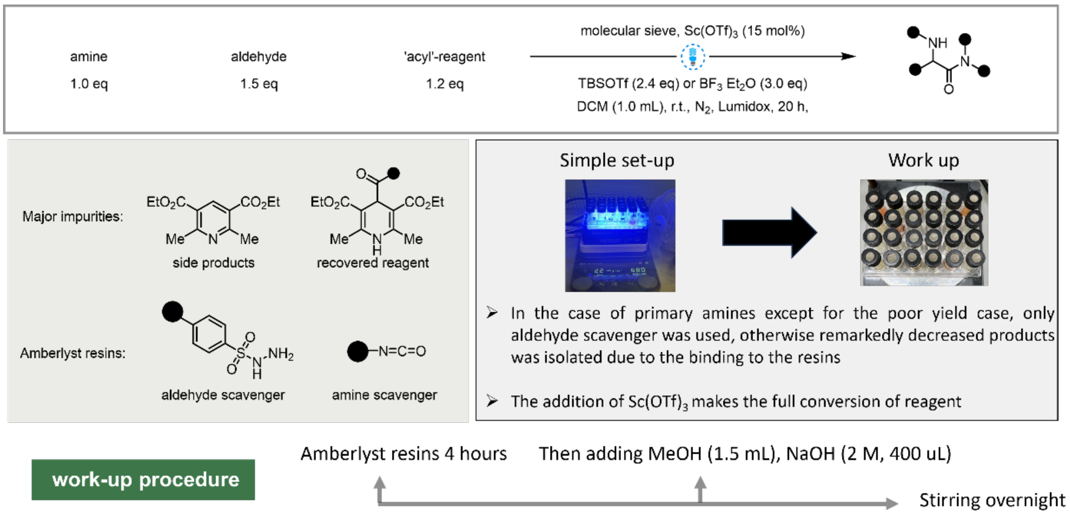

Fig. S40. Work-up of array plate reaction.

General workflow for array applications

After the work-up procedure, the puirification process was explored to provide purfied product for further biological binding study (Fig. S41).

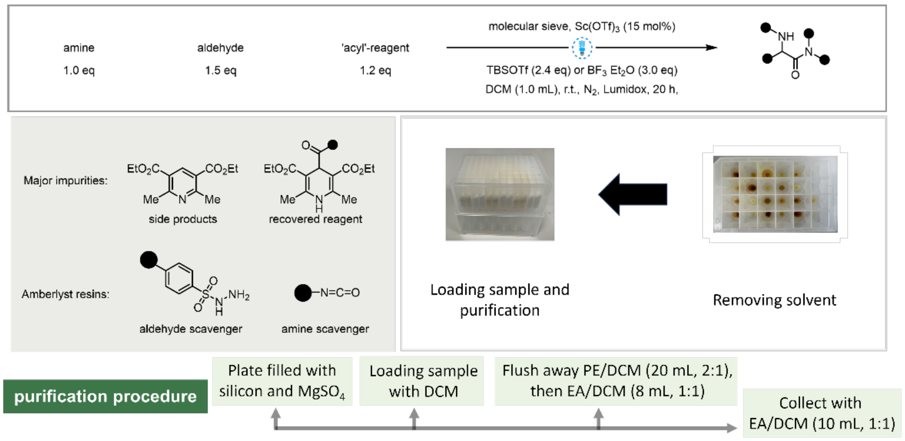

Fig. S41. Purification of array plate reaction.

After the identification of standard procedure, one round of experiments was performed using a 24-well plate containing 24 compounds, which encompassed various combinations involving the dispersion of all the amines, aldehydes and reagents (Fig. S42, S43).

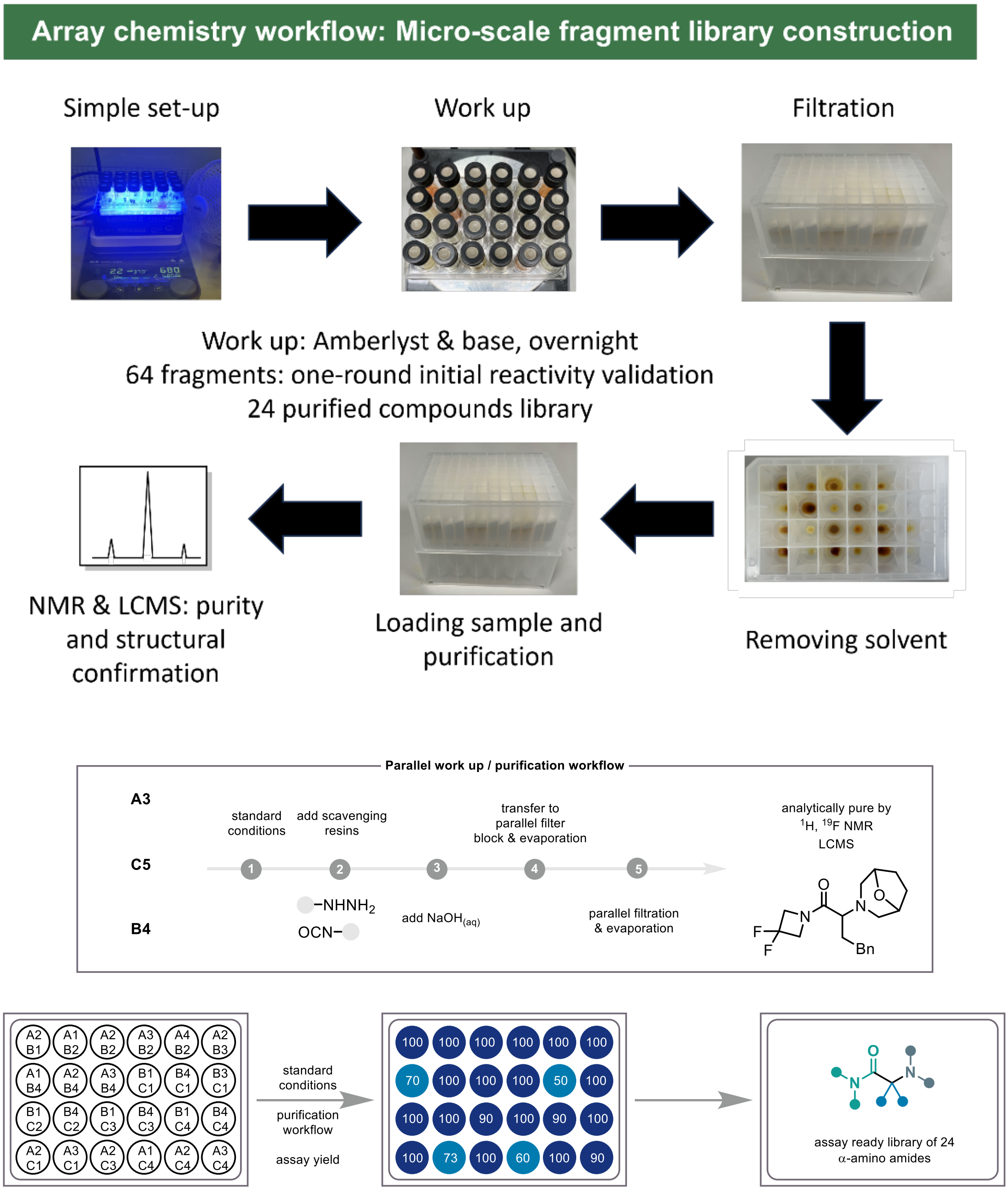

Fig. S42. General workflow for the array library compound construction.

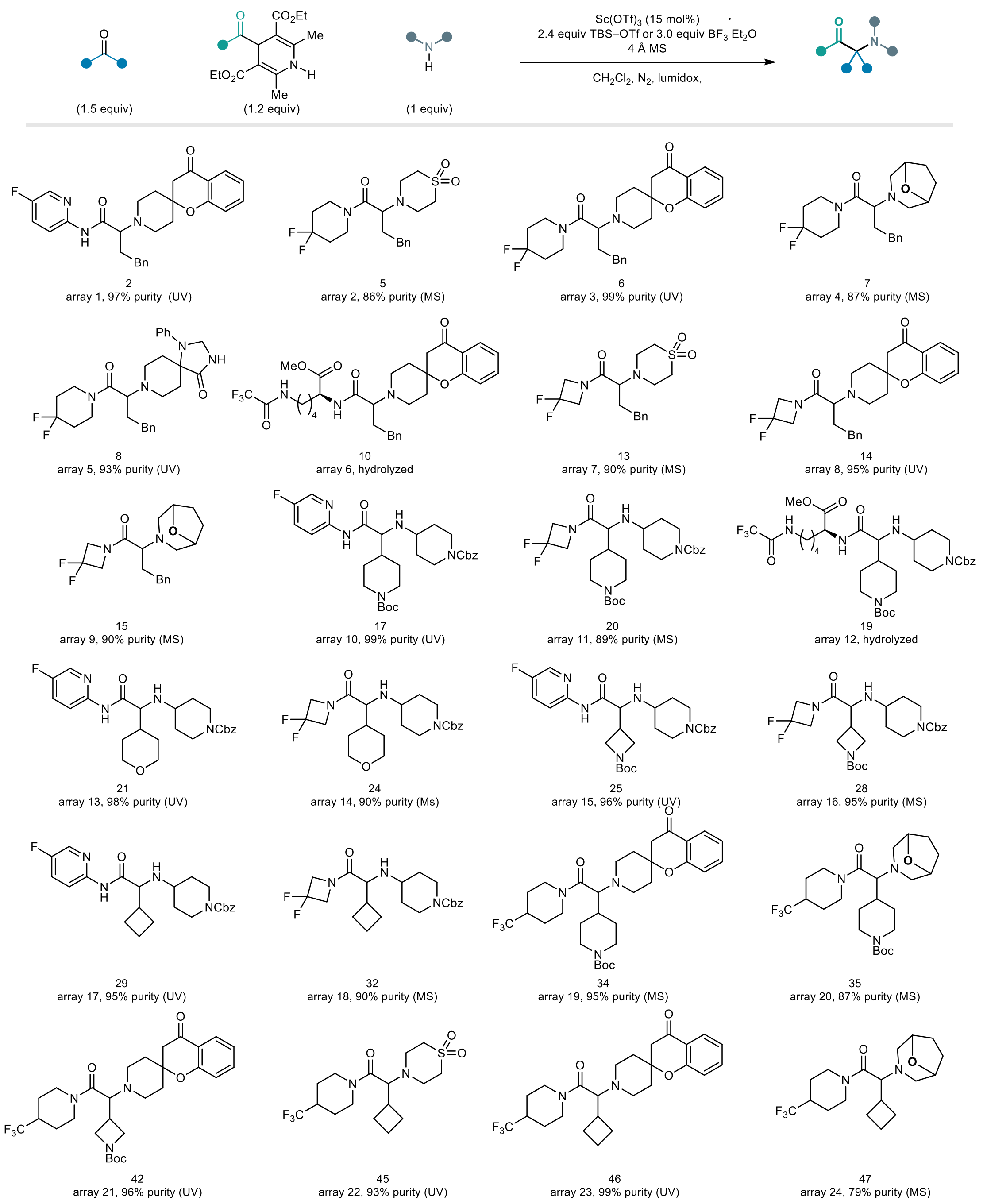

- The number in the first line assigned to the compound indicates its position in the queue relative to the initial 48 array numbers.
- NMR and LCMC to validate the structure and purity confirmations.
- Amino acid ester reagent case (Array 6 and 12, high yield products, purification failed due to the hydrolysation of the products)
- 22 purified compounds library
- using LCMS, purity was identified by liquid chromatography-UV detection (254 nm) or liquid chromatography-mass spectrometry (Compounds poorly absorbed under UV conditions)

Fig. S43. Represeantative 24 purified array library compounds.

Extended supplementary substrates

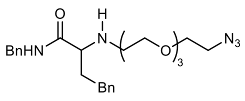

S1 IY 50%

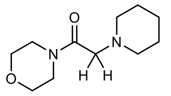

S2 AY 78%, IY 45%

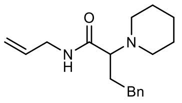

S3 AY 85%, IY 73%

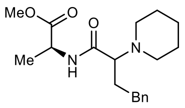

S4 AY 88%, IY 81% (1.2:1 dr)

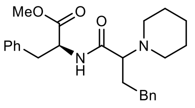

S5 IY 51% (1.2:1 dr)

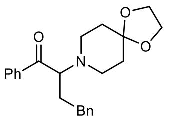

S6 AY 90%, IY 66%

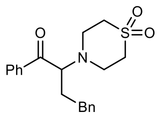

S7 AY 60%, IY 56%

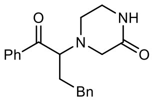

S8 AY 40%, IY 36%

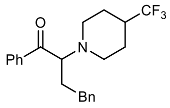

S9 AY 70%, IY 61%

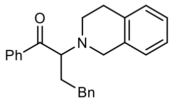

S10 AY 45%, IY 32%

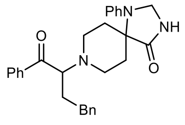

S11 AY 80%, IY 66%

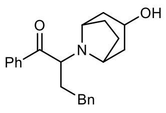

S12 IY 43%

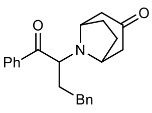

S13 AY 66%, IY 46%

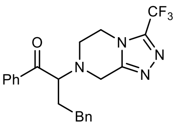

S14 AY 60%, IY 51%

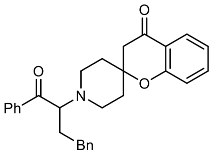

S15 AY 65%, IY 60%

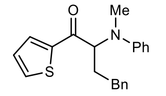

S16 AY 54%, IY 45%

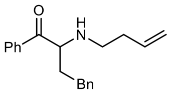

S17 AY 51%, IY 40%

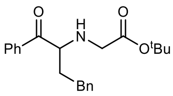

S18 AY 50%, IY 37%

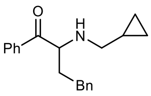

S19 AY 80%, IY 60%

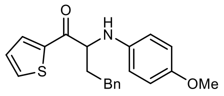

S20 AY 50%, IY 40%

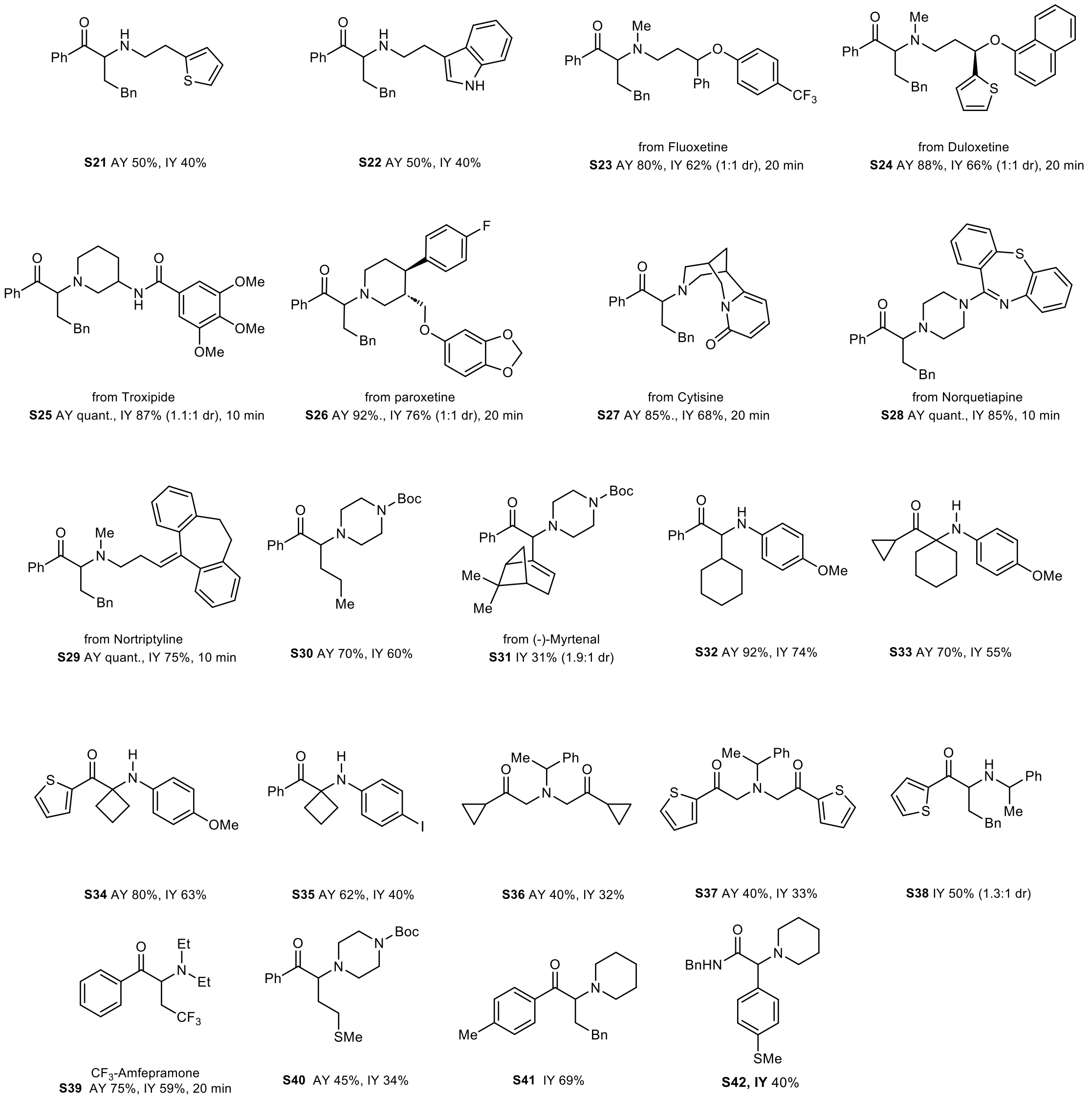

Fig. S44. Supplementary substrate scope for the carbonyl acylative amination

References

50 Fleming, I. *Molecular orbitals and organic chemical reactions*. (John Wiley & Sons, 2011).

51 Gilbert, A. & Baggott, J. E. Essentials of molecular photochemistry. *(No Title)* (1991).

52 Turro, N. J., Ramamurthy, V. & Scaiano, J. C. *Modern molecular photochemistry of organic molecules*. Vol. 188 (University Science Books Sausalito, CA, 2010).

53 Goti, G., Bieszczad, B., Vega-Peñaloza, A. & Melchiorre, P. Stereocontrolled Synthesis of 1,4-Dicarbonyl Compounds by Photochemical Organocatalytic Acyl Radical Addition to Enals. *Angew. Chem. Int. Ed.* **58**, 1213-1217 (2019).

54 Luo, X. & Wang, P. Ynonylation of Acyl Radicals by Electroinduced Homolysis of 4-Acyl-1,4-dihydropyridines. *Org. Lett.* **23**, 4960-4965 (2021).

55 Dubur, G. Y. & Uldrikis, Y. R. Preparation of 3,5-diethoxycarbonyl-2,6-dimethyl-1,4-dihydro-isonicotinic acid and 3,5-diacetyl-2,6-dimethyl-1,4-dihydroisoni-cotinic acid and their salts. *Chemistry of Heterocyclic Compounds* **5**, 762-763 (1972).

56 Alandini, N. *et al.* Amide Synthesis by Nickel/Photoredox - Catalyzed Direct

Carbamoylation of (Hetero) Aryl Bromides. *Angew. Chem.* **132**, 5286-5291 (2020).
57 Mindt, T. L. *et al.* Molecular Assembly of Multifunctional 99mTc Radiopharmaceuticals Using "Clickable" Amino Acid Derivatives. *ChemMedChem* **5**, 2026-2038 (2010).

Carbonyl Acylative Amination products

_N_-benzyl-4-phenyl-2-(piperidin-1-yl)butanamide (4a)

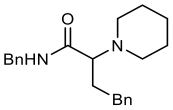

According to general procedure A, the reaction of piperidine (10 uL, 0.1 mmol), hydrocinnamaldehyde (16 uL, 0.12 mmol), diethyl 4-(benzylcarbamoyl)-2,6-dimethyl-1,4-dihydropyridine-3,5-dicarboxylate (46 mg, 0.12 mmol), 4 Å molecular sieve (200 mg), TBSOTf (28 uL, 0.12 mmol) in DCM (2 mL). The crude reaction was purified by flash column chromatography (PE/Acetone = 5:1) to provide product as yellow oil (27 mg, 80%).

If using improved Sc(OTf)$_3$ (20 mol%, 10 mg) and TBSOTf (28 uL, 0.12 mmol) as the activation conditions, 30 mg of product was provided (90% yield).

^{1}H NMR (400 MHz, CDCl$_3$): δ 7.53 (t, *J* = 6.1 Hz, 1H), 7.40 – 7.27 (m, 7H), 7.24-7.19 (m, 3H), 4.55 (dd, *J* = 14.8, 6.2 Hz, 1H), 4.46 (dd, *J* = 14.8, 5.8 Hz, 1H), 2.99 (dd, *J* = 8.1, 4.5 Hz, 1H), 2.93-2.85 (m, 1H), 2.73-2.66 (m, 1H), 2.59-2.53 (m, 2H), 2.46-2.40 (m, 2H), 2.23 – 1.90 (m, 2H), 1.61 – 1.38 (m, 6H); **^{13}C NMR (101 MHz, CDCl$_3$)**: δ 173.31, 142.05, 138.73, 128.64, 128.43, 128.31, 127.58, 127.32, 125.81, 68.58, 51.16, 43.08, 33.55, 28.70, 26.39, 24.21; HRMS *m/z* (ESI) calcd for $C_{22}H_{29}N_2O$ (M + H)$^+$, 337.2280, found 337.2279. IR Qmax/cm-1 (film): 698, 749, 1453, 1495, 151, 1648, 2806, 2850, 2931, 3026, 3304.

_N_-benzyl-2-morpholino-4-phenylbutanamide (4b)

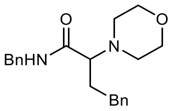

According to general procedure A, the reaction of morpholine (8.7 mg, 0.1 mmol), hydrocinnamaldehyde (16 uL, 0.12 mmol), diethyl 4-(benzylcarbamoyl)-2,6-dimethyl-1,4-dihydropyridine-3,5-dicarboxylate (46 mg, 0.12 mmol), 4 Å molecular sieve (200 mg), TBSOTf (28 uL, 0.12 mmol) in DCM (2 mL). The crude reaction was purified by flash column chromatography (PE/Acetone = 3:1) to provide product as yellow oil (21 mg, 62%). **^{1}H NMR (400 MHz, CDCl$_3$)**: δ 7.41 – 7.27 (m, 7H), 7.23-7.19 (m, 4H), 4.51 (qd, *J* = 14.7, 6.0 Hz, 2H), 3.78 – 3.60 (m, 4H), 3.02 – 2.93 (m, 1H), 2.87 – 2.76 (m, 1H), 2.73-2.65 (m, 1H), 2.62-2.57 (m, 2H), 2.53-2.47 (m, 2H), 2.09 – 1.97 (m, 2H); **^{13}C NMR (101 MHz, CDCl$_3$)**: δ 172.32, 141.59, 138.49, 128.73, 128.40, 128.38, 127.68, 127.51, 125.97, 68.67, 67.10, 50.56, 43.16, 32.82, 29.37; HRMS *m/z* (ESI) calcd for $C_{21}H_{27}N_2O_2$ (M + H)$^+$, 339.2073, found 339.2071. IR Qmax/cm-1 (film): 698, 730, 880, 1046, 1081, 1116, 1453, 1496, 1517, 1648, 2853, 2924, 2968, 3304.

_N_-benzyl-4-phenyl-2-thiomorpholinobutanamide (4c)

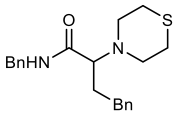

According to general procedure A, the reaction of thiomorpholine (10.3 mg, 0.1 mmol), hydrocinnamaldehyde (16 uL, 0.12 mmol), diethyl 4-(benzylcarbamoyl)-2,6-dimethyl-1,4-dihydropyridine-3,5-dicarboxylate (46 mg, 0.12 mmol), 4 Å molecular sieve (200 mg), TBSOTf (28 uL, 0.12 mmol) in DCM (2 mL). The crude reaction was purified by flash column chromatography (PE/Acetone = 5:1) to provide product as yellow oil (29 mg, 82%). **^{1}H NMR (400 MHz, CDCl$_3$)**: δ 7.42 – 7.30 (m, 7H), 7.27 – 7.17 (m, 4H), 4.55 (dd, *J* = 14.7, 6.3 Hz, 1H), 4.42 (dd, *J* = 14.7, 5.5 Hz, 1H), 3.02 (dd, *J* = 8.4, 4.4 Hz, 1H), 2.97 – 2.81 (m, 3H), 2.79 – 2.67 (m, 3H), 2.64-2.53 (m, 4H), 2.19-2.10 (m, 1H), 1.99-1.90 (m, 1H); **^{13}C NMR (101 MHz, CDCl$_3$)**: δ 172.34, 141.68, 138.53, 128.72, 128.45, 128.36, 127.62, 127.48, 125.92, 68.68, 51.99, 43.22, 33.73, 28.37, 27.68; HRMS *m/z* (ESI) calcd for $C_{21}H_{27}N_2OS$ $(M + H)^+$, 355.1844, found 355.1840. IR Qmax/cm-1 (film): 698, 750, 1453, 1495, 1513, 1647, 2918, 3304.

_N_-benzyl-2-(4-bromopiperidin-1-yl)-4-phenylbutanamide (4d)

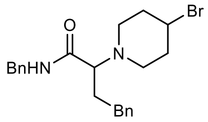

According to general procedure C, the reaction of 4-bromopiperidine hydrobromide salt (24.5 mg, 0.1 mmol), hydrocinnamaldehyde (16 uL, 0.12 mmol), Et$_3$N (14 uL, 0.1 mmol), diethyl 4-(benzylcarbamoyl)-2,6-dimethyl-1,4-dihydropyridine-3,5-dicarboxylate (46 mg, 0.12 mmol), 4 Å molecular sieve (200 mg), TBSOTf (28 uL, 0.12 mmol) in DCM (2 mL). The crude reaction was purified by flash column chromatography (PE/Acetone = 3:1) to provide product as yellow oil (27 mg, 65%). **^{1}H NMR (400 MHz, CDCl$_3$)**: δ 7.41 – 7.34 (m, 2H), 7.34 – 7.28 (m, 5H), 7.23-7.19 (m, 4H), 4.58 – 4.42 (m, 2H), 4.17 (tt, *J* = 8.7, 4.0 Hz, 1H), 3.02 (dd, *J* = 8.3, 4.6 Hz, 1H), 2.93 – 2.79 (m, 2H), 2.77-2.65 (m, 2H), 2.51-2.44 (m, 1H), 2.39-2.32 (m, 1H), 2.20-1.92 (m, 6H); **^{13}C NMR (101 MHz, CDCl$_3$)**: δ 172.48, 141.67, 138.54, 128.71, 128.41, 128.37, 127.62, 127.47, 125.93, 67.92, 49.15, 48.43, 43.15, 36.57, 33.29, 28.85; HRMS *m/z* (ESI) calcd for $C_{22}H_{28}BrN_2O$ $(M + H)^+$, 415.1380, found 415.1361. IR Qmax/cm-1 (film): 698, 732, 1195, 1253, 1453, 1495, 1514, 1646, 2925, 2949, 3294.

2-(Azetidin-1-yl)-N-benzyl-4-phenylbutanamide (4e)

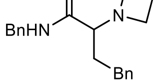

According to general procedure A, the reaction of azetidine (5.7 mg, 0.1 mmol hydrocinnamaldehyde (16 uL, 0.12 mmol), diethyl 4-(benzylcarbamoyl)-2,6-dimethyl-1,4-dihydropyridine-3,5-dicarboxylate (46 mg, 0.12 mmol), 4 Å molecular sieve (200 mg), TBSOTf (28 uL, 0.12 mmol) in DCM (2 mL). The crude reaction was purified by flash column chromatography (PE/Acetone = 3:1) to provide product as yellow oil (25

mg, 81%). **^{1}H NMR (400 MHz, $CDCl_3$)**: δ 7.40 – 7.23 (m, 8H), 7.23 – 7.15 (m, 3H), 4.49 (qd, *J* = 14.7, 5.9 Hz, 2H), 3.26 (dq, *J* = 20.3, 7.0 Hz, 4H), 3.03 – 2.86 (m, 1H), 2.76 – 2.48 (m, 2H), 2.13 – 1.88 (m, 3H), 1.80-1.70 (m, 1H); **^{13}C NMR (101 MHz, $CDCl_3$)**: δ 171.86, 141.74, 138.50, 128.66, 128.37, 128.28, 127.81, 127.41, 125.90, 71.80, 53.46, 43.01, 32.26, 31.08, 17.08; HRMS *m/z* (ESI) calcd for $C_{20}H_{25}N_2O$ (M + H)$^+$, 309.1967, found 309.1965. IR Qmax/cm-1 (film): 698, 748, 1238, 1453, 1496, 1516, 1659, 2925, 2954, 3305.

***N*-benzyl-2-(benzyl(methyl)amino)-4-phenylbutanamide (4f)**

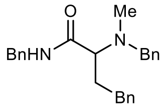

According to general procedure A, the reaction of N-methyl-1-phenylmethanamine (12.2 mg, 0.1 mmol), hydrocinnamaldehyde (16 uL, 0.12 mmol), diethyl 4-(benzylcarbamoyl)-2,6-dimethyl-1,4-dihydropyridine-3,5-dicarboxylate (46 mg, 0.12 mmol), 4 Å molecular sieve (200 mg), TBSOTf (28 uL, 0.12 mmol) in DCM (2 mL). The crude reaction was purified by flash column chromatography (PE/Acetone = 3:1) to provide product as yellow oil (22 mg, 60%). **^{1}H NMR (400 MHz, $CDCl_3$)**: δ 7.41 – 7.20 (m, 14H), 7.16 – 7.10 (m, 2H), 4.51 (qd, *J* = 14.8, 5.9 Hz, 2H), 3.68 – 3.50 (m, 2H), 3.16 (dd, *J* = 8.5, 4.2 Hz, 1H), 3.07 – 2.92 (m, 1H), 2.77-2.69 (m, 1H), 2.24 (s, 3H), 2.22-2.14 (m, 1H), 2.05-1.96 (m, 1H); **^{13}C NMR (101 MHz, $CDCl_3$)**: δ 173.11, 142.01, 138.55, 138.53, 128.67, 128.54, 128.40, 127.71, 127.40, 127.19, 125.89, 65.65, 59.06, 43.20, 38.16, 33.81, 28.02; HRMS *m/z* (ESI) calcd for $C_{25}H_{29}N_2O$ (M + H)$^+$, 373.2280, found 373.2277. IR Qmax/cm-1 (film): 697, 734, 1028, 1453, 1495, 1509, 1648, 2929, 3026, 3061, 3305.

Benzyl 4-((1-oxo-4-phenyl-1-(phenylamino)butan-2-yl)amino)piperidine-1-carboxylate (4g)

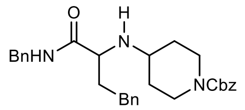

According to general procedure A, the reaction of benzyl 4-aminopiperidine-1-carboxylate (23.4 mg, 0.1 mmol), hydrocinnamaldehyde (16 uL, 0.12 mmol), diethyl 4-(benzylcarbamoyl)-2,6-dimethyl-1,4-dihydropyridine-3,5-dicarboxylate (46 mg, 0.12 mmol), 4 Å molecular sieve (200 mg), $BF_3.Et_2O$ (19 uL, 0.15 mmol) in DCM (2 mL). The crude reaction was purified by flash column chromatography (PE/Acetone = 3:1) to provide product as yellow oil (38 mg, 81%). **^{1}H NMR (400 MHz, $CDCl_3$)**: δ 7.52 (t, *J* = 6.1 Hz, 1H), 7.41 – 7.27 (m, 12H), 7.22 (t, *J* = 8.2 Hz, 3H), 5.13 (s, 2H), 4.47 (d, *J* = 5.0 Hz, 2H), 4.10 (d, *J* = 18.0 Hz, 2H), 3.26 (dd, *J* = 7.9, 4.6 Hz, 1H), 2.76 (q, *J* = 7.9 Hz, 4H), 2.48 (tt, *J* = 10.5, 3.9 Hz, 1H), 2.25 – 2.09 (m, 1H), 1.88 (dq, *J* = 15.3, 7.8 Hz, 1H), 1.80 – 1.60 (m, 2H), 1.51 – 1.35 (m, 1H), 1.24 – 1.07 (m, 2H); **^{13}C NMR (101 MHz, $CDCl_3$)**: δ 174.53, 155.09, 140.91, 138.44, 136.71, 128.65, 128.51, 128.44, 128.32, 127.97, 127.83, 127.60, 127.44, 126.17, 67.05, 60.42, 54.57, 43.01, 42.69, 35.62, 33.01, 32.55, 32.29; HRMS *m/z* (ESI) calcd for $C_{29}H_{33}N_3O_3Na$ (M + Na)$^+$, 494.2414 , found 494.2425.

IR Qmax/cm-1 (film): 697, 733, 1136, 1227, 1274, 1431, 1452, 1469, 1496, 1518, 1693, 2856, 2926, 3314.

***N*-benzyl-2-cyclohexyl-2-thiomorpholinoacetamide (4h)**

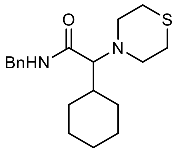

According to general procedure A, the reaction of thiomorpholine (10.3 mg, 0.1 mmol), cyclohexanecarbaldehyde (13 uL, 0.12 mmol), diethyl 4-(benzylcarbamoyl)-2,6-dimethyl-1,4-dihydropyridine-3,5-dicarboxylate (46 mg, 0.12 mmol), 4 Å molecular sieve (200 mg), TBSOTf (28 uL, 0.12 mmol) in DCM (2 mL). The crude reaction was purified by flash column chromatography (PE/Acetone = 5:1) to provide product as yellow oil (22.9 mg, 69%). **^{1}H NMR (400 MHz, CDCl$_3$)**: δ 7.40 – 7.33 (m, 2H), 7.33 – 7.26 (m, 3H), 6.35 (t, *J* = 5.8 Hz, 1H), 4.54 – 4.38 (m, 2H), 2.91 – 2.75 (m, 4H), 2.72 – 2.51 (m, 5H), 2.00 – 1.58 (m, 6H), 1.33 – 0.86 (m, 5H); **^{13}C NMR (101 MHz, CDCl$_3$)**: δ 170.69, 138.38, 128.70, 127.91, 127.50, 75.80, 52.67, 43.15, 36.16, 30.38, 29.49, 28.36, 26.60, 26.34, 26.07; HRMS *m/z* (ESI) calcd for $C_{19}H_{29}N_2OS$ $(M + H)^+$, 333.2001, found 333.2000. IR Qmax/cm-1 (film): 697, 731, 1223, 1280, 1452, 1550, 1638, 2848, 2922, 3279.

Tert-butyl 4-(2-(benzylamino)-2-oxo-1-thiomorpholinoethyl)piperidine-1-carboxylate (4i)

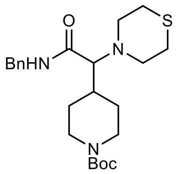

According to general procedure A, the reaction of thiomorpholine (10.3 mg, 0.1 mmol), tert-butyl 4-formylpiperidine-1-carboxylate (26 mg, 0.12 mmol), diethyl 4-(benzylcarbamoyl)-2,6-dimethyl-1,4-dihydropyridine-3,5-dicarboxylate (46 mg, 0.12 mmol), 4 Å molecular sieve (200 mg), TBSOTf (28 uL, 0.12 mmol) in DCM (2 mL). The crude reaction was purified by flash column chromatography (PE/Acetone = 3:1) to provide product as yellow oil (37 mg, 85%). **^{1}H NMR (400 MHz, CDCl$_3$)**: δ 7.38 – 7.25 (m, 5H), 6.27 (s, 1H), 4.54-4.39 (m, 2H), 4.22 – 4.02 (m, 2H), 2.97 – 2.90 (m, 2H), 2.82-2.76 (m, 2H), 2.73 – 2.53 (m, 8H), 2.10 – 1.98 (m, 2H), 1.83 (d, *J* = 13.5 Hz, 1H), 1.58-1.53 (m, 1H), 1.45 (s, 9H); **^{13}C NMR (101 MHz, CDCl$_3$)**: δ 169.67, 154.69, 138.13, 128.74, 127.86, 127.59, 79.31, 75.18, 60.34, 52.62, 43.24, 34.59, 28.45, 28.40, 20.99, 14.14; HRMS *m/z* (ESI) calcd for $C_{23}H_{36}N_3O_3S$ $(M + H)^+$, 434.2477, found 434.2477. IR Qmax/cm-1 (film): 699, 734, 973, 1110, 1144, 1164, 1245, 1285, 1364, 1424, 1452, 1544, 1643, 1689, 2911, 3302.

***N*-benzyl-2-(pyridin-4-yl)-2-thiomorpholinoacetamide (4j)**

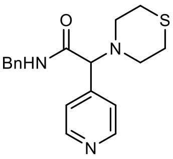

According to general procedure A, the reaction of thiomorpholine (10.3 mg, 0.1 mmol), isonicotinaldehyde (13 mg, 0.12 mmol), diethyl 4-(benzylcarbamoyl)-2,6-dimethyl-1,4-dihydropyridine-3,5-dicarboxylate (46 mg, 0.12 mmol), 4 Å molecular sieve (200 mg), TBSOTf (56 uL, 0.24 mmol) in DCM (2 mL). The crude reaction was purified by flash column chromatography (PE/Acetone = 1:1) to provide product as yellow oil (13.1 mg, 40%). **^{1}H NMR (400 MHz, CDCl$_3$)**: δ 8.62 (d, *J* = 5.1 Hz, 2H), 7.42 – 7.31 (m, 4H), 7.29 – 7.21 (m, 3H), 4.59 – 4.45 (m, 2H), 4.07 (s, 1H), 2.82 – 2.60 (m, 8H); **^{13}C NMR (101 MHz, CDCl$_3$)**: δ 169.36, 149.93, 143.28, 137.97, 128.86, 127.76, 127.68, 124.28, 75.08, 53.32,43.45, 27.98; HRMS *m/z* (ESI) calcd for $C_{18}H_{22}N_3OS$ (M + H)$^+$, 328.1484, found 328.1484. IR Qmax/cm-1 (film): 699, 734, 958, 1028, 1242, 1288, 1416, 1453, 1496, 1516, 1597, 1662, 2912, 3305.

***N*-benzyl-2-thiomorpholinohex-5-enamide (4k)**

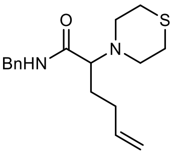

According to general procedure A, the reaction of thiomorpholine (10.3 mg, 0.1 mmol), pent-4-enal (10 mg, 0.12 mmol), diethyl 4-(benzylcarbamoyl)-2,6-dimethyl-1,4-dihydropyridine-3,5-dicarboxylate (46 mg, 0.12 mmol), 4 Å molecular sieve (200 mg), TBSOTf (28 uL, 0.12 mmol) in DCM (2 mL). The crude reaction was purified by flash column chromatography (PE/Acetone = 5:1) to provide product as yellow oil (21 mg, 70%). **^{1}H NMR (400 MHz, CDCl$_3$)**: δ 7.40 – 7.24 (m, 6H), 5.87-5.76 (m, 1H), 5.10 – 4.96 (m, 2H), 4.52 (dd, *J* = 14.7, 6.2 Hz, 1H), 4.41 (dd, *J* = 14.7, 5.6 Hz, 1H), 3.03 (dd, *J* = 7.9, 4.8 Hz, 1H), 2.91-2.85 (m, 2H), 2.82-2.76 (m, 2H), 2.70 – 2.51 (m, 4H), 2.40 – 2.26 (m, 1H), 2.23 – 2.10 (m, 1H), 1.99-1.90 (m, 1H), 1.76-1.67 (m, 1H); **^{13}C NMR (101 MHz, CDCl$_3$)**: δ 172.39, 138.56, 137.95, 128.72, 127.62, 127.47, 115.24, 68.85, 52.12, 43.24, 31.71, 28.43, 25.28.; HRMS *m/z* (ESI) calcd for $C_{17}H_{25}N_2OS$ (M + H)$^+$, 305.1688, found 305.1685. IR Qmax/cm-1 (film): 698, 727, 880, 1045, 1085, 1417, 1453, 1514, 1647, 2908, 2971, 3305.

1-((4-Methoxyphenyl)amino)-N-phenylcyclobutane-1-carboxamide (4l)

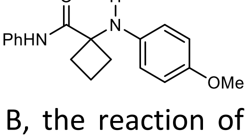

According to general procedure B, the reaction of 4-methoxyaniline (12.3 mg, 0.1 mmol), cyclobutanone (15 uL, 0.2 mmol), diethyl 2,6-dimethyl-4-(phenylcarbamoyl)-1,4-dihydropyridine-3,5-dicarboxylate (45 mg, 0.12 mmol), 4 Å molecular sieve (200 mg), $BF_3.Et_2O$ (19 uL, 0.15 mmol) in DCM (2 mL). The crude reaction was purified by flash column chromatography (PE/Acetone = 3:1) to provide product as yellow oil

(11.8mg, 40%). **^{1}H NMR (400 MHz, CDCl$_3$)**: δ 8.85 (s, 1H), 7.57 – 7.51 (m, 2H), 7.34 – 7.29 (m, 2H), 7.13 – 7.07 (m, 1H), 6.83 – 6.75 (m, 2H), 6.53 – 6.47 (m, 2H), 4.03 (s, 1H), 3.75 (s, 3H), 2.93-2.87 (m, 2H), 2.22 – 2.16 (m, 1H), 2.07 – 1.98 (m, 3H); **^{13}C NMR (101 MHz, CDCl$_3$)**: δ 173.21, 153.36, 137.96, 137.88, 128.89, 124.02, 119.62, 115.96, 114.91, 61.62, 55.61, 31.57, 14.94; HRMS *m/z* (ESI) calcd for $C_{18}H_{21}N_2O_2$ (M + H)$^+$, 297.1603, found 297.1601. IR Qmax/cm-1 (film): 693, 754, 822, 1037, 1076, 1177, 1237, 1276, 1439, 1508, 1598, 1675, 2950, 2988, 3341.

***N*,4-diphenyl-2-(piperidin-1-yl)butanamide (4m)**

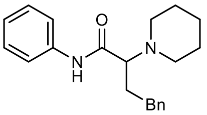

According to general procedure A, the reaction of piperidine (10 mg, 0.1 mmol), hydrocinnamaldehyde (16 uL, 0.12 mmol), diethyl 2,6-dimethyl-4-(phenylcarbamoyl)-1,4-dihydropyridine-3,5-dicarboxylate (45 mg, 0.12 mmol), 4 Å molecular sieve (200 mg), TBSOTf (28 uL, 0.12 mmol) in DCM (2 mL). The crude reaction was purified by flash column chromatography (PE/EA = 1:1) to provide product as yellow oil (23 mg, 71%). **^{1}H NMR (400 MHz, CDCl$_3$)**: δ 9.41 (s, 1H), 7.61 (d, *J* = 7.9 Hz, 2H), 7.37 (t, *J* = 7.8 Hz, 2H), 7.34 – 7.25 (m, 4H), 7.22 (t, *J* = 7.2 Hz, 1H), 7.13 (t, *J* = 7.4 Hz, 1H), 3.09 (dd, *J* = 8.0, 4.3 Hz, 1H), 3.02-2.94 (m, 1H), 2.80-2.72 (m, 1H), 2.67-2.61 (m, 2H), 2.53-2.47 (m, 2H), 2.20-2.11 (m, 1H), 2.06-1.96 (m, 1H), 1.71-1.61 (m, 4H), 1.55-1.49 (m, 2H); **^{13}C NMR (101 MHz, CDCl$_3$)**: δ 171.77, 141.88, 137.97, 128.99, 128.46, 128.36, 125.89, 123.84, 119.25, 68.80, 51.08, 33.81, 28.12, 26.70, 24.18; HRMS *m/z* (ESI) calcd for $C_{21}H_{27}N_2O$ (M + H)$^+$, 323.2123, found 323.2127. IR Qmax/cm-1 (film): 697, 752, 1307, 1438, 1514, 1599, 1688, 2810, 2848, 2933, 3302.

***N*-(2-morpholinoethyl)-4-phenyl-2-(piperidin-1-yl)butanamide (4n)**

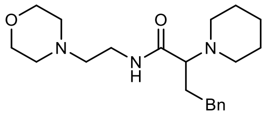

According to general procedure A, the reaction of piperidine (10 mg, 0.1 mmol), hydrocinnamaldehyde (16 uL, 0.12 mmol), diethyl 2,6-dimethyl-4-((2-morpholinoethyl)carbamoyl)-1,4-dihydropyridine-3,5-dicarboxylate (49 mg, 0.12 mmol), 4 Å molecular sieve (200 mg), TBSOTf (56 uL, 0.24 mmol) in DCM (2 mL) 24 h. The crude reaction was purified by flash column chromatography (PE/Acetone = 1:2) to provide product as yellow oil (30 mg, 84%). **^{1}H NMR (400 MHz, CDCl$_3$)**: δ 7.58 (brs, 1H), 7.31 – 7.26 (m, 2H), 7.26 – 7.17 (m, 3H), 3.72 (t, *J* = 4.6 Hz, 4H), 3.52 – 3.33 (m, 2H), 2.97 (dd, *J* = 7.9, 4.7 Hz, 1H), 2.86 (ddd, *J* = 15.1, 10.5, 5.2 Hz, 1H), 2.74 – 2.42 (m, 11H), 2.13 – 1.86 (m, 2H), 1.63-1.58 (m, 4H), 1.48 (p, *J* = 5.9 Hz, 2H); **^{13}C NMR (101 MHz, CDCl$_3$)**: δ 173.08, 142.01, 128.41, 128.31, 125.82, 68.40, 66.99, 57.36, 53.35, 51.12, 35.22, 33.42, 28.70, 26.56, 24.26; HRMS *m/z* (ESI) calcd for $C_{21}H_{34}N_3O_2$ (M + H)$^+$, 360.2651, found 360.2657. IR Qmax/cm-1 (film): 699, 750, 1030, 1117, 1146, 1273, 1296, 1453, 1503, 1669, 2808, 2851, 2932, 3331.

4-Phenyl-2-(piperidin-1-yl)-1-(pyrrolidin-1-yl)butan-1-one (4o)

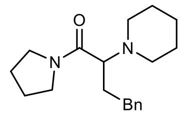

According to general procedure A, the reaction of piperidine (10 mg, 0.1 mmol), hydrocinnamaldehyde (16 uL, 0.12 mmol), diethyl 2,6-dimethyl-4-(pyrrolidine-1-carbonyl)-1,4-dihydropyridine-3,5-dicarboxylate (42 mg, 0.12 mmol), 4 Å molecular sieve (200 mg), TBSOTf (28 uL, 0.12 mmol) in DCM (2 mL). The crude reaction was purified by flash column chromatography (PE/Acetone = 5:1) to provide product as yellow oil (27 mg, 90%). **^{1}H NMR (400 MHz, CDCl$_3$)**: δ 7.28 (td, *J* = 7.3, 1.6 Hz, 2H), 7.24 – 7.15 (m, 3H), 3.64 (dt, *J* = 10.4, 6.6 Hz, 1H), 3.51 (t, *J* = 6.7 Hz, 2H), 3.31 – 3.22 (m, 2H), 2.79 – 2.58 (m, 3H), 2.56 – 2.43 (m, 3H), 2.19 (dtd, *J* = 13.0, 9.4, 5.6 Hz, 1H), 2.00 – 1.79 (m, 5H), 1.65 – 1.49 (m, 4H), 1.47 – 1.32 (m, 2H); **^{13}C NMR (101 MHz, CDCl$_3$)**: δ 170.18, 142.05, 128.40, 128.23, 125.73, 65.90, 50.43, 46.30, 45.58, 32.83, 27.71, 26.48, 26.18, 24.52, 24.18.; HRMS *m/z* (ESI) calcd for $C_{19}H_{29}N_2O$ (M + H)$^+$, 301.2280, found 301.2278. IR Qmax/cm-1 (film): 699, 751, 1112, 1338, 1434, 1636, 2873, 2929.

1-Morpholino-4-phenyl-2-(piperidin-1-yl)butan-1-one (4p)

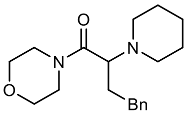

According to general procedure A, the reaction of piperidine (10 mg, 0.1 mmol), hydrocinnamaldehyde (16 uL, 0.12 mmol), diethyl 2,6-dimethyl-4-(morpholine-4-carbonyl)-1,4-dihydropyridine-3,5-dicarboxylate (44 mg, 0.12 mmol), 4 Å molecular sieve (200 mg), TBSOTf (28 uL, 0.12 mmol) in DCM (2 mL). The crude reaction was purified by flash column chromatography (PE/Acetone = 2:1) to provide product as yellow oil (27 mg, 82%). **^{1}H NMR (400 MHz, CDCl$_3$)**: δ 7.33 – 7.24 (m, 2H), 7.24 – 7.18 (m, 3H), 3.92 (ddd, *J* = 13.4, 5.2, 3.0 Hz, 1H), 3.81 – 3.58 (m, 5H), 3.51 – 3.38 (m, 2H), 3.26 (dd, *J* = 10.2, 3.5 Hz, 1H), 2.73 (ddd, *J* = 14.2, 9.7, 5.1 Hz, 1H), 2.57 (dt, *J* = 10.9, 5.3 Hz, 2H), 2.41 (ddd, *J* = 14.0, 9.6, 5.9 Hz, 3H), 2.22 (dtd, *J* = 13.0, 9.6, 5.1 Hz, 1H), 1.88 (dddd, *J* = 13.2, 10.1, 7.0, 3.5 Hz, 1H), 1.52 (p, *J* = 5.4 Hz, 4H), 1.41 (q, *J* = 6.3 Hz, 2H); **^{13}C NMR (101 MHz, CDCl$_3$)**: δ 169.94, 142.04, 128.41, 128.30, 125.80, 67.21, 67.14, 64.74, 50.09, 46.25, 42.31, 33.17, 26.61, 25.84, 24.40; HRMS *m/z* (ESI) calcd for $C_{19}H_{29}N_2O_2$ (M + H)$^+$, 317.2229, found 317.2226. IR Qmax/cm-1 (film): 700, 751, 1025, 1067, 1113, 1233, 1269, 1432, 1453, 1643, 2850, 2929.

Methyl (4-phenyl-2-(piperidin-1-yl)butanoyl)-L-tryptophanate (4q)

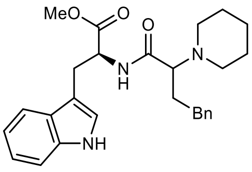

According to general procedure A, the reaction of piperidine (10 mg, 0.1 mmol), hydrocinnamaldehyde (16 uL, 0.12 mmol), diethyl (S)-4-((3-(1H-indol-3-yl)-1-methoxy-1-oxopropan-2-yl)carbamoyl)-2,6-dimethyl-1,4-dihydropyridine-3,5-dicarboxylate (60

mg, 0.12 mmol), 4 Å molecular sieve (200 mg), TBSOTf (28 uL, 0.12 mmol) in DCM (2 mL). The crude reaction was purified by flash column chromatography (PE/Acetone = 3:1) to provide product as yellow oil (32 mg, 72%, 1.1:1 dr). **^{1}H NMR (400 MHz, CDCl$_3$)**: δ 8.33 (d, *J* = 24.4 Hz, 1H), 7.89 (d, *J* = 8.4 Hz, 0.5H), 7.72 (d, *J* = 8.0 Hz, 0.55H), 7.61 (ddd, *J* = 12.2, 8.0, 1.2 Hz, 1H), 7.40 – 7.29 (m, 1H), 7.31 – 7.23 (m, 2H), 7.25 – 7.15 (m, 3H), 7.18 – 7.08 (m, 2H), 7.06 – 7.00 (m, 1H), 5.00-4.94 (m, 1H), 3.73 (d, *J* = 8.7 Hz, 3H), 3.47 – 3.27 (m, 2H), 3.02 – 2.61 (m, 3H), 2.57 – 2.46 (m, 1H), 2.40 – 2.22 (m, 3H), 2.14 – 2.01 (m, 1H), 1.95-1.85 (m, 1H), 1.46 – 1.29 (m, 6H); **^{13}C NMR (101 MHz, CDCl$_3$)**: δ 173.32, 173.25, 172.66, 172.53, 142.05, 141.85, 136.13,136.12, 128.42, 128.32, 128.27, 128.25, 127.59, 127.50, 125.77, 125.75, 122.54, 122.50, 122.16, 122.13, 119.57, 119.56, 118.54, 111.18, 111.17, 110.22, 68.45, 68.00, 52.48, 52.41, 52.23, 52.16, 51.09, 50.74, 33.65, 32.88, 29.10, 27.96, 27.67, 27.58, 26.04, 26.00, 24.10, 24.02, 14.15; HRMS *m/z* (ESI) calcd for $C_{27}H_{34}N_3O_3$ $(M + H)^+$, 448.2600, found 448.2599. IR Qmax/cm-1 (film): 700, 751, 1109, 1172, 1204, 1273, 1302, 1439, 1496, 1675, 1742, 2851, 2931, 3321.

Methyl (4-phenyl-2-(piperidin-1-yl)butanoyl)-L-methioninate (4r)

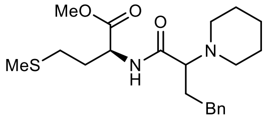

According to general procedure A, the reaction of piperidine (10 mg, 0.1 mmol), hydrocinnamaldehyde (16 uL, 0.12 mmol), diethyl (S)-4-((1-methoxy-4-(methylthio)-1-oxobutan-2-yl)carbamoyl)-2,6-dimethyl-1,4-dihydropyridine-3,5-dicarboxylate (53 mg, 0.12 mmol), 4 Å molecular sieve (200 mg), TBSOTf (28 uL, 0.12 mmol) in DCM (2 mL). The crude reaction was purified by flash column chromatography (PE/Acetone = 5:1) to provide product as yellow oil (24 mg, 61%, 1.2:1 dr). **^{1}H NMR (400 MHz, CDCl$_3$)**: δ 7.89 (d, *J* = 8.6 Hz, 0.55H), 7.76 (d, *J* = 8.4 Hz, 0.45H), 7.32 – 7.27 (m, 2H), 7.25 – 7.17 (m, 3H), 4.77-4.71 (m, 1H), 3.78 (d, *J* = 5.6 Hz, 3H), 3.03 – 2.78 (m, 2H), 2.74 – 2.40 (m, 7H), 2.29-2.17 (m, 1H), 2.12 (d, *J* = 7.4 Hz, 3H), 2.09 – 1.90 (m, 3H), 1.68 – 1.57 (m, 4H), 1.51-1.44 (m, 2H); **^{13}C NMR (101 MHz, CDCl$_3$)**: δ 173.58, 172.46, 142.09, 141.89, 128.45, 128.39, 128.35, 128.31, 125.87, 125.80, 68.62, 68.09, 52.40, 52.35, 51.35, 50.97, 50.95, 33.77, 33.34, 31.91, 31.86, 30.14, 30.06, 29.16, 27.89, 26.52, 26.48, 24.29, 24.23, 15.49, 15.48; HRMS *m/z* (ESI) calcd for $C_{21}H_{33}N_2O_3S$ $(M + H)^+$, 393.2212, found 393.2209. IR Qmax/cm-1 (film): 700, 751, 1109, 1171, 1204, 1273, 1303, 1439, 1496, 1674, 1742, 2809, 2851, 2931, 3321.

Methyl O-(tert-butyldimethylsilyl)-N-(4-phenyl-2-(piperidin-1-yl)butanoyl)-L-serinate (4s)

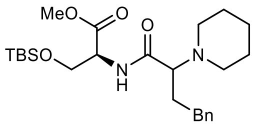

According to general procedure A, the reaction of piperidine (10 mg, 0.1 mmol), hydrocinnamaldehyde (16 uL, 0.12 mmol), diethyl (S)-4-((3-hydroxy-1-methoxy-1-

oxopropan-2-yl)carbamoyl)-2,6-dimethyl-1,4-dihydropyridine-3,5-dicarboxylate (48 mg, 0.12 mmol), 4 Å molecular sieve (200 mg), TBSOTf (28 uL, 0.12 mmol) in DCM (2 mL). The crude reaction was purified by flash column chromatography (PE/Acetone = 10:1) to provide product as yellow oil (26 mg, 56%, 1.2:1 dr). **^{1}H NMR (400 MHz, $CDCl_3$)**: δ 8.15 (d, *J* = 9.0 Hz, 0.55H), 8.01 (d, *J* = 8.7 Hz, 0.45H), 7.32 – 7.17 (m, 5H), 4.69 (ddd, *J* = 9.5, 5.2, 2.2 Hz, 1H), 4.15 (dq, *J* = 10.1, 2.3 Hz, 1H), 3.84 (ddd, *J* = 9.9, 2.9, 1.5 Hz, 2H), 3.76 (dd, *J* = 6.2, 1.6 Hz, 3H), 3.07 – 2.40 (m, 7H), 2.16 – 1.89 (m, 2H), 1.65-1.58 (m, 4H), 1.50-1.43 (m, 2H), 0.90 (dd, *J* = 3.4, 1.6 Hz, 9H), 0.08 – 0.05 (m, 6H); **^{13}C NMR (101 MHz, $CDCl_3$)**: δ 173.45, 173.36, 171.00, 170.96, 142.23, 142.03, 128.48, 128.40, 128.31, 128.28, 125.81, 125.75, 68.51, 68.01, 63.76, 63.64, 53.81, 53.75, 52.21, 52.17, 51.34, 50.91, 33.89, 33.09, 29.30, 27.83, 26.51, 25.65, 25.64, 24.36, 24.26, 18.09, -5.53, -5.56, -5.69, -5.73; HRMS *m/z* (ESI) calcd for $C_{25}H_{43}N_2O_4Si$ $(M + H)^+$, 463.2992, found 463.2991. IR Qmax/cm-1 (film): 699, 778, 835, 1112, 1154, 1210, 1253, 1471, 1496, 1681, 1751, 2855, 2931, 3364.

1,4-Diphenyl-2-(piperidin-1-yl)butan-1-one (6a):

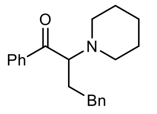

According to general procedure A, the reaction of piperidine (8.5 mg, 0.1 mmol), hydrocinnamaldehyde (16 uL, 0.12 mmol), diethyl 4-benzoyl-2,6-dimethyl-1,4-dihydropyridine-3,5-dicarboxylate (44 mg, 0.12 mmol), 4 Å molecular sieve (200 mg), TBSOTf (28 uL, 0.12 mmol) in DCM (2 mL). The crude reaction was purified by flash column chromatography (petroleum ether/Et_2O = 10:1) to provide product as pale-yellow oil (26.1 mg, 85%). **^{1}H NMR (400 MHz, $CDCl_3$):** δ 8.02 (d, *J* = 7.7 Hz, 2H), 7.57 (t, *J* = 7.4 Hz, 1H), 7.47 (t, *J* = 7.6 Hz, 2H), 7.30 (t, *J* = 7.4 Hz, 2H), 7.20 (d, *J* = 7.4 Hz, 3H), 4.07 (dd, *J* = 9.0, 5.0 Hz, 1H), 2.70-2.53 (m, 6H), 2.31 – 2.16 (m, 1H), 2.09-2.00 (m, 1H), 1.54 (p, *J* = 5.5 Hz, 4H), 1.41 (p, *J* = 6.1 Hz, 2H); **^{13}C NMR (101 MHz, $CDCl_3$):** δ 199.96, 141.88, 137.63, 132.77, 128.54, 128.43, 128.37, 128.31, 125.83, 67.56, 50.74, 32.73, 27.71, 26.53, 24.48; HRMS *m/z* (ESI) calcd for $C_{21}H_{26}NO$ $(M + H)^+$, 308.2009, found 308.1995. IR Qmax/cm-1 (film): 698, 749, 1227, 1447, 1680, 2931.

2-(azetidin-1-yl)-1,4-diphenylbutan-1-one (6b)

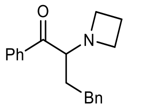

According to general procedure A, the reaction of azetidine (5.7 mg, 0.1 mmol), hydrocinnamaldehyde (16 uL, 0.12 mmol), diethyl 4-benzoyl-2,6-dimethyl-1,4-dihydropyridine-3,5-dicarboxylate (44 mg, 0.12 mmol), 4 Å molecular sieve (200 mg), TBSOTf (28 uL, 0.12 mmol) in DCM (2 mL). The crude reaction was purified by flash column chromatography (DCM/MeOH = 20:1) to provide product as pale-yellow oil (17 mg, 60%). **^{1}H NMR (400 MHz, $CDCl_3$)**: δ 8.07 – 8.03 (m, 2H), 7.59 (t, *J* = 7.4 Hz, 1H), 7.48 (t, *J* = 7.7 Hz, 2H), 7.24 (dd, *J* = 8.0, 6.5 Hz, 2H), 7.20 – 7.15 (m, 1H), 7.10 – 7.03 (m, 2H), 3.99 (dd, *J* = 7.2, 4.5 Hz, 1H), 3.36 (t, *J* = 7.0 Hz, 4H), 2.70 (ddd, *J* = 14.0, 7.0,

4.3 Hz, 1H), 2.46 (ddd, *J* = 13.9, 10.7, 6.1 Hz, 1H), 2.15 (p, *J* = 7.0 Hz, 2H), 2.11 – 1.93 (m, 2H); **^{13}C NMR (101 MHz, CDCl$_3$)**: δ 199.98, 141.51, 136.57, 133.13, 128.59, 128.54, 128.33, 128.27, 125.92, 71.73, 53.68, 31.73, 31.27, 17.78; HRMS *m/z* (ESI) calcd for $C_{19}H_{22}NO$ (M + H)$^+$, 280.1696, found 280.1700. IR Qmax/cm-1 (film): 699, 751, 1252, 1380, 1450, 1598, 1672, 1712, 2958, 3026.

1,4-diphenyl-2-(pyrrolidin-1-yl)butan-1-one (6c)

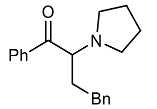

According to general procedure A, the reaction of pyrrolidine (7.1 mg, 0.1 mmol), hydrocinnamaldehyde (16 uL, 0.12 mmol), diethyl 4-benzoyl-2,6-dimethyl-1,4-dihydropyridine-3,5-dicarboxylate (44 mg, 0.12 mmol), 4 Å molecular sieve (200 mg), TBSOTf (28 uL, 0.12 mmol) in DCM (2 mL). The crude reaction was purified by flash column chromatography (DCM/MeOH = 20:1) to provide product as pale-yellow oil (19 mg, 64%). **^{1}H NMR (400 MHz, CDCl$_3$)**: δ 8.07 (d, *J* = 7.8 Hz, 2H), 7.58 (t, *J* = 7.4 Hz, 1H), 7.47 (t, *J* = 7.6 Hz, 2H), 7.29 – 7.24 (m, 3H), 7.19 (t, *J* = 7.3 Hz, 1H), 7.13 (d, *J* = 7.5 Hz, 2H), 4.11 (dd, *J* = 8.5, 4.7 Hz, 1H), 2.83 – 2.73 (m, 2H), 2.70-2.63 (m, 3H), 2.61 – 2.51 (m, 1H), 2.34-2.24 (m, 1H), 2.16 – 2.03 (m, 1H), 1.88 – 1.69 (m, 4H); **^{13}C NMR (101 MHz, CDCl$_3$)**: δ 200.67, 141.62, 137.03, 133.05, 128.56, 128.54, 128.35, 128.33, 125.90, 66.93, 50.59, 32.03, 31.76, 23.53; HRMS *m/z* (ESI) calcd for $C_{20}H_{24}NO$ (M + H)$^+$, 294.1852, found 294.1859. IR Qmax/cm-1 (film): 701, 716, 1252, 1383, 1451, 1601, 1710, 2973.

2-(azepan-1-yl)-1,4-diphenylbutan-1-one (6d)

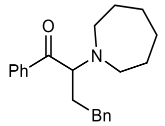

According to general procedure A, the reaction of azepane (10 mg, 0.1 mmol), hydrocinnamaldehyde (16 uL, 0.12 mmol), diethyl 4-benzoyl-2,6-dimethyl-1,4-dihydropyridine-3,5-dicarboxylate (44 mg, 0.12 mmol), 4 Å molecular sieve (200 mg), TBSOTf (28 uL, 0.12 mmol) in DCM (2 mL). The crude reaction was purified by flash column chromatography (PE/EA = 20:1) to provide product as yellow oil (23 mg, 72%). **^{1}H NMR (400 MHz, CDCl$_3$)**: δ 7.97 – 7.91 (m, 2H), 7.57 – 7.53 (m, 1H), 7.45 (t, *J* = 7.8 Hz, 2H), 7.31 (dd, *J* = 8.2, 7.0 Hz, 2H), 7.23 (d, *J* = 7.7 Hz, 3H), 4.11 (dd, *J* = 8.0, 5.7 Hz, 1H), 2.82 – 2.71 (m, 5H), 2.60 (ddd, *J* = 13.7, 9.0, 6.2 Hz, 1H), 2.23 (dtd, *J* = 14.3, 8.5, 6.3 Hz, 1H), 1.98 (ddt, *J* = 13.7, 9.4, 6.0 Hz, 1H), 1.62 – 1.55 (m, 2H), 1.54 – 1.45 (m, 6H); **^{13}C NMR (101 MHz, CDCl$_3$)**: δ 200.41, 142.09, 137.52, 132.53, 128.52, 128.50, 128.35, 128.26, 125.84, 67.65, 51.62, 32.94, 29.88, 27.91, 27.10; HRMS *m/z* (ESI) calcd for $C_{22}H_{28}NO$ (M + H)$^+$, 322.2165, found 322.2172. IR Qmax/cm-1 (film): 697, 749, 1223, 1447, 1495, 1596, 1682, 2851, 2923.

2-(4-bromopiperidin-1-yl)-1,4-diphenylbutan-1-one (6e)

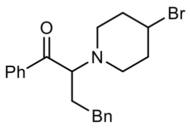

According to general procedure C, the reaction of 4-bromopiperidine hydrobromide salt (24.5 mg, 0.1 mmol), hydrocinnamaldehyde (16 uL, 0.12 mmol), Et_3N (14 uL, 0.1 mmol), diethyl 4-benzoyl-2,6-dimethyl-1,4-dihydropyridine-3,5-dicarboxylate (44 mg, 0.12 mmol), 4 Å molecular sieve (200 mg), TBSOTf (28 uL, 0.12 mmol) in DCM (2 mL). The crude reaction was purified by flash column chromatography (PE/Et_2O = 10 :1) to provide product as yellow oil (23 mg, 60%). **^{1}H NMR (400 MHz, $CDCl_3$)**: δ 7.97 – 7.93 (m, 2H), 7.62 – 7.54 (m, 1H), 7.46 (t, *J* = 7.8 Hz, 2H), 7.30 (dd, *J* = 8.2, 6.8 Hz, 2H), 7.24 – 7.16 (m, 3H), 4.15-4.07 (m, 2H), 2.96 – 2.81 (m, 2H), 2.73-2.66 (m, 1H), 2.62 – 2.52 (m, 2H), 2.49-2.43 (m, 1H), 2.27 – 2.18 (m, 1H), 2.18 – 2.08 (m, 2H), 2.06 – 1.93 (m, 3H); **^{13}C NMR (101 MHz, $CDCl_3$)**: δ 199.64, 141.58, 137.21, 133.00, 128.50, 128.44, 128.40, 125.98, 66.61, 50.05, 36.97, 32.58, 27.68; HRMS *m/z* (ESI) calcd for $C_{21}H_{25}BrNO$ $(M + H)^+$, 386.1120, found 386.1117. IR Qmax/cm-1 (film): 697, 749, 1197, 1221, 1253, 1447, 1682, 2816, 2952.

1-(1-oxo-1,4-diphenylbutan-2-yl)piperidine-4-carbonitrile (6f)

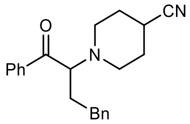

According to general procedure A, the reaction of piperidine-4-carbonitrile (11.2 mg, 0.1 mmol), hydrocinnamaldehyde (16 uL, 0.12 mmol), diethyl 4-benzoyl-2,6-dimethyl-1,4-dihydropyridine-3,5-dicarboxylate (44 mg, 0.12 mmol), 4 Å molecular sieve (200 mg), TBSOTf (28 uL, 0.12 mmol) in DCM (2 mL). The crude reaction was purified by flash column chromatography (PE/EA = 5:1) to provide product as yellow oil (23 mg, 69%). **^{1}H NMR (400 MHz, $CDCl_3$)**: δ 7.96 – 7.88 (m, 2H), 7.65 – 7.54 (m, 1H), 7.47 (t, *J* = 7.8 Hz, 2H), 7.35 – 7.29 (m, 2H), 7.25 – 7.16 (m, 3H), 4.09 (dd, *J* = 8.5, 5.6 Hz, 1H), 2.89-2.79 (m, 2H), 2.72-2.66 (m, 1H), 2.65 – 2.47 (m, 4H), 2.26-2.18 (m, 1H), 2.04-1.96 (m, 1H), 1.95-1.86 (m, 2H), 1.84-1.76 (m, 2H);; **^{13}C NMR (101 MHz, $CDCl_3$)**: δ 199.45, 141.41, 137.09, 133.09, 128.54, 128.42, 128.39, 126.03, 121.62, 66.59, 48.35, 47.28, 32.50, 29.42, 29.39, 27.55, 26.34; HRMS *m/z* (ESI) calcd for $C_{22}H_{25}N_2O$ $(M + H)^+$, 333.1967, found 333.1965. IR Qmax/cm-1 (film): 700, 751, 1225, 1446, 1595, 1681, 2815, 2929, 2950.

2-(4-morpholinopiperidin-1-yl)-1,4-diphenylbutan-1-one (6g)

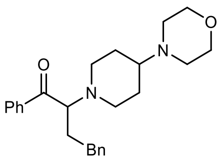

According to general procedure A, the reaction of 4-(piperidin-4-yl)morpholine (17 mg, 0.1 mmol), hydrocinnamaldehyde (16 uL, 0.12 mmol), diethyl 4-benzoyl-2,6-

dimethyl-1,4-dihydropyridine-3,5-dicarboxylate (44 mg, 0.12 mmol), 4 Å molecular sieve (200 mg), TBSOTf (28 uL, 0.12 mmol) in DCM (2 mL). The crude reaction was purified by flash column chromatography (PE/Acetone = 2:1) to provide product as yellow oil (24 mg, 62%). **^{1}H NMR (400 MHz, CDCl$_3$)**: δ 8.01 – 7.93 (m, 2H), 7.61 – 7.52 (m, 1H), 7.45 (dd, *J* = 8.4, 7.0 Hz, 2H), 7.33 – 7.27 (m, 2H), 7.24 – 7.17 (m, 3H), 4.08 (dd, *J* = 8.6, 5.4 Hz, 1H), 3.82 – 3.61 (m, 4H), 3.11 – 2.98 (m, 1H), 2.92 – 2.78 (m, 1H), 2.72 – 2.65 (m, 1H), 2.59 – 2.47 (m, 6H), 2.28 – 1.94 (m, 4H), 1.87 (dt, *J* = 12.2, 3.1 Hz, 1H), 1.76 (dt, *J* = 12.5, 3.2 Hz, 1H), 1.55 – 1.31 (m, 2H); **^{13}C NMR (101 MHz, CDCl$_3$)**: δ 199.92, 141.75, 137.39, 132.87, 128.50, 128.44, 128.42, 128.35, 125.90, 67.19, 66.80, 62.16, 49.79, 49.04, 49.02, 32.63, 28.98, 28.80, 27.81; HRMS *m/z* (ESI) calcd for $C_{25}H_{33}N_2O_2$ $(M + H)^+$, 393.2542, found 393.2539. IR Qmax/cm-1 (film): 701, 729, 750, 877, 1027, 1117, 1223, 1250, 1268, 1448, 1681, 2808, 2852, 2950.

2-morpholino-1,4-diphenylbutan-1-one (6h)

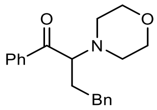

According to general procedure A, the reaction of morpholine (8.7 mg, 0.1 mmol), hydrocinnamaldehyde (16 uL, 0.12 mmol), diethyl 4-benzoyl-2,6-dimethyl-1,4-dihydropyridine-3,5-dicarboxylate (44 mg, 0.12 mmol), 4 Å molecular sieve (200 mg), TBSOTf (28 uL, 0.12 mmol) in DCM (2 mL). The crude reaction was purified by flash column chromatography (PE/EA = 5:1) to provide product as colorless oil (26.5 mg, 86%). **^{1}H NMR (400 MHz, CDCl$_3$)**: δ 7.99 (d, *J* = 7.6 Hz, 2H), 7.59 (t, *J* = 7.4 Hz, 1H), 7.47 (t, *J* = 7.7 Hz, 2H), 7.33 – 7.26 (m, 2H), 7.26 – 7.15 (m, 3H), 4.07 (dd, *J* = 8.6, 5.2 Hz, 1H), 3.67 (t, *J* = 4.6 Hz, 4H), 2.78 – 2.46 (m, 6H), 2.28 – 2.20 (m, 1H), 2.14 – 1.99 (m, 1H); **^{13}C NMR (101 MHz, CDCl$_3$)**: δ 199.42, 141.50, 137.26, 133.03, 128.51, 128.46, 128.39, 128.37, 125.97, 67.38, 67.07, 49.94, 32.40, 27.68; HRMS *m/z* (ESI) calcd for $C_{20}H_{24}NO_2$ $(M + H)^+$, 310.1802, found 310.1806. IR Qmax/cm-1 (film): 700, 749, 955, 1116, 1226, 1448, 1682, 2852, 2955.

1,4-diphenyl-2-thiomorpholinobutan-1-one (6i)

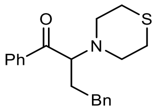

According to general procedure A, the reaction of thiomorpholine (10.3 mg, 0.1 mmol), hydrocinnamaldehyde (16 uL, 0.12 mmol), diethyl 4-benzoyl-2,6-dimethyl-1,4-dihydropyridine-3,5-dicarboxylate (44 mg, 0.12 mmol), 4 Å molecular sieve (200 mg), TBSOTf (28 uL, 0.12 mmol) in DCM (2 mL). The crude reaction was purified by flash column chromatography (PE/Et_2O = 10:1) to provide product as colorless oil (20 mg, 62%). **^{1}H NMR (400 MHz, CDCl$_3$)**: δ 7.96 – 7.89 (m, 2H), 7.61 – 7.52 (m, 1H), 7.46 (t, *J* = 7.8 Hz, 2H), 7.36 – 7.27 (m, 2H), 7.25 – 7.17 (m, 3H), 4.04 (dd, *J* = 8.6, 5.4 Hz, 1H), 2.95-2.87 (m, 4H), 2.75-2.68 (m, 1H), 2.62 – 2.51 (m, 5H), 2.27-2.19 (m, 1H), 2.05-1.97 (m, 1H); **^{13}C NMR (101 MHz, CDCl$_3$)**: δ 199.23, 141.59, 137.24, 132.94, 128.49, 128.46, 128.44, 128.40, 125.98, 67.83, 51.88, 32.63, 28.62, 27.09; HRMS *m/z* (ESI) calcd for

$C_{20}H_{24}NOS$ (M + H)$^+$, 326.1579, found 326.1580. IR Qmax/cm-1 (film): 698, 750, 949, 1211, 1229, 1279, 1447, 1681, 2908, 2951.

tert-butyl 4-(1-oxo-1,4-diphenylbutan-2-yl)piperazine-1-carboxylate (6j)

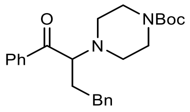

According to general procedure A, the reaction of tert-butyl piperazine-1-carboxylate (18.6 mg, 0.1 mmol), hydrocinnamaldehyde (16 uL, 0.12 mmol), diethyl 4-benzoyl-2,6-dimethyl-1,4-dihydropyridine-3,5-dicarboxylate (44 mg, 0.12 mmol), 4 Å molecular sieve (200 mg), TBSOTf (28 uL, 0.12 mmol) in DCM (2 mL). The crude reaction was purified by flash column chromatography (PE/EA = 5:1) to provide product as colorless oil (30 mg, 74%).**^{1}H NMR (400 MHz, CDCl$_3$)**: δ 7.98 – 7.94 (m, 2H), 7.60 – 7.56 (m, 1H), 7.49 – 7.43 (m, 2H), 7.34 – 7.27 (m, 2H), 7.24 – 7.20 (m, 1H), 7.19 – 7.16 (m, 2H), 4.11 (dd, *J* = 8.6, 5.4 Hz, 1H), 3.38 (s, 4H), 2.70 (ddd, *J* = 13.8, 9.3, 6.1 Hz, 1H), 2.64 – 2.50 (m, 5H), 2.22 (dtd, *J* = 13.6, 8.9, 6.1 Hz, 1H), 2.02 (dddd, *J* = 13.6, 9.4, 6.5, 5.3 Hz, 1H), 1.45 (s, 9H); **^{13}C NMR (101 MHz, CDCl$_3$)**: δ 199.47, 154.64, 141.48, 137.23, 133.06, 128.53, 128.45, 128.42, 128.40, 126.00, 79.56, 66.59, 32.47, 28.37, 27.74; HRMS *m/z* (ESI) calcd for $C_{25}H_{33}N_2O_3$ (M + H)$^+$, 409.2486, found 409.2488. IR Qmax/cm-1 (film): 700, 1119, 1170, 1245, 1364, 1420, 1453, 1687, 2931, 2973.

2-((1R,5S)-8-Oxa-3-azabicyclo[3.2.1]octan-3-yl)-1,4-diphenylbutan-1-ol (6k)

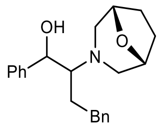

According to general procedure C, the reaction of 8-Oxa-3-azabicyclo[3.2.1]octane hydrochloride salt (16.1 mg, 0.1 mmol), hydrocinnamaldehyde (16 uL, 0.12 mmol), Et_3N (14 uL, 0.1 mmol), diethyl 4-benzoyl-2,6-dimethyl-1,4-dihydropyridine-3,5-dicarboxylate (44 mg, 0.12 mmol), 4 Å molecular sieve (200 mg), TBSOTf (28 uL, 0.12 mmol) in DCM (2 mL). After reaction, the product was reduced by $NaBH_4$ (7.6 mg, 0.2 mmol) in MeOH (2 mL). The crude reaction was purified by flash column chromatography (PE/EA = 3:1) to provide product as colorless oil (22 mg, 65%). **^{1}H NMR (400 MHz, CDCl$_3$)**: δ 7.41-7.33 (m, 5H), 7.21 (t, *J* = 7.4 Hz, 2H), 7.15 (t, *J* = 7.3 Hz, 1H), 6.92 – 6.86 (m, 2H), 4.96 (s, 1H), 4.56 – 4.27 (m, 3H), 3.04 (dd, *J* = 11.1, 2.2 Hz, 1H), 2.80 (dd, *J* = 10.9, 2.3 Hz, 1H), 2.58 (d, *J* = 10.9 Hz, 1H), 2.43 (dt, *J* = 9.7, 5.6 Hz, 1H), 2.35 (d, *J* = 11.0 Hz, 1H), 2.17 – 1.94 (m, 6H), 1.89 – 1.82 (m, 1H), 1.53 – 1.44 (m, 1H); **^{13}C NMR (101 MHz, CDCl$_3$)**: δ 141.57, 141.37, 128.42, 128.22, 128.18, 128.08, 127.65, 125.83, 74.98, 74.27, 73.89, 69.07, 57.60, 49.26, 34.13, 28.31, 28.20, 27.52; HRMS *m/z* (ESI) calcd for $C_{22}H_{28}NO_2$ (M + H)$^+$, 338.2120, found 338.2120. IR Qmax/cm-1 (film): 700, 751, 874, 993, 1027, 1068, 1454, 1494, 2948.

***Tert*-butyl 2-(1-oxo-1,4-diphenylbutan-2-yl)-2,7-diazaspiro[3.5]nonane-7-carboxylate (6l)**

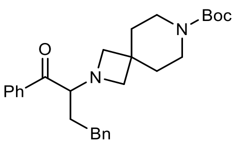

According to general procedure A, the reaction of tert-butyl 2,7-diazaspiro[3.5]nonane-7-carboxylate (22.6 mg, 0.1 mmol), hydrocinnamaldehyde (16 uL, 0.12 mmol), diethyl 4-benzoyl-2,6-dimethyl-1,4-dihydropyridine-3,5-dicarboxylate (44 mg, 0.12 mmol), 4 Å molecular sieve (200 mg), TBSOTf (28 uL, 0.12 mmol) in DCM (2 mL). The crude reaction was purified by flash column chromatography (PE/EA = 3:1) to provide product as pale-yellow oil (20 mg, 51%). **^{1}H NMR (400 MHz, CDCl$_3$)**: δ 8.04 (d, *J* = 7.5 Hz, 2H), 7.59 (t, *J* = 7.4 Hz, 1H), 7.47 (t, *J* = 7.5 Hz, 2H), 7.25 (t, *J* = 7.3 Hz, 2H), 7.18 (t, *J* = 7.2 Hz, 1H), 7.11 – 7.06 (m, 2H), 4.01 (dd, *J* = 7.4, 4.9 Hz, 1H), δ 3.33 (t, *J* = 5.6 Hz, 4H), 3.22 (d, *J* = 6.9 Hz, 2H), 3.15 (d, *J* = 6.9 Hz, 2H), 2.66 (ddd, *J* = 13.6, 10.1, 5.4 Hz, 1H), 2.50 (ddd, *J* = 13.9, 10.3, 6.1 Hz, 1H), 2.18 – 1.94 (m, 2H), 1.77 – 1.67 (m, 4H), 1.47 (s, 9H); **^{13}C NMR (101 MHz, CDCl$_3$)**: δ 200.42, 154.85, 141.41, 136.55, 133.18, 128.60, 128.58, 128.34, 128.30, 125.95, 79.40, 70.82, 61.93, 35.72, 34.67, 31.77, 31.57, 28.42; HRMS *m/z* (ESI) calcd for $C_{28}H_{37}N_2O_3$ $(M + H)^+$, 449.2799, found 449.2802. IR Qmax/cm-1 (film): 699, 1151, 1242, 1365, 1423, 1449, 1687, 2934, 2974.

2-(3,3-Difluoroazetidin-1-yl)-1,4-diphenylbutan-1-one (6m)

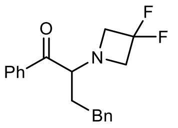

According to general procedure C, the reaction of 3,3-difluoroazetidine hydrochloride salt (13.0 mg, 0.1 mmol), hydrocinnamaldehyde (16 uL, 0.12 mmol), Et_3N (14 uL, 0.1 mmol), diethyl 4-benzoyl-2,6-dimethyl-1,4-dihydropyridine-3,5-dicarboxylate (44 mg, 0.12 mmol), 4 Å molecular sieve (200 mg), TBSOTf (28 uL, 0.12 mmol) in DCM (2 mL). The crude reaction was purified by flash column chromatography (PE/EA = 10:1) to provide product as pale-yellow oil (20 mg, 62%). **^{1}H NMR (400 MHz, CDCl$_3$)**: δ 7.97 (d, *J* = 7.8 Hz, 2H), 7.62 (t, *J* = 7.4 Hz, 1H), 7.49 (t, *J* = 7.6 Hz, 2H), 7.28 (dd, *J* = 8.6, 6.1 Hz, 2H), 7.21 (t, *J* = 7.4 Hz, 1H), 7.11 (d, *J* = 7.4 Hz, 2H), 4.12 (t, *J* = 6.1 Hz, 1H), 3.89 – 3.70 (m, 4H), 2.81 – 2.72 (m, 1H), 2.58 – 2.51 (m, 1H), 2.23 – 1.97 (m, 2H); **^{13}C NMR (101 MHz, CDCl$_3$)**: δ 199.52, 140.93, 135.94, 133.48, 128.75, 128.48, 128.44, 128.33, 126.15, 116.54 (t, *J* = 273.4 Hz), 68.77 , 62.67 (t, *J* = 23.4 Hz), 32.09, 31.24; **^{19}F NMR (376 MHz, CDCl$_3$)**: δ -100.05; HRMS *m/z* (ESI) calcd for $C_{19}H_{20}F_2NO$ $(M + H)^+$, 316.1508, found 316.1507. IR Qmax/cm-1 (film): 699, 749, 898, 1223, 1239, 1349, 1448, 1685, 2967, 3026.

***Tert*-Butyl (1-(1-oxo-1,4-diphenylbutan-2-yl)azetidin-3-yl)carbamate (6n)**

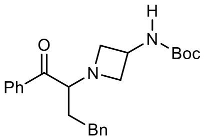

According to general procedure A, the reaction of tert-butyl azetidin-3-ylcarbamate (17.2 mg, 0.1 mmol), hydrocinnamaldehyde (16 uL, 0.12 mmol), diethyl 4-benzoyl-2,6-dimethyl-1,4-dihydropyridine-3,5-dicarboxylate (44 mg, 0.12 mmol), 4 Å molecular sieve (200 mg), TBSOTf (28 uL, 0.12 mmol) in DCM (2 mL). The crude reaction was purified by flash column chromatography (PE/EA = 2:1) to provide product as pale-yellow oil (19 mg, 50%). **^{1}H NMR (400 MHz, CDCl$_3$)**: δ 8.02 – 7.95 (m, 2H), 7.63 – 7.57 (m, 1H), 7.47 (t, *J* = 7.6 Hz, 2H), 7.30 – 7.15 (m, 4H), 7.10 – 7.04 (m, 2H), 4.39 (s, 1H), 4.02 (t, *J* = 6.0 Hz, 1H), 3.76 (dt, *J* = 18.5, 7.2 Hz, 2H), 3.19 (s, 1H), 3.12(s, 1H), 2.69 (ddd, *J* = 14.1, 10.5, 5.7 Hz, 1H), 2.49 (ddd, *J* = 14.0, 10.2, 6.0 Hz, 1H), 2.17 – 1.92 (m, 2H), 1.45 (s, 9H); **^{13}C NMR (101 MHz, CDCl$_3$)**: δ 200.04, 154.99, 141.22, 136.36, 133.32, 128.67, 128.45, 128.38, 128.31, 126.03, 69.98, 60.37, 60.01, 41.94, 31.86, 31.38, 28.33; HRMS *m/z* (ESI) calcd for $C_{24}H_{32}N_2O_3$ $(M + H)^+$, 395.2329, found 395.2328. IR Qmax/cm-1 (film): 699, 750, 1073, 1165, 1251, 1366, 1391, 1452, 1496, 1517, 1709, 1976, 2018, 2033, 2159, 2196, 2976.

2-(Benzyl(methyl)amino)-1,4-diphenylbutan-1-one (6o)

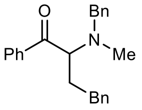

According to general procedure A, the reaction of N-methyl-1-phenylmethanamine (12.2 mg, 0.1 mmol), hydrocinnamaldehyde (16 uL, 0.12 mmol), diethyl 4-benzoyl-2,6-dimethyl-1,4-dihydropyridine-3,5-dicarboxylate (44 mg, 0.12 mmol), 4 Å molecular sieve (200 mg), TBSOTf (28 uL, 0.12 mmol) in DCM (2 mL). The crude reaction was purified by flash column chromatography (PE/Et$_2$O= 10:1) to provide product as pale-yellow oil (23 mg, 67%). **^{1}H NMR (400 MHz, CDCl$_3$)**: δ 7.84 (dd, *J* = 8.2, 1.5 Hz, 2H), 7.62 – 7.53 (m, 1H), 7.43 (t, *J* = 7.6 Hz, 2H), 7.36 – 7.15 (m, 10H), 4.22 (dd, *J* = 8.7, 5.0 Hz, 1H), 3.69 (s, 2H), 2.84-2.73 (m, 1H), 2.61-2.52 (m, 1H), 2.39 – 2.31 (m, 1H), 2.30 (s, 3H), 2.13-2.01 (m, 1H); **^{13}C NMR (101 MHz, CDCl$_3$)**: δ 200.02, 141.85, 139.19, 137.20, 132.81, 128.85, 128.59, 128.53, 128.43, 128.38, 128.21, 126.98, 125.95, 64.56, 58.36, 37.87, 32.80, 26.41; HRMS *m/z* (ESI) calcd for $C_{24}H_{26}NO$ $(M + H)^+$, 344.2014, found 344.2019. IR Qmax/cm-1 (film): 697, 744, 954, 1027, 1074, 1209, 1229, 1447, 1494, 1596, 1682, 2935.

2-(benzylamino)-1,4-diphenylbutan-1-one (6p)

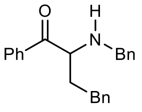

According to general procedure A, the reaction of benzylic amine (10.7 mg, 0.1 mmol), hydrocinnamaldehyde (16 uL, 0.12 mmol), diethyl 4-benzoyl-2,6-dimethyl-1,4-dihydropyridine-3,5-dicarboxylate (44 mg, 0.12 mmol), 4 Å molecular sieve (200 mg), $BF_3.Et_2O$ (19 uL, 0.15 mmol) in DCM (2 mL). The crude reaction was purified by flash column chromatography (PE/Acetone = 20:1) to provide product as pale-yellow oil (16.5 mg, 50%). **^{1}H NMR (400 MHz, CDCl$_3$)**: δ 7.72 – 7.67 (m, 2H), 7.61 – 7.53 (m, 1H), 7.43 – 7.20 (m, 10H), 7.11 – 7.05 (m, 2H), 4.17 (dd, *J* = 8.8, 3.5 Hz, 1H), 3.88 (d, *J* = 12.9

Hz, 1H), 3.58 (d, *J* = 12.9 Hz, 1H), 3.01 – 2.73 (m, 2H), 2.22 (brs, 1H), 2.07 – 1.89 (m, 1H), 1.79-1.70 (m, 1H); **^{13}C NMR (101 MHz, CDCl$_3$)**: δ 203.45, 141.48, 140.30, 135.72, 133.26, 128.73, 128.67, 128.56, 128.37, 128.16, 127.03, 125.95, 60.46, 52.35, 35.71, 32.14; HRMS *m/z* (ESI) calcd for $C_{23}H_{24}NO$ (M + H)$^+$, 330.1858, found 330.1856. IR Qmax/cm-1 (film): 697, 715, 749, 1027, 1057, 1066, 1249, 1381, 1393, 1452, 1495, 1680, 2900.

1,4-Diphenyl-2-((tetrahydro-2H-pyran-4-yl)amino)butan-1-one (6q)

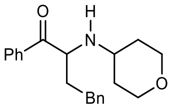

According to general procedure A, the reaction of tetrahydro-2H-pyran-4-amine (10.2 mg, 0.1 mmol), hydrocinnamaldehyde (16 uL, 0.12 mmol), diethyl 4-benzoyl-2,6-dimethyl-1,4-dihydropyridine-3,5-dicarboxylate (44 mg, 0.12 mmol), 4 Å molecular sieve (200 mg), $BF_3.Et_2O$ (19 uL, 0.15 mmol) in DCM (2 mL). The crude reaction was purified by flash column chromatography (PE/Acetone = 5:1) to provide product as pale-yellow oil (16 mg, 50%). **^{1}H NMR (400 MHz, CDCl$_3$)**: δ 7.79 – 7.74 (m, 2H), 7.64 – 7.54 (m, 1H), 7.47 – 7.40 (m, 2H), 7.36 – 7.30 (m, 2H), 7.28 – 7.17 (m, 3H), 4.23 (dd, *J* = 9.1, 3.3 Hz, 1H), 4.04 – 3.88 (m, 2H), 3.34 (qd, *J* = 11.5, 2.5 Hz, 2H), 2.99 – 2.77 (m, 2H), 2.60-2.52 (m, 1H), 2.05-1.96 (m, 2H), 1.83 – 1.64 (m, 3H), 1.61 – 1.43 (m, 2H); **^{13}C NMR (101 MHz, CDCl$_3$)**: δ 203.61, 141.39, 135.37, 133.39, 128.78, 128.75, 128.41, 128.17, 126.09, 66.61, 66.49, 58.13, 52.73, 35.87, 34.41, 33.57, 32.16; HRMS *m/z* (ESI) calcd for $C_{21}H_{26}NO_2$ (M + H)$^+$, 324.1964, found 324.1960. IR Qmax/cm-1 (film): 699, 723, 1096, 1142, 1238, 1381, 1448, 1495, 1596, 1679, 2847, 2934.

Benzyl 4-((1-oxo-1,4-diphenylbutan-2-yl)amino)piperidine-1-carboxylate (6r)

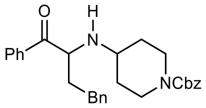

According to general procedure A, the reaction of benzyl 4-aminopiperidine-1-carboxylate (23.4 mg, 0.1 mmol), hydrocinnamaldehyde (16 uL, 0.12 mmol), diethyl 4-benzoyl-2,6-dimethyl-1,4-dihydropyridine-3,5-dicarboxylate (44 mg, 0.12 mmol), 4 Å molecular sieve (200 mg), $BF_3.Et_2O$ (19 uL, 0.15 mmol) in DCM (2 mL). The crude reaction was purified by flash column chromatography (PE/EA = 5:1) to provide product as pale-yellow oil (20 mg, 55%). **^{1}H NMR (400 MHz, CDCl$_3$)**: δ 7.75 (d, *J* = 7.0 Hz, 2H), 7.62 – 7.55 (m, 1H), 7.48 – 7.18 (m, 12H), 5.15 (s, 2H), 4.23 (dd, *J* = 9.1, 3.2 Hz, 1H), 4.08 (s, 2H), 2.98 – 2.77 (m, 4H), 2.52 (tt, *J* = 9.8, 3.8 Hz, 1H), 2.31 (s, 1H), 2.04 – 1.96 (m, 1H), 1.85 – 1.63 (m, 3H), 1.50 – 1.35 (m, 2H); **^{13}C NMR (101 MHz, CDCl$_3$)**: δ 203.42, 155.21, 141.29, 136.86, 135.28, 133.42, 128.74, 128.43, 128.41, 128.16, 127.90, 127.80, 126.11, 66.99, 58.37, 53.27, 42.47, 42.29, 35.80, 32.13; HRMS *m/z* (ESI) calcd for $C_{29}H_{33}N_2O_3$ (M + H)$^+$, 457.2486, found 457.2486. IR Qmax/cm-1 (film): 698, 732, 1228, 1274, 1367, 1431, 1447, 1696, 2860, 2941.

2-(((3s,5s,7s)-adamantan-1-yl)amino)-1,4-diphenylbutan-1-one (6s)

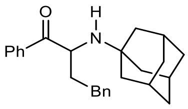

According to general procedure A, the reaction of (1s,3s)-adamantan-1-amine (15.1 mg, 0.1 mmol), hydrocinnamaldehyde (27 uL, 0.2 mmol), diethyl 4-benzoyl-2,6-dimethyl-1,4-dihydropyridine-3,5-dicarboxylate (44 mg, 0.12 mmol), 4 Å molecular sieve (200 mg), $BF_3.Et_2O$ (19 uL, 0.15 mmol) in DCM (2 mL). The crude reaction was purified by flash column chromatography (PE/EA = 10:1) to provide product as pale-yellow oil (15 mg, 40%). **^{1}H NMR (400 MHz, $CDCl_3$)**: δ 7.84 – 7.78 (m, 2H), 7.57 (t, *J* = 7.4 Hz, 1H), 7.44 (t, *J* = 7.7 Hz, 2H), 7.29 (d, *J* = 7.5 Hz, 2H), 7.21 (t, *J* = 4.8 Hz, 3H), 4.28 (dd, *J* = 9.3, 2.9 Hz, 1H), 2.98-2.91 (m, 1H), 2.82-2.74 (m, 1H), 2.07 – 1.84 (m, 5H), 1.68-1.54 (m, 10H), 1.54 – 1.47 (m, 3H); **^{13}C NMR (101 MHz, $CDCl_3$)**: δ 204.44, 141.85, 134.93, 133.18, 128.74, 128.31, 128.27, 125.89, 53.59, 50.55, 43.78, 36.98, 36.58, 32.49, 29.59; HRMS *m/z* (ESI) calcd for $C_{26}H_{32}NO$ $(M + H)^+$, 374.2484, found 374.2481. IR Qmax/cm-1 (film): 697, 745, 1101, 1145, 1234, 1254, 1265, 1447, 1682, 2846, 2902.

2-(4-(3-chlorodibenzo[b,f][1,4]oxazepin-11-yl)piperazin-1-yl)-1,4-diphenylbutan-1-one (6t)

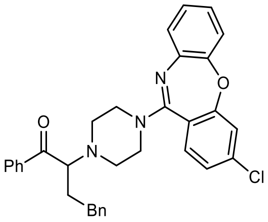

According to general procedure A, the reaction of amoxapine (32 mg, 0.1 mmol), hydrocinnamaldehyde (27 uL, 0.2 mmol), diethyl 4-benzoyl-2,6-dimethyl-1,4-dihydropyridine-3,5-dicarboxylate (44 mg, 0.12 mmol), 4 Å molecular sieve (200 mg), TBSOTf (28 uL, 0.12 mmol) in DCM (2 mL). The crude reaction was purified by flash column chromatography (PE/Acetone = 10:1) to provide product as yellow oil (50 mg, 93%). **^{1}H NMR (400 MHz, $CDCl_3$)**: δ 8.01 (d, *J* = 7.2 Hz, 2H), 7.60 (t, *J* = 7.4 Hz, 1H), 7.49 (t, *J* = 7.6 Hz, 2H), 7.39 (dd, *J* = 8.6, 2.6 Hz, 1H), 7.34 – 7.26 (m, 3H), 7.26 – 7.08 (m, 7H), 7.00 (td, *J* = 7.5, 1.8 Hz, 1H), 4.19 (dd, *J* = 8.5, 5.5 Hz, 1H), 3.50 (s, 4H), 2.89 – 2.66 (m, 5H), 2.64-2.56 (m, 1H), 2.27 (dtd, *J* = 14.8, 8.6, 6.2 Hz, 1H), 2.10 (ddt, *J* = 12.6, 9.2, 6.0 Hz, 1H); **^{13}C NMR (101 MHz, $CDCl_3$)**: δ 199.79, 159.23, 158.92, 151.77, 141.44, 140.10, 137.13, 133.11, 132.43, 130.17, 129.05, 128.55, 128.47, 128.45, 128.40, 127.03, 126.01, 125.71, 124.93, 124.45, 122.60, 120.02, 66.57, 49.23, 47.91, 32.45, 27.99; HRMS *m/z* (ESI) calcd for $C_{33}H_{31}ClN_3O_2$ $(M + H)^+$, 536.2105, found 536.2105. IR Qmax/cm-1 (film): 700, 731, 751, 773, 908, 1101, 1141, 1179, 1212, 1241, 1305, 1402, 1449, 1469, 1557, 1586, 1598, 1680, 2850, 2928.

2-(((1S,4S)-4-(3,4-dichlorophenyl)-1,2,3,4-tetrahydronaphthalen-1-yl)(methyl)amino)-1,4-diphenylbutan-1-one (6u)

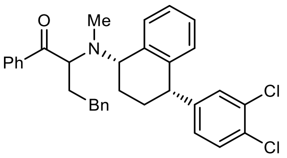

According to general procedure A, the reaction of sertraline hydrochloride salt (34.3 mg, 0.1 mmol), hydrocinnamaldehyde (27 uL, 0.2 mmol), Et_3N (14 uL, 0.1 mmol), diethyl 4-benzoyl-2,6-dimethyl-1,4-dihydropyridine-3,5-dicarboxylate (44 mg, 0.12 mmol), 4 Å molecular sieve (200 mg), TBSOTf (28 uL, 0.12 mmol) in DCM (2 mL). The crude reaction was purified by flash column chromatography (PE/Et_2O = 10:1) to provide product as yellow oil (26 mg, 50%, 1.1:1 dr). **^{1}H NMR (400 MHz, $CDCl_3$)**: δ 8.03 – 7.85 (m, 3H), 7.60-7.56 (m, 1H), 7.48 (t, *J* = 7.5 Hz, 3H), 7.40 – 7.19 (m, 8H), 7.17 – 7.08 (m, 1H), 6.94 – 6.66 (m, 1H), 4.45 – 3.98 (m, 1H), 3.02 (t, *J* = 7.2 Hz, 2H), 2.77 (t, *J* = 7.6 Hz, 3H), 2.59 – 2.35 (m, 1H), 2.25-2.23 (m, 1H), 2.17-2.04 (m, 3H), 2.02 – 1.37 (m, 3H); **^{13}C NMR (101 MHz, $CDCl_3$)**: δ 201.52, 200.06, 147.33, 147.20, 141.75, 141.67, 141.65, 139.21, 138.52, 138.37, 138.22, 137.28, 136.97, 132.92, 132.90, 132.72, 132.07, 132.02, 130.65, 130.62, 130.21, 130.19, 129.95, 129.92, 128.59, 128.56, 128.52, 128.48, 128.46, 128.44, 128.40, 128.36, 128.31, 128.10, 128.06, 127.97, 127.03, 126.90, 126.82, 126.00, 125.97, 125.91, 65.23, 63.71, 61.85, 60.22, 43.72, 43.32, 37.64, 35.16, 33.04, 32.92, 32.31, 30.90, 30.46, 29.94, 29.57, 29.17, 25.66, 20.44; dr = 2:1; HRMS *m/z* (ESI) calcd for $C_{33}H_{32}Cl_2NO$ $(M + H)^+$, 528.1861, found 528.1857. IR Qmax/cm-1 (film): 698, 743, 1029, 1226, 1448, 1467, 1495, 1597, 1682, 2936, 3025.

Ethyl 1-cyclopropyl-6-fluoro-4-oxo-7-(4-(1-oxo-1,4-diphenylbutan-2-yl)piperazin-1-yl)-1,4-dihydroquinoline-3-carboxylate (6v)

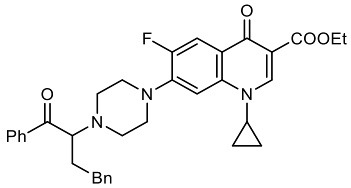

According to general procedure A, the reaction of ciprofloxacin (36 mg, 0.1 mmol), hydrocinnamaldehyde (27 uL, 0.2 mmol), diethyl 4-benzoyl-2,6-dimethyl-1,4-dihydropyridine-3,5-dicarboxylate (44 mg, 0.12 mmol), 4 Å molecular sieve (200 mg), TBSOTf (28 uL, 0.12 mmol) in DCM (2 mL). The crude reaction was purified by flash column chromatography (PE/EA = 1:5) to provide product as yellow oil (37 mg, 64%). **^{1}H NMR (400 MHz, $CDCl_3$)**: δ 8.48 (s, 1H), 8.03 – 7.78 (m, 3H), 7.59 (t, *J* = 7.4 Hz, 1H), 7.48 (t, *J* = 7.6 Hz, 2H), 7.37 – 7.26 (m, 2H), 7.25 – 7.16 (m, 4H), 4.38 (q, *J* = 7.1 Hz, 2H), 4.19 (dd, *J* = 8.6, 5.2 Hz, 1H), 3.43-3.37 (m, 1H), 3.27-3.18 (m, 4H), 2.92-2.80 (m, 4H), 2.76-2.68 (m, 1H), 2.62-2.55 (m, 1H), 2.33-2.23 (m, 1H), 2.19 – 2.05 (m, 1H), 1.40 (t, *J* = 7.1 Hz, 3H), 1.29 (dd, *J* = 7.0, 5.0 Hz, 2H), 1.14-1.09 (m, 2H); **^{13}C NMR (101 MHz, $CDCl_3$)**: δ 199.25, 173.06, 173.04, 165.72, 154.50, 152.02, 147.99, 144.43 (d, *J* = 10.4 Hz), 141.40, 137.88, 137.15, 133.12, 128.56, 128.46, 128.39, 126.01, 122.83 (d, *J* = 6.9 Hz), 113.09 (d, *J* = 22.9 Hz), 110.22, 104.70 (d, *J* = 4.0 Hz), 66.38, 60.75, 50.45, 50.40, 49.18, 34.41, 32.42, 27.72, 14.37, 8.05; **^{19}F NMR (376 MHz, $CDCl_3$)**: δ -133.52; HRMS

m/z (ESI) calcd for $C_{35}H_{37}FN_3O_4$ (M + H)$^+$, 582.2768, found 582.2763. IR Qmax/cm-1 (film): 700, 729, 1163, 1215, 1256, 1310, 1451, 1475, 1492, 1620, 1683, 1723, 2830.

2-(4-(8-Chloro-5,6-dihydro-11H-benzo[5,6]cyclohepta[1,2-b]pyridin-11-ylidene)piperidin-1-yl)-1,4-diphenylbutan-1-one (6w)

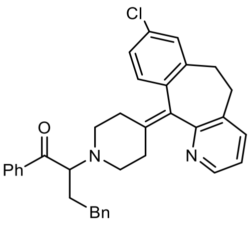

According to general procedure A, the reaction of desloratidine (32 mg, 0.1 mmol), hydrocinnamaldehyde (27 uL, 0.2 mmol), diethyl 4-benzoyl-2,6-dimethyl-1,4-dihydropyridine-3,5-dicarboxylate (44 mg, 0.12 mmol), 4 Å molecular sieve (200 mg), TBSOTf (28 uL, 0.12 mmol) in DCM (2 mL). The crude reaction was purified by flash column chromatography (PE/EA = 1:1) to provide product as yellow foam (32 mg, 56%). **^{1}H NMR (400 MHz, CDCl$_3$)**: δ 8.44 – 8.37 (m, 1H), 7.98 (dd, *J* = 7.5, 2.3 Hz, 2H), 7.56 (td, *J* = 7.3, 1.6 Hz, 1H), 7.50 – 7.38 (m, 3H), 7.28 (t, *J* = 7.3 Hz, 2H), 7.23 – 7.06 (m, 7H), 4.13 (dt, *J* = 9.1, 4.8 Hz, 1H), 3.48 – 3.29 (m, 2H), 2.97 – 2.63 (m, 5H), 2.61 – 2.12 (m, 8H), 2.02 (ddd, *J* = 14.1, 9.5, 5.2 Hz, 1H); **^{13}C NMR (101 MHz, CDCl$_3$)**: δ 200.00, 199.96, 157.52, 157.47, 146.49, 146.46, 141.68, 139.47, 139.43, 138.81, 138.78, 137.77, 137.69, 137.35, 137.23, 133.39, 133.36, 132.92, 132.87, 132.65, 132.63, 132.59, 132.57, 130.80, 130.77, 128.87, 128.46, 128.43, 128.41, 128.40, 128.33, 125.91, 125.89, 122.05, 122.02, 66.60, 51.29, 51.13, 50.69, 50.50, 32.62, 31.75, 31.70, 31.59, 31.52, 31.41, 31.36, 28.12, 27.92.; HRMS *m/z* (ESI) calcd for $C_{35}H_{34}ClN_2O$ (M + H)$^+$, 533.2360, found 533.2359. IR Qmax/cm-1 (film): 699, 730, 828, 1437, 1679, 1977, 2005, 2025, 2158, 2921.

Tert-butyl 4-(1-oxo-1-phenylhex-5-en-2-yl)piperazine-1-carboxylate (6x)

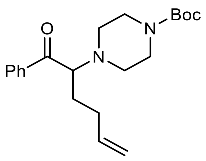

According to general procedure A, the reaction of tert-butyl piperazine-1-carboxylate (18.6 mg, 0.1 mmol), pent-4-enal (10 mg, 0.12 mmol), diethyl 4-benzoyl-2,6-dimethyl-1,4-dihydropyridine-3,5-dicarboxylate (44 mg, 0.12 mmol), 4 Å molecular sieve (200 mg), TBSOTf (28 uL, 0.12 mmol) in DCM (2 mL). The crude reaction was purified by flash column chromatography (PE/EA = 10:1) to provide product as yellow oil (20 mg, 56%). **^{1}H NMR (400 MHz, CDCl$_3$)**: δ 8.04 – 7.99 (m, 2H), 7.61 – 7.55 (m, 1H), 7.48 (dd, *J* = 8.4, 7.0 Hz, 2H), 5.86-5.75 (m, 1H), 5.04 – 4.96 (m, 2H), 4.14 (dd, *J* = 8.3, 5.1 Hz, 1H), 3.37 (t, *J* = 5.1 Hz, 4H), 2.61-2.53 (m, 4H), 2.21 – 1.91 (m, 3H), 1.82-1.72 (m, 1H), 1.45 (s, 9H); **^{13}C NMR (101 MHz, CDCl$_3$)**: δ 199.43, 154.65, 137.82, 137.35, 133.04, 128.53, 128.46, 115.33, 79.56, 66.75, 30.54, 28.38, 25.15; HRMS *m/z* (ESI) calcd for $C_{21}H_{31}N_2O_3$ (M + H)$^+$, 359.2335, found 359.2332. IR Qmax/cm-1 (film): 703, 1002, 1122, 1171, 1245,

1280, 1365, 1420, 1447, 1688, 2928, 2974.

Tert-butyl 4-(6-chloro-1-oxo-1-phenylhexan-2-yl)piperazine-1-carboxylate (6y)

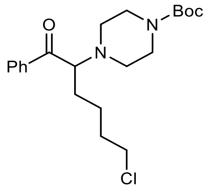

According to general procedure A, the reaction of tert-butyl piperazine-1-carboxylate (18.6 mg, 0.1 mmol), 5-chloropentanal (14.5 mg, 0.12 mmol), diethyl 4-benzoyl-2,6-dimethyl-1,4-dihydropyridine-3,5-dicarboxylate (44 mg, 0.12 mmol), 4 Å molecular sieve (200 mg), TBSOTf (28 uL, 0.12 mmol) in DCM (2 mL). The crude reaction was purified by flash column chromatography (PE/EA = 10:1) to provide product as yellow oil (20 mg, 51%). **^{1}H NMR (400 MHz, $CDCl_3$)**: δ 8.03 (dd, *J* = 8.4, 1.4 Hz, 2H), 7.63 – 7.54 (m, 1H), 7.50 – 7.46 (m, 2H), 4.10 (dd, *J* = 8.8, 5.0 Hz, 1H), 3.64 – 3.44 (m, 2H), 3.38 (t, *J* = 5.0 Hz, 4H), 2.64-2.51 (m, 4H), 1.97 – 1.67 (m, 4H), 1.45 (s, 9H), 1.42 – 1.35 (m, 2H); **^{13}C NMR (101 MHz, $CDCl_3$)**: δ 199.28, 154.61, 137.17, 133.13, 128.57, 128.47, 79.59, 67.63, 49.38, 44.65, 32.57, 28.36, 25.36, 23.95; HRMS *m/z* (ESI) calcd for $C_{21}H_{32}ClN_2O_3$ $(M + H)^+$, 395.2101, found 395.2099. IR Qmax/cm-1 (film): 651, 702, 768, 866, 1002, 1119, 1169, 1246, 1365, 1420, 1447, 1595, 1684, 2862, 2931, 2972.

tert-butyl 4-(2-oxo-2-phenylethyl)piperazine-1-carboxylate (6z)

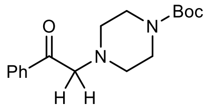

According to general procedure A, the reaction of tert-butyl piperazine-1-carboxylate (18.6 mg, 0.1 mmol), formaldehyde solution (37% in water) (25 mg, 0.3 mmol), diethyl 4-benzoyl-2,6-dimethyl-1,4-dihydropyridine-3,5-dicarboxylate (44 mg, 0.12 mmol), 4 Å molecular sieve (200 mg), TBSOTf (28 uL, 0.12 mmol) in DCM (2 mL). The crude reaction was purified by flash column chromatography (PE/EA = 1:1) to provide product as yellow oil (22 mg, 73%). **^{1}H NMR (400 MHz, $CDCl_3$)**: δ 8.02 – 7.98 (m, 2H), 7.62 – 7.56 (m, 1H), 7.48 (dd, *J* = 8.3, 7.0 Hz, 2H), 3.87 (s, 2H), 3.53 (t, *J* = 5.1 Hz, 4H), 2.59 (t, *J* = 5.0 Hz, 4H), 1.48 (s, 9H); **^{13}C NMR (101 MHz, $CDCl_3$)**: δ 196.01, 154.70, 135.88, 133.35, 128.58, 128.04, 79.70, 64.20, 53.17, 30.89, 28.39; HRMS *m/z* (ESI) calcd for $C_{17}H_{25}N_2O_3$ $(M + H)^+$, 305.1865, found 305.1860. IR Qmax/cm-1 (film): 690, 756, 969, 1003, 1124, 1168, 1245, 1278, 1365, 1392, 1420, 1449, 1690, 2930, 2975.

tert-butyl 4-(1-cyclobutyl-2-oxo-2-phenylethyl)piperazine-1-carboxylate (6aa)

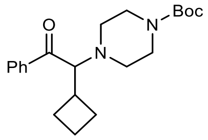

According to general procedure A, the reaction of tert-butyl piperazine-1-carboxylate (18.6 mg, 0.1 mmol), cyclobutanecarbaldehyde (10.1 mg, 0.12 mmol), diethyl 4-

benzoyl-2,6-dimethyl-1,4-dihydropyridine-3,5-dicarboxylate (44 mg, 0.12 mmol), 4 Å molecular sieve (200 mg), TBSOTf (28 uL, 0.12 mmol) in DCM (2 mL). The crude reaction was purified by flash column chromatography (PE/EA = 1:1) to provide product as yellow oil (22 mg, 63%). **^{1}H NMR (400 MHz, CDCl$_3$)**: δ 8.10 (d, *J* = 7.2 Hz, 2H), 7.58 (t, *J* = 7.4 Hz, 1H), 7.47 (t, *J* = 7.6 Hz, 2H), 4.01 – 3.87 (m, 1H), 3.37 (s, 4H), 2.95 (q, *J* = 8.6 Hz, 1H), 2.58 (d, *J* = 36.5 Hz, 3H), 2.20-2.14 (m, 1H), 2.02-1.92 (m, 2H), 1.88-1.80 (m, 2H), 1.67-1.57 (m, 2H), 1.45 (s, 9H); **^{13}C NMR (101 MHz, CDCl$_3$)**: δ 199.88, 154.67, 137.45, 133.04, 128.60, 128.46, 79.59, 50.37, 34.95, 29.69, 29.27, 28.38, 26.26, 19.25; HRMS *m/z* (ESI) calcd for $C_{21}H_{31}N_2O_3$ (M + H)$^+$, 359.2335, found 359.2331. IR Qmax/cm-1 (film): 688, 707, 732, 767, 1001, 1130, 1170, 1247, 1279, 1306, 1365, 1420, 1447, 1477, 1690, 2858, 2931, 2973.

tert-butyl 4-(1-(1-(tert-butoxycarbonyl)azetidin-3-yl)-2-oxo-2-phenylethyl)piperazine-1-carboxylate (6ab)

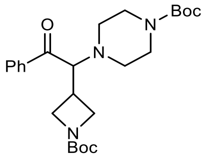

According to general procedure A, the reaction of tert-butyl piperazine-1-carboxylate (18.6 mg, 0.1 mmol), tert-butyl 3-formylazetidine-1-carboxylate (22 mg, 0.12 mmol), diethyl 4-benzoyl-2,6-dimethyl-1,4-dihydropyridine-3,5-dicarboxylate (44 mg, 0.12 mmol), 4 Å molecular sieve (200 mg), TBSOTf (28 uL, 0.12 mmol) in DCM (2 mL). The crude reaction was purified by flash column chromatography (PE/Acetone = 3:1) to provide product as yellow oil (24 mg, 52%). **^{1}H NMR (400 MHz, CDCl$_3$)**: δ 8.01 (dd, *J* = 8.0, 1.5 Hz, 2H), 7.65 – 7.58 (m, 1H), 7.50 (t, *J* = 7.7 Hz, 2H), 4.38 (d, *J* = 10.7 Hz, 1H), 4.16 – 4.06 (m, 1H), 3.99 (t, *J* = 8.7 Hz, 1H), 3.90 (dd, *J* = 8.8, 5.7 Hz, 1H), 3.42 (dd, *J* = 9.0, 5.7 Hz, 1H), 3.36 – 3.30 (m, 4H), 3.24-3.15 (m, 1H), 2.61 – 2.44 (m, 4H), 1.44 (s, 9H), 1.42 (s, 9H); **^{13}C NMR (101 MHz, CDCl$_3$)**: δ 198.50, 156.16, 154.54, 133.53, 129.01, 128.69, 128.55, 79.71, 79.48, 28.35, 28.32, 27.04; HRMS *m/z* (ESI) calcd for $C_{25}H_{38}N_3O_5$ (M + H)$^+$, 460.2811, found 460.2807. IR Qmax/cm-1 (film): 1141, 1167, 1247, 1278, 1365, 1408, 1448, 1692, 2882, 2931, 2974.

Benzyl 4-((2-oxo-2-phenyl-1-(tetrahydro-2H-pyran-4-yl)ethyl)amino)piperidine-1-carboxylate (6ac)

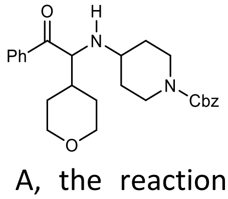

According to general procedure A, the reaction of benzyl 4-aminopiperidine-1-carboxylate (23.4 mg, 0.1 mmol), tetrahydro-2H-pyran-4-carbaldehyde (14 mg, 0.12 mmol), diethyl 4-benzoyl-2,6-dimethyl-1,4-dihydropyridine-3,5-dicarboxylate (44 mg, 0.12 mmol), 4 Å molecular sieve (200 mg), $BF_3.Et_2O$ (19 uL, 0.15 mmol) in DCM (2 mL). The crude reaction was purified by flash column chromatography (PE/Acetone = 5:1) to provide product as yellow oil (17.4 mg, 40%). **^{1}H NMR (400 MHz, CDCl$_3$)**: δ 8.04 –

7.95 (m, 2H), 7.73 – 7.60 (m, 1H), 7.57 – 7.50 (m, 2H), 7.38 – 7.34 (m, 5H), 5.13 (s, 2H), 4.13 (d, *J* = 4.5 Hz, 1H), 4.07 – 3.89 (m, 4H), 3.35-3.22 (m, 2H), 3.01 – 2.89 (m, 2H), 2.49 (tt, *J* = 9.3, 3.7 Hz, 1H), 1.88 – 1.73 (m, 4H), 1.52 – 1.34 (m, 6H); **^{13}C NMR (101 MHz, CDCl$_3$)**: δ 203.93, 155.21, 136.85, 136.42, 133.64, 128.96, 128.44, 128.12, 127.91, 127.81, 67.93, 67.70, 66.99, 42.27, 42.09, 39.25, 30.37, 27.94; HRMS *m/z* (ESI) calcd for $C_{26}H_{33}N_2O_4$ $(M + H)^+$, 437.2440, found 437.2441. IR Qmax/cm-1 (film): 697, 730, 1015, 1089, 1122, 1137, 1226, 1273, 1365, 1431, 1445, 1468, 1696, 2848, 2942.

Benzyl 4-((1-(1-(tert-butoxycarbonyl)piperidin-4-yl)-2-oxo-2-phenylethyl)amino)piperidine-1-carboxylate (6ad)

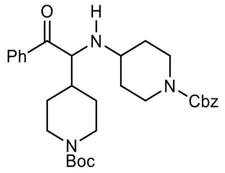

According to general procedure A, the reaction of benzyl 4-aminopiperidine-1-carboxylate (23.4 mg, 0.1 mmol), tert-butyl 4-formylpiperidine-1-carboxylate (25.6 mg, 0.12 mmol), diethyl 4-benzoyl-2,6-dimethyl-1,4-dihydropyridine-3,5-dicarboxylate (44 mg, 0.12 mmol), 4 Å molecular sieve (200 mg), $BF_3.Et_2O$ (19 uL, 0.15 mmol) in DCM (2 mL). The crude reaction was purified by flash column chromatography (PE/Acetone = 5:1) to provide product as yellow oil (25 mg, 47%). **^{1}H NMR (400 MHz, CDCl$_3$)**: δ 7.96 (d, *J* = 8.2 Hz, 2H), 7.70 – 7.61 (m, 1H), 7.53 (t, *J* = 7.7 Hz, 2H), 7.40 – 7.31 (m, 5H), 5.12 (s, 2H), 4.26 – 3.90 (m, 5H), 2.92 (brs, 2H), 2.68 – 2.42 (m, 3H), 1.86 – 1.66 (m, 3H), 1.61 – 1.52 (m, 2H), 1.45 (s, 9H), 1.39 – 1.21 (m, 5H); **^{13}C NMR (101 MHz, CDCl$_3$)**: δ 203.89, 155.19, 154.65, 136.83, 136.29, 133.67, 128.98, 128.43, 128.13, 127.90, 127.80, 79.35, 66.98, 63.23, 60.36, 53.48, 42.29, 42.10, 40.26, 29.67, 28.41; HRMS *m/z* (ESI) calcd for $C_{31}H_{42}N_3O_5$ $(M + H)^+$, 536.3124, found 536.3122. IR Qmax/cm-1 (film): 730, 1134, 1169, 1226, 1275, 1364, 1426, 1446, 1468, 1686, 2854, 2932.

Benzyl 4-((2-oxo-2-phenyl-1-(pyridin-4-yl)ethyl)amino)piperidine-1-carboxylate (6ae)

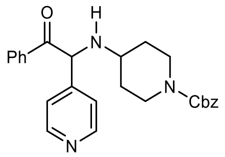

According to general procedure A, the reaction of benzyl 4-aminopiperidine-1-carboxylate (23.4 mg, 0.1 mmol), isonicotinaldehyde (13 mg, 0.12 mmol), diethyl 4-benzoyl-2,6-dimethyl-1,4-dihydropyridine-3,5-dicarboxylate (44 mg, 0.12 mmol), 4 Å molecular sieve (200 mg), $BF_3.Et_2O$ (19 uL, 0.15 mmol) in DCM (2 mL). The crude reaction was purified by flash column chromatography (PE/Acetone = 3:1) to provide product as yellow oil (25 mg, 32%). **^{1}H NMR (400 MHz, CDCl$_3$)**: δ 8.71 – 8.61 (m, 2H), 7.92 – 7.86 (m, 2H), 7.72-7.68 (m, 1H), 7.58 – 7.51 (m, 4H), 7.41 – 7.35 (m, 5H), 5.16 (s, 2H), 4.23-4.05 (m, 3H), 3.59 (tt, *J* = 8.7, 4.2 Hz, 1H), 3.02 (t, *J* = 11.8 Hz, 2H), 1.77-1.69 (m, 5H); **^{13}C NMR (101 MHz, CDCl$_3$)**: δ 197.32, 163.68, 155.26, 150.38, 141.92,

136.79, 135.29, 134.33, 129.48, 129.14, 128.47, 127.97, 127.84, 121.12, 67.09, 59.58, 41.45; HRMS *m/z* (ESI) calcd for $C_{26}H_{26}N_3O_3$ $(M - H)^-$, 428.1969, found 428.1947. IR Qmax/cm-1 (film): 697, 732, 1135, 1229, 1274, 1317, 1431, 1448, 1470, 1594, 1673, 1697, 2855, 2924, 2946.

(1-((4-Methoxyphenyl)amino)cyclobutyl)(phenyl)methanone (6af)

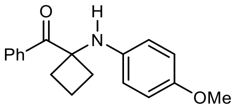

According to general procedure B, the reaction of 4-methoxyaniline (12.3 mg, 0.1 mmol), cyclobutanone (14 mg, 0.2 mmol), diethyl 4-benzoyl-2,6-dimethyl-1,4-dihydropyridine-3,5-dicarboxylate (44 mg, 0.12 mmol), 4 Å molecular sieve (200 mg), $BF_3.Et_2O$ (19 uL, 0.15 mmol) in DCM (2 mL). The crude reaction was purified by flash column chromatography (PE/DCM = 1:3) to provide product as yellow oil (15.2 mg, 54%). **^{1}H NMR (400 MHz, $CDCl_3$)**: δ 7.98 (d, *J* = 7.2 Hz, 2H), 7.46 (t, *J* = 7.4 Hz, 1H), 7.36 (t, *J* = 7.7 Hz, 2H), 6.66 (d, *J* = 8.8 Hz, 2H), 6.38 (d, *J* = 8.9 Hz, 2H), 4.16 (brs, 1H), 3.69 (s, 3H), 3.01 (ddd, *J* = 12.0, 9.1, 5.3 Hz, 2H), 2.26 (ddd, *J* = 12.0, 9.6, 7.1 Hz, 2H), 2.16-2.07 (m, 1H), 2.05 – 1.92 (m, 1H).; **^{13}C NMR (101 MHz, $CDCl_3$)**: δ 202.79, 152.45, 139.29, 134.68, 132.31, 128.96, 128.03, 114.84, 114.74, 65.55, 55.61, 31.91, 14.73; HRMS *m/z* (ESI) calcd for $C_{18}H_{20}NO_2$ $(M + H)^+$, 282.1489, found 282.1492. IR Qmax/cm-1 (film): 521, 540, 701, 732, 822, 1039, 1179, 1237, 1447, 1512, 1674, 2969.

(3-((4-Methoxyphenyl)amino)oxetan-3-yl)(phenyl)methanone (6ag)

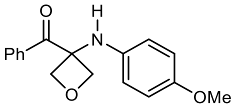

According to general procedure B, the reaction of 4-methoxyaniline (12.3 mg, 0.1 mmol), oxetan-3-one (14.4 mg, 0.2 mmol), diethyl 4-benzoyl-2,6-dimethyl-1,4-dihydropyridine-3,5-dicarboxylate (44 mg, 0.12 mmol), 4 Å molecular sieve (200 mg), $BF_3.Et_2O$ (19 uL, 0.15 mmol) in DCM (2 mL). The crude reaction was purified by flash column chromatography (PE/Acetone = 3:1) to provide product as yellow oil (17 mg, 60%). **^{1}H NMR (400 MHz, $CDCl_3$)**: δ 7.91 (dd, *J* = 8.2, 1.5 Hz, 2H), 7.60 – 7.50 (m, 1H), 7.42 (t, *J* = 7.7 Hz, 2H), 6.70 (d, *J* = 8.9 Hz, 2H), 6.42 (d, *J* = 8.9 Hz, 2H), 5.36 (d, *J* = 6.7 Hz, 2H), 4.81 (d, *J* = 6.7 Hz, 2H), 4.49 (brs, 1H), 3.71 (s, 3H); **^{13}C NMR (101 MHz, $CDCl_3$)**: δ 198.50, 153.31, 137.92, 133.43, 133.30, 128.90, 128.50, 115.52, 114.90, 78.46, 65.20, 55.55; HRMS *m/z* (ESI) calcd for $C_{17}H_{18}NO_3$ $(M + H)^+$, 284.1287, found 284.1284. IR Qmax/cm-1 (film): 707, 821, 988, 1035, 1179, 1239, 1447, 1512, 1679, 2875, 2950.

cyclopropyl(1-((4-methoxyphenyl)amino)cyclopentyl)methanone (6ah)

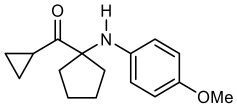

According to general procedure B, the reaction of 4-methoxyaniline (12.3 mg, 0.1 mmol), cyclopentanone (16.8 mg, 0.2 mmol), diethyl 4-(cyclopropanecarbonyl)-2,6-dimethyl-1,4-dihydropyridine-3,5-dicarboxylate (39 mg, 0.12 mmol), 4 Å molecular

sieve (200 mg), $BF_3.Et_2O$ (19 uL, 0.15 mmol) in DCM (2 mL). The crude reaction was purified by flash column chromatography (PE/DCM/EA = 16:3:1) to provide product as yellow oil (10.4 mg, 40%). **^{1}H NMR (400 MHz, $CDCl_3$)**: δ 6.74 (d, *J* = 8.9 Hz, 2H), 6.43 (d, *J* = 8.9 Hz, 2H), 3.97 (brs, 1H), 3.75 (s, 3H), 2.59-2.53 (m, 1H), 2.44 – 2.24 (m, 2H), 1.91 – 1.71 (m, 6H), 0.96 (p, *J* = 3.8 Hz, 2H), 0.82-0.77 (m, 2H); **^{13}C NMR (101 MHz, $CDCl_3$)**: δ 215.06, 152.19, 139.98, 115.12, 114.66, 73.36, 55.67, 36.29, 24.83, 15.83, 11.51; HRMS *m/z* (ESI) calcd for $C_{16}H_{22}NO_2$ $(M + H)^+$, 260.1651, found 260.1647. IR Qmax/cm-1 (film): 819, 1037, 1237, 1373, 1511, 1688, 2950, 3391.

(1-((4-methoxyphenyl)amino)cyclohexyl)(phenyl)methanone (6ai)

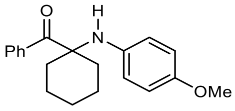

According to general procedure B, the reaction of 4-methoxyaniline (12.3 mg, 0.1 mmol), cyclohexanone (21 uL, 0.2 mmol), diethyl 4-benzoyl-2,6-dimethyl-1,4-dihydropyridine-3,5-dicarboxylate (44 mg, 0.12 mmol), 4 Å molecular sieve (200 mg), $BF_3.Et_2O$ (19 uL, 0.15 mmol) in DCM (2 mL). The crude reaction was purified by flash column chromatography (PE/DCM/EA = 16:3:1) to provide product as pale yellow oil (12 mg, 40%). **^{1}H NMR (400 MHz, $CDCl_3$)**: δ 8.28 – 8.19 (m, 2H), 7.52 – 7.43 (m, 1H), 7.36 (dd, J = 8.4, 7.0 Hz, 2H), 6.70 (d, J = 8.9 Hz, 2H), 6.55 (d, J = 8.9 Hz, 2H), 3.97 (s, 1H), 3.71 (s, 3H), 2.08 – 1.94 (m, 4H), 1.66 – 1.51 (m, 5H), 1.45 – 1.32 (m, 1H); **^{13}C NMR (101 MHz, $CDCl_3$)**: δ 206.18, 152.60, 138.82, 136.93, 131.95, 129.34, 128.06, 116.67, 114.67, 64.48, 55.54, 32.80, 25.33, 21.35; HRMS *m/z* (ESI) calcd for $C_{20}H_{24}NO_2$ $(M + H)^+$, 310.1807, found 310.1806. IR Qmax/cm-1 (film): 698, 819, 1038, 1178, 1237, 1448, 1510, 1669, 2933.

Cyclopropyl(4,4-difluoro-1-((4-methoxyphenyl)amino)cyclohexyl)methanone (6aj)

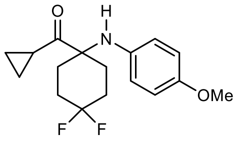

According to general procedure B, the reaction of 4-methoxyaniline (12.3 mg, 0.1 mmol), 4,4-difluorocyclohexan-1-one (26.8 mg, 0.2 mmol), diethyl 4-(cyclopropanecarbonyl)-2,6-dimethyl-1,4-dihydropyridine-3,5-dicarboxylate (39 mg, 0.12 mmol), 4 Å molecular sieve (200 mg), $BF_3.Et_2O$ (19 uL, 0.15 mmol) in DCM (2 mL). The crude reaction was purified by flash column chromatography (PE/Acetone = 10:1) to provide product as yellow oil (13 mg, 42%). **^{1}H NMR (400 MHz, $CDCl_3$)**: δ 6.76 (d, *J* = 9.0 Hz, 2H), 6.53 (d, *J* = 8.9 Hz, 2H), 3.78 (s, 1H), 3.76 (s, 3H), 2.62-2.57 (m, 1H), 2.22-2.17 (m, 2H), 2.08 – 1.97 (m, 6H), 1.07 – 0.99 (m, 2H), 0.90-0.83 (m, 2H); **^{13}C NMR (101 MHz, $CDCl_3$)**: δ 199.90, 141.67, 137.36, 135.06, 134.58, 132.99, 128.74, 128.55, 128.52, 128.49, 128.41, 126.45, 125.98, 125.94, 125.48, 66.65, 52.06, 46.97, 32.64, 30.02, 27.75; **^{13}F NMR (376 MHz, $CDCl_3$)**: δ -95.85, -96.48, -100.83, -101.46; HRMS *m/z* (ESI) calcd for $C_{17}H_{22}FNO_2$ $(M + H)^+$, 310.1619, found 310.1617. IR Qmax/cm-1 (film): 822, 957, 1033, 1085, 1113, 1238, 1269, 1375, 1442, 1512, 1691, 2942, 3401.

1-cyclopropyl-2-((4-methoxyphenyl)amino)-2-methylpropan-1-one (6ak)

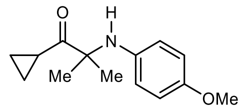

According to general procedure B, the reaction of 4-methoxyaniline (12.3 mg, 0.1 mmol), acetone (23 mg, 0.4 mmol), diethyl 4-(cyclopropanecarbonyl)-2,6-dimethyl-1,4-dihydropyridine-3,5-dicarboxylate (39 mg, 0.12 mmol), 4 Å molecular sieve (200 mg), $BF_3.Et_2O$ (19 uL, 0.15 mmol) in DCM (2 mL). The crude reaction was purified by flash column chromatography (PE/Acetone = 10:1) to provide product as yellow oil (14 mg, 60%). **^{1}H NMR (400 MHz, $CDCl_3$)**: δ 6.75 (d, *J* = 8.9 Hz, 2H), 6.50 (d, *J* = 8.9 Hz, 2H), 3.76 (s, 3H), 2.70 – 2.60 (m, 1H), 1.46 (s, 6H), 1.04-1.00 (m, 2H), 0.88-0.85 (m, 2H); **^{13}C NMR (101 MHz, $CDCl_3$)**: δ 215.58, 152.64, 139.33, 116.45, 114.62, 62.56, 55.65, 24.92, 15.20, 11.89; HRMS *m/z* (ESI) calcd for $C_{14}H_{20}NO_2$ $(M + H)^+$, 234.1494, found 234.1494. IR Qmax/cm-1 (film): 820, 1038, 1056, 1180, 1238, 1379, 1441, 1464, 1511, 1692, 2933, 2986, 3383.

1-cyclopropyl-2-((4-methoxyphenyl)amino)-2-methylheptan-1-one (6al)

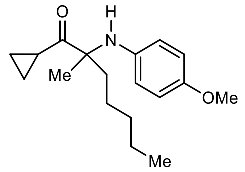

According to general procedure B, the reaction of 4-methoxyaniline (12.3 mg, 0.1 mmol), heptan-2-one (23 mg, 0.2 mmol), diethyl 4-(cyclopropanecarbonyl)-2,6-dimethyl-1,4-dihydropyridine-3,5-dicarboxylate (39 mg, 0.12 mmol), 4 Å molecular sieve (200 mg), $BF_3.Et_2O$ (19 uL, 0.15 mmol) in DCM (2 mL). The crude reaction was purified by flash column chromatography (PE/EA = 10:1) to provide product as yellow oil (15.9 mg, 55%). **^{1}H NMR (400 MHz, $CDCl_3$)**: δ 6.77 – 6.72 (m, 2H), 6.51 (d, *J* = 8.4 Hz, 2H), 3.95 (brs, 1H), 3.75 (s, 3H), 2.60 (tt, *J* = 8.0, 4.6 Hz, 1H), 1.99 – 1.71 (m, 2H), 1.40 (s, 3H), 1.27 (tt, *J* = 9.2, 5.6 Hz, 6H), 1.08-0.96 (m, 2H), 0.94 – 0.77 (m, 5H); **^{13}C NMR (101 MHz, $CDCl_3$)**: δ 215.11, 152.40, 139.46, 116.39, 114.58, 65.39, 55.62, 37.85, 32.00, 23.13, 22.37, 21.27, 15.56, 13.90, 11.80, 11.67; HRMS *m/z* (ESI) calcd for $C_{18}H_{28}NO_2$ $(M + H)^+$, 290.2114, found 290.2120. IR Qmax/cm-1 (film): 819, 1014, 1041, 1178, 1237, 1373, 1463, 1511, 1690, 2860, 2933, 3384.

Tert-butyl 2-benzoyl-2-((4-methoxyphenyl)amino)-7-azaspiro[3.5]nonane-7-carboxylate (6am)

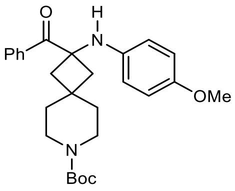

According to general procedure B, the reaction of 4-methoxyaniline (12.3 mg, 0.1 mmol), tert-butyl 2-oxo-7-azaspiro[3.5]nonane-7-carboxylate (29 mg, 0.12 mmol),

diethyl 4-benzoyl-2,6-dimethyl-1,4-dihydropyridine-3,5-dicarboxylate (44 mg, 0.12 mmol), 4 Å molecular sieve (200 mg), $BF_3.Et_2O$ (19 uL, 0.15 mmol) in DCM (2 mL). The crude reaction was purified by flash column chromatography (PE/Acetone = 5:1) to provide product as yellow solid (32 mg, 71%). Melting point: 180-200 °C. **^{1}H NMR (400 MHz, $CDCl_3$)**: δ 7.91 (d, *J* = 7.3 Hz, 2H), 7.49 – 7.44 (m, 1H), 7.36 (t, *J* = 7.6 Hz, 2H), 6.66 (d, *J* = 8.9 Hz, 2H), 6.36 (d, *J* = 8.9 Hz, 2H), 4.20 (brs, 1H), 3.69 (s, 3H), 3.41 – 3.33 (m, 4H), 2.90 (d, *J* = 12.7 Hz, 2H), 2.22 – 2.04 (m, 2H), 1.72 (t, *J* = 5.6 Hz, 2H), 1.55 (t, *J* = 5.7 Hz, 2H), 1.47 (s, 9H); **^{13}C NMR (101 MHz, $CDCl_3$)**: δ 202.94, 154.87, 152.45, 138.81, 134.39, 132.40, 128.80, 128.11, 114.74, 114.63, 79.37, 59.76, 55.58, 41.27, 38.99, 37.30, 31.58, 28.41; HRMS *m/z* (ESI) calcd for $C_{27}H_{35}N_2O_4$ $(M + H)^+$, 451.2597, found 451.2595. IR Qmax/cm-1 (film): 712, 732, 821, 969, 1038, 1151, 1176, 1242, 1425, 1512, 1670, 2929, 2975, 3369.

Benzyl 4-((1-(3-chlorophenyl)-1-oxo-4-phenylbutan-2-yl)amino)piperidine-1-carboxylate (6an)

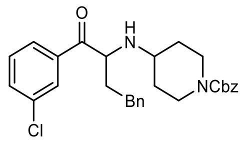

According to general procedure A, the reaction of benzyl 4-aminopiperidine-1-carboxylate (23.4 mg, 0.1 mmol), hydrocinnamaldehyde (16 uL, 0.12 mmol), diethyl 4-(3-chlorobenzoyl)-2,6-dimethyl-1,4-dihydropyridine-3,5-dicarboxylate (47 mg, 0.12 mmol), 4 Å molecular sieve (200 mg), $BF_3.Et_2O$ (19 uL, 0.15 mmol) in DCM (2 mL). The crude reaction was purified by flash column chromatography (PE/Acetone = 8:1) to provide product as yellow oil (25 mg, 50%). **^{1}H NMR (400 MHz, $CDCl_3$)**: δ 7.71 (s, 1H), 7.61 – 7.51 (m, 2H), 7.44 – 7.30 (m, 8H), 7.27-7.16 (m, 3H), 5.15 (s, 2H), 4.16 – 4.01 (m, 3H), 2.99-2.79 (m, 4H), 2.53-2.45 (m, 1H), 2.21 (brs, 1H), 2.03-1.94 (m, 1H), 1.82 – 1.60 (m, 3H), 1.49 – 1.29 (m, 2H); **^{13}C NMR (101 MHz, $CDCl_3$)**: δ 202.30, 155.21, 141.02, 136.85, 136.78, 135.13, 133.37, 130.07, 128.75, 128.57, 128.46, 128.28, 127.94, 127.84, 126.29, 126.21, 67.03, 58.52, 53.30, 42.46, 42.29, 35.63, 32.02; HRMS *m/z* (ESI) calcd for $C_{29}H_{32}ClN_2O_3$ $(M + H)^+$, 491.2101, found 491.2100. IR Qmax/cm-1 (film): 698, 733, 752, 1230, 1275, 1368, 1430, 1472, 1496, 1560, 1697, 2860, 2931, 3028.

Benzyl 4-((1-(benzo[d][1,3]dioxol-5-yl)-1-*oxo*-4-phenylbutan-2-yl)amino)piperidine-1-carboxylate (6ao)

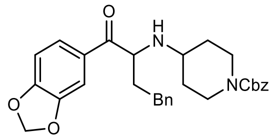

According to general procedure A, the reaction of benzyl 4-aminopiperidine-1-carboxylate (23.4 mg, 0.1 mmol), hydrocinnamaldehyde (16 uL, 0.12 mmol), diethyl 4-(benzo[d][1,3]dioxole-5-carbonyl)-2,6-dimethyl-1,4-dihydropyridine-3,5-dicarboxyla-te (48 mg, 0.12 mmol), 4 Å molecular sieve (200 mg), $BF_3.Et_2O$ (19 uL, 0.15 mmol) in DCM (2 mL). The crude reaction was purified by flash column chromatography

(PE/Acetone = 5:1) to provide product as yellow oil (35 mg, 70%). **^{1}H NMR (400 MHz, $CDCl_3$)**: δ 7.43 – 7.19 (m, 12H), 6.78 (d, *J* = 8.0 Hz, 1H), 6.05 (s, 2H), 5.15 (s, 2H), 4.18 – 3.92 (m, 3H), 2.94-2.77 (m, 4H), 2.52-2.45 (m, 1H), 2.20 (brs, 1H), 2.02-1.93 (m, 1H), 1.85 – 1.63 (m, 3H), 1.54 – 1.32 (m, 2H); **^{13}C NMR (101 MHz, $CDCl_3$)**: δ 201.53, 155.17, 152.02, 148.29, 141.32, 136.85, 130.07, 128.72, 128.41, 127.88, 127.78, 126.10, 124.39, 107.94, 107.90, 101.86, 66.95, 58.03, 53.19, 42.45, 42.27, 36.10, 32.13; HRMS *m/z* (ESI) calcd for $C_{30}H_{33}N_2O_5$ $(M + H)^+$, 501.2389, found 501.2390. IR Qmax/cm-1 (film): 698, 751, 932, 1036, 1095, 1133, 1228, 1250, 1363, 1437, 1487, 1602, 1694, 2855, 2936.

4-Phenyl-2-(piperidin-1-yl)-1-(thiophen-2-yl)butan-1-one (6ap)

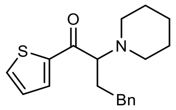

According to general procedure A, the reaction of piperidine (10 uL, 0.1 mmol), hydrocinnamaldehyde (16 uL, 0.12 mmol), diethyl 2,6-dimethyl-4-(thiophene-2-carbonyl)-1,4-dihydropyridine-3,5-dicarboxylate (44 mg, 0.12 mmol), 4 Å molecular sieve (200 mg), TBSOTf (28 uL, 0.12 mmol) in DCM (2 mL). The crude reaction was purified by flash column chromatography (PE/Et_2O = 10:1) to provide product as yellow oil (22 mg, 69%). **^{1}H NMR (400 MHz, $CDCl_3$)**: δ 7.86 (d, *J* = 3.8 Hz, 1H), 7.61 (d, *J* = 4.9 Hz, 1H), 7.34 – 7.24 (m, 2H), 7.24 – 7.17 (m, 3H), 7.12 (t, *J* = 4.4 Hz, 1H), 3.73 (dd, *J* = 8.9, 4.8 Hz, 1H), 2.76-2.68 (m, 1H), 2.64-2.53 (m, 5H), 2.26 – 2.11 (m, 1H), 2.07-1.98 (m, 1H), 1.62-1.56 (m, 4H), 1.47-1.41 (m, 2H); **^{13}C NMR (101 MHz, $CDCl_3$)**: δ 193.57, 143.04, 141.87, 133.67, 132.76, 128.43, 128.31, 127.47, 125.84, 70.52, 51.03, 32.90, 28.12, 26.26, 24.43; HRMS *m/z* (ESI) calcd for $C_{19}H_{24}NOS$ $(M + H)^+$, 314.1579, found 314,1575. IR Qmax/cm-1 (film): 699, 724, 858, 1036, 1113, 1205, 1234, 1353, 1411, 1441, 1452, 1495, 1507, 1661, 2804, 2851, 2931.

1-(Furan-2-yl)-4-phenyl-2-(piperidin-1-yl)butan-1-ol (6aq)

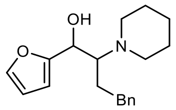

According to general procedure A, the reaction of piperidine (10 uL, 0.1 mmol), hydrocinnamaldehyde (16 uL, 0.12 mmol), diethyl 4-(furan-2-carbonyl)-2,6-dimethyl-1,4-dihydropyridine-3,5-dicarboxylate (42 mg, 0.12 mmol), 4 Å molecular sieve (200 mg), TBSOTf (28 uL, 0.12 mmol) in DCM (2 mL). After reaction, the product was reduced by $NaBH_4$ (7.6 mg, 0.2 mmol) in MeOH (2 mL). The crude reaction was purified by flash column chromatography (PE/Acetone = 5:1) to provide product as yellow oil (22 mg, 74%, dr = 10:1). **^{1}H NMR (400 MHz, $CDCl_3$)**: δ 7.46 – 7.38 (m, 1H), 7.28-7.15 (m, 3H), 7.04 (d, *J* = 7.4 Hz, 2H), 6.40 – 6.31 (m, 2H), 4.96 (d, *J* = 4.8 Hz, 0.12H), 4.35 (d, *J* = 9.9 Hz, 1H), 2.90-2.84 (m, 1H), 2.79-2.68 (m, 2H), 2.60-2.46 (m, 2H), 2.34 – 2.26 (m, 1H), 2.21.2.13 (m, 1H), 1.99 – 1.85 (m, 1H), 1.71 – 1.36 (m, 8H).; **^{13}C NMR (101 MHz, $CDCl_3$)**: δ 154.19, 142.14, 141.83, 141.29, 128.42, 128.33, 128.28, 128.19, 125.98, 125.84, 110.38, 108.85, 106.53, 68.17, 67.59, 66.64, 66.26, 33.91, 33.45, 28.02, 27.45,

26.64, 26.09, 24.59, 24.14; HRMS *m/z* (ESI) calcd for $C_{19}H_{26}NO_2$ $(M + H)^+$, 300.1964, found 300.1964. IR Qmax/cm-1 (film): 699, 734, 1010, 1031, 1061, 1110, 1151, 1375, 1441, 1453, 1495, 2852, 2932.

1,6-Diphenyl-4-(piperidin-1-yl)hexan-3-one (6ar)

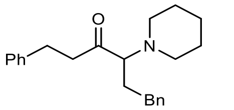

According to general procedure A, the reaction of piperidine (10 uL, 0.1 mmol), hydrocinnamaldehyde (16 uL, 0.12 mmol), diethyl 2,6-dimethyl-4-(3-phenylpropanoyl)-1,4-dihydropyridine-3,5-dicarboxylate (46 mg, 0.12 mmol), 4 Å molecular sieve (200 mg), TBSOTf (28 uL, 0.12 mmol) in DCM (2 mL). The crude reaction was purified by flash column chromatography (PE/Acetone = 10:1) to provide product as yellow oil (23.4 mg, 70%). **^{1}H NMR (400 MHz, CDCl$_3$)**: δ 7.31 – 7.27 (m, 4H), 7.25 – 7.18 (m, 4H), 7.17 – 7.14 (m, 2H), 3.02 (dd, *J* = 8.9, 4.8 Hz, 1H), 3.00 – 2.90 (m, 3H), 2.83 – 2.76 (m, 1H), 2.60 (ddd, *J* = 13.7, 9.9, 5.8 Hz, 1H), 2.48 – 2.38 (m, 5H), 2.00-1.92 (m, 1H), 1.87 – 1.79 (m, 1H), 1.57-1.48 (m, 4H), 1.44-1.38 (m, 2H); **^{13}C NMR (101 MHz, CDCl$_3$)**: δ 210.94, 141.91, 141.44, 128.43, 128.39, 128.33, 125.97, 125.85, 72.98, 50.98, 42.67, 32.90, 29.79, 26.59, 26.55, 24.45; HRMS *m/z* (ESI) calcd for $C_{23}H_{30}NO$ $(M + H)^+$, 336.2327, found 336.2325. IR Qmax/cm-1 (film): 698, 747, 1029, 1065, 1094, 1263, 1453, 1495, 1712, 2932.

1-Cyclopropyl-4-phenyl-2-(piperidin-1-yl)butan-1-one (6as)

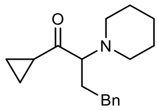

According to general procedure A, the reaction of piperidine (10 uL, 0.1 mmol), hydrocinnamaldehyde (16 uL, 0.12 mmol), diethyl 4-(cyclopropanecarbonyl)-2,6-dimethyl-1,4-dihydropyridine-3,5-dicarboxylate (39 mg, 0.12 mmol), 4 Å molecular sieve (200 mg), TBSOTf (28 uL, 0.12 mmol) in DCM (2 mL). The crude reaction was purified by flash column chromatography (PE/EA = 10:1) to provide product as yellow oil (20 mg, 73%). **^{1}H NMR (400 MHz, CDCl$_3$)**: δ 7.33 – 7.27 (m, 2H), 7.24 – 7.17 (m, 3H), 3.16 (dd, *J* = 8.5, 5.3 Hz, 1H), 2.69-2.63 (m, 1H), 2.61 – 2.48 (m, 5H), 2.35-2.30 (m, 1H), 2.08 – 1.90 (m, 2H), 1.62-1.57 (m, 4H), 1.50 – 1.42 (m, 2H), 1.13 – 1.02 (m, 2H), 0.94 – 0.87 (m, 2H); **^{13}C NMR (101 MHz, CDCl$_3$)**: δ 211.98, 142.08, 128.42, 128.32, 125.81, 74.20, 51.30, 32.68, 28.46, 26.55, 24.57, 19.11, 11.06, 11.03; HRMS *m/z* (ESI) calcd for $C_{18}H_{26}NO$ $(M + H)^+$, 272.2014, found 272.2009. IR Qmax/cm-1 (film): 699, 734, 750, 915, 1000, 1031, 1064, 1114, 1378, 1441, 1453, 1495, 1697, 2932.

1-cyclohexyl-4-phenyl-2-(piperidin-1-yl)butan-1-one (6at)

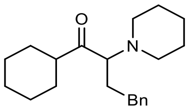

According to general procedure A, the reaction of piperidine (10 uL, 0.1 mmol),

hydrocinnamaldehyde (16 uL, 0.12 mmol), diethyl 4-(cyclohexanecarbonyl)-2,6-dimethyl-1,4-dihydropyridine-3,5-dicarboxylate (44 mg, 0.12 mmol), 4 Å molecular sieve (200 mg), TBSOTf (28 uL, 0.12 mmol) in DCM (2 mL). The crude reaction was purified by flash column chromatography (PE/EA = 10:1) to provide product as yellow oil (26 mg, 80%). **^{1}H NMR (400 MHz, CDCl$_3$)**: δ 7.33 – 7.28 (m, 2H), 7.22 – 7.17 (m, 3H), 3.22 (dd, *J* = 8.9, 4.6 Hz, 1H), 2.66 (dddd, *J* = 28.9, 15.6, 8.1, 4.4 Hz, 2H), 2.55 – 2.39 (m, 5H), 2.09 – 1.94 (m, 1H), 1.88 – 1.66 (m, 6H), 1.57-1.52 (m, 4H), 1.47 – 1.41 (m, 3H), 1.38 – 1.21 (m, 4H); **^{13}C NMR (101 MHz, CDCl$_3$)**: δ 213.95, 142.11, 128.38, 128.30, 125.77, 70.86, 50.82, 48.76, 32.99, 29.18, 28.02, 26.62, 26.00, 25.90, 25.71, 25.51, 24.52; HRMS *m/z* (ESI) calcd for $C_{21}H_{32}NO$ (M + H)$^+$, 314.2484, found 314.2487. IR Qmax/cm-1 (film): 698, 749, 1450, 1707, 2852, 2928.

1-((3r,5r,7r)-adamantan-1-yl)-4-phenyl-2-(piperidin-1-yl)butan-1-one (6au)

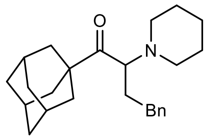

According to general procedure A, the reaction of piperidine (10 uL, 0.1 mmol), hydrocinnamaldehyde (16 uL, 0.12 mmol), diethyl 4-((3R,5R)-adamantane-1-carbonyl)-2,6-dimethyl-1,4-dihydropyridine-3,5-dicarboxylate (50 mg, 0.12 mmol), 4 Å molecular sieve (200 mg), TBSOTf (28 uL, 0.12 mmol) in DCM (2 mL). The crude reaction was purified by flash column chromatography (PE/EA = 10:1) to provide product as yellow oil (30 mg, 82%). **^{1}H NMR (400 MHz, CDCl$_3$)**: δ 7.33 – 7.26 (m, 2H), 7.23 – 7.14 (m, 3H), 3.53 (dd, *J* = 9.3, 4.5 Hz, 1H), 2.60-2.48 (m, 3H), 2.47 – 2.29 (m, 3H), 2.09 – 1.95 (m, 4H), 1.94-1.82 (m, 6H), 1.79-1.68 (m, 7H), 1.56 – 1.47 (m, 4H), 1.40 (p, *J* = 5.7 Hz, 2H); **^{13}C NMR (101 MHz, CDCl$_3$)**: δ 213.06, 142.15, 128.35, 128.27, 125.74, 65.79, 50.21, 45.92, 38.53, 36.65, 32.94, 28.12, 26.65, 24.90, 24.67; HRMS *m/z* (ESI) calcd for $C_{25}H_{36}NO$ (M + H)$^+$, 366.2797, found 366.2796. IR Qmax/cm-1 (film): 698, 749, 990, 1107, 1451, 1693, 2848, 2904, 2927.

2-(Pyrrolidin-1-yl)-1-(p-tolyl)pentan-1-one (7a)

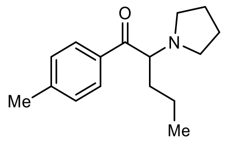

According to general procedure A, the reaction of pyrrolidine (7.1 mg, 0.1 mmol), butyraldehyde (8.7 mg, 0.12 mmol), diethyl 2,6-dimethyl-4-(4-methylbenzoyl)-1,4-dihydropyridine-3,5-dicarboxylate (45 mg, 0.12 mmol), 4 Å molecular sieve (200 mg), TBSOTf (28 uL, 0.12 mmol) in DCM (2 mL). The crude reaction was purified by flash column chromatography (PE/Acetone = 5:1) to provide product as yellow oil (16 mg, 65%). **^{1}H NMR (400 MHz, CDCl$_3$)**: δ 8.04 (d, *J* = 8.3 Hz, 2H), 7.27 (d, *J* = 7.9 Hz, 2H), 3.96 (dd, *J* = 8.9, 4.6 Hz, 1H), 2.76 – 2.70 (m, 2H), 2.63-2.58 (m, 2H), 2.43 (s, 3H), 1.98 – 1.87 (m, 1H), 1.81 – 1.72 (m, 4H), 1.34 – 1.21 (m, 3H), 0.88 (t, *J* = 7.4 Hz, 3H); **^{13}C NMR (101 MHz, CDCl$_3$)**: δ 200.75, 143.77, 134.61, 129.19, 128.73, 68.44, 51.06, 33.12, 23.38,

21.62, 19.33, 14.27; HRMS *m/z* (ESI) calcd for $C_{16}H_{24}NO$ (M + H)$^+$, 246.1858, found 246.1858. IR Qmax/cm-1 (film): 1180, 1204, 1249, 1290, 1378, 1457, 1606, 1675, 2873, 2931, 2959.

1-cyclopropyl-2-(6,7-dihydrothieno[3,2-c]pyridin-5(4H)-yl)-2-(2-fluorophenyl)ethan-1-one (7b)

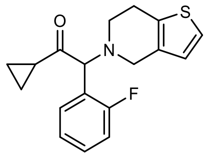

According to general procedure A, the reaction of 4,5,6,7-tetrahydrothieno[3,2-c]pyridine (14 mg, 0.1 mmol), 2-fluorobenzaldehyde (15 mg, 0.12 mmol), diethyl 4-(cyclopropanecarbonyl)-2,6-dimethyl-1,4-dihydropyridine-3,5-dicarboxylate (39 mg, 0.12 mmol), 4 Å molecular sieve (200 mg), $BF_3.Et_2O$ (19 uL, 0.15 mmol) in DCM (2 mL). The crude reaction was purified by flash column chromatography (PE/Et_2O = 5:1) to provide product as yellow oil (13 mg, 40%). **^{1}H NMR (400 MHz, $CDCl_3$)**: δ 7.52 (td, *J* = 7.5, 1.8 Hz, 1H), 7.38-7.32 (m, 1H), 7.24 – 7.11 (m, 2H), 7.08 (d, *J* = 5.1 Hz, 1H), 6.70 (d, *J* = 5.1 Hz, 1H), 4.88 (s, 1H), 3.73 – 3.59 (m, 2H), 2.94 (s, 3H), 2.84 – 2.75 (m, 1H), 2.35-2.28 (m, 1H), 1.17 – 1.00 (m, 2H), 0.94 – 0.82 (m, 2H); **^{13}C NMR (101 MHz, $CDCl_3$)**: δ 207.78, 162.34 (d, *J* = 245.6 Hz), 133.47, 133.21, 130.60 (d, *J* = 3.5 Hz), 129.86 (d, *J* =8.3 Hz), 125.25, 124.39 (d, *J* = 3.7 Hz), 122.69, 122.15 (d, *J* =14.1 Hz,), 115.82 (d, *J* = 22.5 Hz), 71.60, 50.85, 48.56, 25.51, 18.31, 12.10, 11.48; **^{19}F NMR (376 MHz, $CDCl_3$)**: δ -117.15; HRMS *m/z* (ESI) calcd for $C_{18}H_{19}FNOS$ (M + H)$^+$, 316.1171, found 316.1170. IR Qmax/cm-1 (film): 703, 759, 903, 1007, 1081, 1225, 1374, 1454, 1486, 1697, 2819, 2841, 2920.

2-(Tert-butylamino)-1-(3-chlorophenyl)-4,4,4-trifluorobutan-1-one (7c)

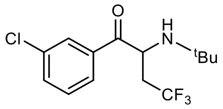

According to general procedure A, the reaction of tert-butylamine (7.3 mg, 0.1 mmol), 3,3,3-trifluoropropanal (33.6 mg, 0.3 mmol), diethyl 4-(3-chlorobenzoyl)-2,6-dimethyl-1,4-dihydropyridine-3,5-dicarboxylate (47 mg, 0.12 mmol), 4 Å molecular sieve (200 mg), $BF_3.Et_2O$ (19 uL, 0.15 mmol) in DCM (2 mL). The crude reaction was purified by flash column chromatography (PE/Et_2O = 5:1) to provide product as yellow oil (12 mg, 38%). **^{1}H NMR (400 MHz, $CDCl_3$)**: δ 8.00 (t, J = 1.9 Hz, 1H), 7.89 (dt, J = 7.8, 1.4 Hz, 1H), 7.63 (dt, J = 8.2, 1.4 Hz, 1H), 7.50 (t, J = 7.9 Hz, 1H), 4.55 (dd, J = 8.1, 4.3 Hz, 1H), 2.46 – 2.18 (m, 2H), 1.86 (s, 1H), 1.05 (s, 9H); **^{13}C NMR (101 MHz, $CDCl_3$)**: δ 201.13, 135.95, 135.56, 133.85, 130.38, 128.53 , 126.34, 125.90 (q, *J* = 276.1 Hz), 77.32, 77.00, 76.68, 51.01, 50.89, 50.86, 39.24 (q, *J* = 27.1 Hz), 29.72; **^{19}F NMR (376 MHz, CDCl3)**: δ -64.10; HRMS *m/z* (ESI) calcd for $C_{14}H_{18}ClF_3NO$ (M + H)$^+$, 308.1024, found 308.1028. IR Qmax/cm-1 (film): 1100, 1151, 1204, 1237, 1257, 1368, 1691, 2961.

2-(diethylamino)-N-(2,6-dimethylphenyl)acetamide-2,2-d2 (7d)

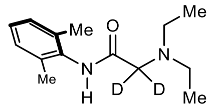

According to general procedure A, the reaction of diethylamine (7.3 mg, 0.1 mmol), CD_2O 20% in D_2O (48 mg, 0.3 mmol), diethyl 4-((2,6-dimethylphenyl)carbamoyl)-2,6-dimethyl-1,4-dihydropyridine-3,5-dicarboxylate (48 mg, 0.12 mmol), 4 Å molecular sieve (200 mg), $BF_3.Et_2O$ (19 uL, 0.15 mmol) in DCM (2 mL). The crude reaction was purified by flash column chromatography (PE/Acetone = 5:1) to provide product as colorless oil (13 mg, 55%). **^{1}H NMR (400 MHz, $CDCl_3$)**: δ 8.95 (s, 1H), 7.09-7.13 (m, 3H), 2.72 (q, J = 7.1 Hz, 4H), 2.26 (s, 6H), 1.16 (t, J = 7.1 Hz, 6H); **^{13}C NMR (101 MHz, $CDCl_3$)**: δ 170.28, 135.05, 133.93, 128.20, 127.04, 48.87, 18.72, 18.55, 12.63; HRMS *m/z* (ESI) calcd for $C_{14}H_{21}D_2N_2O$ $(M + H)^+$, 237.1936, found 237.1937. IR Qmax/cm-1 (film): 767, 1213, 1375, 1494, 1682, 2930, 2968, 3277.

14-azido-N-benzyl-2-phenethyl-6,9,12-trioxa-3-azatetradecanamide (S1)

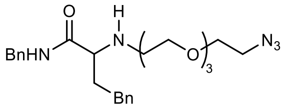

According to general procedure A, the reaction of 2-(2-(2-(2-azidoethoxy)ethoxy)ethoxy)ethan-1-amine (21.8 mg, 0.1 mmol), hydrocinnamaldehyde (16 uL, 0.12 mmol), diethyl 4-(benzylcarbamoyl)-2,6-dimethyl-1,4-dihydropyridine-3,5-dicarboxylate (46 mg, 0.12 mmol), 4 Å molecular sieve (200 mg), $BF_3.Et_2O$ (38 uL, 0.30 mmol) in DCM (2 mL). The crude reaction was purified by flash column chromatography (PE/Acetone = 3:1) to provide product as yellow oil (24 mg, 50%). **^{1}H NMR (400 MHz, $CDCl_3$)**: δ 7.72 (t, J = 6.0 Hz, 1H), 7.37 – 7.29 (m, 7H), 7.23-7.18 (m, 3H), 4.48 (qd, J = 14.9, 6.0 Hz, 2H), 3.66 – 3.46 (m, 13H), 3.36 (t, J = 5.0 Hz, 2H), 3.19 (dd, J = 7.7, 5.0 Hz, 1H), 2.90 – 2.64 (m, 4H), 2.19-2.09 (m, 1H), 1.98 – 1.85 (m, 1H); **^{13}C NMR (101 MHz, $CDCl_3$)**: δ 174.19, 141.23, 138.68, 128.59, 128.42, 128.40, 127.56, 127.27, 126.00, 77.32, 77.00, 76.68, 70.65, 70.60, 70.56, 70.18, 69.98, 62.32, 50.59, 47.93, 42.91, 35.44, 32.27; HRMS *m/z* (ESI) calcd for $C_{25}H_{36}N_5O_4$ $(M + H)^+$, 470.2767, found 470.2764. IR Qmax/cm-1 (film): 700, 749, 1029, 1120, 1248, 1284, 1347, 1453, 1496, 1518, 1656, 2103, 2864, 2917, 3318.

1-Morpholino-2-(piperidin-1-yl)ethan-1-one (S2)

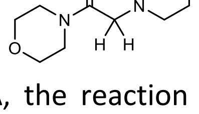

According to general procedure A, the reaction of piperidine (10 mg, 0.1 mmol), formalin 37% in water (25 mg, 0.3 mmol), diethyl 2,6-dimethyl-4-(morpholine-4-carbonyl)-1,4-dihydropyridine-3,5-dicarboxylate (44 mg, 0.12 mmol), 4 Å molecular sieve (200 mg), TBSOTf (28 uL, 0.12 mmol) in DCM (2 mL). The crude reaction was purified by flash column chromatography (DCM/MeOH = 5:1) to provide product as yellow oil (10 mg, 45%). **^{1}H NMR (400 MHz, $CDCl_3$)**: δ 3.69-3.66 (m, 6H), 3.65 – 3.60 (m, 2H), 3.16 (s, 2H), 2.45 (t, J = 5.5 Hz, 4H), 1.59 (p, J = 5.6 Hz, 4H), 1.52 – 1.39 (m, 2H);

^{13}C NMR (101 MHz, $CDCl_3$): δ 168.59, 67.09, 67.01, 62.35, 54.34, 46.24, 42.22, 25.93, 23.86; HRMS *m/z* (ESI) calcd for $C_{11}H_{21}N_2O_2$ $(M + H)^+$, 213.1598, found 213.1588. IR Qmax/cm-1 (film): 989, 1113, 1242, 1272, 1301, 1439, 1459, 1643, 2851, 2931.

N-allyl-4-phenyl-2-(piperidin-1-yl)butanamide (S3)

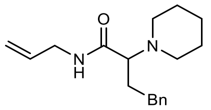

According to general procedure A, the reaction of piperidine (10 mg, 0.1 mmol), hydrocinnamaldehyde (16 uL, 0.12 mmol), diethyl 4-(allylcarbamoyl)-2,6-dimethyl-1,4-dihydropyridine-3,5-dicarboxylate (40 mg, 0.12 mmol), 4 Å molecular sieve (200 mg), TBSOTf (28 uL, 0.12 mmol) in DCM (2 mL). The crude reaction was purified by flash column chromatography (PE/Acetone = 5:1) to provide product as yellow oil (21 mg, 73%). **^1H NMR (400 MHz, $CDCl_3$):** δ 7.35 – 7.26 (m, 3H), 7.25 – 7.15 (m, 3H), 5.89 (ddt, *J* = 17.1, 10.7, 5.5 Hz, 1H), 5.28 – 5.13 (m, 2H), 4.10 – 3.83 (m, 2H), 2.95 (dd, *J* = 8.0, 4.5 Hz, 1H), 2.88 (ddd, *J* = 13.7, 10.4, 5.3 Hz, 1H), 2.69 (ddd, *J* = 13.7, 10.2, 6.6 Hz, 1H), 2.59-2.54 (m, 2H), 2.46-2.41 (m, 2H), 2.15 – 1.90 (m, 2H), 1.59 (tdd, *J* = 11.9, 6.6, 4.6 Hz, 4H), 1.47 (p, *J* = 5.8 Hz, 2H); **^{13}C NMR (101 MHz, $CDCl_3$)**: δ 173.24, 142.09, 134.62, 128.43, 128.31, 125.80, 115.83, 68.55, 51.17, 41.29, 33.60, 28.65, 26.51, 24.26; HRMS *m/z* (ESI) calcd for $C_{18}H_{27}N_2O$ $(M + H)^+$, 287.2123, found 287.2121. IR Qmax/cm-1 (film): 698, 749, 914, 1112, 1261, 1441, 1453, 1496, 1510, 1642, 2806, 2851, 2931, 3306.

Methyl (4-phenyl-2-(piperidin-1-yl)butanoyl)-L-alaninate (S4)

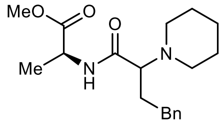

According to general procedure A, the reaction of piperidine (10 mg, 0.1 mmol), hydrocinnamaldehyde (16 uL, 0.12 mmol), diethyl (S)-4-((1-methoxy-1-oxopropan-2-yl)carbamoyl)-2,6-dimethyl-1,4-dihydropyridine-3,5-dicarboxylate (46 mg, 0.12 mmol), 4 Å molecular sieve (200 mg), TBSOTf (28 uL, 0.12 mmol) in DCM (2 mL). The crude reaction was purified by flash column chromatography (PE/Acetone = 3:1) to provide product as yellow oil (27 mg, 81%, 1.2:1 dr). **^1H NMR (400 MHz, $CDCl_3$)**: δ 7.77 (d, *J* = 8.1 Hz, 0.55H), 7.68 (d, *J* = 8.0 Hz, 0.45H), 7.34 – 7.26 (m, 2H), 7.26 – 7.16 (m, 3H), 4.64 (pd, *J* = 7.3, 2.9 Hz, 1H), 3.77 (d, *J* = 5.6 Hz, 3H), 3.02 – 2.77 (m, 2H), 2.74 – 2.38 (m, 5H), 2.17 – 1.86 (m, 2H), 1.67-1.55 (m, 4H), 1.51-1.44 (m, 5H); **^{13}C NMR (101 MHz, $CDCl_3$)**: δ 173.54, 173.51, 173.14, 173.12, 142.15, 141.98, 128.46, 128.39, 128.33, 128.29, 125.83, 125.77, 68.45, 68.03, 52.32, 52.27, 51.28, 50.91, 47.44, 47.40, 33.75, 33.14, 29.15, 27.92, 26.50, 26.45, 24.31, 24.24, 18.60, 18.38; HRMS *m/z* (ESI) calcd for $C_{19}H_{29}N_2O_3$ $(M + H)^+$, 333.2178, found 333.2182. IR Qmax/cm-1 (film): 699, 750, 1110, 1155, 1208, 1452, 1496, 1671, 1743, 2809, 2850, 2932, 3325.

Methyl (4-phenyl-2-(piperidin-1-yl)butanoyl)-L-phenylalaninate (S5)

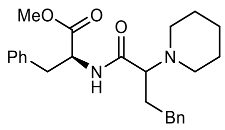

According to general procedure A, the reaction of piperidine (10 mg, 0.1 mmol), hydrocinnamaldehyde (16 uL, 0.12 mmol), diethyl (S)-4-((1-methoxy-1-oxo-3-phenylpropan-2-yl)carbamoyl)-2,6-dimethyl-1,4-dihydropyridine-3,5-dicarboxylate (55 mg, 0.12 mmol), 4 Å molecular sieve (200 mg), TBSOTf (28 uL, 0.12 mmol) in DCM (2 mL). The crude reaction was purified by flash column chromatography (PE/Acetone = 4:1) to provide product as yellow oil (21 mg, 51%, 1.2:1 dr). **^{1}H NMR (400 MHz, $CDCl_3$)**: δ 7.71 (d, J = 8.5 Hz, 0.55H), 7.62 (d, J = 8.2 Hz, 0.45H), 7.34 – 7.24 (m, 5H), 7.23 – 7.15 (m, 5H), 5.00 – 4.87 (m, 1H), 3.76 (d, J = 9.8 Hz, 3H), 3.29 – 3.02 (m, 2H), 2.96 – 2.46 (m, 4H), 2.42 – 2.26 (m, 3H), 2.09 – 1.83 (m, 2H), 1.52 – 1.36 (m, 6H); **^{13}C NMR (101 MHz, $CDCl_3$)**: δ 173.42, 172.30, 142.10, 142.00, 136.09, 136.06, 129.12, 129.07, 128.59, 128.56, 128.44, 128.40, 128.28, 128.27, 127.03, 125.78, 125.76, 68.39, 68.22, 52.50, 52.39, 52.24, 52.17, 51.13, 51.03, 38.01, 37.85, 33.49, 33.34, 28.76, 28.36, 26.40, 24.22, 24.19; HRMS *m/z* (ESI) calcd for $C_{25}H_{33}N_2O_3$ $(M + H)^+$, 409.2491, found 409.2489. IR Qmax/cm-1 (film): 699, 746, 1108, 1176, 1202, 1276, 1441, 1453, 1495, 1675, 1744, 2851, 2932, 3329.

1,4-diphenyl-2-(1,4-dioxa-8-azaspiro[4.5]decan-8-yl)butan-1-one (S6)

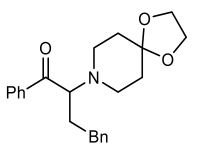

According to general procedure A, the reaction of 1,4-dioxa-8-azaspiro[4.5]decane (14.3 mg, 0.1 mmol), hydrocinnamaldehyde (16 uL, 0.12 mmol), diethyl 4-benzoyl-2,6-dimethyl-1,4-dihydropyridine-3,5-dicarboxylate (44 mg, 0.12 mmol), 4 Å molecular sieve (200 mg), TBSOTf (28 uL, 0.12 mmol) in DCM (2 mL). The crude reaction was purified by flash column chromatography (PE/EA = 2:1) to provide product as colorless oil (24.5 mg, 66%). **^{1}H NMR (400 MHz, $CDCl_3$)**: δ 8.00 (d, *J* = 8.0 Hz, 2H), 7.61 – 7.53 (m, 1H), 7.51 – 7.43 (m, 2H), 7.29 (t, *J* = 7.3 Hz, 2H), 7.20 (dd, *J* = 11.8, 7.1 Hz, 3H), 4.11 (dd, *J* = 9.0, 4.9 Hz, 1H), 3.93 (s, 4H), 2.70 (h, *J* = 6.0 Hz, 5H), 2.53 (ddd, *J* = 14.2, 9.0, 6.6 Hz, 1H), 2.31 – 2.19 (m, 1H), 2.03 (ddt, *J* = 14.0, 10.2, 5.8 Hz, 1H), 1.68 (t, *J* = 5.6 Hz, 4H); **^{13}C NMR (101 MHz, $CDCl_3$)**: δ 199.50, 141.73, 137.30, 132.85, 128.56, 128.42, 128.39, 128.33, 125.88, 107.11, 66.67, 64.13, 47.48, 35.44, 32.69, 27.41; HRMS *m/z* (ESI) calcd for $C_{23}H_{28}NO_3$ $(M + H)^+$, 366.2064, found 366.2069. IR Qmax/cm-1 (film): 701, 911, 946, 1038, 1077, 1093, 1144, 1210, 1228, 1681, 2882, 2954.

2-(1,1-dioxidothiomorpholino)-1,4-diphenylbutan-1-one (S7)

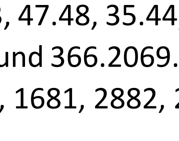

According to general procedure C, the reaction of thiomorpholine 1,1-dioxide

hydrochloride salt (17.2 mg, 0.1 mmol), hydrocinnamaldehyde (16 uL, 0.12 mmol), Et$_3$N (14 uL, 0.1 mmol) diethyl 4-benzoyl-2,6-dimethyl-1,4-dihydropyridine-3,5-dicarboxylate (44 mg, 0.12 mmol), 4 Å molecular sieve (200 mg), TBSOTf (28 uL, 0.12 mmol) in DCM (2 mL). The crude reaction was purified by flash column chromatography (PE/EA = 2:1) to provide product as colorless oil (19 mg, 56%). **^{1}H NMR (400 MHz, CDCl$_3$)**: δ 7.85 (d, *J* = 7.7 Hz, 2H), 7.61 (t, *J* = 7.4 Hz, 1H), 7.47 (t, *J* = 7.6 Hz, 2H), 7.32 (t, *J* = 7.4 Hz, 2H), 7.24 (t, *J* = 7.2 Hz, 1H), 7.18 (d, *J* = 7.4 Hz, 2H), 4.22 (t, *J* = 7.1 Hz, 1H), 3.17 (t, *J* = 4.7 Hz, 4H), 2.99 (t, *J* = 4.4 Hz, 4H), 2.78-2.71 (m, 1H), 2.65-2.57 (m, 1H), 2.31-2.22 (m, 1H), 2.11 – 1.91 (m, 1H); **^{13}C NMR (101 MHz, CDCl$_3$)**: δ 198.86, 140.82, 136.43, 133.48, 128.75, 128.56, 128.40, 128.28, 126.30, 65.92, 52.56, 47.56, 32.40, 28.13; HRMS *m/z* (ESI) calcd for $C_8H_{10}NO$ (M + H)$^+$, 358.1477, found 358.1475. IR Qmax/cm-1 (film): 700, 1124, 1271, 1302, 1680, 2841, 2932.

4-(1-oxo-1,4-diphenylbutan-2-yl)piperazin-2-one (S8)

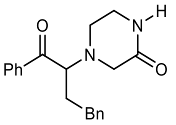

According to general procedure A, the reaction of piperazin-2-one (10 mg, 0.1 mmol), hydrocinnamaldehyde (16 uL, 0.12 mmol), diethyl 4-benzoyl-2,6-dimethyl-1,4-dihydropyridine-3,5-dicarboxylate (44 mg, 0.12 mmol), 4 Å molecular sieve (200 mg), TBSOTf (28 uL, 0.12 mmol) in DCM (2 mL). The crude reaction was purified by flash column chromatography (PE/Acetone = 1:1) to provide product as colorless oil (12 mg, 37%). **^{1}H NMR (400 MHz, CDCl$_3$)**: δ 7.91 – 7.85 (m, 2H), 7.59 (t, *J* = 7.4 Hz, 1H), 7.46 (t, *J* = 7.6 Hz, 2H), 7.31 (t, *J* = 7.9 Hz, 2H), 7.23 (t, *J* = 7.3 Hz, 1H), 7.21 – 7.14 (m, 2H), 6.38 (s, 1H), 4.16 (q, *J* = 6.8 Hz, 1H), 3.50 (d, *J* = 16.5 Hz, 1H), 3.37 (d, *J* = 16.5 Hz, 1H), 3.30-3.27 m, 2H), 2.93 – 2.84 (m, 2H), 2.77 – 2.70 (m, 1H), 2.66 – 2.59 (m, 1H), 2.29 – 2.17 (m, 1H), 2.09 – 2.00 (m, 1H); **^{13}C NMR (101 MHz, CDCl$_3$)**: δ 199.60, 169.48, 141.02, 136.73, 133.37, 128.70, 128.50, 128.46, 128.33, 126.20, 64.51, 53.95, 44.68, 41.90, 32.24, 27.97; HRMS *m/z* (ESI) calcd for $C_{20}H_{23}N_2O_2$ (M + H)$^+$, 323.1760, found 323.1758. IR Qmax/cm-1 (film): 699, 1030, 1156, 1230, 1345, 1447, 1495, 1595, 1671, 2939, 3025.

1,4-diphenyl-2-(4-(trifluoromethyl)piperidin-1-yl)butan-1-one (S9)

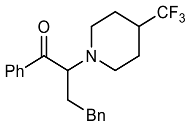

According to general procedure C, the reaction of 4-(trifluoromethyl)piperidine hydrochloride salt (19 mg, 0.1 mmol), hydrocinnamaldehyde (16 uL, 0.12 mmol), Et$_3$N (14 uL, 0.1 mmol), diethyl 4-benzoyl-2,6-dimethyl-1,4-dihydropyridine-3,5-dicarboxylate (44 mg, 0.12 mmol), 4 Å molecular sieve (200 mg), TBSOTf (28 uL, 0.12 mmol) in DCM (2 mL). The crude reaction was purified by flash column chromatography (PE/Et$_2$O = 10:1) to provide product as yellow oil (23 mg, 61%).**^{1}H NMR (400 MHz, CDCl$_3$)**: δ 7.99 – 7.89 (m, 2H), 7.59 (t, *J* = 7.4 Hz, 1H), 7.47 (t, *J* = 7.7

Hz, 2H), 7.38 – 7.27 (m, 2H), 7.26 – 7.17 (m, 3H), 4.12 (dd, J = 8.4, 5.7 Hz, 1H), 3.08 (d, J = 11.9 Hz, 1H), 2.86 (d, J = 11.6 Hz, 1H), 2.74-2.66 (m, 1H), 2.61-2.48 (m, 2H), 2.35 – 2.12 (m, 2H), 2.06 – 1.93 (m, 2H), 1.87 (dt, J = 12.8, 3.0 Hz, 1H), 1.76 (dt, J = 12.8, 3.1 Hz, 1H), 1.62-1.45 (m, 2H); **^{13}C NMR (101 MHz, CDCl$_3$)**: δ 199.79, 141.58, 137.29, 133.04, 128.54, 128.46, 128.41, 126.00, 66.56, 48.82, 48.24, 40.51 (q, J = 27.1 Hz), 32.56, 27.86, 25.36 (q, J = 2.5 Hz), 25.19 (q, J = 2.5 Hz); **^{19}F NMR (376 MHz, CDCl$_3$)**: δ -74.78; HRMS *m/z* (ESI) calcd for $C_{22}H_{25}F_3NO$ (M + H)$^+$, 376.1888, found 376.1891. IR Qmax/cm-1 (film): 695, 750, 1080, 1134, 1152, 1225, 1254, 1282, 1301, 1339, 1449, 1682, 2934, 2953.

2-(3,4-dihydroisoquinolin-2(1H)-yl)-1,4-diphenylbutan-1-one (S10)

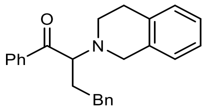

According to general procedure A, the reaction of 1,2,3,4-tetrahydroisoquinoline (13.3 mg, 0.1 mmol), hydrocinnamaldehyde (16 uL, 0.12 mmol), diethyl 4-benzoyl-2,6-dimethyl-1,4-dihydropyridine-3,5-dicarboxylate (44 mg, 0.12 mmol), 4 Å molecular sieve (200 mg), TBSOTf (28 uL, 0.12 mmol) in DCM (2 mL). The crude reaction was purified by flash column chromatography (PE/EA = 10:1) to provide product as yellow oil (11.4 mg, 32%). **^{1}H NMR (400 MHz, CDCl$_3$)**: δ 8.02 (d, J = 7.3 Hz, 2H), 7.57 (t, J = 7.3 Hz, 1H), 7.46 (t, J = 7.7 Hz, 2H), 7.31 (t, J = 7.5 Hz, 2H), 7.26 – 7.19 (m, 3H), 7.15 – 7.11 (m, 2H), 7.10 – 7.07 (m, 1H), 7.03 – 6.99 (m, 1H), 4.29 (dd, J = 8.6, 5.3 Hz, 1H), 3.95 – 3.79 (m, 2H), 2.94 (t, J = 5.8 Hz, 2H), 2.84 (q, J = 5.6 Hz, 2H), 2.74 (ddd, J = 13.8, 9.5, 6.0 Hz, 1H), 2.61 (ddd, J = 13.8, 9.1, 6.4 Hz, 1H), 2.37-2.32 (m, 1H), 2.20 – 2.09 (m, 1H); **^{13}C NMR (101 MHz, CDCl$_3$)**: δ 199.90, 141.67, 137.36, 135.06, 134.58, 132.99, 128.74, 128.55, 128.52, 128.49, 128.41, 126.45, 125.98, 125.94, 125.48, 66.65, 52.06, 46.97, 32.64, 30.02, 27.75; HRMS *m/z* (ESI) calcd for $C_{25}H_{26}NO$ (M + H)$^+$, 356.2009, found 356.2013. IR Qmax/cm-1 (film): 700, 713, 746, 1225, 1447, 1681, 2916, 3024.

8-(1-Oxo-1,4-diphenylbutan-2-yl)-1-phenyl-1,3,8-triazaspiro[4.5]decan-4-one (S11)

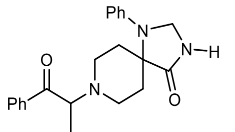

According to general procedure A, the reaction of 1-phenyl-1,3,8-triazaspiro[4.5]decan-4-one (23.1 mg, 0.1 mmol), hydrocinnamaldehyde (16 uL, 0.12 mmol), diethyl 4-benzoyl-2,6-dimethyl-1,4-dihydropyridine-3,5-dicarboxylate (44 mg, 0.12 mmol), 4 Å molecular sieve (200 mg), TBSOTf (28 uL, 0.12 mmol) in DCM (2 mL). The crude reaction was purified by flash column chromatography (PE/EA = 1:1) to provide product as colorless oil (30 mg, 66%). **^{1}H NMR (400 MHz, CDCl$_3$)**: δ 8.12 – 8.10 (m, 2H), 7.78 (s, 1H), 7.65 (t, J = 7.4 Hz, 1H), 7.53 (t, J = 7.6 Hz, 2H), 7.31 (q, J = 7.2, 6.8 Hz, 2H), 7.27 – 7.22 (m, 3H), 7.16 (t, J = 7.7 Hz, 2H), 6.80 (t, J = 7.3 Hz, 1H), 6.73 (d, J = 8.2 Hz, 2H), 4.72 (q, J = 4.4 Hz, 2H), 4.22 – 4.11 (m, 1H), 3.34 (td, J = 11.8, 3.2 Hz, 1H), 3.06 – 2.89 (m, 2H), 2.82-2.7 (m, 3H), 2.59-2.46 (m, 2H), 2.36-2.27 (m, 1H), 2.15 – 2.06

(m, 1H), 1.73 (d, *J* = 13.7 Hz, 1H), 1.67 – 1.57 (m, 1H); **^{13}C NMR (101 MHz, CDCl$_3$)**: δ 199.35, 178.59, 142.90, 141.91, 137.15, 132.72, 129.08, 128.78, 128.43, 128.38, 128.28, 125.89, 118.04, 114.02, 67.62, 59.39, 58.88, 48.09, 44.47, 32.97, 29.45, 29.15, 26.38; HRMS *m/z* (ESI) calcd for $C_{29}H_{32}N_3O_2$ (M + H)$^+$, 454.2495, found 454.2492. IR Qmax/cm-1 (film): 696, 746, 1192, 1263, 1306, 1370, 1447, 1501, 1597, 1682, 1704, 2847, 2924, 3199.

2-(3-hydroxy-8-azabicyclo[3.2.1]octan-8-yl)-1,4-diphenylbutan-1-one (S12)

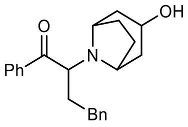

According to general procedure A, the reaction of Nortropine (13 mg, 0.1 mmol), hydrocinnamaldehyde (16 uL, 0.12 mmol), diethyl 4-benzoyl-2,6-dimethyl-1,4-dihydropyridine-3,5-dicarboxylate (44 mg, 0.12 mmol), 4 Å molecular sieve (200 mg), TBSOTf (28 uL, 0.12 mmol) in DCM (2 mL). The crude reaction was purified by flash column chromatography (PE/Acetone = 2:1) to provide product as colorless oil (13 mg, 36%). **^{1}H NMR (400 MHz, CDCl$_3$)**: δ 8.37 – 8.28 (m, 2H), 7.62 – 7.56 (m, 1H), 7.47 (dd, *J* = 8.4, 7.0 Hz, 2H), 7.27 – 7.21 (m, 2H), 7.20 – 7.14 (m, 1H), 7.08 (dd, *J* = 6.9, 1.7 Hz, 2H), 4.10 (t, *J* = 5.2 Hz, 1H), 3.86 (dd, *J* = 9.4, 4.1 Hz, 1H), 3.51 – 3.37 (m, 1H), 3.24 (dt, *J* = 6.8, 3.2 Hz, 1H), 3.09 (brs, 1H), 2.61 – 2.43 (m, 2H), 2.31 – 1.79 (m, 8H), 1.67 (dt, *J* = 14.7, 2.7 Hz, 1H), 1.56 (dt, *J* = 14.1, 2.7 Hz, 1H); **^{13}C NMR (101 MHz, CDCl$_3$)**: δ 201.29, 141.51, 136.28, 133.05, 129.28, 128.39, 128.28, 128.21, 125.93, 66.90, 65.25, 57.05, 55.30, 38.84, 38.33, 33.13, 31.97, 26.99, 26.77; HRMS *m/z* (ESI) calcd for $C_{23}H_{28}NO_2$ (M + H)$^+$, 350.2120, found 350.2117. IR Qmax/cm-1 (film): 699, 750, 1043, 1087, 1259, 1451, 1495, 1597, 1671, 1712, 2927.

8-(1-oxo-1,4-diphenylbutan-2-yl)-8-azabicyclo[3.2.1]octan-3-one (S13)

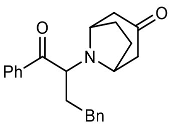

According to general procedure C, the reaction of nortropinone hydrochloride salt (16.1 mg, 0.1 mmol), hydrocinnamaldehyde (16 uL, 0.12 mmol), Et_3N (14 uL, 0.1 mmol), diethyl 4-benzoyl-2,6-dimethyl-1,4-dihydropyridine-3,5-dicarboxylate (44 mg, 0.12 mmol), 4 Å molecular sieve (200 mg), TBSOTf (28 uL, 0.12 mmol) in DCM (2 mL). The crude reaction was purified by flash column chromatography (PE/EA = 5:1) to provide product as colorless oil (16 mg, 46%). **^{1}H NMR (400 MHz, CDCl$_3$)**: δ 8.31 – 8.25 (m, 2H), 7.62 (t, *J* = 7.4 Hz, 1H), 7.50 (t, *J* = 7.7 Hz, 2H), 7.23 (dd, *J* = 22.9, 7.4 Hz, 3H), 7.14 – 7.07 (m, 2H), 4.11 (dd, *J* = 9.3, 4.2 Hz, 1H), 3.75-3.71 (m, 1H), 3.70-3.65 (m, 1H), 2.75 – 2.49 (m, 4H), 2.40-2.30 (m, 1H), 2.29 – 2.14 (m, 3H), 2.16 – 2.01 (m, 1H), 2.00 – 1.87 (m, 1H), 1.66-1.58 (m, 2H); **^{13}C NMR (101 MHz, CDCl$_3$)**: δ 209.60, 200.39, 141.11, 135.92, 133.41, 129.05, 128.62, 128.45, 128.25, 126.13, 65.51, 56.93, 56.76, 47.73, 47.44, 33.26, 31.86, 28.71, 28.26; HRMS *m/z* (ESI) calcd for $C_{23}H_{26}NO_2$ (M + H)$^+$,

348.1964, found 348.1962. IR Qmax/cm-1 (film): 699, 750, 1195, 1229, 1448, 1674, 1713, 2954.

1,4-diphenyl-2-(3-(trifluoromethyl)-5,6-dihydro-[1,2,4]triazolo[4,3-a]pyrazin-7(8H)-yl)butan-1-one (S14)

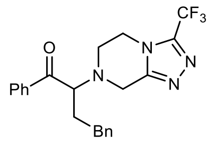

According to general procedure C, the reaction of 3-(trifluoromethyl)-5,6,7,8-tetrahydro-[1,2,4]triazolo[4,3-a]pyrazine hydrochloride salt (14.3 mg, 0.1 mmol), hydrocinnamaldehyde (16 uL, 0.12 mmol), Et_3N (14 uL, 0.1 mmol), diethyl 4-benzoyl-2,6-dimethyl-1,4-dihydropyridine-3,5-dicarboxylate (44 mg, 0.12 mmol), 4 Å molecular sieve (200 mg), TBSOTf (28 uL, 0.12 mmol) in DCM (2 mL). The crude reaction was purified by flash column chromatography (PE/EA = 2:1) to provide product as colorless oil (21 mg, 51%). **^{1}H NMR (400 MHz, $CDCl_3$)**: δ 7.84 – 7.79 (m, 2H), 7.64 – 7.57 (m, 1H), 7.52 – 7.42 (m, 2H), 7.37 – 7.30 (m, 2H), 7.28 – 7.23 (m, 1H), 7.20 – 7.16 (m, 2H), 4.38 (t, *J* = 7.1 Hz, 1H), 4.28 (d, *J* = 15.5 Hz, 1H), 4.15 (d, *J* = 15.5 Hz, 1H), 4.10-3.99 (m, 2H), 3.31 – 3.15 (m, 2H), 2.84 – 2.66 (m, 2H), 2.32-2.25 (m, 1H), 2.19 – 2.06 (m, 1H); **^{13}C NMR (101 MHz, $CDCl_3$)**: δ 199.72, 152.13, 140.55, 143.15 (q, *J* = 33 Hz) 136.23, 133.75, 128.91, 128.62, 128.47, 128.23, 126.43, 64.65, 46.57, 44.44, 44.28, 32.20, 28.82; **^{19}F NMR (376 MHz, $CDCl_3$)**: δ -63.29; HRMS *m/z* (ESI) calcd for $C_{22}H_{22}F_3N_4O$ $(M + H)^+$, 415.1746, found 415.1744. IR Qmax/cm-1 (film): 700, 750, 1017, 1138, 1174, 1202, 1272, 1342, 1448, 1496, 1682, 2932, 3026.

1'-(1-Oxo-1,4-diphenylbutan-2-yl)spiro[chromane-2,4'-piperidin]-4-one (S15)

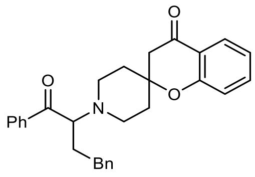

According to general procedure C, the reaction of spiro[chromane-2,4'-piperidin]-4-one hydrochloride salt (25.4 mg, 0.1 mmol), hydrocinnamaldehyde (16 uL, 0.12 mmol), Et_3N (14 uL, 0.1 mmol), diethyl 4-benzoyl-2,6-dimethyl-1,4-dihydropyridine-3,5-dicarboxylate (44 mg, 0.12 mmol), 4 Å molecular sieve (200 mg), TBSOTf (28 uL, 0.12 mmol) in DCM (2 mL). The crude reaction was purified by flash column chromatography (PE/Acetone = 5:1) to provide product as pale-yellow oil (26 mg, 60%). **^{1}H NMR (400 MHz, $CDCl_3$)**: δ 7.98 (d, *J* = 7.7 Hz, 2H), 7.85 (dd, *J* = 7.8, 1.7 Hz, 1H), 7.59 (t, *J* = 7.4 Hz, 1H), 7.52 – 7.45 (m, 3H), 7.34 – 7.28 (m, 2H), 7.25 – 7.17 (m, 3H), 7.02 – 6.92 (m, 2H), 4.13 (dd, *J* = 8.9, 5.0 Hz, 1H), 2.94-2.87 (m, 1H), 2.79 – 2.52 (m, 7H), 2.34 – 2.17 (m, 1H), 2.13 – 1.96 (m, 3H), 1.74 – 1.61 (m, 1H); **^{13}C NMR (101 MHz, $CDCl_3$)**: δ 199.61, 192.02, 159.12, 141.63, 137.29, 136.18, 132.99, 128.50, 128.46, 128.43, 128.40, 126.46, 125.98, 120.89, 120.75, 118.40, 77.87, 66.77, 47.84, 45.37, 44.52, 34.87, 34.84, 32.66, 27.66; HRMS *m/z* (ESI) calcd for $C_{29}H_{30}NO_3$ $(M + H)^+$,

440.2228, found 440.2224. IR Qmax/cm-1 (film): 701, 763, 1115, 1229, 1301, 1325, 1462, 1607, 1686, 2942.

2-(methyl(phenyl)amino)-4-phenyl-1-(thiophen-2-yl)butan-1-one (S16)

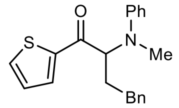

According to general procedure A, the reaction of N-methylaniline (11 mg, 0.1 mmol), hydrocinnamaldehyde (16 uL, 0.12 mmol), diethyl diethyl 2,6-dimethyl-4-(thiophene-2-carbonyl)-1,4-dihydropyridine-3,5-dicarboxylate (44 mg, 0.12 mmol), 4 Å molecular sieve (200 mg), TBSOTf (28 uL, 0.12 mmol) in DCM (2 mL).The crude reaction was purified by flash column chromatography (PE/Et_2O = 10:1) to provide product as colorless oil (15 mg, 45%). **^{1}H NMR (400 MHz, $CDCl_3$)**: δ 7.61 (dd, *J* = 3.8, 1.2 Hz, 1H), 7.57 (dd, *J* = 4.9, 1.2 Hz, 1H), 7.32 – 7.22 (m, 2H), 7.21 – 7.16 (m, 3H), 7.02 (dd, *J* = 5.0, 3.8 Hz, 1H), 6.84 – 6.80 (m, 1H), 6.78 – 6.75 (m, 2H), 4.94 (t, *J* = 7.0 Hz, 1H), 2.83 (s, 3H), 2.80 – 2.65 (m, 2H), 2.58 – 2.46 (m, 1H), 2.16-2.03 (m, 1H); **^{13}C NMR (101 MHz, $CDCl_3$)**: δ 191.78, 148.90, 142.17, 141.33, 133.81, 132.33, 129.41, 128.54, 128.42, 127.96, 126.05, 117.76, 113.12, 63.47, 33.16, 32.47, 29.32; HRMS *m/z* (ESI) calcd for $C_{21}H_{22}NOS$ $(M + H)^+$, 336.1422, found 336.1423. IR Qmax/cm-1 (film): 698, 726, 749, 1033, 1236, 1307, 1352, 1411, 1453, 1503, 1597, 1661, 2944, 3024.

2-(but-3-en-1-ylamino)-1,4-diphenylbutan-1-one (S17)

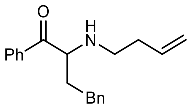

According to general procedure A, the reaction of but-3-en-1-amine (7 mg, 0.1 mmol), hydrocinnamaldehyde (16 uL, 0.12 mmol), diethyl 4-benzoyl-2,6-dimethyl-1,4-dihydropyridine-3,5-dicarboxylate (44 mg, 0.12 mmol), 4 Å molecular sieve (200 mg), $BF_3.Et_2O$ (19 uL, 0.15 mmol) in DCM (2 mL). The crude reaction was purified by flash column chromatography (PE/Et_2O = 10:1) to provide product as pale yellow oil (12 mg, 40%). **^{1}H NMR (400 MHz, $CDCl_3$)**: δ 7.80 (d, *J* = 7.6 Hz, 2H), 7.57 (t, *J* = 7.4 Hz, 1H), 7.44 (t, *J* = 7.8 Hz, 2H), 7.35 – 7.27 (m, 2H), 7.27 – 7.17 (m, 3H), 5.90-5.79 (m, 1H), 5.22 – 4.97 (m, 2H), 4.18 (dd, *J* = 8.3, 4.1 Hz, 1H), 2.80 (t, *J* = 7.5 Hz, 2H), 2.71-2.64 (m, 1H), 2.59-2.52 (m, 1H), 2.37 – 2.23 (m, *J* = 7.1, 6.7 Hz, 2H), 2.13 (brs, 1H), 2.09-1.99 (m, 1H), 1.78 (tt, *J* = 14.4, 6.9 Hz, 1H); **^{13}C NMR (101 MHz, $CDCl_3$)**: δ 203.39, 141.48, 136.40, 135.82, 133.29, 128.70, 128.68, 128.39, 128.18, 126.01, 116.15, 61.54, 47.71, 35.50, 34.75, 32.04; HRMS *m/z* (ESI) calcd for $C_{20}H_{24}NO$ $(M + H)^+$, 294.1858, found 294,1853. IR Qmax/cm-1 (film): 700, 716, 751, 1256, 1450, 1600, 1641, 1670, 1706, 2927, 2976, 3027.

***Tert*-butyl (1-oxo-1,4-diphenylbutan-2-yl)glycinate (S18)**

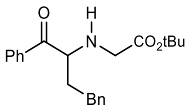

According to general procedure A, the reaction of tert-butyl glycinate (13.1 mg, 0.1

mmol), hydrocinnamaldehyde (27 uL, 0.20 mmol), diethyl 4-benzoyl-2,6-dimethyl-1,4-dihydropyridine-3,5-dicarboxylate (44 mg, 0.12 mmol), 4 Å molecular sieve (200 mg), $BF_3.Et_2O$ (19 uL, 0.15 mmol) in DCM (2 mL). The crude reaction was purified by flash column chromatography (PE/EA = 5:1) to provide product as pale-yellow oil (13.1 mg, 37%). **^{1}H NMR (400 MHz, CDCl$_3$)**: δ 7.82 – 7.78 (m, 2H), 7.63 – 7.55 (m, 1H), 7.43 (t, *J* = 7.7 Hz, 2H), 7.31-7.27(d, *J* = 4.1 Hz, 2H), 7.24 – 7.16 (m, 3H), 4.24 (dd, *J* = 8.2, 4.0 Hz, 1H), 3.43 – 3.17 (m, 2H), 2.90 – 2.72 (m, 2H), 2.10-2.01 (m, 1H), 1.87-1.77 (m, 2H), 1.46 (s, 9H); **^{13}C NMR (101 MHz, CDCl$_3$)**: δ 202.26, 171.15, 141.41, 135.65, 133.32, 128.70, 128.67, 128.40, 128.23, 126.02, 81.14, 61.14, 50.40, 35.58, 31.96, 28.07; HRMS *m/z* (ESI) calcd for $C_{22}H_{28}NO_3$ $(M + H)^+$, 354.2069, found 354.2064. IR Qmax/cm-1 (film): 699, 749, 1153, 1239, 1367, 1392, 1452, 1682, 1735, 2930, 2977.

2-((Cyclopropylmethyl)amino)-1,4-diphenylbutan-1-one (S19)

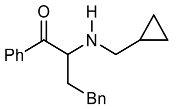

According to general procedure A, the reaction of cyclopropanamine (7.1 mg, 0.1 mmol), hydrocinnamaldehyde (16 uL, 0.12 mmol), diethyl 4-benzoyl-2,6-dimethyl-1,4-dihydropyridine-3,5-dicarboxylate (44 mg, 0.12 mmol), 4 Å molecular sieve (200 mg), $BF_3.Et_2O$ (19 uL, 0.15 mmol) in DCM (2 mL). The crude reaction was purified by flash column chromatography (PE/Acetone = 10:1) to provide product as pale-yellow oil (17.6 mg, 60%). **^{1}H NMR (400 MHz, CDCl$_3$)**: δ 7.78 (d, *J* = 7.8 Hz, 2H), 7.57 (t, *J* = 7.4 Hz, 1H), 7.43 (t, *J* = 7.6 Hz, 2H), 7.31 (d, *J* = 7.3 Hz, 1H), 7.25-7.18 (m, 4H), 4.25 (dd, *J* = 8.3, 4.0 Hz, 1H), 2.85 – 2.77 (m, 2H), 2.58 (dd, *J* = 11.8, 6.1 Hz, 1H), 2.43 (brs, 1H), 2.27 (dd, *J* = 11.8, 7.5 Hz, 1H), 2.08-1.99 (m, 1H), 1.83-1.74 (m, 1H), 1.00-0.92 (m, 1H), 0.47 (d, *J* = 8.3 Hz, 2H), 0.26 – 0.02 (m, 2H); **^{13}C NMR (101 MHz, CDCl$_3$)**: δ 203.30, 141.46, 135.73, 133.28,128.72, 128.70, 128.38, 128.15, 126.02, 60.94, 53.65, 35.54, 32.03, 11.45, 3.59, 3.33; HRMS *m/z* (ESI) calcd for $C_{20}H_{24}NO$ $(M + H)^+$, 294.1858, found 294.1856. IR Qmax/cm-1 (film): 700, 1025, 1067, 1249, 1384, 1451, 1601, 1669, 2924, 3002.

2-((4-methoxyphenyl)amino)-4-phenyl-1-(thiophen-2-yl)butan-1-one (S20)

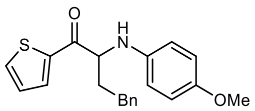

According to general procedure A, the reaction of 4-methoxyaniline (12.3 mg, 0.1 mmol), hydrocinnamaldehyde (16 uL, 0.12 mmol), diethyl diethyl 2,6-dimethyl-4-(thiophene-2-carbonyl)-1,4-dihydropyridine-3,5-dicarboxylate (44 mg, 0.12 mmol), 4 Å molecular sieve (200 mg), $BF_3.Et_2O$ (19 uL, 0.15 mmol) in DCM (2 mL). The crude reaction was purified by flash column chromatography (PE/Acetone = 10:1) to provide product as pale yellow oil (14 mg, 40%). **^{1}H NMR (400 MHz, CDCl$_3$)**: δ 7.66 (d, *J* = 5.0 Hz, 1H), 7.60 (d, *J* = 3.9 Hz, 1H), 7.33 (t, *J* = 7.3 Hz, 2H), 7.28 – 7.20 (m, 3H), 7.12 (t, *J* = 4.3 Hz, 1H), 6.76 (d, *J* = 8.8 Hz, 2H), 6.64 (d, *J* = 8.8 Hz, 2H), 4.69 (dd, *J* = 8.4, 4.3 Hz, 1H), 4.21 (brs, 1H), 3.74 (s, 3H), 2.87 (t, *J* = 7.5 Hz, 2H), 2.32-2.23 (m, 1H), 2.09-2.00 (m, 1H); **^{13}C NMR (101 MHz, CDCl$_3$)**: δ 194.54, 152.81, 141.74, 141.16, 140.94, 134.34, 132.30,

128.63, 128.52, 128.14, 126.22, 115.59, 114.88, 60.24, 55.66, 35.62, 31.92; HRMS *m/z* (ESI) calcd for $C_1H_{18}NO_2S$ (M + H)$^+$, 352.1371, found 352.1374. IR Qmax/cm-1 (film): 700, 728, 820, 1036, 1235, 1409, 1453, 1510, 1659, 2932, 3025.

1,4-diphenyl-2-((2-(thiophen-2-yl)ethyl)amino)butan-1-one (S21)

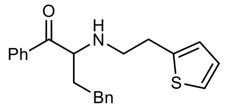

According to general procedure A, the reaction of 2-(thiophen-2-yl)ethan-1-amine (12.7 mg, 0.1 mmol), hydrocinnamaldehyde (16 uL, 0.12 mmol), diethyl 4-benzoyl-2,6-dimethyl-1,4-dihydropyridine-3,5-dicarboxylate (44 mg, 0.12 mmol), 4 Å molecular sieve (200 mg), $BF_3.Et_2O$ (19 uL, 0.15 mmol) in DCM (2 mL). The crude reaction was purified by flash column chromatography (PE/EA = 10:1) to provide product as pale yellow oil (14 mg, 40%). **^{1}H NMR (400 MHz, CDCl$_3$)**: δ 7.78 (d, *J* = 7.7 Hz, 2H), 7.57 (t, *J* = 7.4 Hz, 1H), 7.43 (t, *J* = 7.6 Hz, 2H), 7.29 (d, *J* = 8.0 Hz, 2H), 7.25 – 7.14 (m, 4H), 6.95 (t, *J* = 4.3 Hz, 1H), 6.88 (d, *J* = 3.4 Hz, 1H), 4.17 (dd, *J* = 8.6, 3.9 Hz, 1H), 3.05 (t, *J* = 6.9 Hz, 2H), 3.00 – 2.93 (m, 1H), 2.81 (t, *J* = 7.5 Hz, 2H), 2.76-2.69 (m, 1H), 2.07-1.98 (m, 1H), 1.96 (brs, 3H), 1.80-1.70 (m, 1H); **^{13}C NMR (101 MHz, CDCl$_3$)**: δ 203.18, 142.67, 141.47, 135.70, 133.29, 128.69, 128.40, 128.18, 126.66, 126.02, 124.84, 123.48, 61.63, 49.87, 35.53, 32.04, 31.21; HRMS *m/z* (ESI) calcd for $C_{22}H_{24}NOS$ (M + H)$^+$, 350.1579, found 350.1577. IR Qmax/cm-1 (film): 699, 1027, 1056, 1066, 1075, 1249, 1382, 1393, 1448, 2900, 2971, 2987.

2-((2-(1H-indol-3-yl)ethyl)amino)-1,4-diphenylbutan-1-one (S22)

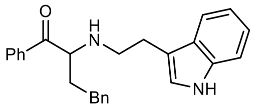

According to general procedure A, the reaction of 2-(1H-indol-3-yl)ethan-1-amine (16 mg, 0.1 mmol), hydrocinnamaldehyde (16 uL, 0.12 mmol), diethyl 4-benzoyl-2,6-dimethyl-1,4-dihydropyridine-3,5-dicarboxylate (44 mg, 0.12 mmol), 4 Å molecular sieve (200 mg), $BF_3.Et_2O$ (19 uL, 0.15 mmol) in DCM (2 mL). The crude reaction was purified by flash column chromatography (PE/EA = 4:1) to provide product as pale yellow oil (15 mg, 40%). **^{1}H NMR (400 MHz, CDCl$_3$)**: δ 8.04 (s, 1H), 7.75 (d, *J* = 7.7 Hz, 2H), 7.62 (d, *J* = 8.0 Hz, 1H), 7.56 (t, *J* = 7.5 Hz, 1H), 7.45 – 7.35 (m, 3H), 7.27 – 7.18 (m, 4H), 7.15 – 7.08 (m, 4H), 4.22 (dd, *J* = 8.1, 4.1 Hz, 1H), 3.06-2.98 (m, 3H), 2.92-2.85 (m, 1H), 2.76 (t, *J* = 7.6 Hz, 2H), 2.57 – 2.41 (m, 1H), 2.09-2.00 (m, 1H), 1.85-1.75 (m, 1H); **^{13}C NMR (101 MHz, CDCl$_3$)**: δ 202.91, 141.27, 136.30, 135.72, 133.33, 129.85, 128.69, 128.62, 128.37, 128.18, 126.00, 121.98, 121.82, 119.26, 118.85, 113.86, 111.09, 61.46, 48.44, 35.13, 31.79, 26.06; HRMS *m/z* (ESI) calcd for $C_{26}H_{27}N_2O$ (M + H)$^+$, 383.2118, found 383.2121. IR Qmax/cm-1 (film): 699, 717, 744, 1251, 1382, 1453, 1495, 1600, 1671, 2976, 3027, 3058, 3274.

2-(Methyl(3-phenyl-3-(4-(trifluoromethyl)phenoxy)propyl)amino)-1,4-diphenylbutan-1-one (S23)

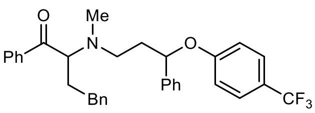

According to general procedure A, the reaction of fluoxetine hydrochloride salt (34.5 mg, 0.1 mmol), hydrocinnamaldehyde (27 uL, 0.2 mmol), Et_3N (14 uL, 0.1 mmol), diethyl 4-benzoyl-2,6-dimethyl-1,4-dihydropyridine-3,5-dicarboxylate (44 mg, 0.12 mmol), 4 Å molecular sieve (200 mg), TBSOTf (28 uL, 0.12 mmol) in DCM (2 mL). The crude reaction was purified by flash column chromatography (PE/EA = 10:1) to provide product as yellow oil (33 mg, 62%, 1.1:1 dr). **^{1}H NMR (400 MHz, $CDCl_3$)**: δ 7.88 (d, *J* = 7.4 Hz, 1H), 7.82 (d, *J* = 7.4 Hz, 1H), 7.56 – 7.39 (m, 2H), 7.38 – 7.13 (m, 13H), 6.84 (d, *J* = 8.5 Hz, 1H), 6.70 (d, *J* = 8.5 Hz, 1H), 5.20 (dd, *J* = 8.6, 4.2 Hz, 0.5H), 5.10 (dd, *J* = 9.3, 3.5 Hz, 0.5H), 4.14 (ddd, *J* = 15.8, 9.0, 4.6 Hz, 1H), 2.93-2.83 (m, 1H), 2.75 – 2.53 (m, 2H), 2.55 – 2.45 (m, 1H), 2.46 (s, 1.5H), 2.34 (s, 1.5H), 2.32 – 2.18 (m, 1H), 2.19 – 1.86 (m, 3H); **^{13}C NMR (101 MHz, $CDCl_3$)**: δ 199.56, 199.00, 160.55, 160.43, 141.76, 141.69, 141.54, 141.32, 137.25, 136.92, 128.72, 128.69, 128.41, 128.39, 128.37, 128.33, 127.69, 127.59, 126.69 (q, *J* = 5.6 Hz), 126.53 (q, *J* = 5.6 Hz), 125.95, 125.92, 125.70, 125.55, 126.69, 122.61 (q, *J* = 26.8 Hz), 122.28 (q, *J* = 26.5 Hz), 115.72, 115.46, 66.59, 66.19, 50.30, 48.93, 38.35, 37.37, 37.34, 37.20, 32.88, 32.80, 26.44, 26.12; **^{19}F NMR (376 MHz, $CDCl_3$)**: δ -61.45, -61.47; dr = 1:1; HRMS *m/z* (ESI) calcd for $C_{33}H_{33}F_3NO_2$ (M + H)$^+$, 532.2463, found 532.2467. IR Qmax/cm-1 (film): 699, 751, 834, 955, 1009, 1067, 1110, 1160, 1177, 1250, 1327, 1452, 1516, 1614, 1683, 2928, 3026.

2-(Methyl((S)-3-(naphthalen-1-yloxy)-3-(thiophen-2-yl)propyl)amino)-1,4-diphenylbutan-1-one (S24)

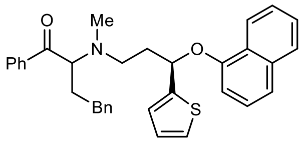

According to general procedure A, the reaction of duloxetine hydrochloride salt (33.4 mg, 0.1 mmol), hydrocinnamaldehyde (27 uL, 0.2 mmol), Et_3N (14 uL, 0.1 mmol), diethyl 4-benzoyl-2,6-dimethyl-1,4-dihydropyridine-3,5-dicarboxylate (44 mg, 0.12 mmol), 4 Å molecular sieve (200 mg), TBSOTf (28 uL, 0.12 mmol) in DCM (2 mL). The crude reaction was purified by flash column chromatography (PE/EA = 10:1) to provide product as yellow oil (34 mg, 66%, 1.1:1 dr). **^{1}H NMR (400 MHz, $CDCl_3$)**: δ 8.37 (td, *J* = 6.4, 2.7 Hz, 1H), 7.93 – 7.72 (m, 3H), 7.59 – 7.33 (m, 5H), 7.32 – 7.17 (m, 6H), 7.18 – 7.10 (m, 2H), 7.07 – 6.89 (m, 2H), 6.73 (d, *J* = 7.7 Hz, 1H), 5.69 (ddd, *J* = 22.7, 8.0, 4.8 Hz, 1H), 4.15 (dd, *J* = 9.0, 4.6 Hz, 0.52H), 4.06 (dd, *J* = 8.2, 5.6 Hz, 0.48H), 2.92 (dt, *J* = 13.5, 7.1 Hz, 1H), 2.82 – 2.72 (m, 1H), 2.70 – 2.10 (m, 8H), 2.00 – 1.89 (m, 1H); **^{13}C NMR (101 MHz, $CDCl_3$)**: δ 200.19, 199.56, 153.46, 153.24, 145.55, 145.44, 141.74, 137.32, 137.17, 134.55, 132.74, 132.69, 128.43, 128.40, 128.36, 128.33, 128.26, 127.46, 127.45, 126.51, 126.46, 126.26, 126.24, 126.08, 126.05, 125.90, 125.87, 125.75, 125.68, 125.20, 125.15, 124.61, 124.56, 124.37, 122.11, 122.08, 120.41, 106.91, 106.83, 73.78, 73.58, 66.22, 50.63, 50.36, 37.65, 37.53, 37.50, 37.23, 32.79, 32.74, 27.54, 26.29; HRMS *m/z* (ESI) calcd for $C_{34}H_{34}NO_2S$ (M + H)$^+$, 520.2310, found 520.2313.

IR Qmax/cm-1 (film): 698, 732, 771, 791, 1065, 1094, 1235, 1263, 1396, 1447, 1461, 1578, 1595, 1681, 2954.

3,4,5-Trimethoxy-N-(1-(1-oxo-1,4-diphenylbutan-2-yl)piperidin-3-yl)benzamide (S25)

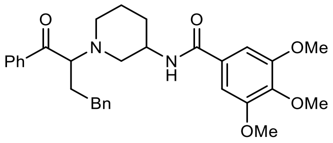

According to general procedure A, the reaction of troxipide (29.4 mg, 0.1 mmol), hydrocinnamaldehyde (27 uL, 0.2 mmol), diethyl 4-benzoyl-2,6-dimethyl-1,4-dihydropyridine-3,5-dicarboxylate (44 mg, 0.12 mmol), 4 Å molecular sieve (200 mg), TBSOTf (28 uL, 0.12 mmol) in DCM (2 mL). The crude reaction was purified by flash column chromatography (PE/EA = 1:1) to provide product as yellow oil (45 mg, 87%, 1.1:1 dr). **^{1}H NMR (400 MHz, CDCl$_3$)**: δ 7.89 – 7.81 (m, 2H), 7.55 – 7.45 (m, 1H), 7.38 (t, *J* = 7.8 Hz, 1H), 7.36 – 7.28 (m, 1H), 7.30 – 7.22 (m, 2H), 7.22 – 7.10 (m, 3H), 6.92 (s, 1H), 6.87 (s, 1H), 6.78 (d, *J* = 8.3 Hz, 0.42H), 6.61 (d, *J* = 7.9 Hz, 0.48H), 4.30 – 4.15 (m, 1H), 4.15 – 4.07 (m, 1H), 3.87 (d, *J* = 6.8 Hz, 3H), 3.79 (s, 1H), 3.77 (s, 3H), 2.90 – 2.38 (m, 6H), 2.31 – 2.12 (m, 1H), 2.05 – 1.91 (m, 1H), 1.82 – 1.50 (m, 4H); **^{13}C NMR (101 MHz, CDCl$_3$)**: δ 199.51, 199.01, 166.23, 166.11, 153.04, 152.99, 141.29, 141.24, 140.65, 136.86, 136.80, 133.07, 133.05, 130.33, 130.30, 128.61, 128.54, 128.44, 128.42, 128.34, 128.29, 128.16, 128.15, 126.07, 126.05, 104.28, 104.23, 66.60, 66.10, 60.82, 60.79, 56.21, 56.12, 55.83, 53.39, 51.70, 49.19, 45.66, 45.40, 32.90, 32.38, 28.95, 28.88, 27.57, 27.28, 22.72, 22.22; HRMS *m/z* (ESI) calcd for $C_{31}H_{37}N_2O_5$ $(M + H)^+$, 517.2702, found 517.2702. IR Qmax/cm-1 (film): 700, 730, 753, 1002, 1126, 1179, 1231, 1339, 1413, 1448, 1496, 1539, 1582, 1636, 1681, 2937, 3312.

2-((3S,4R)-3-((Benzo[d][1,3]dioxol-5-yloxy)methyl)-4-(4-fluorophenyl)piperidin-1-yl)-1,4-diphenylbutan-1-one (S26)

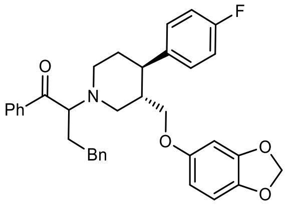

According to general procedure A, the reaction of paroxetine hydrochloride salt (37.5 mg, 0.1 mmol), hydrocinnamaldehyde (27 uL, 0.2 mmol), Et_3N (14 uL, 0.1 mmol), diethyl 4-benzoyl-2,6-dimethyl-1,4-dihydropyridine-3,5-dicarboxylate (44 mg, 0.12 mmol), 4 Å molecular sieve (200 mg), TBSOTf (28 uL, 0.12 mmol) in DCM (2 mL). The crude reaction was purified by flash column chromatography (PE/Acetone = 10:1) to provide product as yellow oil (42 mg, 76%, 1.1:1 dr). **^{1}H NMR (400 MHz, CDCl$_3$)**: δ 8.03 (dd, *J* = 8.4, 1.7 Hz, 2H), 7.63-7.57 (m, 1H), 7.52-7.47 (m, 2H), 7.39 – 7.27 (m, 2H), 7.23 (t, *J* = 6.5 Hz, 3H), 7.17-7.11 (m, 2H), 7.02 – 6.94 (m, 2H), 6.64 (ddd, *J* = 15.5, 8.4, 1.5 Hz, 1H), 6.38 (t, *J* = 2.0 Hz, 0.5H), 6.31 (t, *J* = 2.0 Hz, 0.5H), 6.17 (dt, *J* = 8.5, 2.0 Hz, 0.5H), 6.10 (dt, *J* = 8.5, 2.0 Hz, 0.5H), 5.91 (d, *J* = 1.5 Hz, 1H), 5.89 (d, *J* = 1.5 Hz, 1H), 4.28 –

4.14 (m, 1H), 3.62 – 3.57 (m, 0.5H), 3.53 – 3.41 (m, 1H), 3.42 – 3.33 (m, 1H), 3.19 – 3.07 (m, 1H), 2.89 (d, *J* = 11.3 Hz, 0.5H), 2.80 – 2.57 (m, 3H), 2.50 – 2.04 (m, 5H), 1.91 – 1.69 (m, 2H); **^{13}C NMR (101 MHz, $CDCl_3$)**: δ 200.07, 199.78, 161.45 (d, *J* = 242.7 Hz), 154.34, 154.31, 148.11, 148.05, 141.80, 141.74, 141.54, 141.49, 139.68, 139.65, 139.62, 137.42, 137.37, 132.95, 132.93, 128.80, 128.77, 128.72, 128.70, 128.54, 128.51, 128.47, 128.39, 125.94, 115.40, 115.20, 107.81, 107.75, 105.63, 105.60, 101.05, 101.01, 98.00, 69.66, 69.52, 67.17, 67.02, 53.79, 50.23, 50.07, 44.36, 44.24, 42.80, 42.63, 35.02, 34.85, 32.79, 32.67, 28.21, 27.75; **^{19}F NMR (376 MHz, $CDCl_3$)**: δ -117.55, -117.57; HRMS *m/z* (ESI) calcd for $C_{35}H_{35}FNO_4$ $(M + H)^+$, 552.2550, found 552.2549. IR Qmax/cm-1 (film): 700, 832, 1038, 1134, 1158, 1184, 1223, 1447, 1467, 1487, 1502, 1508, 1682, 2917.

3-(1-Oxo-1,4-diphenylbutan-2-yl)-1,2,3,4,5,6-hexahydro-8H-1,5-methanopyrido[1,2-a][1,5]diazocin-8-one (S27)

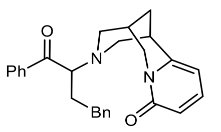

According to general procedure A, the reaction of cytisine (19 mg, 0.1 mmol), hydrocinnamaldehyde (27 uL, 0.2 mmol), diethyl 4-benzoyl-2,6-dimethyl-1,4-dihydropyridine-3,5-dicarboxylate (44 mg, 0.12 mmol), 4 Å molecular sieve (200 mg), TBSOTf (28 uL, 0.12 mmol) in DCM (2 mL). The crude reaction was purified by flash column chromatography (PE/Acetone = 1:1) to provide product as yellow oil (28 mg, 68%).**^{1}H NMR (400 MHz, $CDCl_3$)**: δ 7.73 – 7.63 (m, 2H), 7.51 (t, *J* = 7.4 Hz, 1H), 7.34 – 7.26 (m, 5H), 7.19 (t, *J* = 7.3 Hz, 1H), 7.12 – 7.03 (m, 2H), 6.40 (dd, *J* = 9.1, 1.4 Hz, 1H), 6.02 (dd, *J* = 6.7, 1.4 Hz, 1H), 3.90 (t, *J* = 7.1 Hz, 1H), 3.80 (dd, *J* = 15.4, 6.6 Hz, 1H), 3.69 (d, *J* = 15.4 Hz, 1H), 3.09 – 2.96 (m, 3H), 2.78 – 2.62 (m, 2H), 2.46 – 2.27 (m, 3H), 2.09-1.99 (m, 1H), 1.92 – 1.72 (m, 3H); **^{13}C NMR (101 MHz, $CDCl_3$)**: δ 199.81, 163.24, 151.35, 141.36, 138.28, 136.42, 133.01, 128.52, 128.35, 128.33, 127.94, 125.90, 116.84, 104.41, 66.54, 56.66, 56.49, 49.74, 35.58, 32.06, 27.96, 26.99, 25.77; HRMS *m/z* (ESI) calcd for $C_{27}H_{29}N_2O_2$ $(M + H)^+$, 413.2229, found 413.2228. IR Qmax/cm-1 (film): 699, 731, 797, 909, 1138, 1447, 1546, 1566, 1651, 1681, 2935.

2-(4-(Dibenzo[b,f][1,4]thiazepin-11-yl)piperazin-1-yl)-1,4-diphenylbutan-1-one (S28)

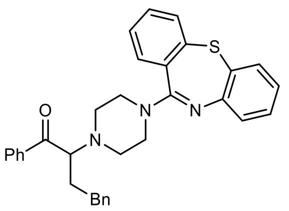

According to general procedure A, the reaction of norquetiapine (29.5 mg, 0.1 mmol), hydrocinnamaldehyde (27 uL, 0.2 mmol), diethyl 4-benzoyl-2,6-dimethyl-1,4-dihydropyridine-3,5-dicarboxylate (44 mg, 0.12 mmol), 4 Å molecular sieve (200 mg), TBSOTf (28 uL, 0.12 mmol) in DCM (2 mL). The crude reaction was purified by flash

column chromatography (PE/EA = 10:1) to provide product as yellow foam (44 mg, 85%). **^{1}H NMR (400 MHz, CDCl$_3$)**: δ 8.01 (dd, *J* = 8.1, 3.3 Hz, 2H), 7.60 (q, *J* = 6.9 Hz, 1H), 7.54-7.45 (m, 3H), 7.42 (d, *J* = 7.7 Hz, 1H), 7.38 – 7.26 (m, 5H), 7.25-7.17 (m, 4H), 7.10 (t, *J* = 7.7 Hz, 1H), 6.91 (t, *J* = 7.5 Hz, 1H), 4.22-4.16 (m, 1H), 3.46 (s, 3H), 2.89 – 2.66 (m, 5H), 2.65 – 2.55 (m, 1H), 2.33-2.22 (m, 1H), 2.14-2.04 (m, 1H); **^{13}C NMR (101 MHz, CDCl$_3$)**: δ 199.86, 199.57, 160.89, 148.76, 141.47, 141.41, 139.87, 139.84, 137.27, 137.09, 134.00, 133.10, 133.06, 132.10, 132.05, 130.72, 130.68, 129.96, 129.06, 129.01, 128.96, 128.55, 128.50, 128.48, 128.43, 128.40, 128.37, 128.27, 128.19, 128.16, 127.98, 127.96, 125.98, 125.26, 122.79, 66.65, 66.38, 49.24, 32.49, 32.40, 28.12, 27.77; HRMS *m/z* (ESI) calcd for $C_{33}H_{32}N_3OS$ $(M + H)^+$, 518.2266, found 518.2261. IR Qmax/cm-1 (film): 700, 741, 761, 1000, 1016, 1142, 1245, 1256, 1305, 1366, 1413, 1452, 1557, 1574, 1596, 1681, 2849, 2929.

2-((3-(10,11-dihydro-5H-dibenzo[a,d][7]annulen-5-ylidene)propyl)(methyl)amino)-1,4-diphenylbutan-1-one (S29)

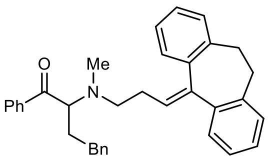

According to general procedure C, the reaction of nortriptyline hydrochloride salt (30 mg, 0.1 mmol), hydrocinnamaldehyde (27 uL, 0.2 mmol), Et_3N (0.1 mmol, 14 uL), diethyl 4-benzoyl-2,6-dimethyl-1,4-dihydropyridine-3,5-dicarboxylate (44 mg, 0.12 mmol), 4 Å molecular sieve (200 mg), TBSOTf (28 uL, 0.12 mmol) in DCM (2 mL). The crude reaction was purified by flash column chromatography (PE/Et_2O = 10:1) to provide product as yellow foam (38 mg, 76%). **^{1}H NMR (400 MHz, CDCl$_3$)**: δ 7.98 – 7.91 (m, 2H), 7.47 (t, *J* = 7.4 Hz, 1H), 7.37 – 7.27 (m, 4H), 7.25 – 7.10 (m, 10H), 7.05 (d, *J* = 7.2 Hz, 1H), 5.79 (t, *J* = 7.5 Hz, 1H), 4.11 (dd, *J* = 8.9, 4.7 Hz, 1H), 3.47 – 2.84 (m, 3H), 2.80 – 2.59 (m, 4H), 2.57 – 2.43 (m, 1H), 2.31-2.20 (m, 6H), 1.95 (s, 1H); **^{13}C NMR (101 MHz, CDCl$_3$)**: δ 199.75, 154.66, 143.30, 141.86, 141.19, 140.07, 139.38, 137.09, 136.90, 132.65, 129.88, 129.80, 128.50, 128.48, 128.41, 128.33, 128.24, 128.20, 127.85, 127.27, 126.82, 125.86, 125.58, 66.66, 54.09, 33.72, 32.84, 31.86, 28.11, 26.22; HRMS *m/z* (ESI) calcd for $C_{35}H_{36}NO$ $(M + H)^+$, 486.2797, found 486.2794. IR Qmax/cm-1 (film): 698, 743, 754, 767, 776, 908, 1227, 1446, 1485, 1682, 2924.

Tert-butyl 4-(1-oxo-1-phenylpentan-2-yl)piperazine-1-carboxylate (S30)

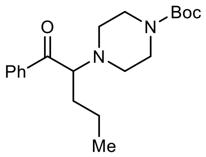

According to general procedure A, the reaction of tert-butyl piperazine-1-carboxylate (18.6 mg, 0.1 mmol), butyraldehyde (8.7 mg, 0.12 mmol), diethyl 4-benzoyl-2,6-dimethyl-1,4-dihydropyridine-3,5-dicarboxylate (44 mg, 0.12 mmol), 4 Å molecular sieve (200 mg), TBSOTf (28 uL, 0.12 mmol) in DCM (2 mL). The crude reaction was purified by flash column chromatography (PE/EA = 10:1) to provide product as yellow

oil (20.7 mg, 60%). **^{1}H NMR (400 MHz, CDCl$_3$)**: δ 8.06 – 8.02 (m, 2H), 7.61 – 7.55 (m, 1H), 7.48 (dd, *J* = 8.3, 6.9 Hz, 2H), 4.08 (dd, *J* = 9.0, 5.0 Hz, 1H), 3.38 (t, *J* = 5.1 Hz, 4H), 2.75 – 2.51 (m, 4H), 1.96 – 1.80 (m, 1H), 1.76 – 1.60 (m, 1H), 1.45 (s, 9H), 1.36 – 1.20 (m, 2H), 0.93 (t, *J* = 7.3 Hz, 3H); **^{13}C NMR (101 MHz, CDCl$_3$)**: δ 199.86, 154.66, 137.35, 132.99, 128.52, 128.47, 79.54, 67.85, 28.58, 28.39, 19.88, 14.19; HRMS *m/z* (ESI) calcd for $C_{20}H_{31}N_2O_3$ $(M + H)^+$, 347.2335, found 347.2333. IR Qmax/cm-1 (film): 700, 1005, 1171, 1245, 1364, 1420, 1448, 1689, 2861, 2931, 2959.

tert-butyl 4-(1-(6,6-dimethylbicyclo[3.1.1]hept-2-en-2-yl)-2-oxo-2-phenylethyl)piperazine-1-carboxylate (S31)

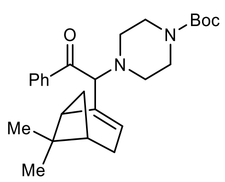

According to general procedure A, the reaction of tert-butyl piperazine-1-carboxylate (18.6 mg, 0.1 mmol), (-)-myrtenal (18 uL, 0.12 mmol), diethyl 4-benzoyl-2,6-dimethyl-1,4-dihydropyridine-3,5-dicarboxylate (44 mg, 0.12 mmol), 4 Å molecular sieve (200 mg), TBSOTf (28 uL, 0.12 mmol) in DCM (2 mL). The crude reaction was purified by flash column chromatography (PE/EA = 10:1) to provide product as pale yellow oil (13 mg, 31%, dr = 1.9 :1). **^{1}H NMR (400 MHz, CDCl$_3$)**: δ 8.07 – 7.96 (m, 2H), 7.59 – 7.52 (m, 1H), 7.47-7.41 (m, 2H), 5.67 (s, *J* = 1.7 Hz, 0.64H), 5.52 (q, *J* = 2.7 Hz, 0.33H), 4.47 (d, *J* = 4.7 Hz, 1H), 3.59 – 3.42 (m, 4H), 2.71 – 2.54 (m, 2H), 2.50 – 2.38 (m, 4H), 2.33 – 2.03 (m, 4H), 1.47 (s, 9H), 1.29 (s, 1H), 1.21 (s, 2H), 1.14 – 0.92 (m, 2H), 0.73 (s, 1.16H), 0.37 (s, 1.89H); **^{13}C NMR (101 MHz, CDCl$_3$)**: δ 197.99, 196.41, 154.76, 154.70, 142.90, 142.43, 137.12, 137.06, 133.01, 132.89, 128.95, 128.69, 128.62, 128.39, 128.32, 126.63, 79.52, 75.72, 61.87, 51.52, 50.75, 44.51, 41.55, 40.58, 40.42, 37.82, 37.56, 31.94, 31.92, 31.86, 31.70, 28.41, 28.36, 26.15, 25.87, 23.95, 21.35, 20.53, 13.25; HRMS *m/z* (ESI) calcd for $C_{26}H_{37}N_2O_3$ $(M + H)^+$, 425.2804, found 425.2802. IR Qmax/cm-1 (film): 689, 724, 76, 871, 965, 1001, 1125, 1171, 1248, 1286, 1364, 1421, 1447, 1688, 2917, 2976.

2-cyclohexyl-2-((4-methoxyphenyl)amino)-1-phenylethan-1-one (S32)

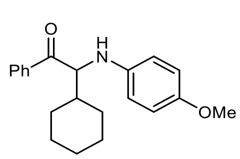

According to general procedure A, the reaction of 4-methoxyaniline (12.3 mg, 0.1 mmol), cyclohexanecarbaldehyde (13.5 mg, 0.12 mmol), diethyl 4-benzoyl-2,6-dimethyl-1,4-dihydropyridine-3,5-dicarboxylate (44 mg, 0.12 mmol), 4 Å molecular sieve (200 mg), $BF_3.Et_2O$ (19 uL, 0.15 mmol) in DCM (2 mL). The crude reaction was purified by flash column chromatography (PE/EA = 10:1) to provide product as pale yellow oil (24 mg, 74%). **^{1}H NMR (400 MHz, CDCl$_3$)**: δ 7.98 (d, *J* = 6.9 Hz, 2H), 7.65 – 7.57 (m, 1H), 7.51 (t, *J* = 7.6 Hz, 2H), 6.76 (d, *J* = 9.0 Hz, 2H), 6.70 (d, *J* = 9.0 Hz, 2H),

4.79 (d, *J* = 4.3 Hz, 1H), 4.40 (s, 1H), 3.74 (s, 3H), 1.91 – 1.71 (m, 4H), 1.65 (d, *J* = 7.3 Hz, 2H), 1.55 – 1.35 (m, 1H), 1.32 – 1.10 (m, 4H); **^{13}C NMR (101 MHz, $CDCl_3$)**: δ 201.81, 152.52, 142.44, 136.16, 133.36, 128.77, 128.26, 115.71, 114.83, 64.71, 55.71, 41.82, 30.87, 27.68, 26.37, 26.13, 26.01; HRMS *m/z* (ESI) calcd for $C_{21}H_{26}NO_2$ (M + H)$^+$, 324.1964, found 324.1962. IR Qmax/cm-1 (film): 688, 819, 1038, 1179, 1239, 1447, 1510, 1678, 2851, 2926.

Cyclopropyl(1-((4-methoxyphenyl)amino)cyclohexyl)methanone (S33)

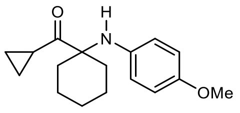

According to general procedure B, the reaction of 4-methoxyaniline (12.3 mg, 0.1 mmol), cyclohexanone (21 uL, 0.2 mmol), diethyl 4-(cyclopropanecarbonyl)-2,6-dimethyl-1,4-dihydropyridine-3,5-dicarboxylate (39 mg, 0.12 mmol), 4 Å molecular sieve (200 mg), $BF_3.Et_2O$ (19 uL, 0.15 mmol) in DCM (2 mL). The crude reaction was purified by flash column chromatography (PE/Et_2O = 5:1) to provide product as yellow oil (15 mg, 55%). **^{1}H NMR (400 MHz, $CDCl_3$)**: δ 6.74 (d, *J* = 8.8 Hz, 2H), 6.49 (d, *J* = 9.0 Hz, 2H), 3.91 (brs, 1H), 3.75 (s, 3H), 2.70-2.64 (m, 1H), 1.99 – 1.89 (m, 2H), 1.84-1.76 (m, 2H), 1.65-1.58 (m, 3H), 1.55-1.43 (m, 2H), 1.39 – 1.27 (m, 1H), 1.07 – 0.97 (m, 2H), 0.86-0.80 (m, 2H); **^{13}C NMR (101 MHz, $CDCl_3$)**: δ 216.50, 152.36, 139.05, 116.03, 114.60, 64.27, 55.62, 31.20, 25.33, 21.24, 15.41, 11.64; HRMS *m/z* (ESI) calcd for $C_{17}H_{24}NO_2$ (M + H)$^+$, 274.1807, found 274.1804. IR Qmax/cm-1 (film): 820, 909, 996, 1033, 1237, 1371, 1450, 1511, 1689, 2933.

(1-((4-methoxyphenyl)amino)cyclobutyl)(thiophen-2-yl)methanone (S34)

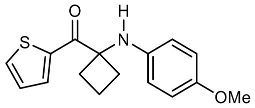

According to general procedure B, the reaction of 4-methoxyaniline (12.3 mg, 0.1 mmol), cyclobutanone (17 uL, 0.2 mmol), diethyl diethyl 2,6-dimethyl-4-(thiophene-2-carbonyl)-1,4-dihydropyridine-3,5-dicarboxylate (44 mg, 0.12 mmol), 4 Å molecular sieve (200 mg), $BF_3.Et_2O$ (19 uL, 0.15 mmol) in DCM (2 mL). The crude reaction was purified by flash column chromatography (PE/Acetone = 10:1) to provide product as pale yellow oil (18 mg, 63%). **^{1}H NMR (400 MHz, $CDCl_3$)**: δ 8.05 (dd, *J* = 3.9, 1.2 Hz, 1H), 7.49 (dd, *J* = 4.9, 1.3 Hz, 1H), 7.05 (dd, *J* = 5.0, 3.8 Hz, 1H), 6.79 – 6.64 (m, 2H), 6.45 (d, *J* = 8.9 Hz, 2H), 4.28 (brs, 1H), 3.70 (s, 3H), 3.08 – 2.78 (m, 2H), 2.22 – 2.08 (m, 2H), 2.07 – 1.90 (m, 2H); **^{13}C NMR (101 MHz, $CDCl_3$)**: δ 195.29, 152.66, 138.91, 137.03, 134.27, 133.57, 127.16, 114.93, 114.87, 65.07, 55.56, 31.79, 14.37; HRMS *m/z* (ESI) calcd for $C_{16}H_{18}NO_2S$ (M + H)$^+$, 288.1058, found 288.1053. IR Qmax/cm-1 (film): 731, 821, 1036, 1178, 1235, 1291, 1355, 1409, 1511, 1651, 2949, 2991, 3375.

(1-((4-iodophenyl)amino)cyclobutyl)(phenyl)methanone (S35)

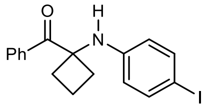

According to general procedure B, the reaction of 4-iodoaniline (21.9 mg, 0.1 mmol), cyclobutanone (17 uL, 0.2 mmol), diethyl 4-benzoyl-2,6-dimethyl-1,4-dihydropyridine-3,5-dicarboxylate (44 mg, 0.12 mmol), 4 Å molecular sieve (200 mg), $BF_3.Et_2O$ (19 uL, 0.15 mmol) in DCM (2 mL). The crude reaction was purified by flash column chromatography (DCM/PE/EA = 3:50:1) to provide product as pale yellow oil (15 mg, 40%). **^{1}H NMR (400 MHz, CDCl$_3$)**: δ 7.94 – 7.88 (m, 2H), 7.51 – 7.44 (m, 1H), 7.39 – 7.34 (m, 2H), 7.34 – 7.27 (m, 2H), 6.20 (d, *J* = 8.8 Hz, 2H), 4.46 (s, 1H), 3.11 – 2.96 (m, 2H), 2.37 – 2.21 (m, 2H), 2.17 – 1.94 (m, 2H); **^{13}C NMR (101 MHz, CDCl$_3$)**: δ 202.02, 144.78, 137.77, 134.31, 132.54, 128.83, 128.14, 115.50, 79.01, 64.82, 31.68, 14.75; HRMS *m/z* (ESI) calcd for $C_{17}H_{17}INO$ $(M + H)^+$, 378.0355, found 378.0353. IR Qmax/cm-1 (film): 689, 716, 809, 1181, 1223, 1253, 1283, 1297, 1315, 1446, 1491, 1589, 1668, 2949, 3392.

2,2'-((1-phenylethyl)azanediyl)bis(1-cyclopropylethan-1-one) (S36)

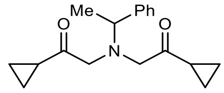

According to general procedure A, the reaction of 1-phenylethan-1-amine (12.3 mg, 0.1 mmol), formaldehyde solution (37% in water) (25 mg, 0.3 mmol), diethyl 4-(cyclopropanecarbonyl)-2,6-dimethyl-1,4-dihydropyridine-3,5-dicarboxylate (39 mg, 0.12 mmol), 4 Å molecular sieve (200 mg), $BF_3.Et_2O$ (19 uL, 0.15 mmol) in DCM (2 mL). The crude reaction was purified by flash column chromatography (PE/Acetone = 10:1) to provide product as pale yellow oil (9 mg, 32%). **^{1}H NMR (400 MHz, CDCl$_3$)**: δ 7.45 – 7.40 (m, 2H), 7.38 – 7.34 (m, 2H), 7.28 – 7.25 (m, 1H), 4.08 (q, *J* = 6.8 Hz, 1H), 3.67 – 3.49 (m, 4H), 2.24 (tt, *J* = 7.9, 4.6 Hz, 2H), 1.37 (d, *J* = 6.8 Hz, 3H), 1.07 – 0.96 (m, 2H), 0.94 – 0.81 (m, 2H); **^{13}C NMR (101 MHz, CDCl$_3$)**: δ 210.31, 143.48, 128.40, 127.62, 127.23, 61.56, 60.75, 18.43, 18.14, 11.07, 11.04; HRMS *m/z* (ESI) calcd for $C_{18}H_{24}NO_2$ $(M + H)^+$, 286.1807, found 286.1805. IR Qmax/cm-1 (film): 702, 899, 1025, 1071, 1383, 1451, 1693, 2974, 3007.

2,2'-((1-phenylethyl)azanediyl)bis(1-(thiophen-2-yl)ethan-1-one) (S37)

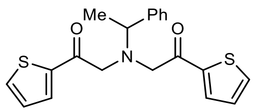

According to general procedure A, the reaction of 1-phenylethan-1-amine (12.3 mg, 0.1 mmol), formaldehyde solution (37% in water) (25 mg, 0.3 mmol), diethyl diethyl 2,6-dimethyl-4-(thiophene-2-carbonyl)-1,4-dihydropyridine-3,5-dicarboxylate (44 mg, 0.12 mmol), 4 Å molecular sieve (200 mg), $BF_3.Et_2O$ (19 uL, 0.15 mmol) in DCM (2 mL). The crude reaction was purified by flash column chromatography (PE/EA = 10:1) to provide product as pale yellow oil (12 mg, 33%). **^{1}H NMR (400 MHz, CDCl$_3$)**: δ 7.77 (dd, *J* = 3.8, 1.1 Hz, 2H), 7.63 (dd, *J* = 5.0, 1.1 Hz, 2H), 7.46 – 7.43 (m, 2H), 7.32 (t, *J* = 7.3 Hz, 2H), 7.27 – 7.23 (m, 1H), 7.10 (dd, *J* = 5.0, 3.8 Hz, 2H), 4.40 (q, *J* = 6.7 Hz, 1H), 4.32 – 4.07 (m, 4H), 1.43 (d, *J* = 6.8 Hz, 3H); **^{13}C NMR (101 MHz, CDCl$_3$)**: δ 191.88, 143.64, 142.29, 133.59, 132.34, 128.46, 128.02, 127.72, 127.30, 60.65, 57.62, 19.42; HRMS

m/z (ESI) calcd for $C_{20}H_{20}NO_2S_2$ $(M + H)^+$, 370.0930, found 370.0919. IR Qmax/cm-1 (film): 700, 729, 1239, 1354, 1409, 1654, 3090.

4-phenyl-2-((1-phenylethyl)amino)-1-(thiophen-2-yl)butan-1-one (S38)

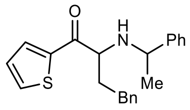

According to general procedure A, the reaction of 1-phenylpropan-2-amine (12.1 mg, 0.1 mmol), hydrocinnamaldehyde (16 uL, 0.12 mmol), diethyl 2,6-dimethyl-4-(thiophene-2-carbonyl)-1,4-dihydropyridine-3,5-dicarboxylate (44 mg, 0.12 mmol), 4 Å molecular sieve (200 mg), $BF_3.Et_2O$ (19 uL, 0.15 mmol) in DCM (2 mL). The crude reaction was purified by flash column chromatography (PE/Acetone = 20:1) to provide product as pale-yellow oil (35 mg, 50%, 1.3 :1 dr). **^{1}H NMR (400 MHz, CDCl$_3$)**: δ 7.65 – 7.60 (m, 1H), 7.37 – 7.31 (m, 5H), 7.20 – 7.15 (m, 3H), 7.01 – 6.96 (m, 2H), 6.95 – 6.91 (m, 2H), 3.78 (dd, *J* = 8.9, 3.6 Hz, 1H), 3.64 (q, *J* = 6.6 Hz, 1H), 2.85 (ddd, *J* = 13.6, 8.0, 5.2 Hz, 1H), 2.64 (dt, *J* = 13.5, 8.1 Hz, 1H), 2.39 (s, 2H), 1.94 – 1.73 (m, 2H), 1.41 (d, *J* = 6.5 Hz, 3H); **^{13}C NMR (101 MHz, CDCl$_3$)**: δ 197.28, 145.15, 142.87, 141.46, 134.18, 132.17, 128.62, 128.42, 128.26, 128.11, 127.37, 127.16, 125.82, 60.53, 56.84, 36.58, 32.26, 25.10; HRMS *m/z* (ESI) calcd for $C_{22}H_{24}NOS$ $(M + H)^+$, 350.1579, found 350.1580. IR Qmax/cm-1 (film): 700, 726, 758, 811, 1029, 1240, 1272, 1353, 1412, 1452, 1495, 1658, 2924, 2959, 3025 and **^{1}H NMR (400 MHz, CDCl$_3$)**: δ 7.63 (d, *J* = 4.9 Hz, 1H), 7.37 – 7.28 (m, 6H), 7.25 – 7.19 (m, 5H), 7.04 (t, *J* = 4.4 Hz, 1H), 4.04 (dd, *J* = 7.9, 4.7 Hz, 1H), 3.81 (q, *J* = 6.5 Hz, 1H), 2.84-2.79 (m, 2H), 2.12 – 2.02 (m, 2H), 1.93-1.84 (m, 1H), 1.37 (d, *J* = 6.5 Hz, 3H); **^{13}C NMR (101 MHz, CDCl$_3$)**: δ 195.99, 145.40, 142.86, 141.50, 133.98, 131.94, 128.67, 128.39, 128.33, 127.99, 127.07, 126.93, 126.02, 60.13, 56.11, 35.73, 32.04, 22.73; HRMS *m/z* (ESI) calcd for $C_{22}H_{24}NOS$ $(M + H)^+$, 350.1579, found 350.1580. IR Qmax/cm-1 (film): 699, 726, 750, 1243, 1372, 1411, 1453, 1496, 1649, 2926, 3026.

2-(Diethylamino)-4,4,4-trifluoro-1-phenylbutan-1-one (S39)

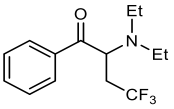

According to general procedure A, the reaction of diethylamine(7.3 mg, 0.1 mmol), 3,3,3-trifluoropropanal (33.6 mg, 0.3 mmol), diethyl 4-benzoyl-2,6-dimethyl-1,4-dihydropyridine-3,5-dicarboxylate (44 mg, 0.12 mmol), 4 Å molecular sieve (200 mg), TBSOTf (28 uL, 0.12 mmol) in DCM (2 mL). The crude reaction was purified by flash column chromatography (PE/Et_2O = 5:1) to provide product as yellow oil (16 mg, 59%). **^{1}H NMR (400 MHz, CDCl$_3$)**: δ 8.08 (dd, *J* = 8.3, 1.4 Hz, 2H), 7.63 – 7.55 (m, 1H), 7.48 (dd, J = 8.4, 7.0 Hz, 2H), 4.61 (dd, J = 8.8, 2.4 Hz, 1H), 3.23 – 3.04 (m, 1H), 2.77 – 2.56 (m, 1H), 2.50-2.39 (m, 2H), 2.37-2.27 (m, 1H), 1.05 (t, *J* = 7.1 Hz, 6H); **^{13}C NMR (101 MHz, CDCl$_3$)**: δ 196.62, 136.58, 132.98, 128.85, 128.38, 126.86 (q, *J* = 275.1 Hz), 58.04, 44.41, 27.97 (q, *J* = 27.4 Hz), 13.86; **^{19}F NMR (376 MHz, CDCl3)**: δ -64.97; HRMS *m/z* (ESI) calcd for $C_{14}H_{19}NOF_3$ $(M + H)^+$, 274.1413, found 274.1420. IR Qmax/cm-1 (film): 691, 752, 1106, 1143, 1162, 1220, 1255, 1330, 1385, 1448, 1689, 2935, 2973.

tert-butyl 4-(4-(methylthio)-1-oxo-1-phenylbutan-2-yl)piperazine-1-carboxylate (S40)

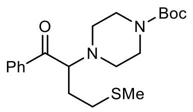

According to general procedure A, the reaction of tert-butyl piperazine-1-carboxylate (18.6 mg, 0.1 mmol), 3-(methylthio)propanal (12.5 mg, 0.12 mmol), diethyl 4-benzoyl-2,6-dimethyl-1,4-dihydropyridine-3,5-dicarboxylate (44 mg, 0.12 mmol), 4 Å molecular sieve (200 mg), TBSOTf (28 uL, 0.12 mmol) in DCM (2 mL). The crude reaction was purified by flash column chromatography (PE/EA = 5:1) to provide product as pale yellow oil (13 mg, 34%). **^{1}H NMR (400 MHz, $CDCl_3$)**: δ 8.07 – 7.98 (m, 2H), 7.66 – 7.56 (m, 1H), 7.51-7.46 (m, 2H), 4.39 (dd, *J* = 8.4, 5.3 Hz, 1H), 3.36 (t, *J* = 5.0 Hz, 4H), 2.71 – 2.56 (m, 5H), 2.44 (dt, *J* = 13.1, 7.1 Hz, 1H), 2.28 – 2.15 (m, 1H), 2.10 (s, 3H), 2.00-1.91 (m, 1H), 1.45 (s, 9H); **^{13}C NMR (101 MHz, $CDCl_3$)**: δ 198.98, 154.63, 137.20, 133.14, 128.56, 79.61 , 65.86, 49.28, 31.23, 28.37, 24.83, 15.42; HRMS *m/z* (ESI) calcd for $C_{20}H_{31}N_2O_3S$ (M + H)$^+$, 379.2055, found 379.2053. IR Qmax/cm-1 (film): 1002, 1118, 1170, 1247, 1278, 1365, 1421, 1447, 1690, 2917, 2973.

4-phenyl-2-(piperidin-1-yl)-1-(p-tolyl)butan-1-one (S41)

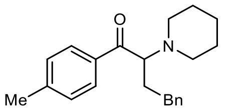

According to general procedure A, the reaction of piperidine (10 uL, 0.1 mmol), hydrocinnamaldehyde (16 uL, 0.12 mmol), diethyl 2,6-dimethyl-4-(4-methylbenzoyl)-1,4-dihydropyridine-3,5-dicarboxylate (45 mg, 0.12 mmol), 4 Å molecular sieve (200 mg), TBSOTf (28 uL, 0.12 mmol) in DCM (2 mL). The crude reaction was purified by flash column chromatography (PE/EA = 10:1) to provide product as pale-yellow oil (22 mg, 69%). **^{1}H NMR (400 MHz, $CDCl_3$)**: δ 7.92 (d, *J* = 8.0 Hz, 2H), 7.33 – 7.24 (m, 4H), 7.24 – 7.17 (m, 3H), 4.04 (dd, *J* = 8.8, 5.1 Hz, 1H), 2.72-2.63 (m, 1H), 2.61-2.49 (m, 5H), 2.44 (s, 3H), 2.28-2.16 (m, 1H), 2.06-1.97 (m, 1H), 1.55-1.49 (m, 4H), 1.42-1.37 (m, 2H); **^{13}C NMR (101 MHz, $CDCl_3$)**: δ 199.68, 143.52, 142.02, 135.18, 129.07, 128.70, 128.45, 128.29, 125.79, 67.46, 50.79, 32.74, 27.96, 26.62, 24.55, 21.62.; HRMS *m/z* (ESI) calcd for $C_{22}H_{28}NO$ (M + H)$^+$, 322.2171, found 322.2170. IR Qmax/cm-1 (film): 699, 751, 1114, 1180, 1202, 1231, 1441, 1452, 1605, 1677, 2804, 2852, 2931.

***N*-benzyl-2-(4-(methylthio)phenyl)-2-(piperidin-1-yl)acetamide (S42)**

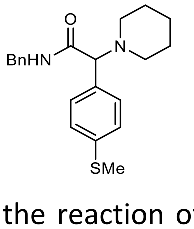

According to general procedure A, the reaction of piperidine (10 uL, 0.1 mmol), 4-(methylthio)benzaldehyde (16 uL, 0.12 mmol), diethyl 4-(benzylcarbamoyl)-2,6-dimethyl-1,4-dihydropyridine-3,5-dicarboxylate (46 mg, 0.12 mmol), 4 Å molecular

sieve (200 mg), TBSOTf (28 uL, 0.12 mmol) in DCM (2 mL). The crude reaction was purified by flash column chromatography (PE/Acetone = 5:1) to provide product as yellow oil (14 mg, 40%).

^{1}H NMR (400 MHz, $CDCl_3$): δ 7.56 (s, 1H), 7.39-7.32 (m, 2H), 7.34-7.29 (m, 1H), 7.29-7.24 (m, 2H), 7.23 (s, 4H), 4.50 (qd, J = 14.9, 6.0 Hz, 2H), 3.88 (s, 1H), 2.50 (s, 3H), 2.37 (t, J = 5.6 Hz, 4H), 1.54 (p, J = 5.6 Hz, 4H), 1.42 (d, J = 6.3 Hz, 2H); **^{13}C NMR (101 MHz, $CDCl_3$)**: δ 171.74, 138.50, 138.16, 132.72, 129.48, 128.67, 127.65, 127.41, 126.47, 75.87, 52.73, 43.20, 26.18, 24.16, 15.80; HRMS *m/z* (ESI) calcd for $C_{21}H_{26}N_2OS$ $(M + H)^+$, 355. 1839, found 355.1843. IR Qmax/cm-1 (film): 699, 1439, 1453, 1493, 1514, 1655, 2804, 2850, 2931, 3290, 3304.

^1H and ^{13}C NMR Spectra Data

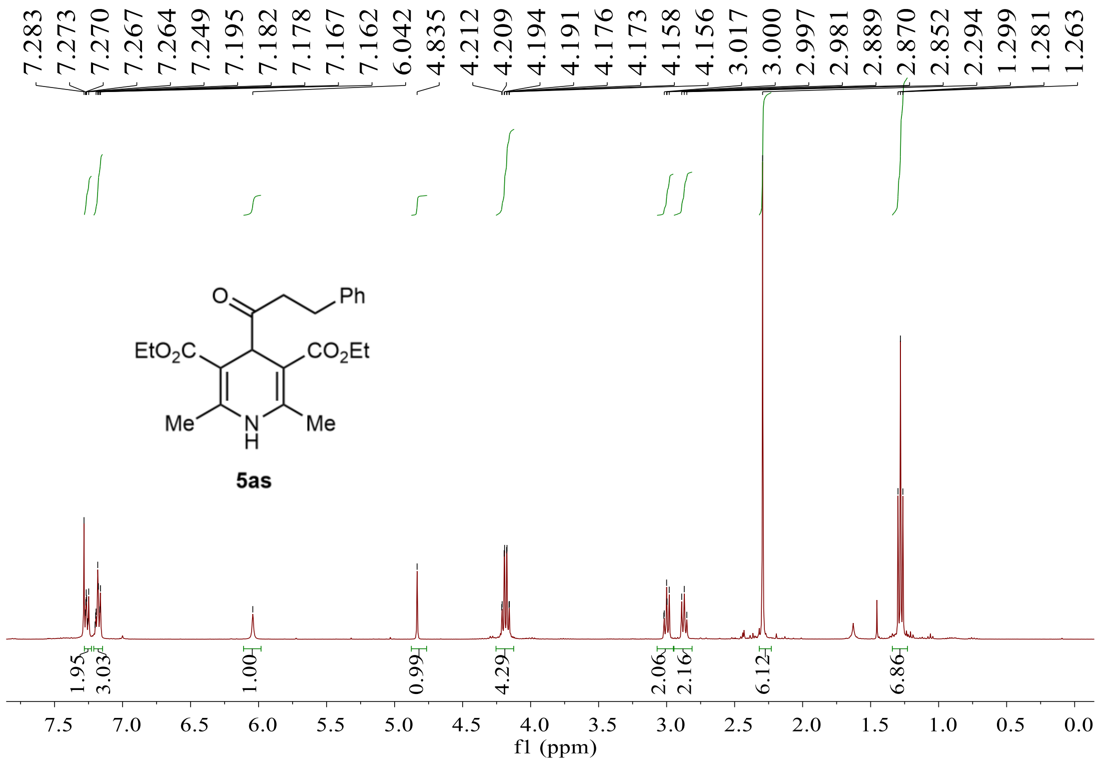

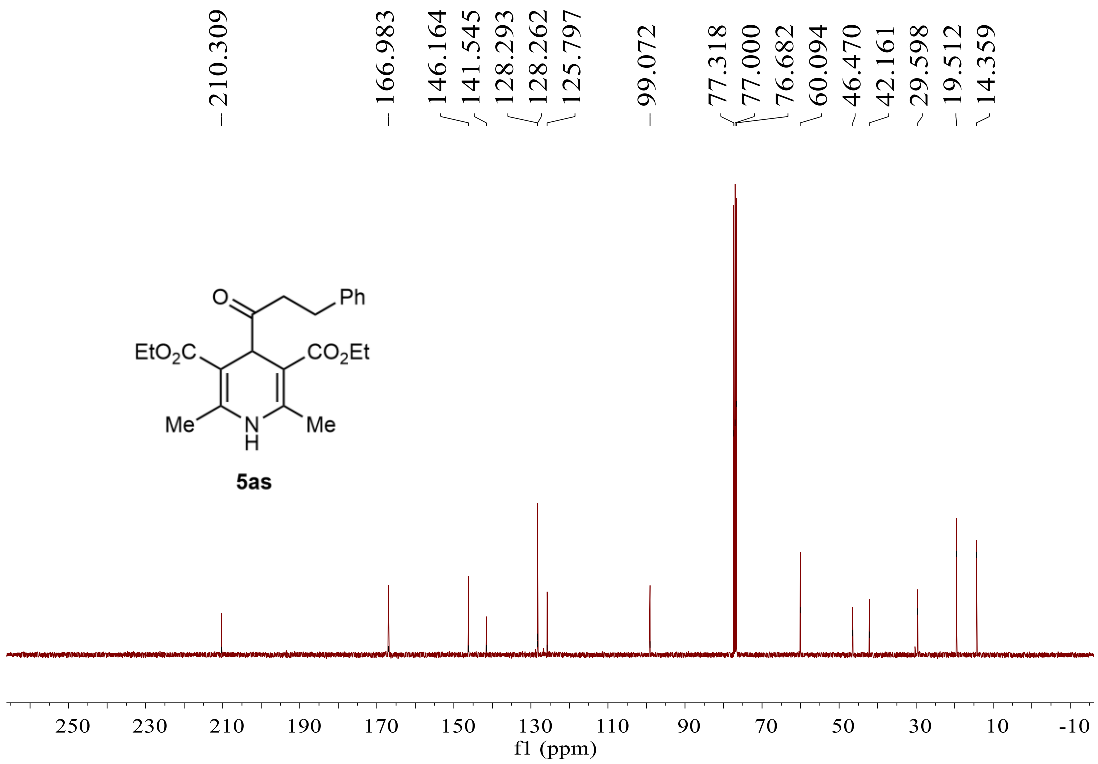

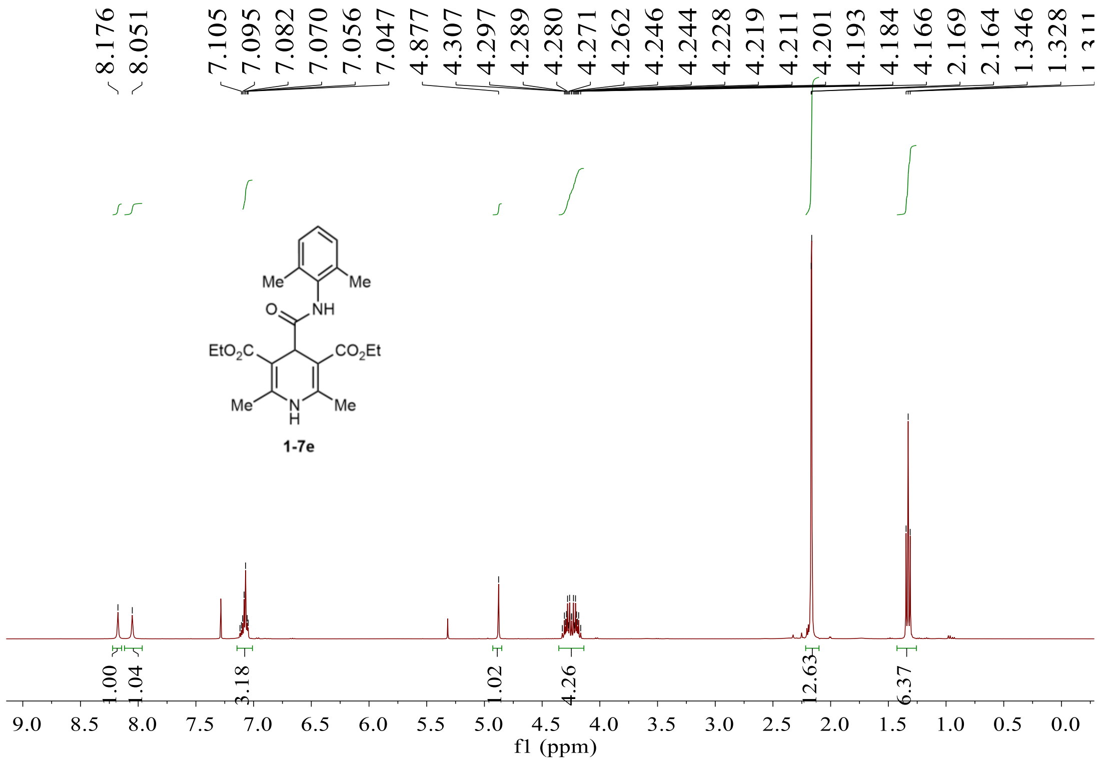
8.176
8.051
7.105
7.095
7.082
7.070
7.056
7.047
4.877
4.307
4.297
4.289
4.280
4.271
4.262
4.246
4.244
4.228
4.219
4.211
4.201
4.193
4.184
4.166
2.169
2.164
1.346
1.328
Me
Me
O
NH
EtO2C
CO2Et
Me
N
H
Me
1-7e
1.00
1.04
3.18
1.02
4.26
12.63
6.37
f1 (ppm)

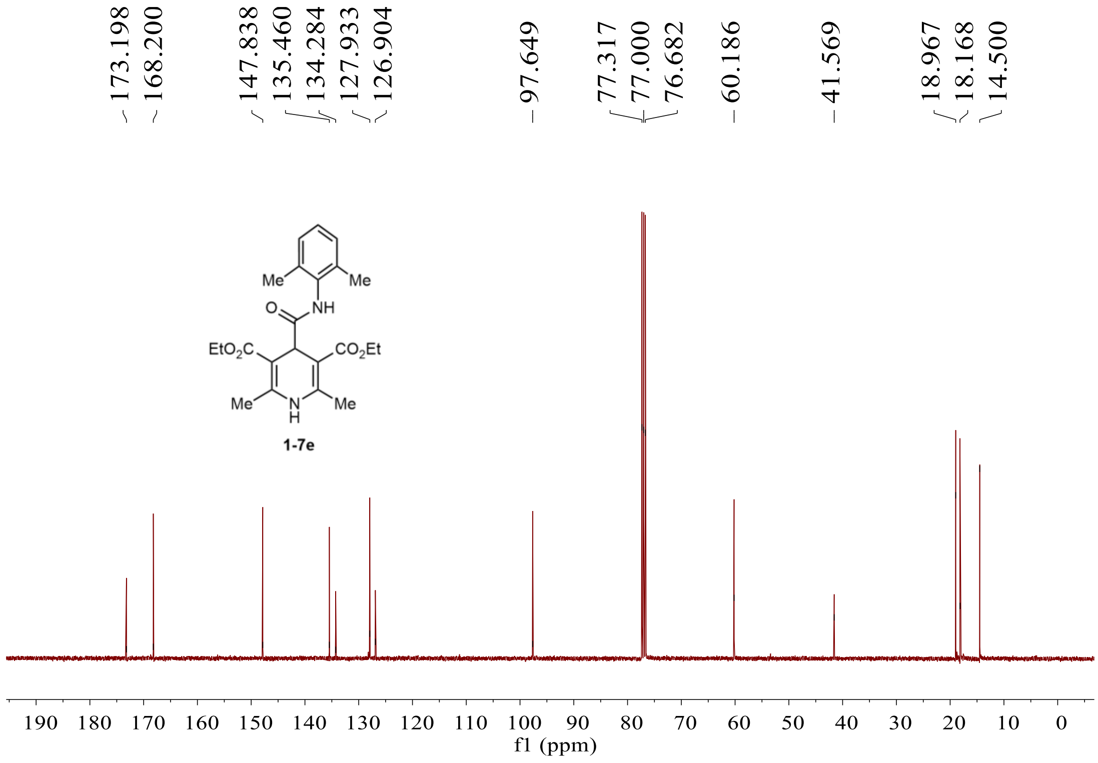
173.198
168.200
147.838
135.460
134.284
127.933
126.904
97.649
77.317
77.000
76.682
60.186
41.569
18.967
18.168
14.500
Me
Me
O
NH
EtO2C
CO2Et
Me
N
H
Me
1-7e
f1 (ppm)

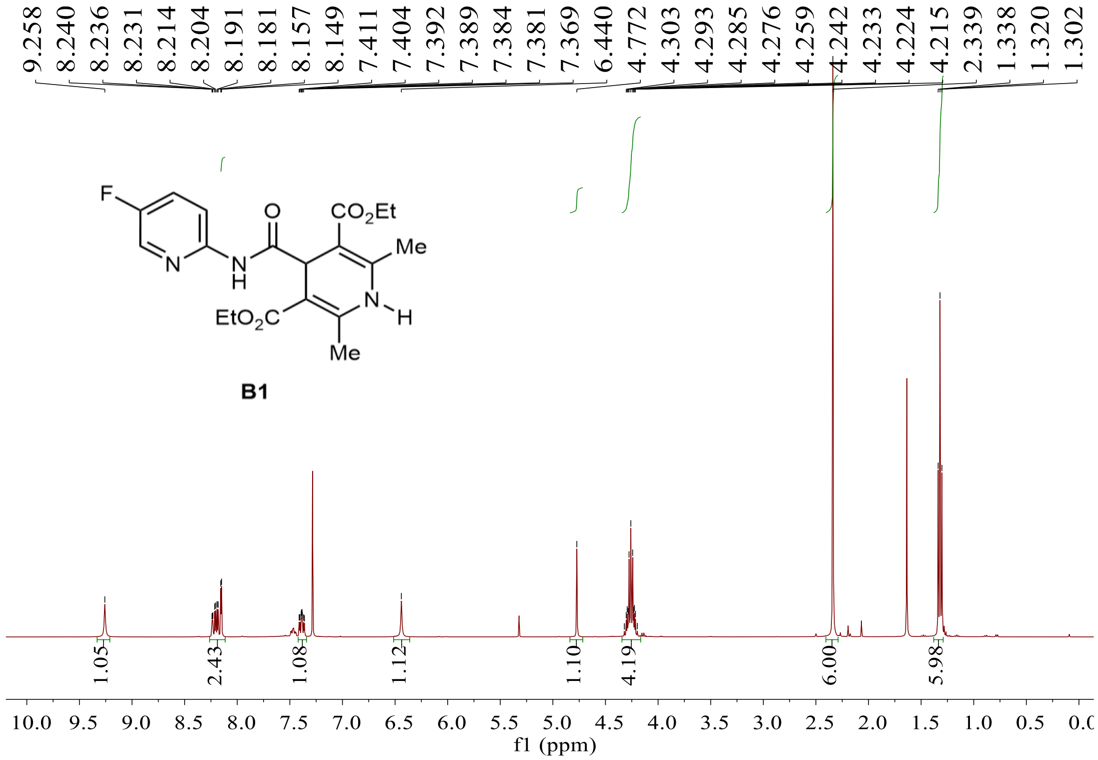
9.258
8.240
8.236
8.231
8.214
8.204
8.191
8.181
8.157
8.149
7.411
7.404
7.392
7.389
7.384
7.381
7.369
6.440
4.772
4.303
4.293
4.285
4.276
4.259
4.242
4.233
4.224
4.215
2.339
1.338
1.320
1.302
F
N
O
N
H
CO2Et
Me
N
H
EtO2C
Me
B1
1.05
2.43
1.08
1.12
1.10
4.19
6.00
5.98
10.0 9.5 9.0 8.5 8.0 7.5 7.0 6.5 6.0 5.5 5.0 4.5 4.0 3.5 3.0 2.5 2.0 1.5 1.0 0.5 0.0
f1 (ppm)

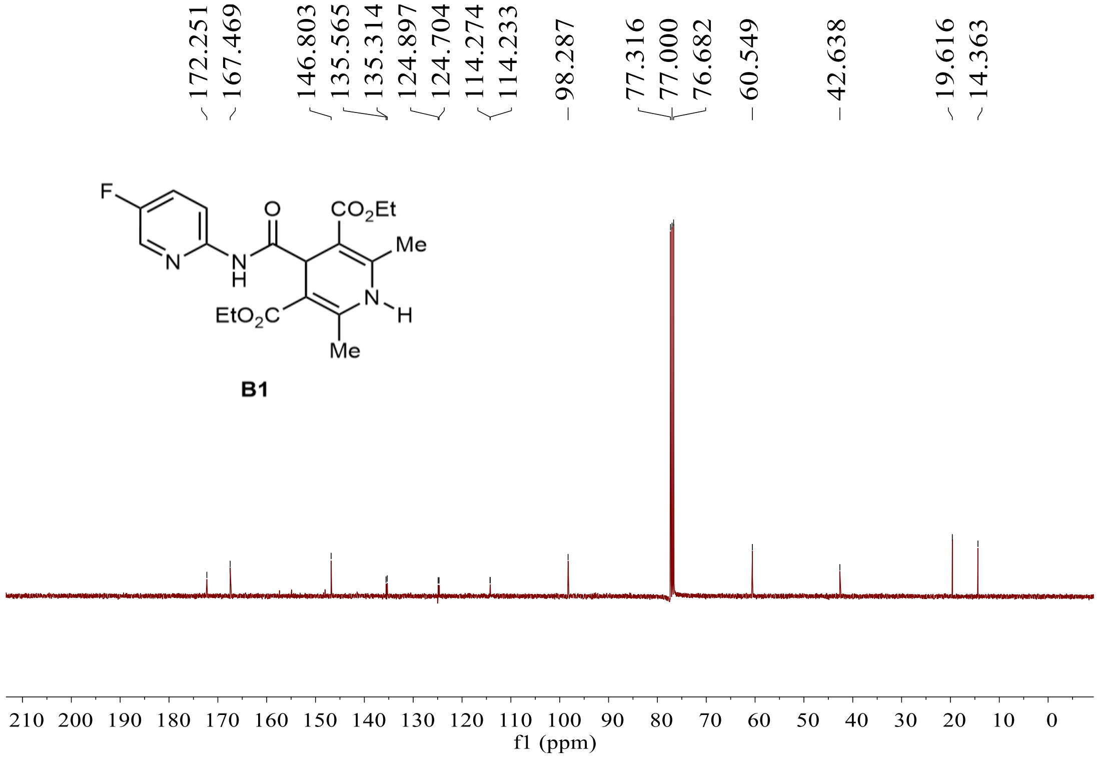
172.251
167.469
146.803
135.565
135.314
124.897
124.704
114.274
114.233
98.287
77.316
77.000
76.682
60.549
42.638
19.616
14.363
F
N
O
N
H
CO2Et
Me
N
H
EtO2C
Me
B1
210 200 190 180 170 160 150 140 130 120 110 100 90 80 70 60 50 40 30 20 10 0
f1 (ppm)

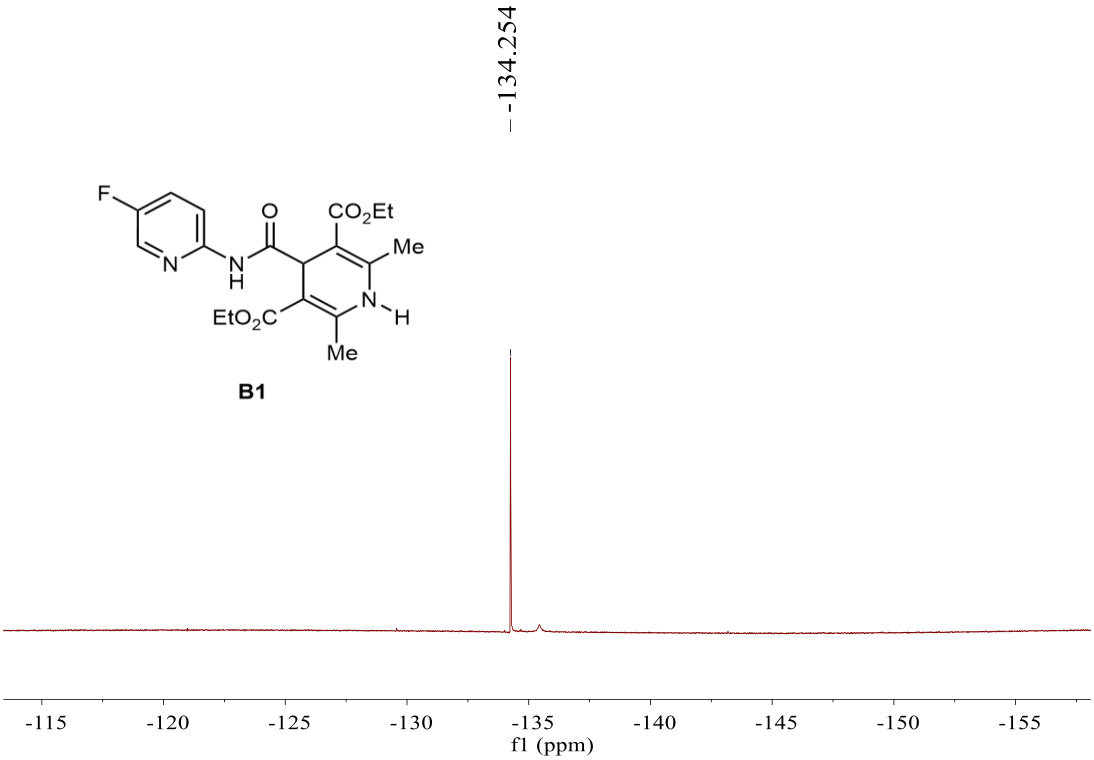

-134.254
F
O
CO2Et
Me
N
H
N
N
H
EtO2C
Me
B1
-115
-120
-125
-130
-135
-140
-145
-150
-155
f1 (ppm)

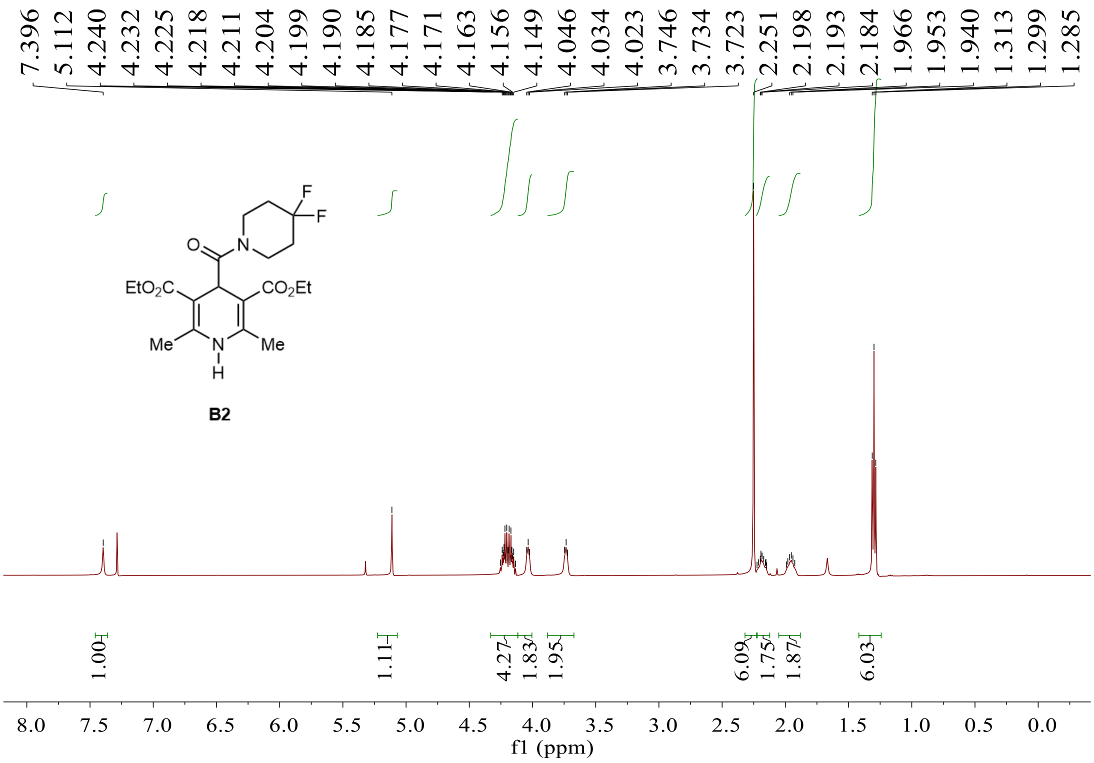

7.396
5.112
4.240
4.232
4.225
4.218
4.211
4.204
4.199
4.190
4.185
4.177
4.171
4.163
4.156
4.149
4.046
4.034
4.023
3.746
3.734
3.723
2.251
2.198
2.193
2.184
1.966
1.953
1.940
1.313
1.299
1.285
F
F
O
N
EtO2C
CO2Et
Me
N
H
Me
B2
1.00
1.11
4.27
1.83
1.95
6.09
1.75
1.87
6.03
8.0
7.5
7.0
6.5
6.0
5.5
5.0
4.5
4.0
3.5
3.0
2.5
2.0
1.5
1.0
0.5
0.0
f1 (ppm)

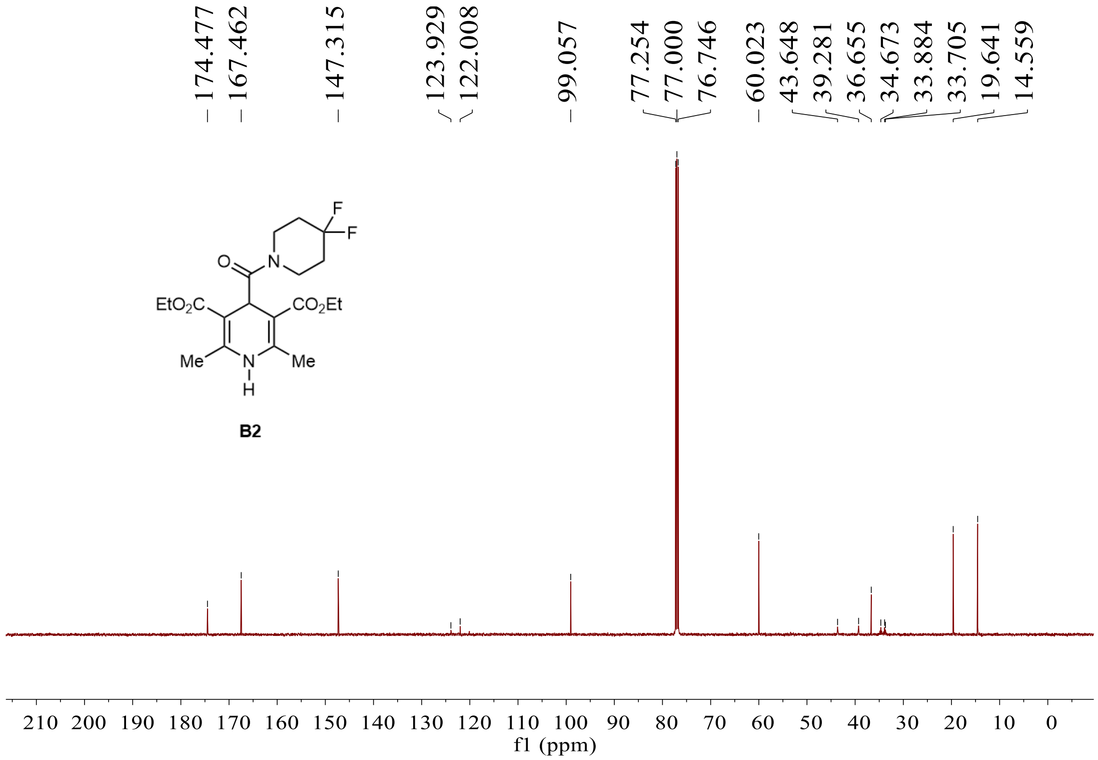
174.477
167.462
147.315
123.929
122.008
99.057
77.254
77.000
76.746
60.023
43.648
39.281
36.655
34.673
33.884
33.705
19.641
14.559
F
F
O
N
EtO2C
CO2Et
Me
N
H
Me
B2
210 200 190 180 170 160 150 140 130 120 110 100 90 80 70 60 50 40 30 20 10 0
f1 (ppm)

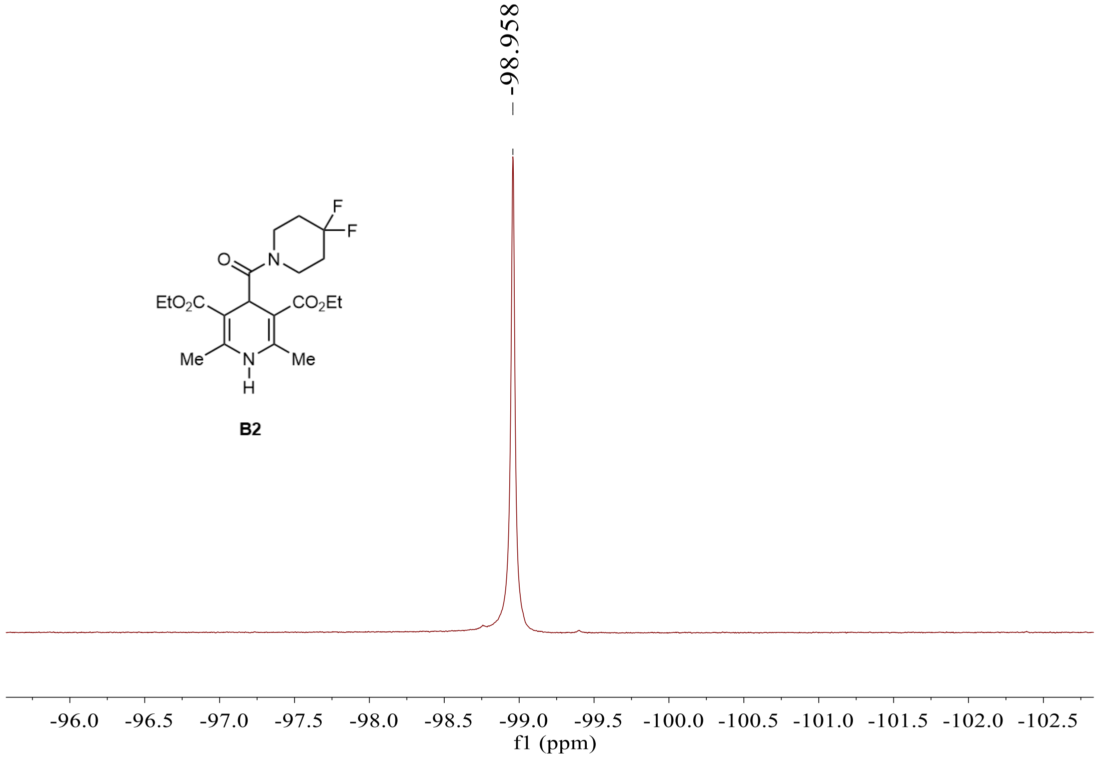
-98.958
F
F
O
N
EtO2C
CO2Et
Me
N
H
Me
B2
-96.0 -96.5 -97.0 -97.5 -98.0 -98.5 -99.0 -99.5 -100.0 -100.5 -101.0 -101.5 -102.0 -102.5
f1 (ppm)

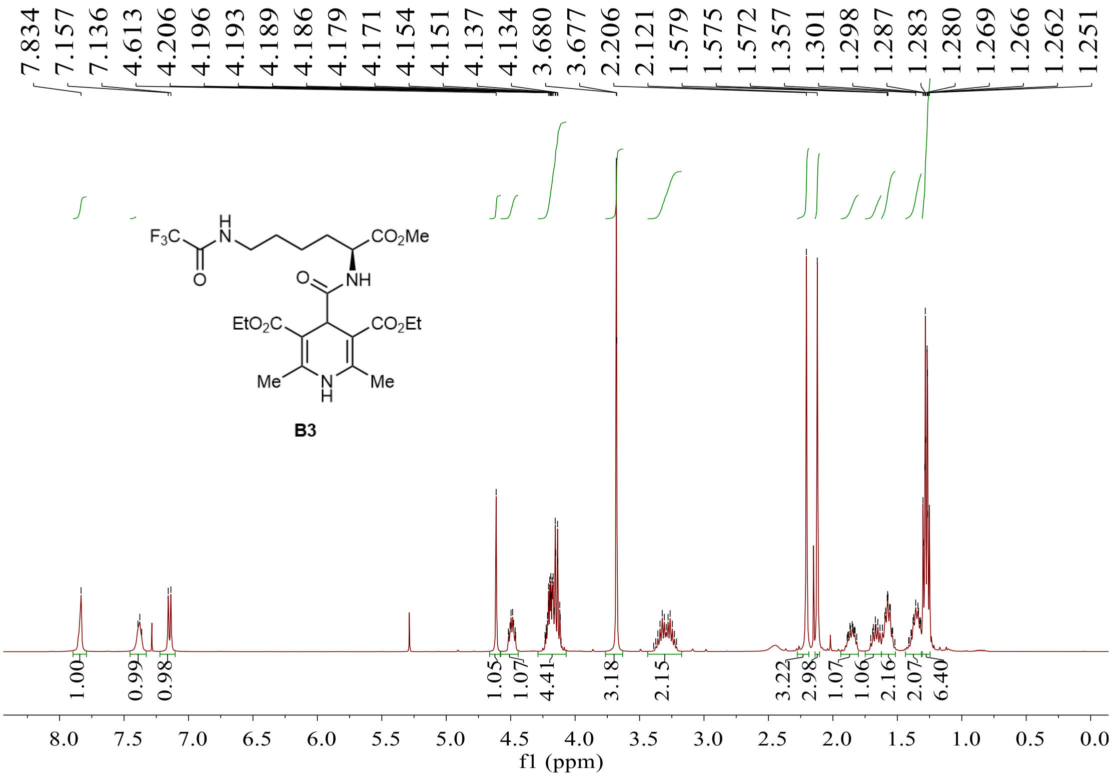
7.834
7.157
7.136
4.613
4.206
4.196
4.193
4.189
4.186
4.179
4.171
4.154
4.151
4.137
4.134
3.680
3.677
2.206
2.121
1.579
1.575
1.572
1.357
1.301
1.298
1.287
1.283
1.280
1.269
1.266
1.262
1.251
F3C
H
N
O
CO2Me
O
NH
EtO2C
CO2Et
Me
N
H
Me
B3
1.00
0.99
0.98
1.05
1.07
4.41
3.18
2.15
3.22
2.98
1.07
1.06
2.16
2.07
6.40
8.0 7.5 7.0 6.5 6.0 5.5 5.0 4.5 4.0 3.5 3.0 2.5 2.0 1.5 1.0 0.5 0.0
f1 (ppm)

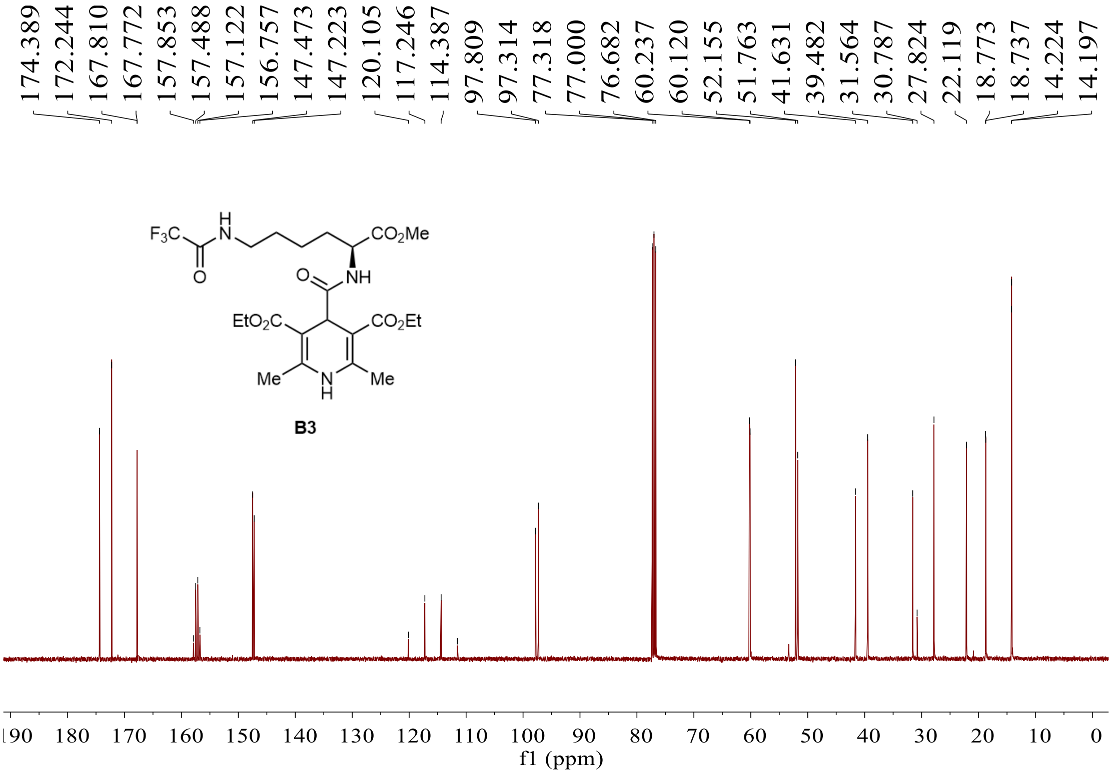
174.389
172.244
167.810
167.772
157.853
157.488
157.122
156.757
147.473
147.223
120.105
117.246
114.387
97.809
97.314
77.318
77.000
76.682
60.237
60.120
52.155
51.763
41.631
39.482
31.564
30.787
27.824
22.119
18.773
18.737
14.224
14.197
F3C
H
N
O
CO2Me
O
NH
EtO2C
CO2Et
Me
N
H
Me
B3
190 180 170 160 150 140 130 120 110 100 90 80 70 60 50 40 30 20 10 0
f1 (ppm)

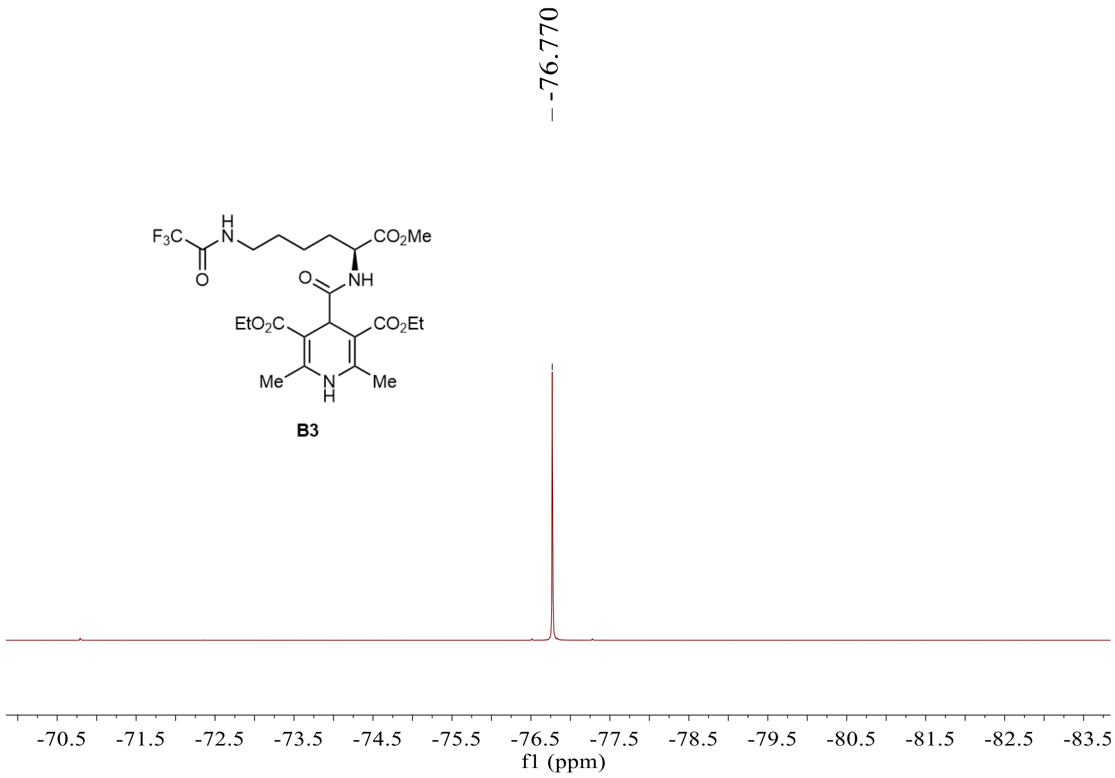

-76.770
F3C
H
N
CO2Me
O
O
NH
EtO2C
CO2Et
Me
N
H
Me
B3
-70.5 -71.5 -72.5 -73.5 -74.5 -75.5 -76.5 -77.5 -78.5 -79.5 -80.5 -81.5 -82.5 -83.5
f1 (ppm)

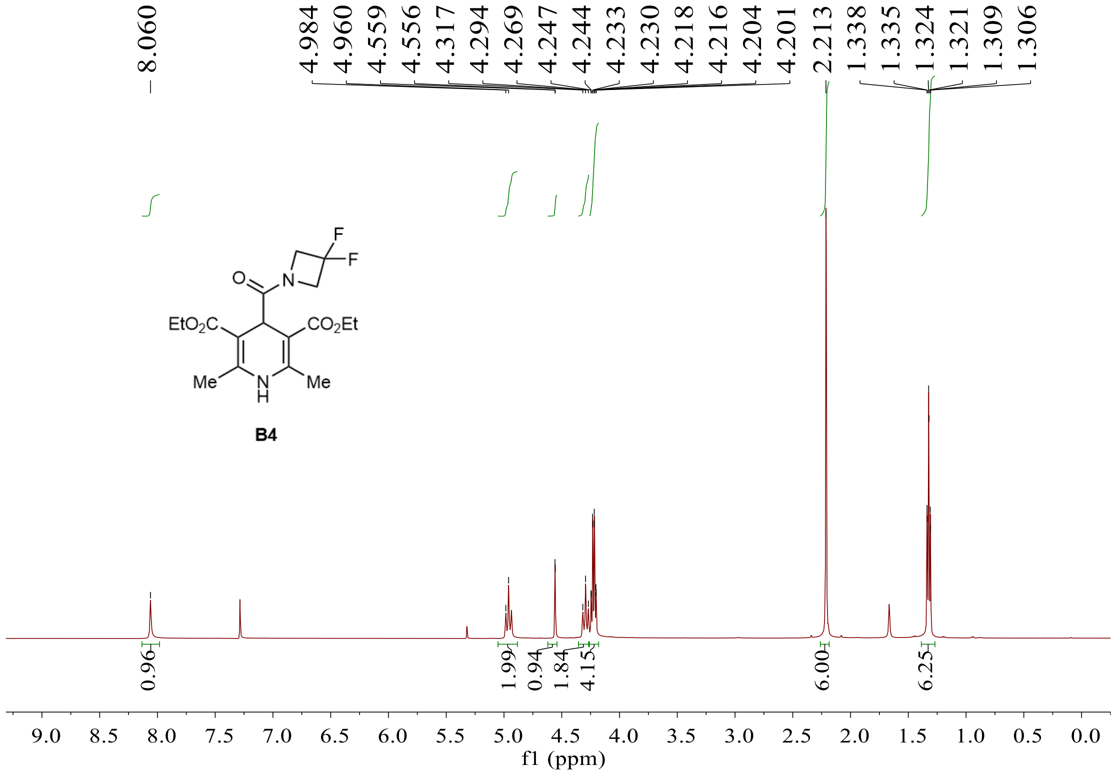

8.060
4.984 4.960 4.559 4.556 4.317 4.294 4.269 4.247 4.244 4.233 4.230 4.218 4.216 4.204 4.201
2.213
1.338 1.335 1.324 1.321 1.309 1.306
F
F
O
N
EtO2C
CO2Et
Me
N
H
Me
B4
0.96
1.99
0.94
1.84
4.15
6.00
6.25
9.0 8.5 8.0 7.5 7.0 6.5 6.0 5.5 5.0 4.5 4.0 3.5 3.0 2.5 2.0 1.5 1.0 0.5 0.0
f1 (ppm)

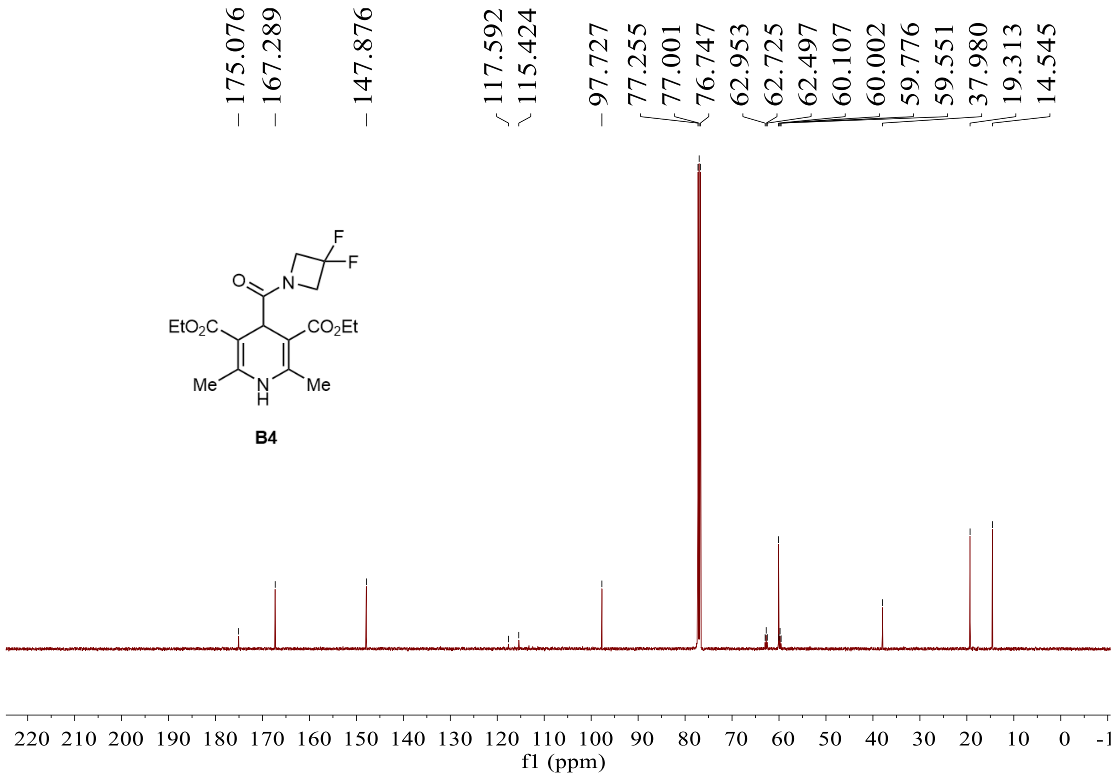
175.076
167.289
147.876
117.592
115.424
97.727
77.255
77.001
76.747
62.953
62.725
62.497
60.107
60.002
59.776
59.551
37.980
19.313
14.545
EtO2C
CO2Et
Me
Me
N
H
O
N
F
F
B4
220 210 200 190 180 170 160 150 140 130 120 110 100 90 80 70 60 50 40 30 20 10 0
f1 (ppm)

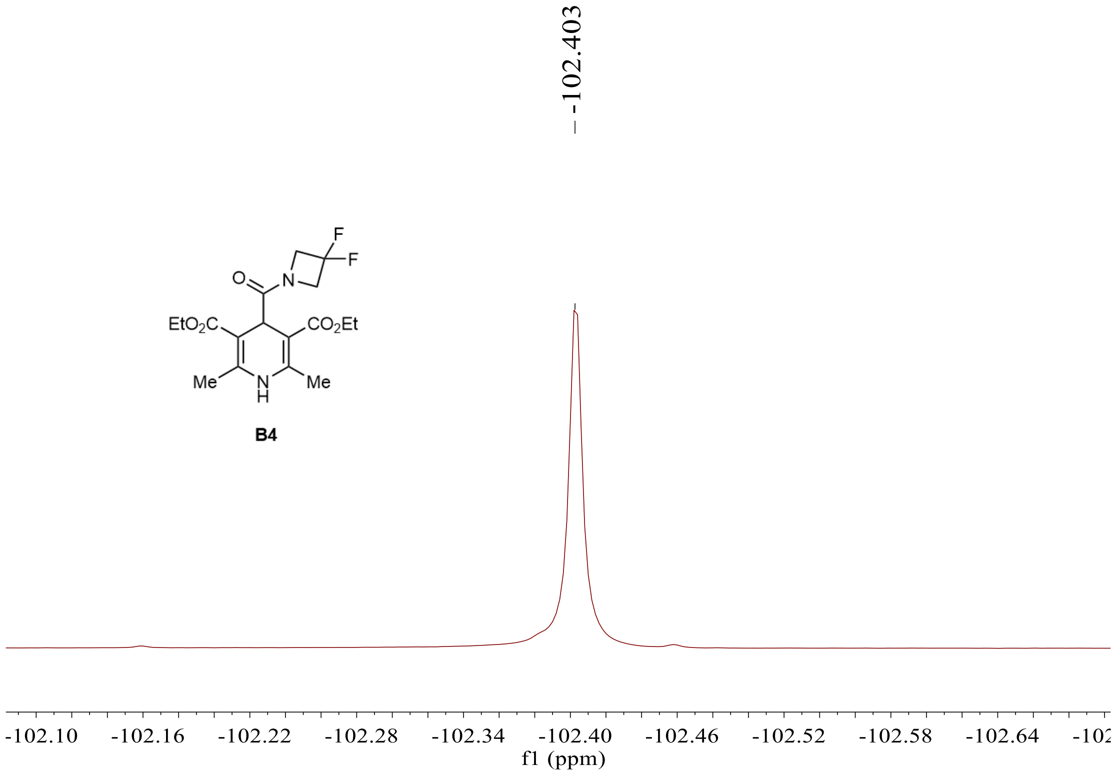
-102.403
EtO2C
CO2Et
Me
Me
N
H
O
N
F
F
B4
-102.10 -102.16 -102.22 -102.28 -102.34 -102.40 -102.46 -102.52 -102.58 -102.64
f1 (ppm)

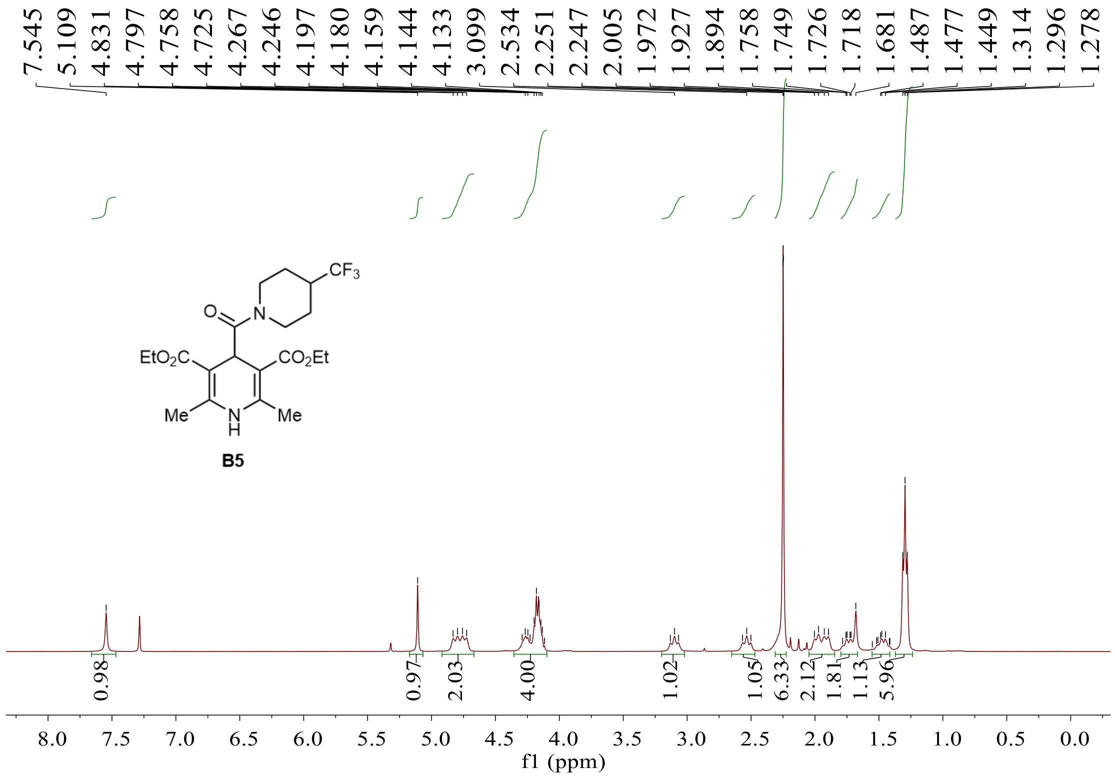

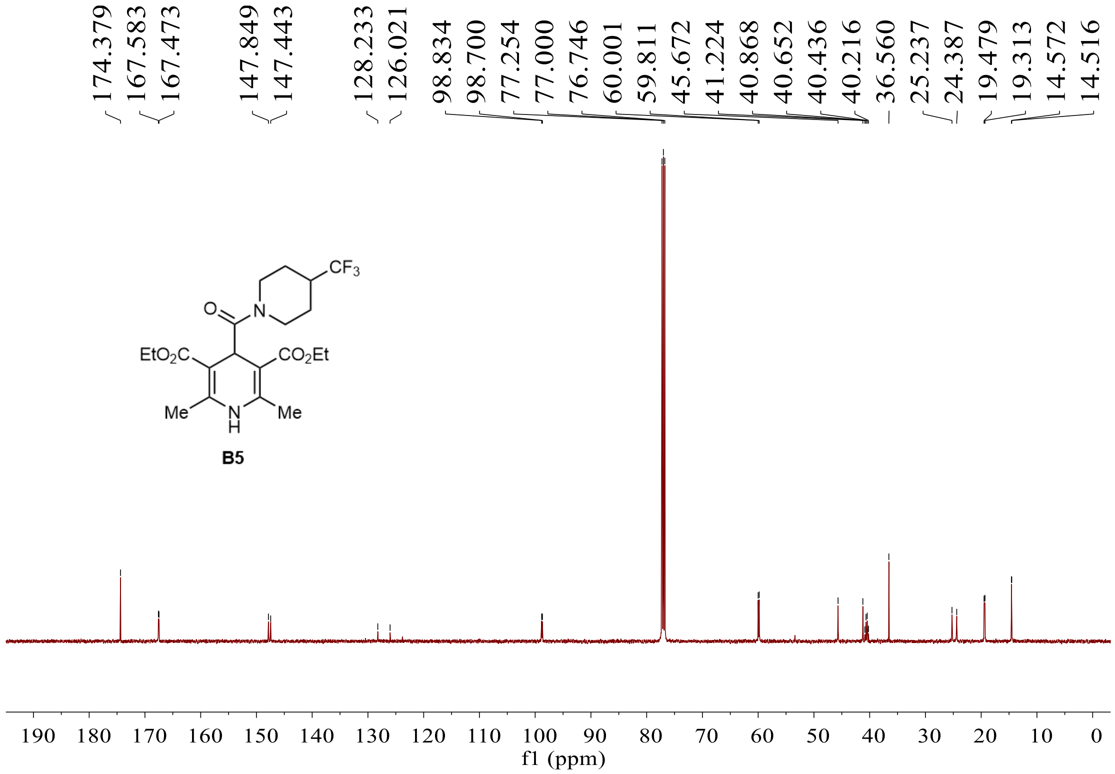

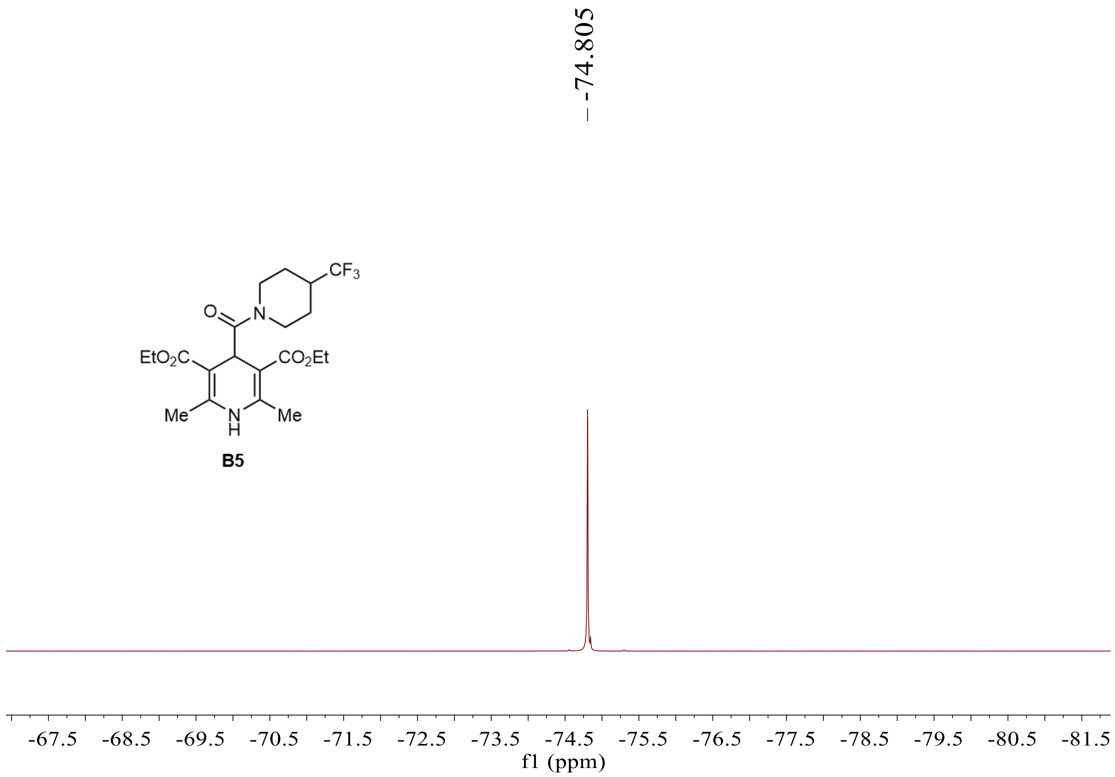
-74.805
CF3
O
N
EtO2C
CO2Et
Me
N
H
Me
B5
-67.5 -68.5 -69.5 -70.5 -71.5 -72.5 -73.5 -74.5 -75.5 -76.5 -77.5 -78.5 -79.5 -80.5 -81.5
f1 (ppm)

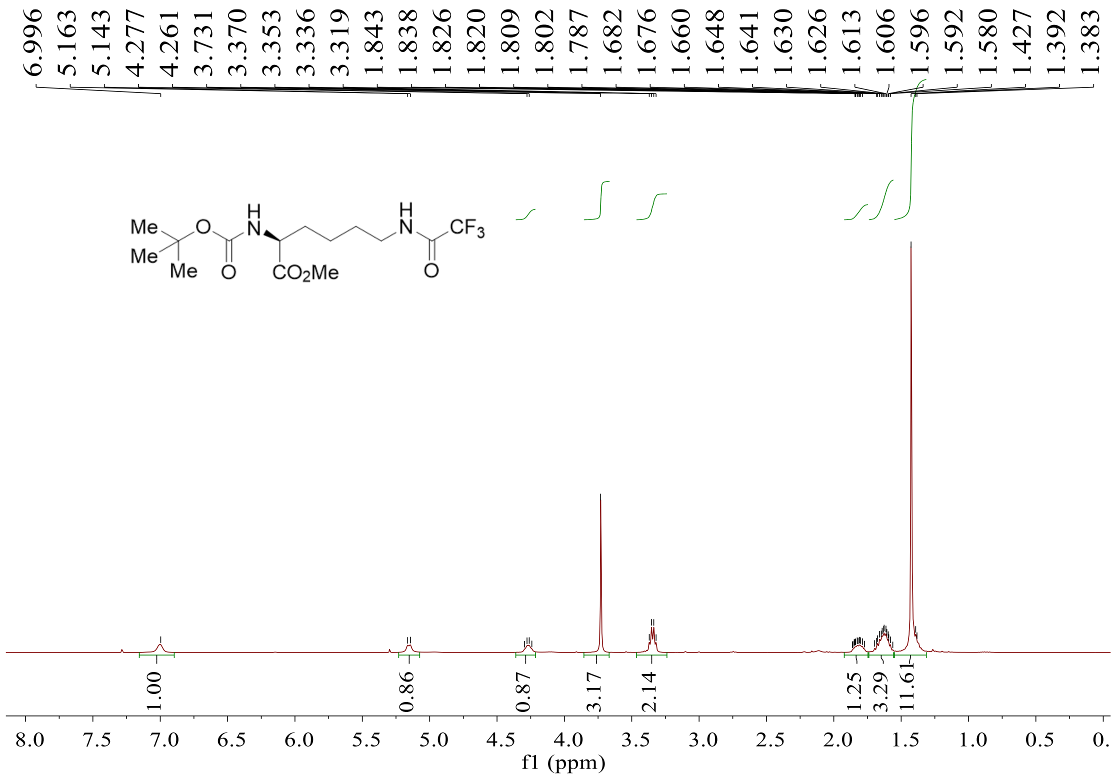
6.996 5.163 5.143 4.277 4.261 3.731 3.370 3.353 3.336 3.319 1.843 1.838 1.826 1.820 1.809 1.802 1.787 1.682 1.676 1.660 1.648 1.641 1.630 1.626 1.613 1.606 1.596 1.592 1.580 1.427 1.392 1.383
Me
Me
Me
O
H
N
O
CO2Me
H
N
CF3
O
1.00
0.86
0.87
3.17
2.14
1.25
3.29
11.61
8.0 7.5 7.0 6.5 6.0 5.5 5.0 4.5 4.0 3.5 3.0 2.5 2.0 1.5 1.0 0.5 0.
f1 (ppm)

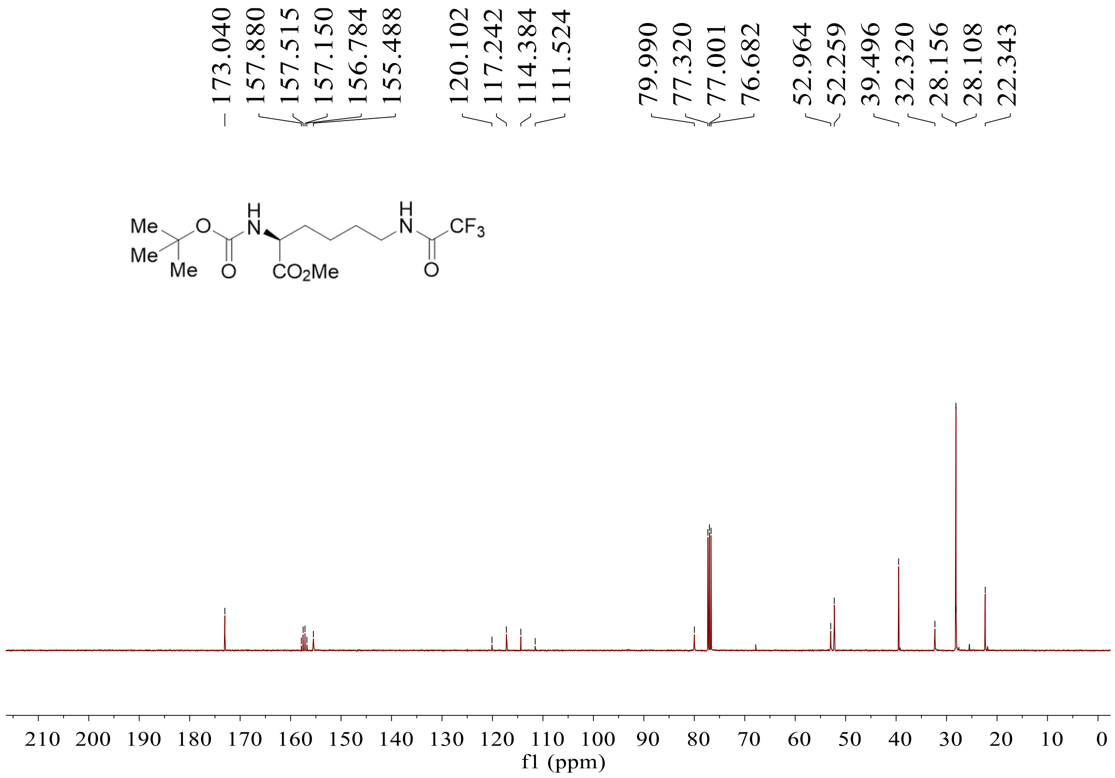
173.040
157.880
157.515
157.150
156.784
155.488
120.102
117.242
114.384
111.524
79.990
77.320
77.001
76.682
52.964
52.259
39.496
32.320
28.156
28.108
22.343
Me
Me
Me
O
H
N
O
CO2Me
H
N
CF3
O
210 200 190 180 170 160 150 140 130 120 110 100 90 80 70 60 50 40 30 20 10 0
f1 (ppm)

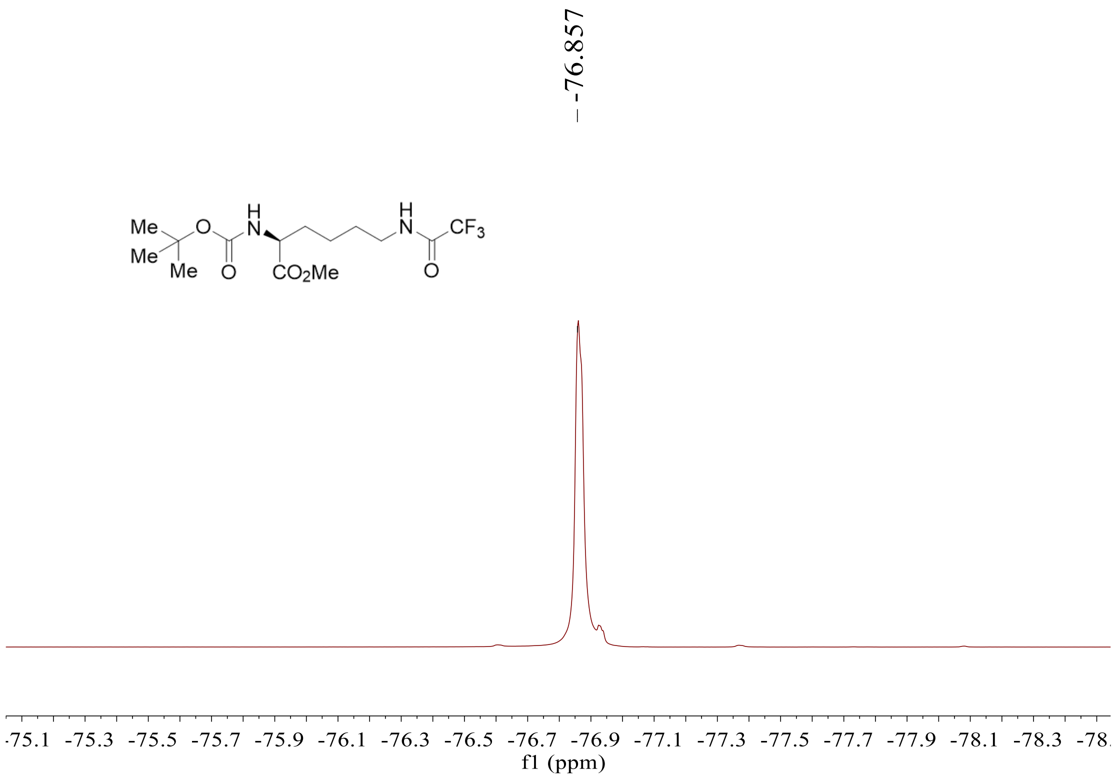
-76.857
Me
Me
Me
O
H
N
O
CO2Me
H
N
CF3
O
-75.1 -75.3 -75.5 -75.7 -75.9 -76.1 -76.3 -76.5 -76.7 -76.9 -77.1 -77.3 -77.5 -77.7 -77.9 -78.1 -78.3
f1 (ppm)

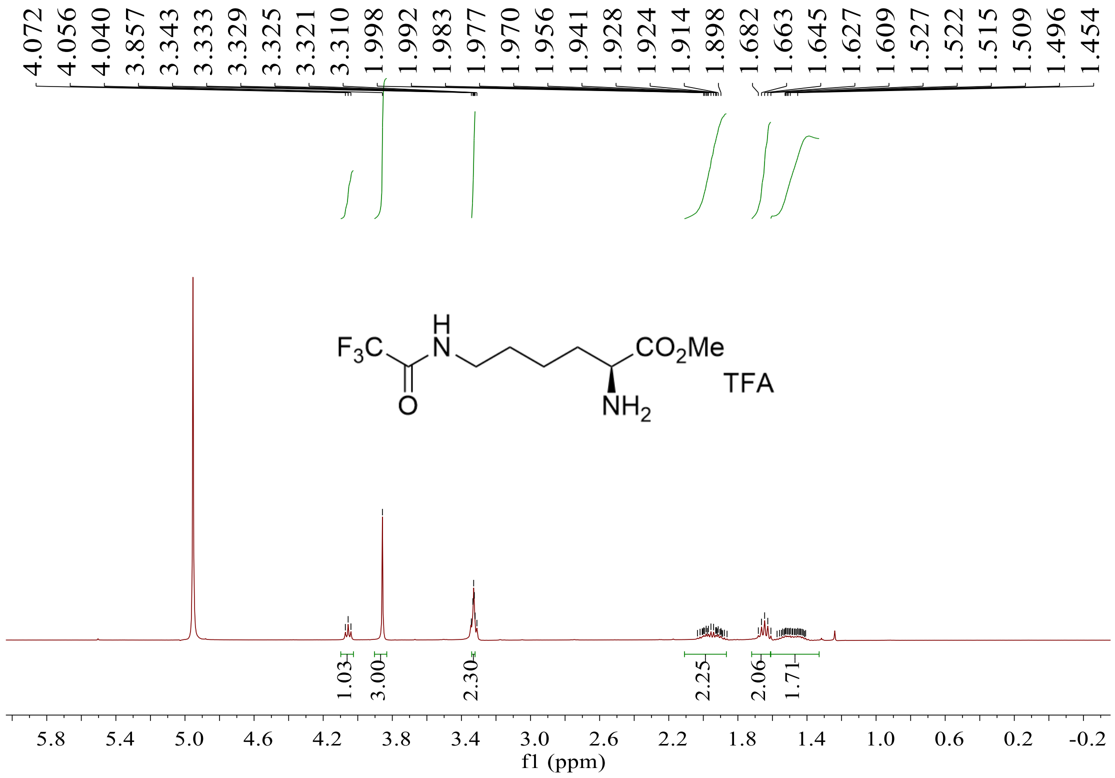
4.072
4.056
4.040
3.857
3.343
3.333
3.329
3.325
3.321
3.310
1.998
1.992
1.983
1.977
1.970
1.956
1.941
1.928
1.924
1.914
1.898
1.682
1.663
1.645
1.627
1.609
1.527
1.522
1.515
1.509
1.496
1.454
F3C
H
N
O
CO2Me
NH2
TFA
1.03
3.00
2.30
2.25
2.06
1.71
5.8 5.4 5.0 4.6 4.2 3.8 3.4 3.0 2.6 2.2 1.8 1.4 1.0 0.6 0.2 -0.2
f1 (ppm)

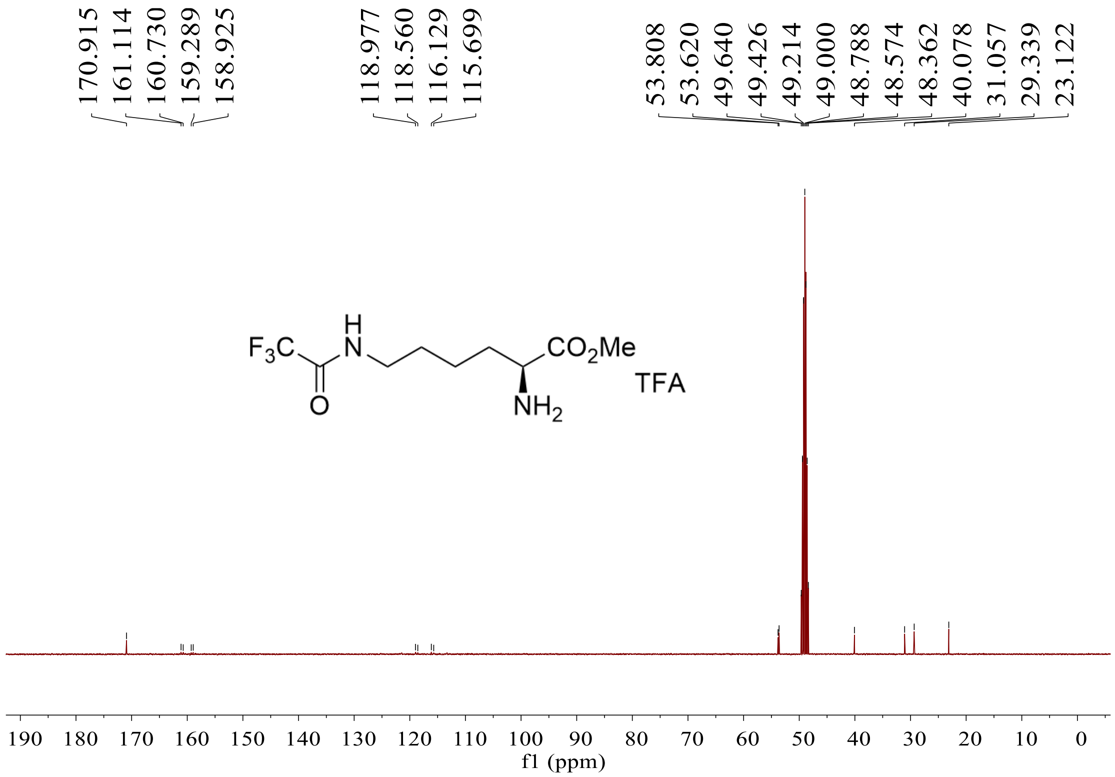
170.915
161.114
160.730
159.289
158.925
118.977
118.560
116.129
115.699
53.808
53.620
49.640
49.426
49.214
49.000
48.788
48.574
48.362
40.078
31.057
29.339
23.122
F3C
H
N
O
CO2Me
NH2
TFA
190 180 170 160 150 140 130 120 110 100 90 80 70 60 50 40 30 20 10 0
f1 (ppm)

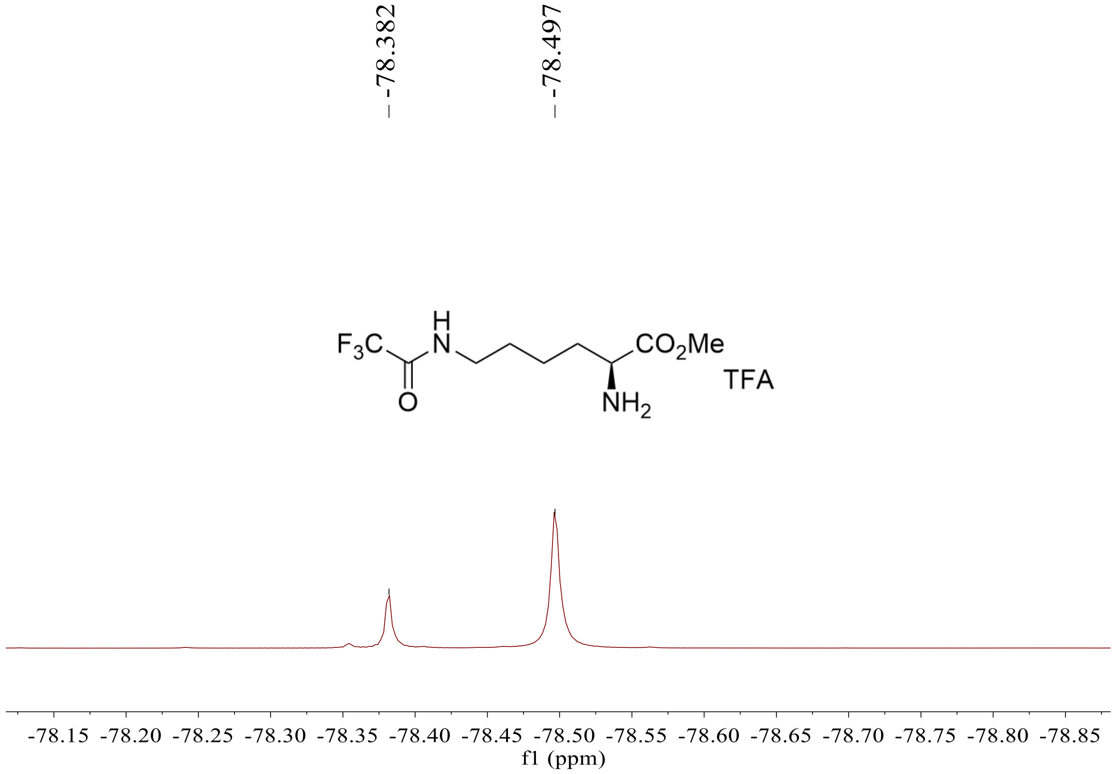
-78.382
-78.497
F3C
H
N
O
CO2Me
NH2
TFA
-78.15 -78.20 -78.25 -78.30 -78.35 -78.40 -78.45 -78.50 -78.55 -78.60 -78.65 -78.70 -78.75 -78.80 -78.85
f1 (ppm)

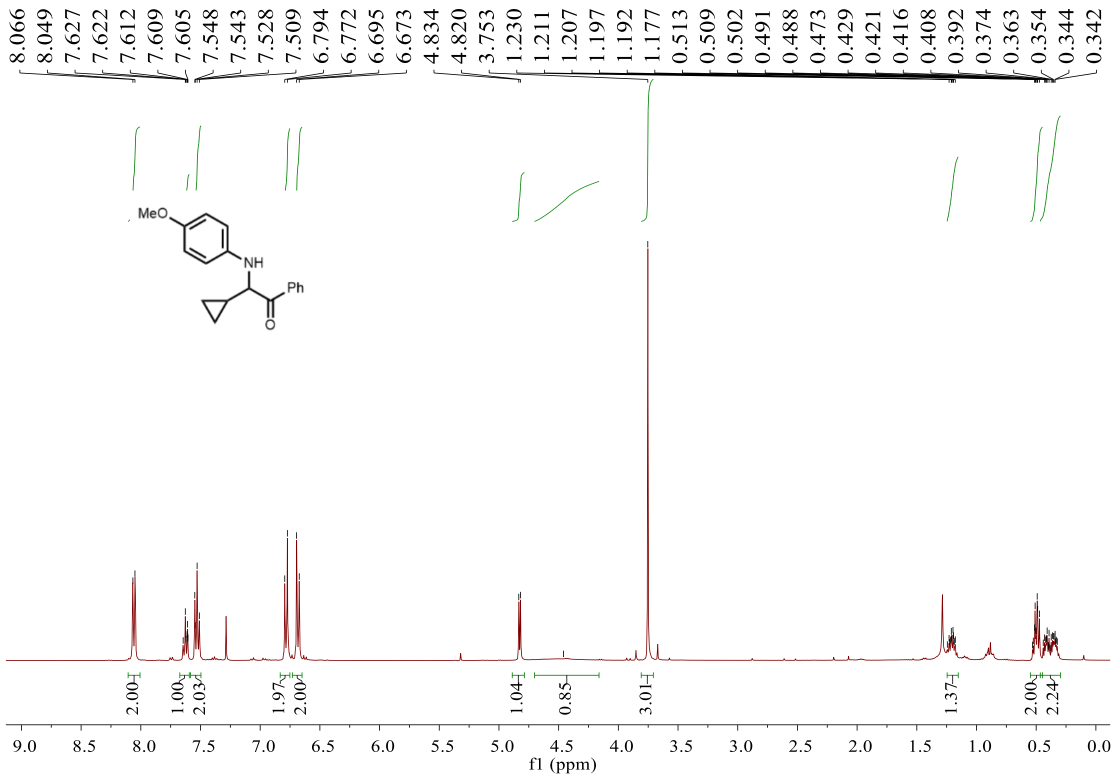
8.066 8.049 7.627 7.622 7.612 7.609 7.605 7.548 7.543 7.528 7.509 6.794 6.772 6.695 6.673 4.834 4.820 3.753 1.230 1.211 1.207 1.197 1.192 1.177 0.513 0.509 0.502 0.491 0.488 0.473 0.429 0.421 0.416 0.408 0.392 0.374 0.363 0.354 0.344 0.342
MeO
NH
Ph
O
2.00 1.00 2.03 1.97 2.00 1.04 0.85 3.01 1.37 2.00 2.24
f1 (ppm)

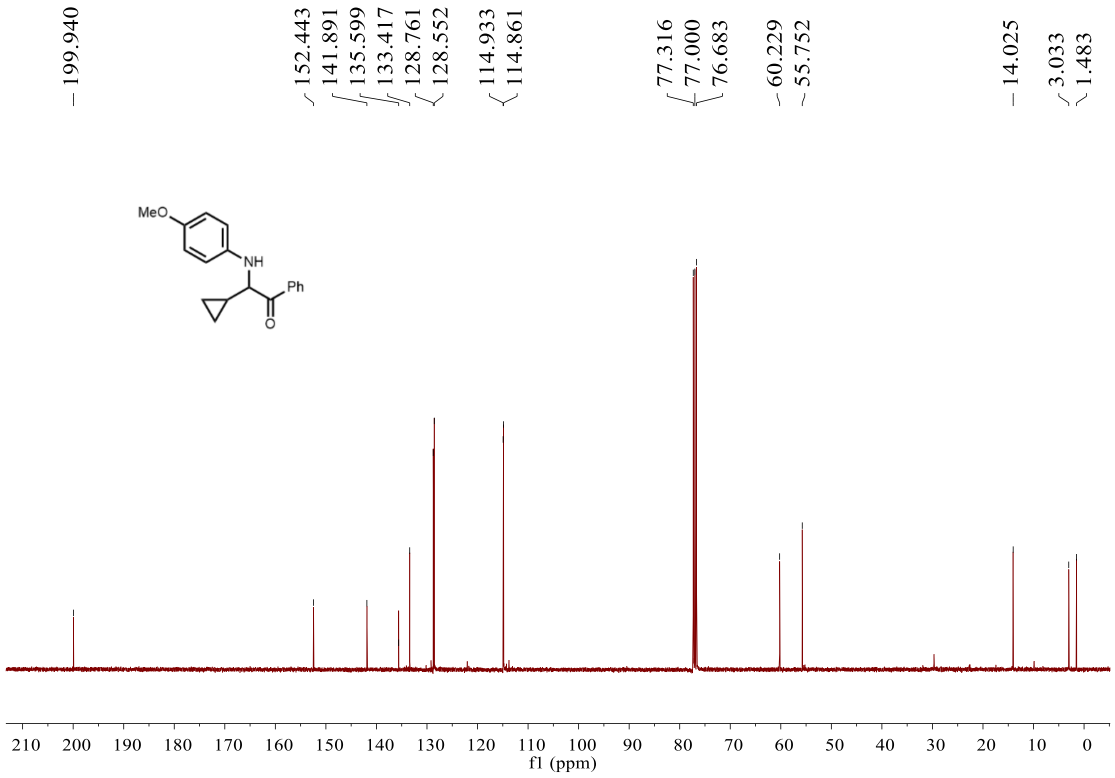
199.940
152.443 141.891 135.599 133.417 128.761 128.552 114.933 114.861
77.316 77.000 76.683 60.229 55.752
14.025 3.033 1.483
MeO
NH
Ph
O
f1 (ppm)

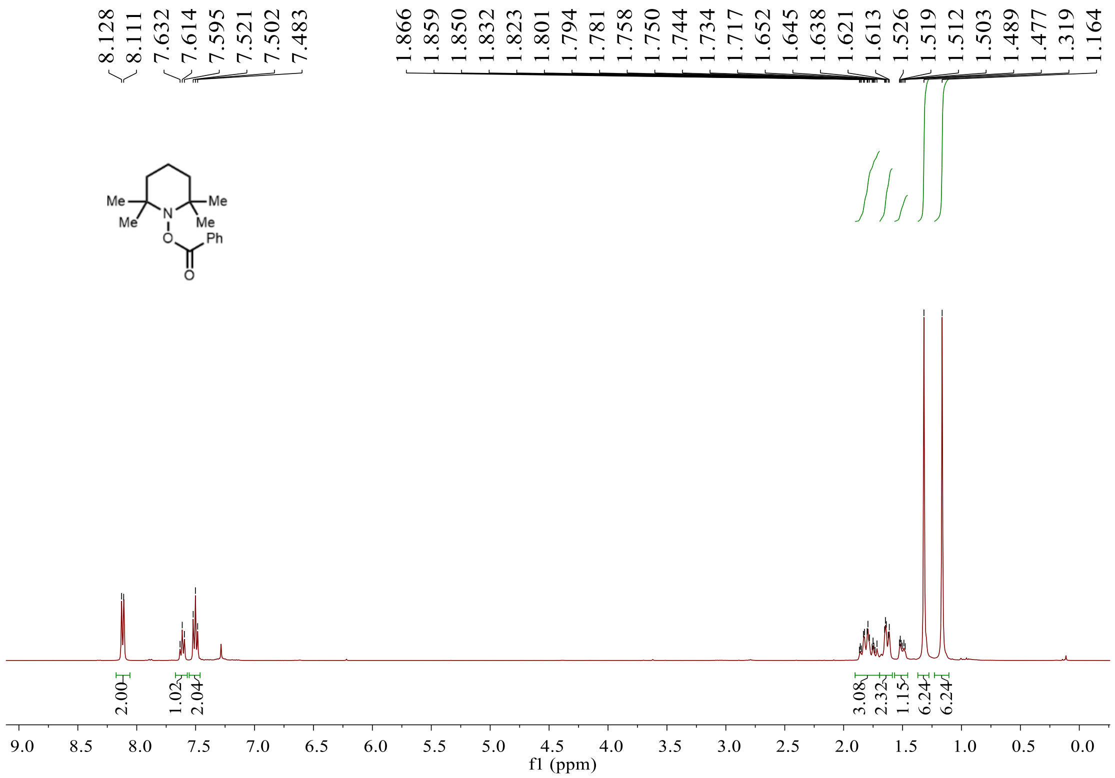
8.128
8.111
7.632
7.614
7.595
7.521
7.502
7.483
1.866
1.859
1.850
1.832
1.823
1.801
1.794
1.781
1.758
1.750
1.744
1.734
1.717
1.652
1.645
1.638
1.621
1.613
1.526
1.519
1.512
1.503
1.489
1.477
1.319
1.164
Me
Me
Me
Me
N
O
Ph
O
2.00
1.02
2.04
3.08
2.32
1.15
6.24
6.24
9.0 8.5 8.0 7.5 7.0 6.5 6.0 5.5 5.0 4.5 4.0 3.5 3.0 2.5 2.0 1.5 1.0 0.5 0.0
f1 (ppm)

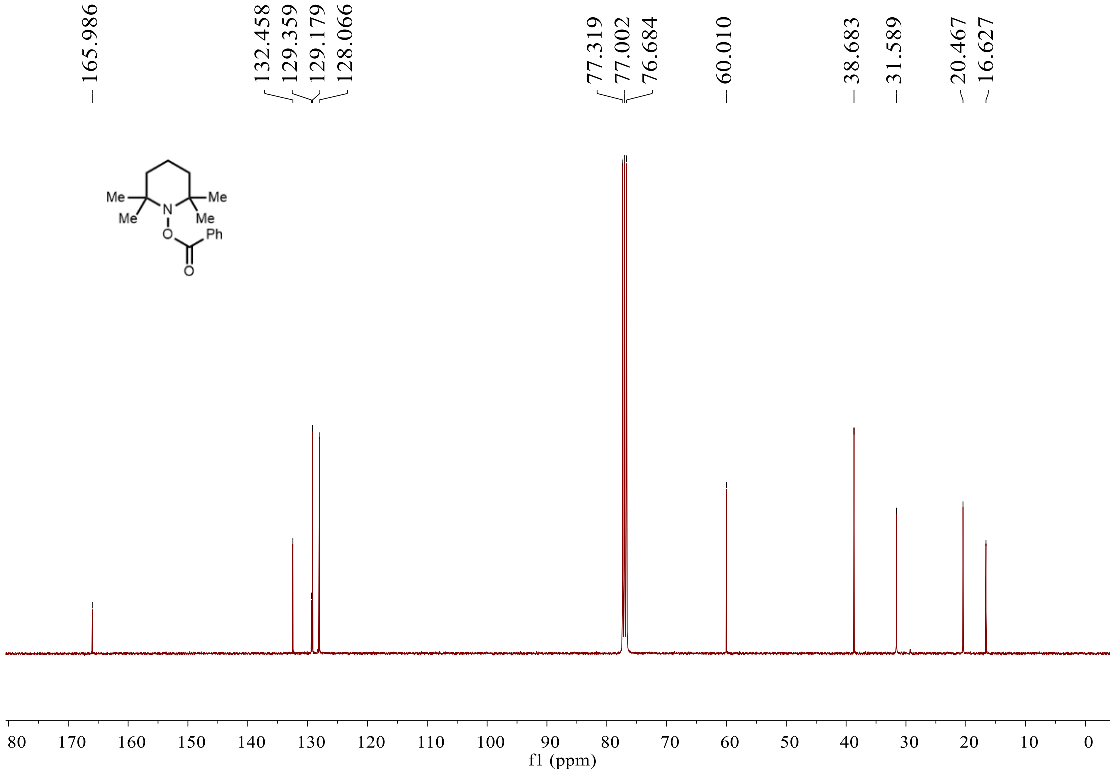
165.986
132.458
129.359
129.179
128.066
77.319
77.002
76.684
60.010
38.683
31.589
20.467
16.627
Me
Me
Me
Me
N
O
Ph
O
80 170 160 150 140 130 120 110 100 90 80 70 60 50 40 30 20 10 0
f1 (ppm)

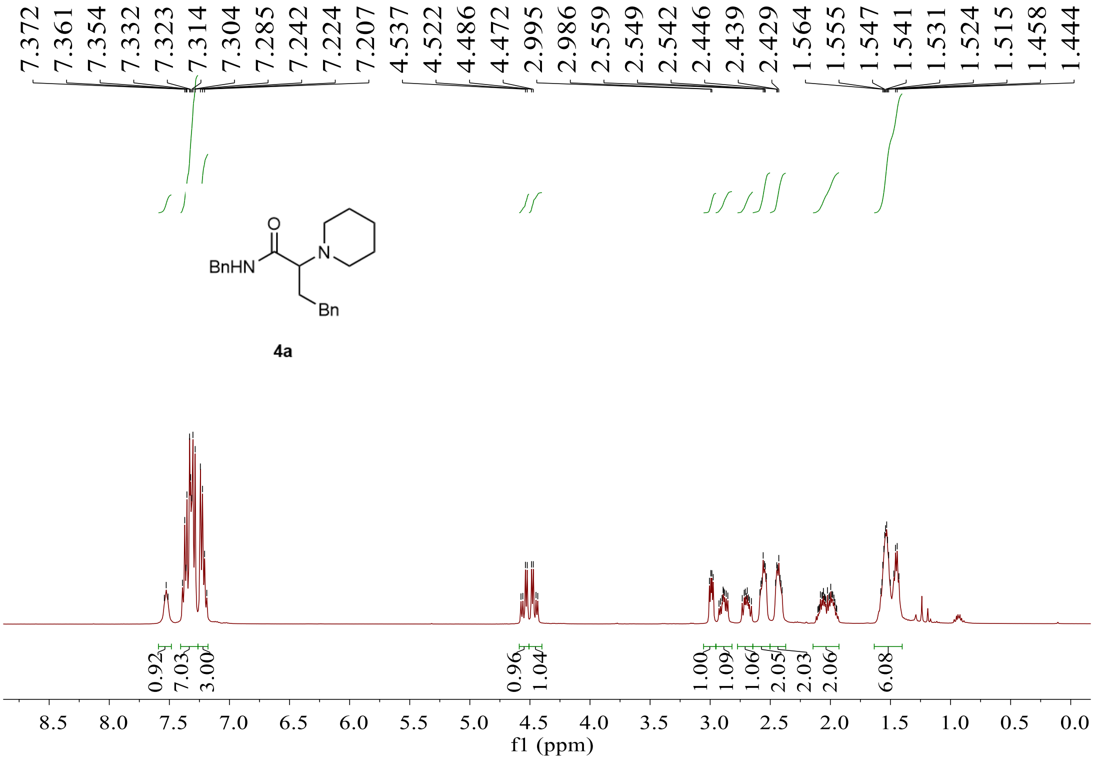
7.372
7.361
7.354
7.332
7.323
7.314
7.304
7.285
7.242
7.224
7.207
4.537
4.522
4.486
4.472
2.995
2.986
2.559
2.549
2.542
2.446
2.439
2.429
1.564
1.555
1.547
1.541
1.531
1.524
1.515
1.458
1.444
BnHN
O
N
Bn
4a
0.92
7.03
3.00
0.96
1.04
1.00
1.09
1.06
2.05
2.03
2.06
6.08
8.5 8.0 7.5 7.0 6.5 6.0 5.5 5.0 4.5 4.0 3.5 3.0 2.5 2.0 1.5 1.0 0.5 0.0
f1 (ppm)

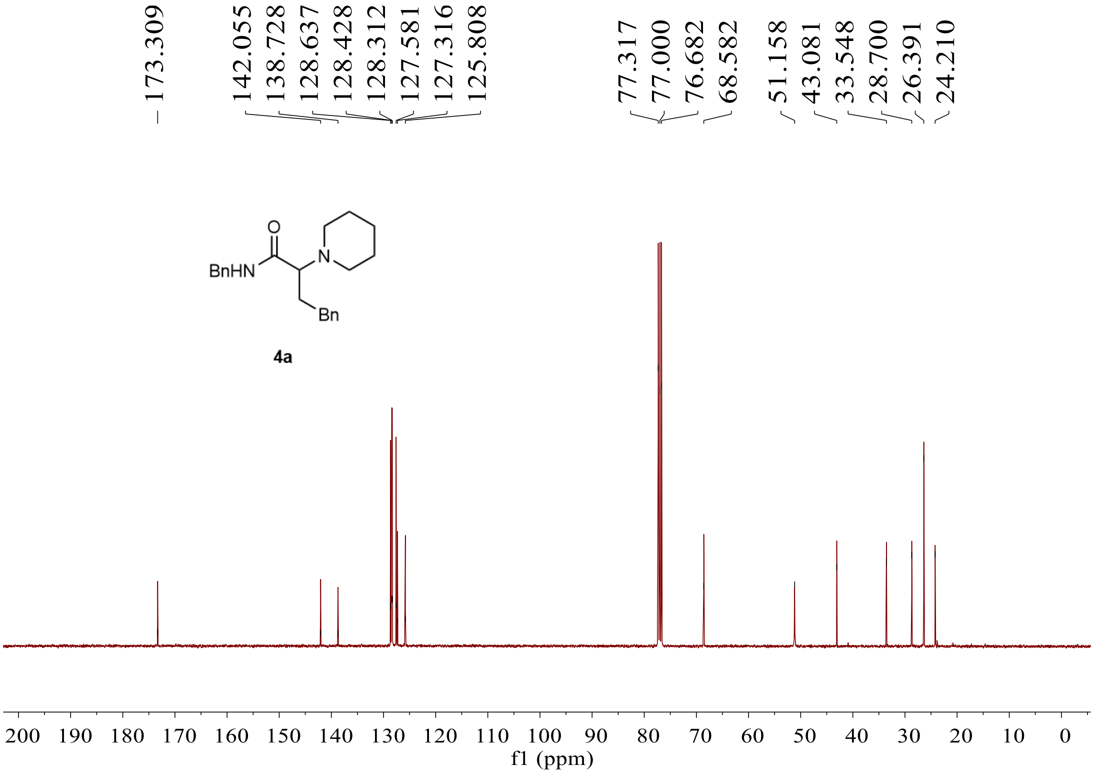
173.309
142.055
138.728
128.637
128.428
128.312
127.581
127.316
125.808
77.317
77.000
76.682
68.582
51.158
43.081
33.548
28.700
26.391
24.210
BnHN
O
N
Bn
4a
200 190 180 170 160 150 140 130 120 110 100 90 80 70 60 50 40 30 20 10 0
f1 (ppm)

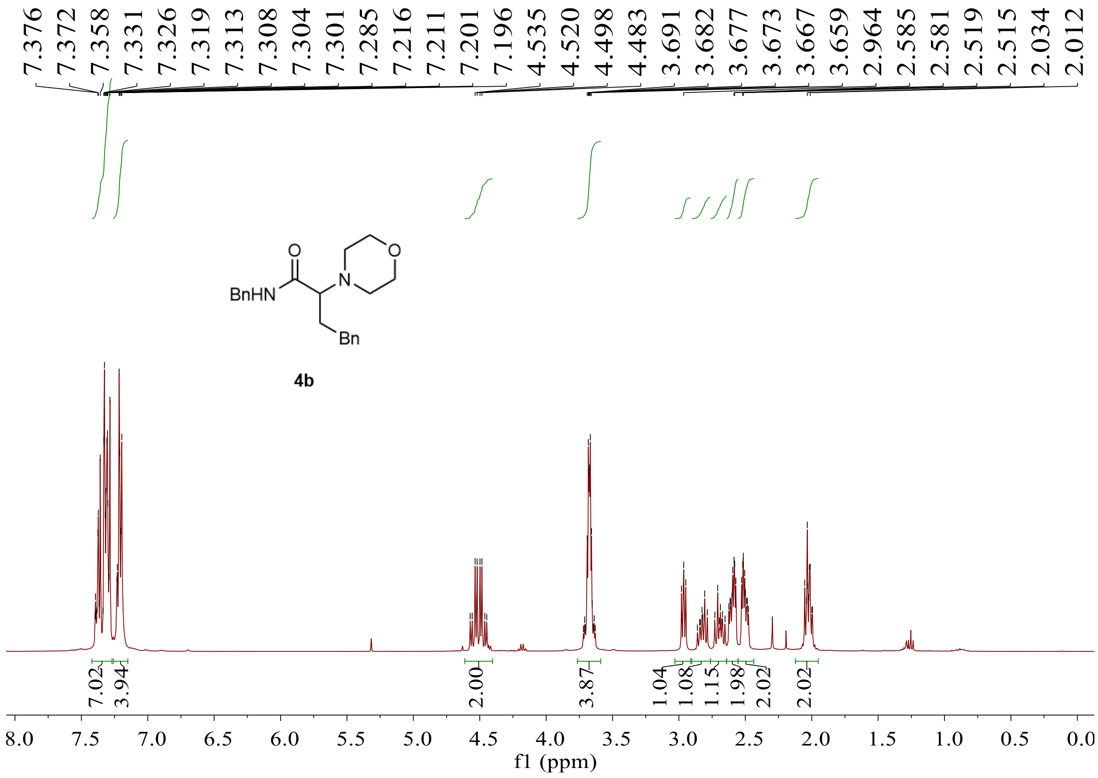
7.376
7.372
7.358
7.331
7.326
7.319
7.313
7.308
7.304
7.301
7.285
7.216
7.211
7.201
7.196
4.535
4.520
4.498
4.483
3.691
3.682
3.677
3.673
3.667
3.659
2.964
2.585
2.581
2.519
2.515
2.034
2.012
BnHN
O
N
O
Bn
4b
7.02
3.94
2.00
3.87
1.04
1.08
1.15
1.98
2.02
2.02
f1 (ppm)

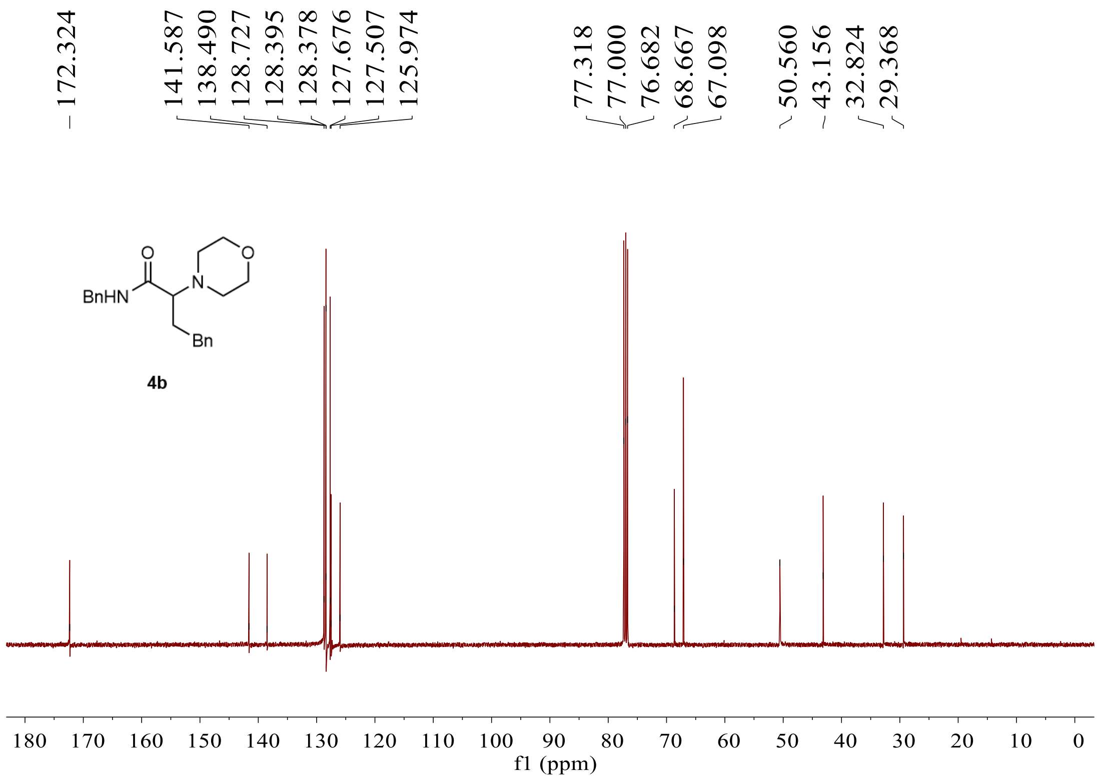
172.324
141.587
138.490
128.727
128.395
128.378
127.676
127.507
125.974
77.318
77.000
76.682
68.667
67.098
50.560
43.156
32.824
29.368
BnHN
O
N
O
Bn
4b
f1 (ppm)

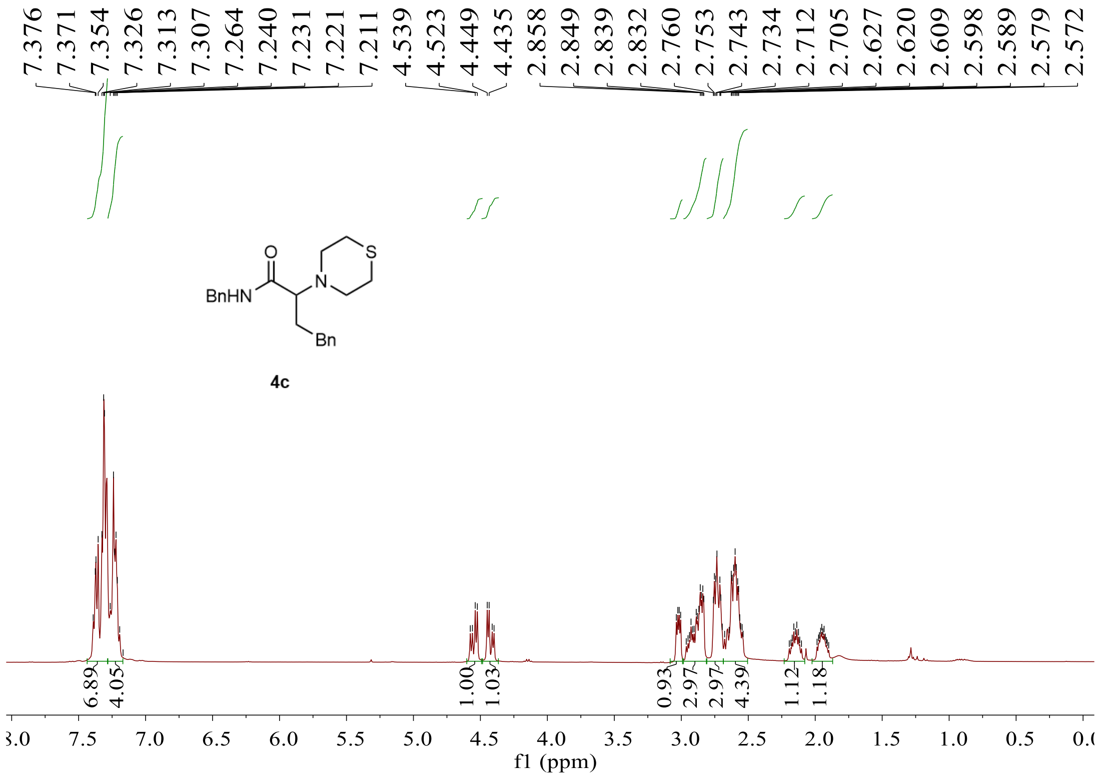

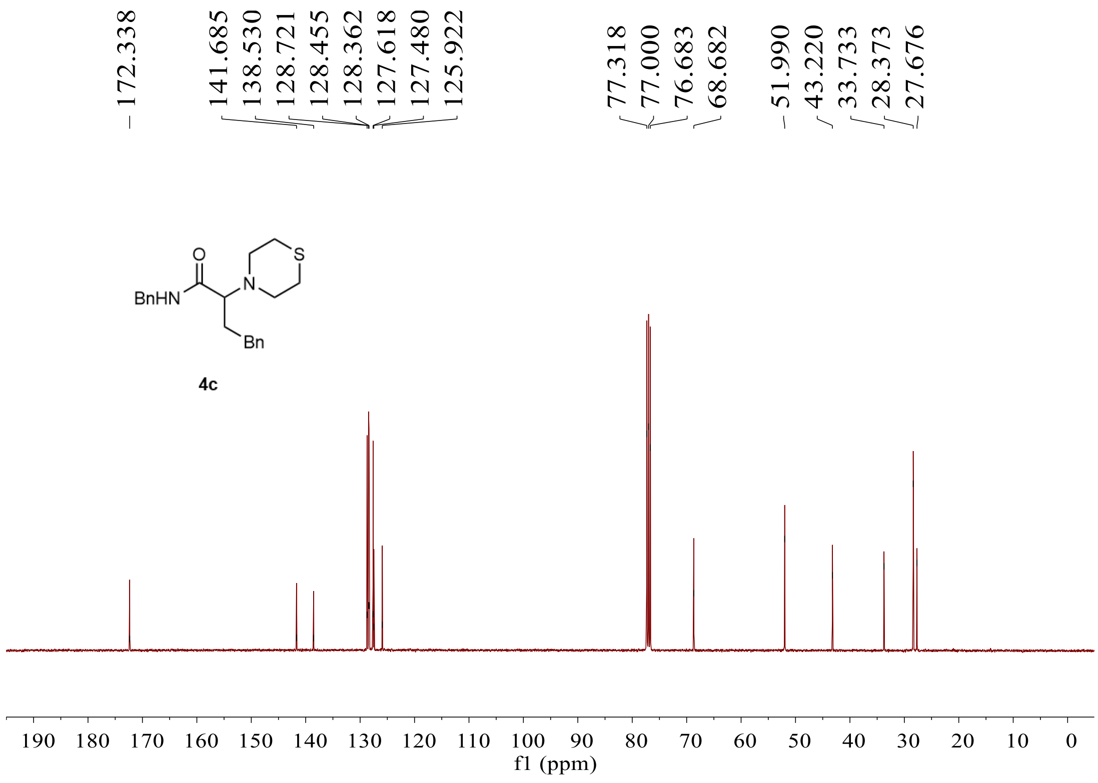

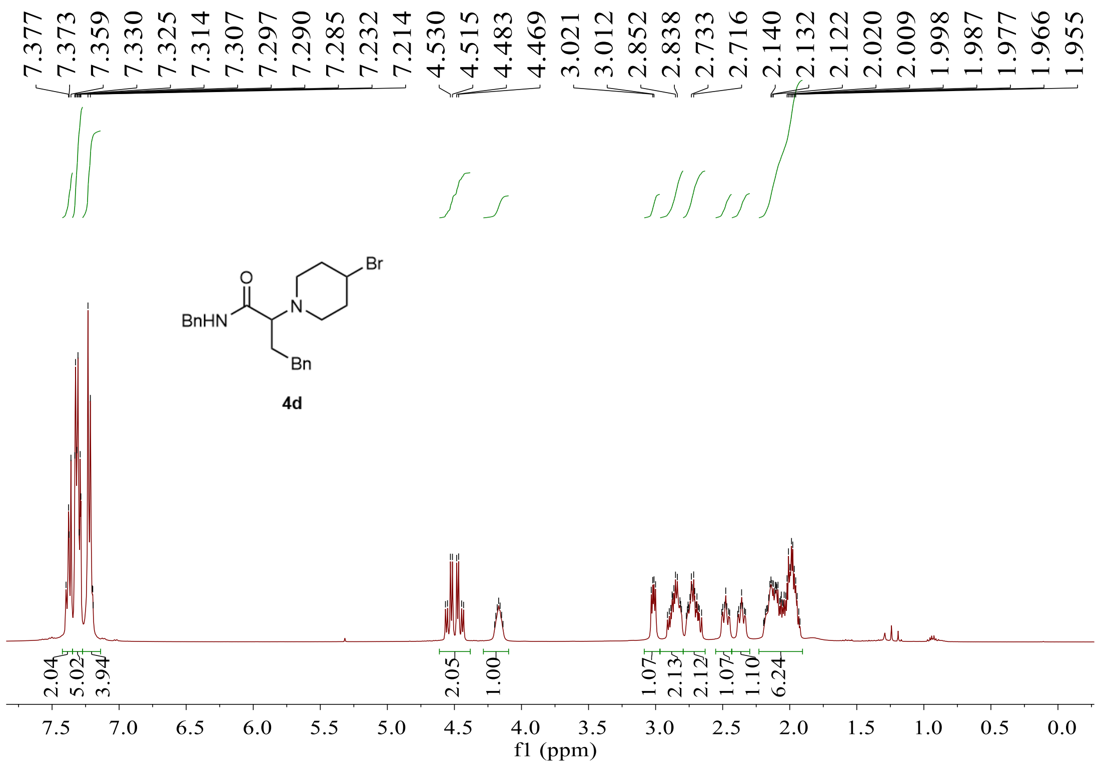
7.377 7.373 7.359 7.330 7.325 7.314 7.307 7.297 7.290 7.285 7.232 7.214 4.530 4.515 4.483 4.469 3.021 3.012 2.852 2.838 2.733 2.716 2.140 2.132 2.122 2.020 2.009 1.998 1.987 1.977 1.966 1.955
BnHN
O
N
Br
Bn
4d
2.04 5.02 3.94 2.05 1.00 1.07 2.13 2.12 1.07 1.10 6.24
7.5 7.0 6.5 6.0 5.5 5.0 4.5 4.0 3.5 3.0 2.5 2.0 1.5 1.0 0.5 0.0
f1 (ppm)

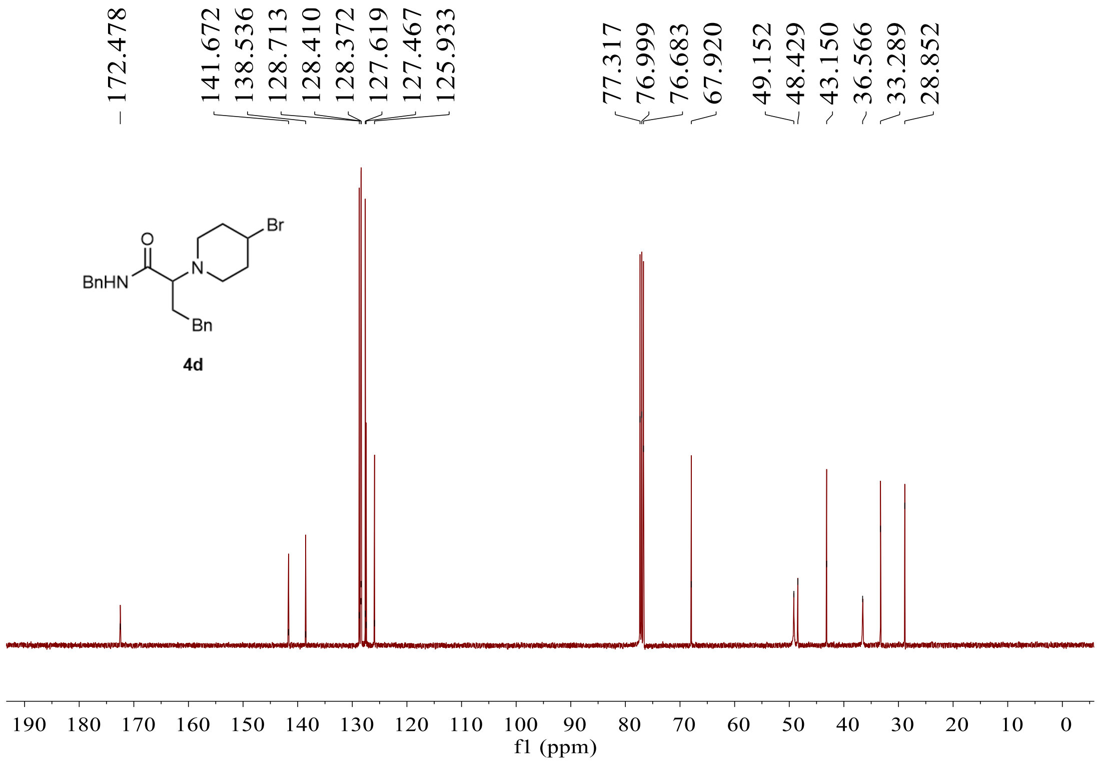
172.478 141.672 138.536 128.713 128.410 128.372 127.619 127.467 125.933 77.317 76.999 76.683 67.920 49.152 48.429 43.150 36.566 33.289 28.852
BnHN
O
N
Br
Bn
4d
190 180 170 160 150 140 130 120 110 100 90 80 70 60 50 40 30 20 10 0
f1 (ppm)

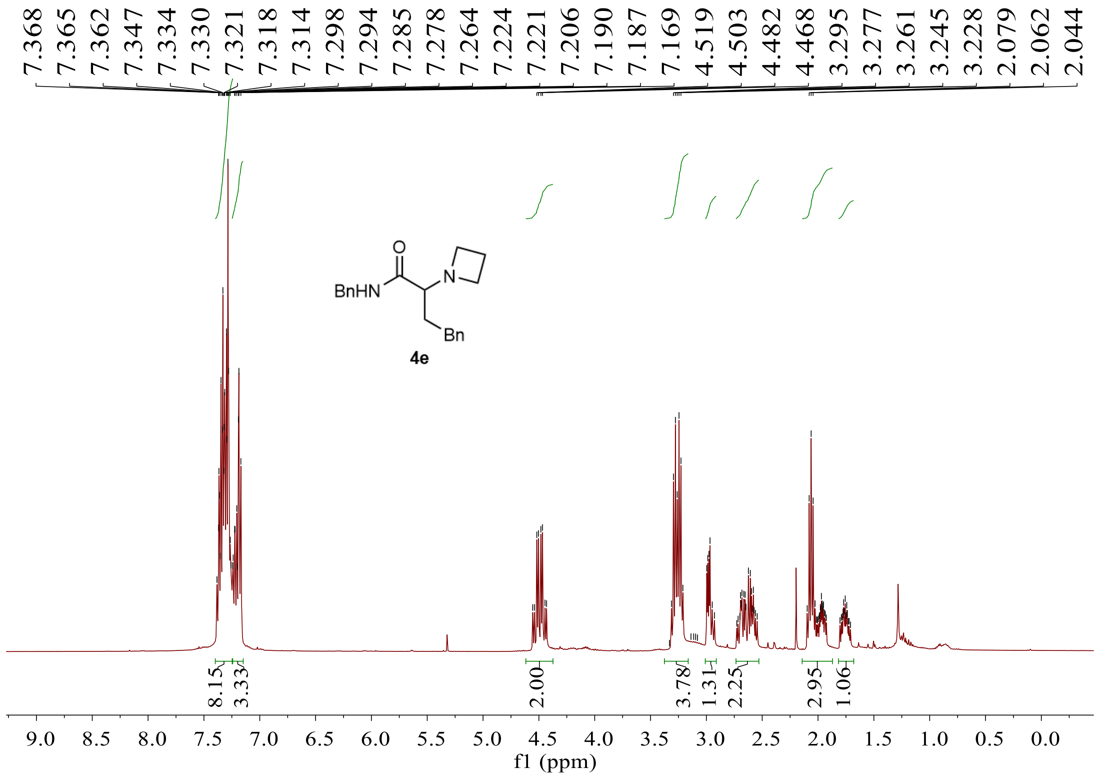

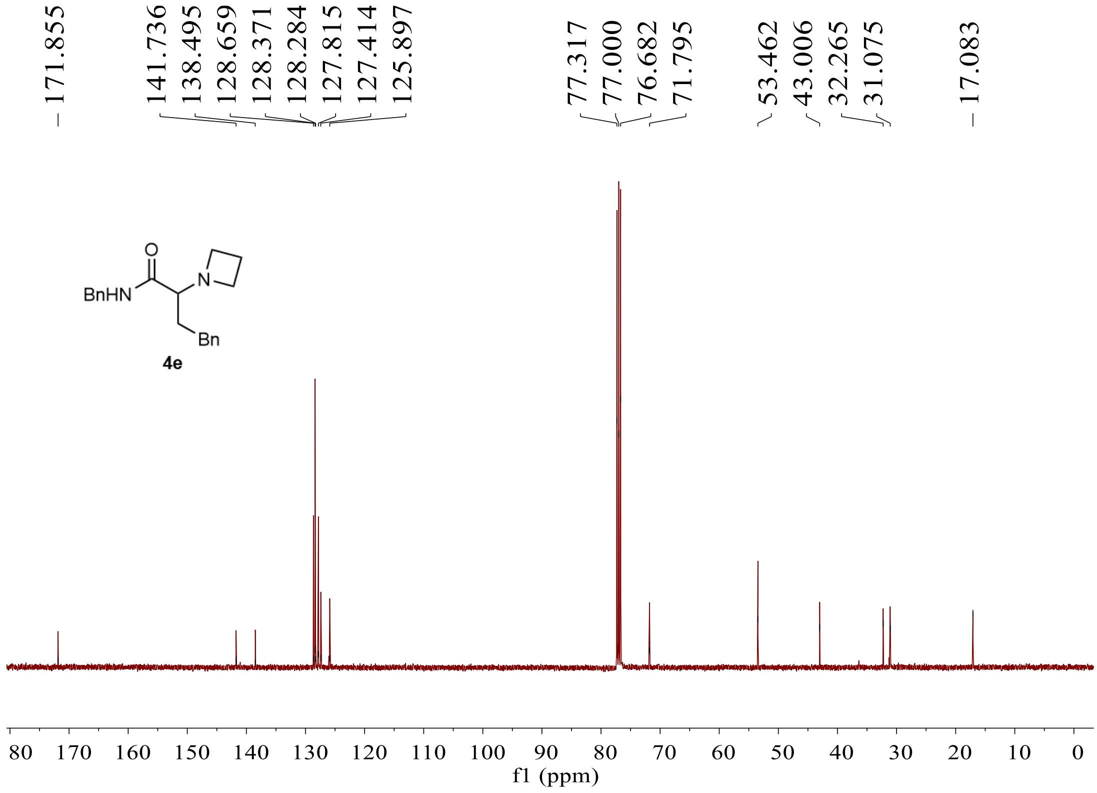

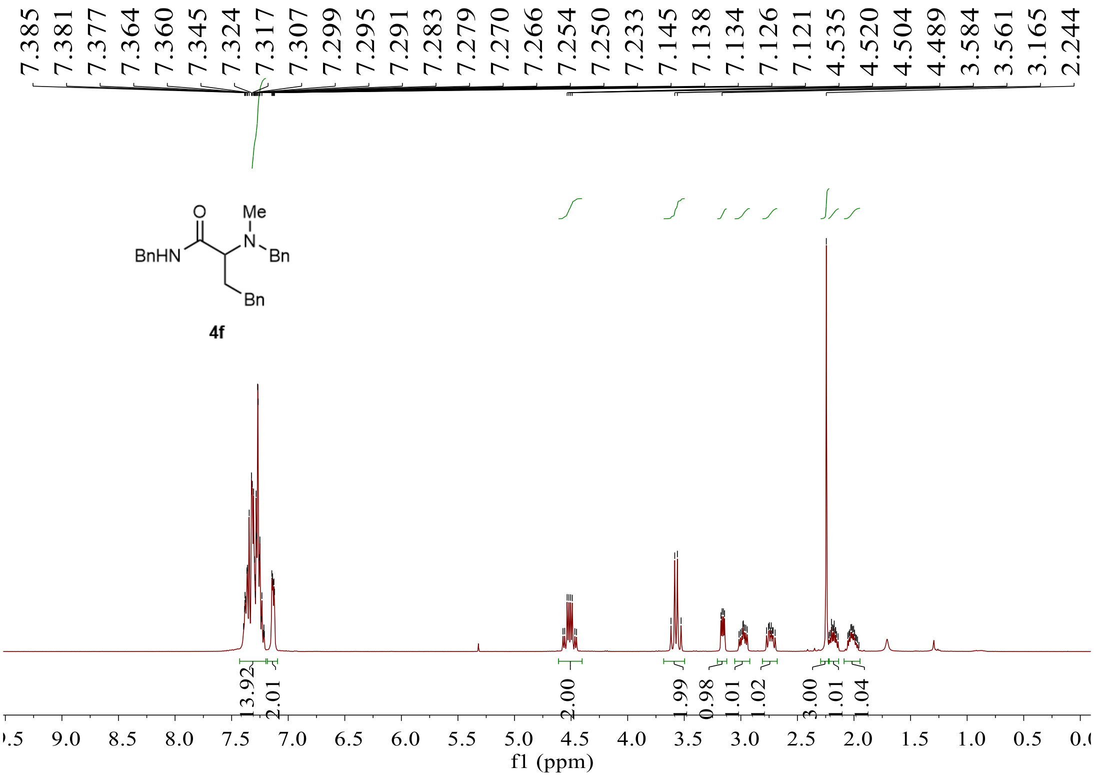
7.385
7.381
7.377
7.364
7.360
7.345
7.324
7.317
7.307
7.299
7.295
7.291
7.283
7.279
7.270
7.266
7.254
7.250
7.233
7.145
7.138
7.134
7.126
7.121
4.535
4.520
4.504
4.489
3.584
3.561
3.165
2.244
O
Me
N
BnHN
Bn
Bn
4f
13.92
2.01
2.00
1.99
0.98
1.01
1.02
3.00
1.01
1.04
9.0 8.5 8.0 7.5 7.0 6.5 6.0 5.5 5.0 4.5 4.0 3.5 3.0 2.5 2.0 1.5 1.0 0.5
f1 (ppm)

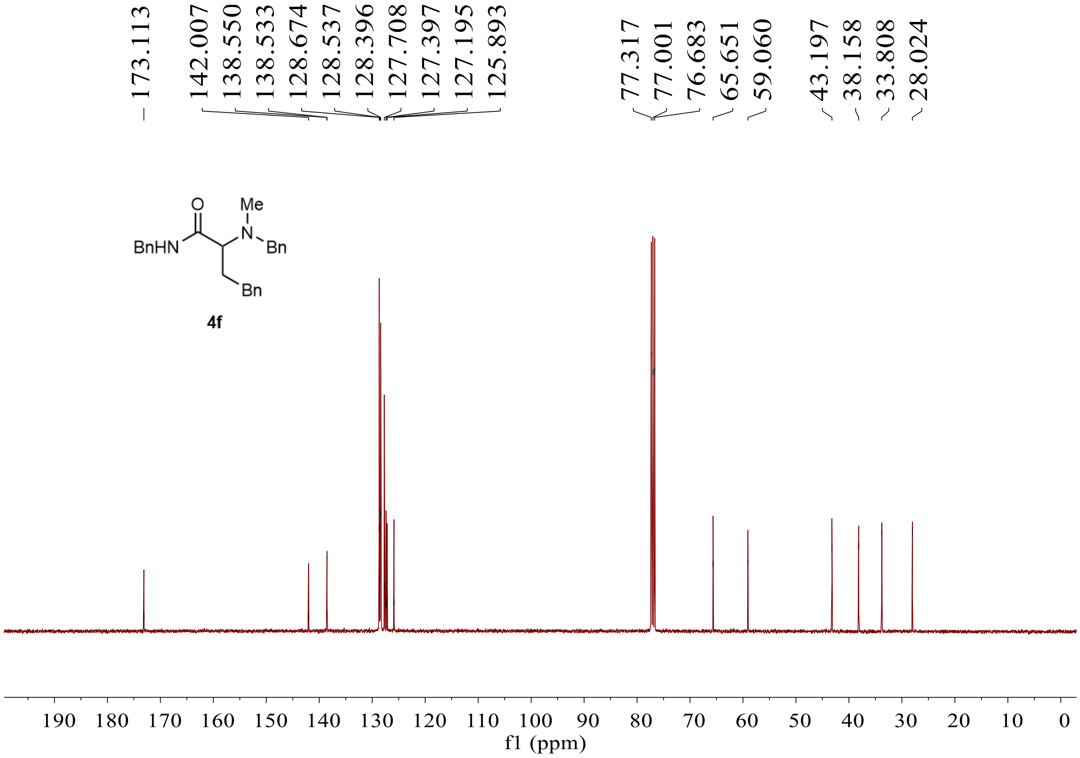
173.113
142.007
138.550
138.533
128.674
128.537
128.396
127.708
127.397
127.195
125.893
77.317
77.001
76.683
65.651
59.060
43.197
38.158
33.808
28.024
O
Me
N
BnHN
Bn
Bn
4f
190 180 170 160 150 140 130 120 110 100 90 80 70 60 50 40 30 20 10 0
f1 (ppm)

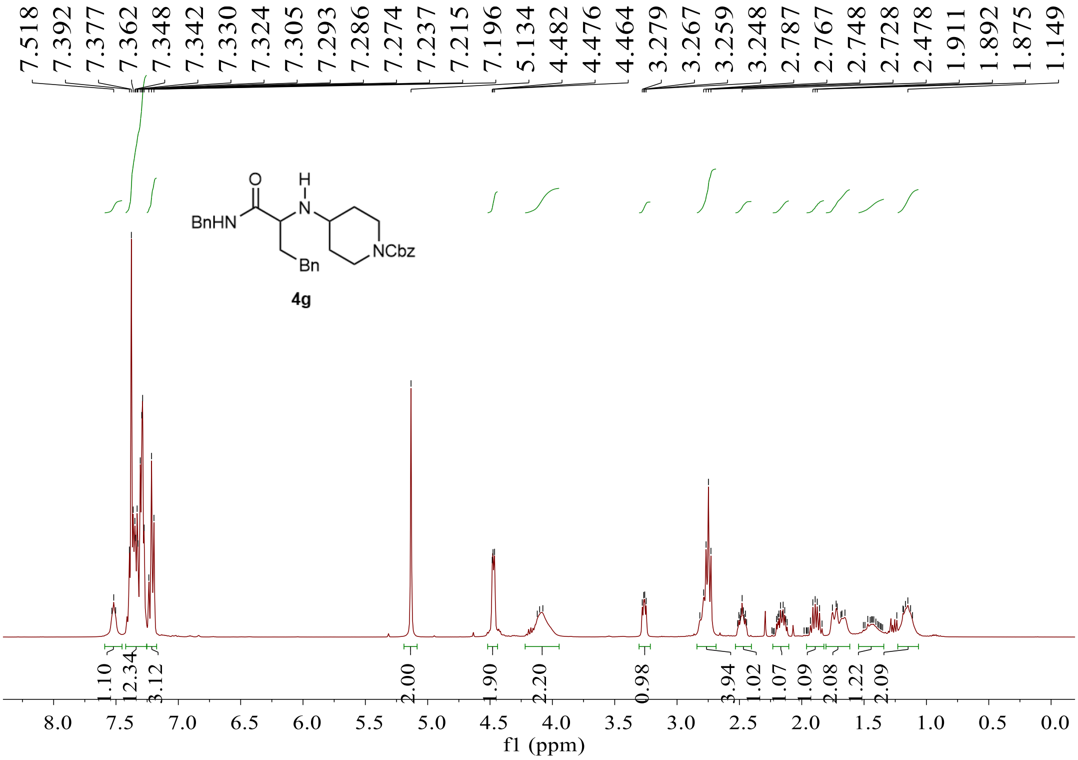
7.518
7.392
7.377
7.362
7.348
7.342
7.330
7.324
7.305
7.293
7.286
7.274
7.237
7.215
7.196
5.134
4.482
4.476
4.464
3.279
3.267
3.259
3.248
2.787
2.767
2.748
2.728
2.478
1.911
1.892
1.875
1.149
BnHN
O
H
N
NCbz
Bn
4g
1.10
12.34
3.12
2.00
1.90
2.20
0.98
3.94
1.02
1.07
1.09
2.08
1.22
2.09
8.0 7.5 7.0 6.5 6.0 5.5 5.0 4.5 4.0 3.5 3.0 2.5 2.0 1.5 1.0 0.5 0.0
f1 (ppm)

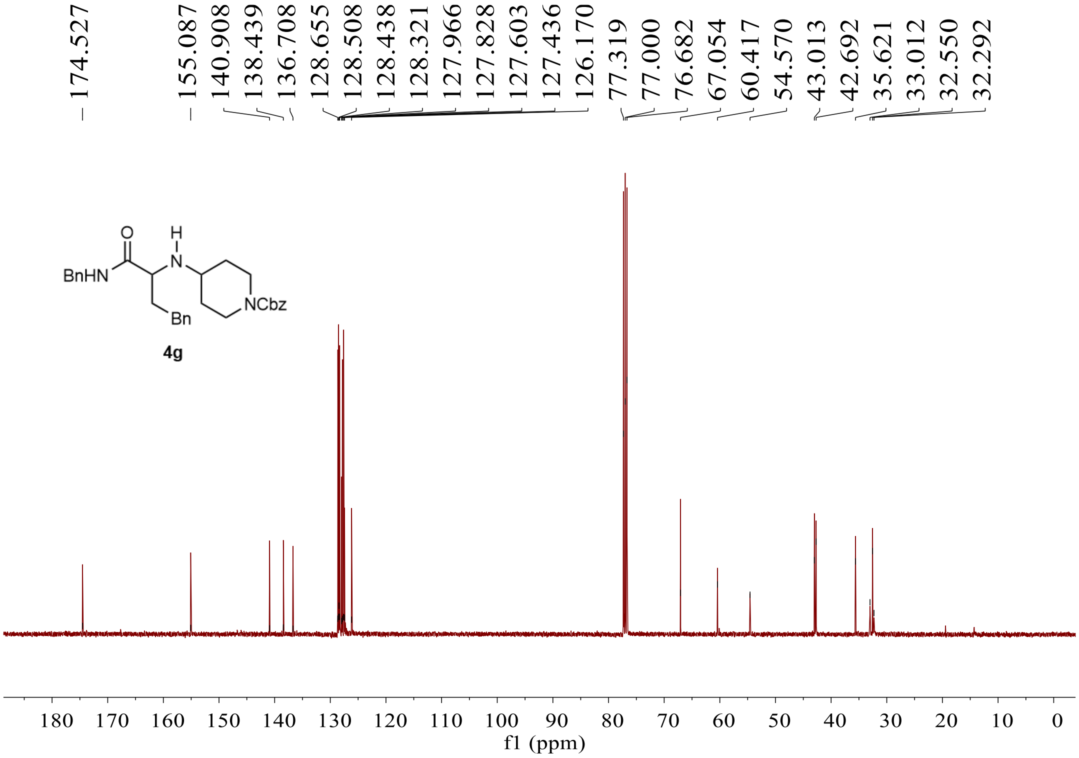
174.527
155.087
140.908
138.439
136.708
128.655
128.508
128.438
128.321
127.966
127.828
127.603
127.436
126.170
77.319
77.000
76.682
67.054
60.417
54.570
43.013
42.692
35.621
33.012
32.550
32.292
BnHN
O
H
N
NCbz
Bn
4g
180 170 160 150 140 130 120 110 100 90 80 70 60 50 40 30 20 10 0
f1 (ppm)

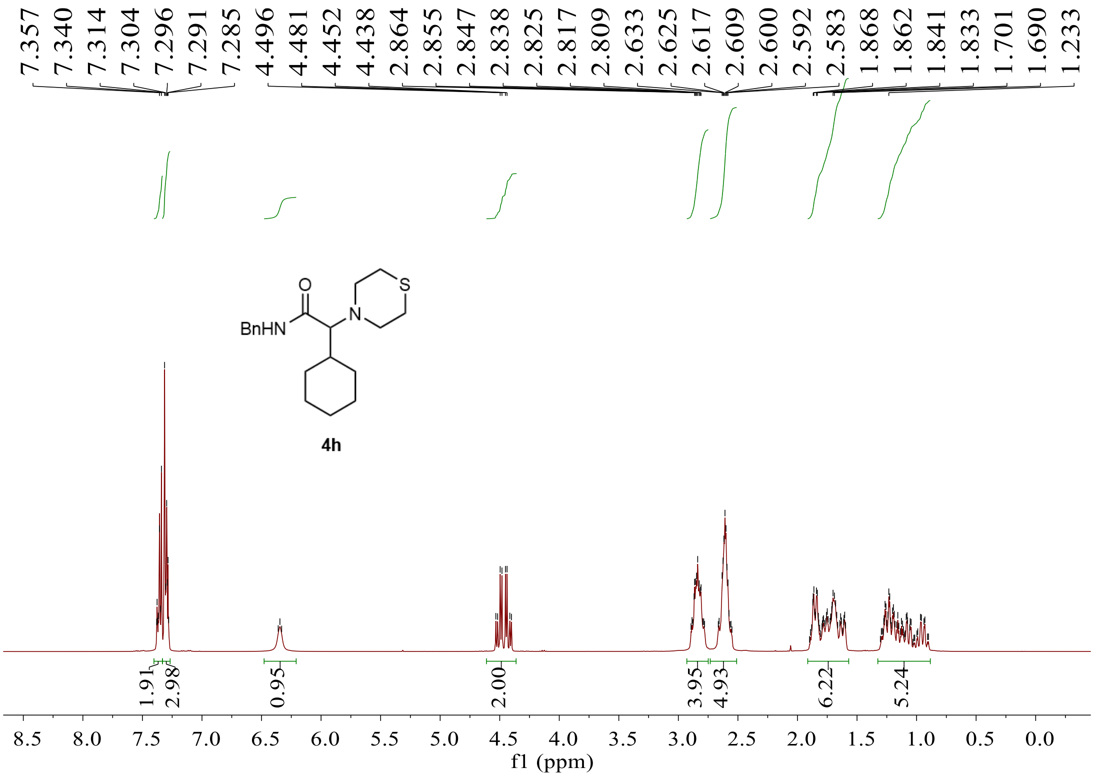

7.357 7.340 7.314 7.304 7.296 7.291 7.285 4.496 4.481 4.452 4.438 2.864 2.855 2.847 2.838 2.825 2.817 2.809 2.633 2.625 2.617 2.609 2.600 2.592 2.583 1.868 1.862 1.841 1.833 1.701 1.690 1.233
BnHN
O
N
S
4h
1.91 2.98 0.95 2.00 3.95 4.93 6.22 5.24
8.5 8.0 7.5 7.0 6.5 6.0 5.5 5.0 4.5 4.0 3.5 3.0 2.5 2.0 1.5 1.0 0.5 0.0
f1 (ppm)

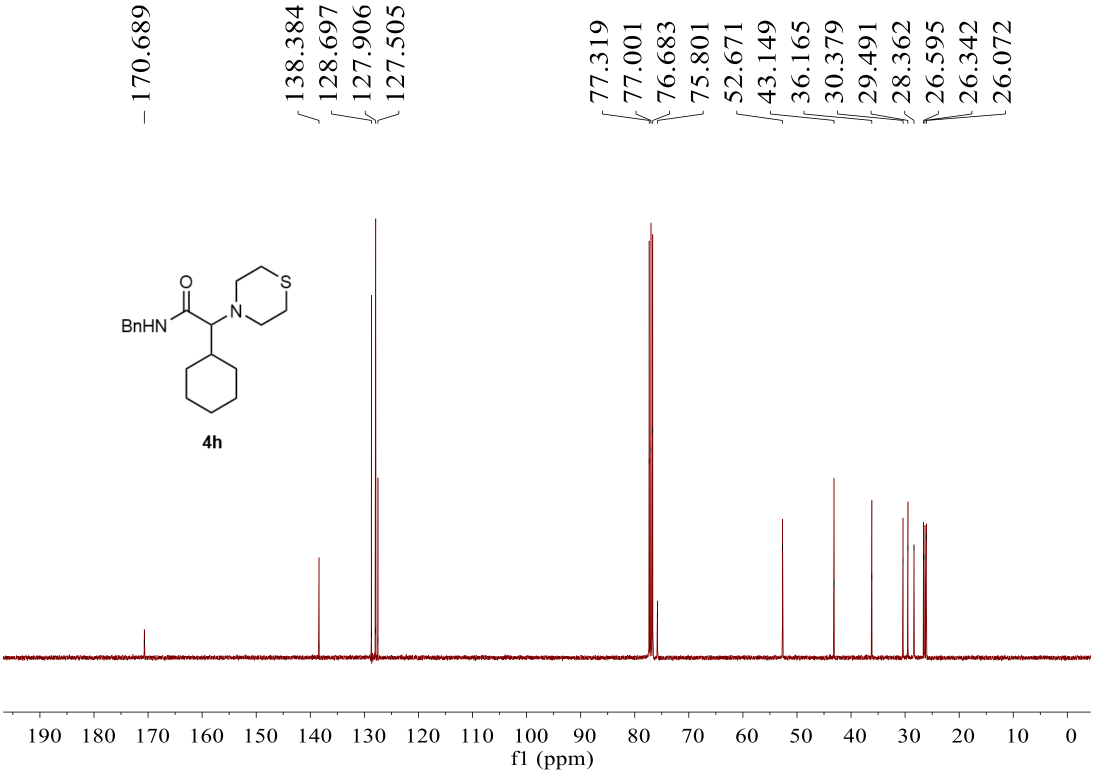

170.689
138.384 128.697 127.906 127.505
77.319 77.001 76.683 75.801 52.671 43.149 36.165 30.379 29.491 28.362 26.595 26.342 26.072
BnHN
O
N
S
4h
190 180 170 160 150 140 130 120 110 100 90 80 70 60 50 40 30 20 10 0
f1 (ppm)

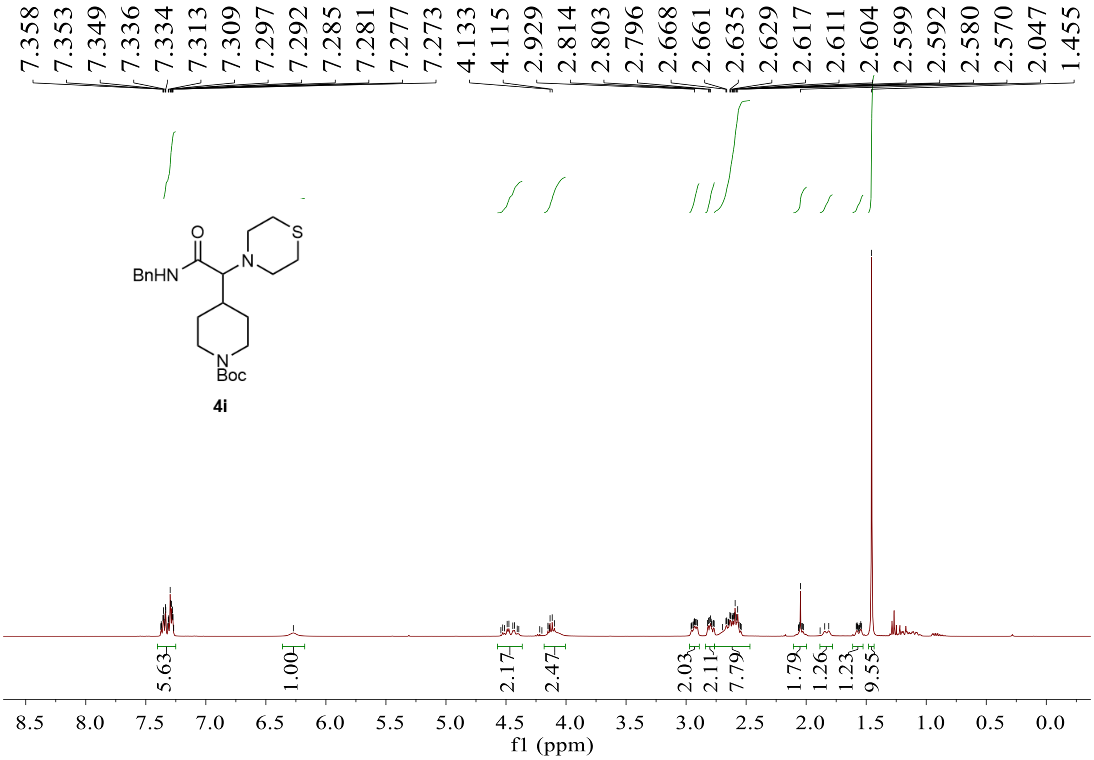
7.358 7.353 7.349 7.336 7.334 7.313 7.309 7.297 7.292 7.285 7.281 7.277 7.273 4.133 4.115 2.929 2.814 2.803 2.796 2.668 2.661 2.635 2.629 2.617 2.611 2.604 2.599 2.592 2.580 2.570 2.047 1.455
O
BnHN
N
S
N
Boc
4i
5.63
1.00
2.17
2.47
2.03
2.11
7.79
1.79
1.26
1.23
9.55
8.5 8.0 7.5 7.0 6.5 6.0 5.5 5.0 4.5 4.0 3.5 3.0 2.5 2.0 1.5 1.0 0.5 0.0
f1 (ppm)

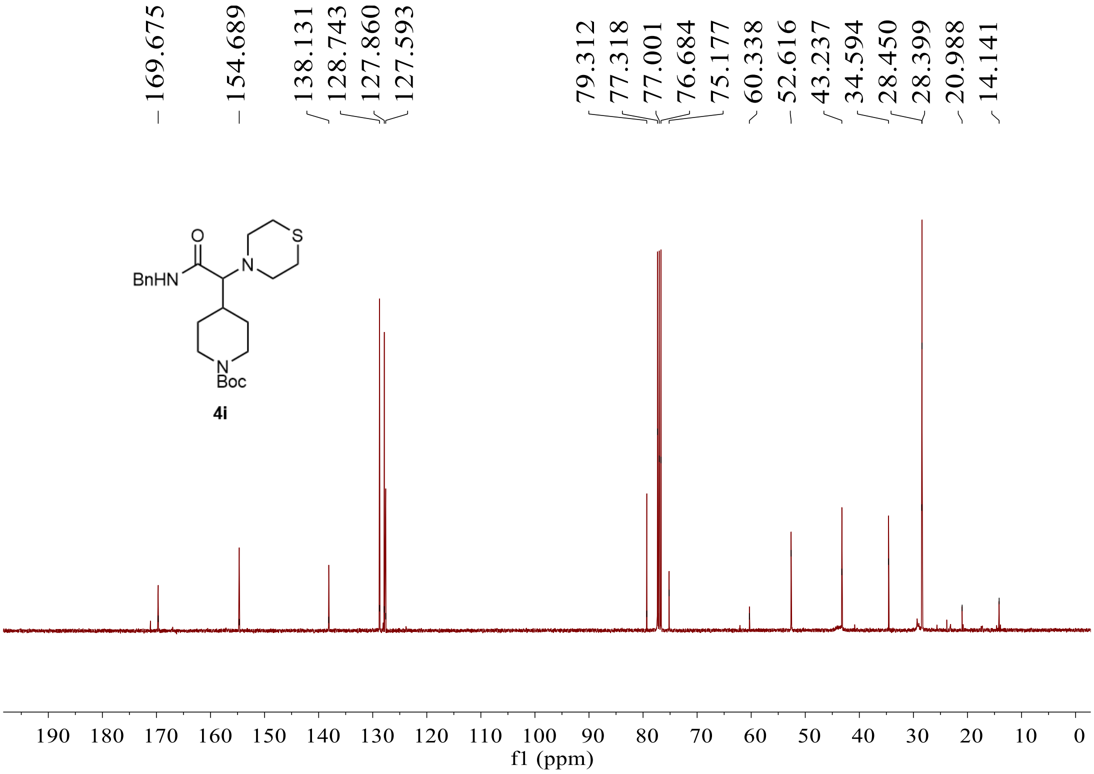
169.675
154.689
138.131 128.743 127.860 127.593
79.312 77.318 77.001 76.684 75.177 60.338 52.616 43.237 34.594 28.450 28.399 20.988 14.141
O
BnHN
N
S
N
Boc
4i
190 180 170 160 150 140 130 120 110 100 90 80 70 60 50 40 30 20 10 0
f1 (ppm)

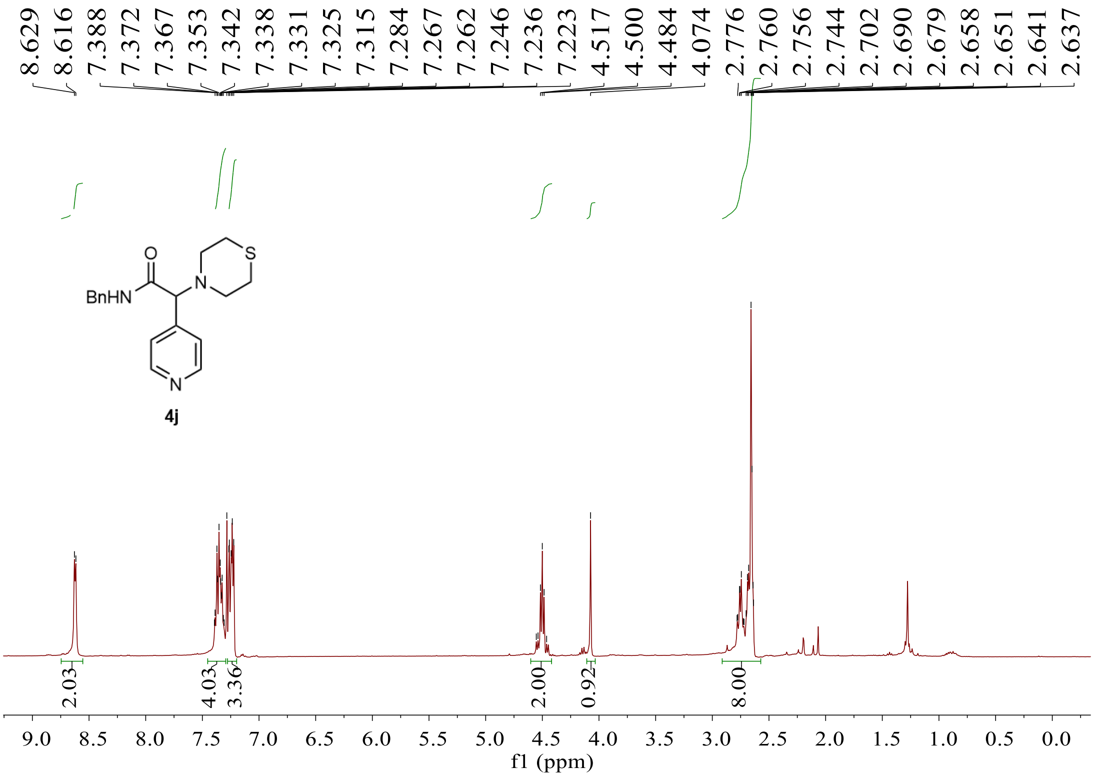
8.629
8.616
7.388
7.372
7.367
7.353
7.342
7.338
7.331
7.325
7.315
7.284
7.267
7.262
7.246
7.236
7.223
4.517
4.500
4.484
4.074
2.776
2.760
2.756
2.744
2.702
2.690
2.679
2.658
2.651
2.641
2.637
BnHN
O
N
S
N
4j
2.03
4.03
3.36
2.00
0.92
8.00
f1 (ppm)

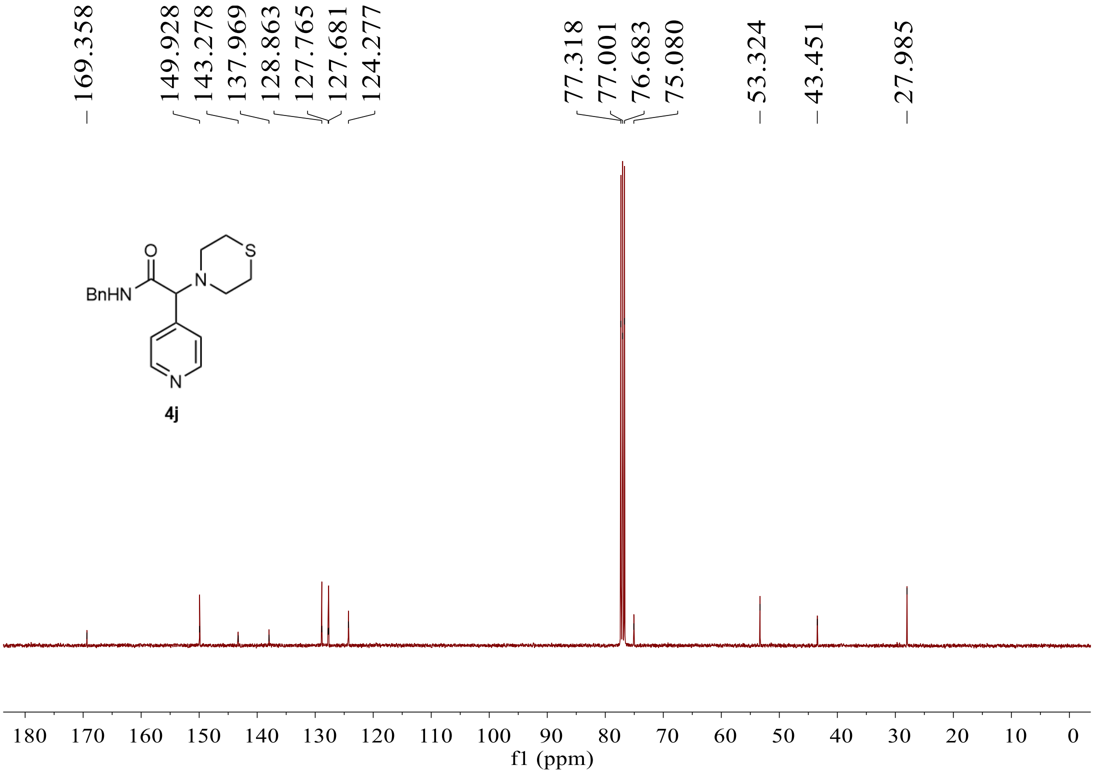
169.358
149.928
143.278
137.969
128.863
127.765
127.681
124.277
77.318
77.001
76.683
75.080
53.324
43.451
27.985
BnHN
O
N
S
N
4j
f1 (ppm)

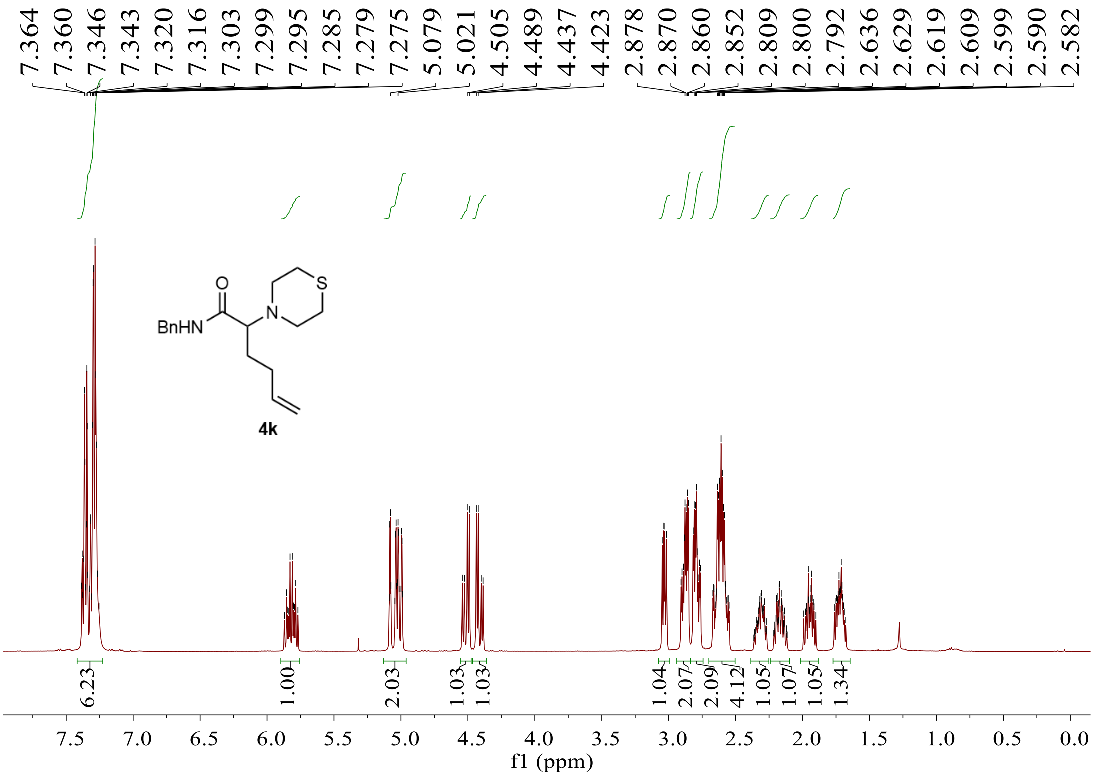
7.364
7.360
7.346
7.343
7.320
7.316
7.303
7.299
7.295
7.285
7.279
7.275
5.079
5.021
4.505
4.489
4.437
4.423
2.878
2.870
2.860
2.852
2.809
2.800
2.792
2.636
2.629
2.619
2.609
2.599
2.590
2.582
BnHN
O
N
S
4k
6.23
1.00
2.03
1.03
1.03
1.04
2.07
2.09
4.12
1.05
1.07
1.05
1.34
f1 (ppm)

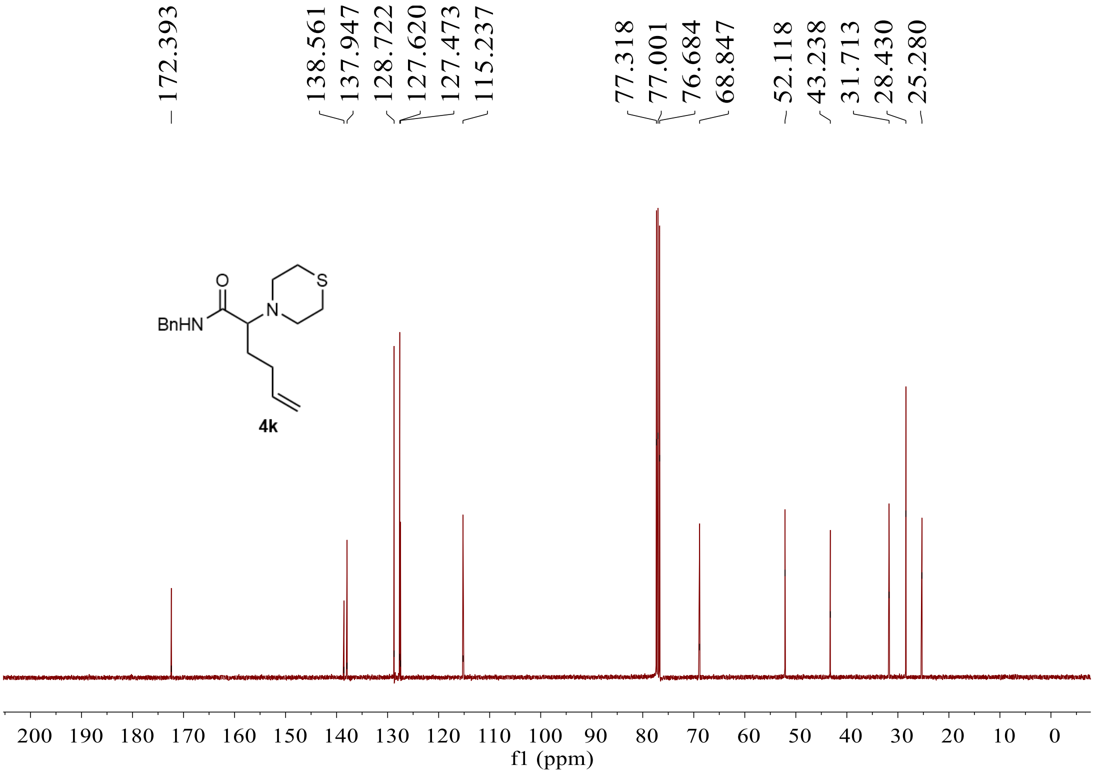
172.393
138.561
137.947
128.722
127.620
127.473
115.237
77.318
77.001
76.684
68.847
52.118
43.238
31.713
28.430
25.280
BnHN
O
N
S
4k
f1 (ppm)

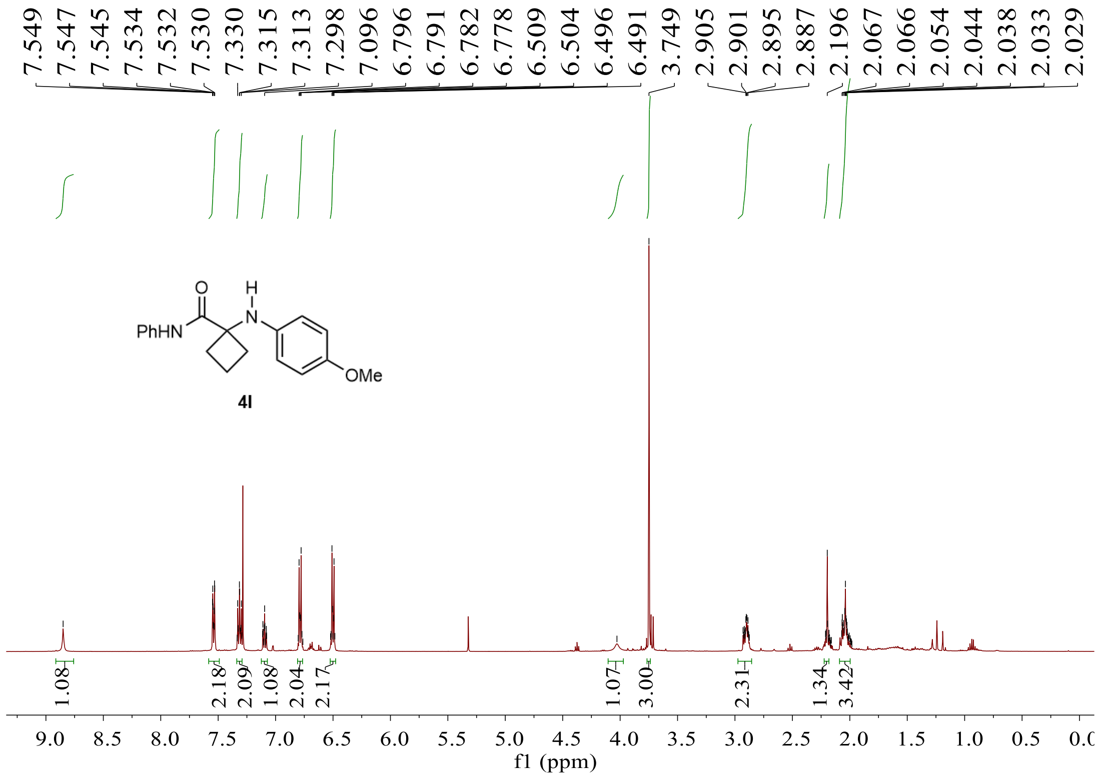
7.549
7.547
7.545
7.534
7.532
7.530
7.330
7.315
7.313
7.298
7.096
6.796
6.791
6.782
6.778
6.509
6.504
6.496
6.491
3.749
2.905
2.901
2.895
2.887
2.196
2.067
2.066
2.054
2.044
2.038
2.033
2.029
PhHN
O
H
N
OMe
4l
1.08
2.18
2.09
1.08
2.04
2.17
1.07
3.00
2.31
1.34
3.42
f1 (ppm)

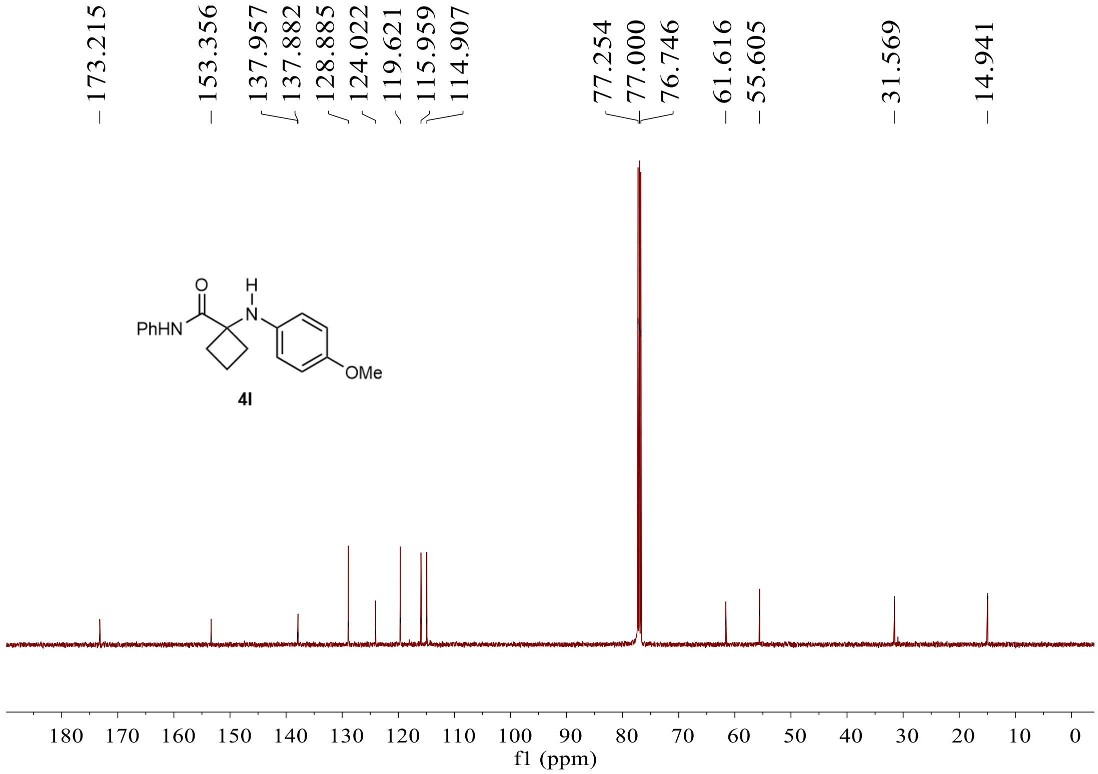
173.215
153.356
137.957
137.882
128.885
124.022
119.621
115.959
114.907
77.254
77.000
76.746
61.616
55.605
31.569
14.941
PhHN
O
H
N
OMe
4l
f1 (ppm)

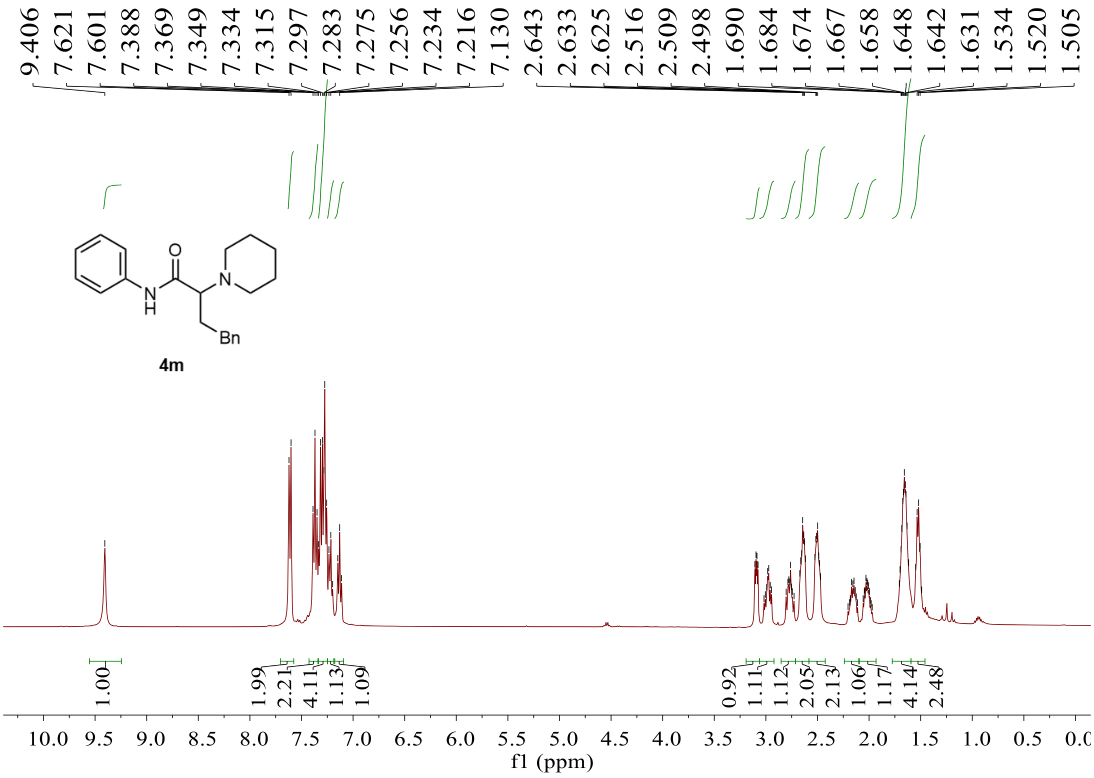

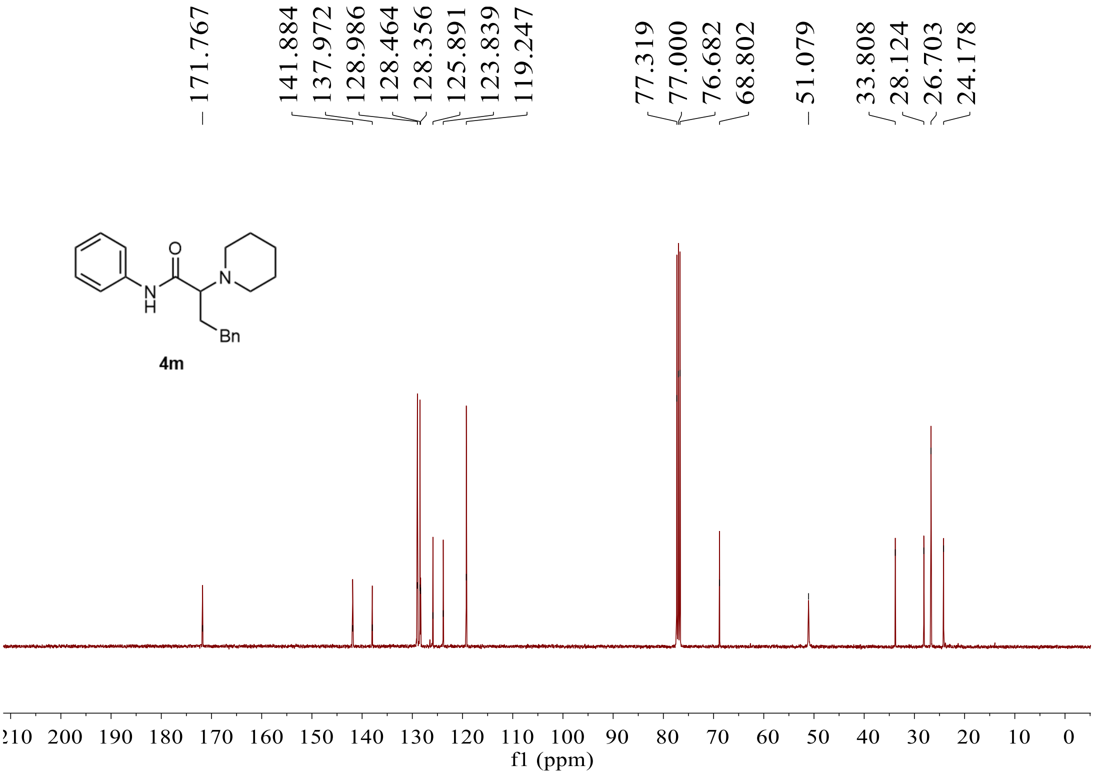

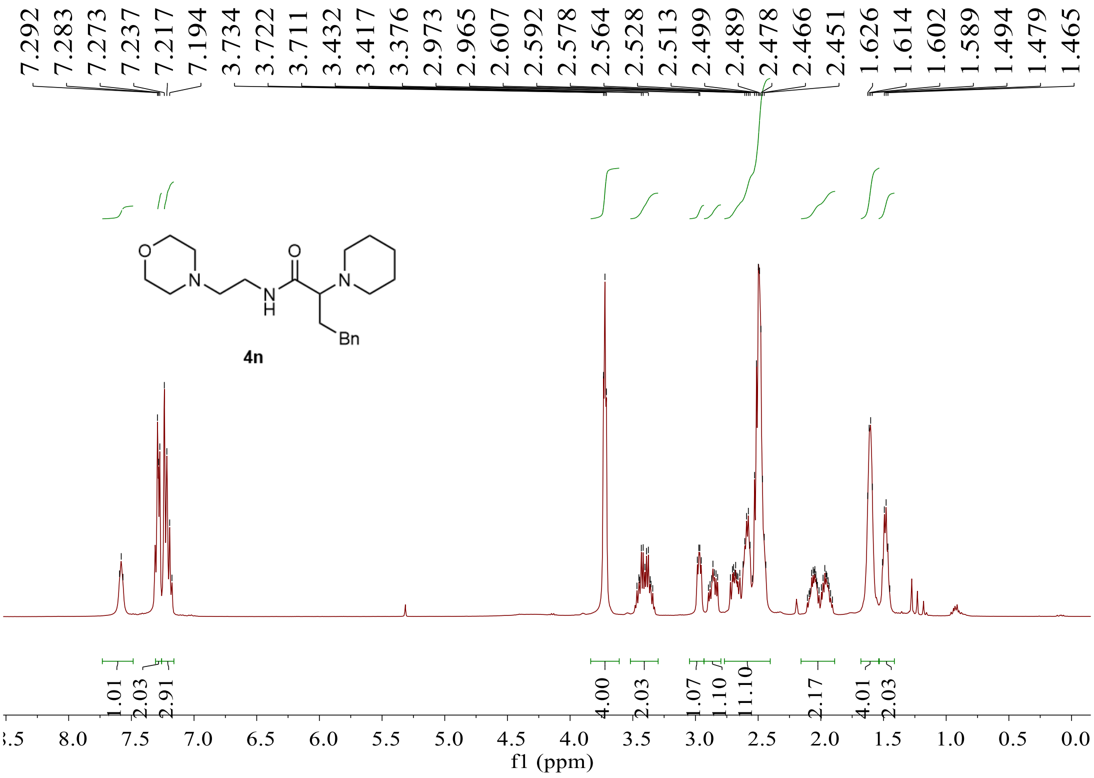
7.292 7.283 7.273 7.237 7.217 7.194 3.734 3.722 3.711 3.432 3.417 3.376 2.973 2.965 2.607 2.592 2.578 2.564 2.528 2.513 2.499 2.489 2.478 2.466 2.451 1.626 1.614 1.602 1.589 1.494 1.479 1.465
O
N
N
H
O
N
Bn
4n
1.01 2.03 2.91 4.00 2.03 1.07 1.10 11.10 2.17 4.01 2.03
8.0 7.5 7.0 6.5 6.0 5.5 5.0 4.5 4.0 3.5 3.0 2.5 2.0 1.5 1.0 0.5 0.0
f1 (ppm)

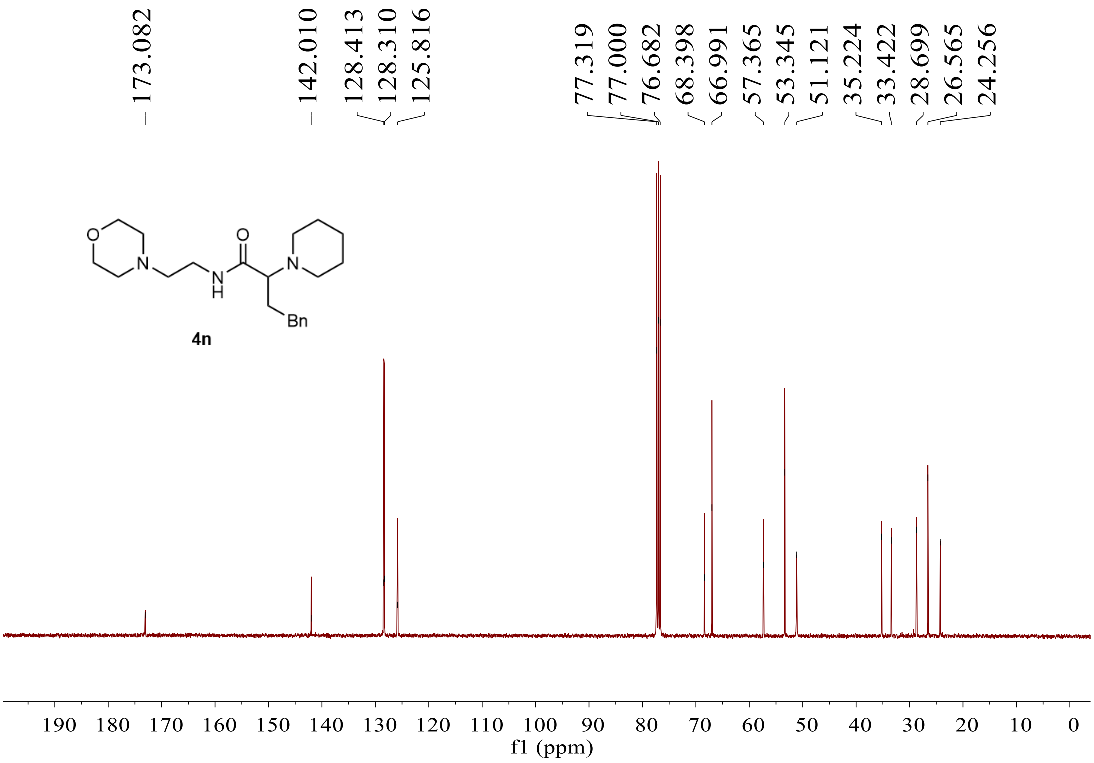
173.082
142.010 128.413 128.310 125.816
77.319 77.000 76.682 68.398 66.991 57.365 53.345 51.121 35.224 33.422 28.699 26.565 24.256
O
N
N
H
O
N
Bn
4n
190 180 170 160 150 140 130 120 110 100 90 80 70 60 50 40 30 20 10 0
f1 (ppm)

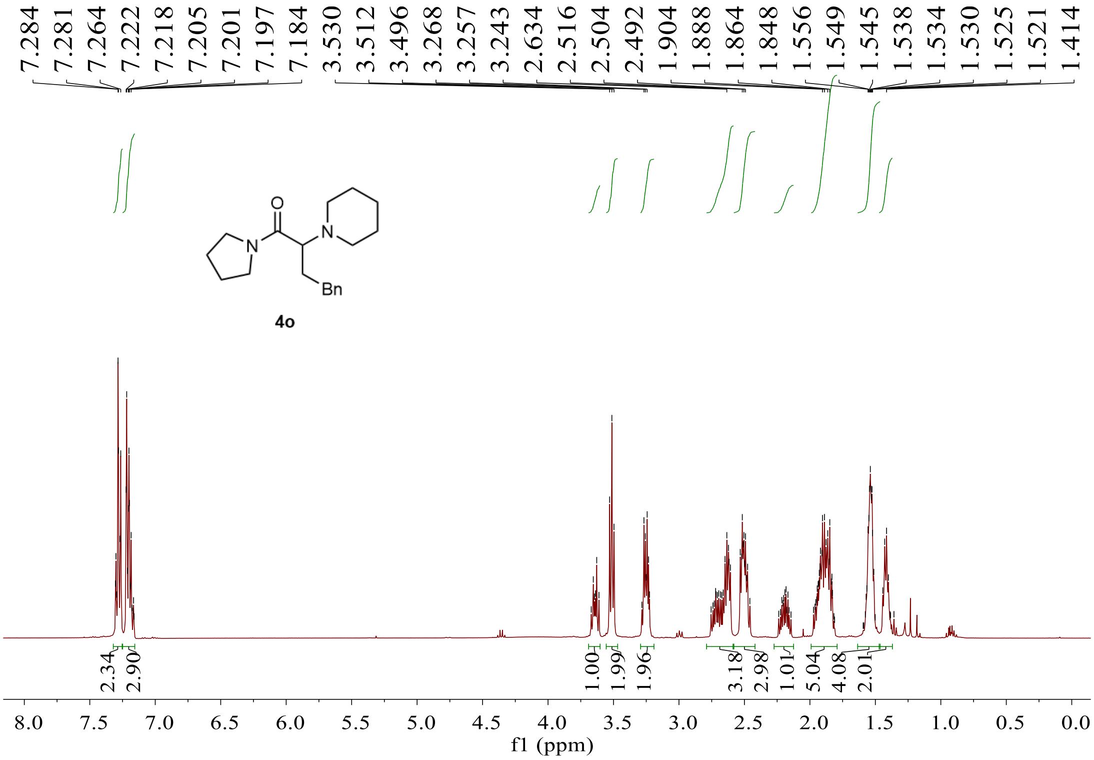
7.284 7.281 7.264 7.222 7.218 7.205 7.201 7.197 7.184 3.530 3.512 3.496 3.268 3.257 3.243 2.634 2.516 2.504 2.492 1.904 1.888 1.864 1.848 1.556 1.549 1.545 1.538 1.534 1.530 1.525 1.521 1.414
O
N
N
Bn
4o
2.34 2.90 1.00 1.99 1.96 3.18 2.98 1.01 5.04 4.08 2.01
8.0 7.5 7.0 6.5 6.0 5.5 5.0 4.5 4.0 3.5 3.0 2.5 2.0 1.5 1.0 0.5 0.0
f1 (ppm)

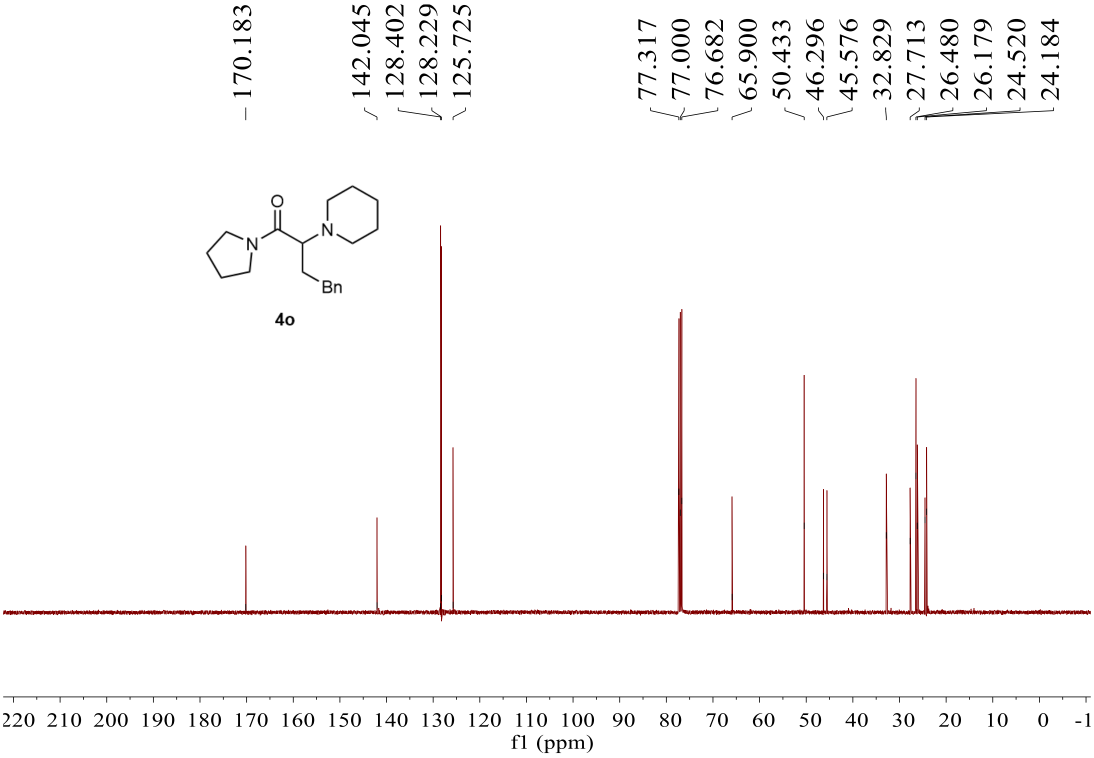
170.183
142.045 128.402 128.229 125.725
77.317 77.000 76.682 65.900 50.433 46.296 45.576 32.829 27.713 26.480 26.179 24.520 24.184
O
N
N
Bn
4o
220 210 200 190 180 170 160 150 140 130 120 110 100 90 80 70 60 50 40 30 20 10 0 -1
f1 (ppm)

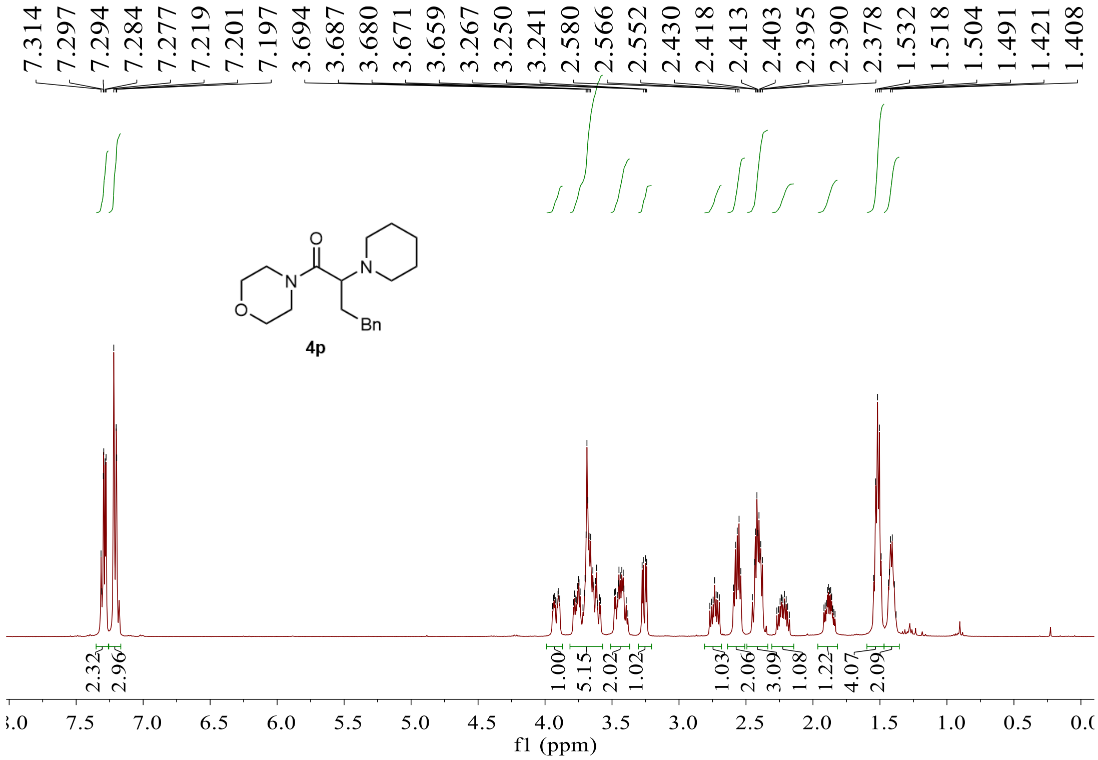

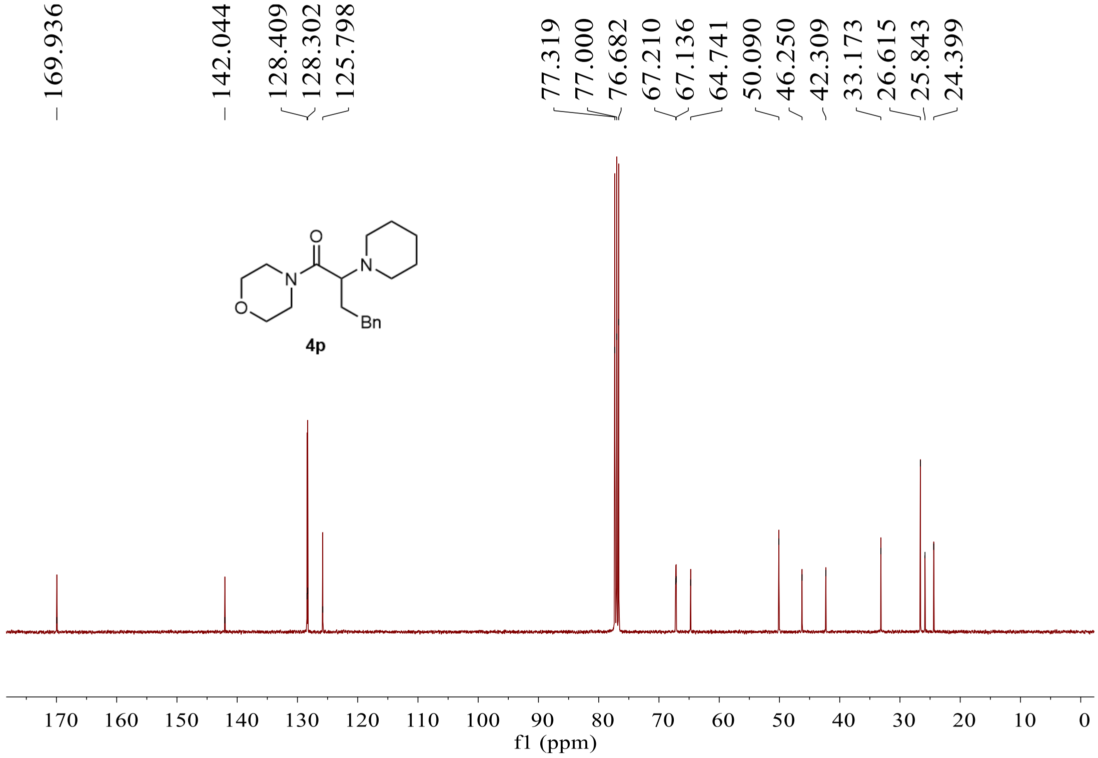

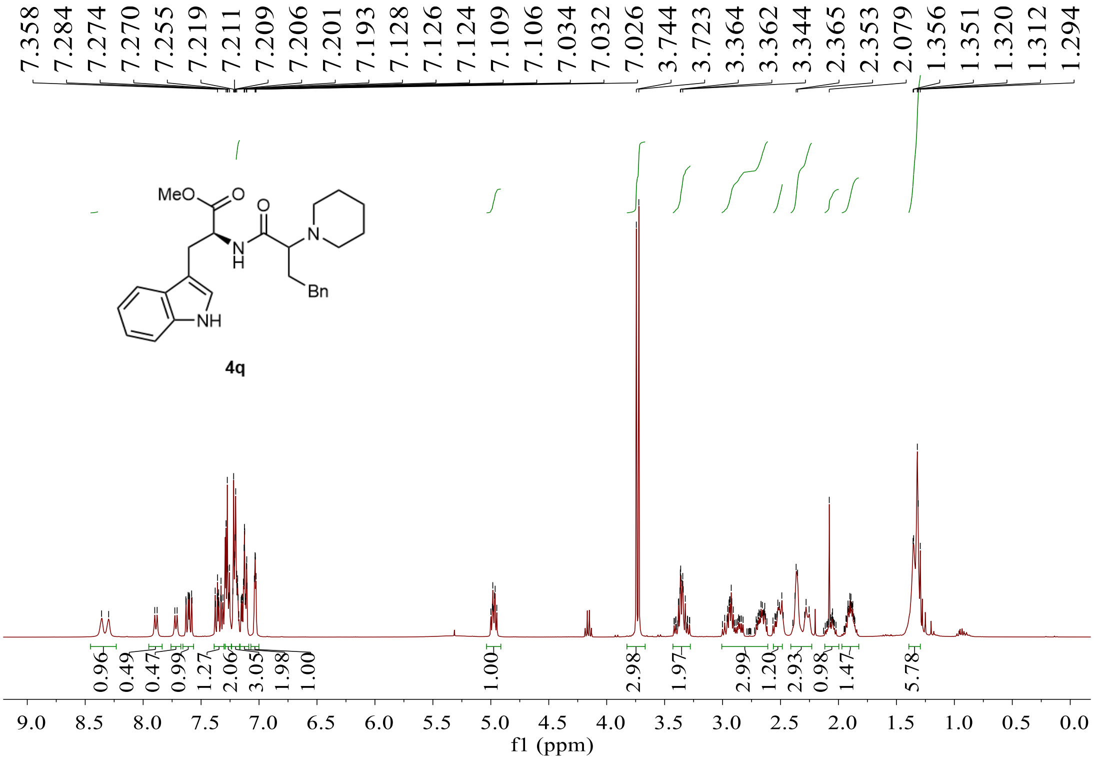
7.358 7.284 7.274 7.270 7.255 7.219 7.211 7.209 7.206 7.201 7.193 7.128 7.126 7.124 7.109 7.106 7.034 7.032 7.026 3.744 3.723 3.364 3.362 3.344 2.365 2.353 2.079 1.356 1.351 1.320 1.312 1.294
MeO O N H O N Bn NH
4q
0.96 0.49 0.47 0.99 1.27 2.06 3.05 1.98 1.00 1.00 2.98 1.97 2.99 1.20 2.93 0.98 1.47 5.78
9.0 8.5 8.0 7.5 7.0 6.5 6.0 5.5 5.0 4.5 4.0 3.5 3.0 2.5 2.0 1.5 1.0 0.5 0.0
f1 (ppm)

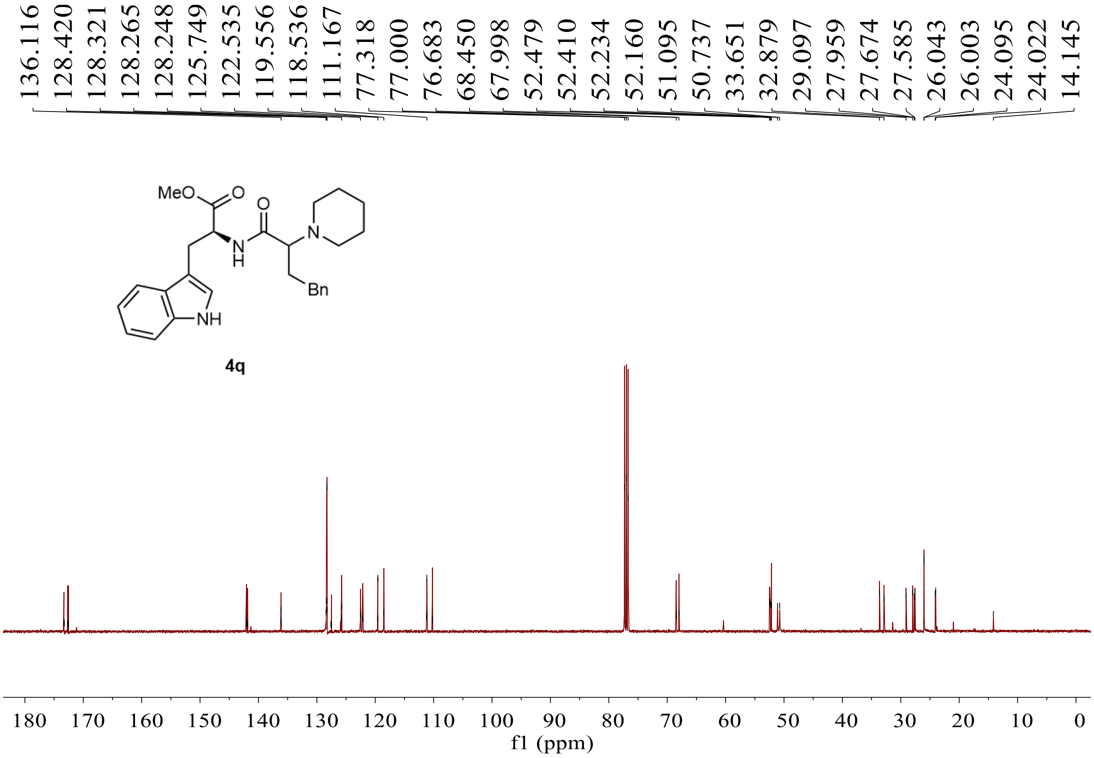
136.116 128.420 128.321 128.265 128.248 125.749 122.535 119.556 118.536 111.167 77.318 77.000 76.683 68.450 67.998 52.479 52.410 52.234 52.160 51.095 50.737 33.651 32.879 29.097 27.959 27.674 27.585 26.043 26.003 24.095 24.022 14.145
MeO O N H O N Bn NH
4q
180 170 160 150 140 130 120 110 100 90 80 70 60 50 40 30 20 10 0
f1 (ppm)

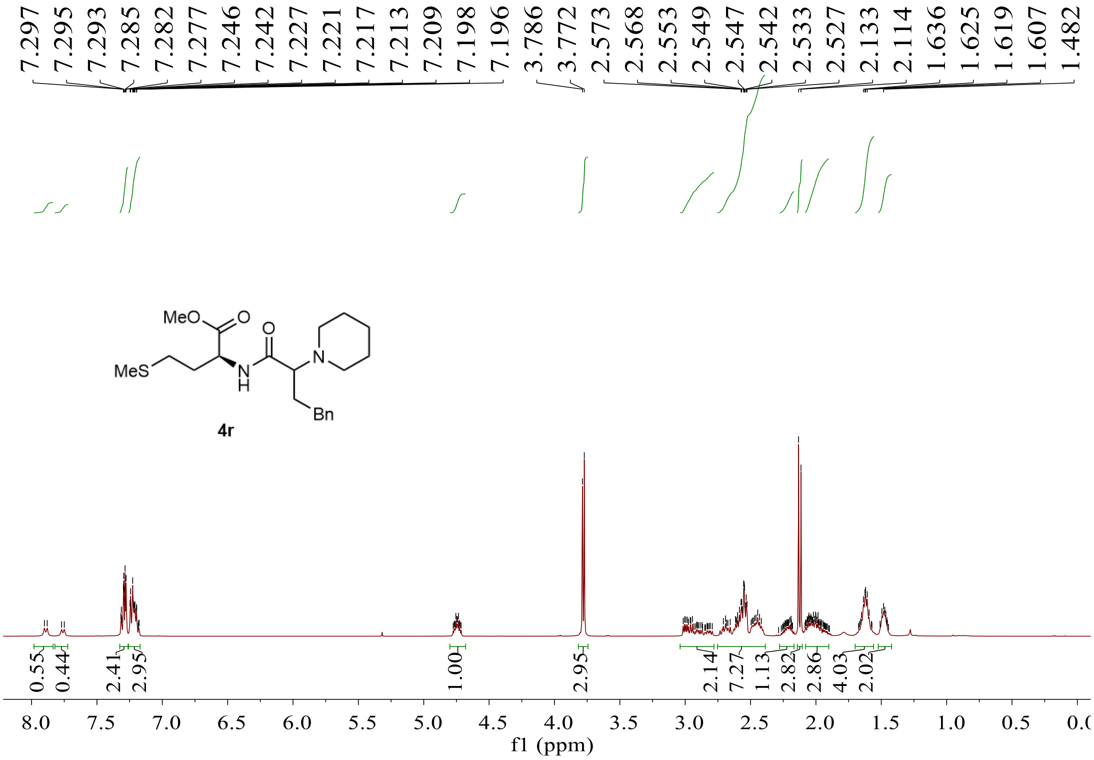
7.297 7.295 7.293 7.285 7.282 7.277 7.246 7.242 7.227 7.221 7.217 7.213 7.209 7.198 7.196 3.786 3.772 2.573 2.568 2.553 2.549 2.547 2.542 2.533 2.527 2.133 2.114 1.636 1.625 1.619 1.607 1.482
MeO
O
O
N
MeS
N
H
Bn
4r
0.55 0.44 2.41 2.95 1.00 2.95 2.14 7.27 1.13 2.82 2.86 4.03 2.02
f1 (ppm)

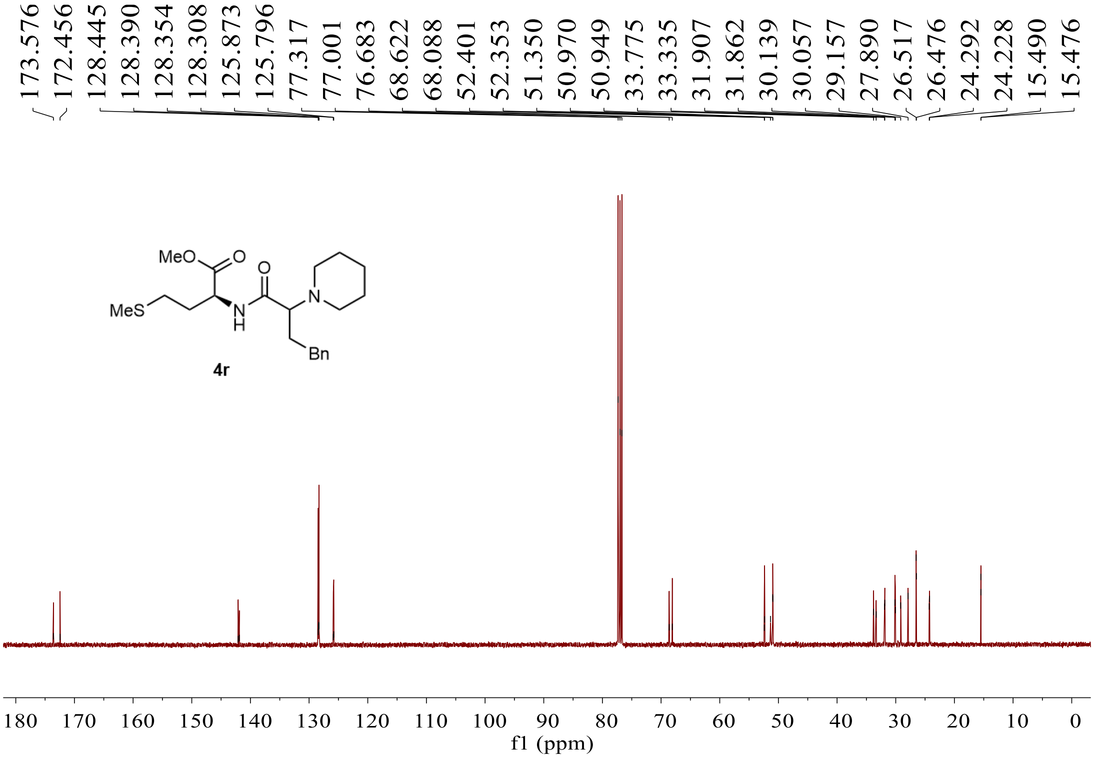
173.576 172.456 128.445 128.390 128.354 128.308 125.873 125.796 77.317 77.001 76.683 68.622 68.088 52.401 52.353 51.350 50.970 50.949 33.775 33.335 31.907 31.862 30.139 30.057 29.157 27.890 26.517 26.476 24.292 24.228 15.490 15.476
MeO
O
O
N
MeS
N
H
Bn
4r
f1 (ppm)

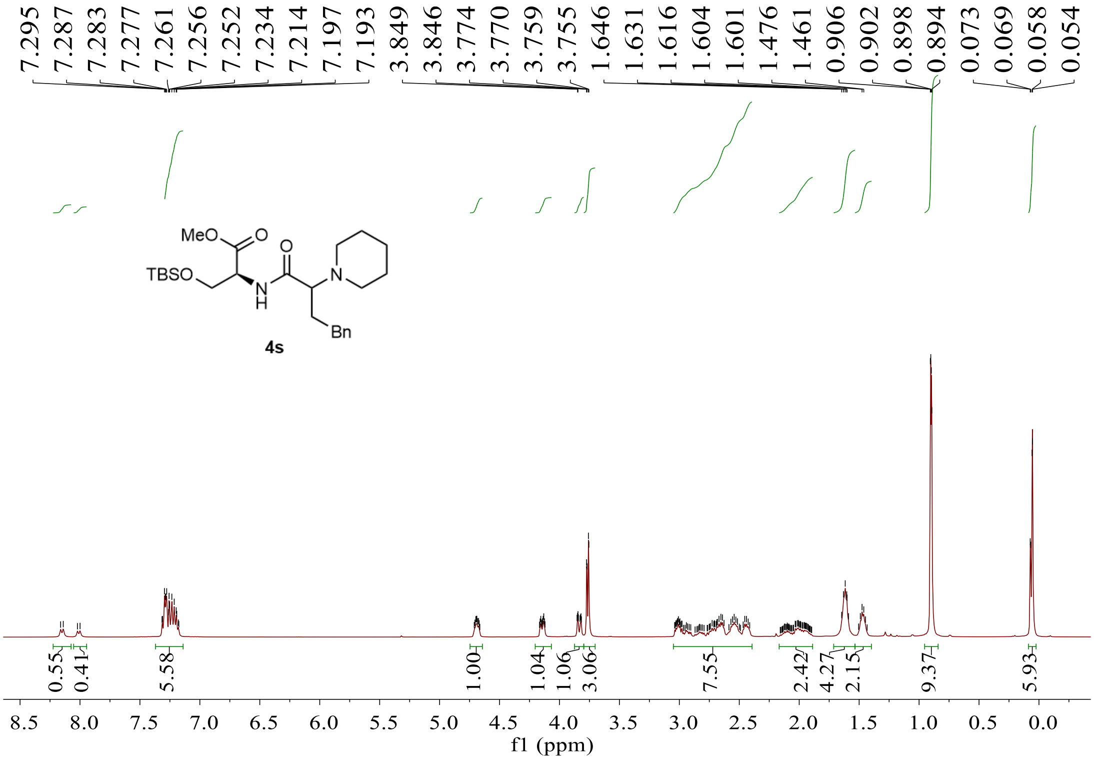
7.295 7.287 7.283 7.277 7.261 7.256 7.252 7.234 7.214 7.197 7.193 3.849 3.846 3.774 3.770 3.759 3.755 1.646 1.631 1.616 1.604 1.601 1.476 1.461 0.906 0.902 0.898 0.894 0.073 0.069 0.058 0.054
MeO
O
TBSO
N
H
O
N
Bn
4s
0.55 0.41 5.58 1.00 1.04 1.06 3.06 7.55 2.42 4.27 2.15 9.37 5.93
8.5 8.0 7.5 7.0 6.5 6.0 5.5 5.0 4.5 4.0 3.5 3.0 2.5 2.0 1.5 1.0 0.5 0.0
f1 (ppm)

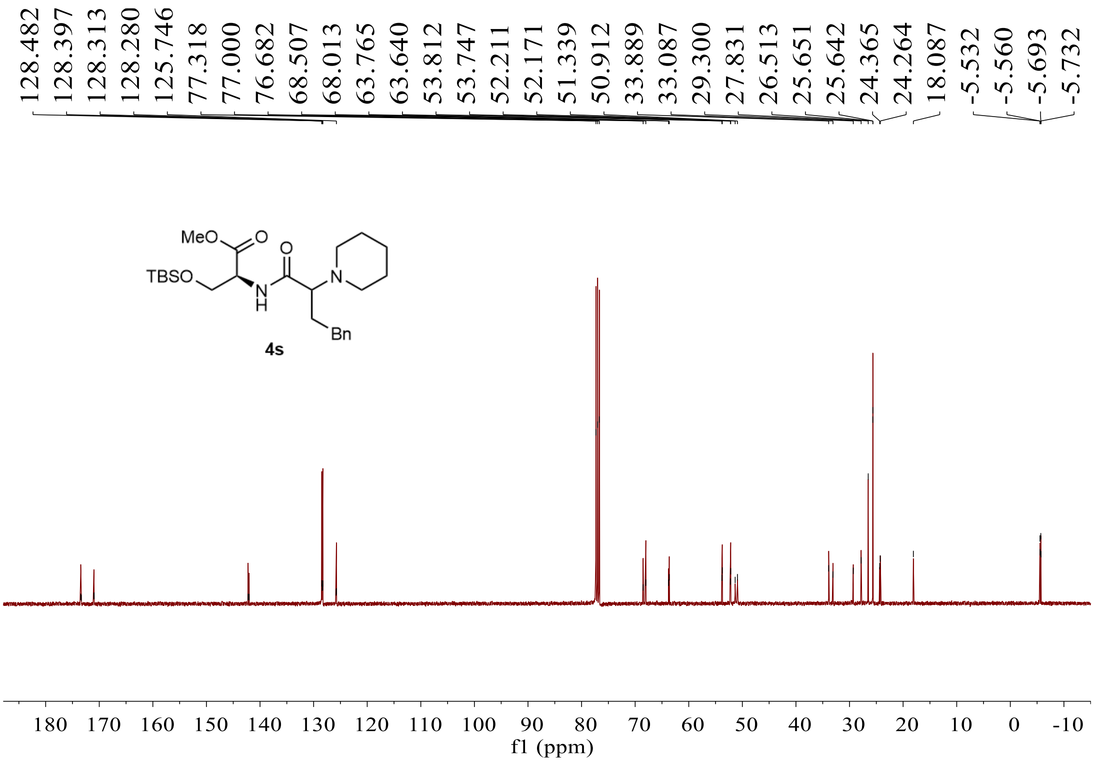
128.482 128.397 128.313 128.280 125.746 77.318 77.000 76.682 68.507 68.013 63.765 63.640 53.812 53.747 52.211 52.171 51.339 50.912 33.889 33.087 29.300 27.831 26.513 25.651 25.642 24.365 24.264 18.087 -5.532 -5.560 -5.693 -5.732
MeO
O
TBSO
N
H
O
N
Bn
4s
180 170 160 150 140 130 120 110 100 90 80 70 60 50 40 30 20 10 0 -10
f1 (ppm)

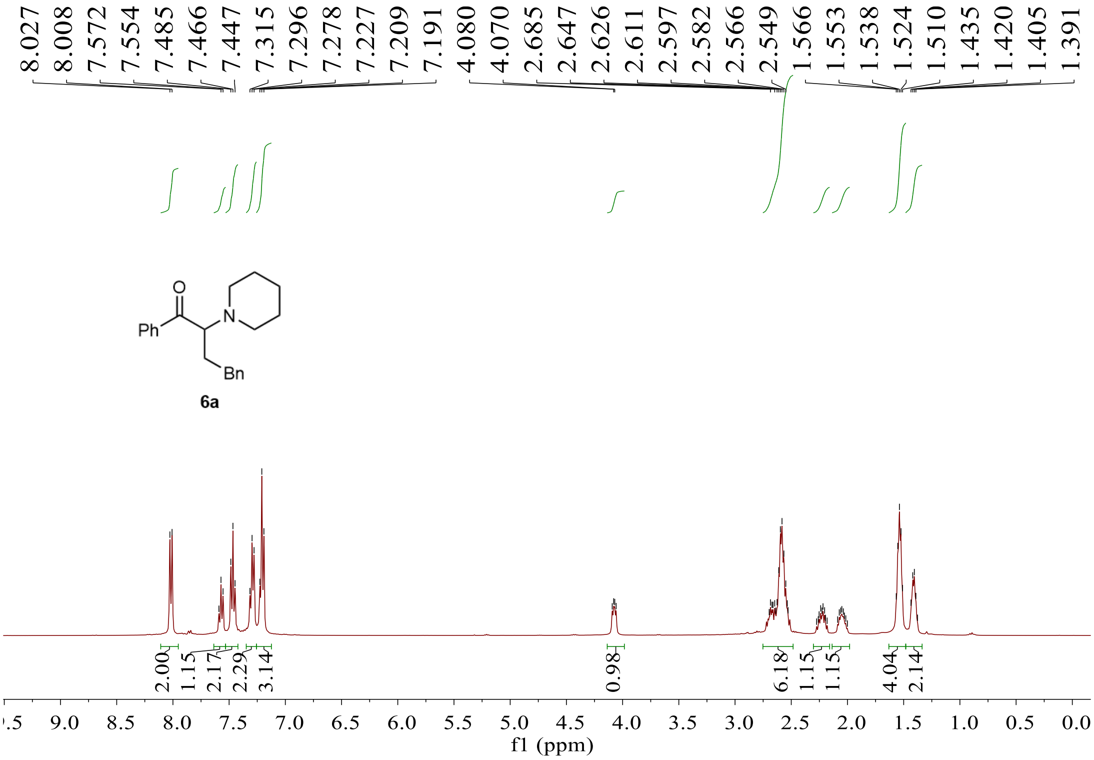
8.027 8.008 7.572 7.554 7.485 7.466 7.447 7.315 7.296 7.278 7.227 7.209 7.191 4.080 4.070 2.685 2.647 2.626 2.611 2.597 2.582 2.566 2.549 1.566 1.553 1.538 1.524 1.510 1.435 1.420 1.405 1.391
O
Ph
N
Bn
6a
2.00 1.15 2.17 2.29 3.14 0.98 6.18 1.15 1.15 4.04 2.14
9.0 8.5 8.0 7.5 7.0 6.5 6.0 5.5 5.0 4.5 4.0 3.5 3.0 2.5 2.0 1.5 1.0 0.5 0.0
f1 (ppm)

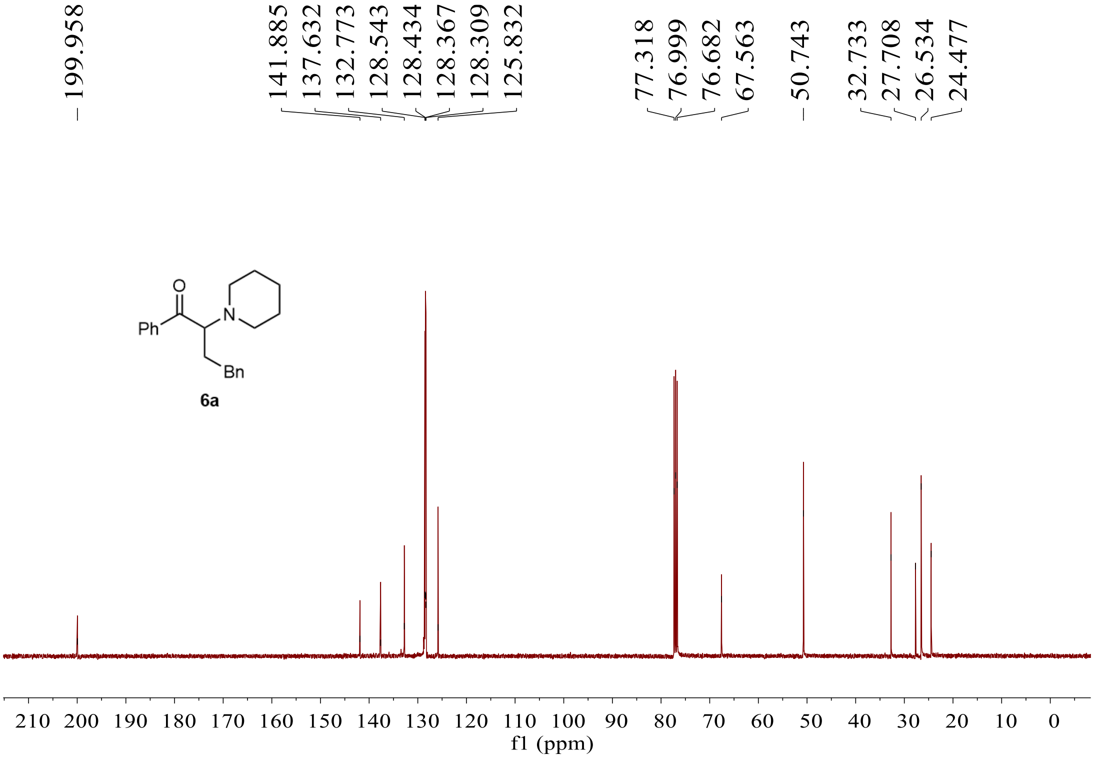
199.958
141.885 137.632 132.773 128.543 128.434 128.367 128.309 125.832
77.318 76.999 76.682 67.563
50.743
32.733 27.708 26.534 24.477
O
Ph
N
Bn
6a
210 200 190 180 170 160 150 140 130 120 110 100 90 80 70 60 50 40 30 20 10 0
f1 (ppm)

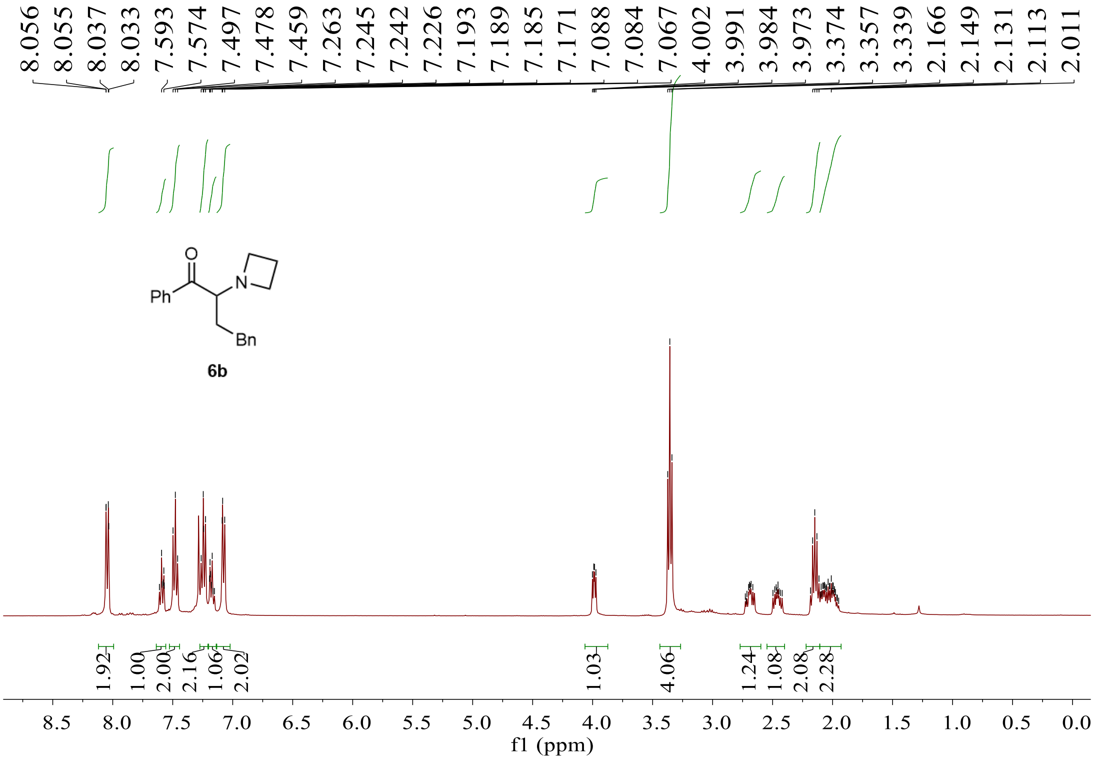

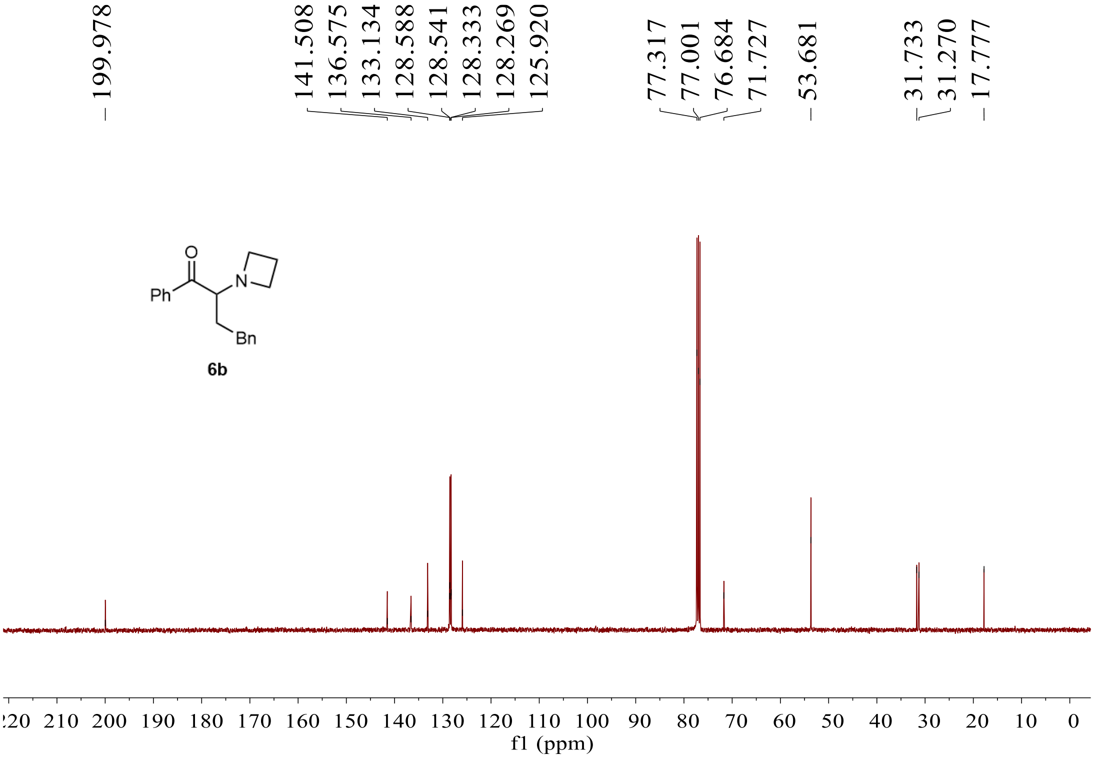

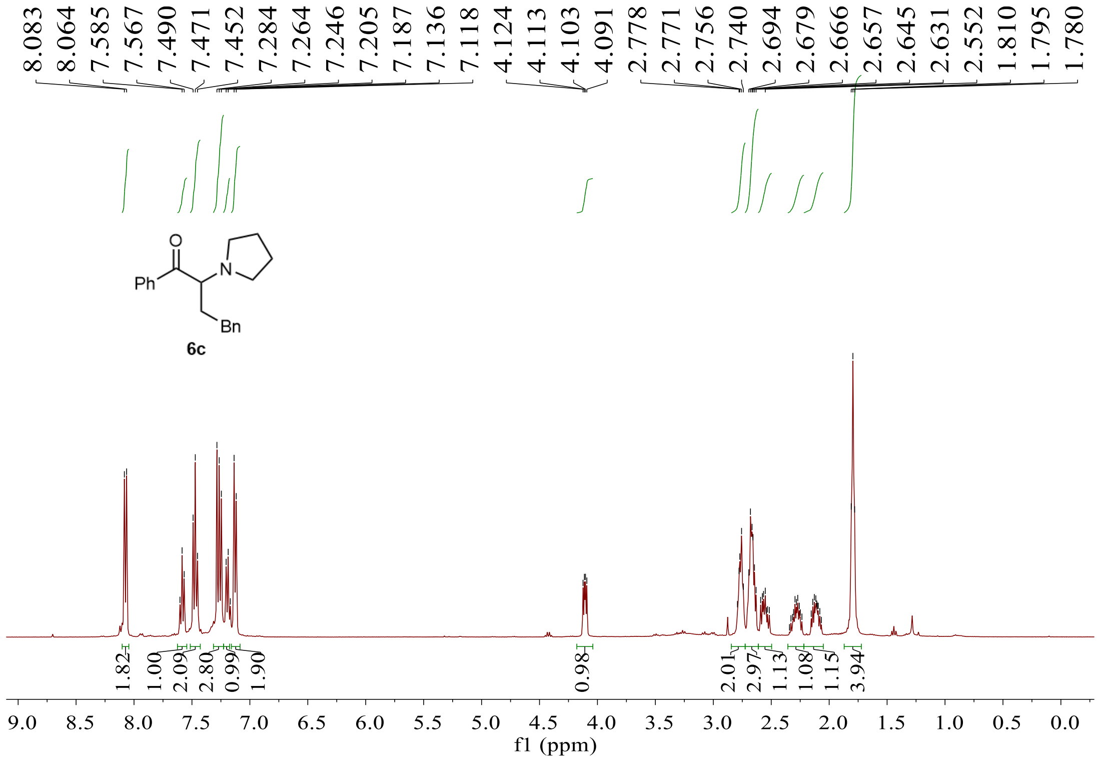

8.083
8.064
7.585
7.567
7.490
7.471
7.452
7.284
7.264
7.246
7.205
7.187
7.136
7.118
4.124
4.113
4.103
4.091
2.778
2.771
2.756
2.740
2.694
2.679
2.666
2.657
2.645
2.631
2.552
1.810
1.795
1.780
O
Ph
N
Bn
6c
1.82
1.00
2.09
2.80
0.99
1.90
0.98
2.01
2.97
1.13
1.08
1.15
3.94
9.0 8.5 8.0 7.5 7.0 6.5 6.0 5.5 5.0 4.5 4.0 3.5 3.0 2.5 2.0 1.5 1.0 0.5 0.0
f1 (ppm)

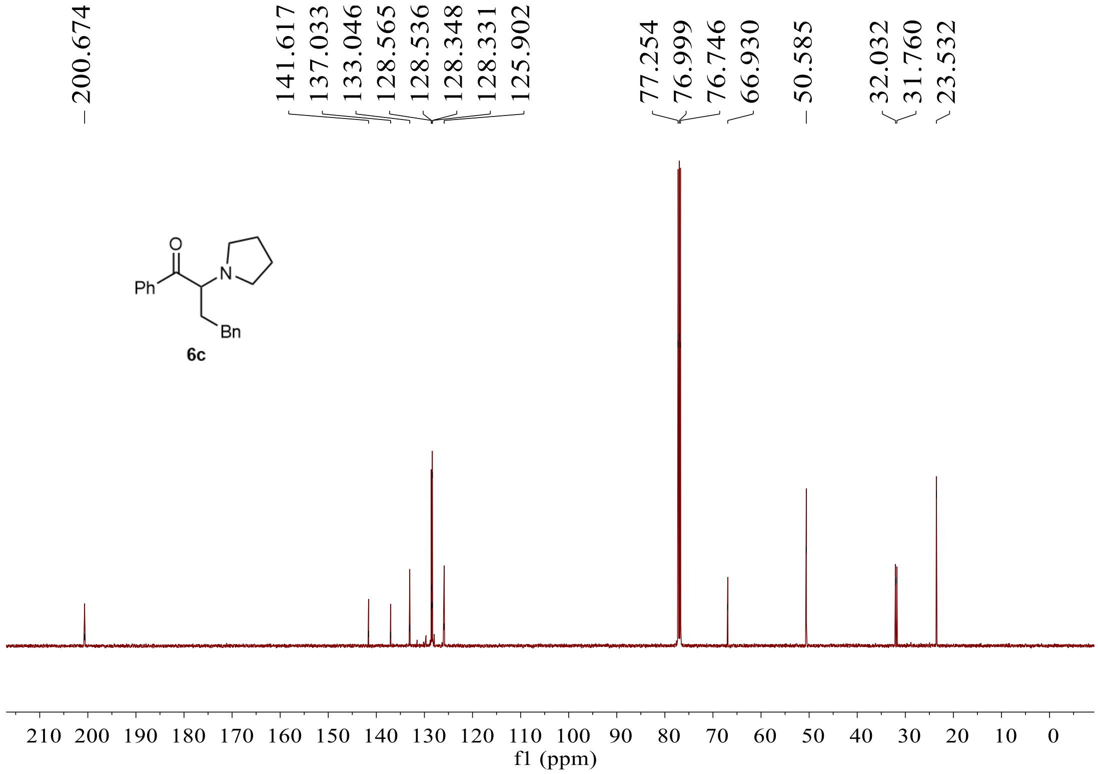

200.674
141.617
137.033
133.046
128.565
128.536
128.348
128.331
125.902
77.254
76.999
76.746
66.930
50.585
32.032
31.760
23.532
O
Ph
N
Bn
6c
210 200 190 180 170 160 150 140 130 120 110 100 90 80 70 60 50 40 30 20 10 0
f1 (ppm)

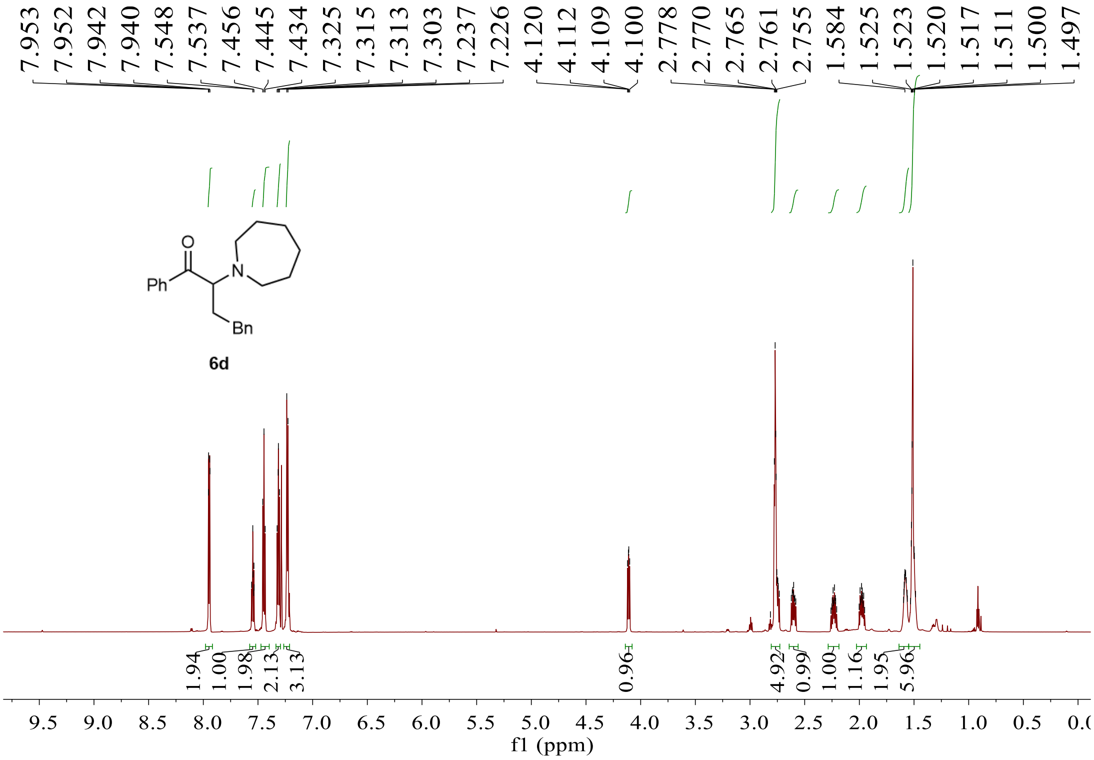
7.953 7.952 7.942 7.940 7.548 7.537 7.456 7.445 7.434 7.325 7.315 7.313 7.303 7.237 7.226
4.120 4.112 4.109 4.100
2.778 2.770 2.765 2.761 2.755
1.584 1.525 1.523 1.520 1.517 1.511 1.500 1.497
O
N
Ph
Bn
6d
1.94 1.00 1.98 2.13 3.13 0.96 4.92 0.99 1.00 1.16 1.95 5.96
9.5 9.0 8.5 8.0 7.5 7.0 6.5 6.0 5.5 5.0 4.5 4.0 3.5 3.0 2.5 2.0 1.5 1.0 0.5 0.0
f1 (ppm)

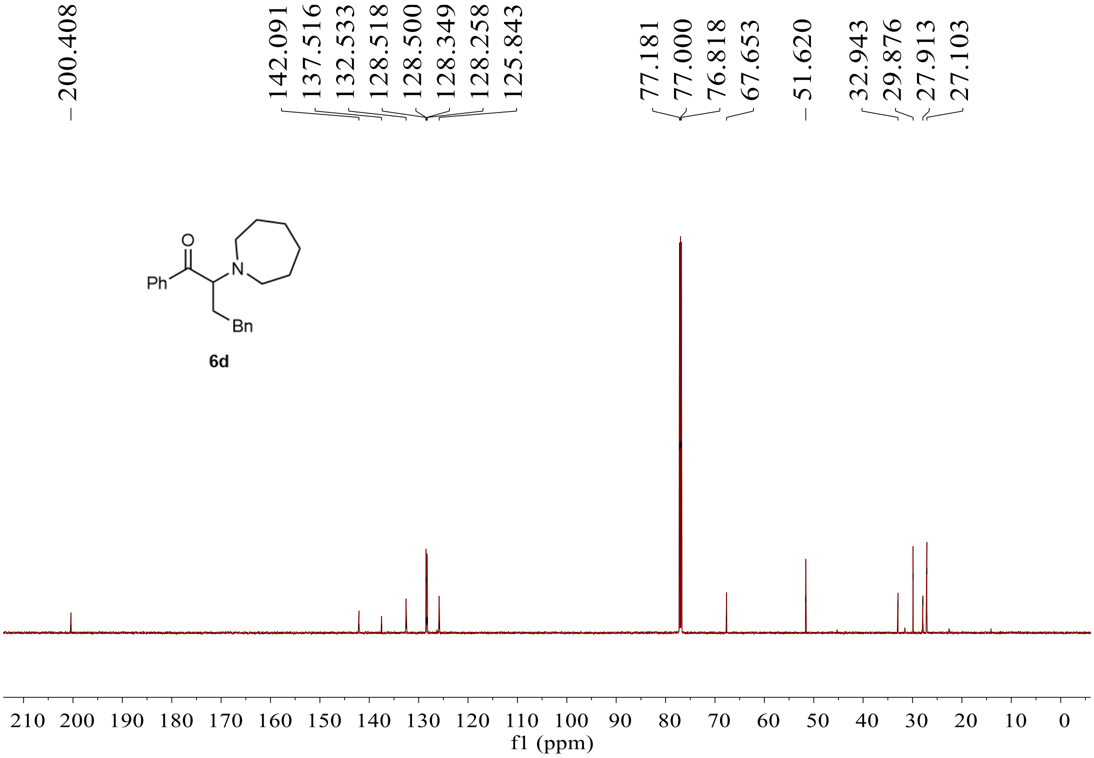
200.408
142.091 137.516 132.533 128.518 128.500 128.349 128.258 125.843
77.181 77.000 76.818 67.653
51.620
32.943 29.876 27.913 27.103
O
N
Ph
Bn
6d
210 200 190 180 170 160 150 140 130 120 110 100 90 80 70 60 50 40 30 20 10 0
f1 (ppm)

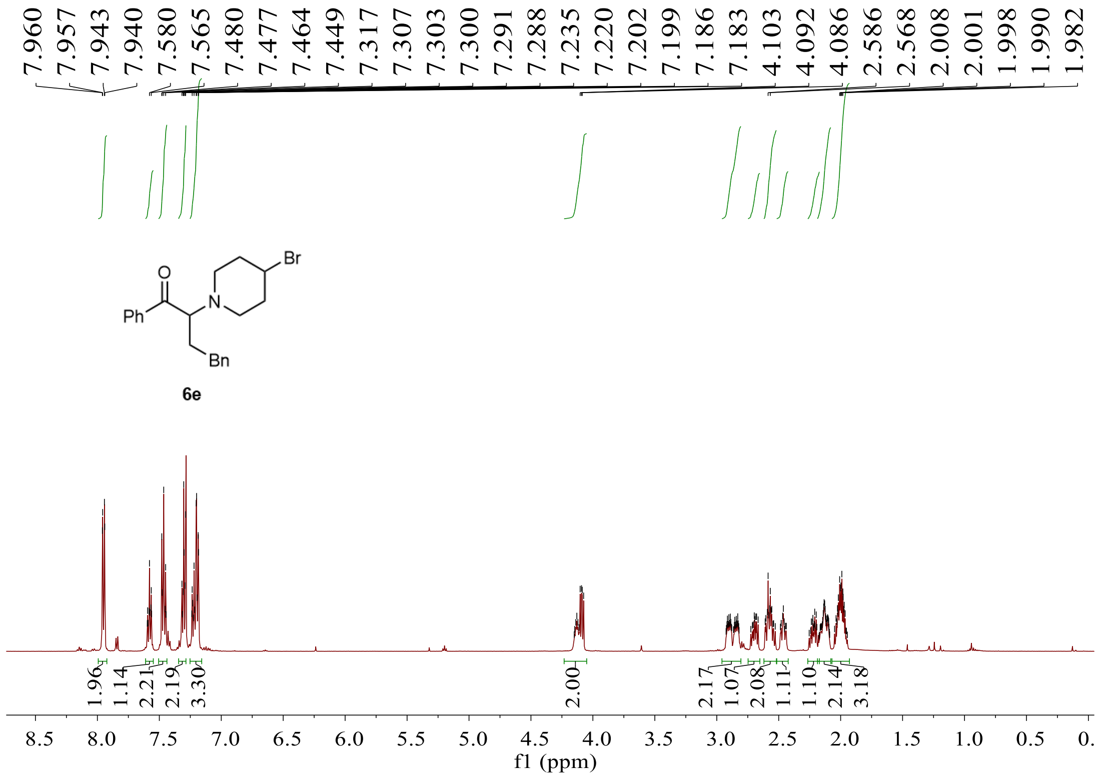
7.960
7.957
7.943
7.940
7.580
7.565
7.480
7.477
7.464
7.449
7.317
7.307
7.303
7.300
7.291
7.288
7.235
7.220
7.202
7.199
7.186
7.183
4.103
4.092
4.086
2.586
2.568
2.008
2.001
1.998
1.990
1.982
O
Br
Ph
N
Bn
6e
1.96
1.14
2.21
2.19
3.30
2.00
2.17
1.07
2.08
1.11
1.10
2.14
3.18
8.5 8.0 7.5 7.0 6.5 6.0 5.5 5.0 4.5 4.0 3.5 3.0 2.5 2.0 1.5 1.0 0.5 0.
f1 (ppm)

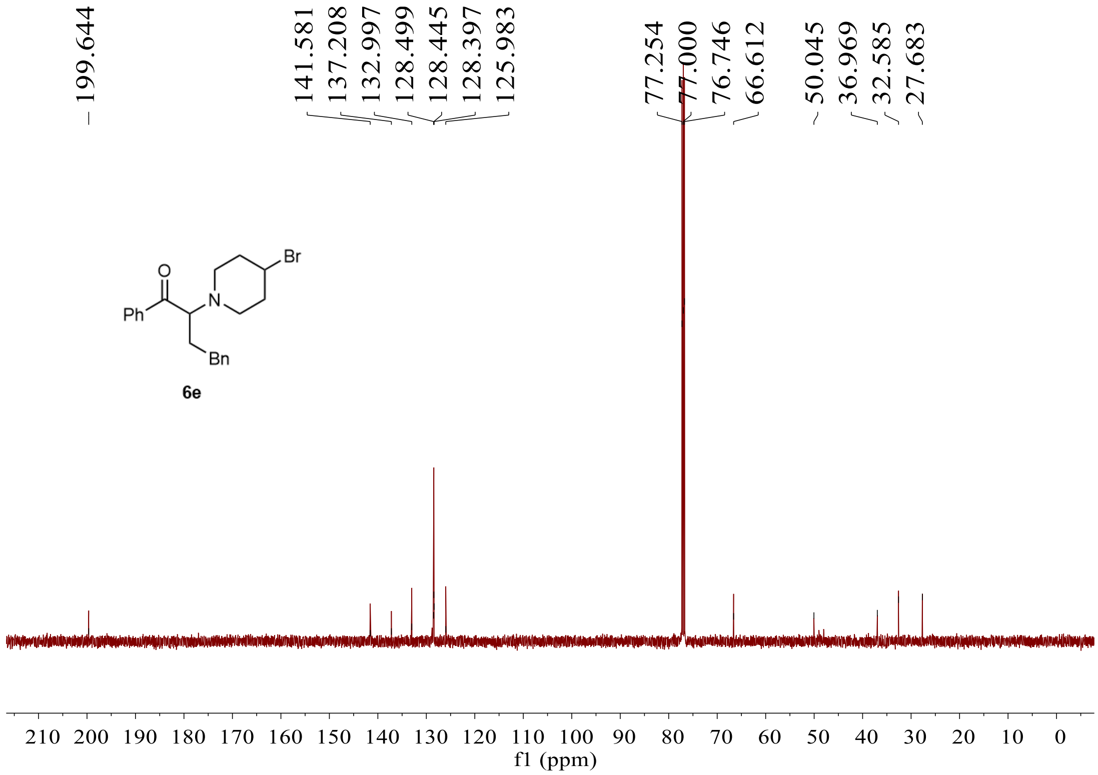
199.644
141.581
137.208
132.997
128.499
128.445
128.397
125.983
77.254
77.000
76.746
66.612
50.045
36.969
32.585
27.683
O
Br
Ph
N
Bn
6e
210 200 190 180 170 160 150 140 130 120 110 100 90 80 70 60 50 40 30 20 10 0
f1 (ppm)

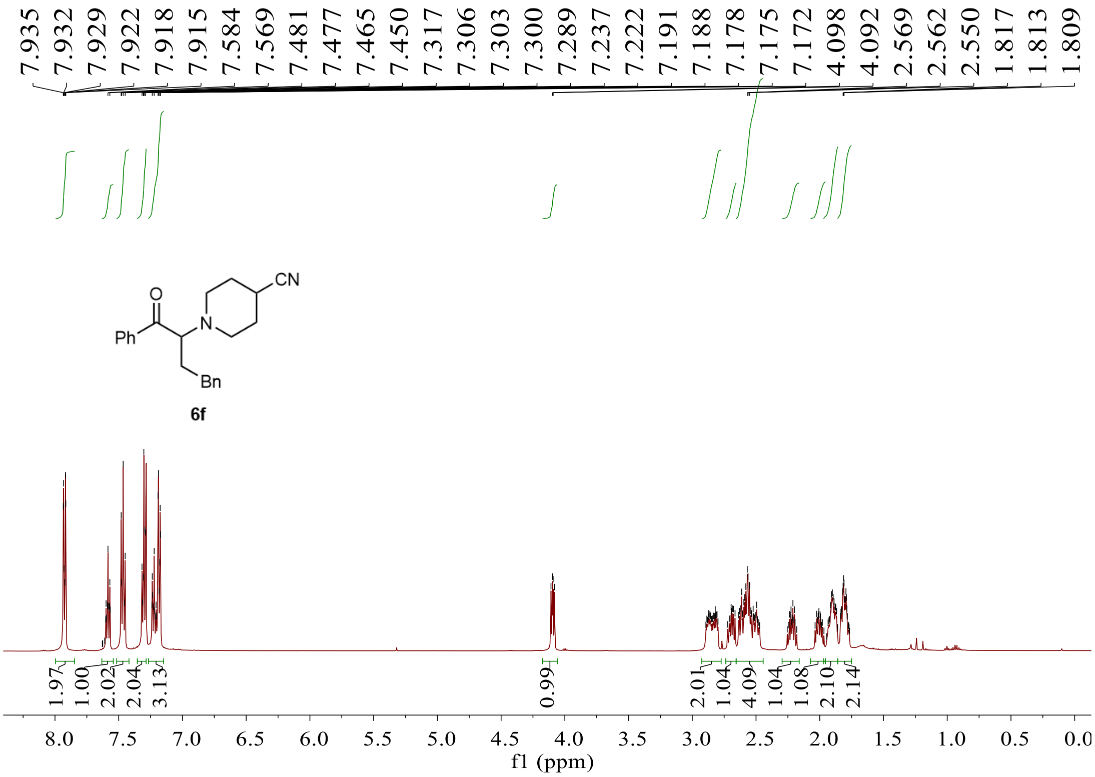
7.935 7.932 7.929 7.922 7.918 7.915 7.584 7.569 7.481 7.477 7.465 7.450 7.317 7.306 7.303 7.300 7.289 7.237 7.222 7.191 7.188 7.178 7.175 7.172 4.098 4.092 2.569 2.562 2.550 1.817 1.813 1.809
CN
O
N
Ph
Bn
6f
1.97 1.00 2.02 2.04 3.13 0.99 2.01 1.04 4.09 1.04 1.08 2.10 2.14
8.0 7.5 7.0 6.5 6.0 5.5 5.0 4.5 4.0 3.5 3.0 2.5 2.0 1.5 1.0 0.5 0.0
f1 (ppm)

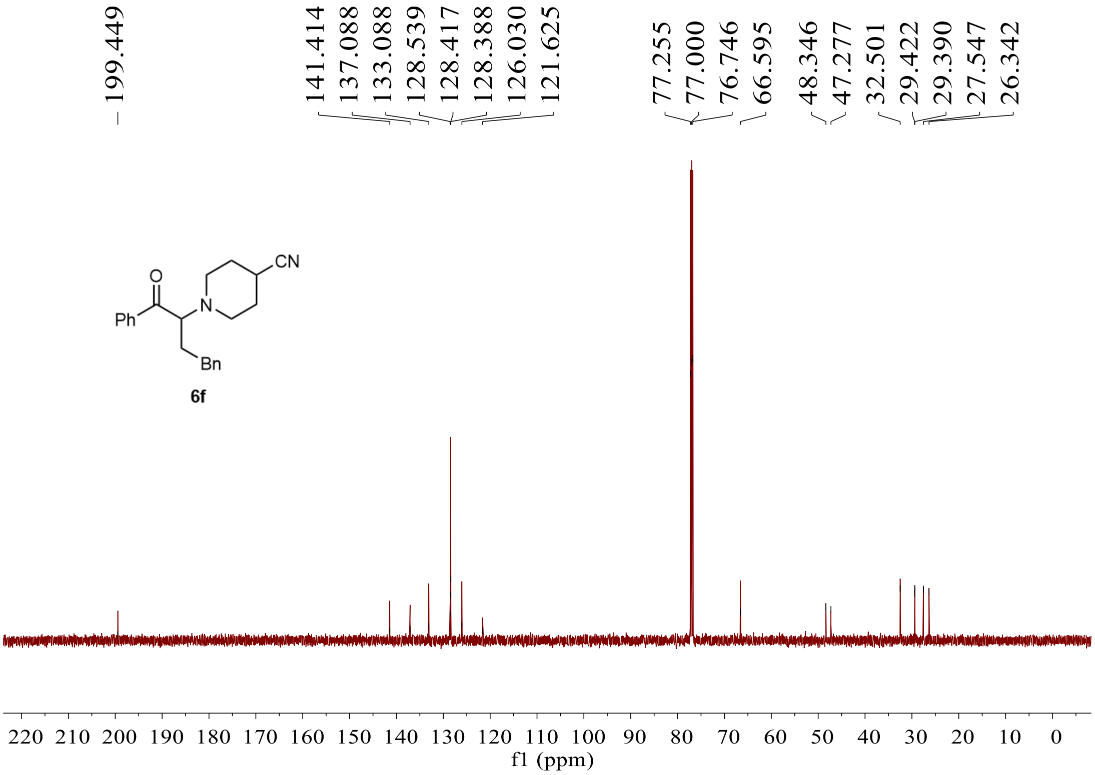
199.449
141.414 137.088 133.088 128.539 128.417 128.388 126.030 121.625
77.255 77.000 76.746 66.595
48.346 47.277
32.501 29.422 29.390 27.547 26.342
CN
O
N
Ph
Bn
6f
220 210 200 190 180 170 160 150 140 130 120 110 100 90 80 70 60 50 40 30 20 10 0
f1 (ppm)

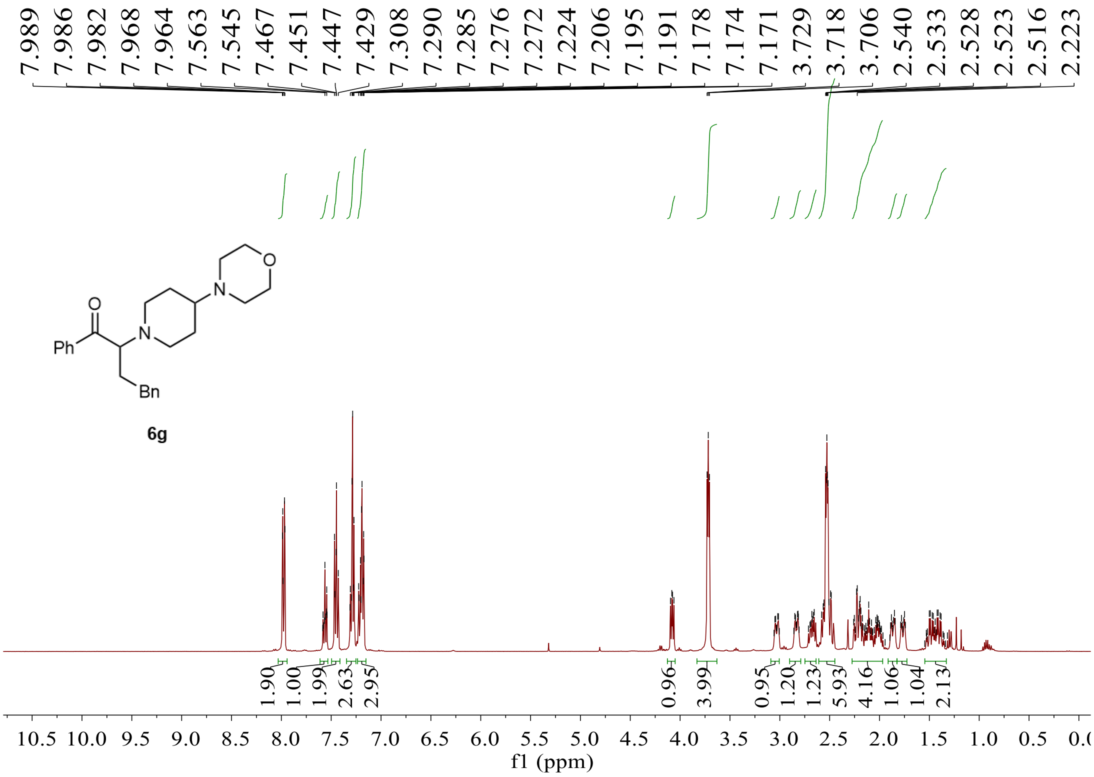
7.989
7.986
7.982
7.968
7.964
7.563
7.545
7.467
7.451
7.447
7.429
7.308
7.290
7.285
7.276
7.272
7.224
7.206
7.195
7.191
7.178
7.174
7.171
3.729
3.718
3.706
2.540
2.533
2.528
2.523
2.516
2.223
O
N
N
O
Ph
Bn
6g
1.90
1.00
1.99
2.63
2.95
0.96
3.99
0.95
1.20
1.23
5.93
4.16
1.06
1.04
2.13
10.5 10.0 9.5 9.0 8.5 8.0 7.5 7.0 6.5 6.0 5.5 5.0 4.5 4.0 3.5 3.0 2.5 2.0 1.5 1.0 0.5 0.0
f1 (ppm)

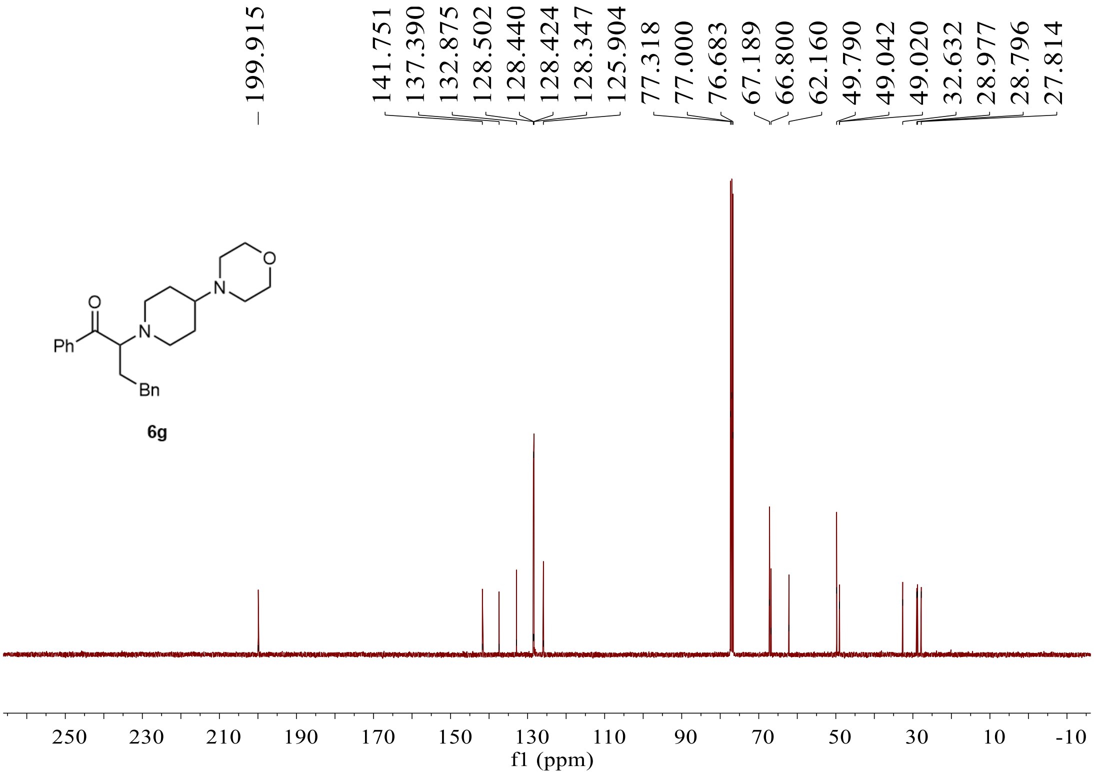
199.915
141.751
137.390
132.875
128.502
128.440
128.424
128.347
125.904
77.318
77.000
76.683
67.189
66.800
62.160
49.790
49.042
49.020
32.632
28.977
28.796
27.814
O
N
N
O
Ph
Bn
6g
250 230 210 190 170 150 130 110 90 70 50 30 10 -10
f1 (ppm)

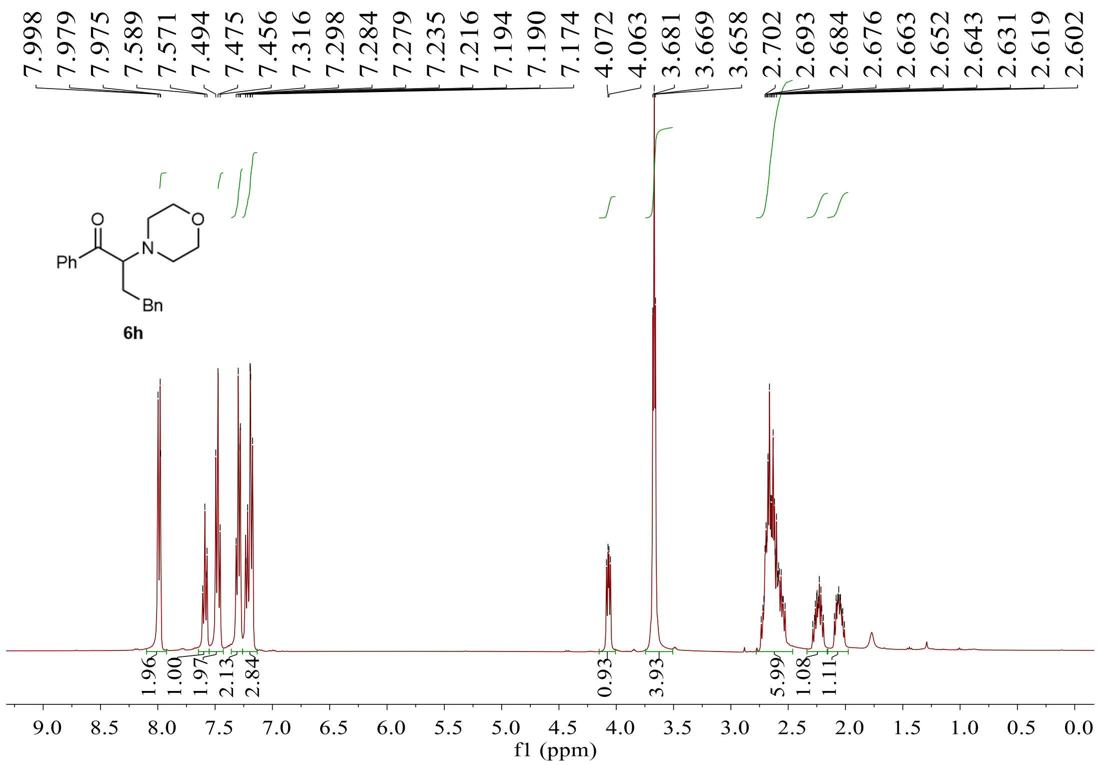
7.998
7.979
7.975
7.589
7.571
7.494
7.475
7.456
7.316
7.298
7.284
7.279
7.235
7.216
7.194
7.190
7.174
4.072
4.063
3.681
3.669
3.658
2.702
2.693
2.684
2.676
2.663
2.652
2.643
2.631
2.619
2.602
Ph
O
N
O
Bn
6h
1.96
1.00
1.97
2.13
2.84
0.93
3.93
5.99
1.08
1.11
9.0 8.5 8.0 7.5 7.0 6.5 6.0 5.5 5.0 4.5 4.0 3.5 3.0 2.5 2.0 1.5 1.0 0.5 0.0
f1 (ppm)

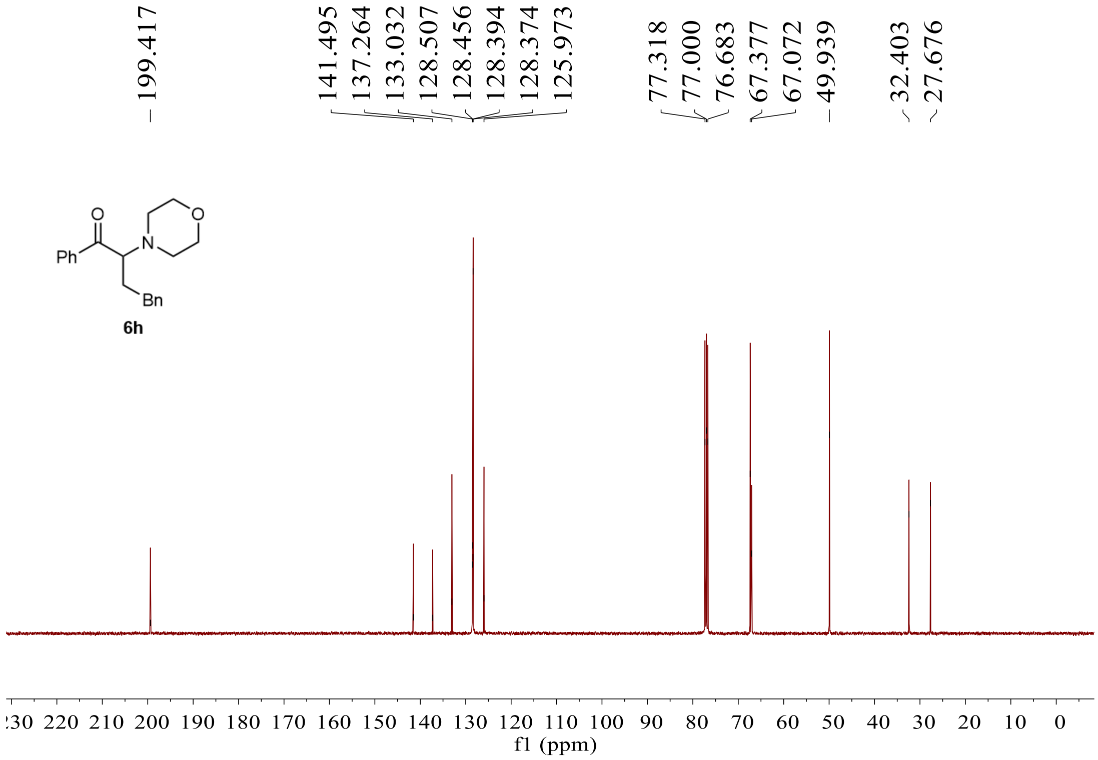
199.417
141.495
137.264
133.032
128.507
128.456
128.394
128.374
125.973
77.318
77.000
76.683
67.377
67.072
49.939
32.403
27.676
Ph
O
N
O
Bn
6h
230 220 210 200 190 180 170 160 150 140 130 120 110 100 90 80 70 60 50 40 30 20 10 0
f1 (ppm)

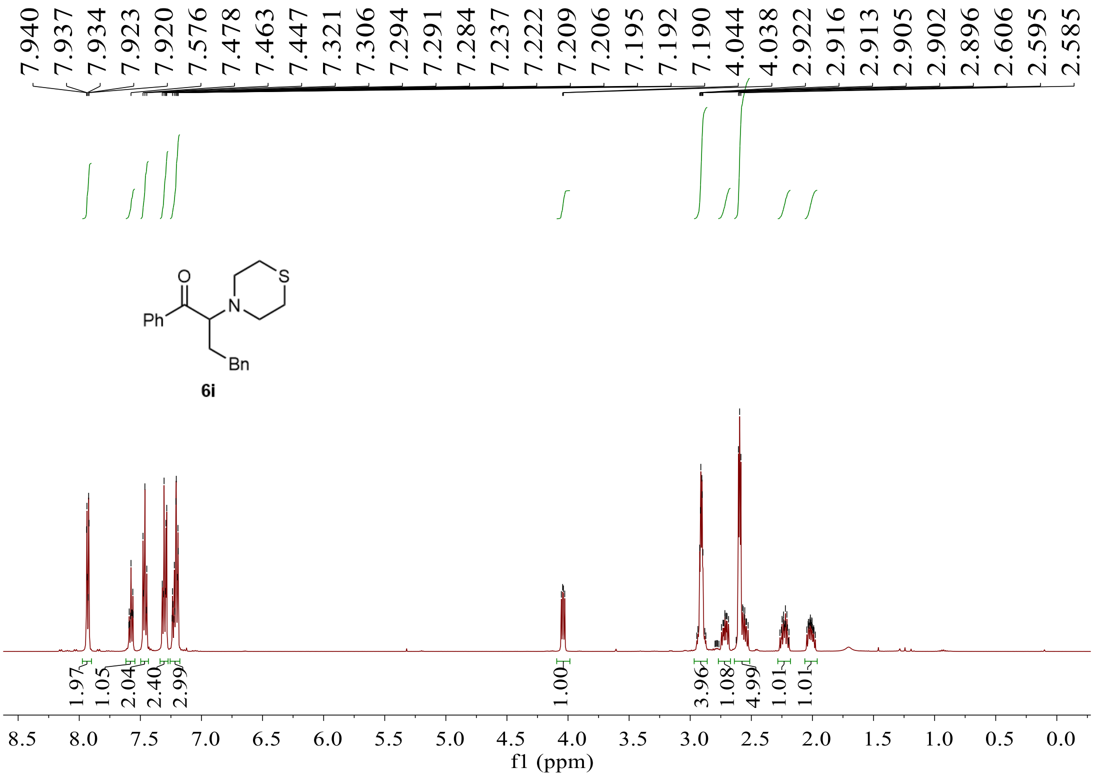
7.940
7.937
7.934
7.923
7.920
7.576
7.478
7.463
7.447
7.321
7.306
7.294
7.291
7.284
7.237
7.222
7.209
7.206
7.195
7.192
7.190
4.044
4.038
2.922
2.916
2.913
2.905
2.902
2.896
2.606
2.595
2.585
O
N
S
Ph
Bn
6i
1.97
1.05
2.04
2.40
2.99
1.00
3.96
1.08
4.99
1.01
1.01
8.5 8.0 7.5 7.0 6.5 6.0 5.5 5.0 4.5 4.0 3.5 3.0 2.5 2.0 1.5 1.0 0.5 0.0
f1 (ppm)

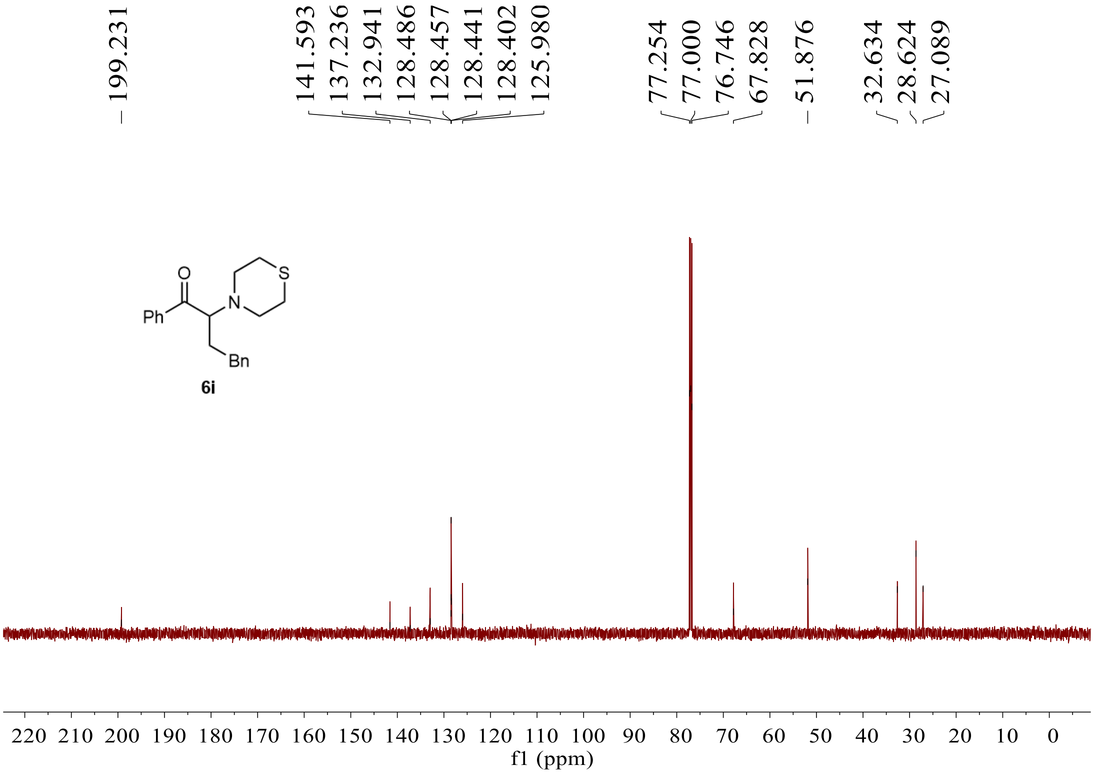
199.231
141.593
137.236
132.941
128.486
128.457
128.441
128.402
125.980
77.254
77.000
76.746
67.828
51.876
32.634
28.624
27.089
O
N
S
Ph
Bn
6i
220 210 200 190 180 170 160 150 140 130 120 110 100 90 80 70 60 50 40 30 20 10 0
f1 (ppm)

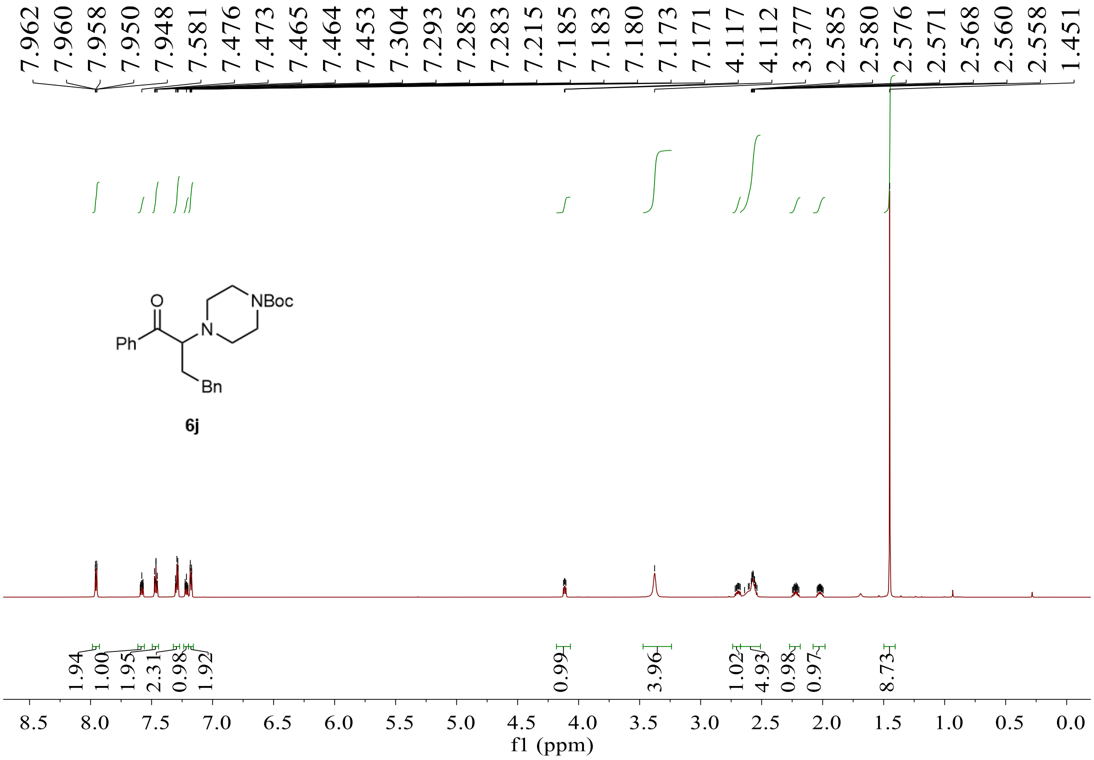
7.962
7.960
7.958
7.950
7.948
7.581
7.476
7.473
7.465
7.464
7.453
7.304
7.293
7.285
7.283
7.215
7.185
7.183
7.180
7.173
7.171
4.117
4.112
3.377
2.585
2.580
2.576
2.571
2.568
2.560
2.558
1.451
O
Ph
N
NBoc
Bn
6j
1.94
1.00
1.95
2.31
0.98
1.92
0.99
3.96
1.02
4.93
0.98
0.97
8.73
8.5 8.0 7.5 7.0 6.5 6.0 5.5 5.0 4.5 4.0 3.5 3.0 2.5 2.0 1.5 1.0 0.5 0.0
f1 (ppm)

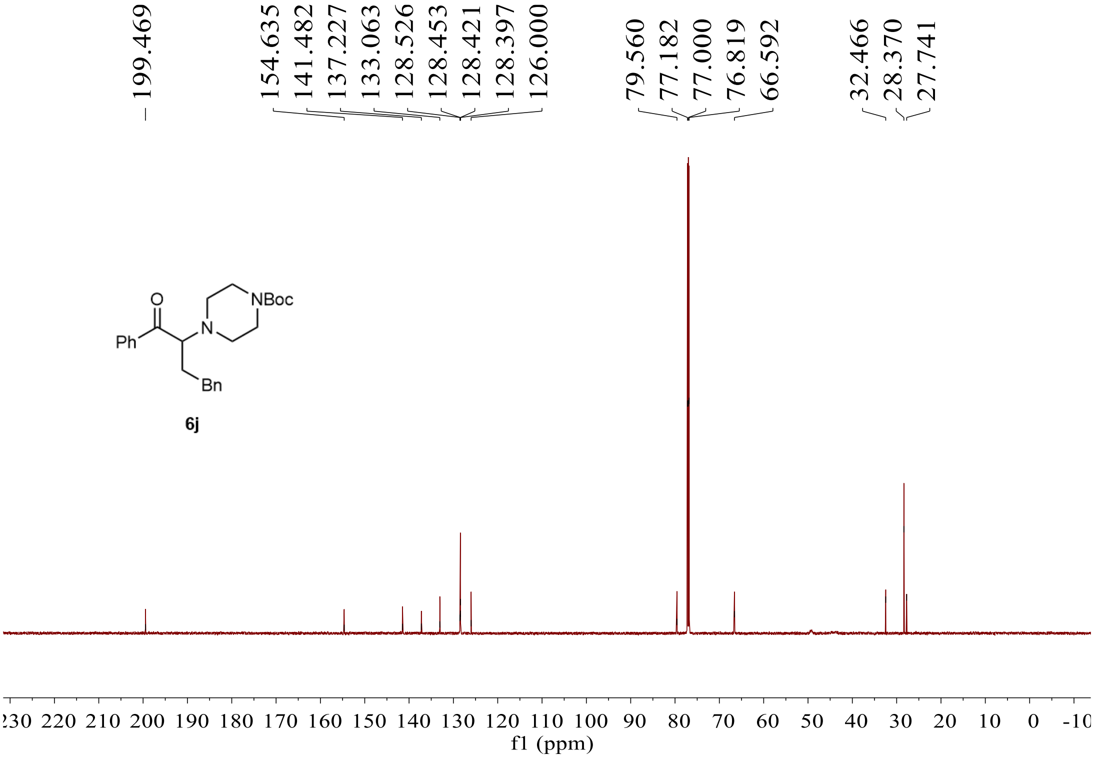
199.469
154.635
141.482
137.227
133.063
128.526
128.453
128.421
128.397
126.000
79.560
77.182
77.000
76.819
66.592
32.466
28.370
27.741
O
Ph
N
NBoc
Bn
6j
230 220 210 200 190 180 170 160 150 140 130 120 110 100 90 80 70 60 50 40 30 20 10 0 -10
f1 (ppm)

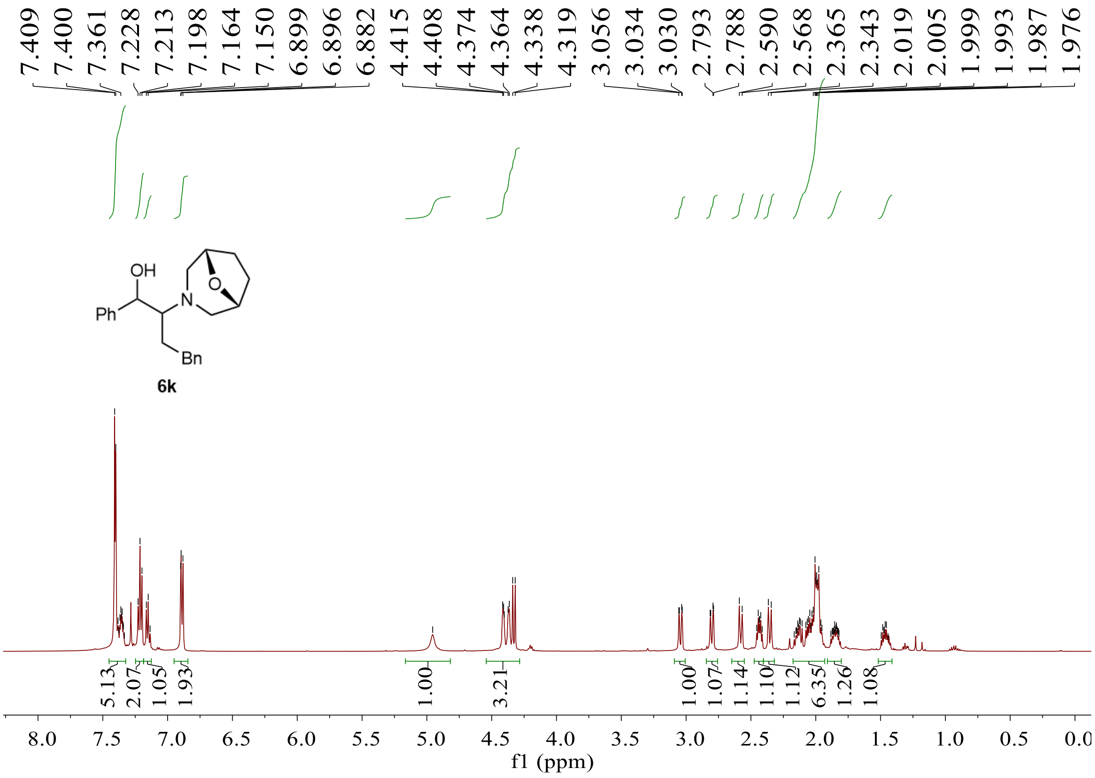
7.409 7.400 7.361 7.228 7.213 7.198 7.164 7.150 6.899 6.896 6.882 4.415 4.408 4.374 4.364 4.338 4.319 3.056 3.034 3.030 2.793 2.788 2.590 2.568 2.365 2.343 2.019 2.005 1.999 1.993 1.987 1.976
OH
Ph
N
O
Bn
6k
5.13 2.07 1.05 1.93 1.00 3.21 1.00 1.07 1.14 1.10 1.12 6.35 1.26 1.08
8.0 7.5 7.0 6.5 6.0 5.5 5.0 4.5 4.0 3.5 3.0 2.5 2.0 1.5 1.0 0.5 0.0
f1 (ppm)

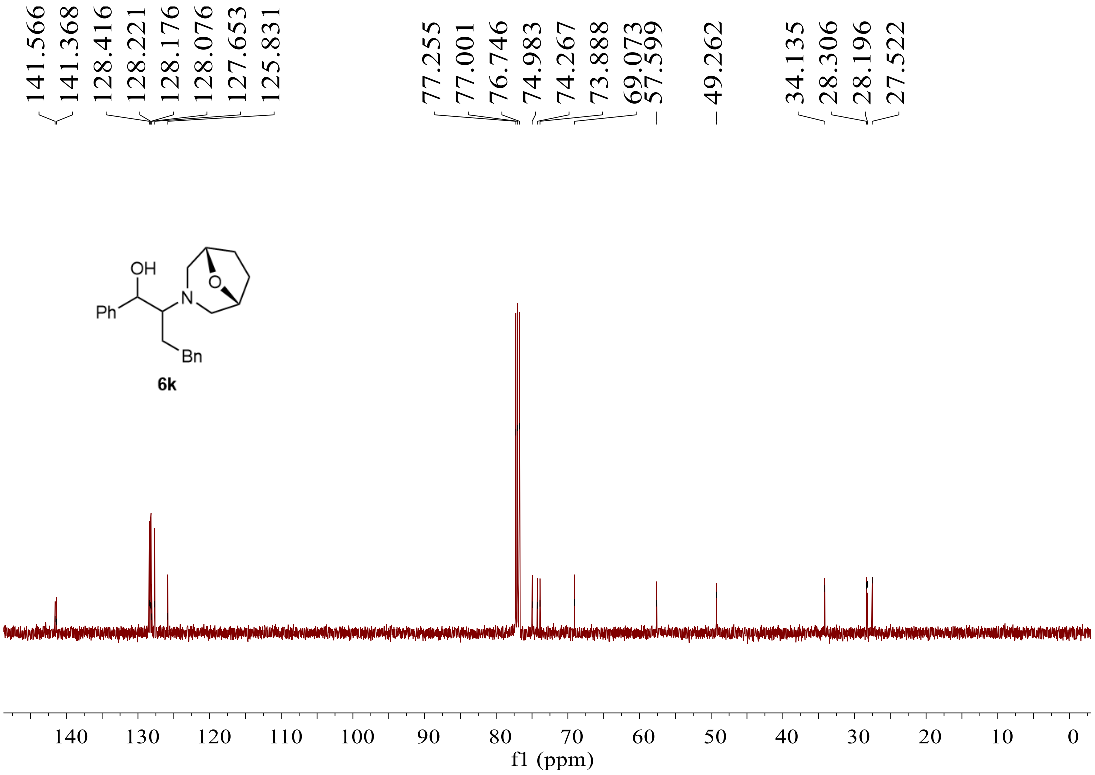
141.566 141.368 128.416 128.221 128.176 128.076 127.653 125.831 77.255 77.001 76.746 74.983 74.267 73.888 69.073 57.599 49.262 34.135 28.306 28.196 27.522
OH
Ph
N
O
Bn
6k
140 130 120 110 100 90 80 70 60 50 40 30 20 10 0
f1 (ppm)

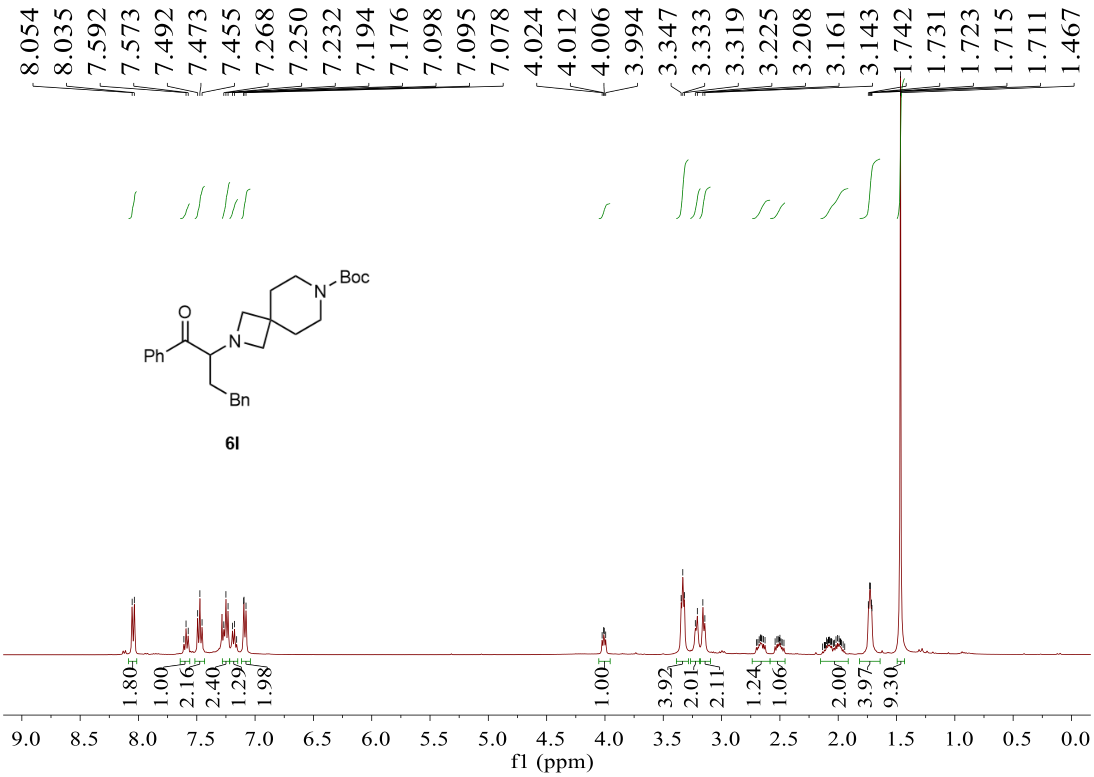
8.054 8.035 7.592 7.573 7.492 7.473 7.455 7.268 7.250 7.232 7.194 7.176 7.098 7.095 7.078 4.024 4.012 4.006 3.994 3.347 3.333 3.319 3.225 3.208 3.161 3.143 1.742 1.731 1.723 1.715 1.711 1.467
Boc
N
O
N
Ph
Bn
6l
1.80 1.00 2.16 2.40 1.29 1.98 1.00 3.92 2.01 2.11 1.24 1.06 2.00 3.97 9.30
9.0 8.5 8.0 7.5 7.0 6.5 6.0 5.5 5.0 4.5 4.0 3.5 3.0 2.5 2.0 1.5 1.0 0.5 0.0
f1 (ppm)

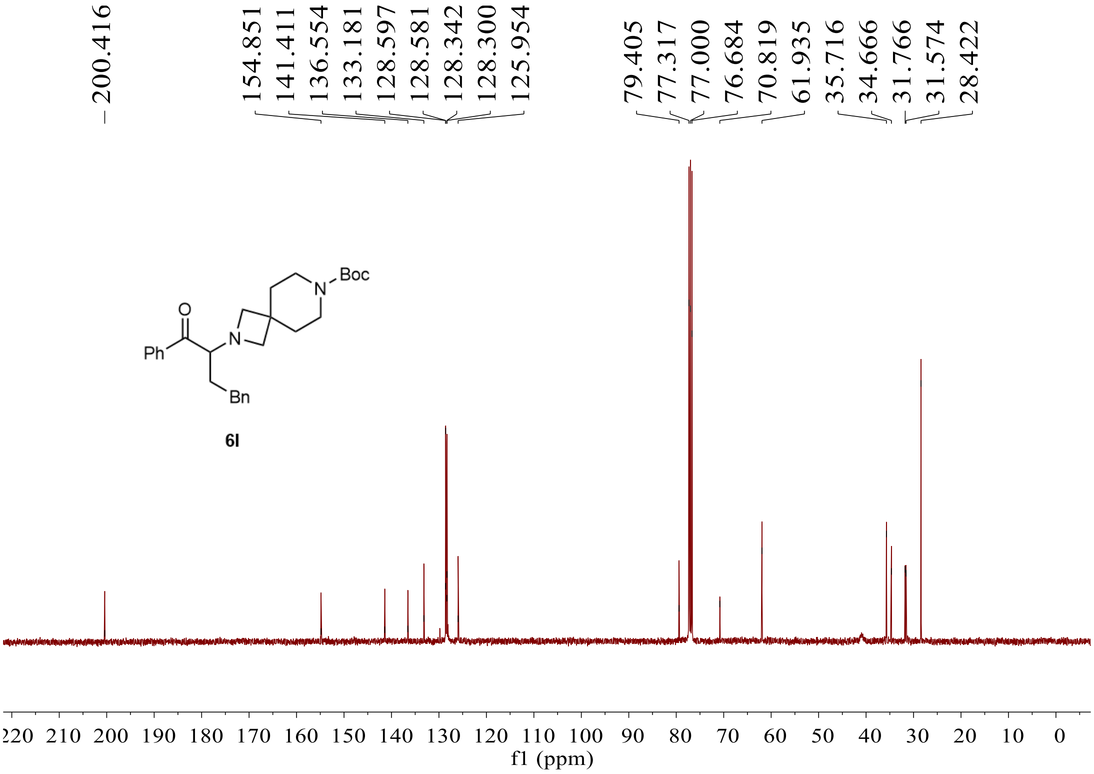
200.416
154.851 141.411 136.554 133.181 128.597 128.581 128.342 128.300 125.954
79.405 77.317 77.000 76.684 70.819 61.935 35.716 34.666 31.766 31.574 28.422
Boc
N
O
N
Ph
Bn
6l
220 210 200 190 180 170 160 150 140 130 120 110 100 90 80 70 60 50 40 30 20 10 0
f1 (ppm)

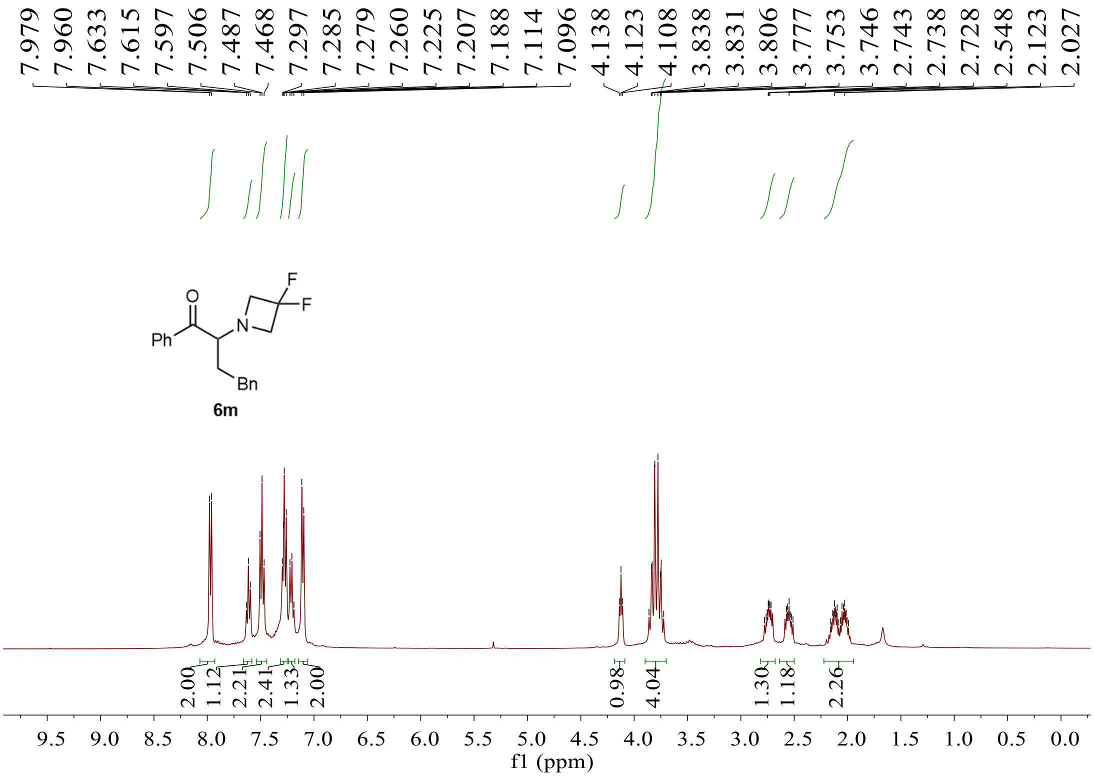
7.979
7.960
7.633
7.615
7.597
7.506
7.487
7.468
7.297
7.285
7.279
7.260
7.225
7.207
7.188
7.114
7.096
4.138
4.123
4.108
3.838
3.831
3.806
3.777
3.753
3.746
2.743
2.738
2.728
2.548
2.123
2.027
O
F
F
N
Ph
Bn
6m
2.00
1.12
2.21
2.41
1.33
2.00
0.98
4.04
1.30
1.18
2.26
9.5 9.0 8.5 8.0 7.5 7.0 6.5 6.0 5.5 5.0 4.5 4.0 3.5 3.0 2.5 2.0 1.5 1.0 0.5 0.0
f1 (ppm)

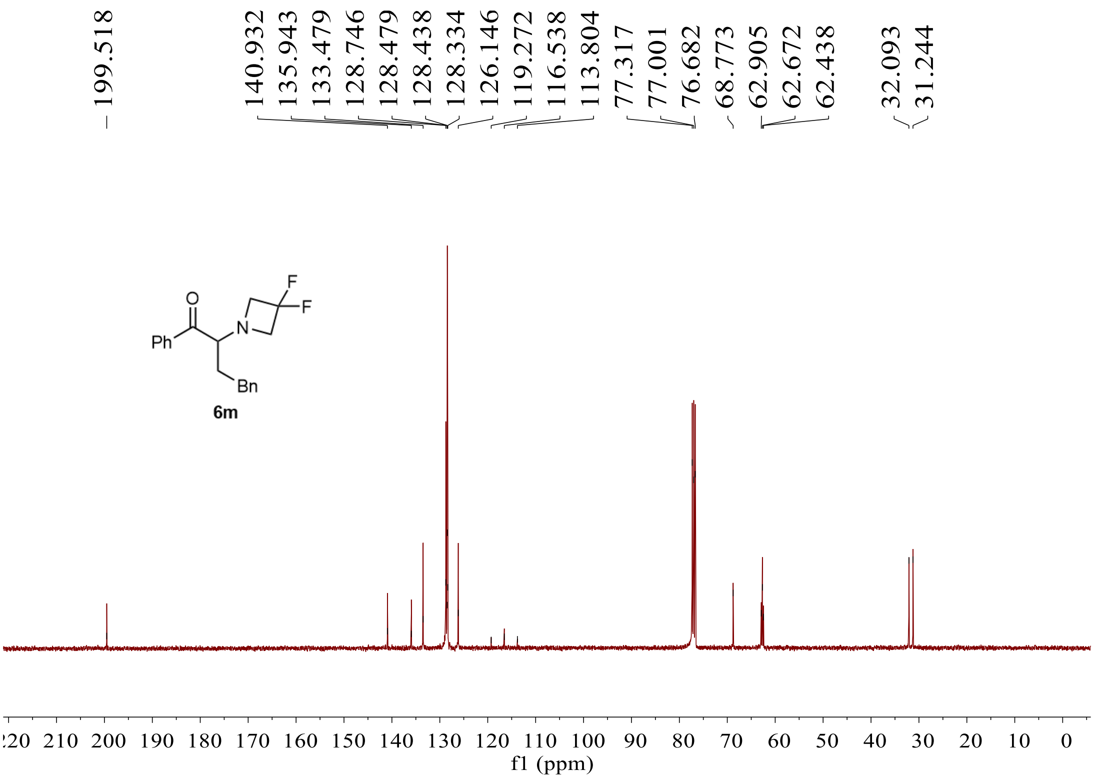
199.518
140.932
135.943
133.479
128.746
128.479
128.438
128.334
126.146
119.272
116.538
113.804
77.317
77.001
76.682
68.773
62.905
62.672
62.438
32.093
31.244
O
F
F
N
Ph
Bn
6m
220 210 200 190 180 170 160 150 140 130 120 110 100 90 80 70 60 50 40 30 20 10 0
f1 (ppm)

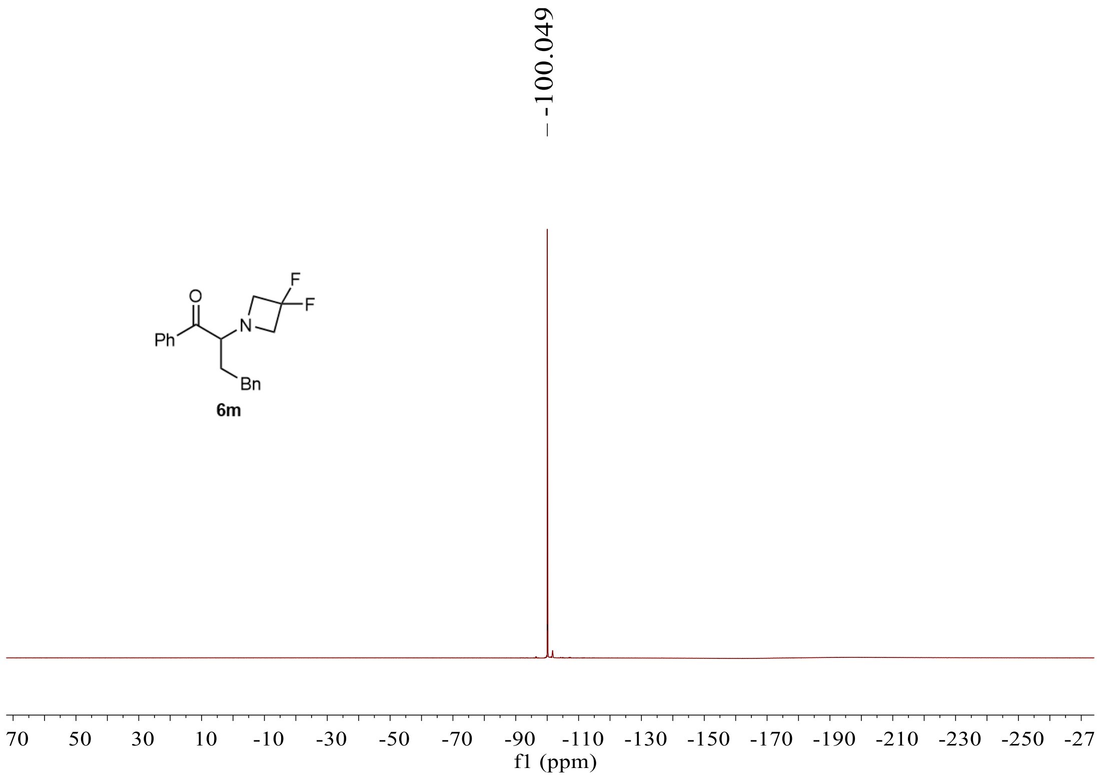
-100.049
6m
70 50 30 10 -10 -30 -50 -70 -90 -110 -130 -150 -170 -190 -210 -230 -250 -27
f1 (ppm)

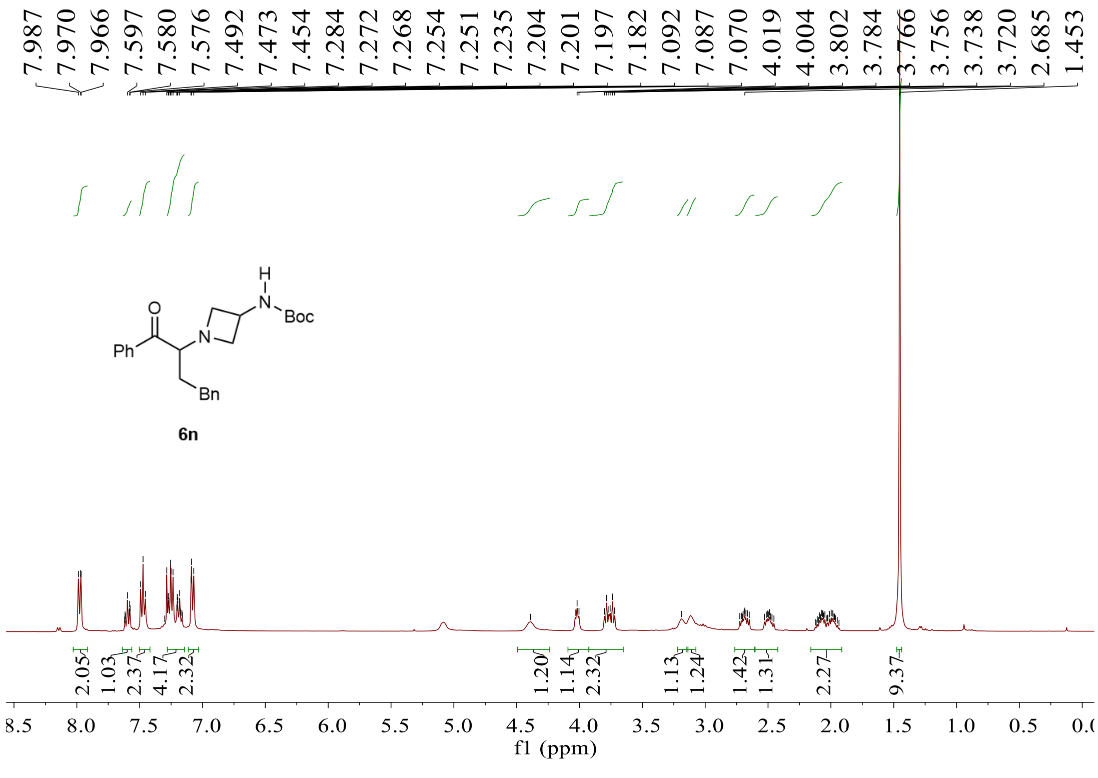
7.987 7.970 7.966 7.597 7.580 7.576 7.492 7.473 7.454 7.284 7.272 7.268 7.254 7.251 7.235 7.204 7.201 7.197 7.182 7.092 7.087 7.070 4.019 4.004 3.802 3.784 3.766 3.756 3.738 3.720 2.685 1.453
6n
2.05 1.03 2.37 4.17 2.32 1.20 1.14 2.32 1.13 1.24 1.42 1.31 2.27 9.37
8.5 8.0 7.5 7.0 6.5 6.0 5.5 5.0 4.5 4.0 3.5 3.0 2.5 2.0 1.5 1.0 0.5 0.0
f1 (ppm)

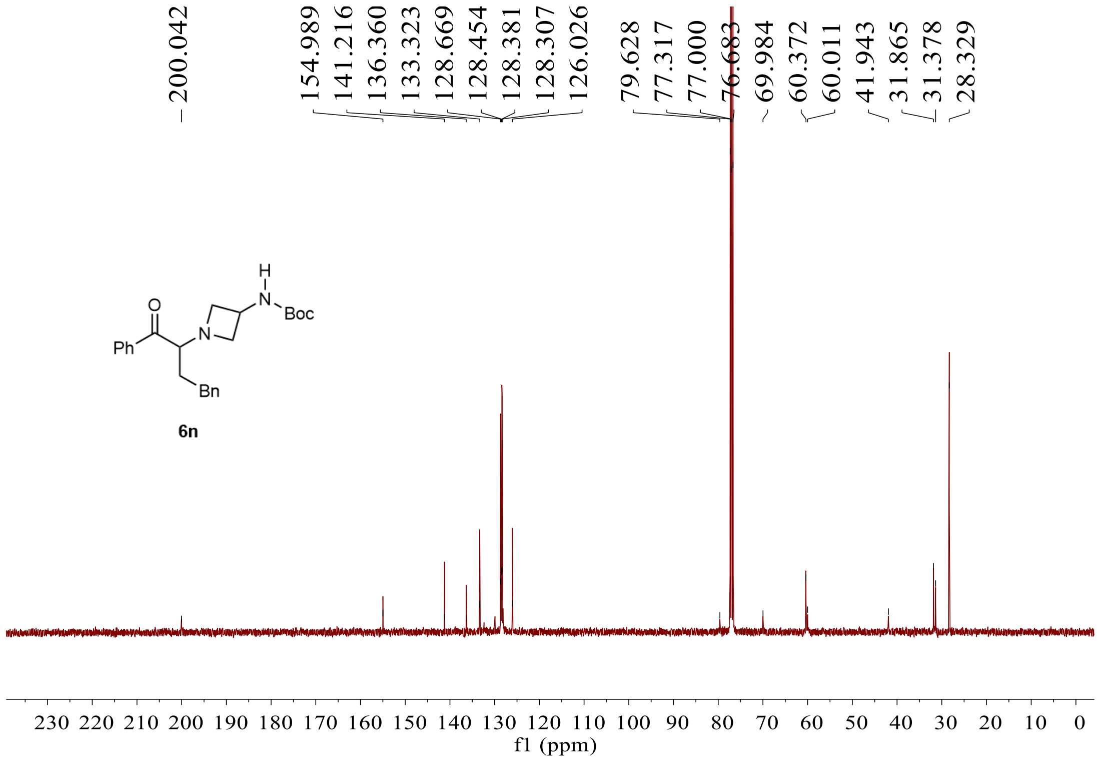
200.042
154.989
141.216
136.360
133.323
128.669
128.454
128.381
128.307
126.026
79.628
77.317
77.000
76.683
69.984
60.372
60.011
41.943
31.865
31.378
28.329
Ph
O
N
H
N
Boc
Bn
6n
230 220 210 200 190 180 170 160 150 140 130 120 110 100 90 80 70 60 50 40 30 20 10 0
f1 (ppm)

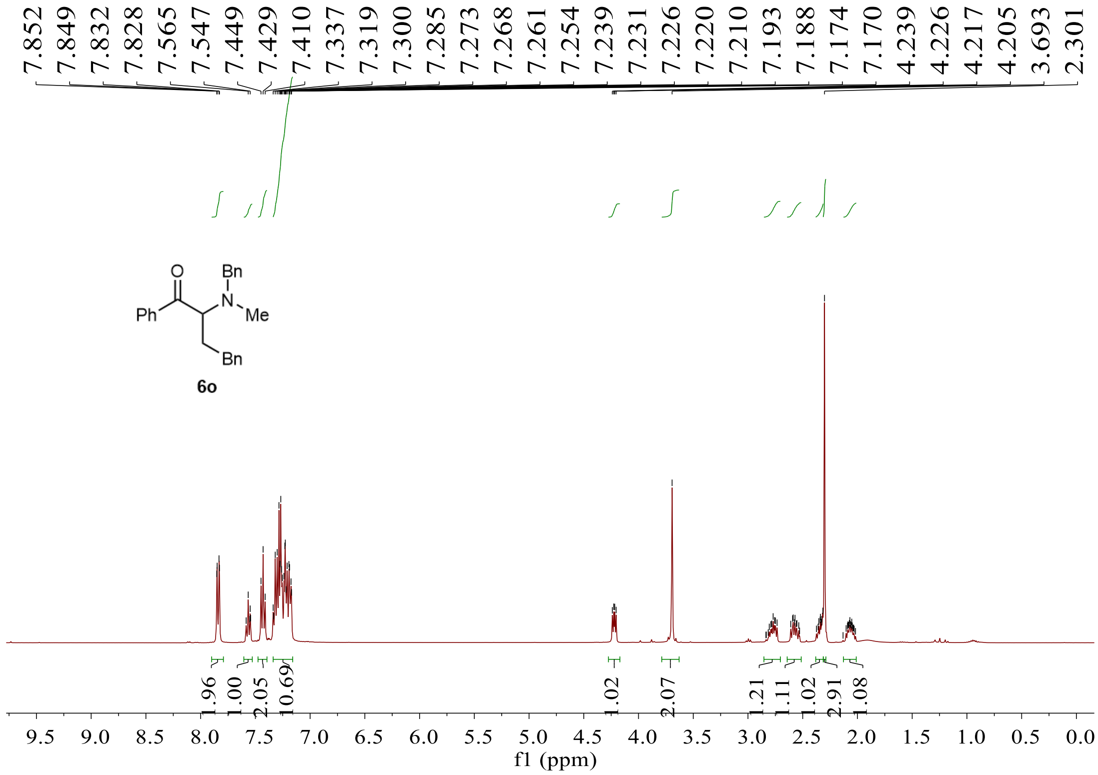
7.852
7.849
7.832
7.828
7.565
7.547
7.449
7.429
7.410
7.337
7.319
7.300
7.285
7.273
7.268
7.261
7.254
7.239
7.231
7.226
7.220
7.210
7.193
7.188
7.174
7.170
4.239
4.226
4.217
4.205
3.693
2.301
Ph
O
Bn
N
Me
Bn
6o
1.96
1.00
2.05
10.69
1.02
2.07
1.21
1.11
1.02
2.91
1.08
9.5 9.0 8.5 8.0 7.5 7.0 6.5 6.0 5.5 5.0 4.5 4.0 3.5 3.0 2.5 2.0 1.5 1.0 0.5 0.0
f1 (ppm)

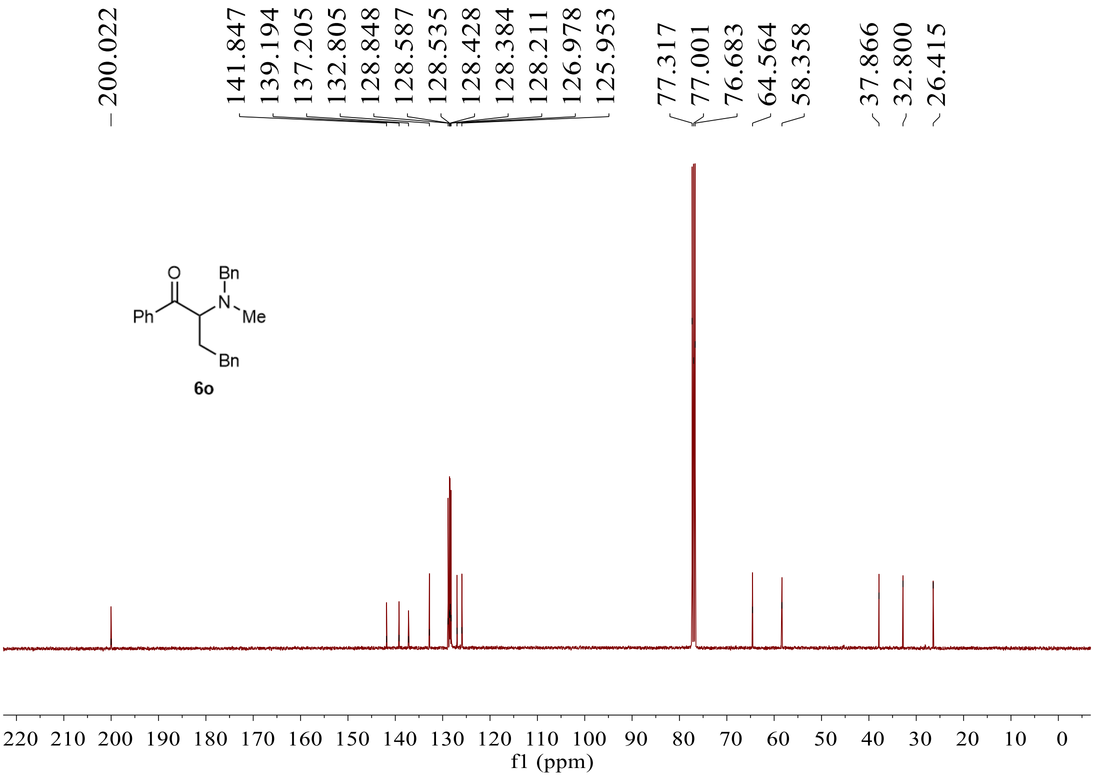
200.022
141.847
139.194
137.205
132.805
128.848
128.587
128.535
128.428
128.384
128.211
126.978
125.953
77.317
77.001
76.683
64.564
58.358
37.866
32.800
26.415
O
Bn
N
Ph
Me
Bn
6o
220 210 200 190 180 170 160 150 140 130 120 110 100 90 80 70 60 50 40 30 20 10 0
f1 (ppm)

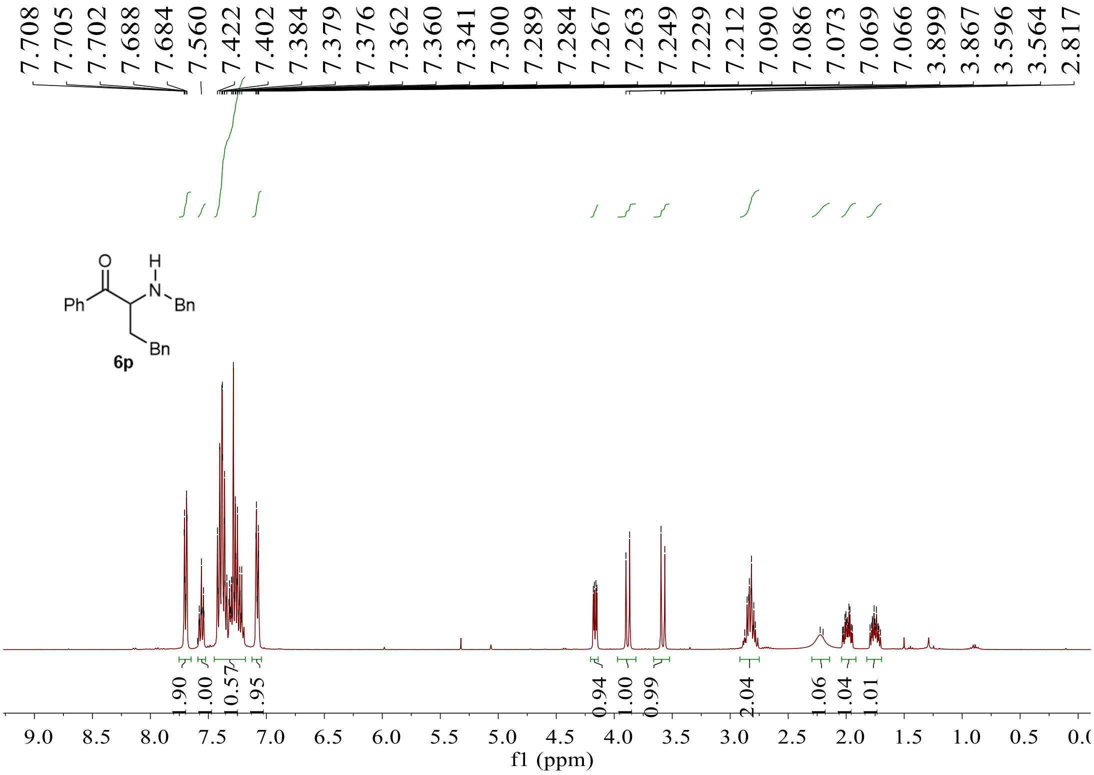
7.708
7.705
7.702
7.688
7.684
7.560
7.422
7.402
7.384
7.379
7.376
7.362
7.360
7.341
7.300
7.289
7.284
7.267
7.263
7.249
7.229
7.212
7.090
7.086
7.073
7.069
7.066
3.899
3.867
3.596
3.564
2.817
O
H
N
Ph
Bn
Bn
6p
1.90
1.00
10.57
1.95
0.94
1.00
0.99
2.04
1.06
1.04
1.01
9.0 8.5 8.0 7.5 7.0 6.5 6.0 5.5 5.0 4.5 4.0 3.5 3.0 2.5 2.0 1.5 1.0 0.5 0.0
f1 (ppm)

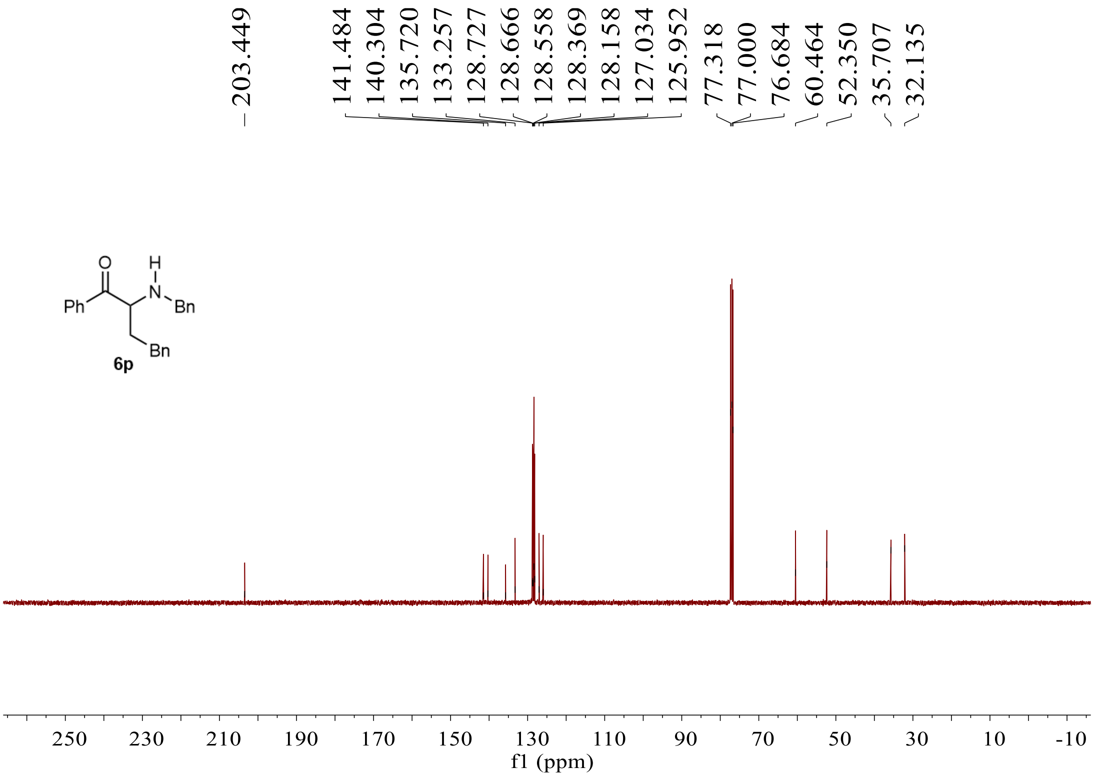
203.449
141.484
140.304
135.720
133.257
128.727
128.666
128.558
128.369
128.158
127.034
125.952
77.318
77.000
76.684
60.464
52.350
35.707
32.135
Ph
O
H
N
Bn
Bn
6p
250
230
210
190
170
150
130
110
90
70
50
30
10
-10
f1 (ppm)

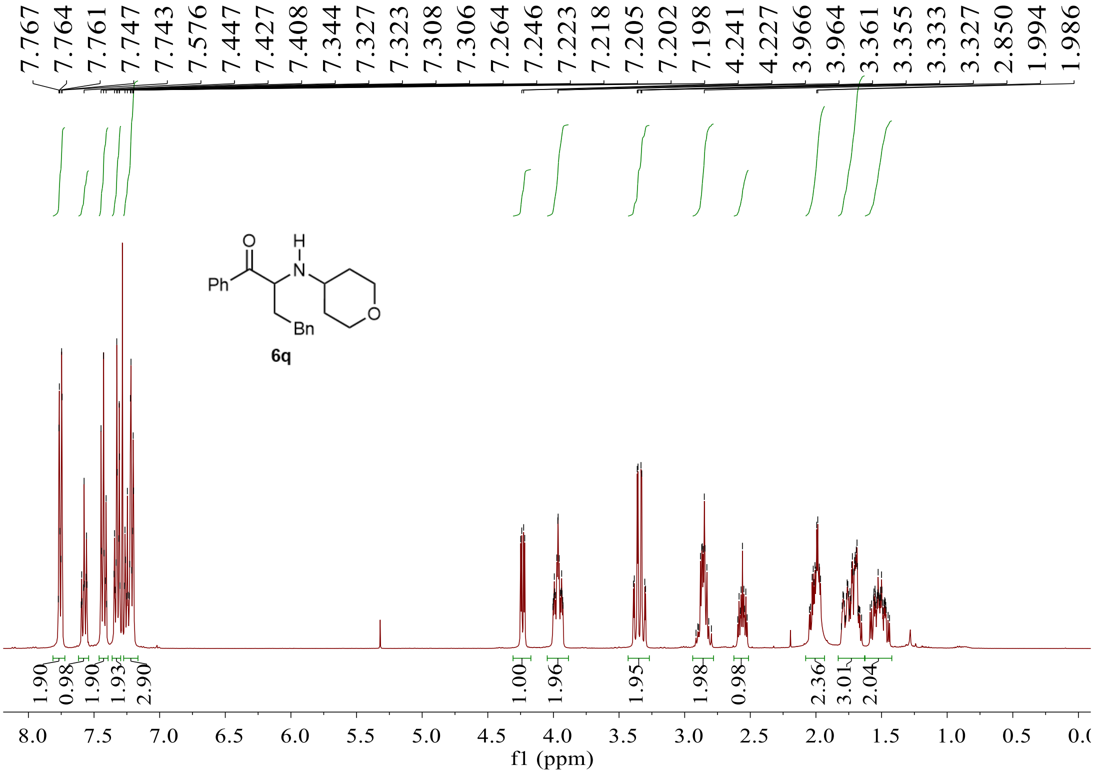
7.767
7.764
7.761
7.747
7.743
7.576
7.447
7.427
7.408
7.344
7.327
7.323
7.308
7.306
7.264
7.246
7.223
7.218
7.205
7.202
7.198
4.241
4.227
3.966
3.964
3.361
3.355
3.333
3.327
2.850
1.994
1.986
Ph
O
H
N
O
Bn
6q
1.90
0.98
1.90
1.93
2.90
1.00
1.96
1.95
1.98
0.98
2.36
3.01
2.04
8.0
7.5
7.0
6.5
6.0
5.5
5.0
4.5
4.0
3.5
3.0
2.5
2.0
1.5
1.0
0.5
f1 (ppm)

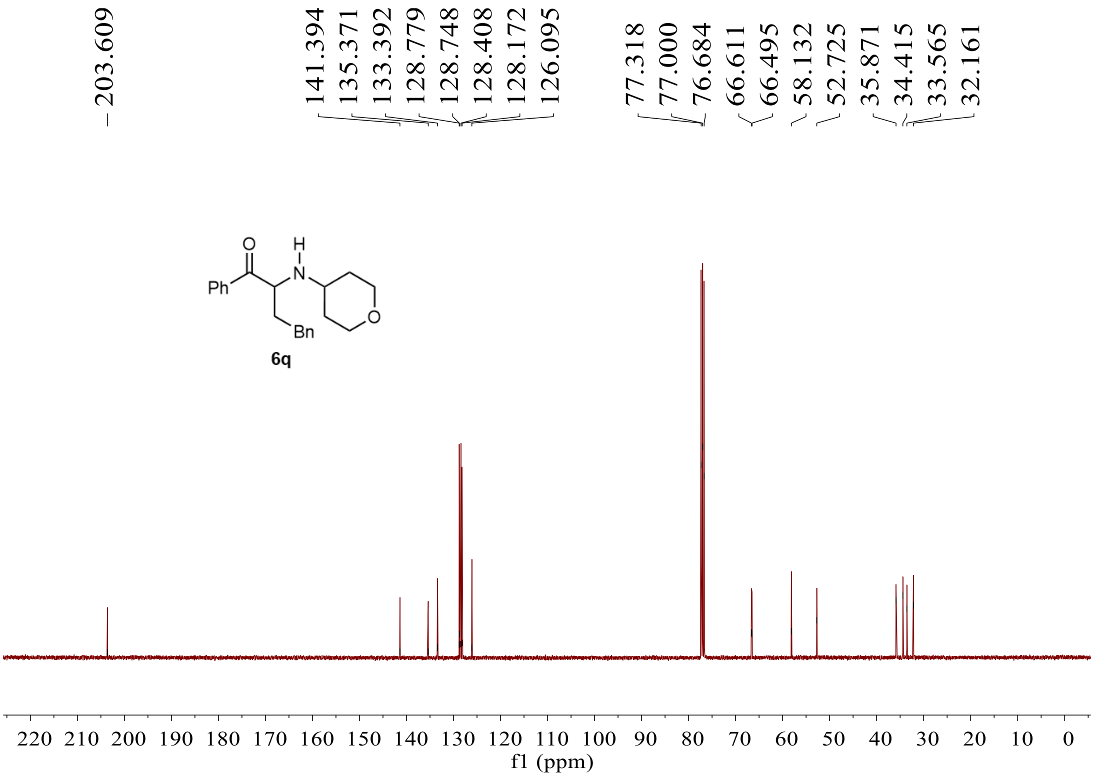

203.609
141.394
135.371
133.392
128.779
128.748
128.408
128.172
126.095
77.318
77.000
76.684
66.611
66.495
58.132
52.725
35.871
34.415
33.565
32.161
Ph
O
H
N
O
Bn
6q
220 210 200 190 180 170 160 150 140 130 120 110 100 90 80 70 60 50 40 30 20 10 0
f1 (ppm)

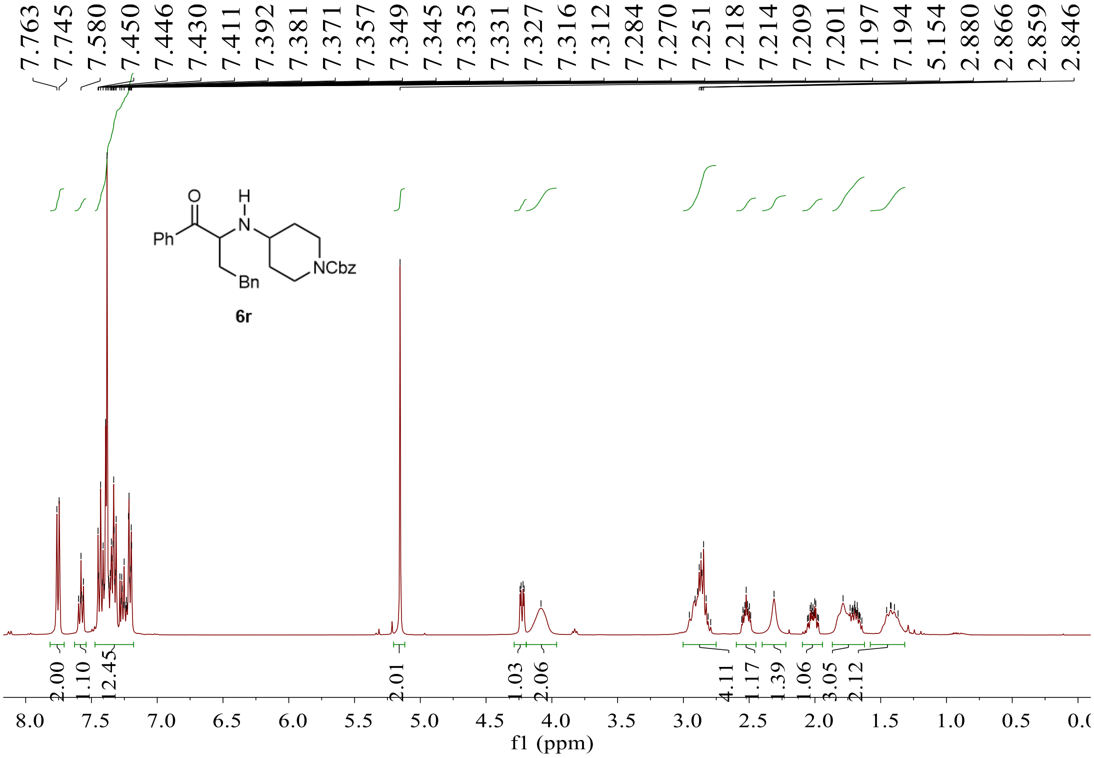

7.763
7.745
7.580
7.450
7.446
7.430
7.411
7.392
7.381
7.371
7.357
7.349
7.345
7.335
7.331
7.327
7.316
7.312
7.284
7.270
7.251
7.218
7.214
7.209
7.201
7.197
7.194
5.154
2.880
2.866
2.859
2.846
Ph
O
H
N
NCbz
Bn
6r
2.00
1.10
12.45
2.01
1.03
2.06
4.11
1.17
1.39
1.06
3.05
2.12
8.0 7.5 7.0 6.5 6.0 5.5 5.0 4.5 4.0 3.5 3.0 2.5 2.0 1.5 1.0 0.5 0.0
f1 (ppm)

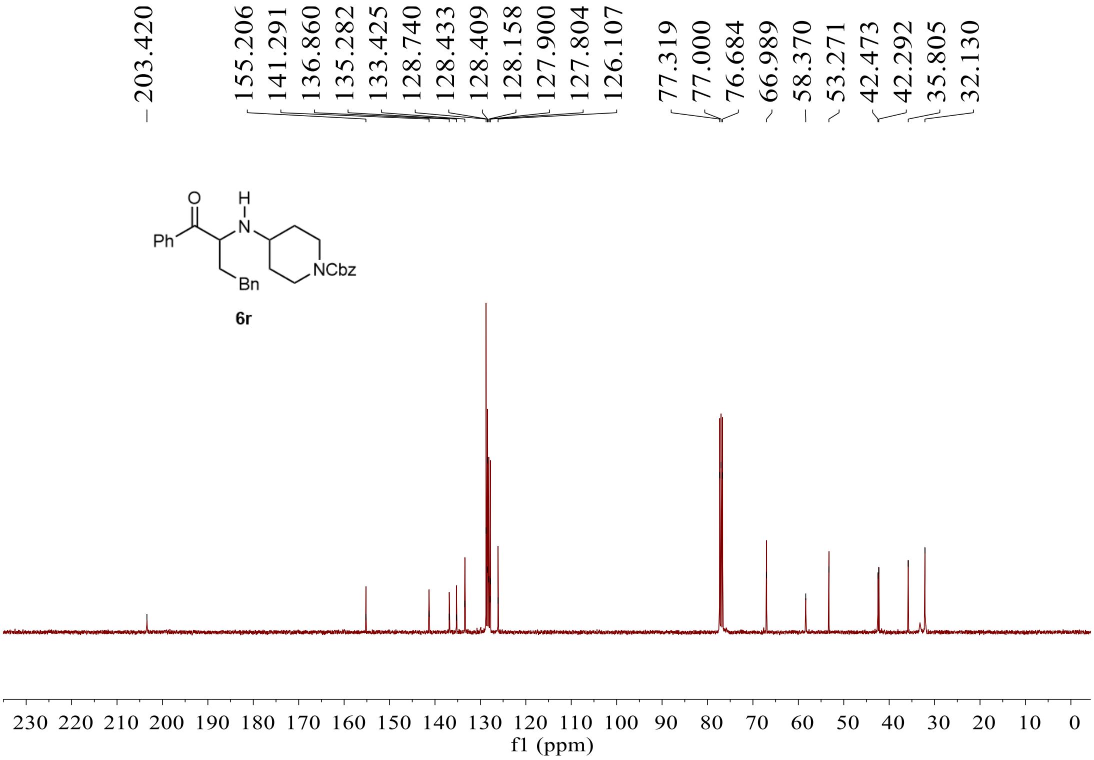
203.420
155.206
141.291
136.860
135.282
133.425
128.740
128.433
128.409
128.158
127.900
127.804
126.107
77.319
77.000
76.684
66.989
58.370
53.271
42.473
42.292
35.805
32.130
Ph
O
H
N
NCbz
Bn
6r
230 220 210 200 190 180 170 160 150 140 130 120 110 100 90 80 70 60 50 40 30 20 10 0
f1 (ppm)

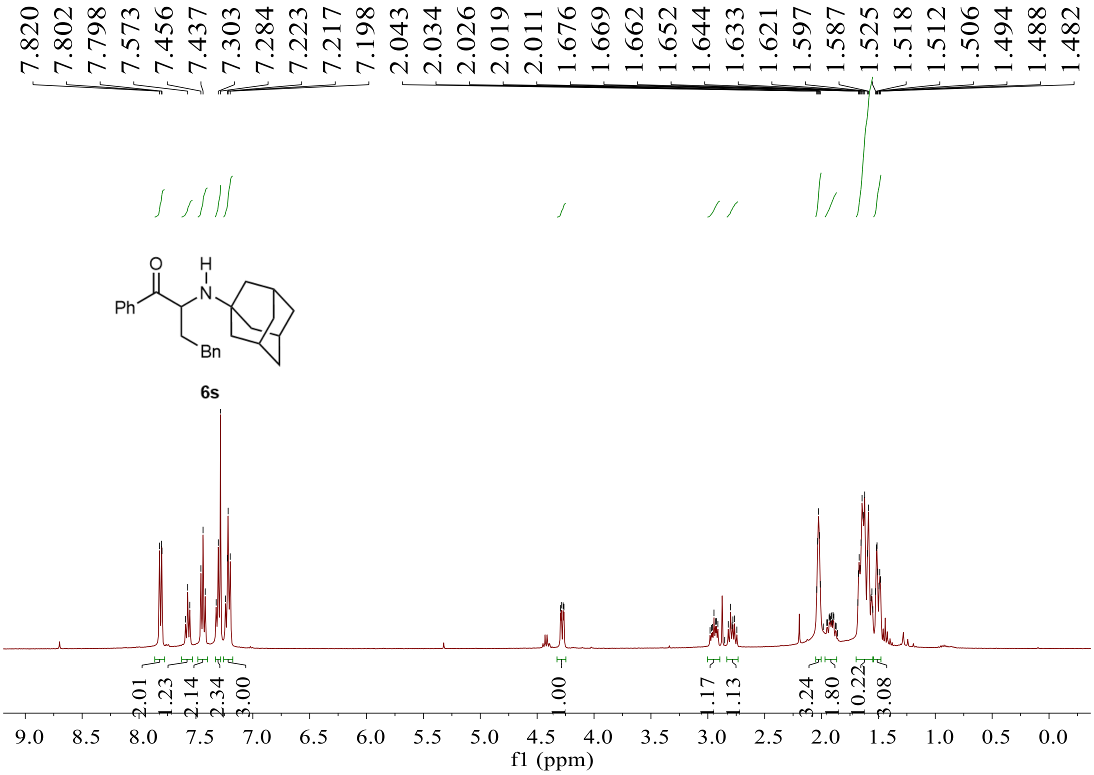
7.820
7.802
7.798
7.573
7.456
7.437
7.303
7.284
7.223
7.217
7.198
2.043
2.034
2.026
2.019
2.011
1.676
1.669
1.662
1.652
1.644
1.633
1.621
1.597
1.587
1.525
1.518
1.512
1.506
1.494
1.488
1.482
Ph
O
H
N
Bn
6s
2.01
1.23
2.14
2.34
3.00
1.00
1.17
1.13
3.24
1.80
10.22
3.08
9.0 8.5 8.0 7.5 7.0 6.5 6.0 5.5 5.0 4.5 4.0 3.5 3.0 2.5 2.0 1.5 1.0 0.5 0.0
f1 (ppm)

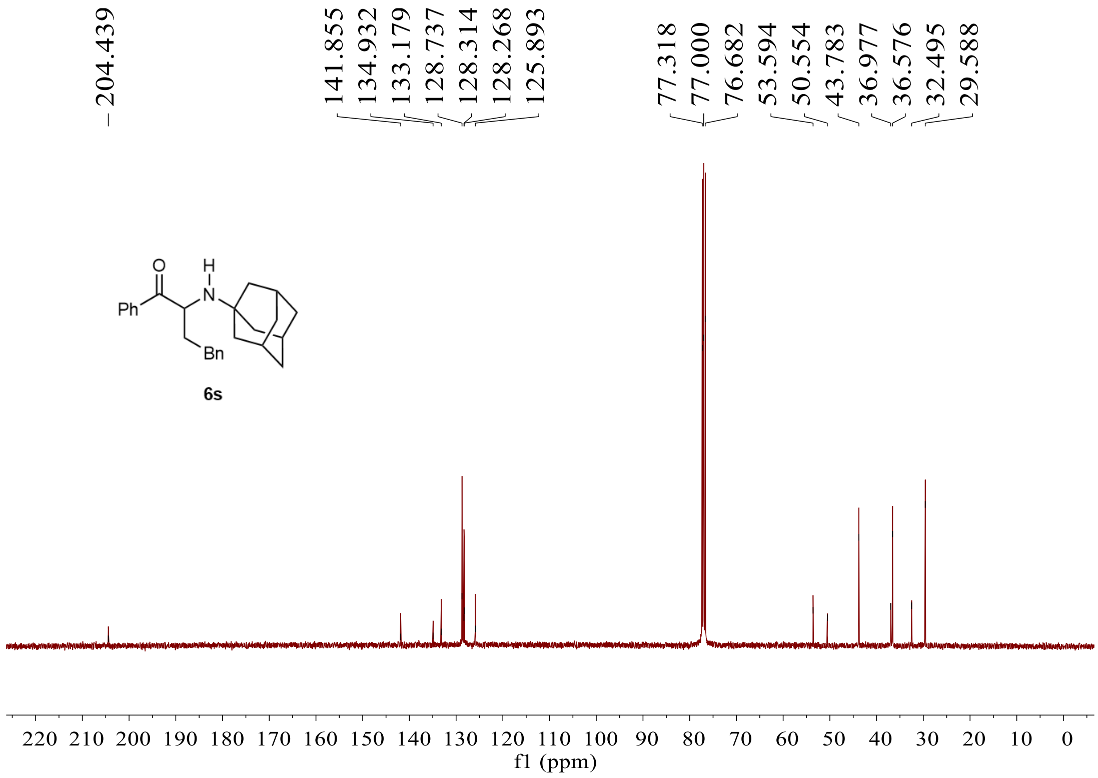

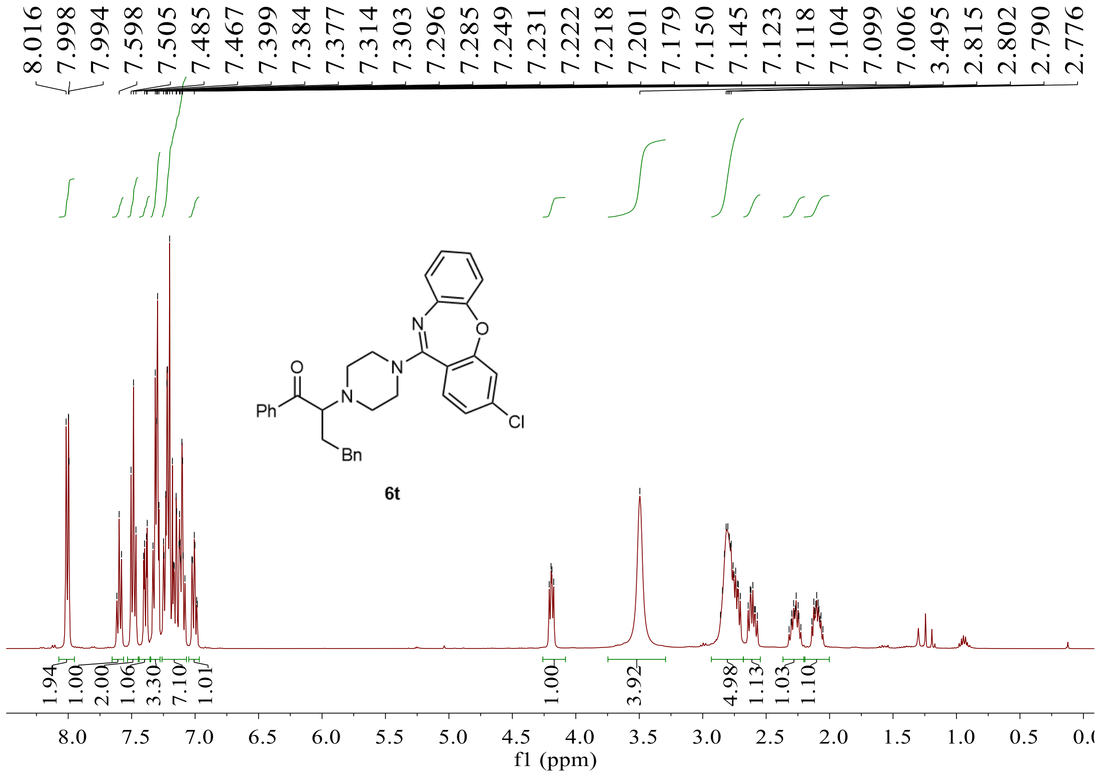

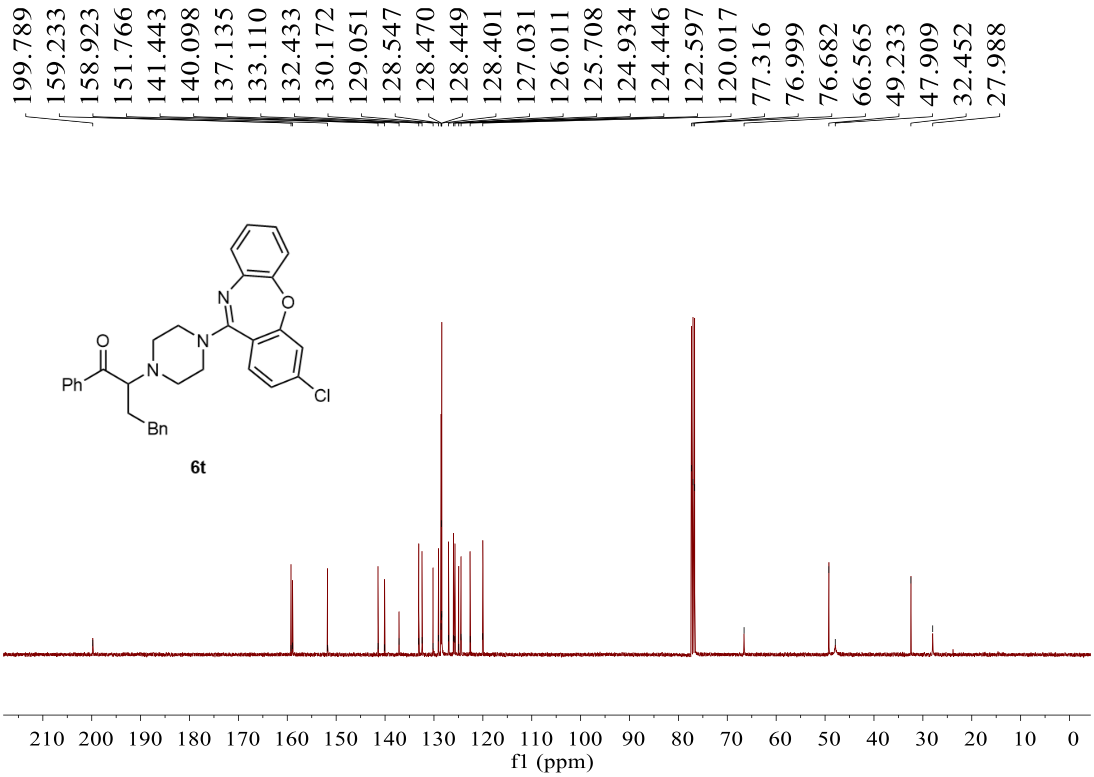
199.789
159.233
158.923
151.766
141.443
140.098
137.135
133.110
132.433
130.172
129.051
128.547
128.470
128.449
128.401
127.031
126.011
125.708
124.934
124.446
122.597
120.017
77.316
76.999
76.682
66.565
49.233
47.909
32.452
27.988
O
Ph
N
N
N
O
Cl
Bn
6t
210 200 190 180 170 160 150 140 130 120 110 100 90 80 70 60 50 40 30 20 10 0
f1 (ppm)

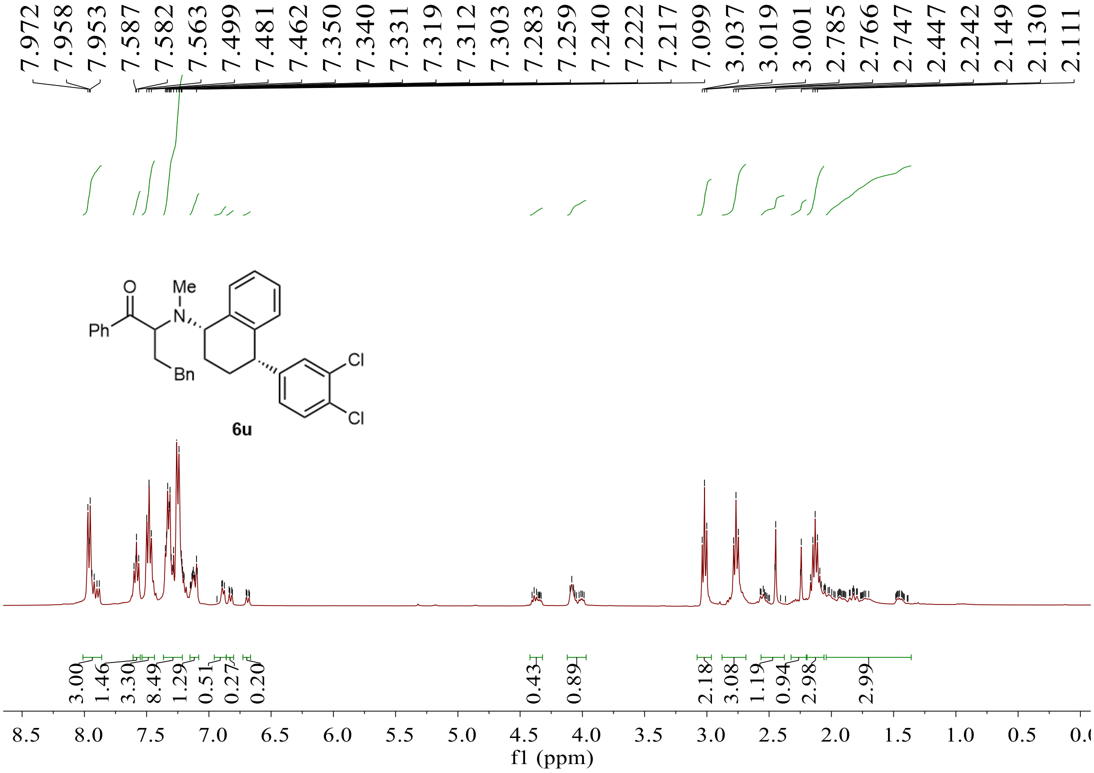
7.972
7.958
7.953
7.587
7.582
7.563
7.499
7.481
7.462
7.350
7.340
7.331
7.319
7.312
7.303
7.283
7.259
7.240
7.222
7.217
7.099
3.037
3.019
3.001
2.785
2.766
2.747
2.447
2.242
2.149
2.130
2.111
O
Me
Ph
N
Bn
Cl
Cl
6u
3.00
1.46
3.30
8.49
1.29
0.51
0.27
0.20
0.43
0.89
2.18
3.08
1.19
0.94
2.98
2.99
8.5 8.0 7.5 7.0 6.5 6.0 5.5 5.0 4.5 4.0 3.5 3.0 2.5 2.0 1.5 1.0 0.5
f1 (ppm)

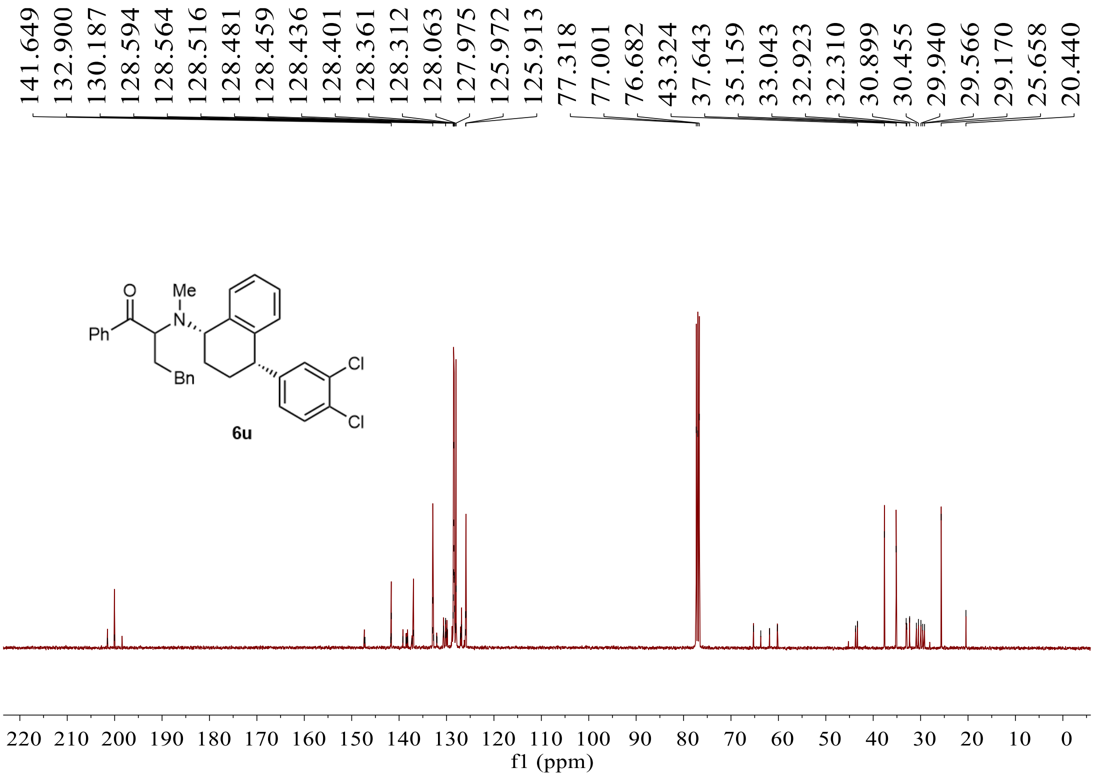
141.649
132.900
130.187
128.594
128.564
128.516
128.481
128.459
128.436
128.401
128.361
128.312
128.063
127.975
125.972
125.913
77.318
77.001
76.682
43.324
37.643
35.159
33.043
32.923
32.310
30.899
30.455
29.940
29.566
29.170
25.658
20.440
O
Me
N
Ph
Bn
Cl
Cl
6u
220 210 200 190 180 170 160 150 140 130 120 110 100 90 80 70 60 50 40 30 20 10 0
f1 (ppm)

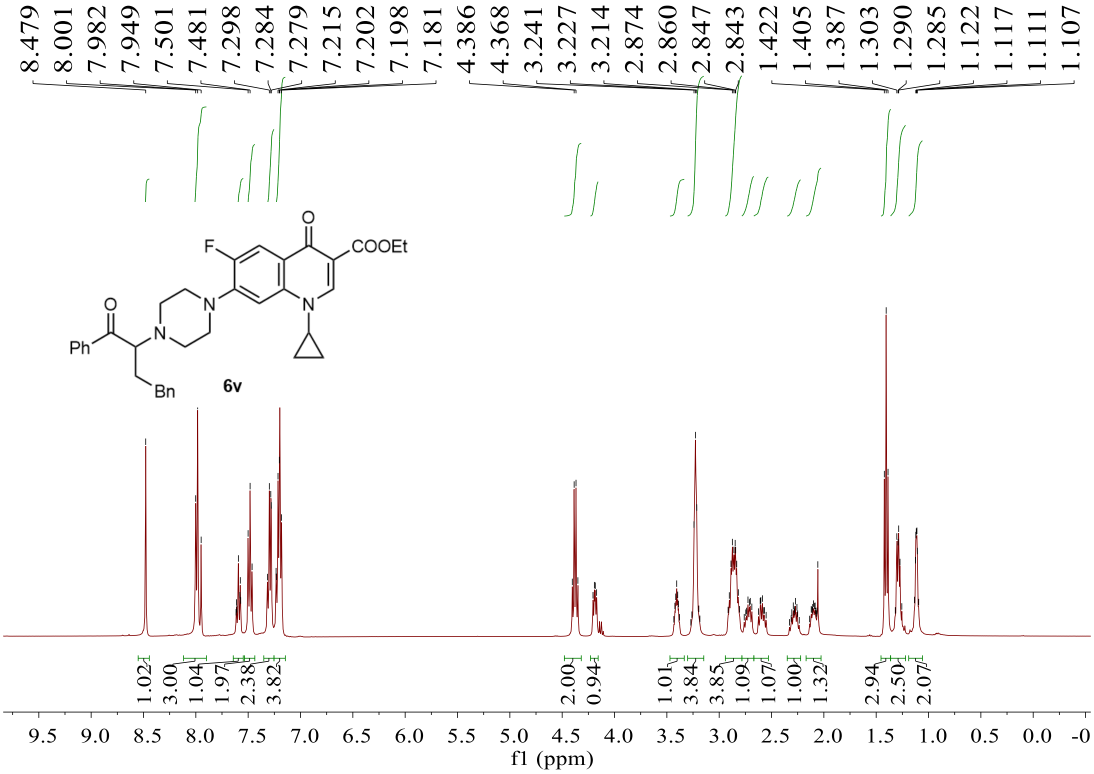
8.479
8.001
7.982
7.949
7.501
7.481
7.298
7.284
7.279
7.215
7.202
7.198
7.181
4.386
4.368
3.241
3.227
3.214
2.874
2.860
2.847
2.843
1.422
1.405
1.387
1.303
1.290
1.285
1.122
1.117
1.111
1.107
O
F
COOEt
N
N
N
Ph
Bn
6v
1.02
3.00
1.04
1.97
2.38
3.82
2.00
0.94
1.01
3.84
3.85
1.09
1.07
1.00
1.32
2.94
2.50
2.07
9.5 9.0 8.5 8.0 7.5 7.0 6.5 6.0 5.5 5.0 4.5 4.0 3.5 3.0 2.5 2.0 1.5 1.0 0.5 0.0 -0
f1 (ppm)

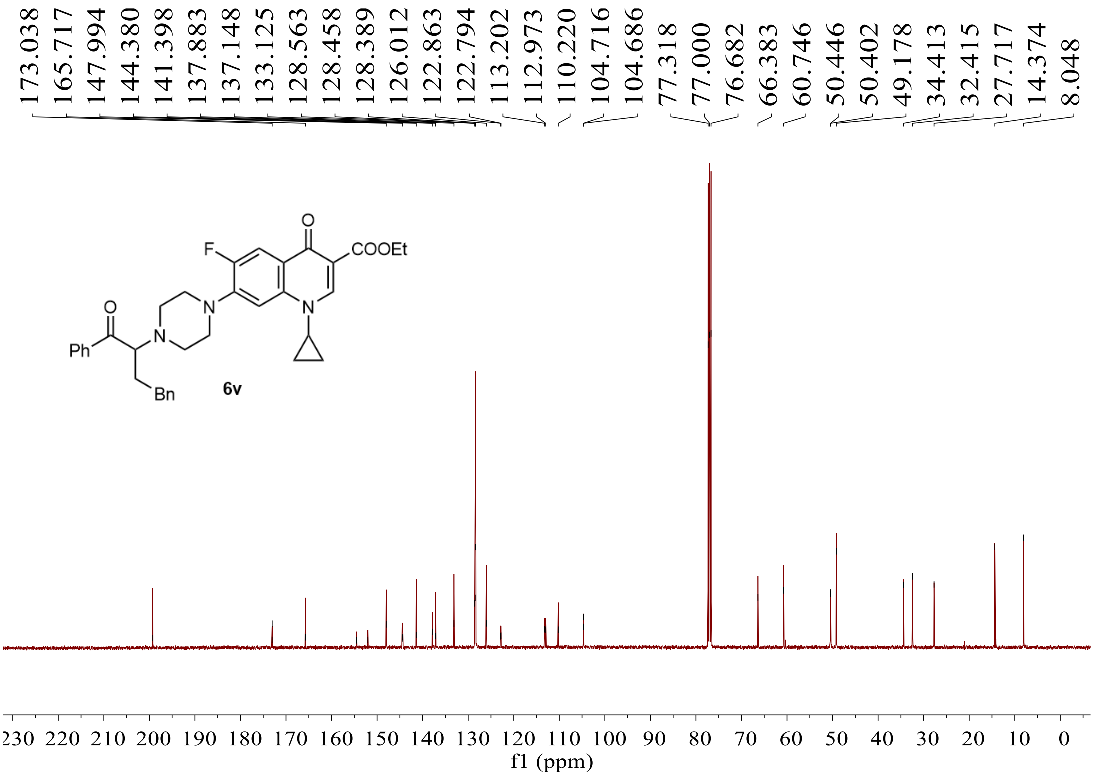
173.038
165.717
147.994
144.380
141.398
137.883
137.148
133.125
128.563
128.458
128.389
126.012
122.863
122.794
113.202
112.973
110.220
104.716
104.686
77.318
77.000
76.682
66.383
60.746
50.446
50.402
49.178
34.413
32.415
27.717
14.374
8.048
F
O
COOEt
N
N
N
O
Ph
Bn
6v
230 220 210 200 190 180 170 160 150 140 130 120 110 100 90 80 70 60 50 40 30 20 10 0
f1 (ppm)

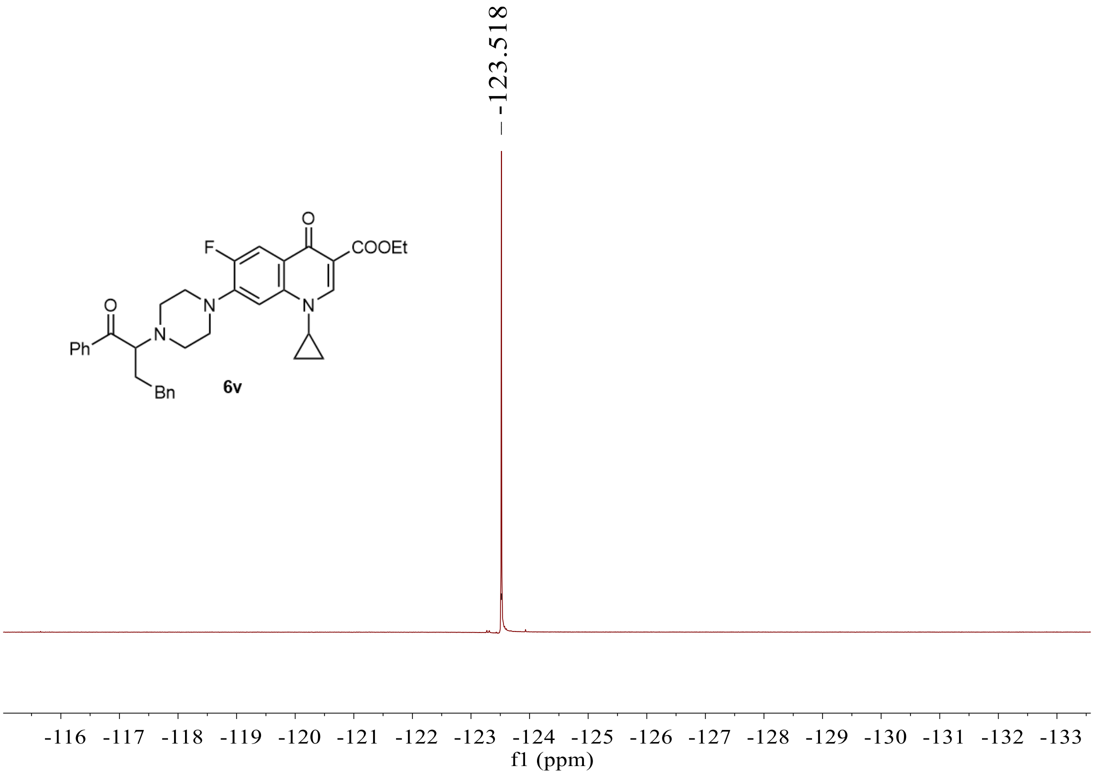
-123.518
F
O
COOEt
N
N
N
O
Ph
Bn
6v
-116 -117 -118 -119 -120 -121 -122 -123 -124 -125 -126 -127 -128 -129 -130 -131 -132 -133
f1 (ppm)

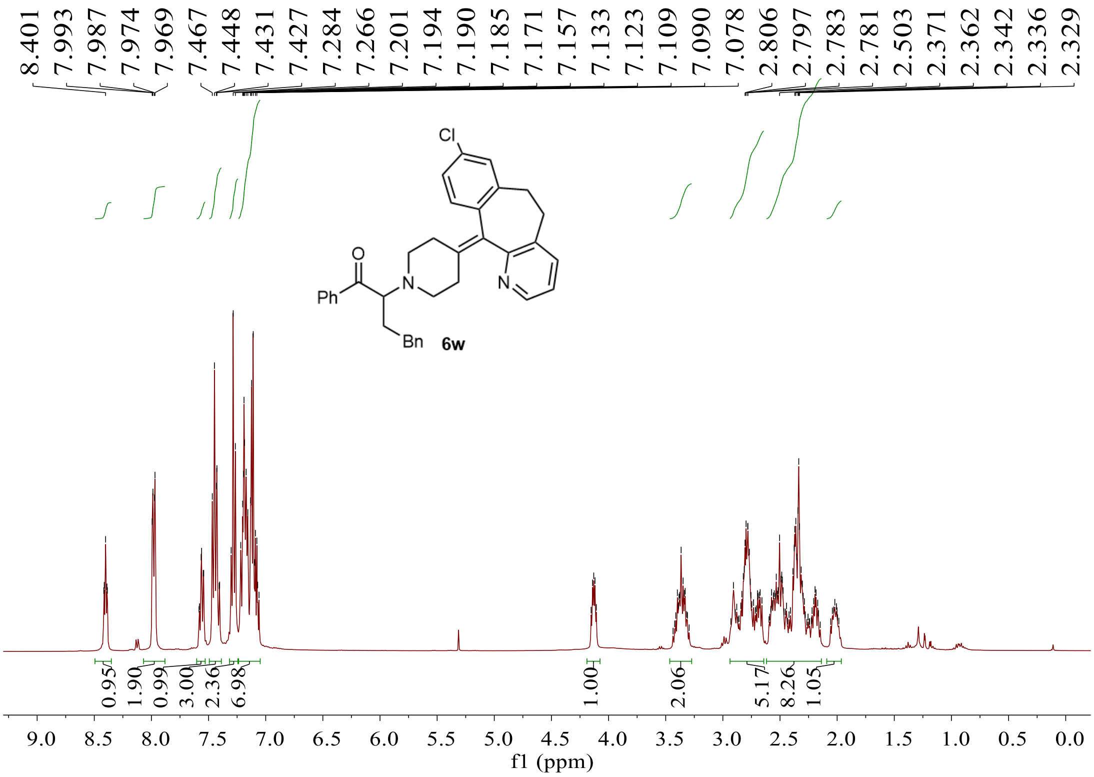
8.401 7.993 7.987 7.974 7.969 7.467 7.448 7.431 7.427 7.284 7.266 7.201 7.194 7.190 7.185 7.171 7.157 7.133 7.123 7.109 7.090 7.078 2.806 2.797 2.783 2.781 2.503 2.371 2.362 2.342 2.336 2.329
Cl
O
Ph
N
N
Bn
6w
0.95 1.90 0.99 3.00 2.36 6.98 1.00 2.06 5.17 8.26 1.05
9.0 8.5 8.0 7.5 7.0 6.5 6.0 5.5 5.0 4.5 4.0 3.5 3.0 2.5 2.0 1.5 1.0 0.5 0.0
f1 (ppm)

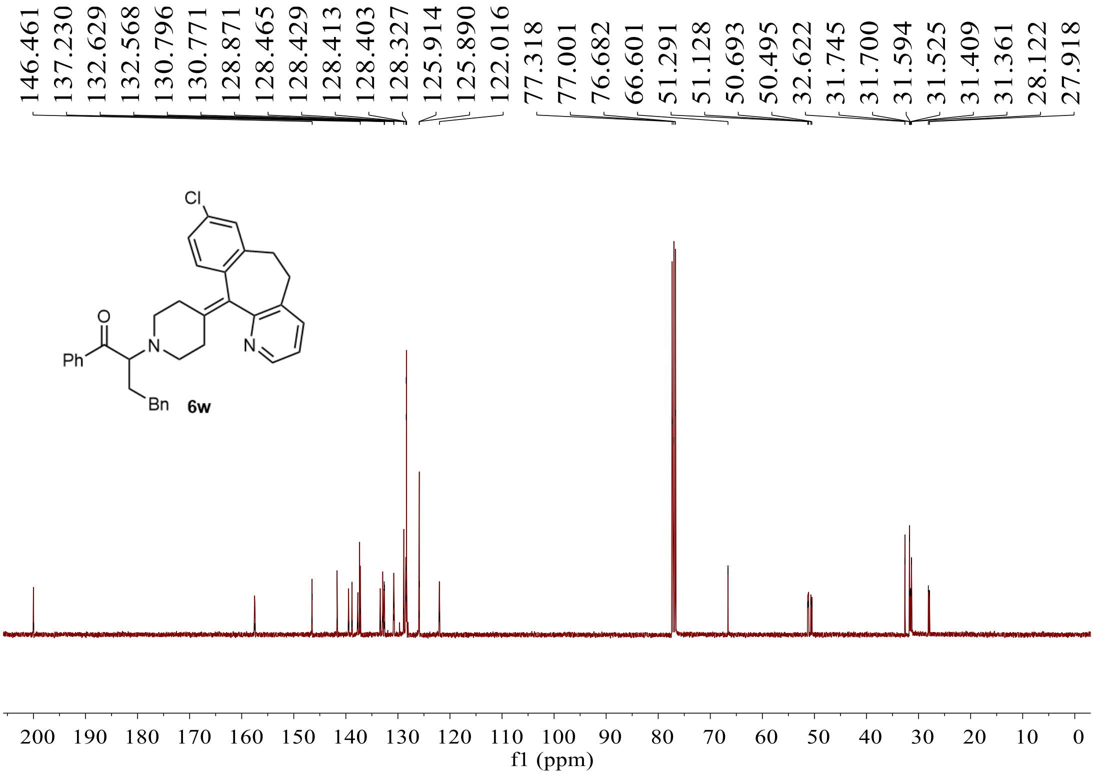
146.461 137.230 132.629 132.568 130.796 130.771 128.871 128.465 128.429 128.413 128.403 128.327 125.914 125.890 122.016 77.318 77.001 76.682 66.601 51.291 51.128 50.693 50.495 32.622 31.745 31.700 31.594 31.525 31.409 31.361 28.122 27.918
Cl
O
Ph
N
N
Bn
6w
200 190 180 170 160 150 140 130 120 110 100 90 80 70 60 50 40 30 20 10 0
f1 (ppm)

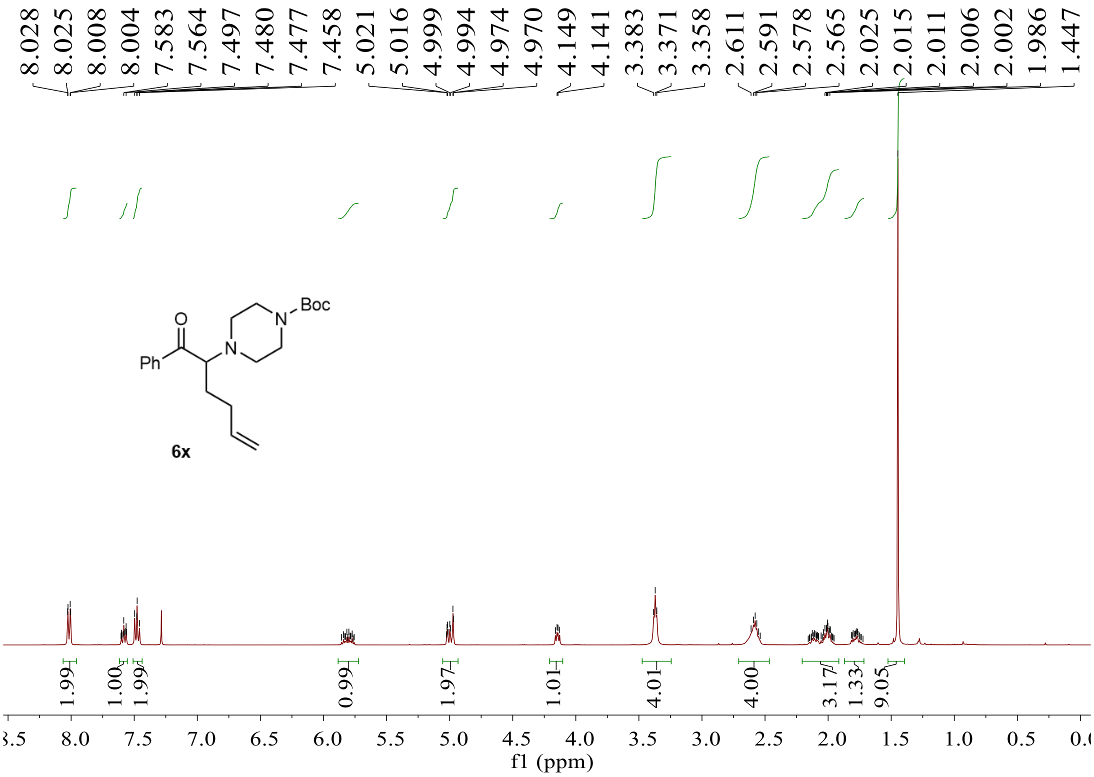
8.028 8.025 8.008 8.004 7.583 7.564 7.497 7.480 7.477 7.458 5.021 5.016 4.999 4.994 4.974 4.970 4.149 4.141 3.383 3.371 3.358 2.611 2.591 2.578 2.565 2.025 2.015 2.011 2.006 2.002 1.986 1.447
Boc
O
N
Ph
N
6x
1.99 1.00 1.99 0.99 1.97 1.01 4.01 4.00 3.17 1.33 9.05
f1 (ppm)

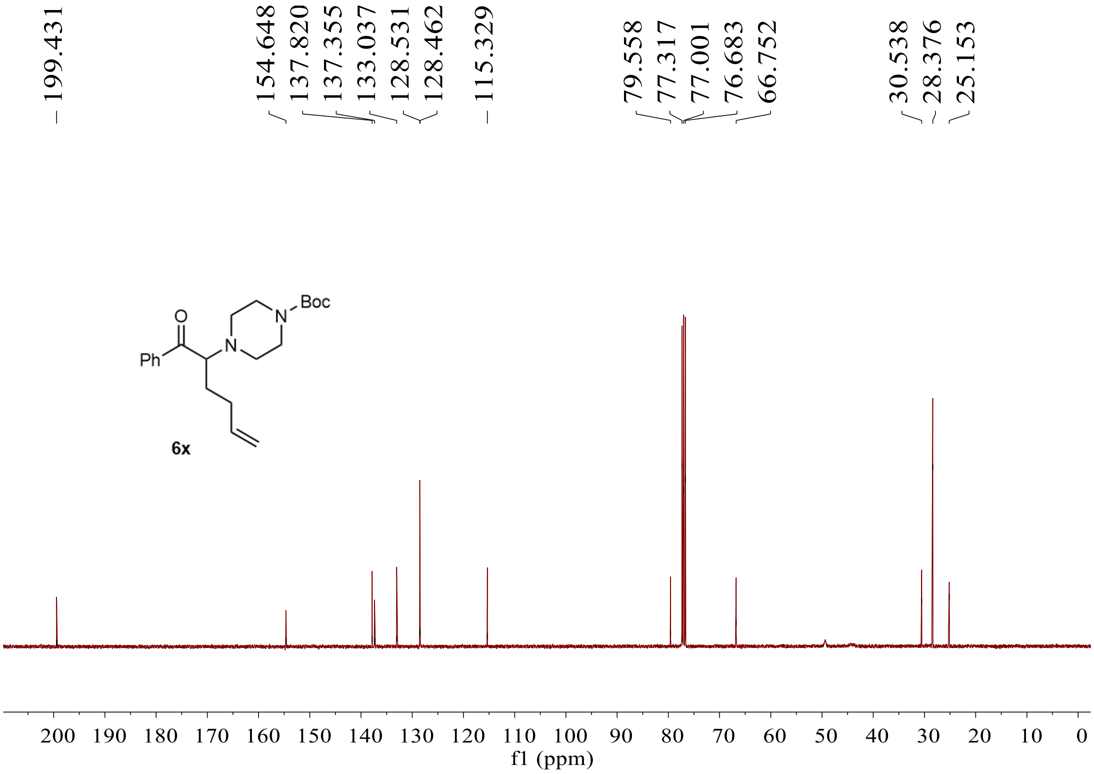
199.431
154.648 137.820 137.355 133.037 128.531 128.462 115.329
79.558 77.317 77.001 76.683 66.752
30.538 28.376 25.153
Boc
O
N
Ph
N
6x
f1 (ppm)

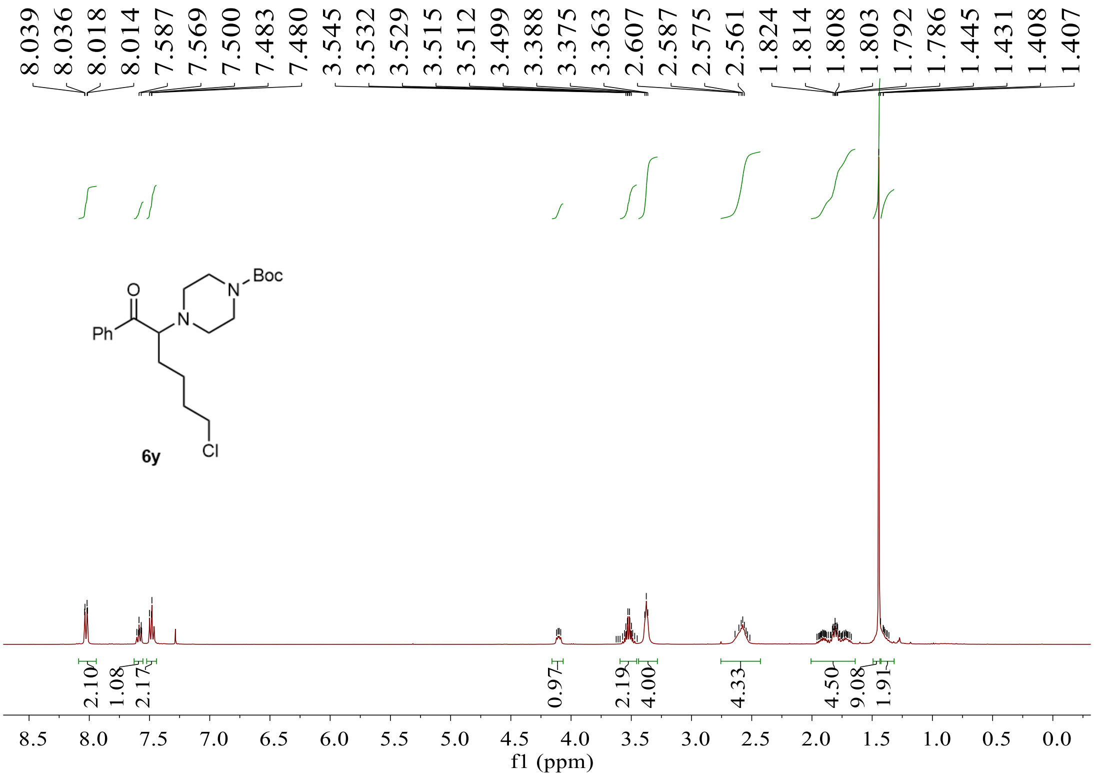
8.039
8.036
8.018
8.014
7.587
7.569
7.500
7.483
7.480
3.545
3.532
3.529
3.515
3.512
3.499
3.388
3.375
3.363
2.607
2.587
2.575
2.561
1.824
1.814
1.808
1.803
1.792
1.786
1.445
1.431
1.408
1.407
O
N
N
Boc
Ph
Cl
6y
2.10
1.08
2.17
0.97
2.19
4.00
4.33
4.50
9.08
1.91
8.5 8.0 7.5 7.0 6.5 6.0 5.5 5.0 4.5 4.0 3.5 3.0 2.5 2.0 1.5 1.0 0.5 0.0
f1 (ppm)

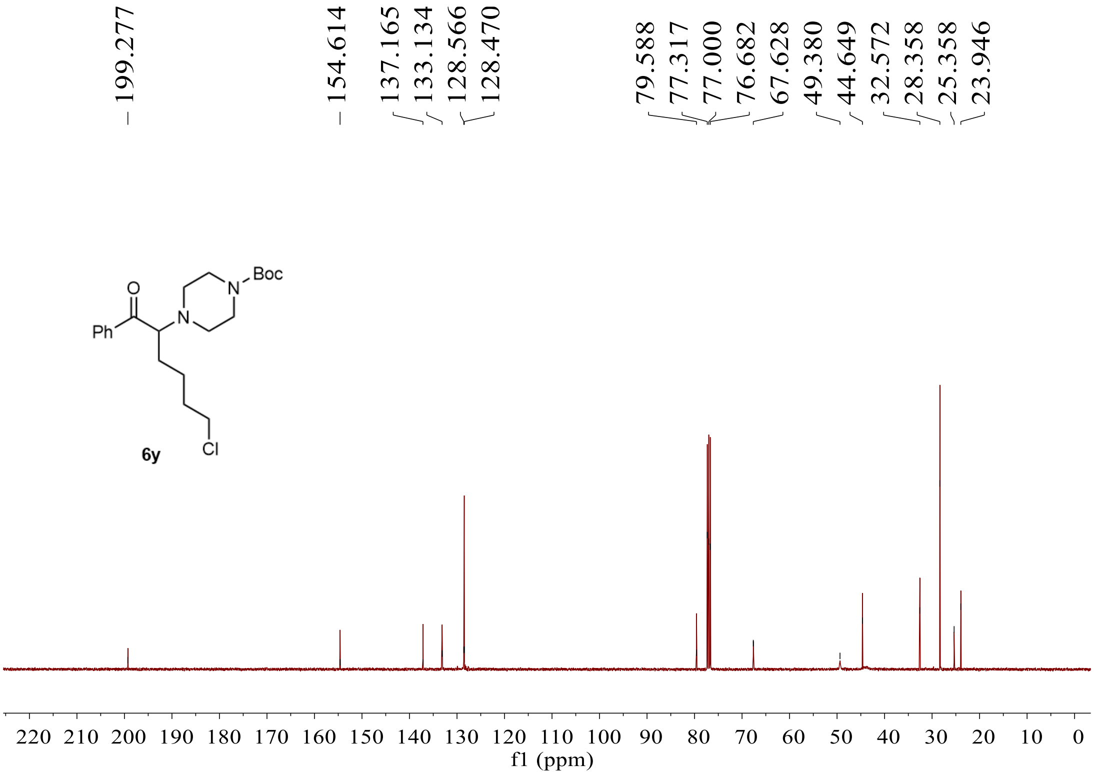
199.277
154.614
137.165
133.134
128.566
128.470
79.588
77.317
77.000
76.682
67.628
49.380
44.649
32.572
28.358
25.358
23.946
O
N
N
Boc
Ph
Cl
6y
220 210 200 190 180 170 160 150 140 130 120 110 100 90 80 70 60 50 40 30 20 10 0
f1 (ppm)

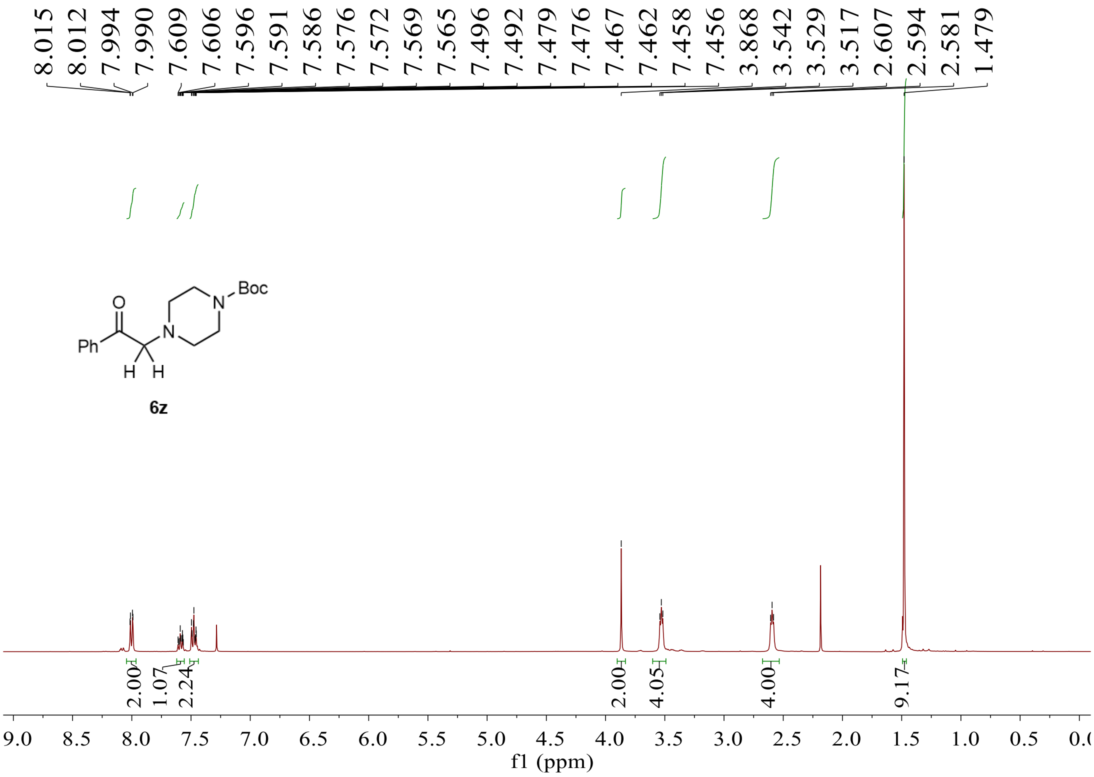
8.015 8.012 7.994 7.990 7.609 7.606 7.596 7.591 7.586 7.576 7.572 7.569 7.565 7.496 7.492 7.479 7.476 7.467 7.462 7.458 7.456 3.868 3.542 3.529 3.517 2.607 2.594 2.581 1.479
Boc
O
N
Ph
N
H H
6z
2.00
1.07
2.24
2.00
4.05
4.00
9.17
9.0 8.5 8.0 7.5 7.0 6.5 6.0 5.5 5.0 4.5 4.0 3.5 3.0 2.5 2.0 1.5 1.0 0.5
f1 (ppm)

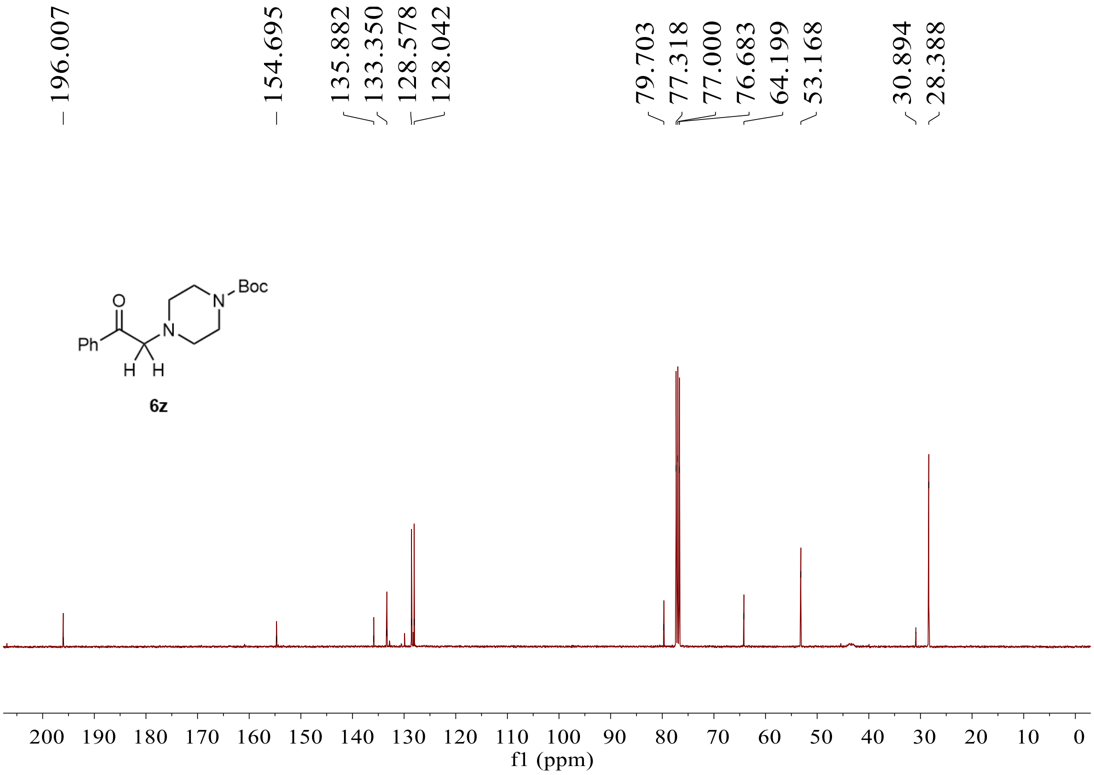
196.007
154.695
135.882 133.350 128.578 128.042
79.703 77.318 77.000 76.683 64.199 53.168
30.894 28.388
Boc
O
N
Ph
N
H H
6z
200 190 180 170 160 150 140 130 120 110 100 90 80 70 60 50 40 30 20 10 0
f1 (ppm)

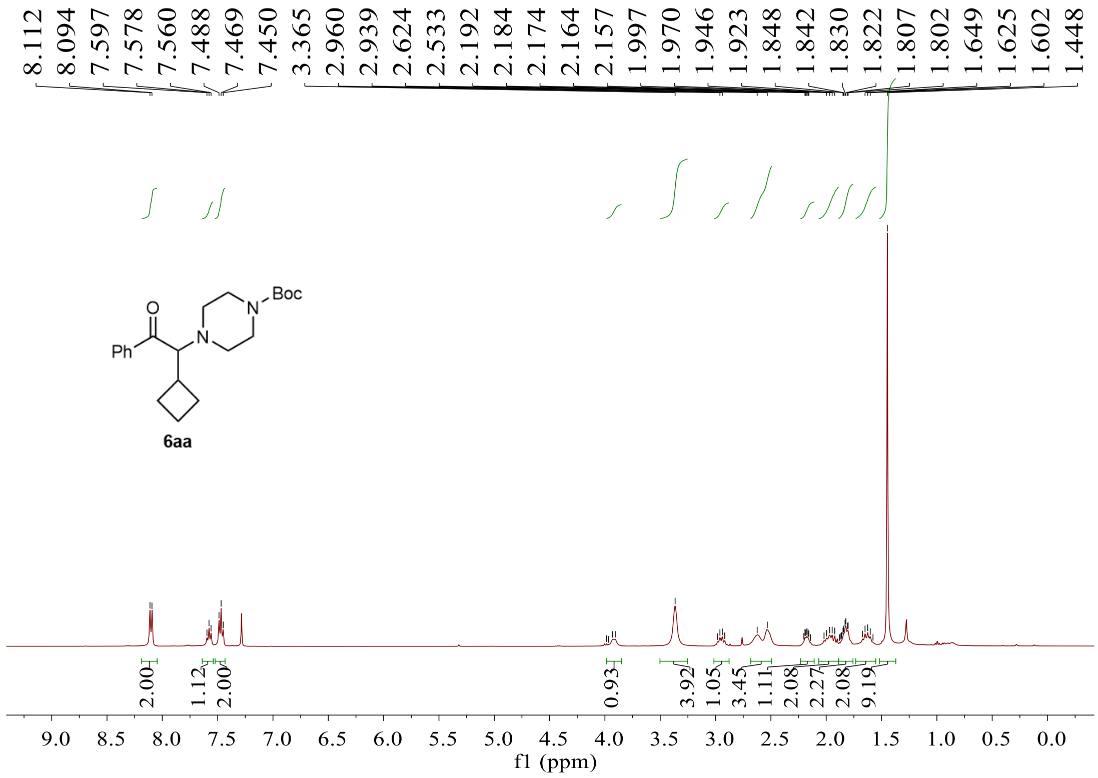
8.112
8.094
7.597
7.578
7.560
7.488
7.469
7.450
3.365
2.960
2.939
2.624
2.533
2.192
2.184
2.174
2.164
2.157
1.997
1.970
1.946
1.923
1.848
1.842
1.830
1.822
1.807
1.802
1.649
1.625
1.602
1.448
Boc
O
N
N
Ph
6aa
2.00
1.12
2.00
0.93
3.92
1.05
3.45
1.11
2.08
2.27
2.08
9.19
9.0 8.5 8.0 7.5 7.0 6.5 6.0 5.5 5.0 4.5 4.0 3.5 3.0 2.5 2.0 1.5 1.0 0.5 0.0
f1 (ppm)

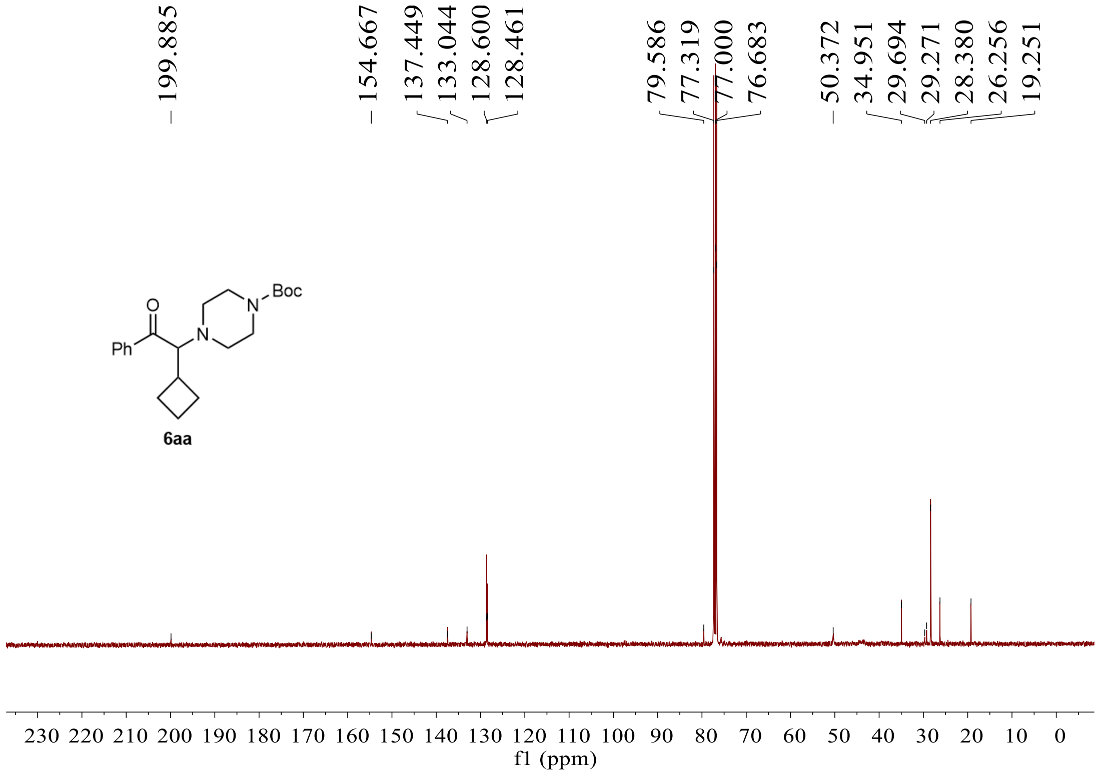
199.885
154.667
137.449
133.044
128.600
128.461
79.586
77.319
77.000
76.683
50.372
34.951
29.694
29.271
28.380
26.256
19.251
Boc
O
N
N
Ph
6aa
230 220 210 200 190 180 170 160 150 140 130 120 110 100 90 80 70 60 50 40 30 20 10 0
f1 (ppm)

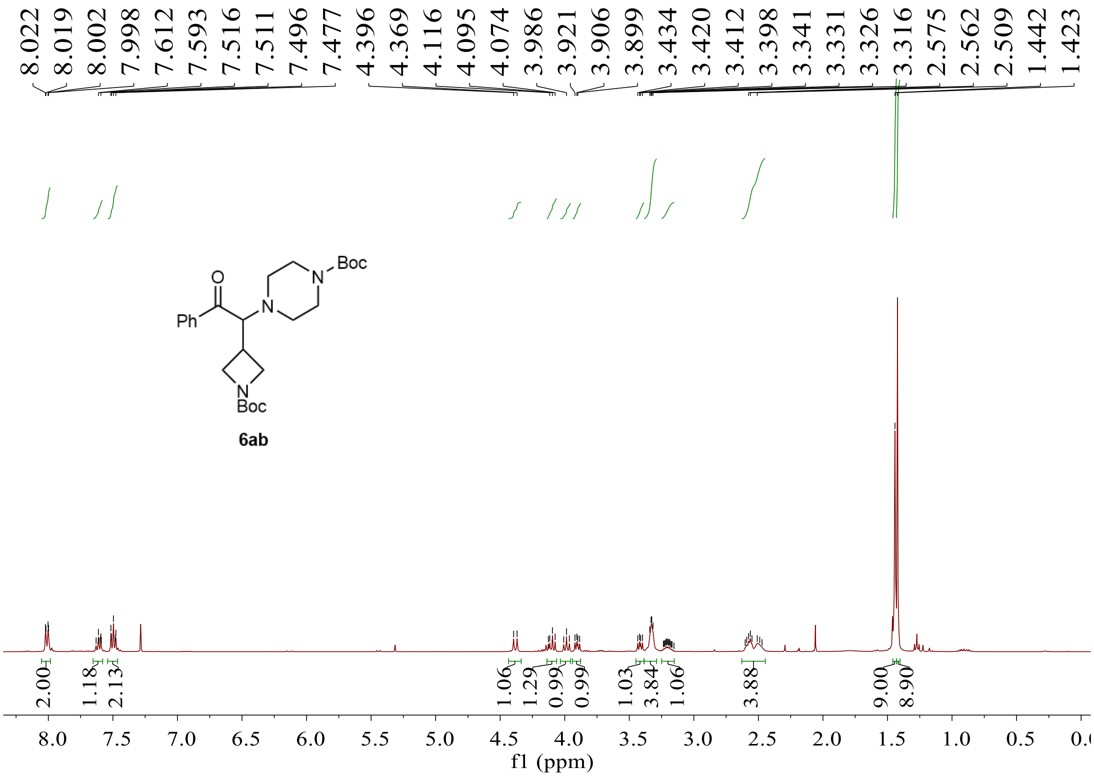
8.022 8.019 8.002 7.998 7.612 7.593 7.516 7.511 7.496 7.477 4.396 4.369 4.116 4.095 4.074 3.986 3.921 3.906 3.899 3.434 3.420 3.412 3.398 3.341 3.331 3.326 3.316 2.575 2.562 2.509 1.442 1.423
Ph
O
N
N
Boc
N
Boc
6ab
2.00 1.18 2.13 1.06 1.29 0.99 0.99 1.03 3.84 1.06 3.88 9.00 8.90
8.0 7.5 7.0 6.5 6.0 5.5 5.0 4.5 4.0 3.5 3.0 2.5 2.0 1.5 1.0 0.5 0.0
f1 (ppm)

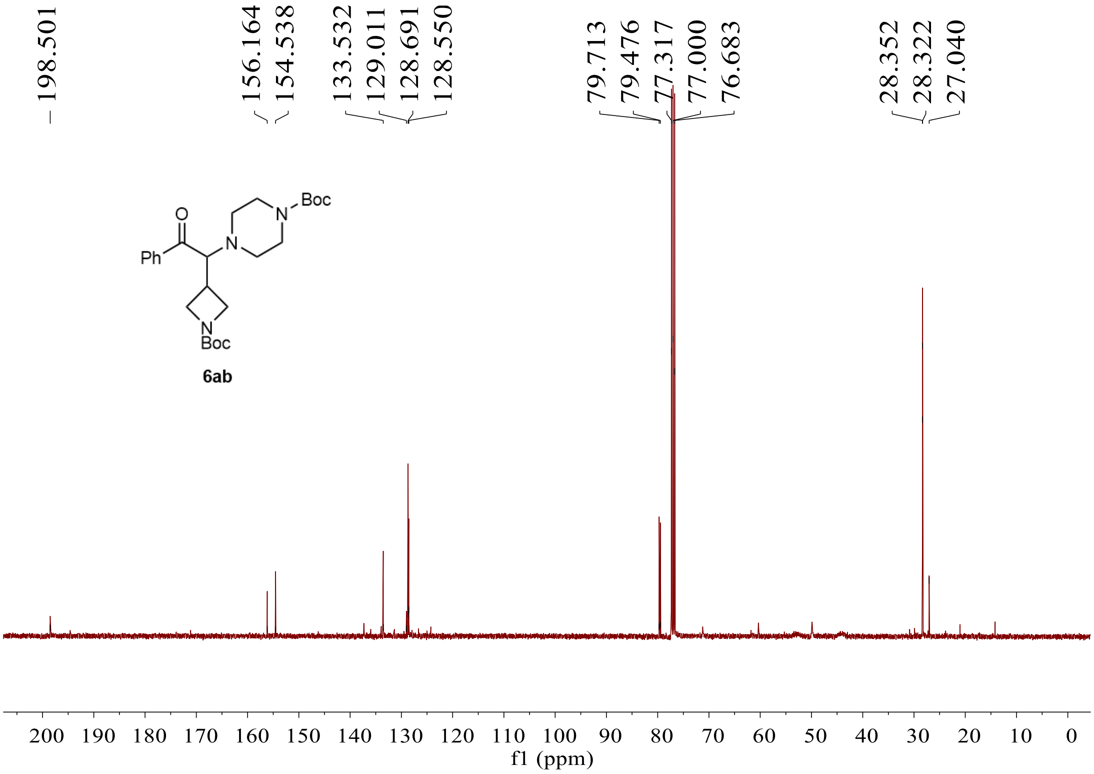
198.501
156.164 154.538
133.532 129.011 128.691 128.550
79.713 79.476 77.317 77.000 76.683
28.352 28.322 27.040
Ph
O
N
N
Boc
N
Boc
6ab
200 190 180 170 160 150 140 130 120 110 100 90 80 70 60 50 40 30 20 10 0
f1 (ppm)

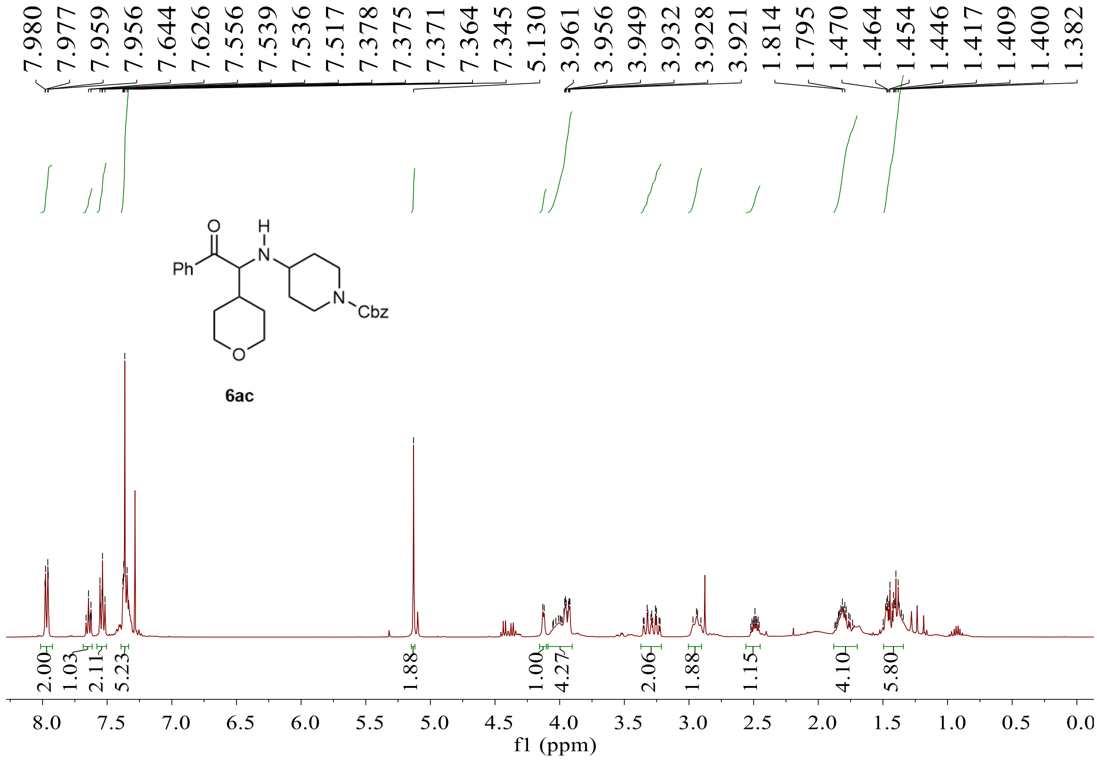

7.980
7.977
7.959
7.956
7.644
7.626
7.556
7.539
7.536
7.517
7.378
7.375
7.371
7.364
7.345
5.130
3.961
3.956
3.949
3.932
3.928
3.921
1.814
1.795
1.470
1.464
1.454
1.446
1.417
1.409
1.400
1.382
Ph
O
H
N
N
Cbz
O
2.00
1.03
2.11
5.23
1.88
1.00
4.27
2.06
1.88
1.15
4.10
5.80
8.0 7.5 7.0 6.5 6.0 5.5 5.0 4.5 4.0 3.5 3.0 2.5 2.0 1.5 1.0 0.5 0.0
f1 (ppm)

6ac

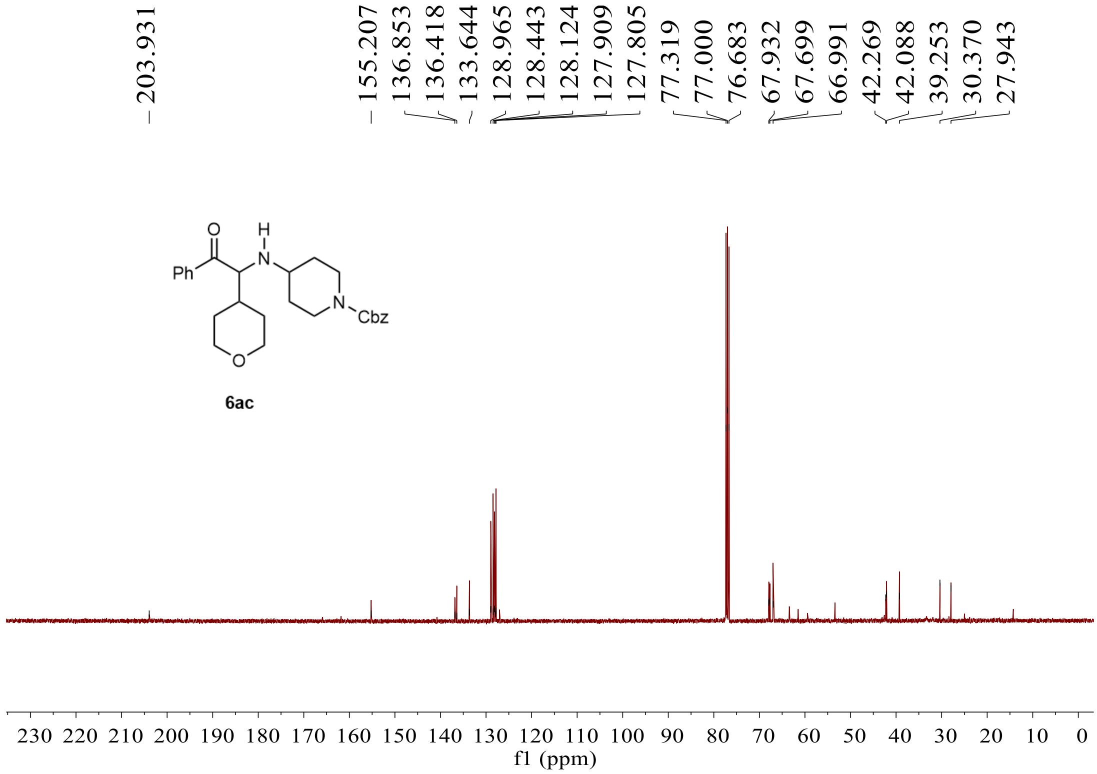

203.931
155.207
136.853
136.418
133.644
128.965
128.443
128.124
127.909
127.805
77.319
77.000
76.683
67.932
67.699
66.991
42.269
42.088
39.253
30.370
27.943
Ph
O
H
N
N
Cbz
O
230 220 210 200 190 180 170 160 150 140 130 120 110 100 90 80 70 60 50 40 30 20 10 0
f1 (ppm)

6ac

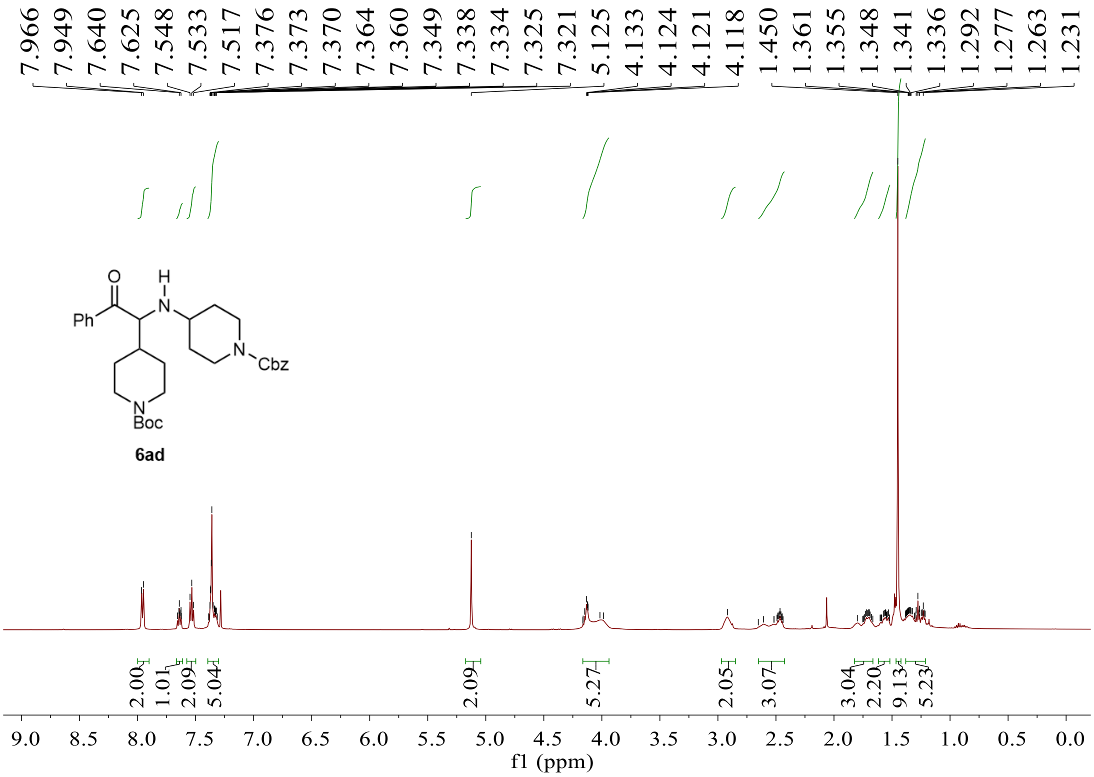
7.966
7.949
7.640
7.625
7.548
7.533
7.517
7.376
7.373
7.370
7.364
7.360
7.349
7.338
7.334
7.325
7.321
5.125
4.133
4.124
4.121
4.118
1.450
1.361
1.355
1.348
1.341
1.336
1.292
1.277
1.263
1.231
Ph
O
H
N
N
Cbz
N
Boc
6ad
2.00
1.01
2.09
5.04
2.09
5.27
2.05
3.07
3.04
2.20
9.13
5.23
9.0 8.5 8.0 7.5 7.0 6.5 6.0 5.5 5.0 4.5 4.0 3.5 3.0 2.5 2.0 1.5 1.0 0.5 0.0
f1 (ppm)

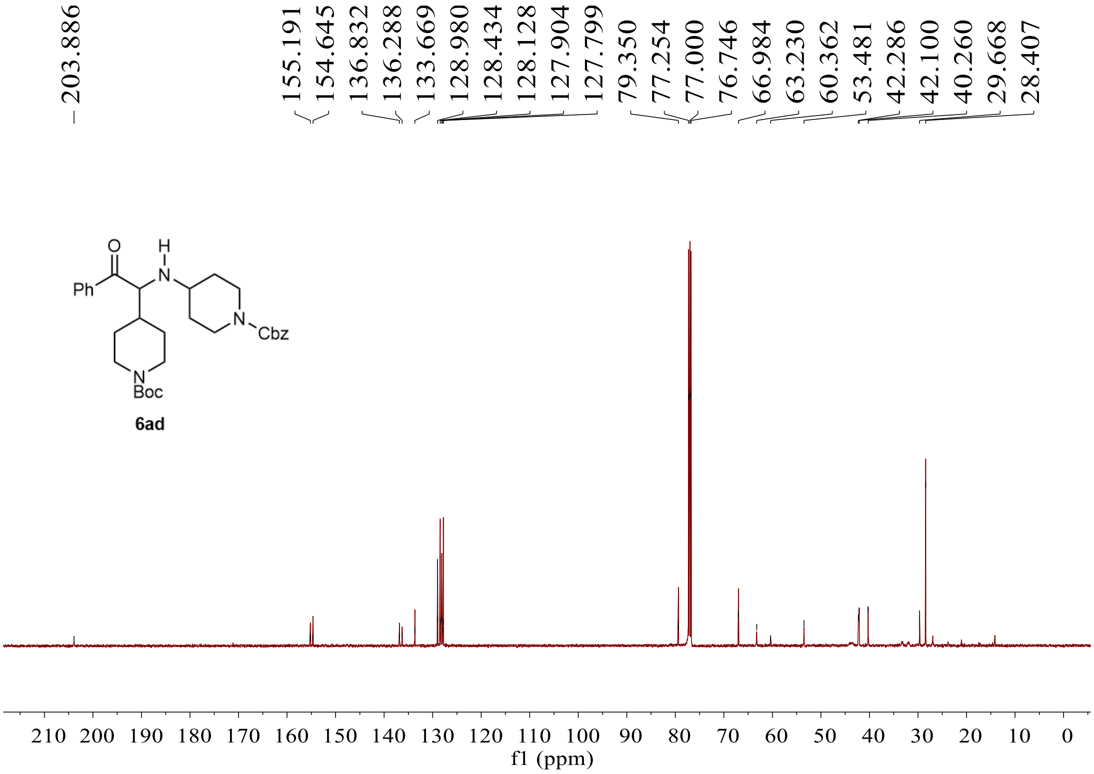
203.886
155.191
154.645
136.832
136.288
133.669
128.980
128.434
128.128
127.904
127.799
79.350
77.254
77.000
76.746
66.984
63.230
60.362
53.481
42.286
42.100
40.260
29.668
28.407
Ph
O
H
N
N
Cbz
N
Boc
6ad
210 200 190 180 170 160 150 140 130 120 110 100 90 80 70 60 50 40 30 20 10 0
f1 (ppm)

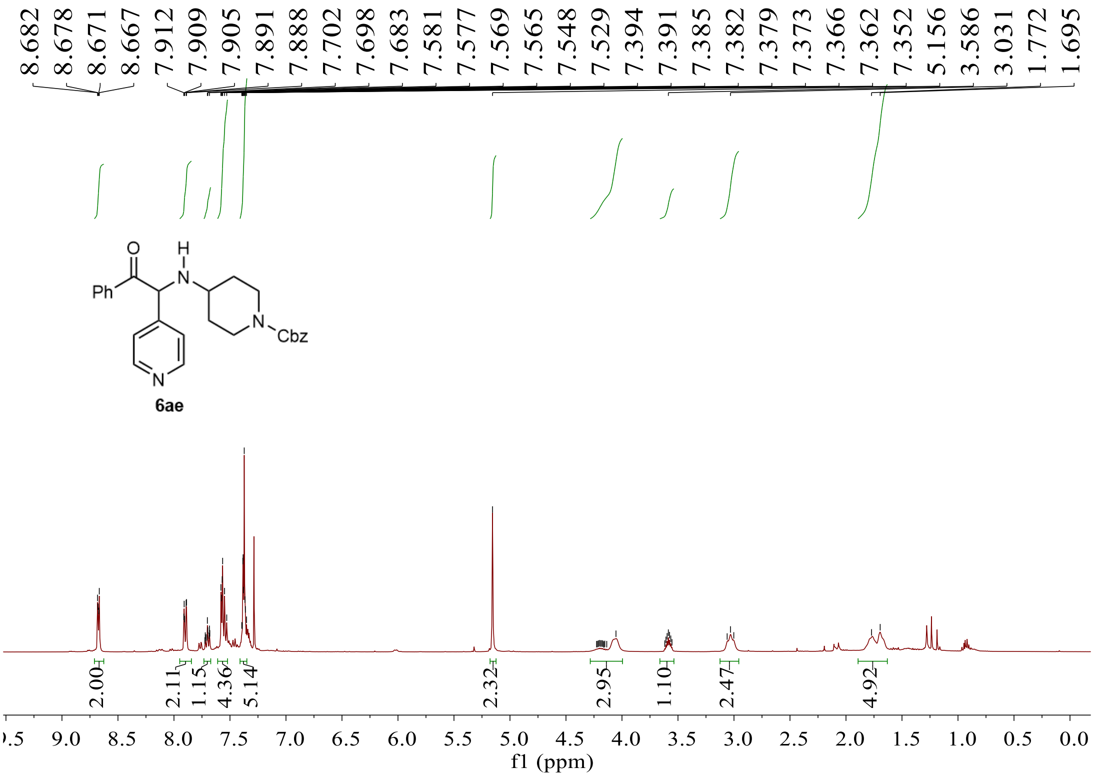
8.682 8.678 8.671 8.667 7.912 7.909 7.905 7.891 7.888 7.702 7.698 7.683 7.581 7.577 7.569 7.565 7.548 7.529 7.394 7.391 7.385 7.382 7.379 7.373 7.366 7.362 7.352 5.156 3.586 3.031 1.772 1.695
Ph
O
H
N
N
Cbz
N
6ae
2.00 2.11 1.15 4.36 5.14 2.32 2.95 1.10 2.47 4.92
f1 (ppm)

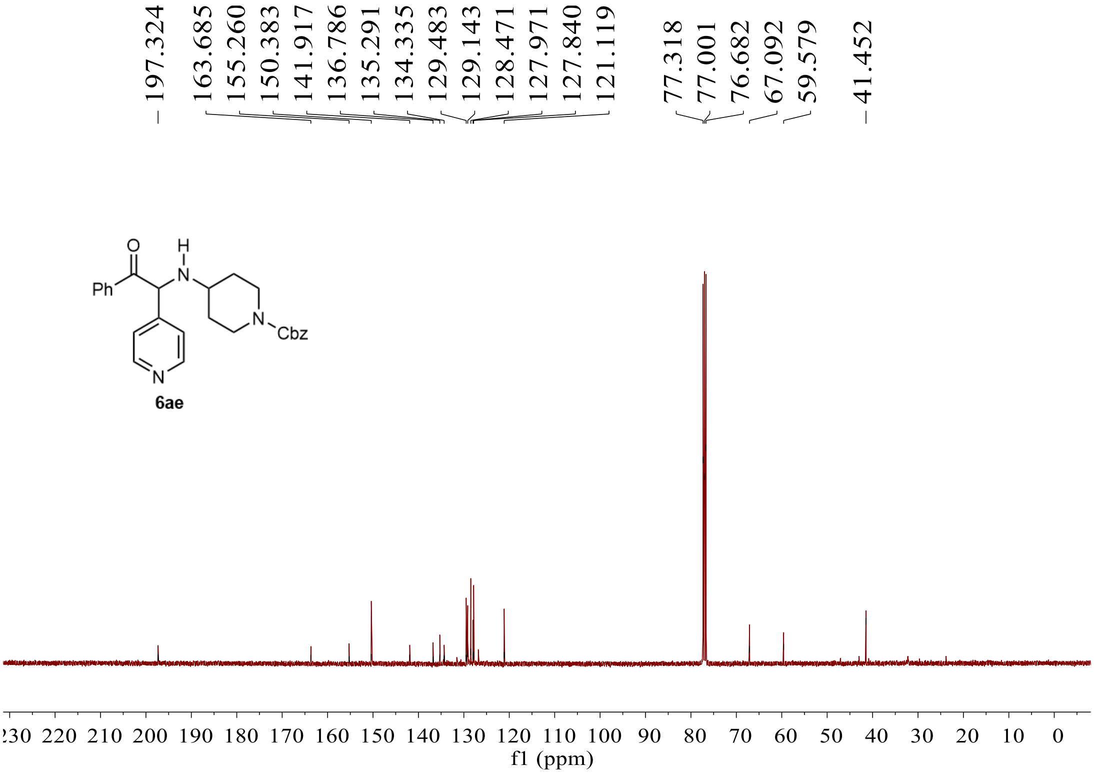
197.324 163.685 155.260 150.383 141.917 136.786 135.291 134.335 129.483 129.143 128.471 127.971 127.840 121.119 77.318 77.001 76.682 67.092 59.579 41.452
Ph
O
H
N
N
Cbz
N
6ae
f1 (ppm)

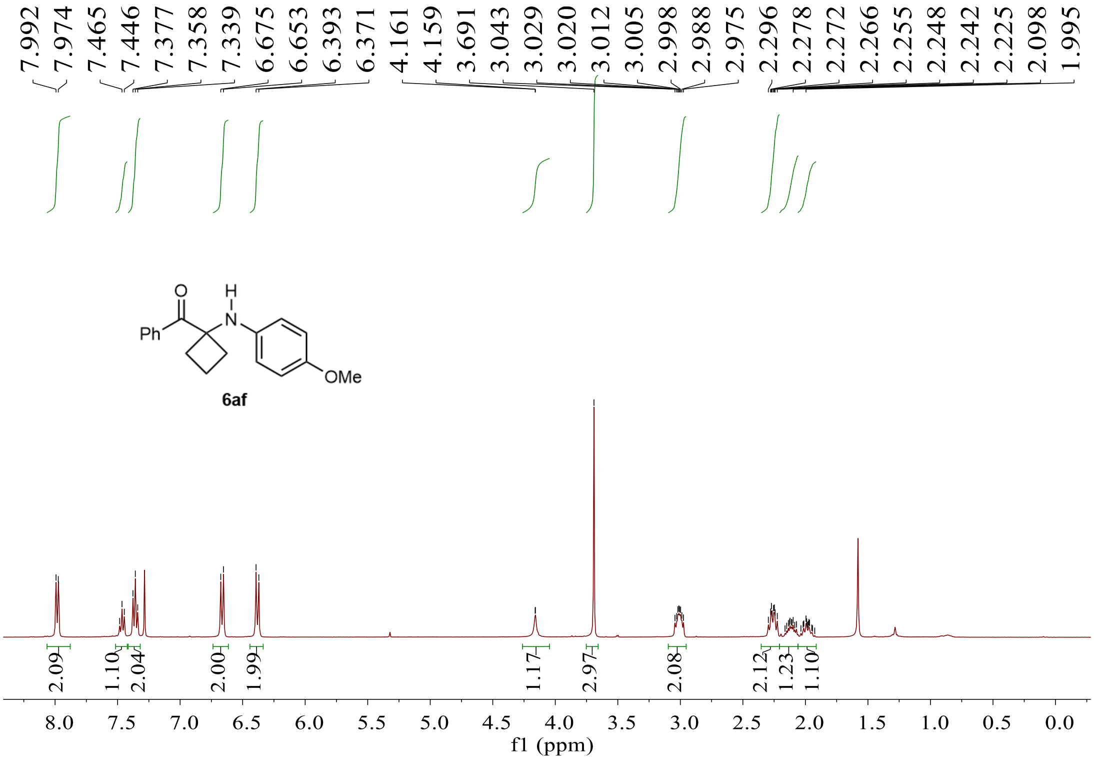
7.992 7.974 7.465 7.446 7.377 7.358 7.339 6.675 6.653 6.393 6.371 4.161 4.159 3.691 3.043 3.029 3.020 3.012 3.005 2.998 2.988 2.975 2.296 2.278 2.272 2.266 2.255 2.248 2.242 2.225 2.098 1.995
Ph
O
H
N
OMe
6af
2.09 1.10 2.04 2.00 1.99 1.17 2.97 2.08 2.12 1.23 1.10
8.0 7.5 7.0 6.5 6.0 5.5 5.0 4.5 4.0 3.5 3.0 2.5 2.0 1.5 1.0 0.5 0.0
f1 (ppm)

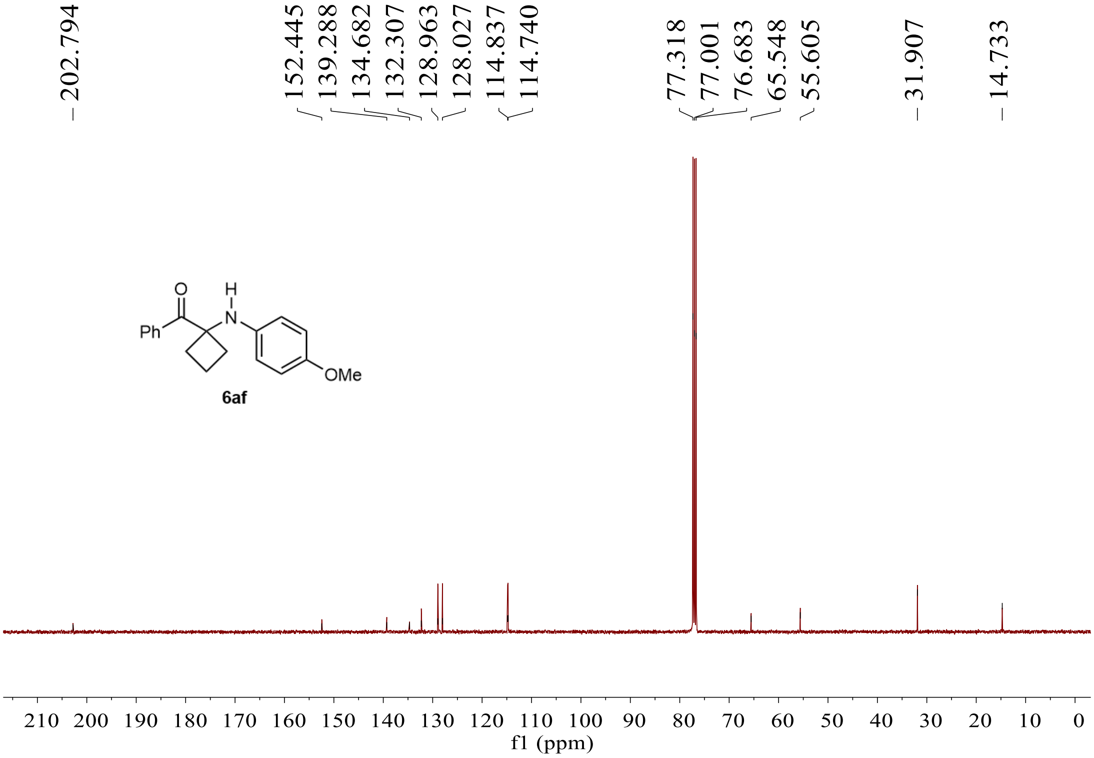
202.794
152.445 139.288 134.682 132.307 128.963 128.027 114.837 114.740
77.318 77.001 76.683 65.548 55.605
31.907
14.733
Ph
O
H
N
OMe
6af
210 200 190 180 170 160 150 140 130 120 110 100 90 80 70 60 50 40 30 20 10 0
f1 (ppm)

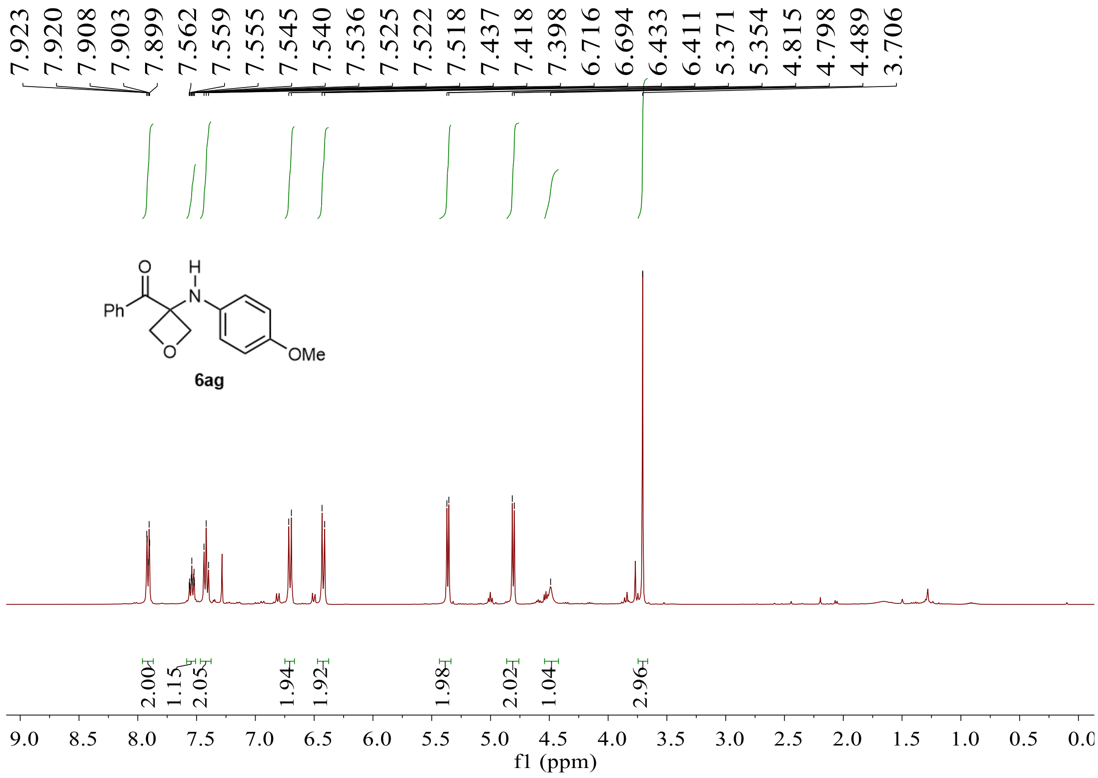
7.923
7.920
7.908
7.903
7.899
7.562
7.559
7.555
7.545
7.540
7.536
7.525
7.522
7.518
7.437
7.418
7.398
6.716
6.694
6.433
6.411
5.371
5.354
4.815
4.798
4.489
3.706
Ph
O
H
N
O
OMe
6ag
2.00
1.15
2.05
1.94
1.92
1.98
2.02
1.04
2.96
f1 (ppm)

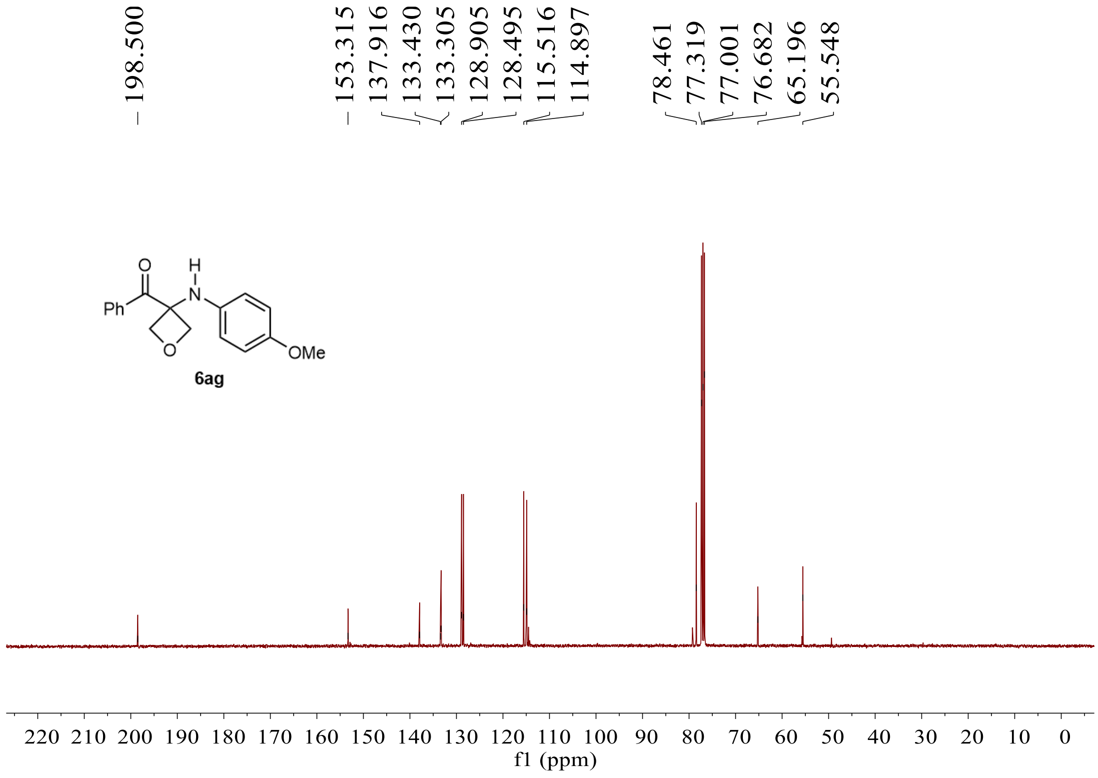
198.500
153.315
137.916
133.430
133.305
128.905
128.495
115.516
114.897
78.461
77.319
77.001
76.682
65.196
55.548
Ph
O
H
N
O
OMe
6ag
f1 (ppm)

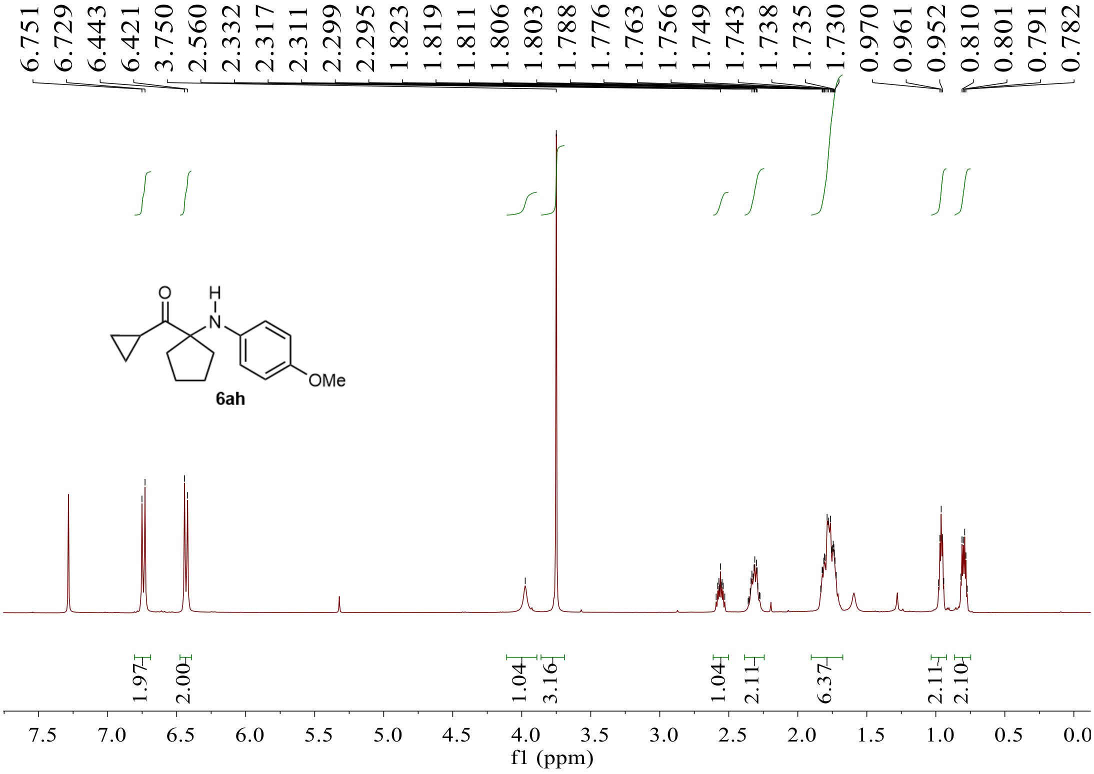
6.751
6.729
6.443
6.421
3.750
2.560
2.332
2.317
2.311
2.299
2.295
1.823
1.819
1.811
1.806
1.803
1.788
1.776
1.763
1.756
1.749
1.743
1.738
1.735
1.730
0.970
0.961
0.952
0.810
0.801
0.791
0.782
O
H
N
OMe
6ah
1.97
2.00
1.04
3.16
1.04
2.11
6.37
2.11
2.10
7.5 7.0 6.5 6.0 5.5 5.0 4.5 4.0 3.5 3.0 2.5 2.0 1.5 1.0 0.5 0.0
f1 (ppm)

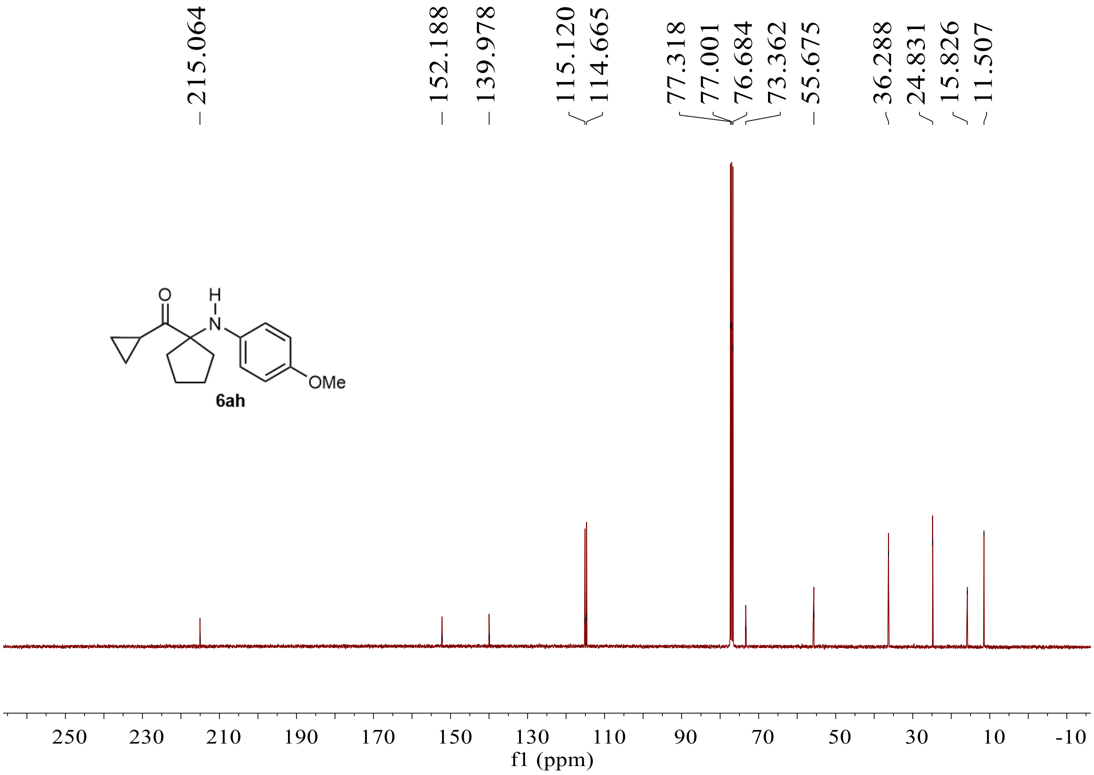
215.064
152.188
139.978
115.120
114.665
77.318
77.001
76.684
73.362
55.675
36.288
24.831
15.826
11.507
O
H
N
OMe
6ah
250 230 210 190 170 150 130 110 90 70 50 30 10 -10
f1 (ppm)

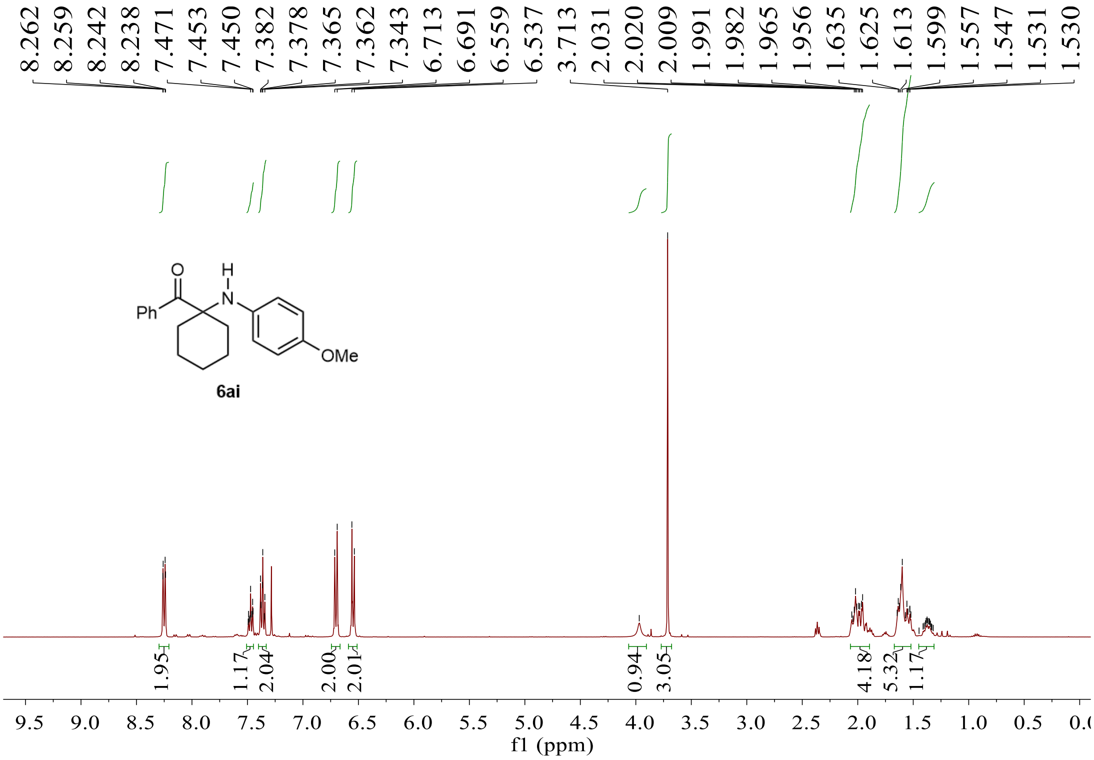
8.262 8.259 8.242 8.238 7.471 7.453 7.450 7.382 7.378 7.365 7.362 7.343 6.713 6.691 6.559 6.537 3.713 2.031 2.020 2.009 1.991 1.982 1.965 1.956 1.635 1.625 1.613 1.599 1.557 1.547 1.531 1.530
Ph
O
H
N
OMe
6ai
1.95 1.17 2.04 2.00 2.01 0.94 3.05 4.18 5.32 1.17
9.5 9.0 8.5 8.0 7.5 7.0 6.5 6.0 5.5 5.0 4.5 4.0 3.5 3.0 2.5 2.0 1.5 1.0 0.5
f1 (ppm)

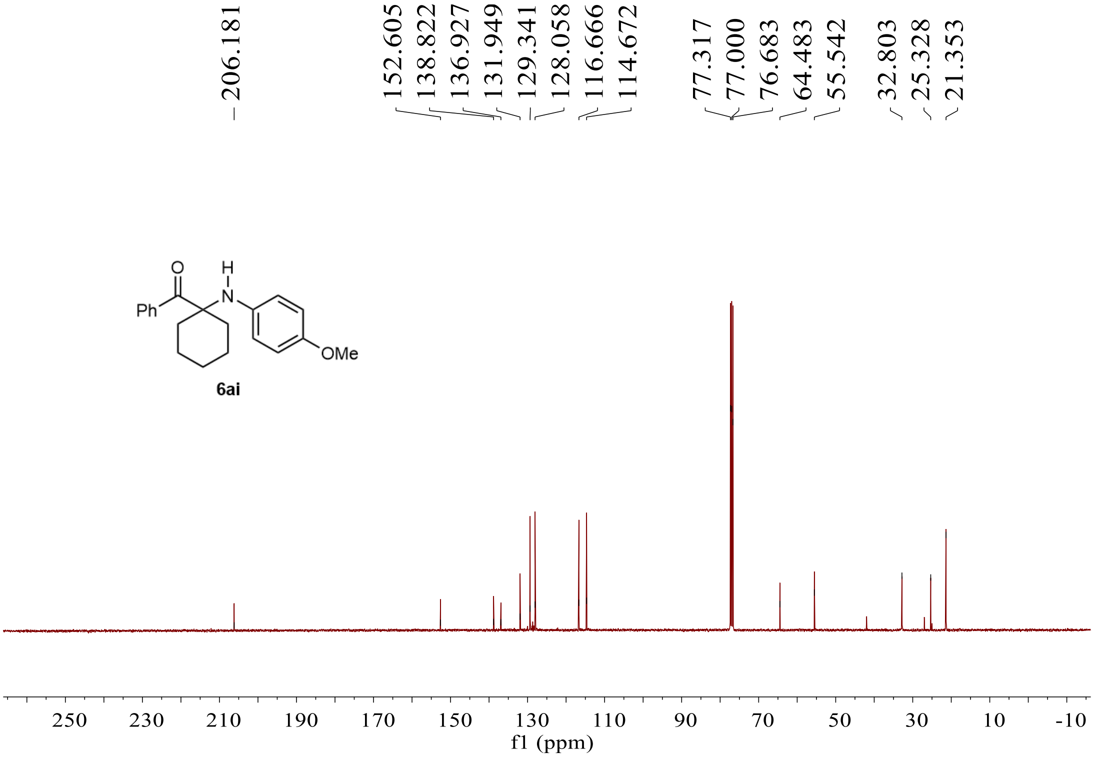
206.181
152.605 138.822 136.927 131.949 129.341 128.058 116.666 114.672
77.317 77.000 76.683 64.483 55.542
32.803 25.328 21.353
Ph
O
H
N
OMe
6ai
250 230 210 190 170 150 130 110 90 70 50 30 10 -10
f1 (ppm)

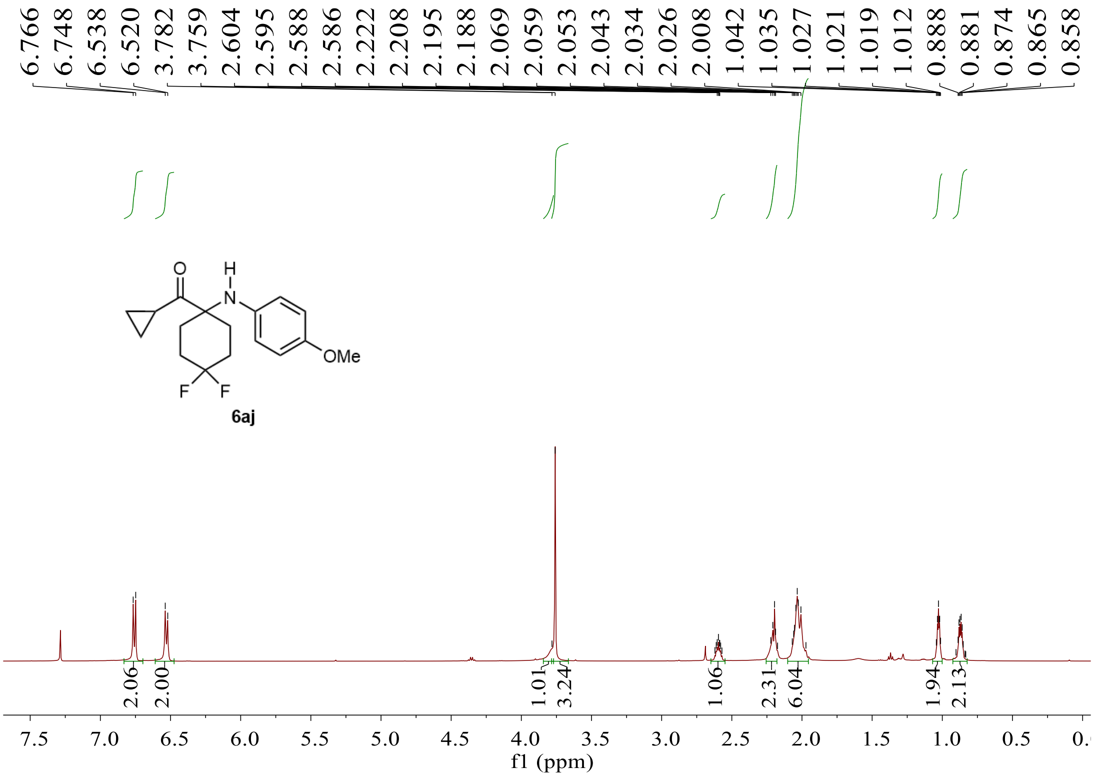

6.766
6.748
6.538
6.520
3.782
3.759
2.604
2.595
2.588
2.586
2.222
2.208
2.195
2.188
2.069
2.059
2.053
2.043
2.034
2.026
2.008
1.042
1.035
1.027
1.021
1.019
1.012
0.888
0.881
0.874
0.865
0.858
OMe
F
F
6aj
2.06
2.00
1.01
3.24
1.06
2.31
6.04
1.94
2.13
f1 (ppm)

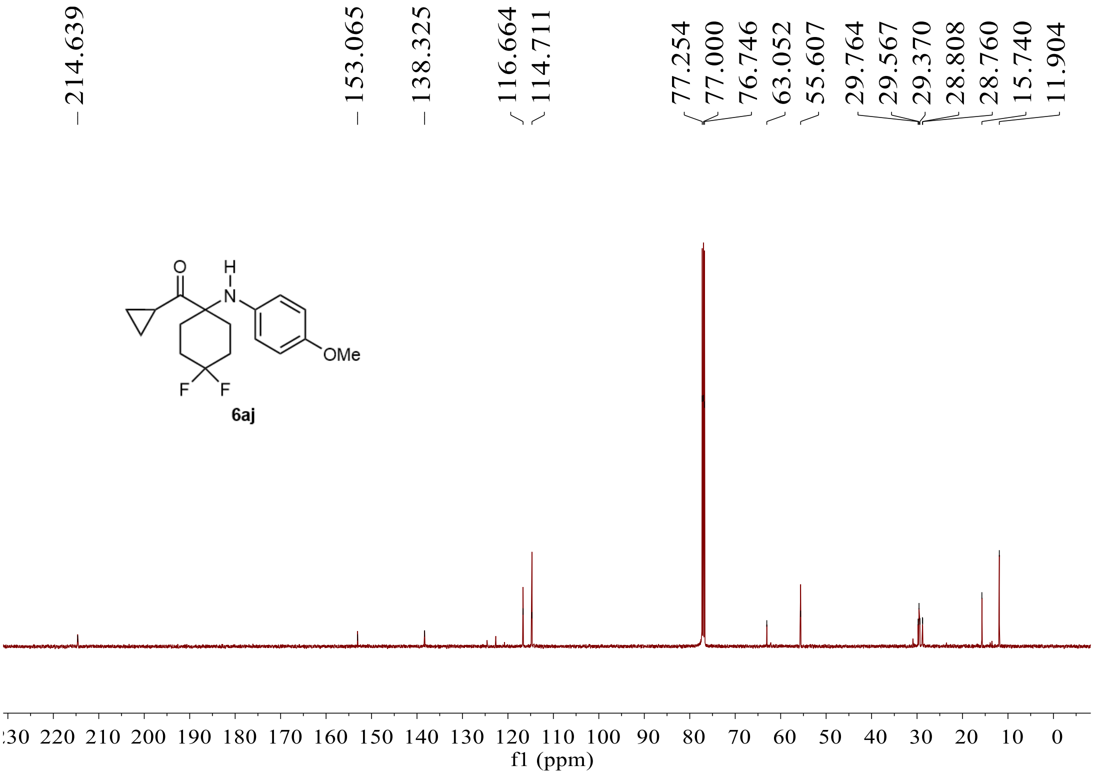

214.639
153.065
138.325
116.664
114.711
77.254
77.000
76.746
63.052
55.607
29.764
29.567
29.370
28.808
28.760
15.740
11.904
OMe
F
F
6aj
f1 (ppm)

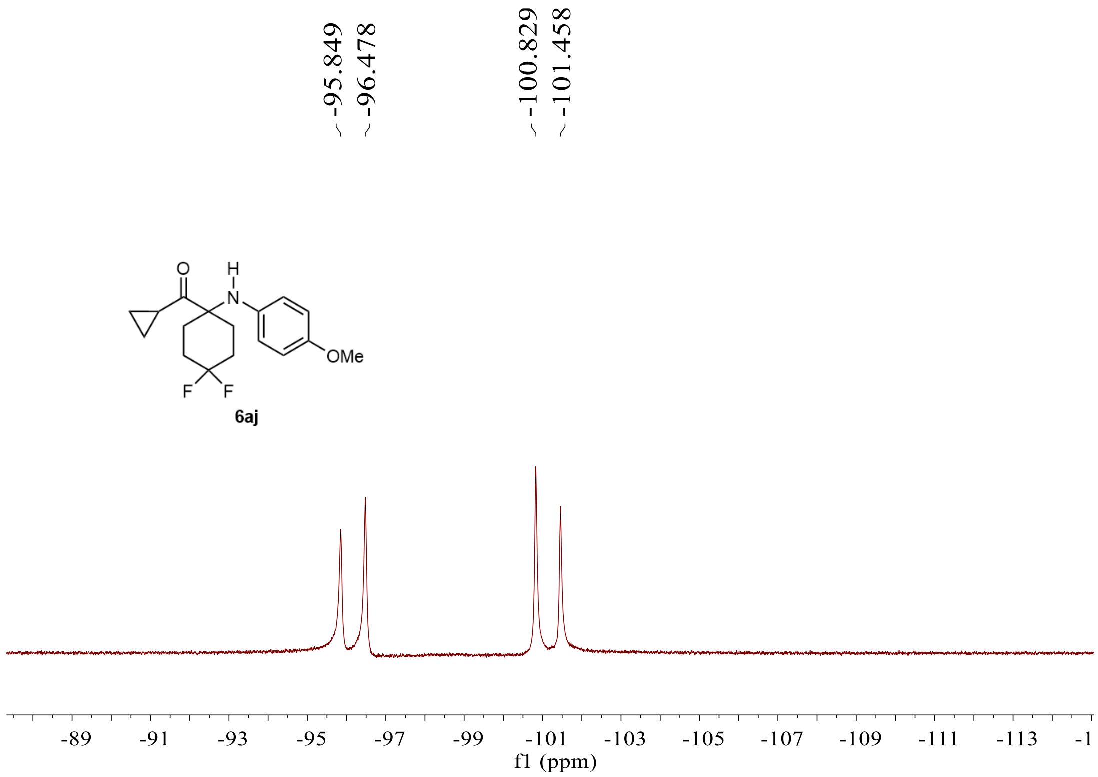
-95.849
-96.478
-100.829
-101.458
O
H
N
OMe
F
F
6aj
-89
-91
-93
-95
-97
-99
-101
-103
-105
-107
-109
-111
-113
f1 (ppm)

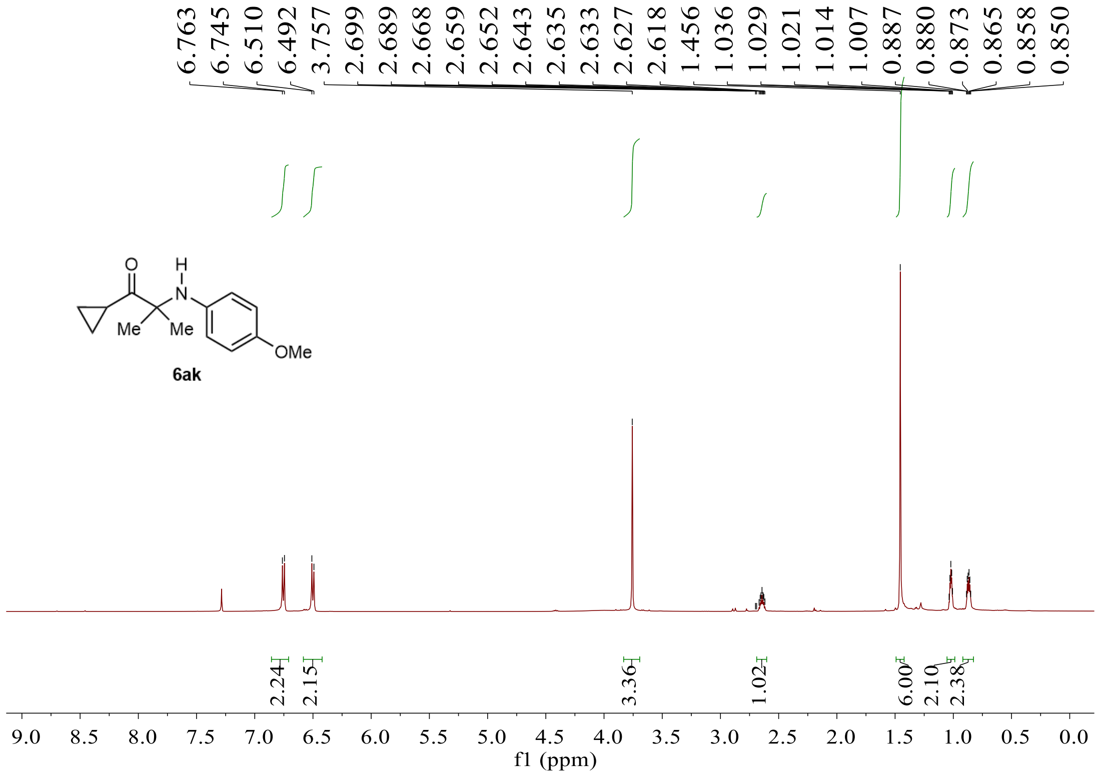
6.763
6.745
6.510
6.492
3.757
2.699
2.689
2.668
2.659
2.652
2.643
2.635
2.633
2.627
2.618
1.456
1.036
1.029
1.021
1.014
1.007
0.887
0.880
0.873
0.865
0.858
0.850
O
H
N
Me
Me
OMe
6ak
2.24
2.15
3.36
1.02
6.00
2.10
2.38
9.0
8.5
8.0
7.5
7.0
6.5
6.0
5.5
5.0
4.5
4.0
3.5
3.0
2.5
2.0
1.5
1.0
0.5
0.0
f1 (ppm)

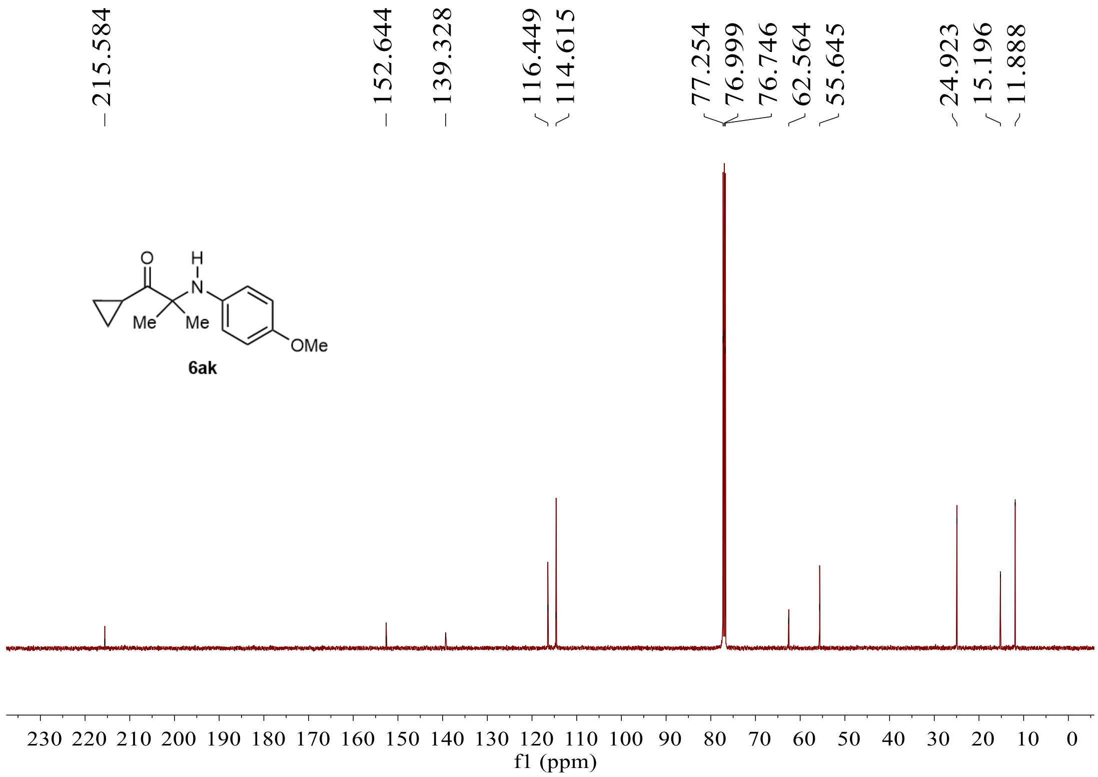
215.584
152.644
139.328
116.449
114.615
77.254
76.999
76.746
62.564
55.645
24.923
15.196
11.888
O
H
N
Me
Me
OMe
6ak
230 220 210 200 190 180 170 160 150 140 130 120 110 100 90 80 70 60 50 40 30 20 10 0
f1 (ppm)

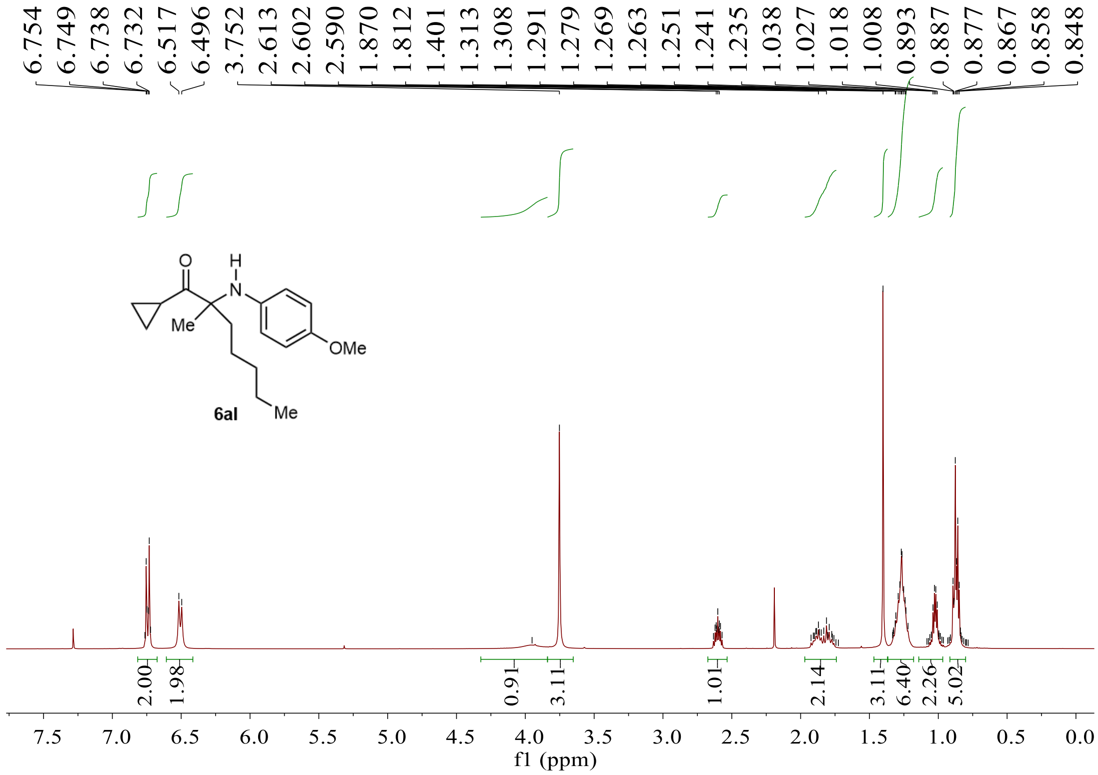
6.754
6.749
6.738
6.732
6.517
6.496
3.752
2.613
2.602
2.590
1.870
1.812
1.401
1.313
1.308
1.291
1.279
1.269
1.263
1.251
1.241
1.235
1.038
1.027
1.018
1.008
0.893
0.887
0.877
0.867
0.858
0.848
O
H
N
Me
OMe
6al
Me
2.00
1.98
0.91
3.11
1.01
2.14
3.11
6.40
2.26
5.02
7.5 7.0 6.5 6.0 5.5 5.0 4.5 4.0 3.5 3.0 2.5 2.0 1.5 1.0 0.5 0.0
f1 (ppm)

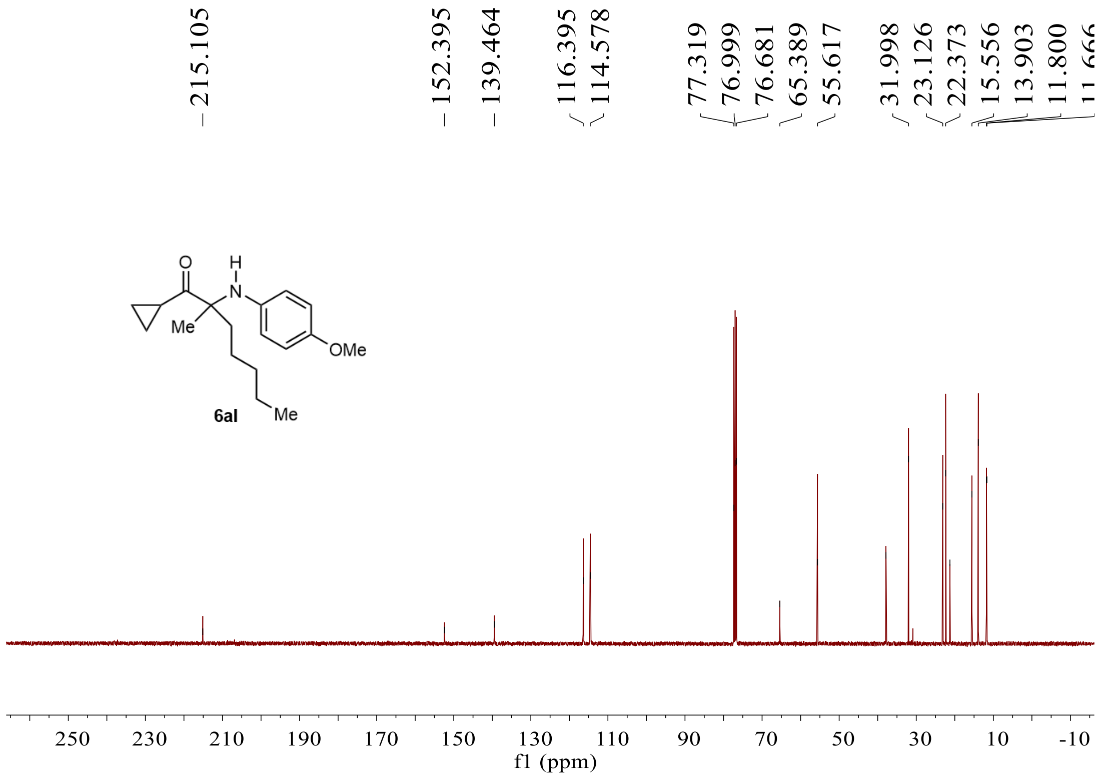
215.105
152.395
139.464
116.395
114.578
77.319
76.999
76.681
65.389
55.617
31.998
23.126
22.373
15.556
13.903
11.800
11.666
O
H
N
Me
OMe
6al
Me
250 230 210 190 170 150 130 110 90 70 50 30 10 -10
f1 (ppm)

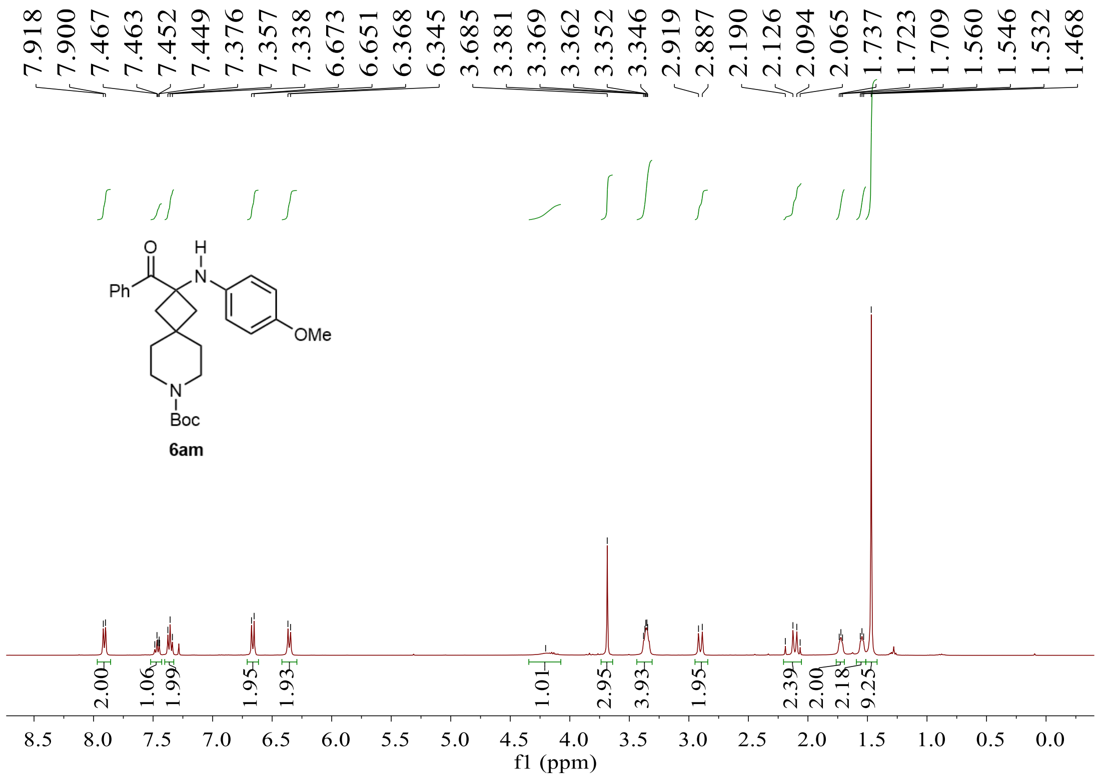
7.918
7.900
7.467
7.463
7.452
7.449
7.376
7.357
7.338
6.673
6.651
6.368
6.345
3.685
3.381
3.369
3.362
3.352
3.346
2.919
2.887
2.190
2.126
2.094
2.065
1.737
1.723
1.709
1.560
1.546
1.532
1.468
O
H
Ph
N
OMe
N
Boc
6am
2.00
1.06
1.99
1.95
1.93
1.01
2.95
3.93
1.95
2.39
2.00
2.18
9.25
8.5 8.0 7.5 7.0 6.5 6.0 5.5 5.0 4.5 4.0 3.5 3.0 2.5 2.0 1.5 1.0 0.5 0.0
f1 (ppm)

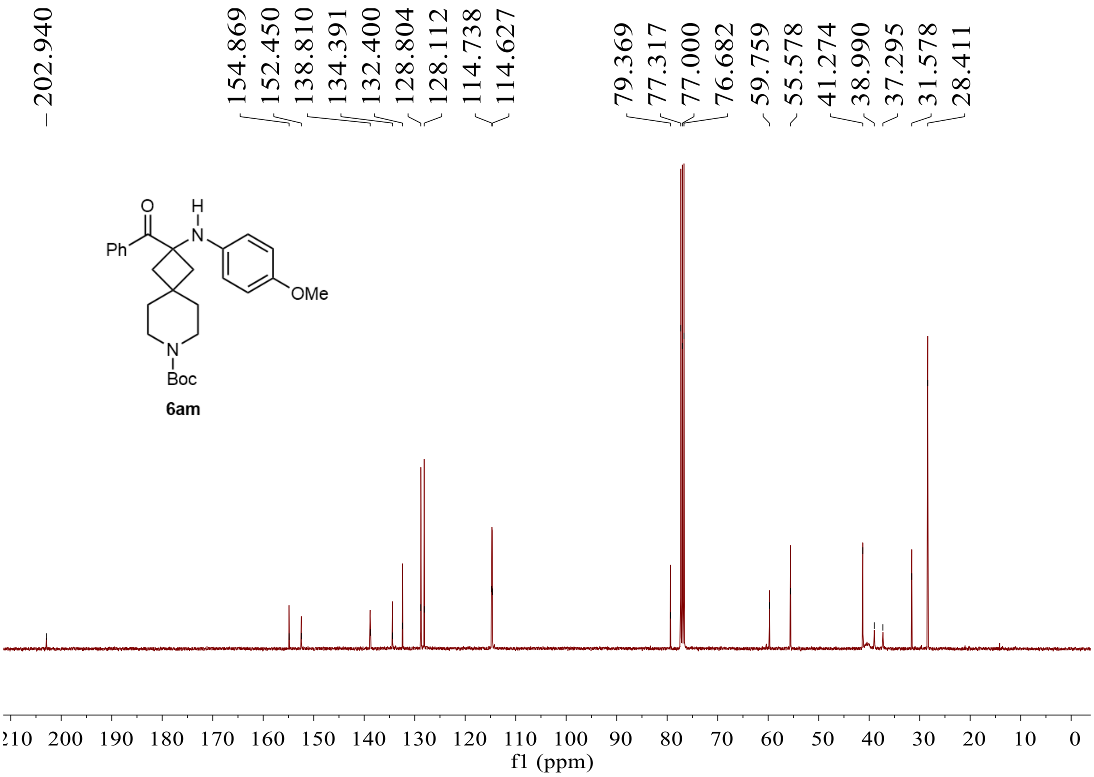

202.940
154.869
152.450
138.810
134.391
132.400
128.804
128.112
114.738
114.627
79.369
77.317
77.000
76.682
59.759
55.578
41.274
38.990
37.295
31.578
28.411
O
H
N
Ph
OMe
N
Boc
6am
210 200 190 180 170 160 150 140 130 120 110 100 90 80 70 60 50 40 30 20 10 0
f1 (ppm)

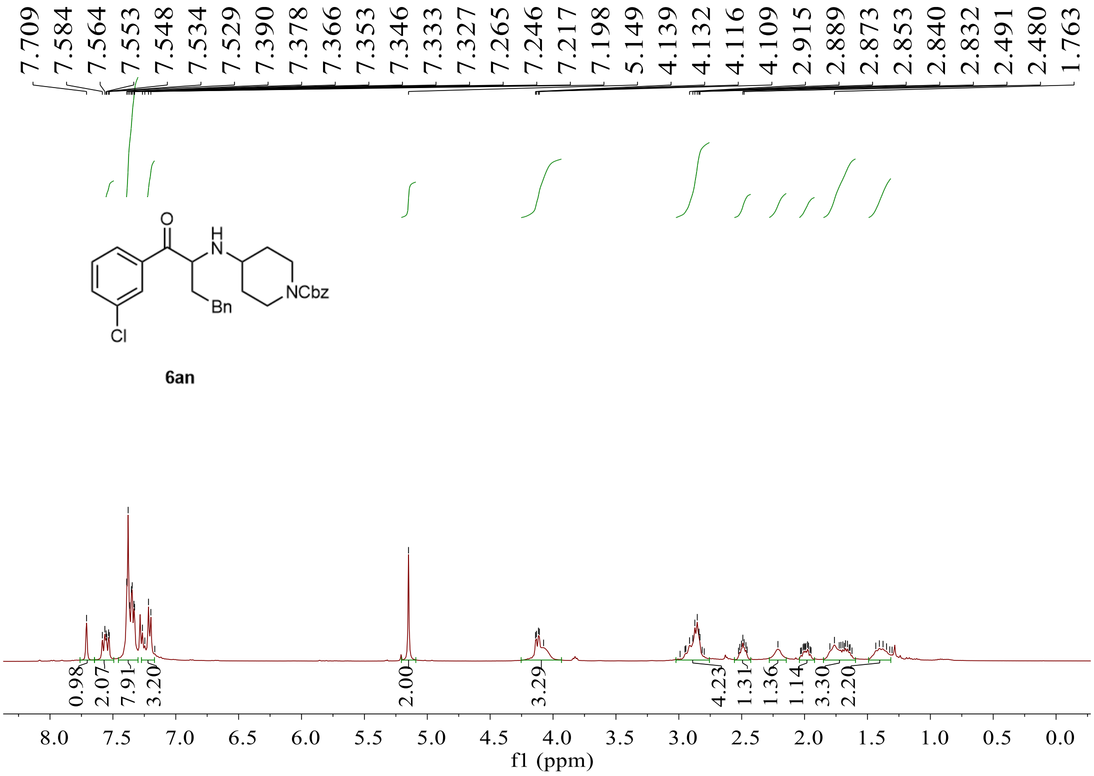

7.709
7.584
7.564
7.553
7.548
7.534
7.529
7.390
7.378
7.366
7.353
7.346
7.333
7.327
7.265
7.246
7.217
7.198
5.149
4.139
4.132
4.116
4.109
2.915
2.889
2.873
2.853
2.840
2.832
2.491
2.480
1.763
O
H
N
Cl
Bn
NCbz
6an
0.98
2.07
7.91
3.20
2.00
3.29
4.23
1.31
1.36
1.14
3.30
2.20
8.0 7.5 7.0 6.5 6.0 5.5 5.0 4.5 4.0 3.5 3.0 2.5 2.0 1.5 1.0 0.5 0.0
f1 (ppm)

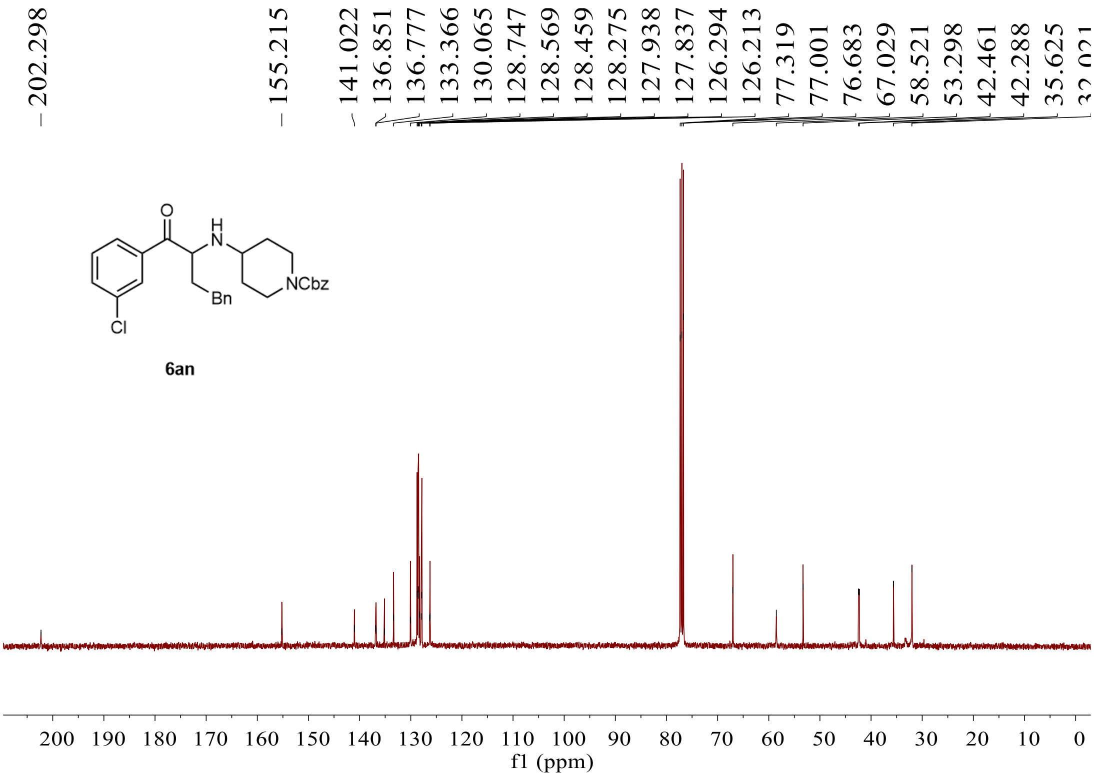

202.298
155.215
141.022
136.851
136.777
133.366
130.065
128.747
128.569
128.459
128.275
127.938
127.837
126.294
126.213
77.319
77.001
76.683
67.029
58.521
53.298
42.461
42.288
35.625
6an
NCbz
Bn
Cl
O
H
N
200 190 180 170 160 150 140 130 120 110 100 90 80 70 60 50 40 30 20 10 0
f1 (ppm)

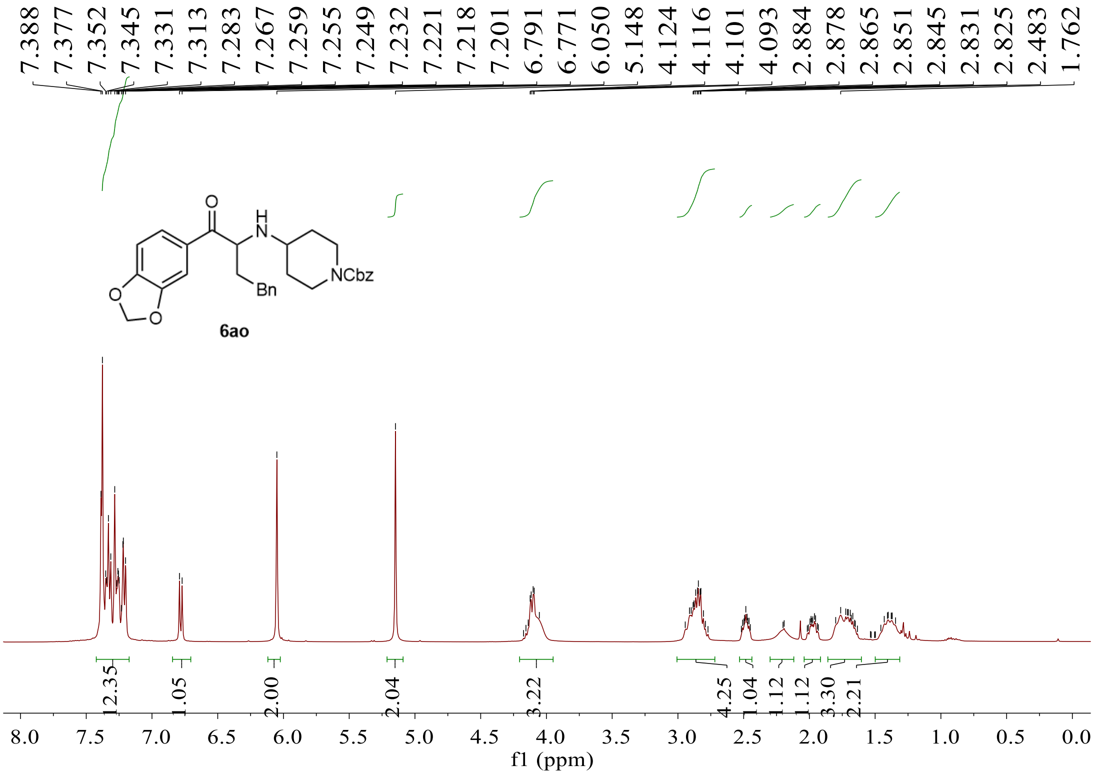

7.388
7.377
7.352
7.345
7.331
7.313
7.283
7.267
7.259
7.255
7.249
7.232
7.221
7.218
7.201
6.791
6.771
6.050
5.148
4.124
4.116
4.101
4.093
2.884
2.878
2.865
2.851
2.845
2.831
2.825
2.483
1.762
6ao
NCbz
Bn
O
H
N
12.35
1.05
2.00
2.04
3.22
4.25
1.04
1.12
1.12
3.30
2.21
8.0 7.5 7.0 6.5 6.0 5.5 5.0 4.5 4.0 3.5 3.0 2.5 2.0 1.5 1.0 0.5 0.0
f1 (ppm)

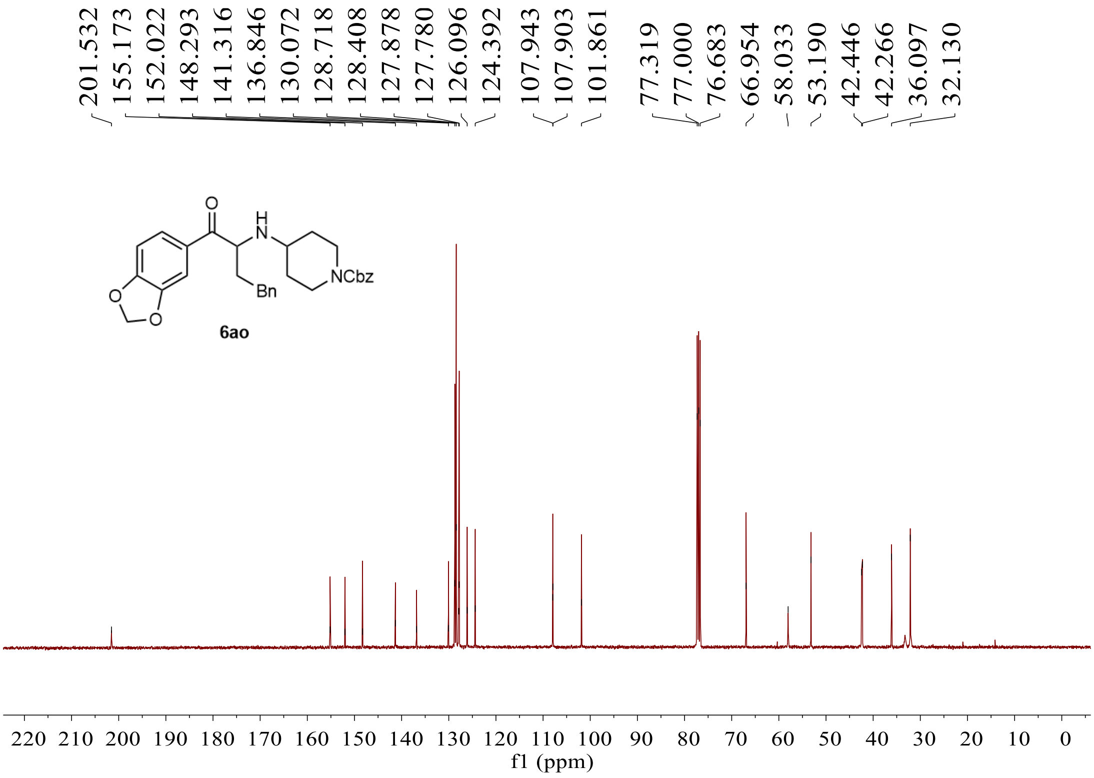
201.532 155.173 152.022 148.293 141.316 136.846 130.072 128.718 128.408 127.878 127.780 126.096 124.392 107.943 107.903 101.861 77.319 77.000 76.683 66.954 58.033 53.190 42.446 42.266 36.097 32.130
NCbz
Bn
6ao
220 210 200 190 180 170 160 150 140 130 120 110 100 90 80 70 60 50 40 30 20 10 0
f1 (ppm)

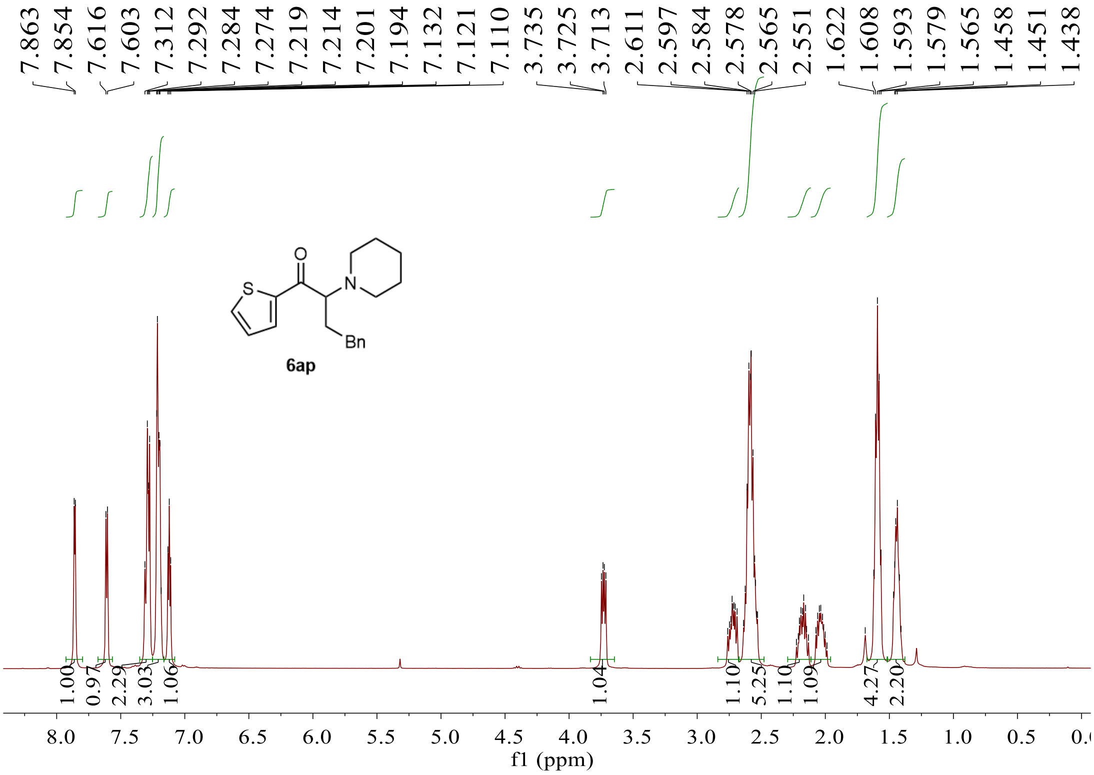
7.863 7.854 7.616 7.603 7.312 7.292 7.284 7.274 7.219 7.214 7.201 7.194 7.132 7.121 7.110 3.735 3.725 3.713 2.611 2.597 2.584 2.578 2.565 2.551 1.622 1.608 1.593 1.579 1.565 1.458 1.451 1.438
S
N
Bn
6ap
1.00 0.97 2.29 3.03 1.06 1.04 1.10 5.25 1.10 1.09 4.27 2.20
8.0 7.5 7.0 6.5 6.0 5.5 5.0 4.5 4.0 3.5 3.0 2.5 2.0 1.5 1.0 0.5
f1 (ppm)

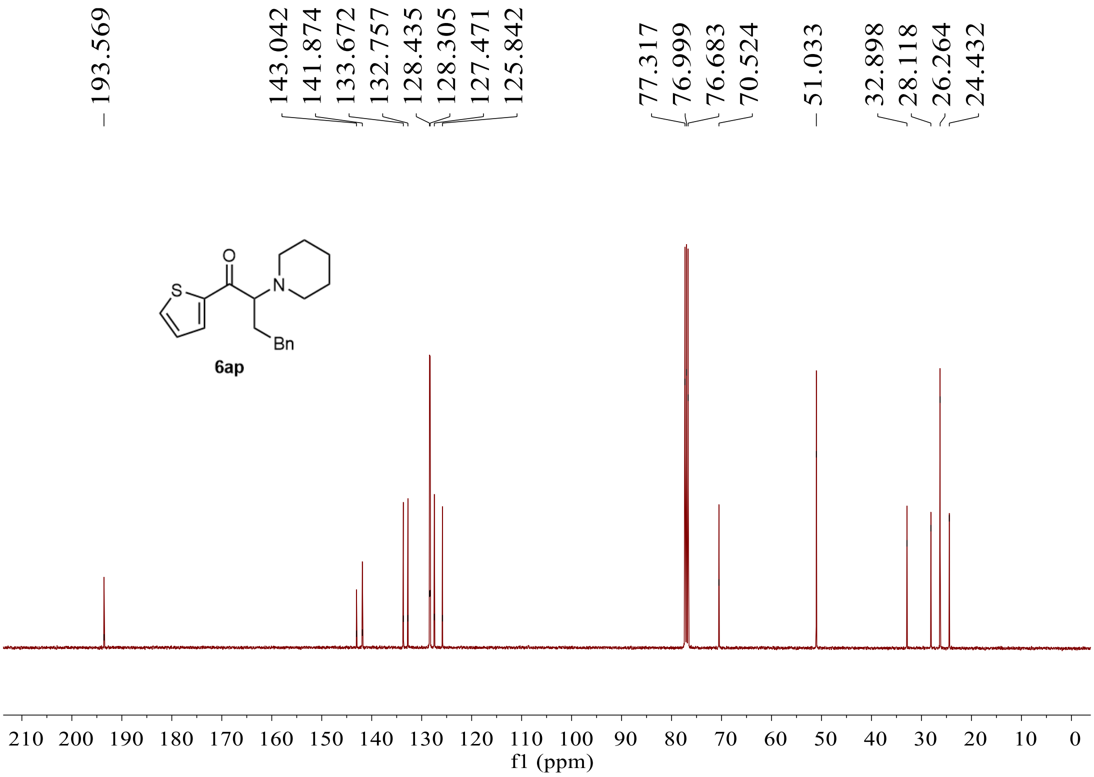
193.569
143.042
141.874
133.672
132.757
128.435
128.305
127.471
125.842
77.317
76.999
76.683
70.524
51.033
32.898
28.118
26.264
24.432
O
S
N
Bn
6ap
210 200 190 180 170 160 150 140 130 120 110 100 90 80 70 60 50 40 30 20 10 0
f1 (ppm)

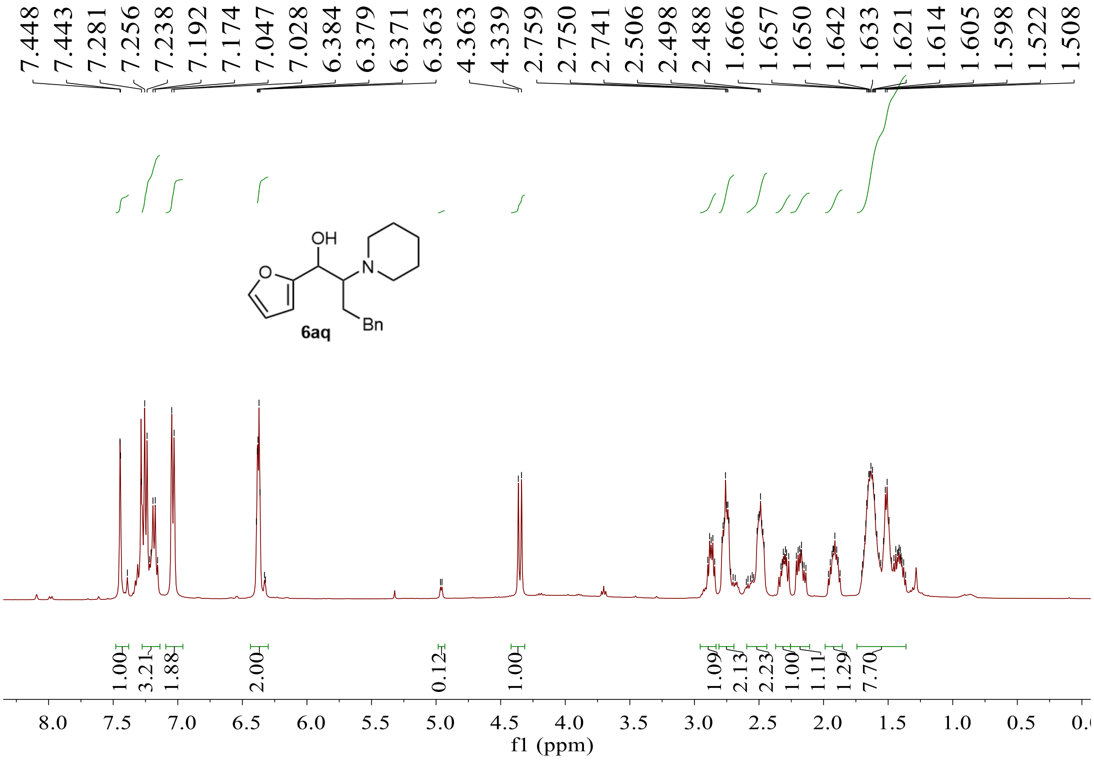
7.448
7.443
7.281
7.256
7.238
7.192
7.174
7.047
7.028
6.384
6.379
6.371
6.363
4.363
4.339
2.759
2.750
2.741
2.506
2.498
2.488
1.666
1.657
1.650
1.642
1.633
1.621
1.614
1.605
1.598
1.522
1.508
OH
O
N
Bn
6aq
1.00
3.21
1.88
2.00
0.12
1.00
1.09
2.13
2.23
1.00
1.11
1.29
7.70
8.0 7.5 7.0 6.5 6.0 5.5 5.0 4.5 4.0 3.5 3.0 2.5 2.0 1.5 1.0 0.5
f1 (ppm)

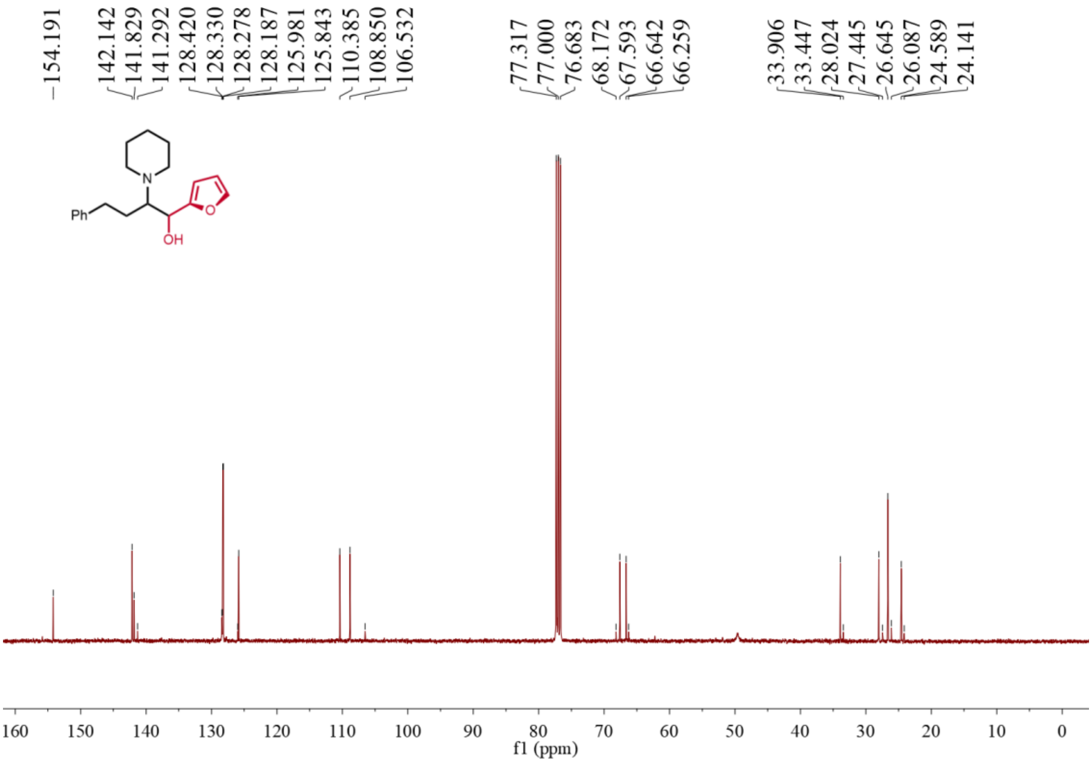
154.191
142.142
141.829
141.292
128.420
128.330
128.278
128.187
125.981
125.843
110.385
108.850
106.532
77.317
77.000
76.683
68.172
67.593
66.642
66.259
33.906
33.447
28.024
27.445
26.645
26.087
24.589
24.141
Ph
N
O
OH
160 150 140 130 120 110 100 90 80 70 60 50 40 30 20 10 0
f1 (ppm)

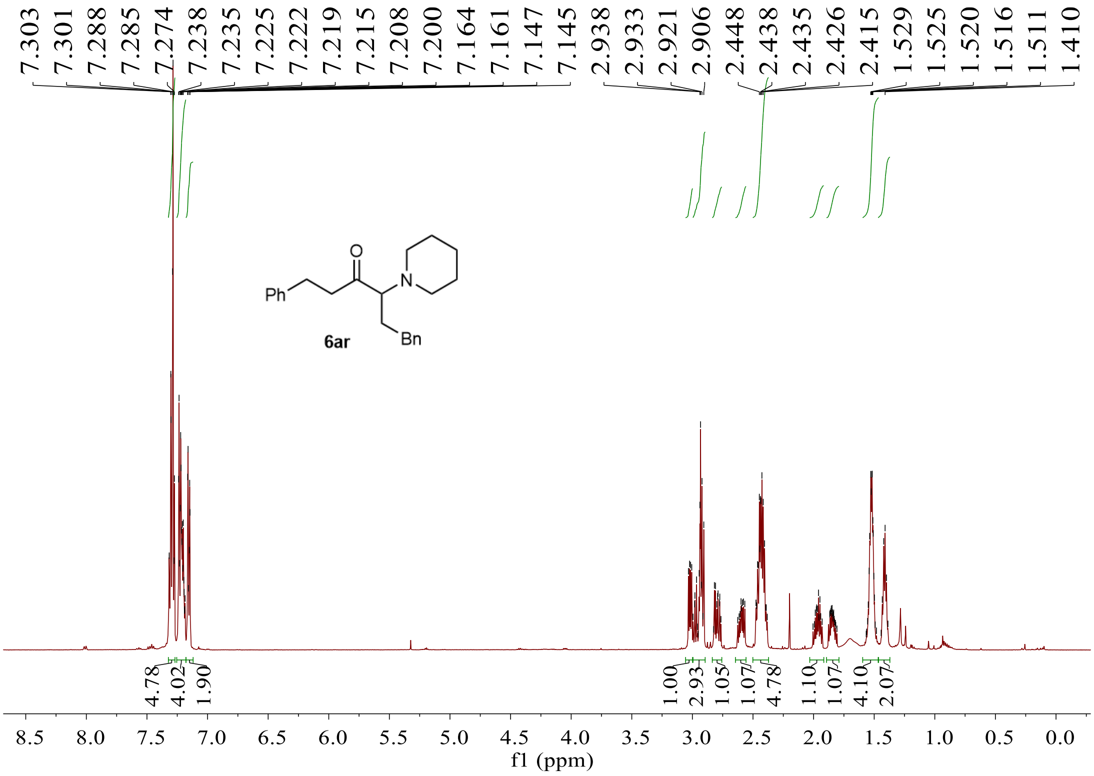
7.303
7.301
7.288
7.285
7.274
7.238
7.235
7.225
7.222
7.219
7.215
7.208
7.200
7.164
7.161
7.147
7.145
2.938
2.933
2.921
2.906
2.448
2.438
2.435
2.426
2.415
1.529
1.525
1.520
1.516
1.511
1.410
Ph
O
N
Bn
6ar
4.78
4.02
1.90
1.00
2.93
1.05
1.07
4.78
1.10
1.07
4.10
2.07
8.5 8.0 7.5 7.0 6.5 6.0 5.5 5.0 4.5 4.0 3.5 3.0 2.5 2.0 1.5 1.0 0.5 0.0
f1 (ppm)

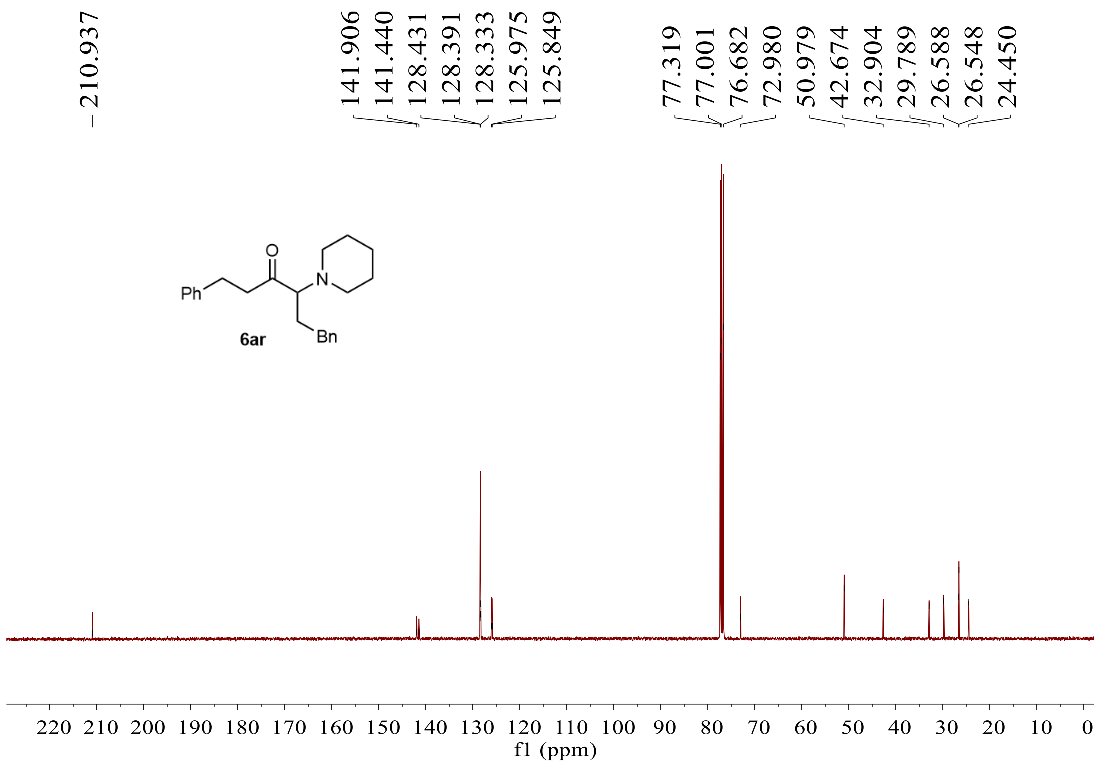
210.937
141.906
141.440
128.431
128.391
128.333
125.975
125.849
77.319
77.001
76.682
72.980
50.979
42.674
32.904
29.789
26.588
26.548
24.450
O
N
Ph
6ar
Bn
220 210 200 190 180 170 160 150 140 130 120 110 100 90 80 70 60 50 40 30 20 10 0
f1 (ppm)

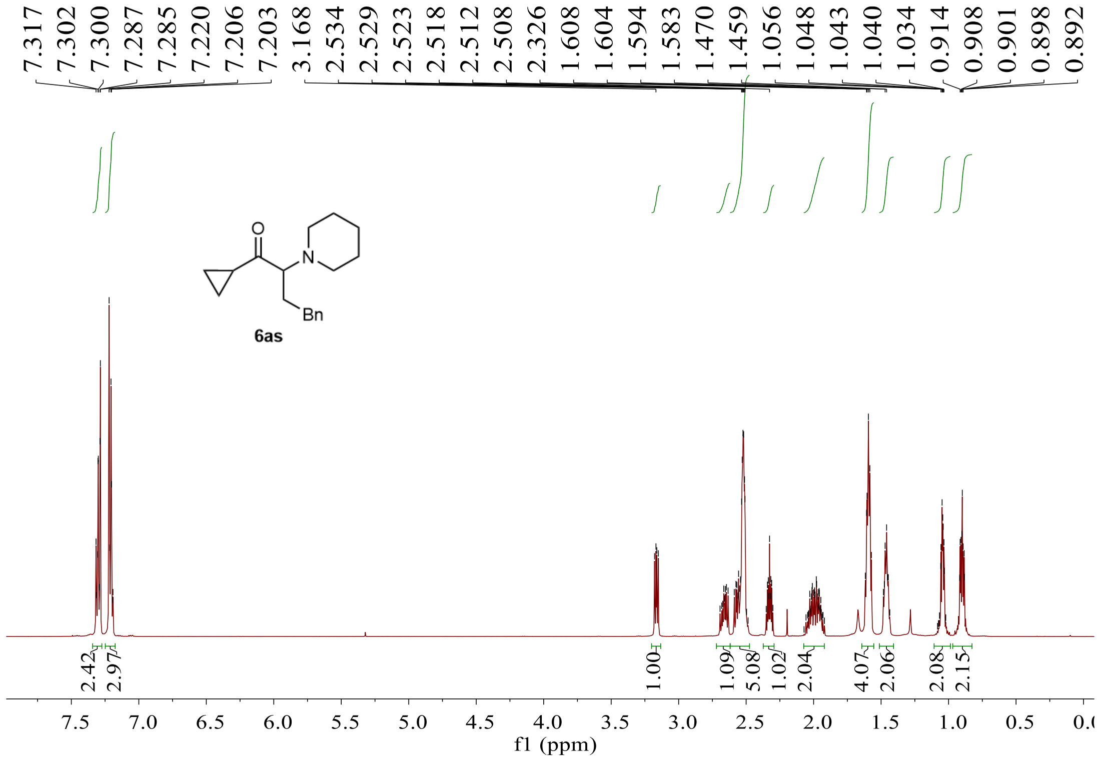
7.317
7.302
7.300
7.287
7.285
7.220
7.206
7.203
3.168
2.534
2.529
2.523
2.518
2.512
2.508
2.326
1.608
1.604
1.594
1.583
1.470
1.459
1.056
1.048
1.043
1.040
1.034
0.914
0.908
0.901
0.898
0.892
O
N
Bn
6as
2.42
2.97
1.00
1.09
5.08
1.02
2.04
4.07
2.06
2.08
2.15
7.5 7.0 6.5 6.0 5.5 5.0 4.5 4.0 3.5 3.0 2.5 2.0 1.5 1.0 0.5
f1 (ppm)

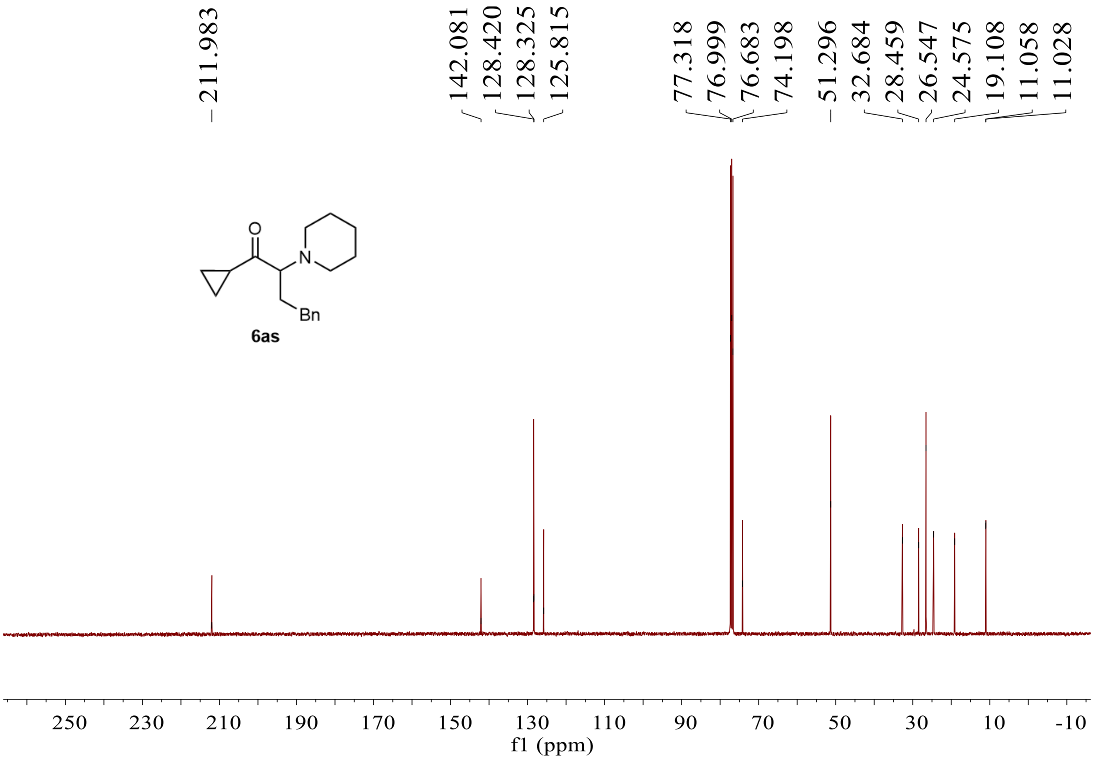
211.983
142.081
128.420
128.325
125.815
77.318
76.999
76.683
74.198
51.296
32.684
28.459
26.547
24.575
19.108
11.058
11.028
O
N
Bn
6as
250 230 210 190 170 150 130 110 90 70 50 30 10 -10
f1 (ppm)

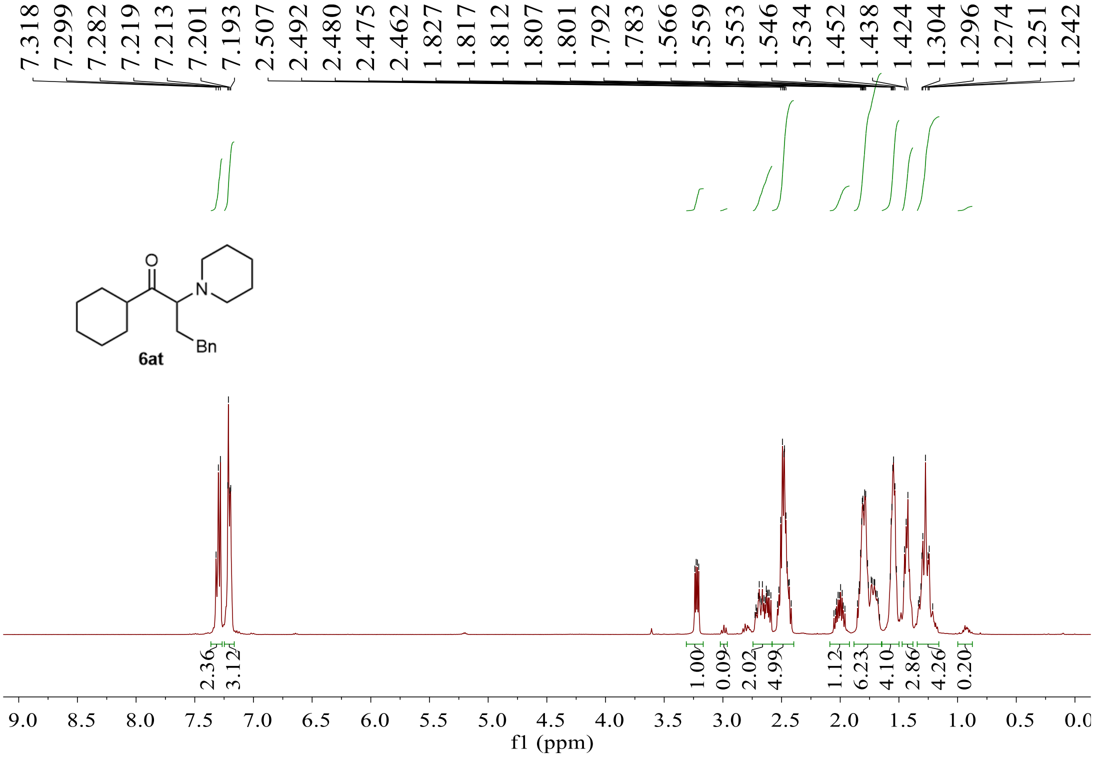
7.318
7.299
7.282
7.219
7.213
7.201
7.193
2.507
2.492
2.480
2.475
2.462
1.827
1.817
1.812
1.807
1.801
1.792
1.783
1.566
1.559
1.553
1.546
1.534
1.452
1.438
1.424
1.304
1.296
1.274
1.251
1.242
O
N
Bn
6at
2.36
3.12
1.00
0.09
2.02
4.99
1.12
6.23
4.10
2.86
4.26
0.20
9.0 8.5 8.0 7.5 7.0 6.5 6.0 5.5 5.0 4.5 4.0 3.5 3.0 2.5 2.0 1.5 1.0 0.5 0.0
f1 (ppm)

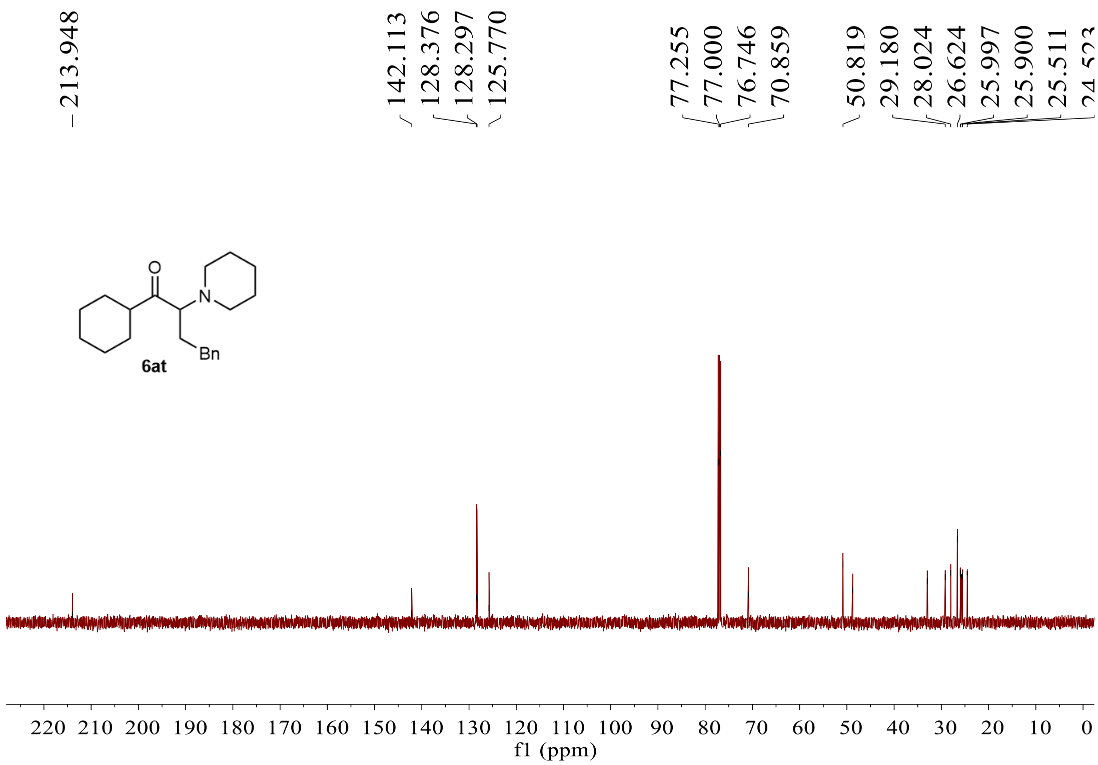
213.948
142.113
128.376
128.297
125.770
77.255
77.000
76.746
70.859
50.819
29.180
28.024
26.624
25.997
25.900
25.511
O
N
Bn
6at
220 210 200 190 180 170 160 150 140 130 120 110 100 90 80 70 60 50 40 30 20 10 0
f1 (ppm)

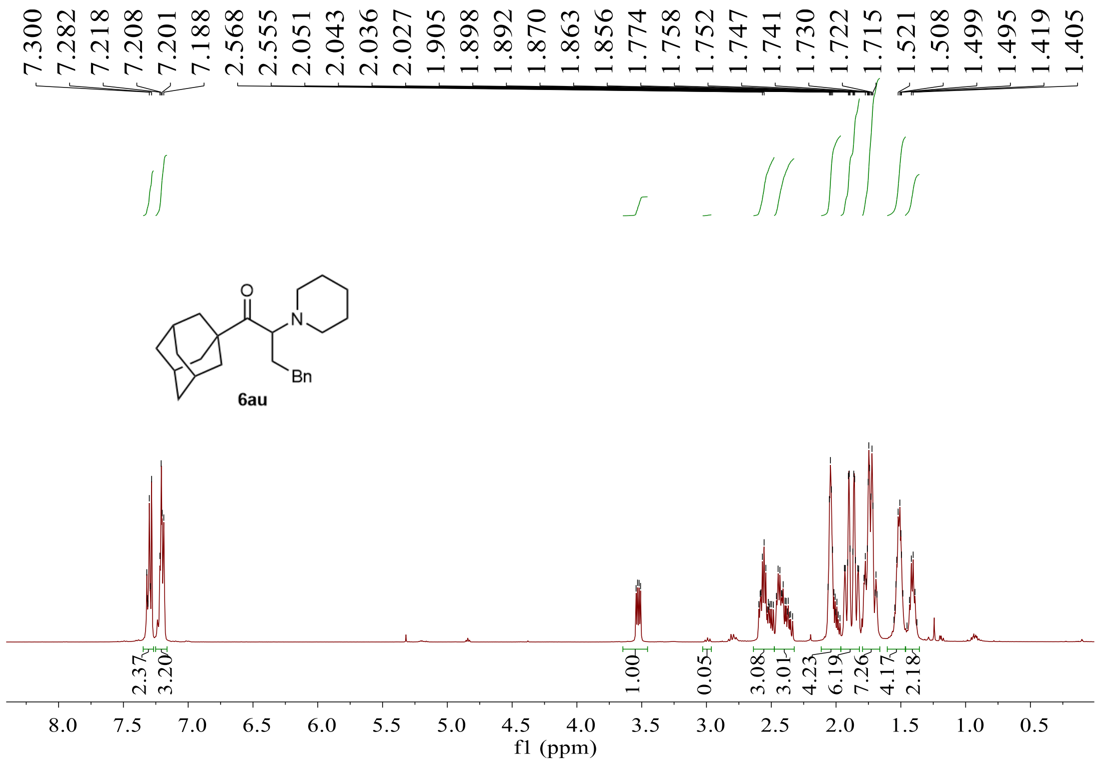
7.300
7.282
7.218
7.208
7.201
7.188
2.568
2.555
2.051
2.043
2.036
2.027
1.905
1.898
1.892
1.870
1.863
1.856
1.774
1.758
1.752
1.747
1.741
1.730
1.722
1.715
1.521
1.508
1.499
1.495
1.419
1.405
O
N
Bn
6au
2.37
3.20
1.00
0.05
3.08
3.01
4.23
6.19
7.26
4.17
2.18
8.0 7.5 7.0 6.5 6.0 5.5 5.0 4.5 4.0 3.5 3.0 2.5 2.0 1.5 1.0 0.5
f1 (ppm)

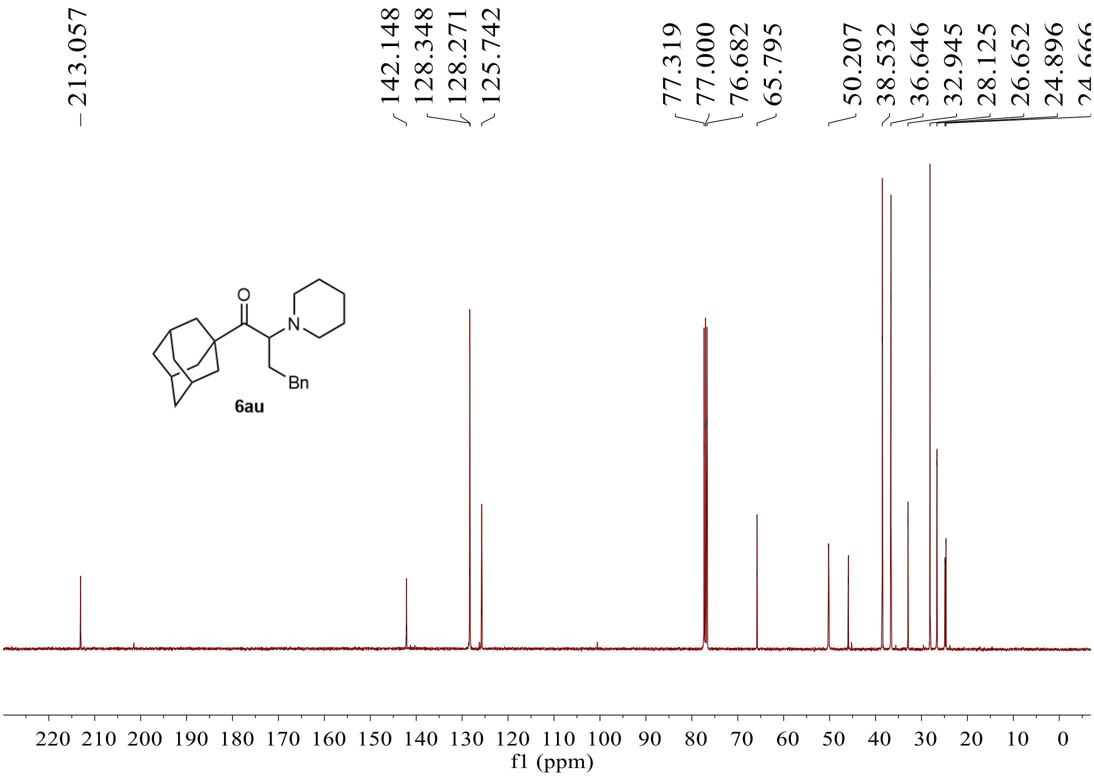
213.057
142.148
128.348
128.271
125.742
77.319
77.000
76.682
65.795
50.207
38.532
36.646
32.945
28.125
26.652
24.896
O
N
Bn
6au
220 210 200 190 180 170 160 150 140 130 120 110 100 90 80 70 60 50 40 30 20 10 0
f1 (ppm)

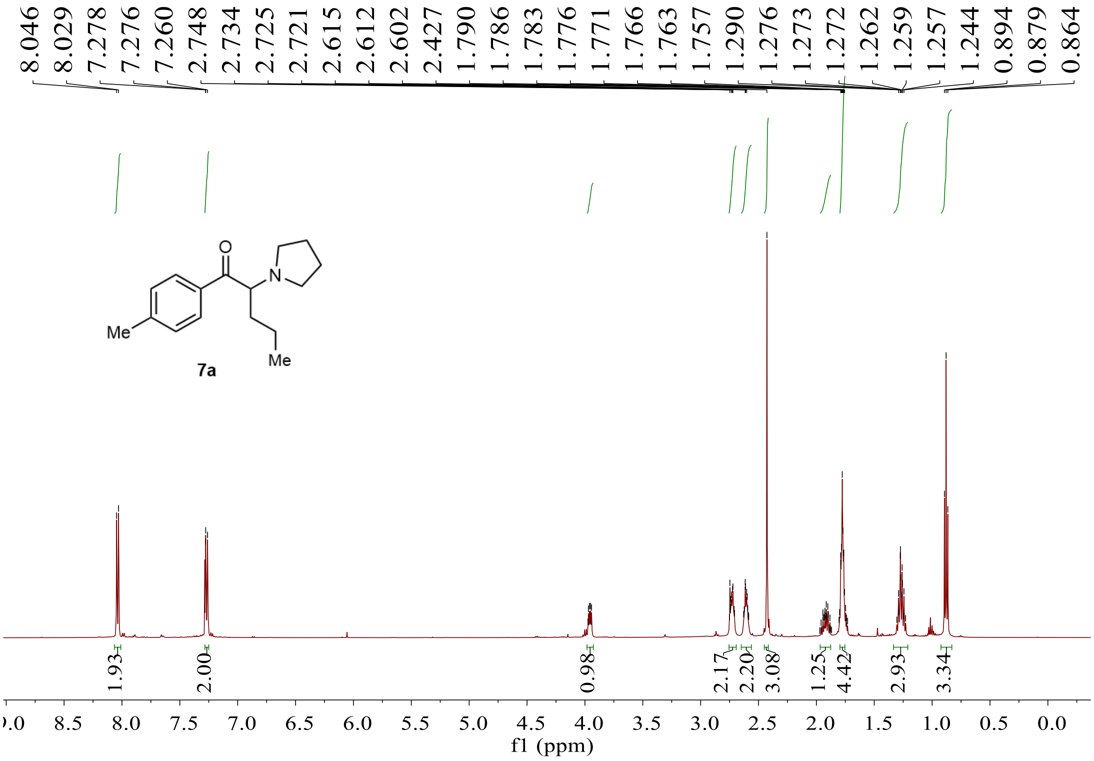
8.046
8.029
7.278
7.276
7.260
2.748
2.734
2.725
2.721
2.615
2.612
2.602
2.427
1.790
1.786
1.783
1.776
1.771
1.766
1.763
1.757
1.290
1.276
1.273
1.272
1.262
1.259
1.257
1.244
0.894
0.879
0.864
O
N
Me
Me
7a
1.93
2.00
0.98
2.17
2.20
3.08
1.25
4.42
2.93
3.34
8.5 8.0 7.5 7.0 6.5 6.0 5.5 5.0 4.5 4.0 3.5 3.0 2.5 2.0 1.5 1.0 0.5 0.0
f1 (ppm)

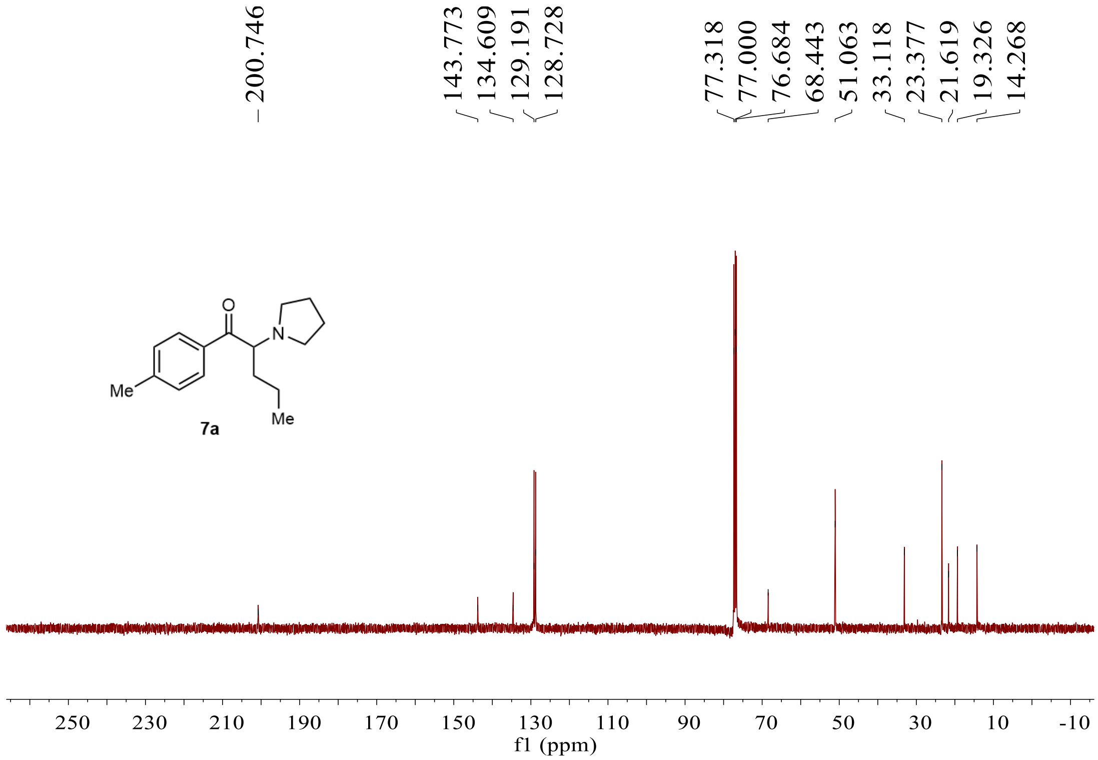

200.746
143.773
134.609
129.191
128.728
77.318
77.000
76.684
68.443
51.063
33.118
23.377
21.619
19.326
14.268
O
N
Me
Me
7a
250 230 210 190 170 150 130 110 90 70 50 30 10 -10
f1 (ppm)

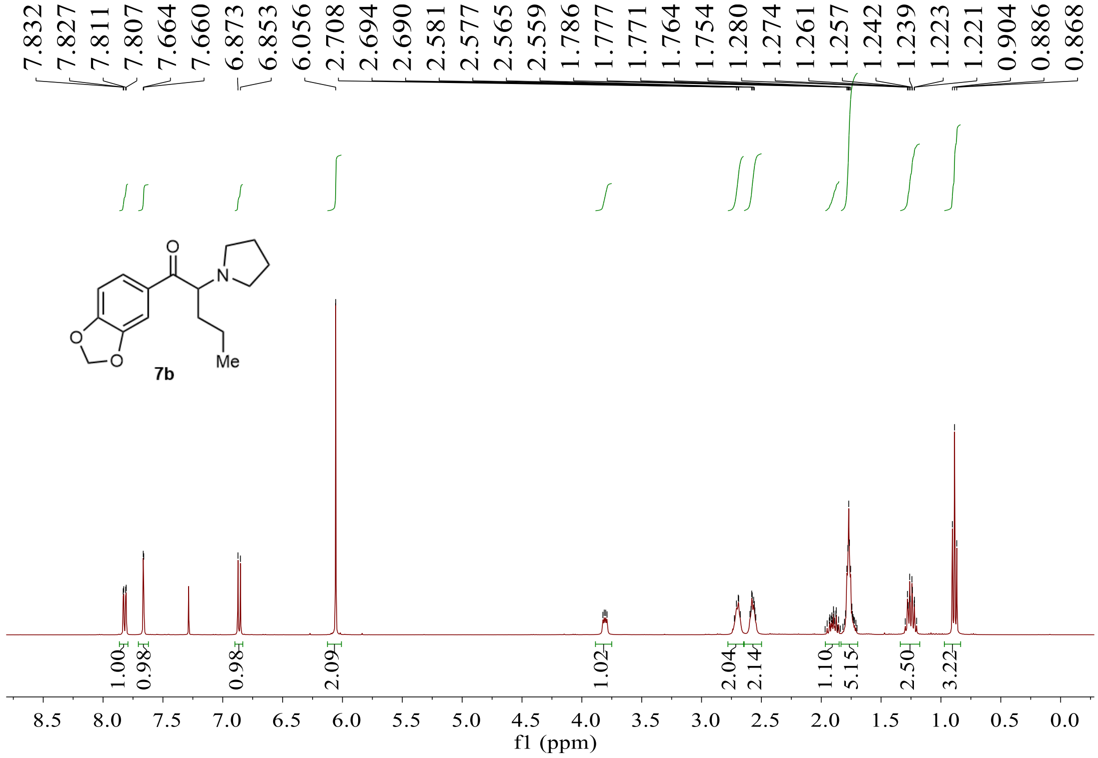

7.832
7.827
7.811
7.807
7.664
7.660
6.873
6.853
6.056
2.708
2.694
2.690
2.581
2.577
2.565
2.559
1.786
1.777
1.771
1.764
1.754
1.280
1.274
1.261
1.257
1.242
1.239
1.223
1.221
0.904
0.886
0.868
O
N
O
O
7b
Me
1.00
0.98
0.98
2.09
1.02
2.04
2.14
1.10
5.15
2.50
3.22
8.5 8.0 7.5 7.0 6.5 6.0 5.5 5.0 4.5 4.0 3.5 3.0 2.5 2.0 1.5 1.0 0.5 0.0
f1 (ppm)

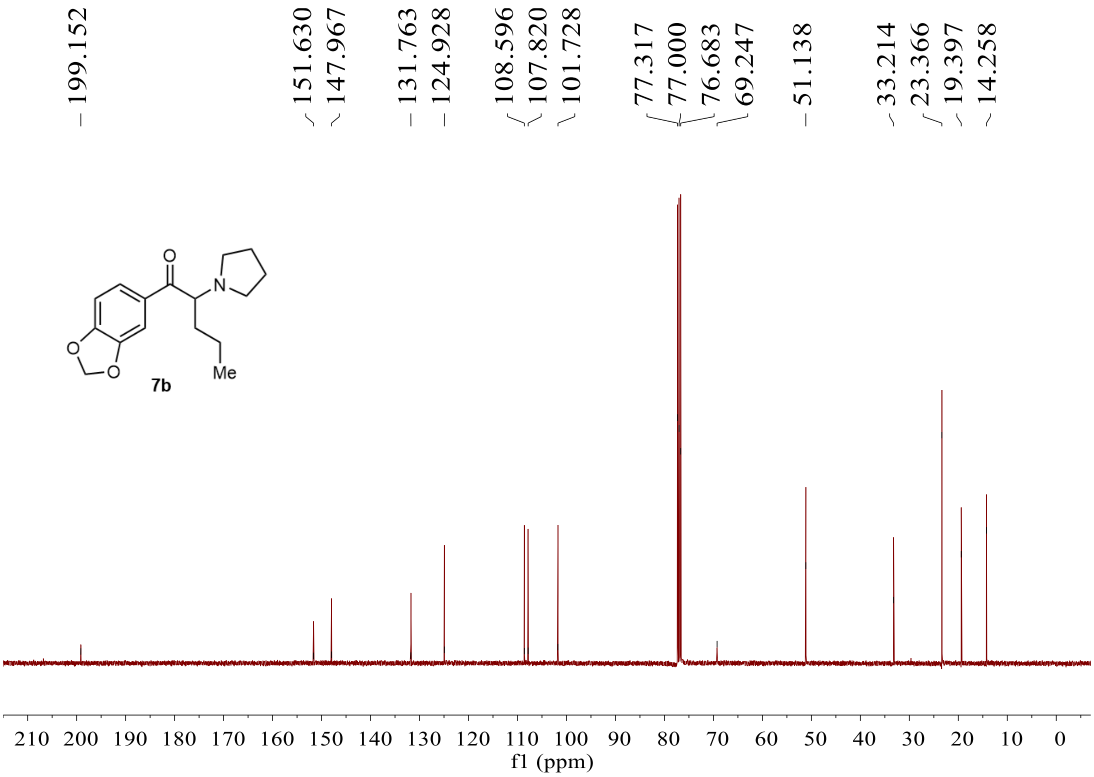

199.152
151.630
147.967
131.763
124.928
108.596
107.820
101.728
77.317
77.000
76.683
69.247
51.138
33.214
23.366
19.397
14.258
O
N
O
O
Me
7b
210 200 190 180 170 160 150 140 130 120 110 100 90 80 70 60 50 40 30 20 10 0
f1 (ppm)

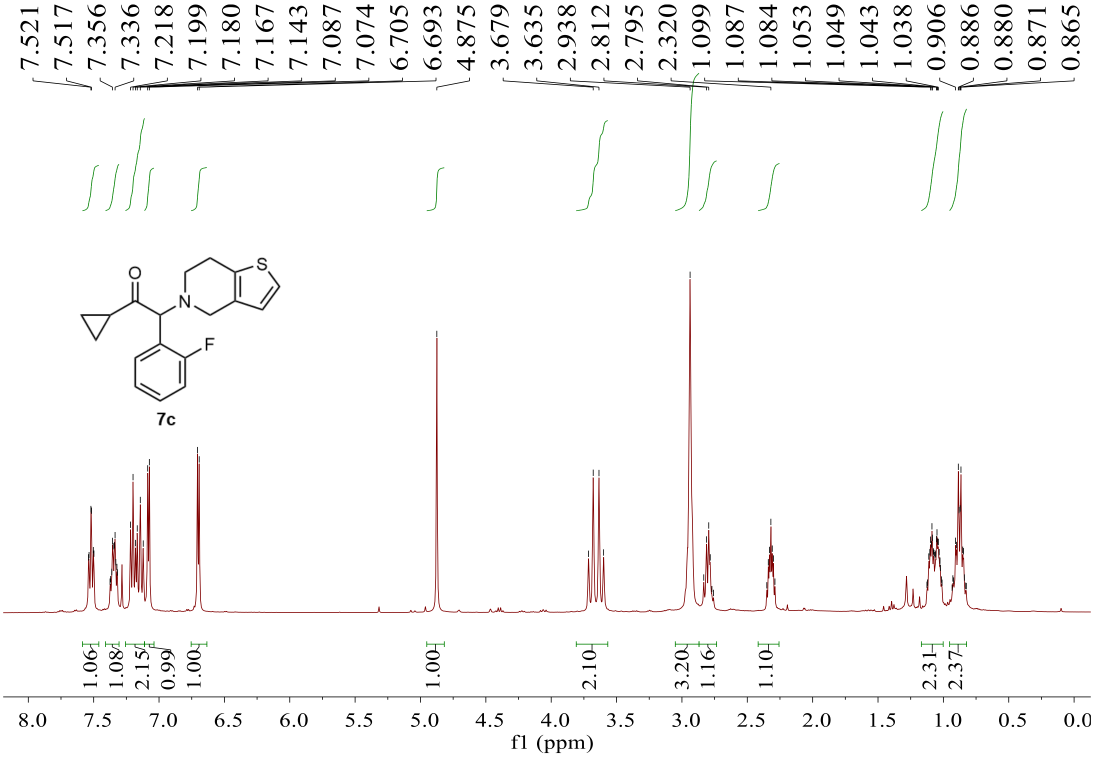

7.521
7.517
7.356
7.336
7.218
7.199
7.180
7.167
7.143
7.087
7.074
6.705
6.693
4.875
3.679
3.635
2.938
2.812
2.795
2.320
1.099
1.087
1.084
1.053
1.049
1.043
1.038
0.906
0.886
0.880
0.871
0.865
O
N
S
F
7c
1.06
1.08
2.15
0.99
1.00
1.00
2.10
3.20
1.16
1.10
2.31
2.37
8.0 7.5 7.0 6.5 6.0 5.5 5.0 4.5 4.0 3.5 3.0 2.5 2.0 1.5 1.0 0.5 0.0
f1 (ppm)

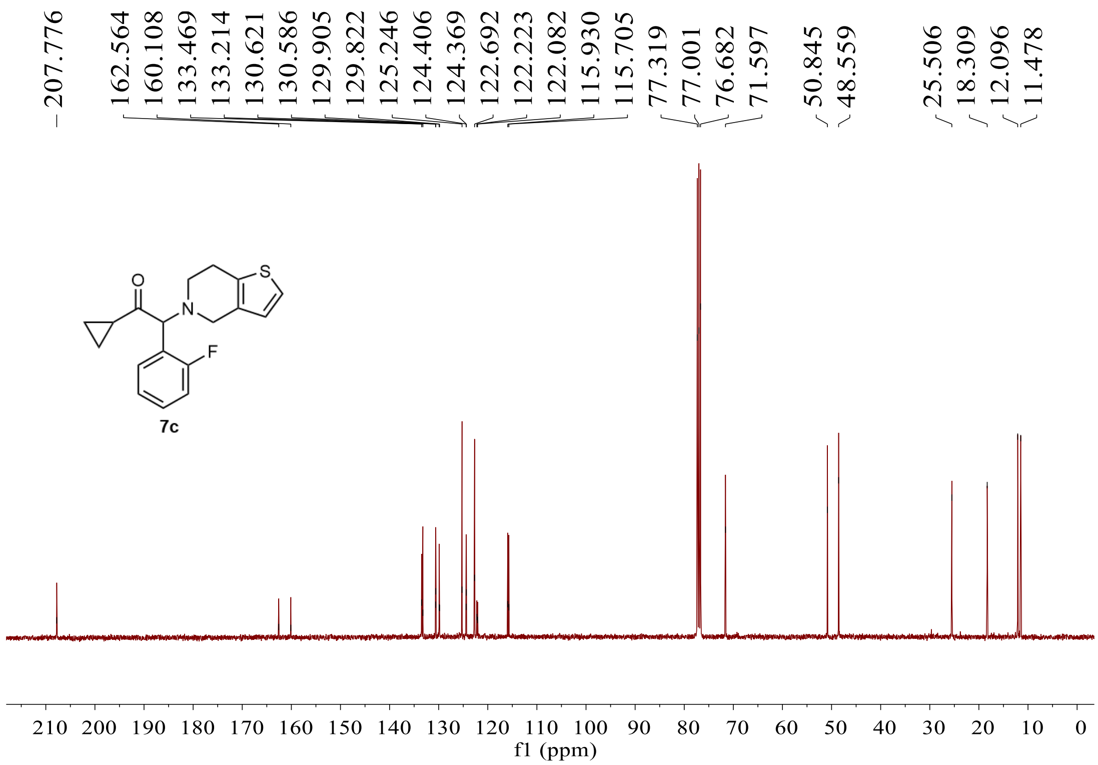
207.776
162.564
160.108
133.469
133.214
130.621
130.586
129.905
129.822
125.246
124.406
124.369
122.692
122.223
122.082
115.930
115.705
77.319
77.001
76.682
71.597
50.845
48.559
25.506
18.309
12.096
11.478
O
N
S
F
7c
210 200 190 180 170 160 150 140 130 120 110 100 90 80 70 60 50 40 30 20 10 0
f1 (ppm)

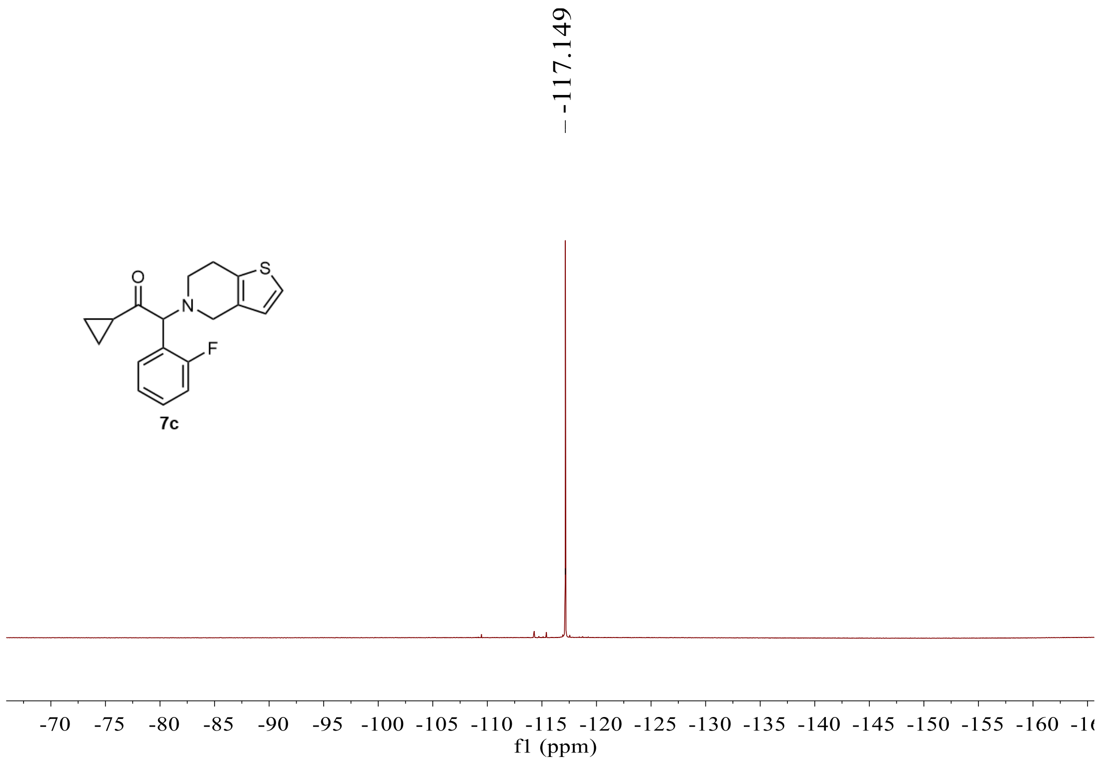
-117.149
O
N
S
F
7c
-70 -75 -80 -85 -90 -95 -100 -105 -110 -115 -120 -125 -130 -135 -140 -145 -150 -155 -160
f1 (ppm)

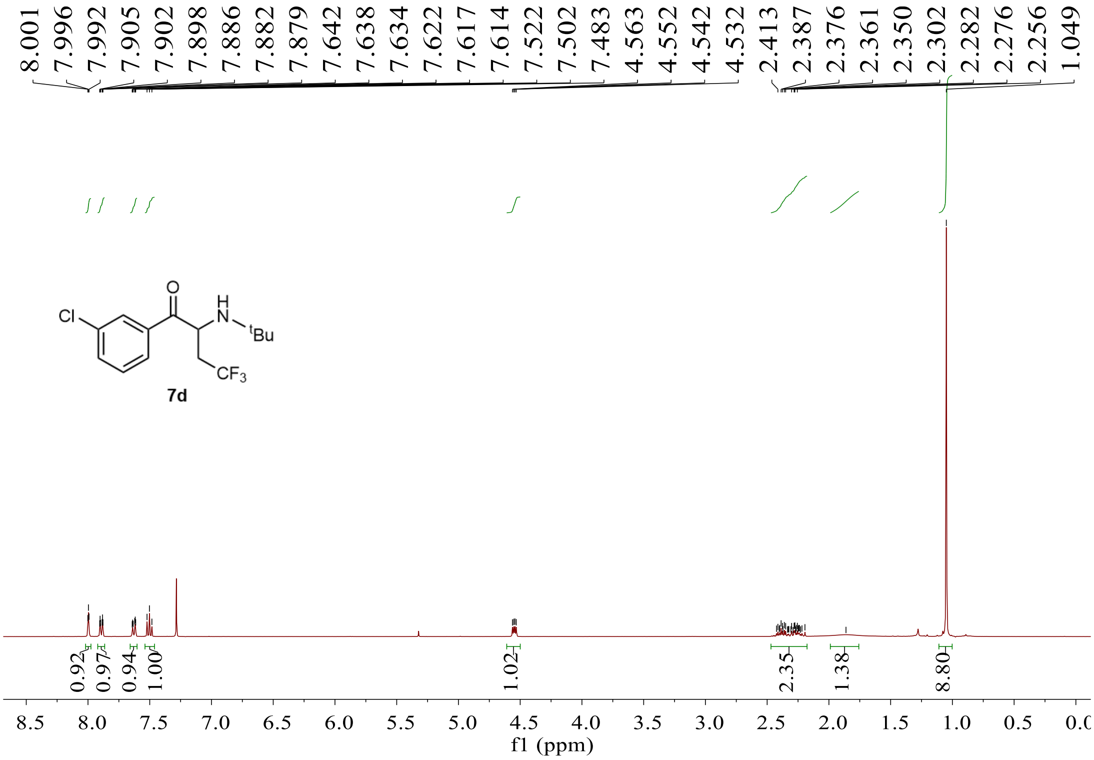
8.001
7.996
7.992
7.905
7.902
7.898
7.886
7.882
7.879
7.642
7.638
7.634
7.622
7.617
7.614
7.522
7.502
7.483
4.563
4.552
4.542
4.532
2.413
2.387
2.376
2.361
2.350
2.302
2.282
2.276
2.256
1.049
Cl
O
H
N
tBu
CF3
7d
0.92
0.97
0.94
1.00
1.02
2.35
1.38
8.80
8.5 8.0 7.5 7.0 6.5 6.0 5.5 5.0 4.5 4.0 3.5 3.0 2.5 2.0 1.5 1.0 0.5 0.0
f1 (ppm)

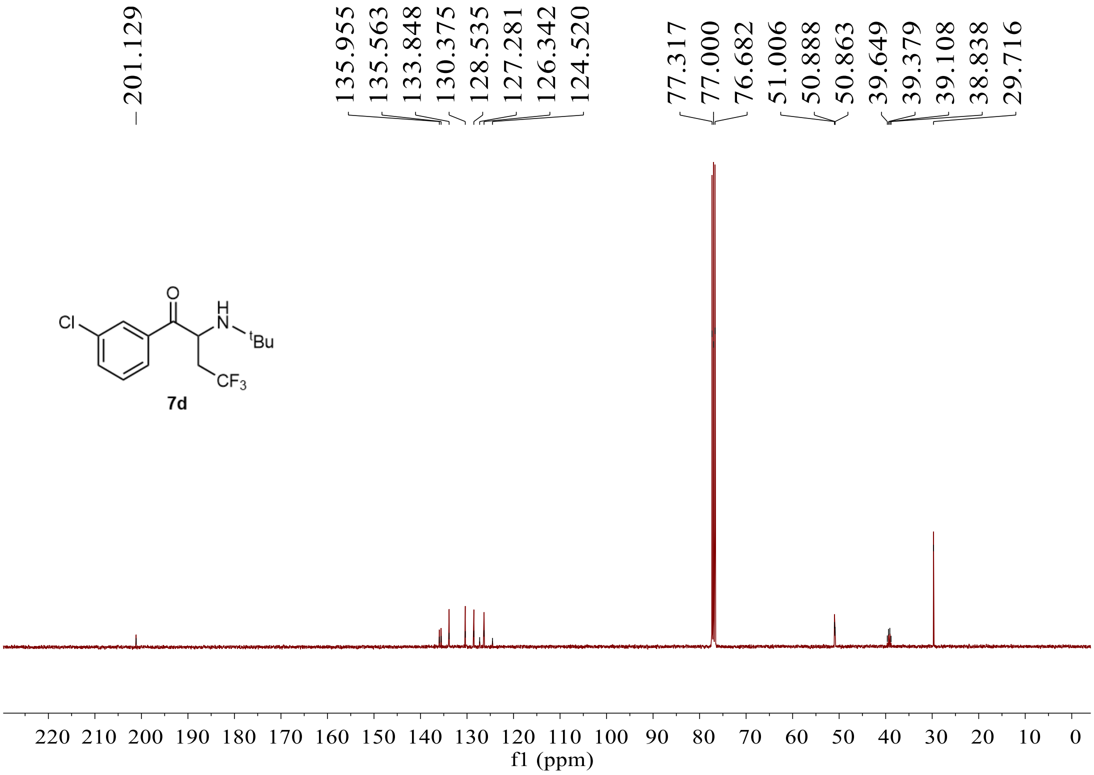
201.129
135.955
135.563
133.848
130.375
128.535
127.281
126.342
124.520
77.317
77.000
76.682
51.006
50.888
50.863
39.649
39.379
39.108
38.838
29.716
Cl
O
H
N
tBu
CF3
7d
220 210 200 190 180 170 160 150 140 130 120 110 100 90 80 70 60 50 40 30 20 10 0
f1 (ppm)

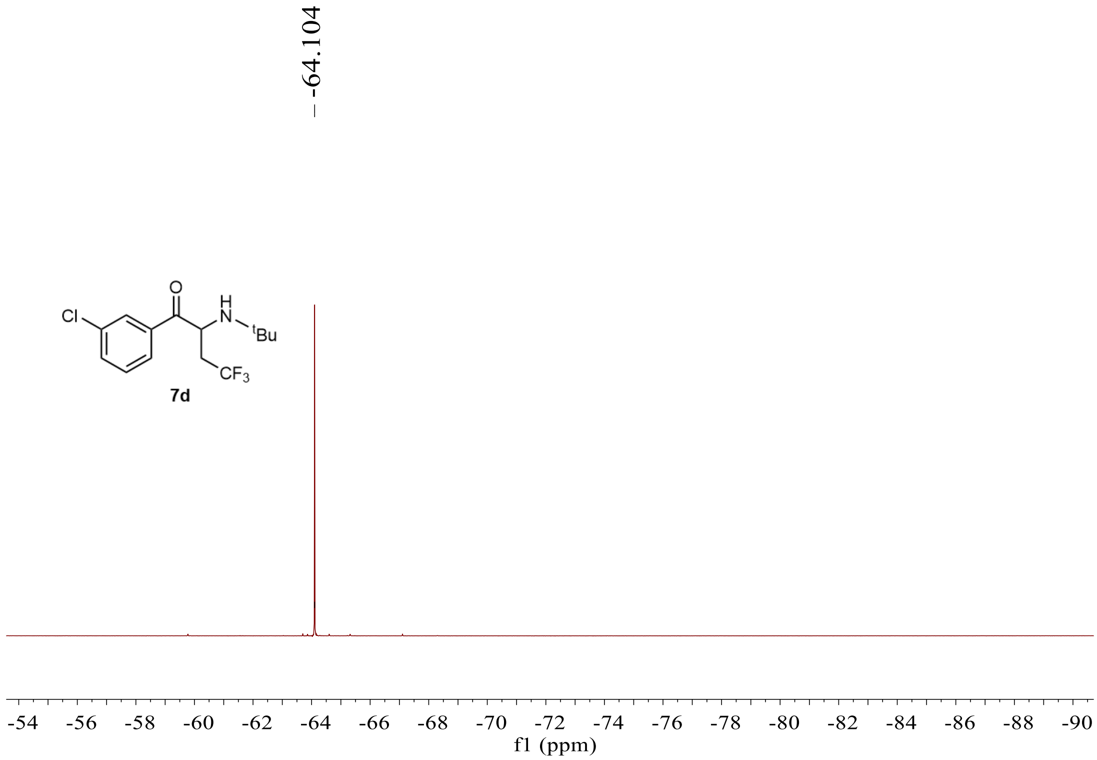
-64.104
Cl
O
H
N
tBu
CF3
7d
-54 -56 -58 -60 -62 -64 -66 -68 -70 -72 -74 -76 -78 -80 -82 -84 -86 -88 -90
f1 (ppm)

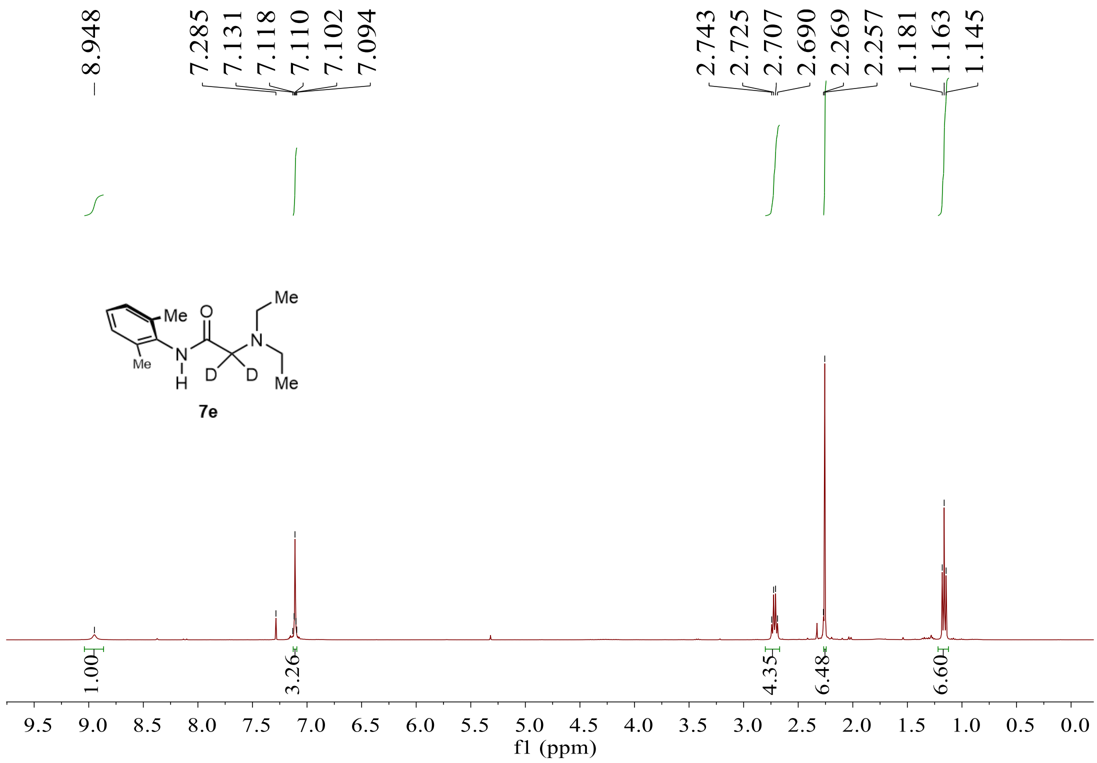
8.948
7.285
7.131
7.118
7.110
7.102
7.094
2.743
2.725
2.707
2.690
2.269
2.257
1.181
1.163
1.145
Me
O
Me
N
Me
N
H
D
D
Me
7e
1.00
3.26
4.35
6.48
6.60
9.5 9.0 8.5 8.0 7.5 7.0 6.5 6.0 5.5 5.0 4.5 4.0 3.5 3.0 2.5 2.0 1.5 1.0 0.5 0.0
f1 (ppm)

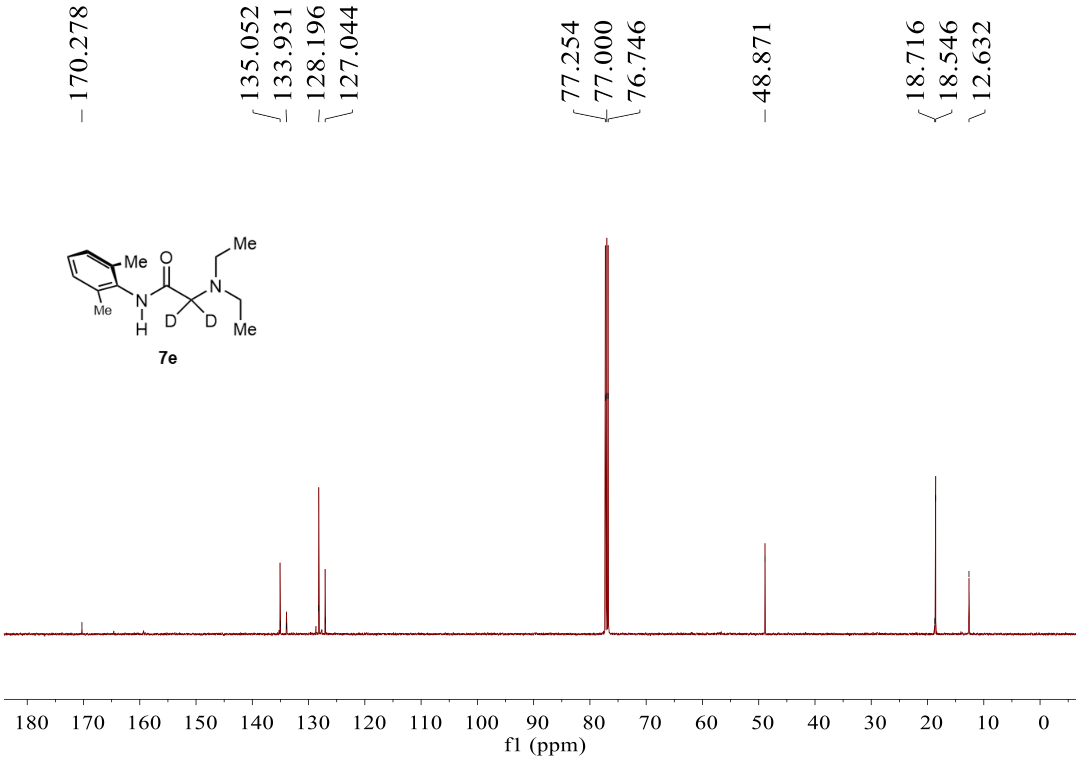
170.278
135.052
133.931
128.196
127.044
77.254
77.000
76.746
48.871
18.716
18.546
12.632
Me
O
N
H
D
D
Me
Me
Me
N
7e
180 170 160 150 140 130 120 110 100 90 80 70 60 50 40 30 20 10 0
f1 (ppm)

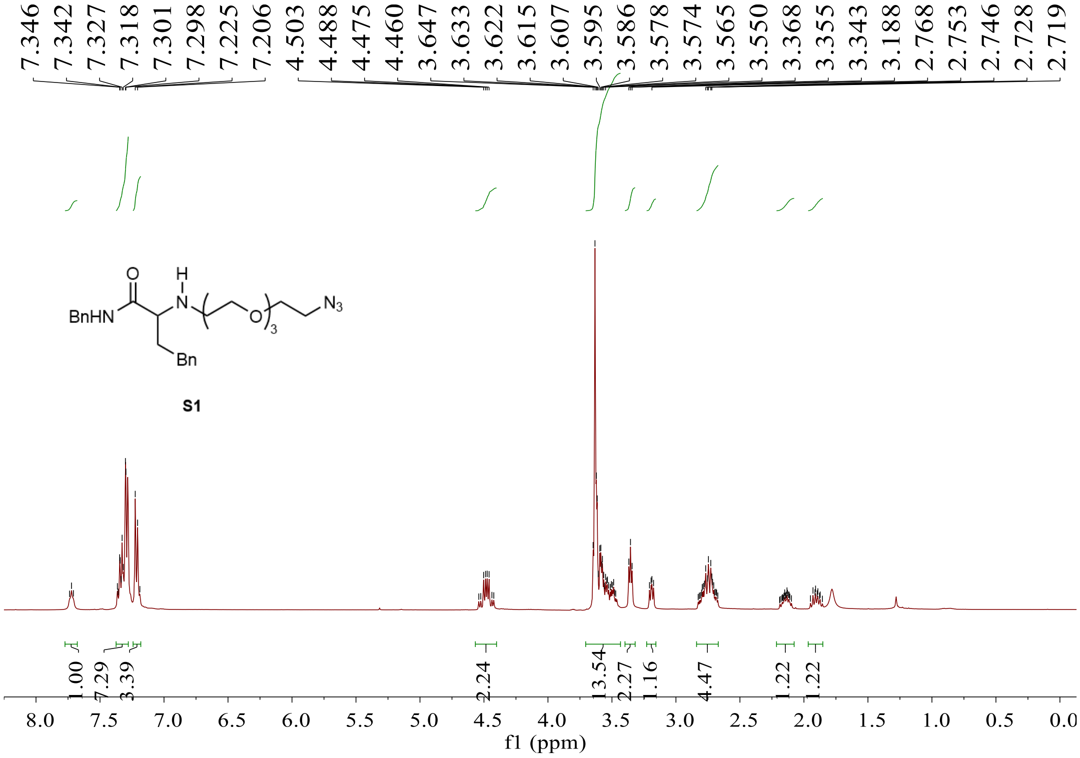
7.346
7.342
7.327
7.318
7.301
7.298
7.225
7.206
4.503
4.488
4.475
4.460
3.647
3.633
3.622
3.615
3.607
3.595
3.586
3.578
3.574
3.565
3.550
3.368
3.355
3.343
3.188
2.768
2.753
2.746
2.728
2.719
O
H
N
BnHN
O
3
N3
Bn
S1
1.00
7.29
3.39
2.24
13.54
2.27
1.16
4.47
1.22
1.22
8.0 7.5 7.0 6.5 6.0 5.5 5.0 4.5 4.0 3.5 3.0 2.5 2.0 1.5 1.0 0.5 0.0
f1 (ppm)

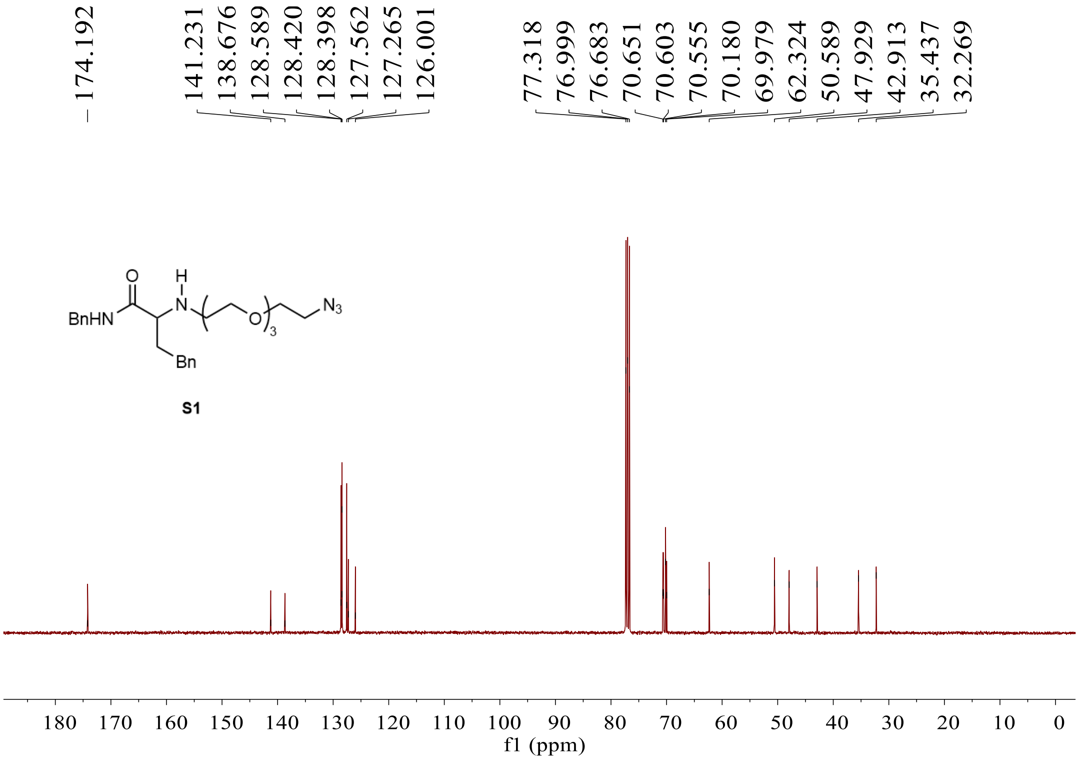
174.192
141.231
138.676
128.589
128.420
128.398
127.562
127.265
126.001
77.318
76.999
76.683
70.651
70.603
70.555
70.180
69.979
62.324
50.589
47.929
42.913
35.437
32.269
BnHN
H
N
O
O
3
N3
Bn
S1
180 170 160 150 140 130 120 110 100 90 80 70 60 50 40 30 20 10 0
f1 (ppm)

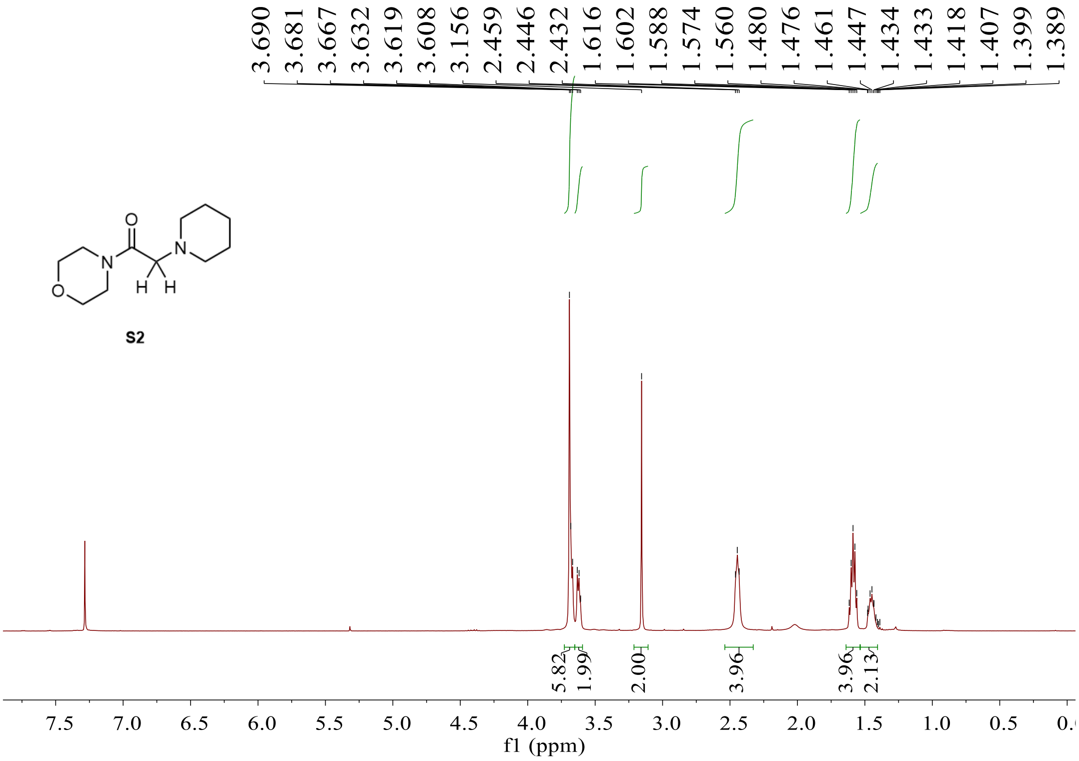
3.690
3.681
3.667
3.632
3.619
3.608
3.156
2.459
2.446
2.432
1.616
1.602
1.588
1.574
1.560
1.480
1.476
1.461
1.447
1.434
1.433
1.418
1.407
1.399
1.389
O
N
O
N
H H
S2
5.82
1.99
2.00
3.96
3.96
2.13
7.5 7.0 6.5 6.0 5.5 5.0 4.5 4.0 3.5 3.0 2.5 2.0 1.5 1.0 0.5 0.
f1 (ppm)

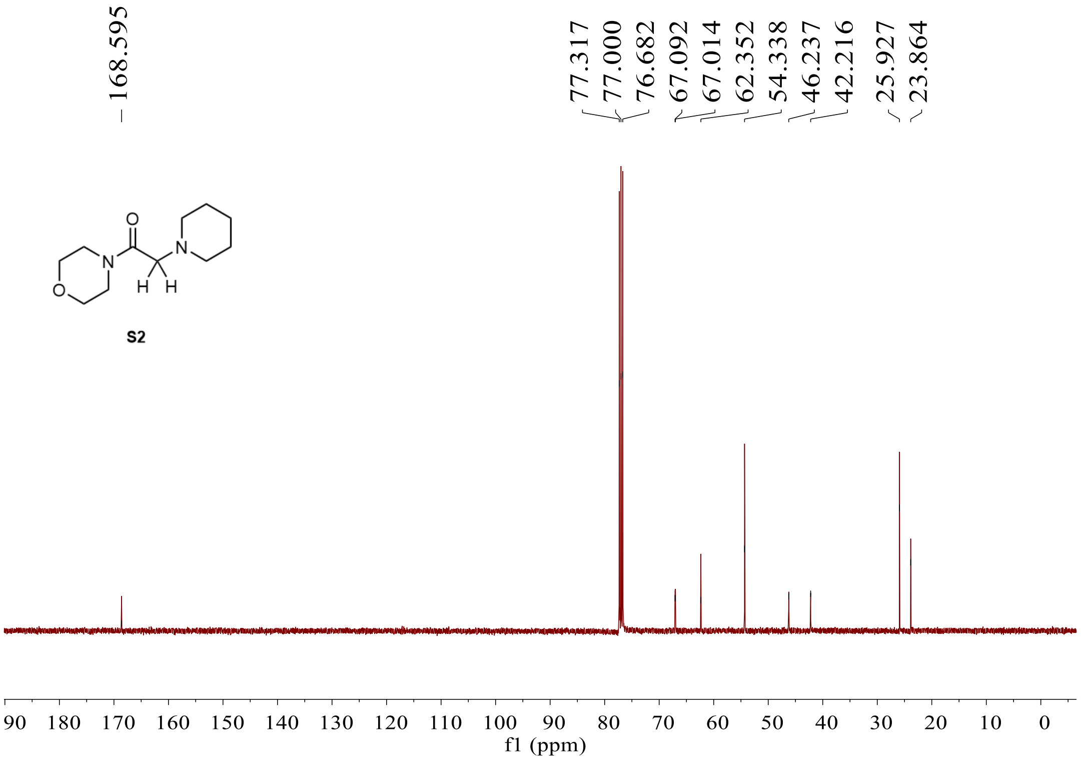
168.595
77.317
77.000
76.682
67.092
67.014
62.352
54.338
46.237
42.216
25.927
23.864
O
N
N
O
H H
S2
90 180 170 160 150 140 130 120 110 100 90 80 70 60 50 40 30 20 10 0
f1 (ppm)

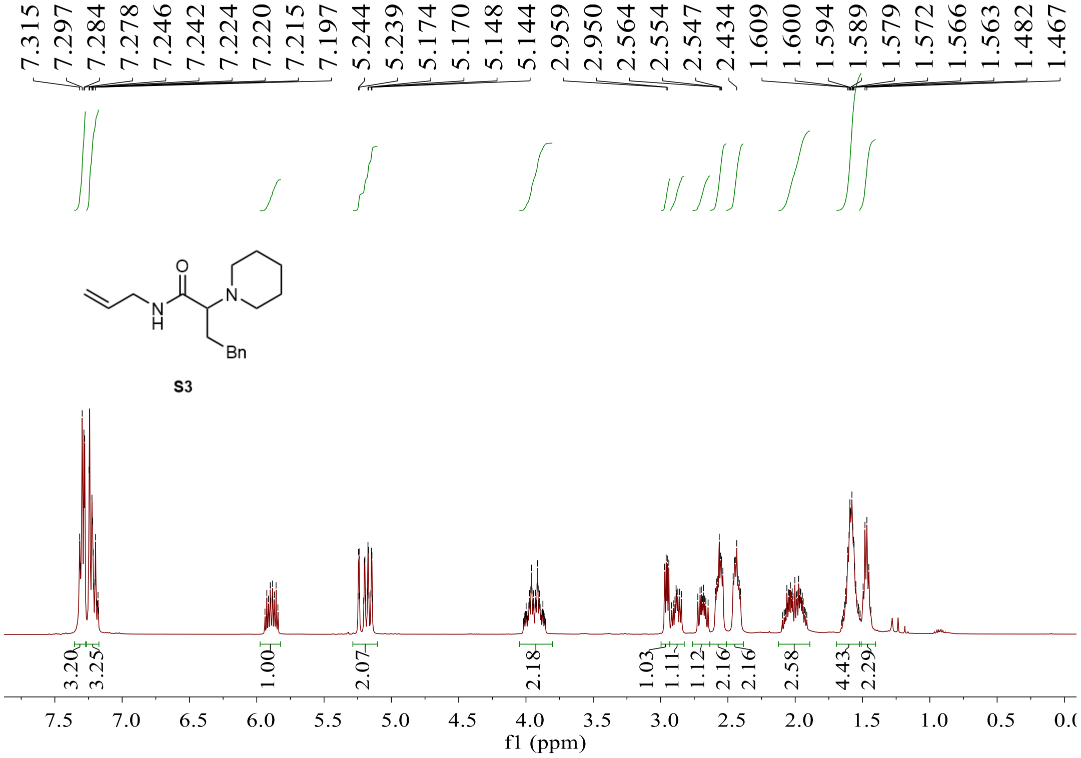
7.315
7.297
7.284
7.278
7.246
7.242
7.224
7.220
7.215
7.197
5.244
5.239
5.174
5.170
5.148
5.144
2.959
2.950
2.564
2.554
2.547
2.434
1.609
1.600
1.594
1.589
1.579
1.572
1.566
1.563
1.482
1.467
O
N
H
N
Bn
S3
3.20
3.25
1.00
2.07
2.18
1.03
1.11
1.12
2.16
2.16
2.58
4.43
2.29
7.5 7.0 6.5 6.0 5.5 5.0 4.5 4.0 3.5 3.0 2.5 2.0 1.5 1.0 0.5 0.0
f1 (ppm)

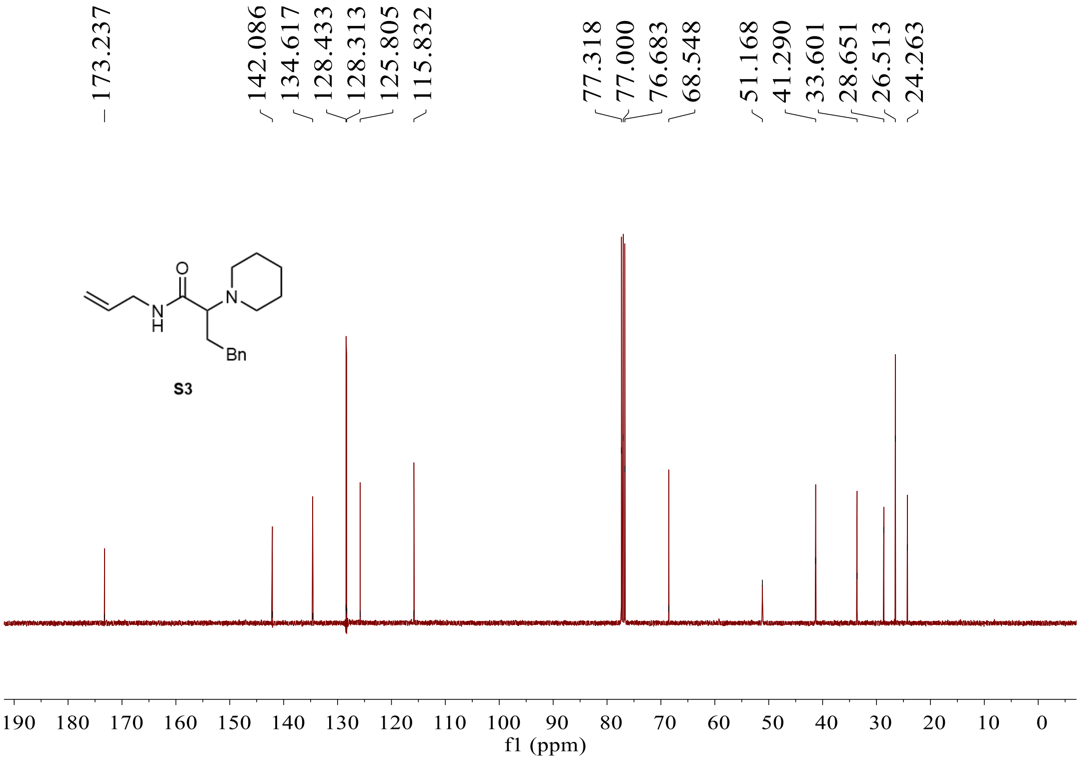
173.237
142.086
134.617
128.433
128.313
125.805
115.832
77.318
77.000
76.683
68.548
51.168
41.290
33.601
28.651
26.513
24.263
O
N
H
N
Bn
S3
190 180 170 160 150 140 130 120 110 100 90 80 70 60 50 40 30 20 10 0
f1 (ppm)

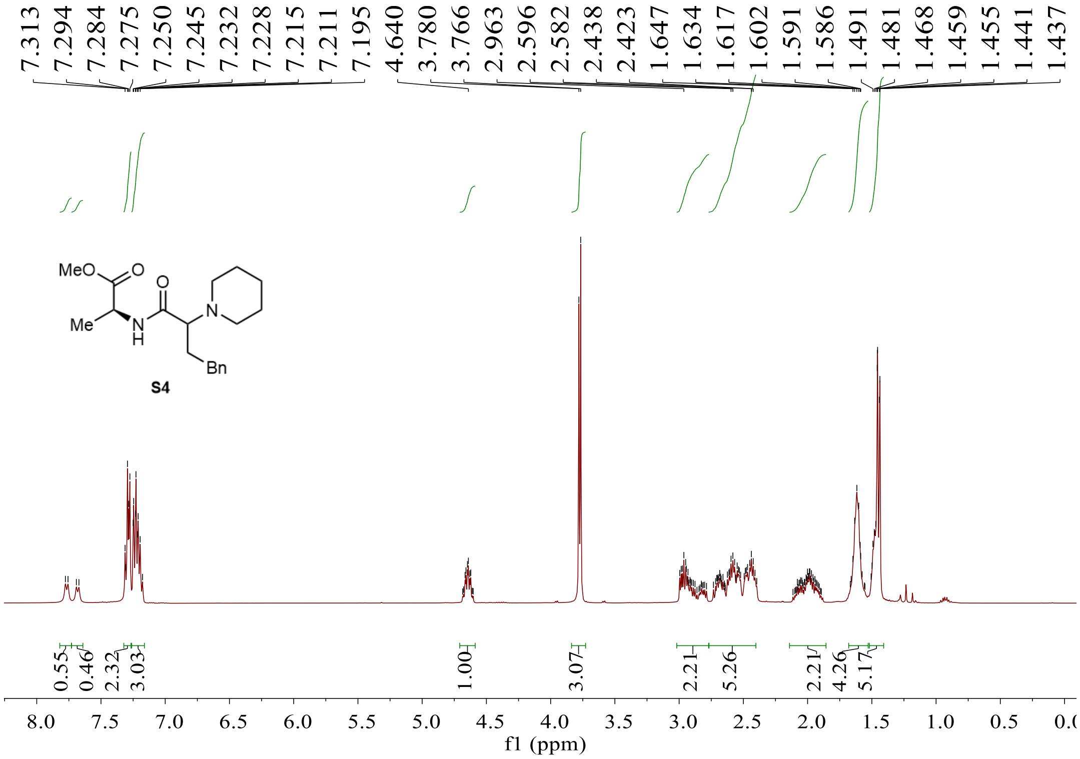
7.313
7.294
7.284
7.275
7.250
7.245
7.232
7.228
7.215
7.211
7.195
4.640
3.780
3.766
2.963
2.596
2.582
2.438
2.423
1.647
1.634
1.617
1.602
1.591
1.586
1.491
1.481
1.468
1.459
1.455
1.441
1.437
MeO
O
O
Me
N
H
N
Bn
S4
0.55
0.46
2.32
3.03
1.00
3.07
2.21
5.26
2.21
4.26
5.17
8.0 7.5 7.0 6.5 6.0 5.5 5.0 4.5 4.0 3.5 3.0 2.5 2.0 1.5 1.0 0.5
f1 (ppm)

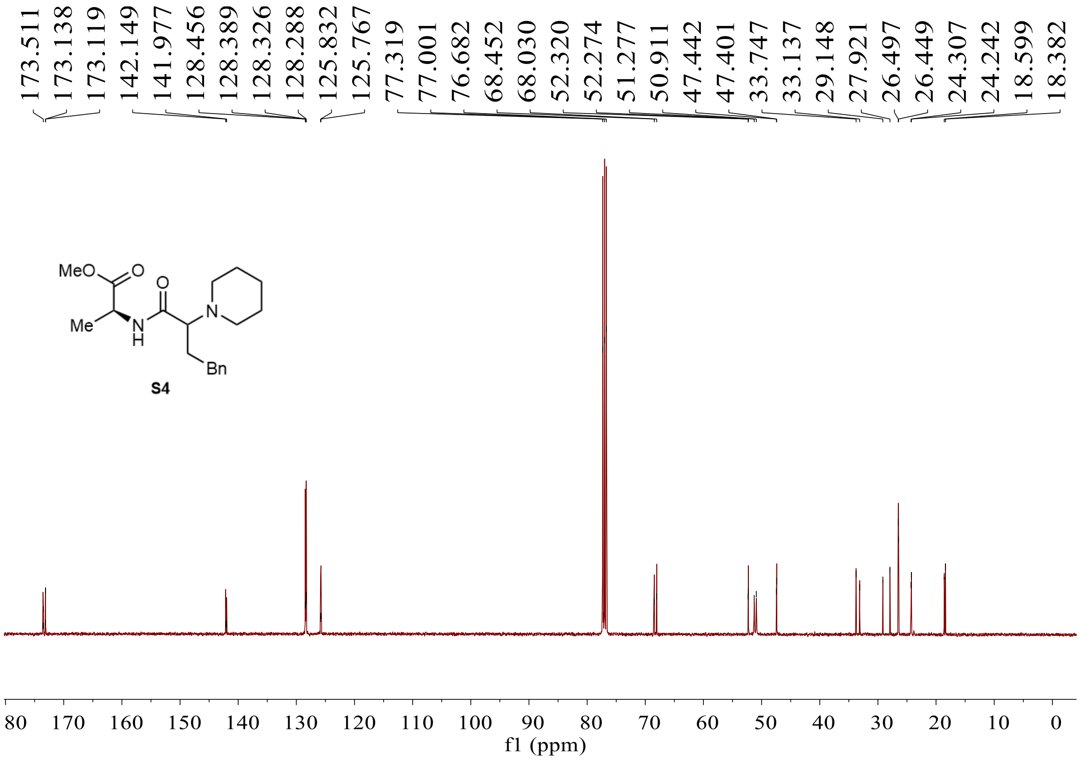

173.511
173.138
173.119
142.149
141.977
128.456
128.389
128.326
128.288
125.832
125.767
77.319
77.001
76.682
68.452
68.030
52.320
52.274
51.277
50.911
47.442
47.401
33.747
33.137
29.148
27.921
26.497
26.449
24.307
24.242
18.599
18.382
MeO
O
O
N
Me
N
H
Bn
S4
80 170 160 150 140 130 120 110 100 90 80 70 60 50 40 30 20 10 0
f1 (ppm)

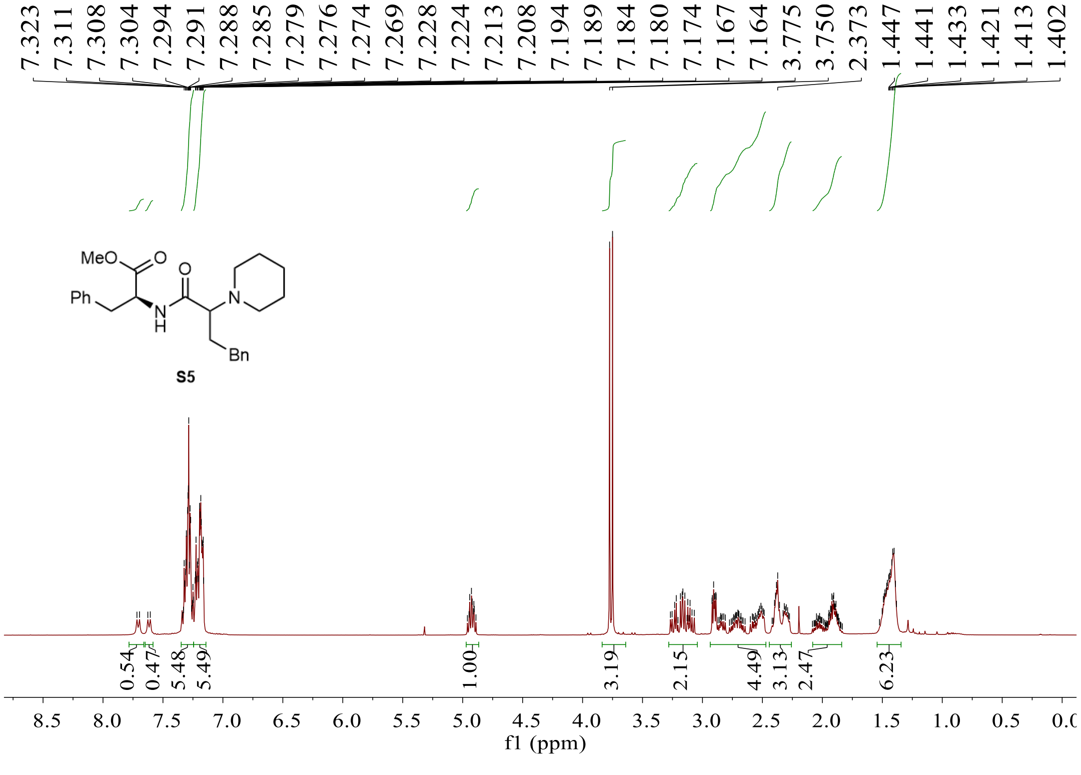

7.323
7.311
7.308
7.304
7.294
7.291
7.288
7.285
7.279
7.276
7.274
7.269
7.228
7.224
7.213
7.208
7.194
7.189
7.184
7.180
7.174
7.167
7.164
3.775
3.750
2.373
1.447
1.441
1.433
1.421
1.413
1.402
MeO
O
O
N
Ph
N
H
Bn
S5
0.54
0.47
5.48
5.49
1.00
3.19
2.15
4.49
3.13
2.47
6.23
8.5 8.0 7.5 7.0 6.5 6.0 5.5 5.0 4.5 4.0 3.5 3.0 2.5 2.0 1.5 1.0 0.5 0.0
f1 (ppm)

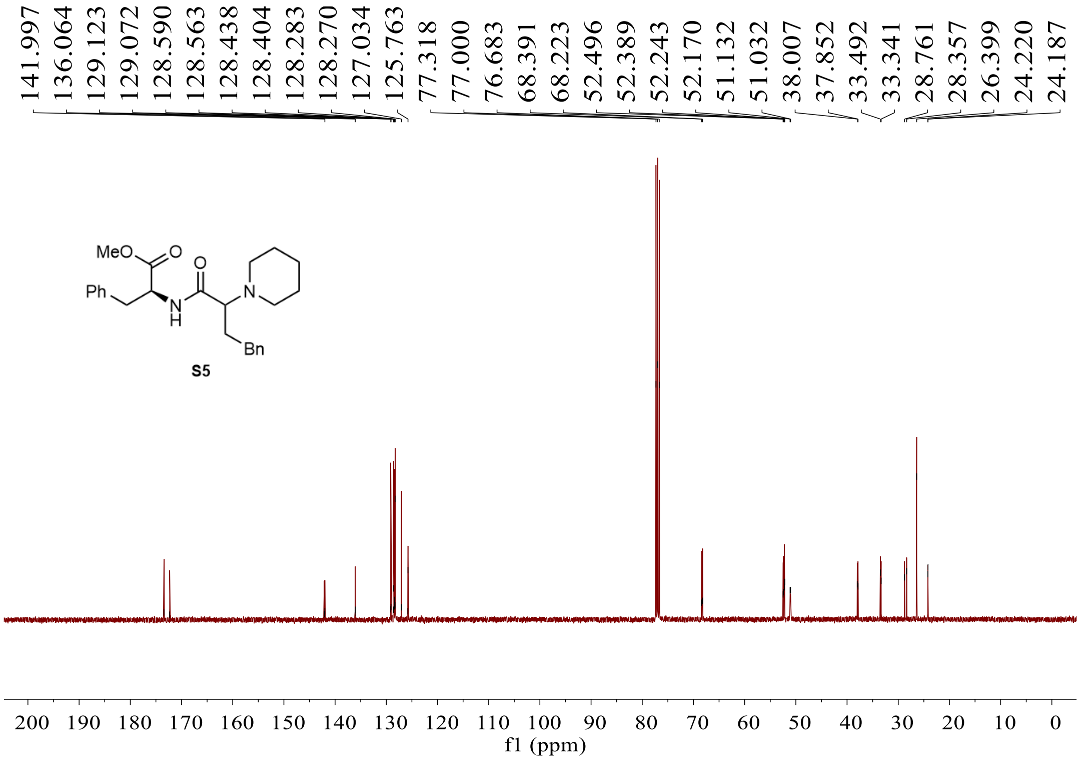

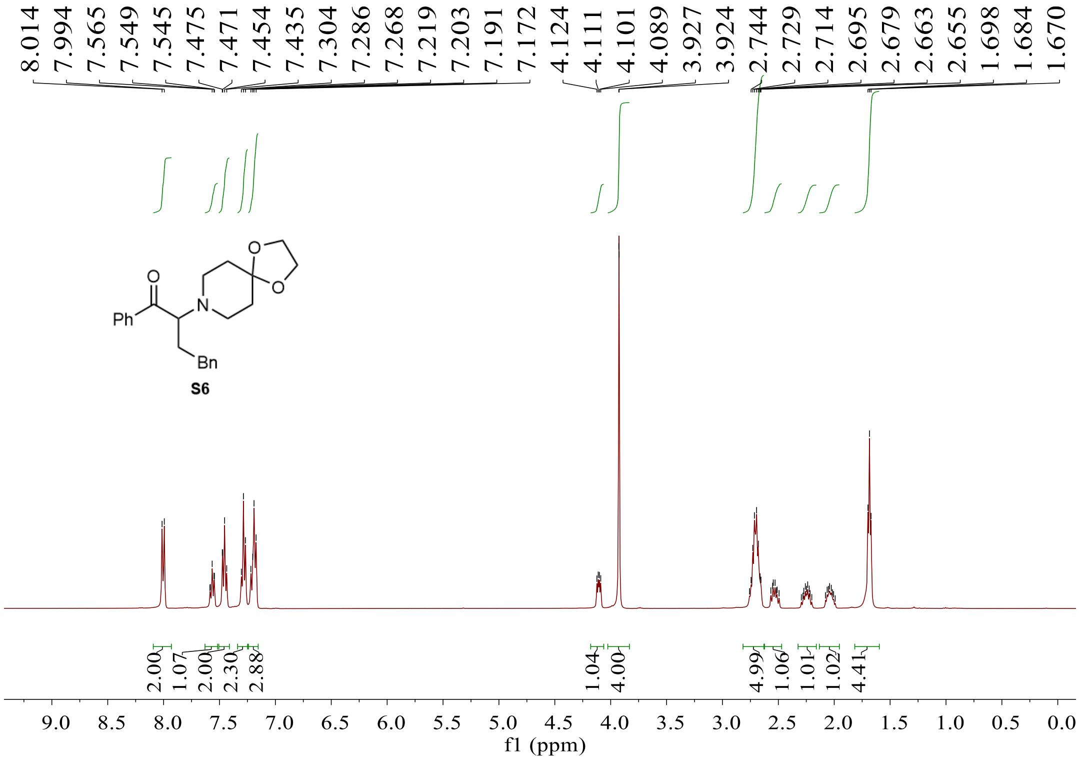

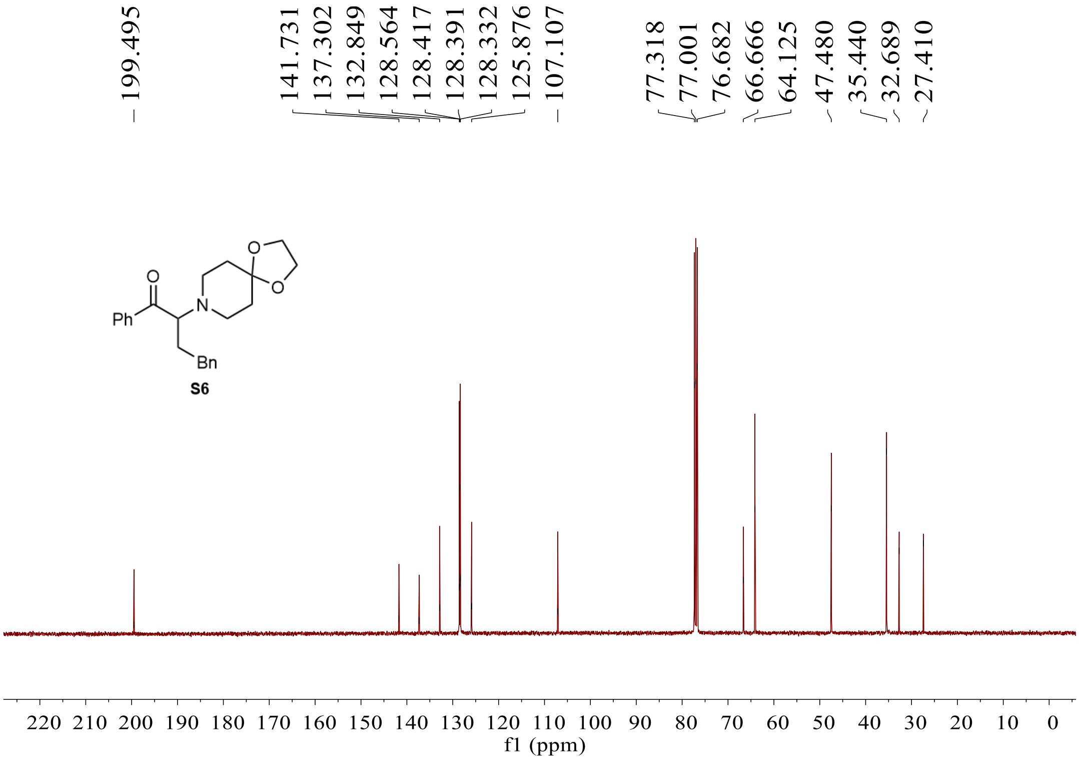
199.495
141.731
137.302
132.849
128.564
128.417
128.391
128.332
125.876
107.107
77.318
77.001
76.682
66.666
64.125
47.480
35.440
32.689
27.410
O
Ph
N
O
O
Bn
S6
220 210 200 190 180 170 160 150 140 130 120 110 100 90 80 70 60 50 40 30 20 10 0
f1 (ppm)

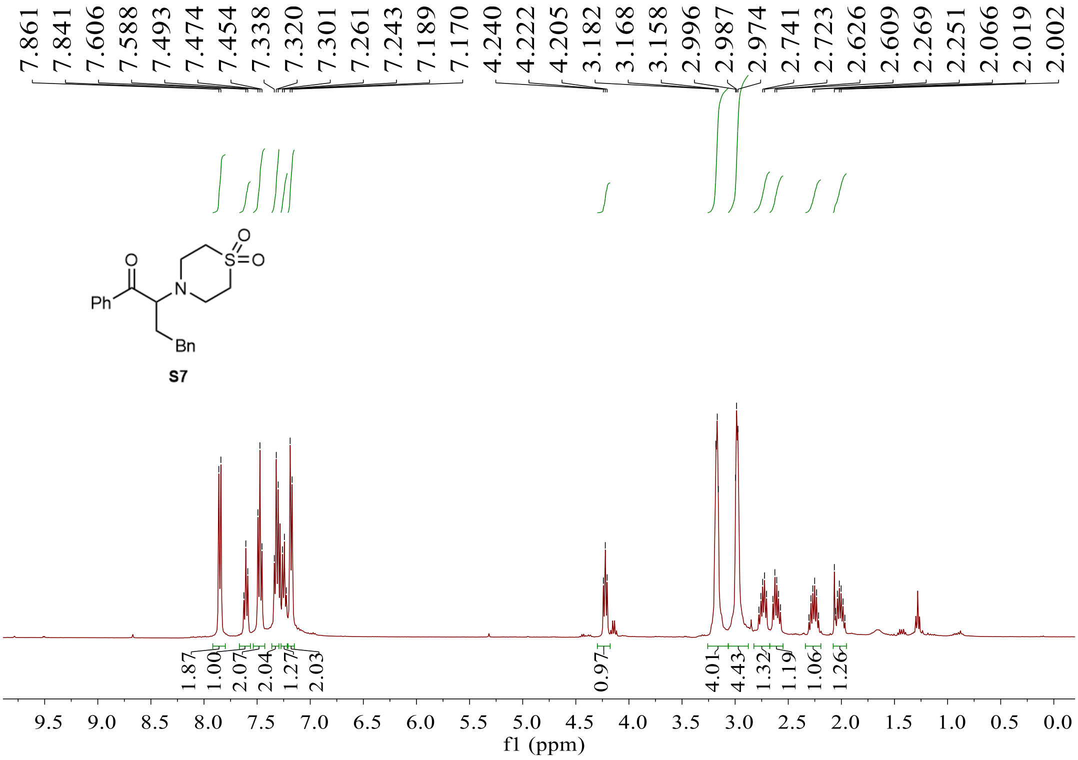
7.861
7.841
7.606
7.588
7.493
7.474
7.454
7.338
7.320
7.301
7.261
7.243
7.189
7.170
4.240
4.222
4.205
3.182
3.168
3.158
2.996
2.987
2.974
2.741
2.723
2.626
2.609
2.269
2.251
2.066
2.019
2.002
O
Ph
N
S
O
O
Bn
S7
1.87
1.00
2.07
2.04
1.27
2.03
0.97
4.01
4.43
1.32
1.19
1.06
1.26
9.5 9.0 8.5 8.0 7.5 7.0 6.5 6.0 5.5 5.0 4.5 4.0 3.5 3.0 2.5 2.0 1.5 1.0 0.5 0.0
f1 (ppm)

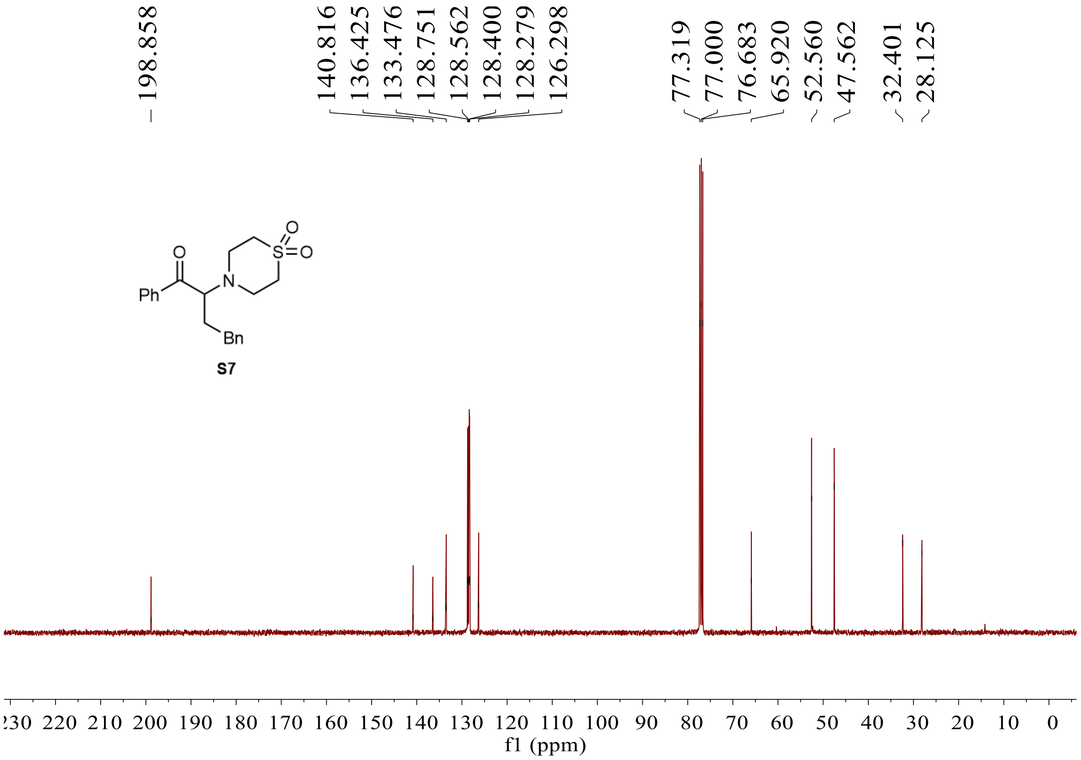
198.858
140.816
136.425
133.476
128.751
128.562
128.400
128.279
126.298
77.319
77.000
76.683
65.920
52.560
47.562
32.401
28.125
Ph
Bn
f1 (ppm)

S7

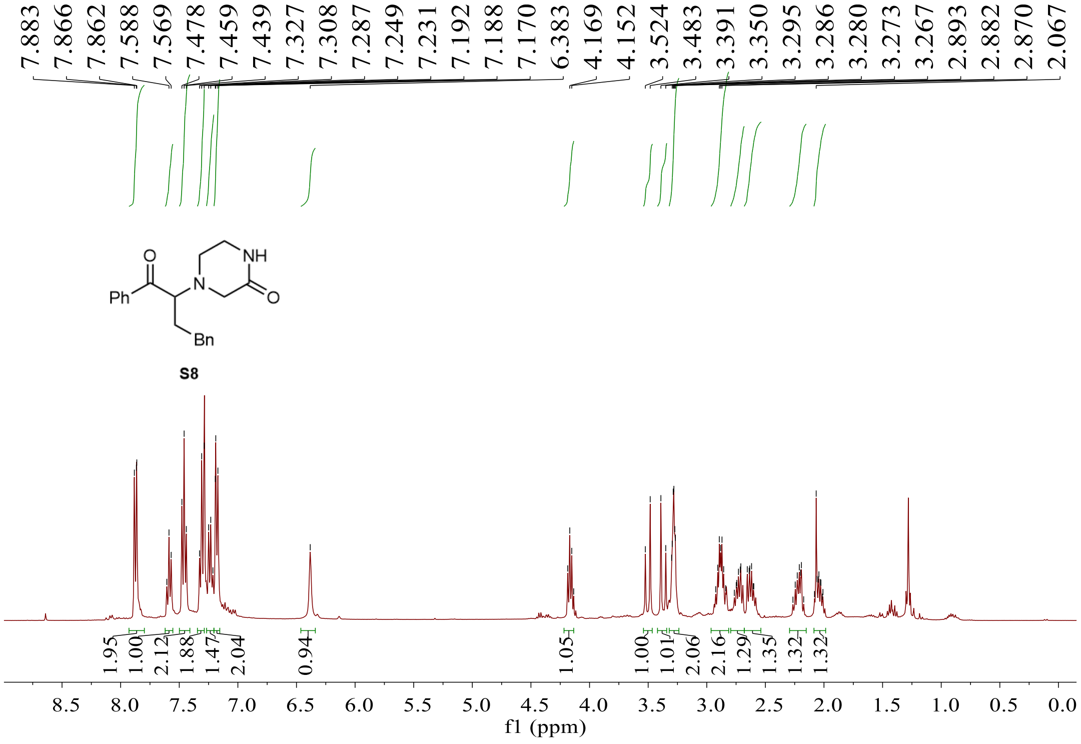
7.883
7.866
7.862
7.588
7.569
7.478
7.459
7.439
7.327
7.308
7.287
7.249
7.231
7.192
7.188
7.170
6.383
4.169
4.152
3.524
3.483
3.391
3.350
3.295
3.286
3.280
3.273
3.267
2.893
2.882
2.870
2.067
Ph
Bn
NH
f1 (ppm)

S8

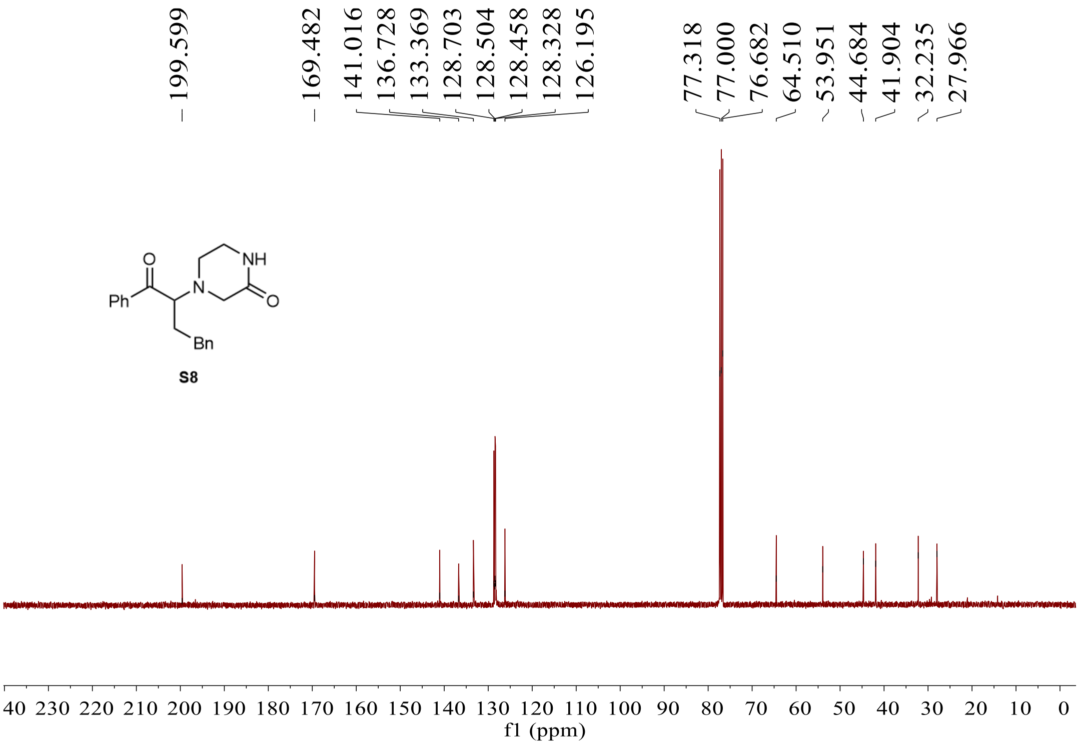
199.599
169.482
141.016
136.728
133.369
128.703
128.504
128.458
128.328
126.195
77.318
77.000
76.682
64.510
53.951
44.684
41.904
32.235
27.966
O
NH
Ph
N
O
Bn
S8
40 230 220 210 200 190 180 170 160 150 140 130 120 110 100 90 80 70 60 50 40 30 20 10 0
f1 (ppm)

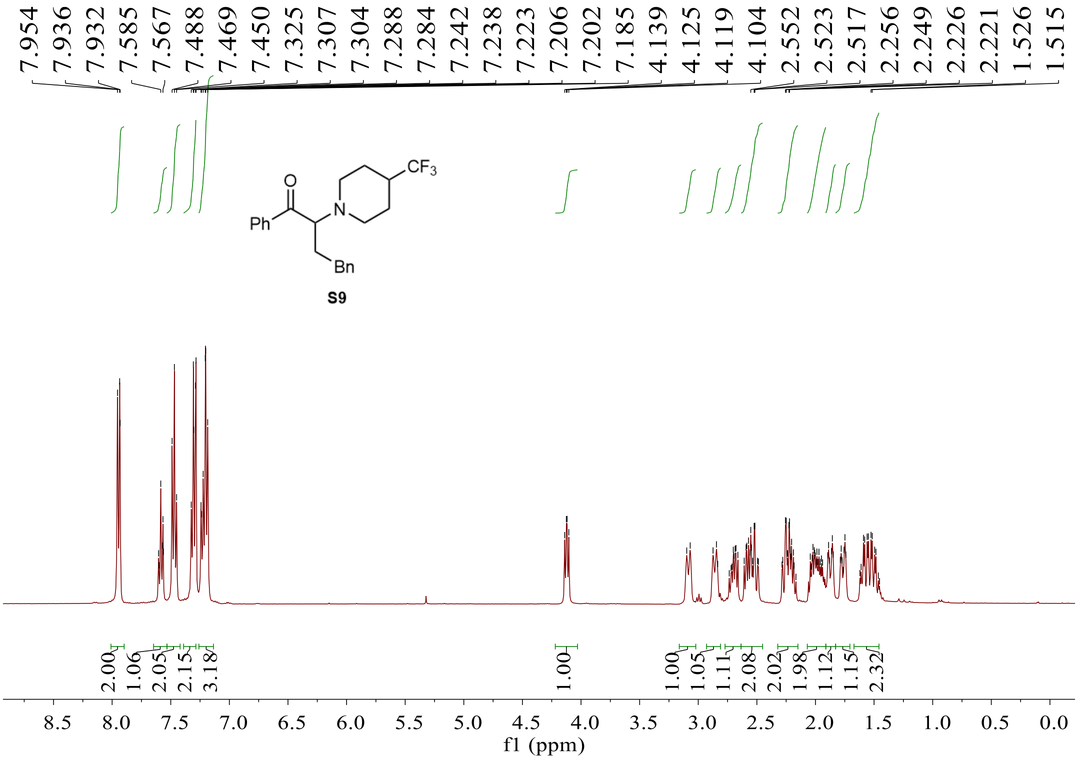
7.954
7.936
7.932
7.585
7.567
7.488
7.469
7.450
7.325
7.307
7.304
7.288
7.284
7.242
7.238
7.223
7.206
7.202
7.185
4.139
4.125
4.119
4.104
2.552
2.523
2.517
2.256
2.249
2.226
2.221
1.526
1.515
CF3
O
Ph
N
Bn
S9
2.00
1.06
2.05
2.15
3.18
1.00
1.00
1.05
1.11
2.08
2.02
1.98
1.12
1.15
2.32
8.5 8.0 7.5 7.0 6.5 6.0 5.5 5.0 4.5 4.0 3.5 3.0 2.5 2.0 1.5 1.0 0.5 0.0
f1 (ppm)

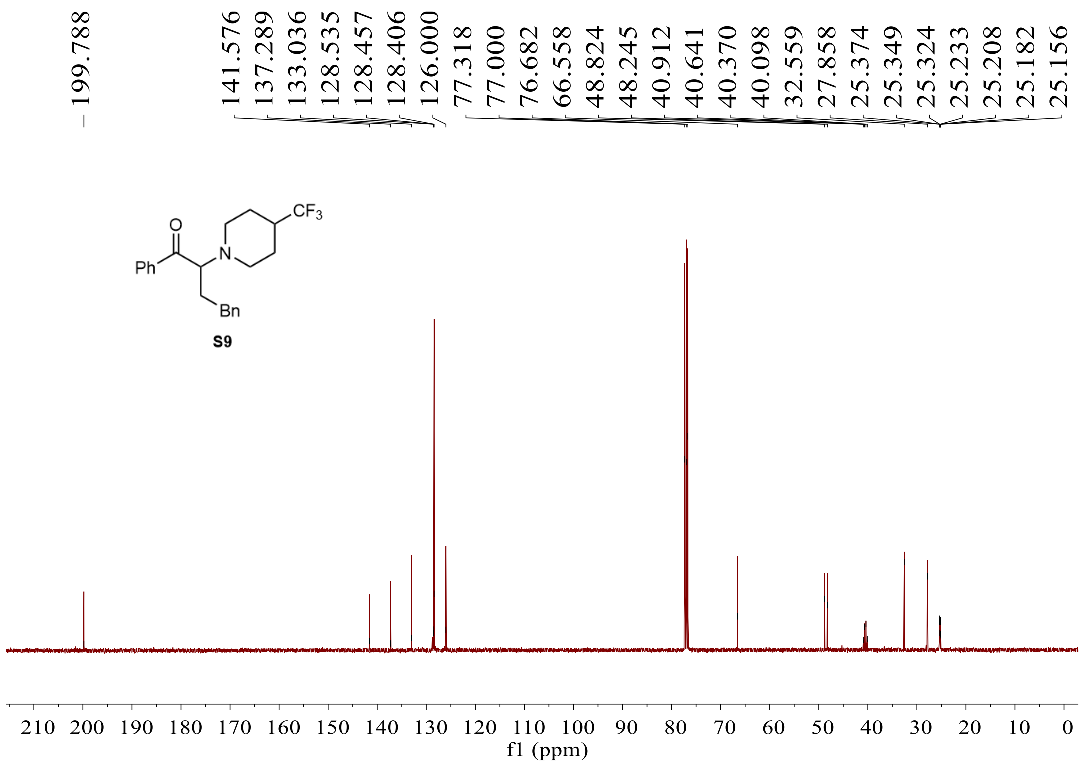
199.788
141.576
137.289
133.036
128.535
128.457
128.406
126.000
77.318
77.000
76.682
66.558
48.824
48.245
40.912
40.641
40.370
40.098
32.559
27.858
25.374
25.349
25.324
25.233
25.208
25.182
25.156
CF3
O
N
Ph
Bn
S9
210 200 190 180 170 160 150 140 130 120 110 100 90 80 70 60 50 40 30 20 10 0
f1 (ppm)

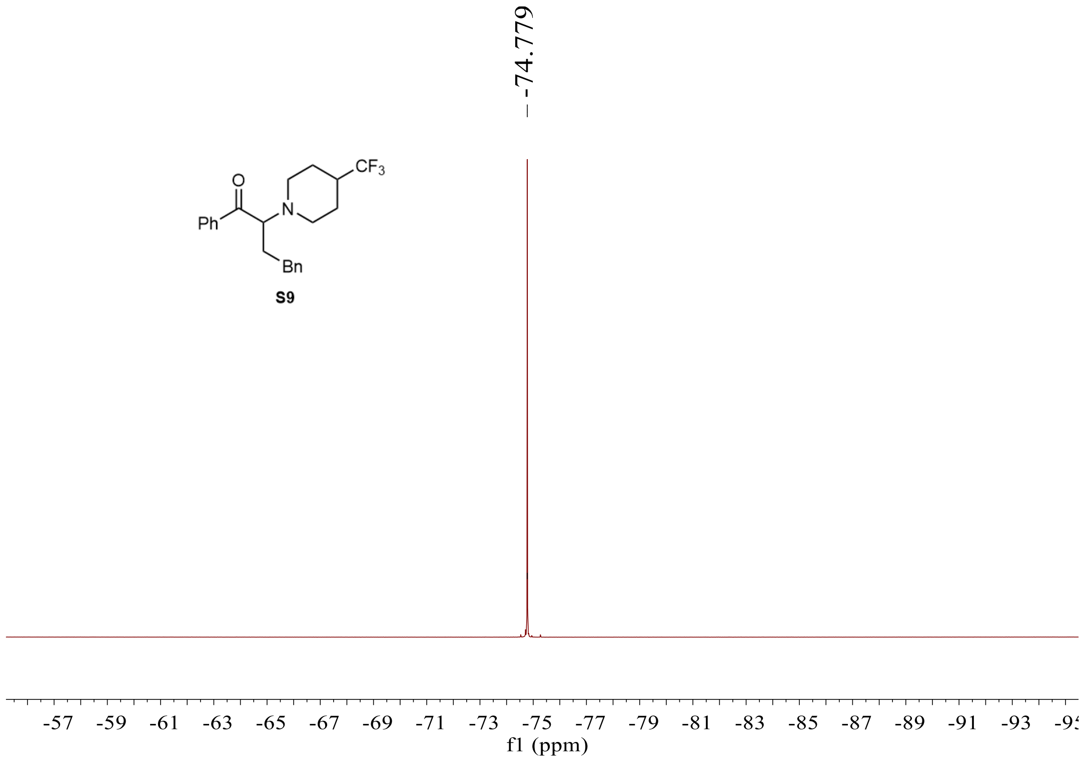
-74.779
CF3
O
N
Ph
Bn
S9
-57 -59 -61 -63 -65 -67 -69 -71 -73 -75 -77 -79 -81 -83 -85 -87 -89 -91 -93
f1 (ppm)

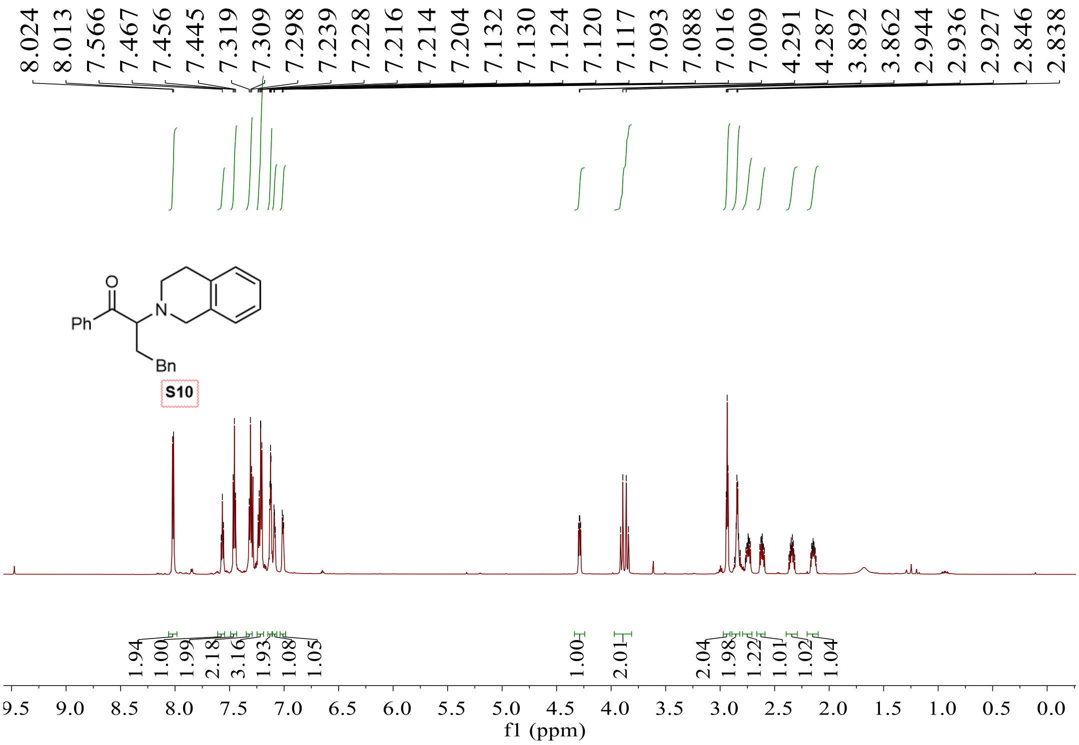
8.024
8.013
7.566
7.467
7.456
7.445
7.319
7.309
7.298
7.239
7.228
7.216
7.214
7.204
7.132
7.130
7.124
7.120
7.117
7.093
7.088
7.016
7.009
4.291
4.287
3.892
3.862
2.944
2.936
2.927
2.846
2.838
O
Ph
N
Bn
S10
1.94
1.00
1.99
2.18
3.16
1.93
1.08
1.05
1.00
2.01
2.04
1.98
1.22
1.01
1.02
1.04
9.5 9.0 8.5 8.0 7.5 7.0 6.5 6.0 5.5 5.0 4.5 4.0 3.5 3.0 2.5 2.0 1.5 1.0 0.5 0.0
f1 (ppm)

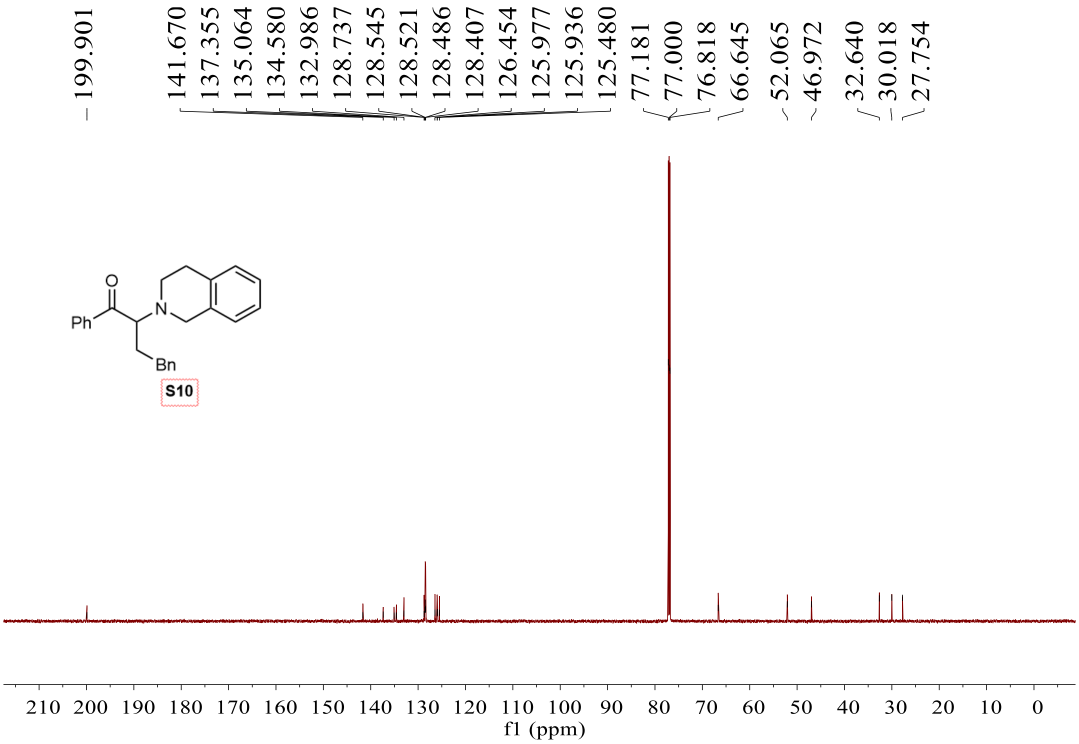
199.901
141.670
137.355
135.064
134.580
132.986
128.737
128.545
128.521
128.486
128.407
126.454
125.977
125.936
125.480
77.181
77.000
76.818
66.645
52.065
46.972
32.640
30.018
27.754
O
Ph
N
Bn
S10
210 200 190 180 170 160 150 140 130 120 110 100 90 80 70 60 50 40 30 20 10 0
f1 (ppm)

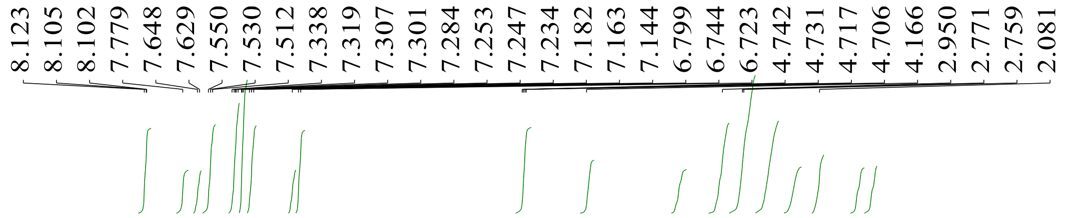

S11

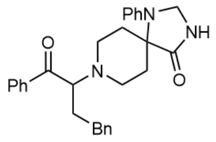

S11

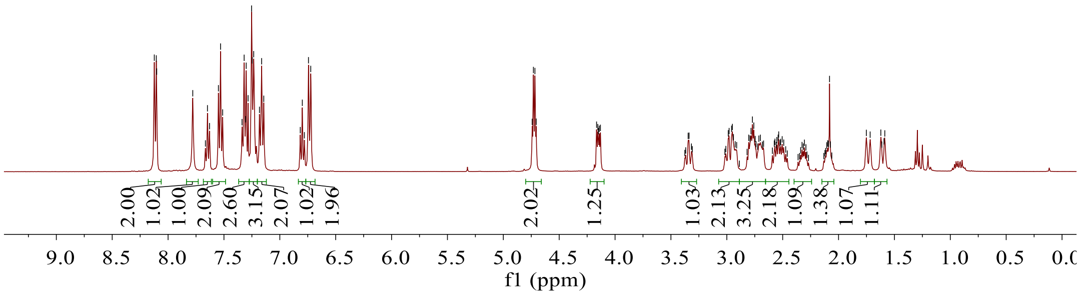

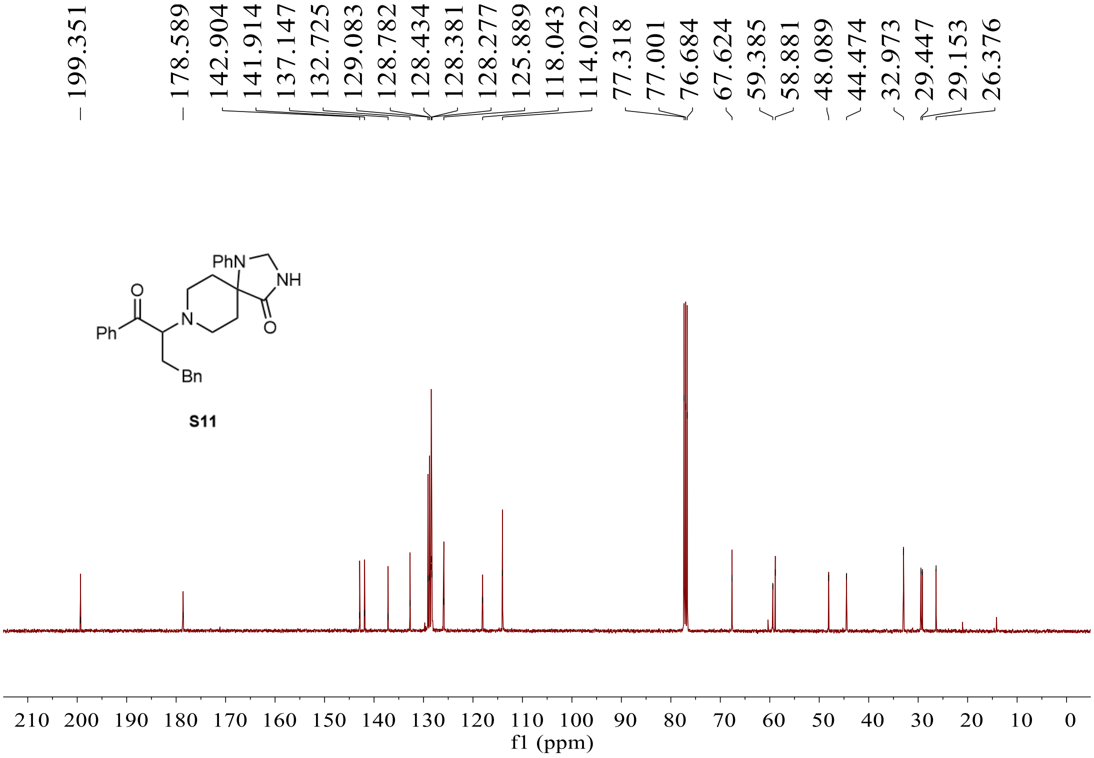

S11

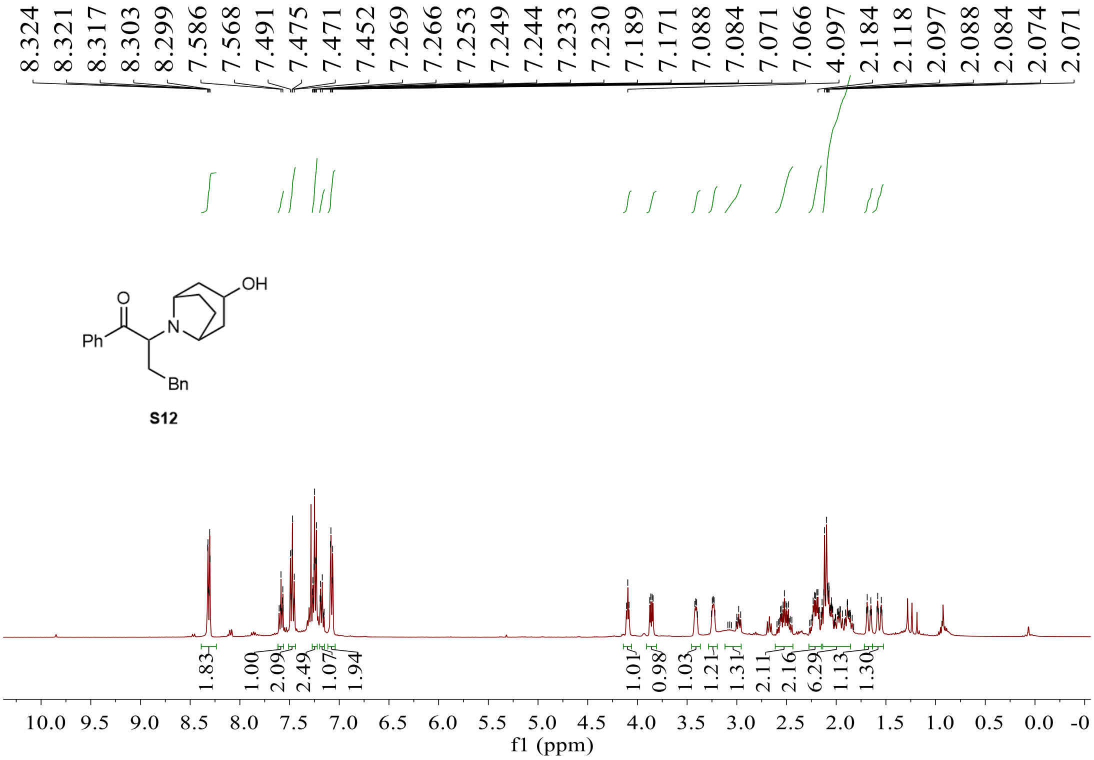
8.324 8.321 8.317 8.303 8.299 7.586 7.568 7.491 7.475 7.471 7.452 7.269 7.266 7.253 7.249 7.244 7.233 7.230 7.189 7.171 7.088 7.084 7.071 7.066 4.097 2.184 2.118 2.097 2.088 2.084 2.074 2.071
O
OH
N
Ph
Bn
S12
1.83 1.00 2.09 2.49 1.07 1.94 1.01 0.98 1.03 1.21 1.31 2.11 2.16 6.29 1.13 1.30
10.0 9.5 9.0 8.5 8.0 7.5 7.0 6.5 6.0 5.5 5.0 4.5 4.0 3.5 3.0 2.5 2.0 1.5 1.0 0.5 0.0 -0
f1 (ppm)

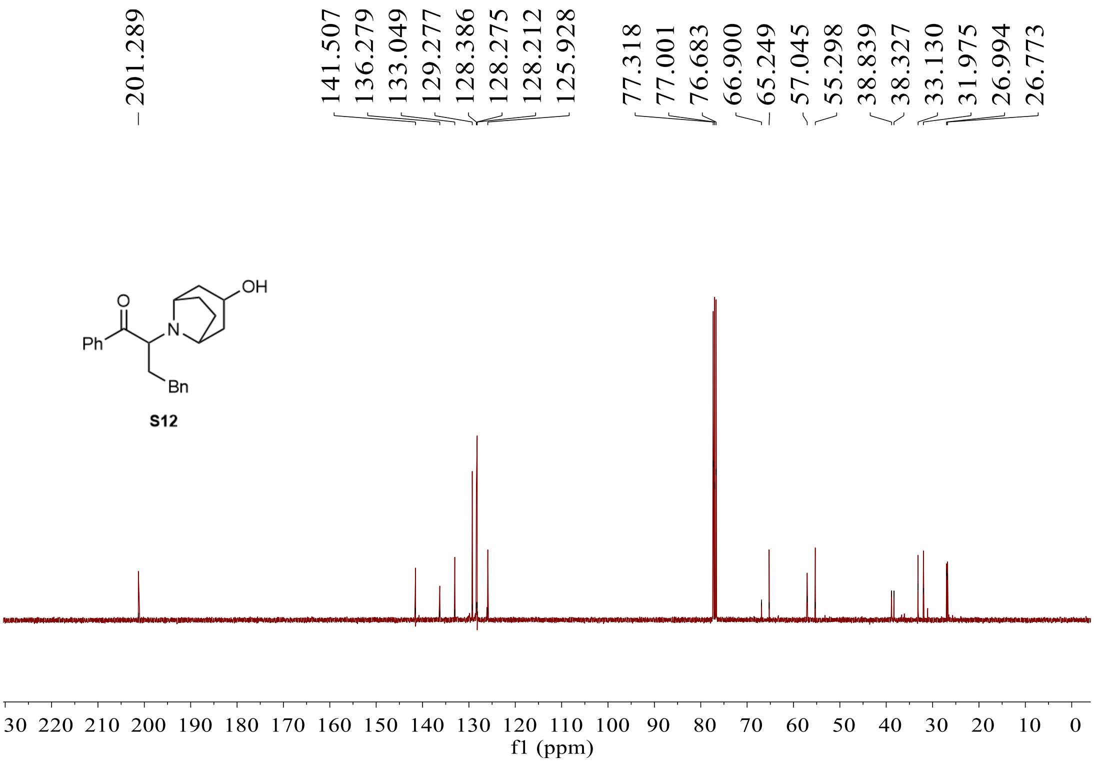
201.289
141.507 136.279 133.049 129.277 128.386 128.275 128.212 125.928
77.318 77.001 76.683 66.900 65.249 57.045 55.298 38.839 38.327 33.130 31.975 26.994 26.773
O
OH
N
Ph
Bn
S12
30 220 210 200 190 180 170 160 150 140 130 120 110 100 90 80 70 60 50 40 30 20 10 0
f1 (ppm)

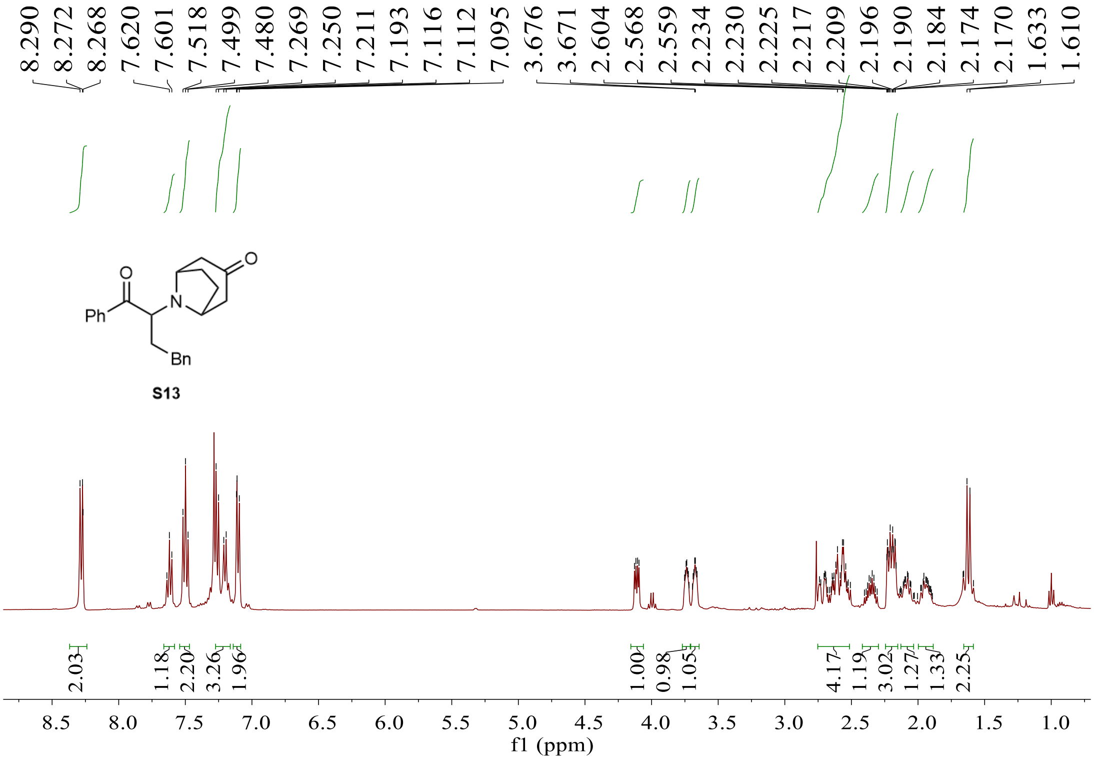
8.290
8.272
8.268
7.620
7.601
7.518
7.499
7.480
7.269
7.250
7.211
7.193
7.116
7.112
7.095
3.676
3.671
2.604
2.568
2.559
2.234
2.230
2.225
2.217
2.209
2.196
2.190
2.184
2.174
2.170
1.633
1.610
O
O
N
Ph
Bn
S13
2.03
1.18
2.20
3.26
1.96
1.00
0.98
1.05
4.17
1.19
3.02
1.27
1.33
2.25
8.5 8.0 7.5 7.0 6.5 6.0 5.5 5.0 4.5 4.0 3.5 3.0 2.5 2.0 1.5 1.0
f1 (ppm)

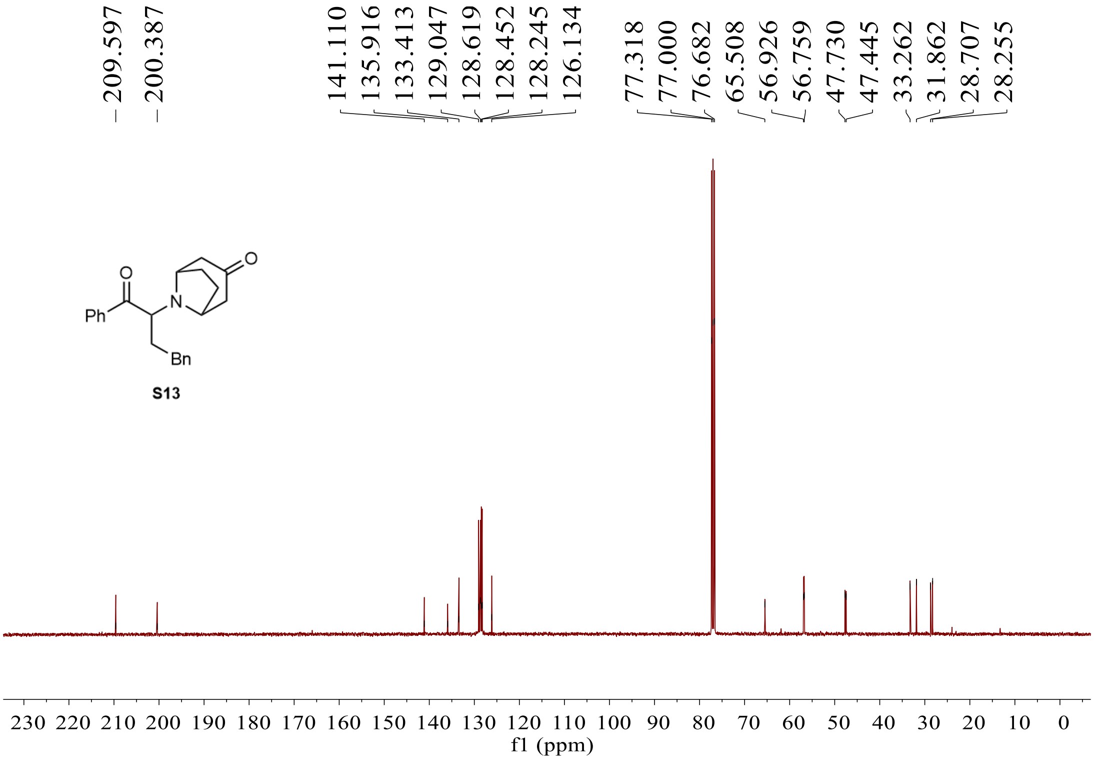
209.597
200.387
141.110
135.916
133.413
129.047
128.619
128.452
128.245
126.134
77.318
77.000
76.682
65.508
56.926
56.759
47.730
47.445
33.262
31.862
28.707
28.255
O
O
N
Ph
Bn
S13
230 220 210 200 190 180 170 160 150 140 130 120 110 100 90 80 70 60 50 40 30 20 10 0
f1 (ppm)

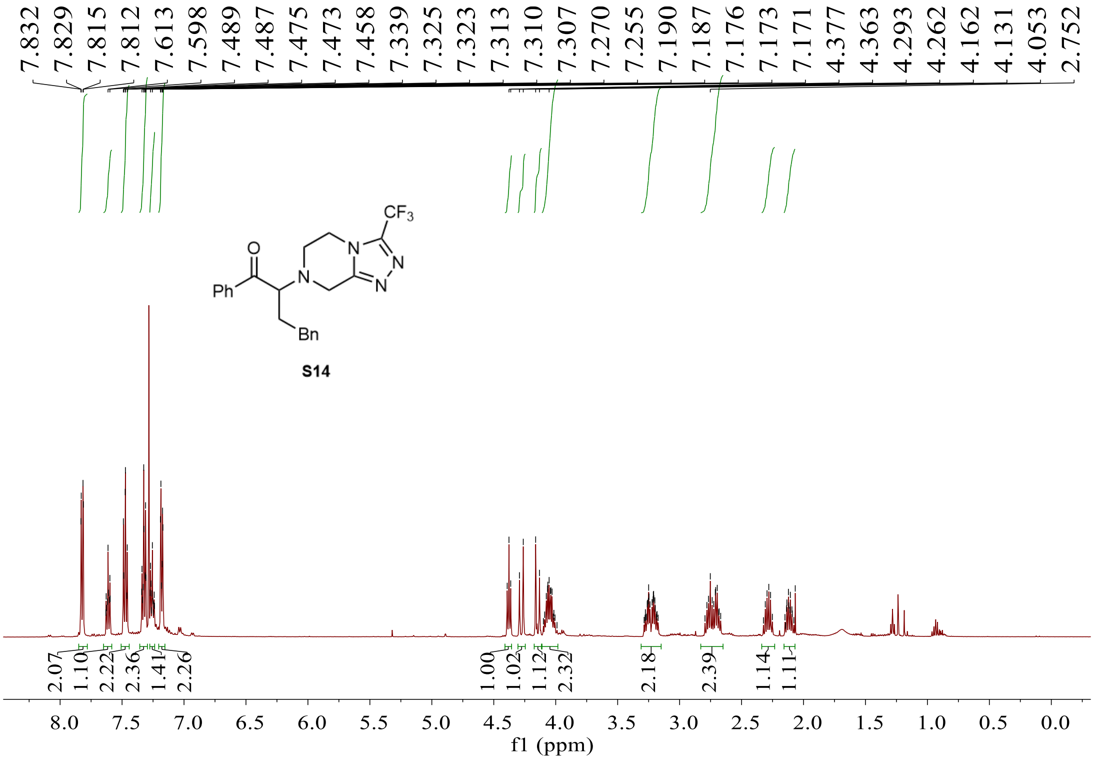
7.832 7.829 7.815 7.812 7.613 7.598 7.489 7.487 7.475 7.473 7.458 7.339 7.325 7.323 7.313 7.310 7.307 7.270 7.255 7.190 7.187 7.176 7.173 7.171 4.377 4.363 4.293 4.262 4.162 4.131 4.053 2.752
CF3
O
Ph
N
N
N
N
Bn
S14
2.07 1.10 2.22 2.36 1.41 2.26
1.00 1.02 1.12 2.32
2.18
2.39
1.14 1.11
8.0 7.5 7.0 6.5 6.0 5.5 5.0 4.5 4.0 3.5 3.0 2.5 2.0 1.5 1.0 0.5 0.0
f1 (ppm)

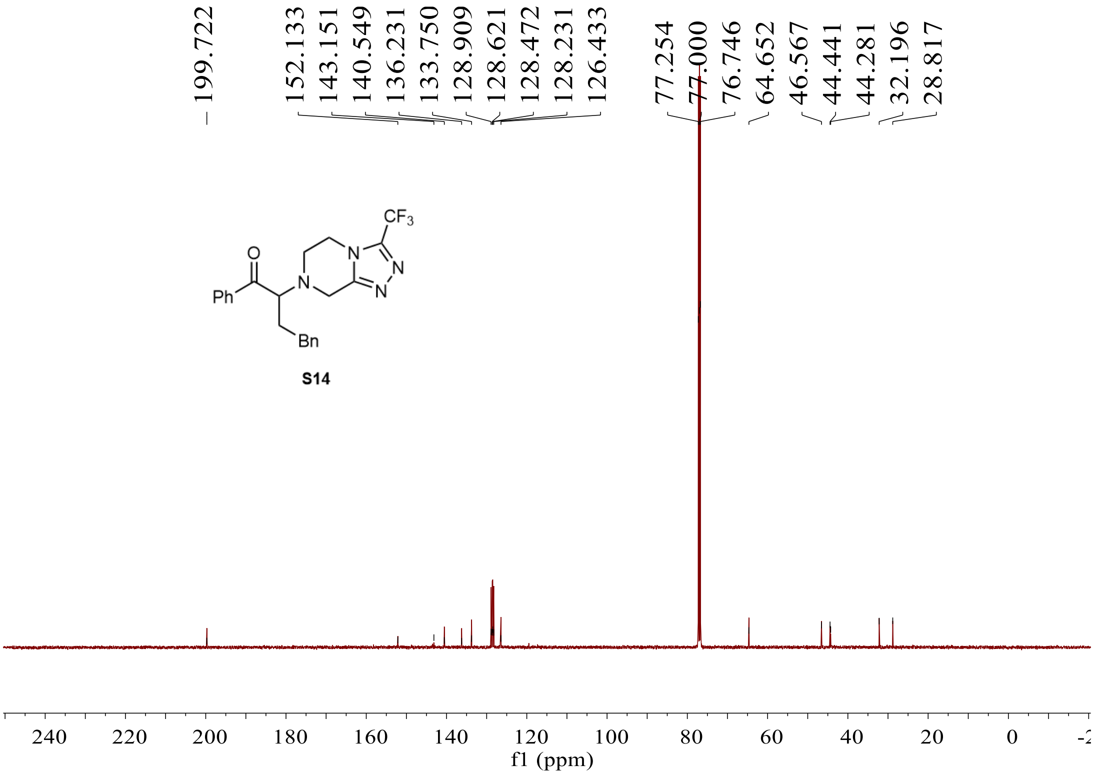
199.722
152.133 143.151 140.549 136.231 133.750 128.909 128.621 128.472 128.231 126.433
77.254 77.000 76.746 64.652 46.567 44.441 44.281 32.196 28.817
CF3
O
Ph
N
N
N
N
Bn
S14
240 220 200 180 160 140 120 100 80 60 40 20 0
f1 (ppm)

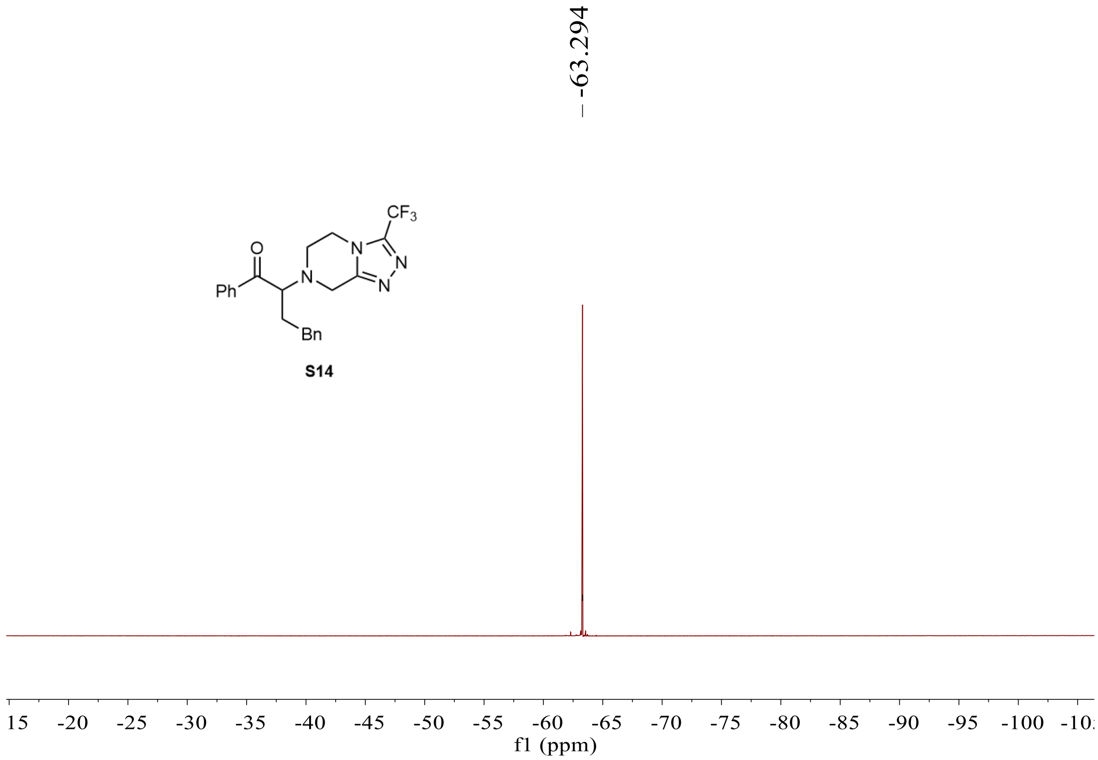
-63.294
CF3
O
Ph
N
N
N
N
Bn
S14
15 -20 -25 -30 -35 -40 -45 -50 -55 -60 -65 -70 -75 -80 -85 -90 -95 -100
f1 (ppm)

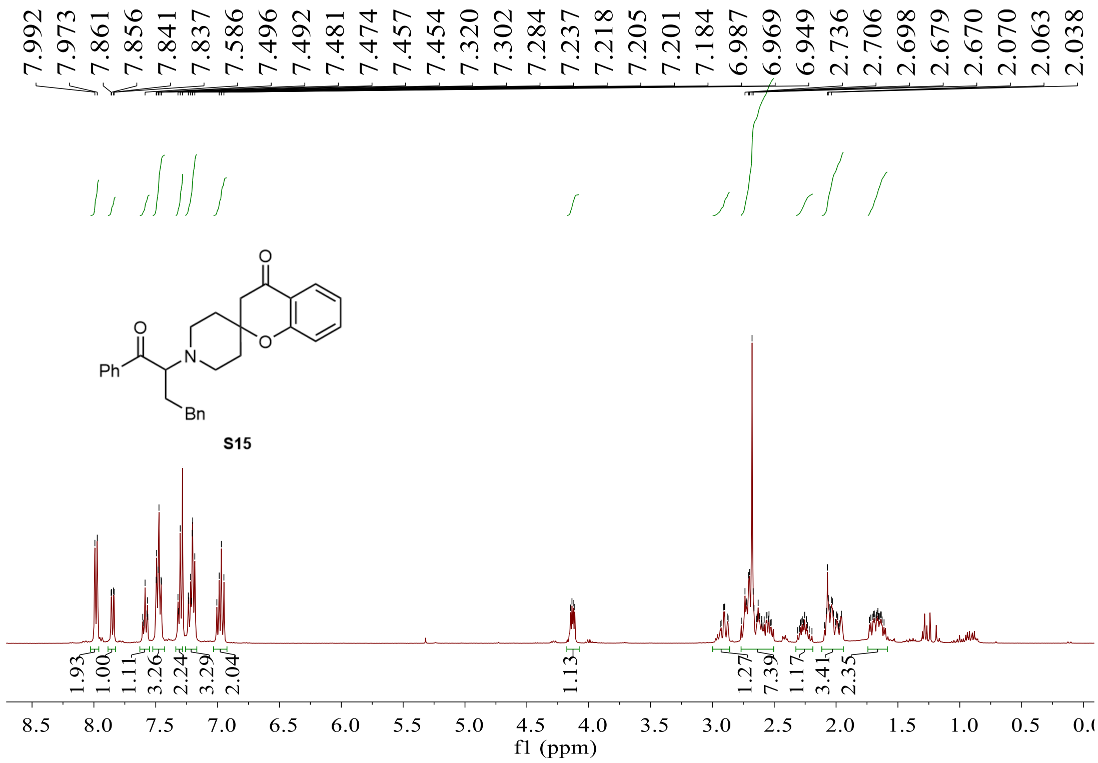
7.992 7.973 7.861 7.856 7.841 7.837 7.586 7.496 7.492 7.481 7.474 7.457 7.454 7.320 7.302 7.284 7.237 7.218 7.205 7.201 7.184 6.987 6.969 6.949 2.736 2.706 2.698 2.679 2.670 2.070 2.063 2.038
O
O
Ph
N
O
Bn
S15
1.93 1.00 1.11 3.26 2.24 3.29 2.04 1.13 1.27 7.39 1.17 3.41 2.35
8.5 8.0 7.5 7.0 6.5 6.0 5.5 5.0 4.5 4.0 3.5 3.0 2.5 2.0 1.5 1.0 0.5
f1 (ppm)

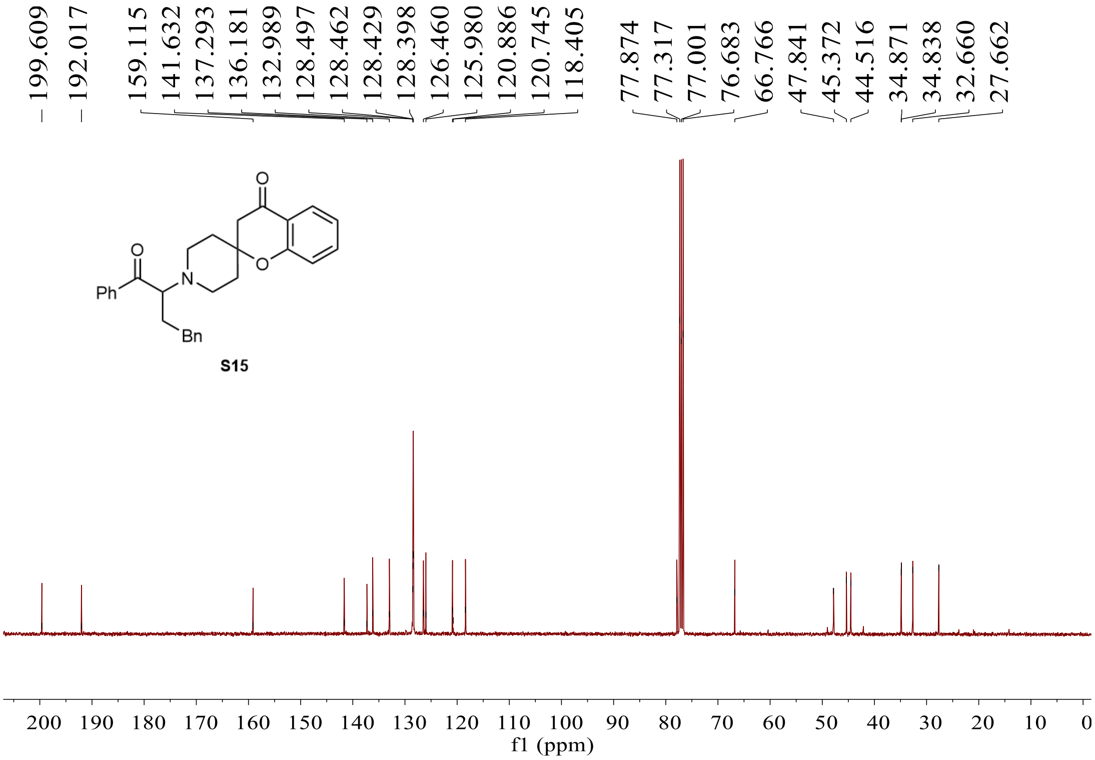

199.609
192.017
159.115
141.632
137.293
136.181
132.989
128.497
128.462
128.429
128.398
126.460
125.980
120.886
120.745
118.405
77.874
77.317
77.001
76.683
66.766
47.841
45.372
44.516
34.871
34.838
32.660
27.662
O
O
N
O
Ph
Bn
S15
200 190 180 170 160 150 140 130 120 110 100 90 80 70 60 50 40 30 20 10 0
f1 (ppm)

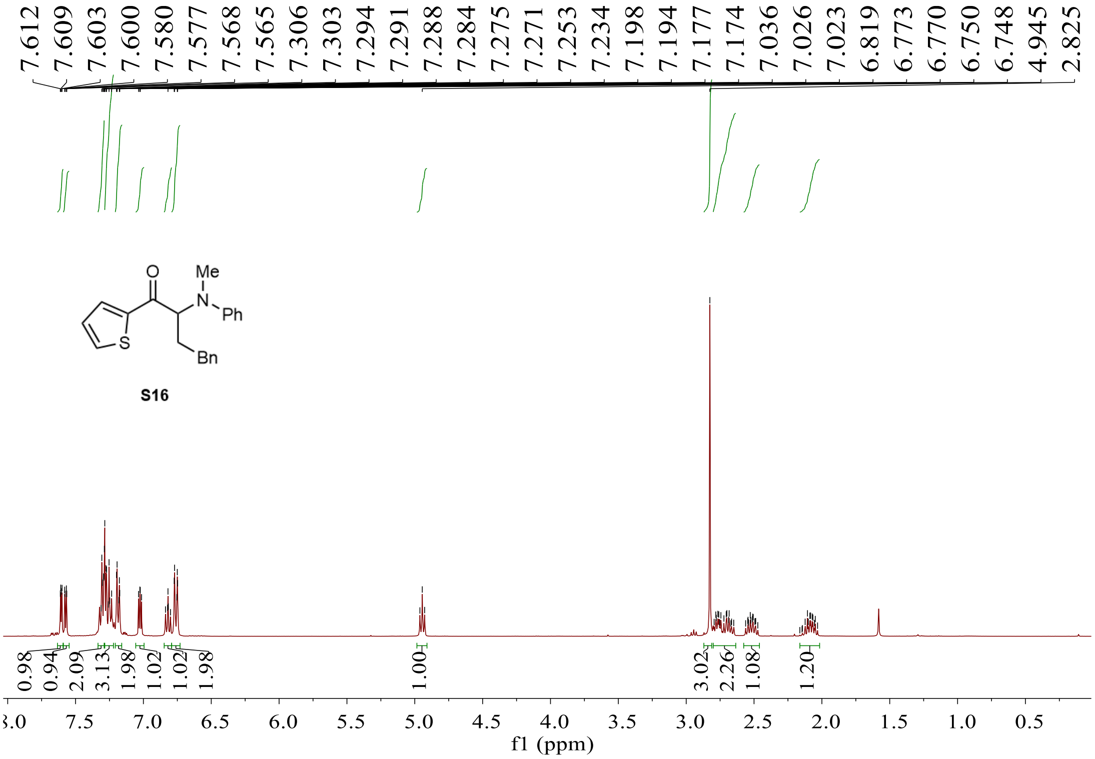

7.612
7.609
7.603
7.600
7.580
7.577
7.568
7.565
7.306
7.303
7.294
7.291
7.288
7.284
7.275
7.271
7.253
7.234
7.198
7.194
7.177
7.174
7.036
7.026
7.023
6.819
6.773
6.770
6.750
6.748
4.945
2.825
O
Me
N
Ph
S
Bn
S16
0.98
0.94
2.09
3.13
1.98
1.02
1.02
1.98
1.00
3.02
2.26
1.08
1.20
7.5 7.0 6.5 6.0 5.5 5.0 4.5 4.0 3.5 3.0 2.5 2.0 1.5 1.0 0.5
f1 (ppm)

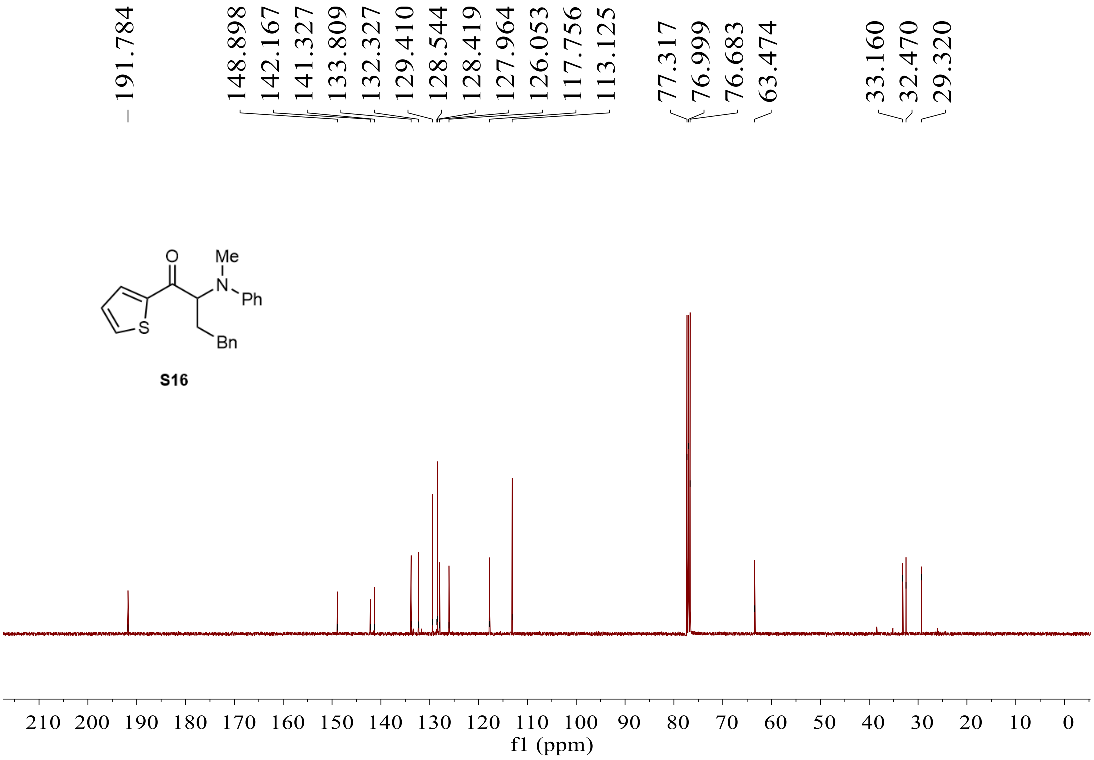
191.784
148.898
142.167
141.327
133.809
132.327
129.410
128.544
128.419
127.964
126.053
117.756
113.125
77.317
76.999
76.683
63.474
33.160
32.470
29.320
Me
N
Ph
O
S
Bn
S16
210 200 190 180 170 160 150 140 130 120 110 100 90 80 70 60 50 40 30 20 10 0
f1 (ppm)

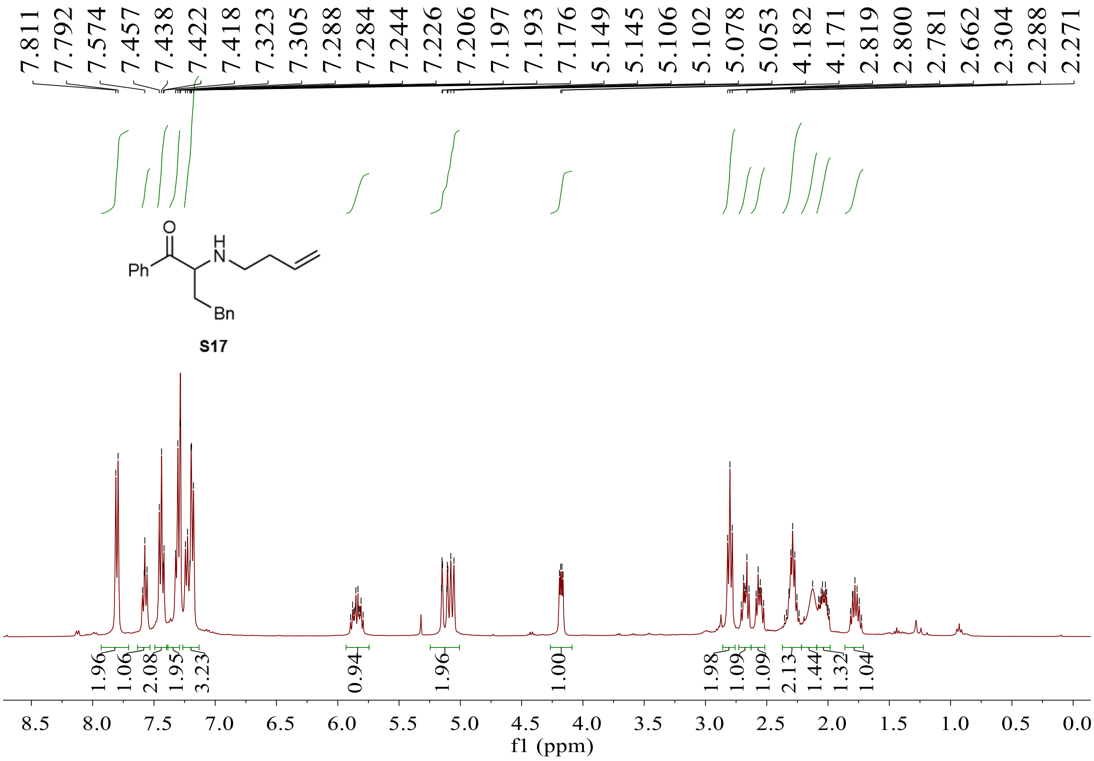
7.811
7.792
7.574
7.457
7.438
7.422
7.418
7.323
7.305
7.288
7.284
7.244
7.226
7.206
7.197
7.193
7.176
5.149
5.145
5.106
5.102
5.078
5.053
4.182
4.171
2.819
2.800
2.781
2.662
2.304
2.288
2.271
Ph
O
H
N
Bn
S17
1.96
1.06
2.08
1.95
3.23
0.94
1.96
1.00
1.98
1.09
1.09
2.13
1.44
1.32
1.04
8.5 8.0 7.5 7.0 6.5 6.0 5.5 5.0 4.5 4.0 3.5 3.0 2.5 2.0 1.5 1.0 0.5 0.0
f1 (ppm)

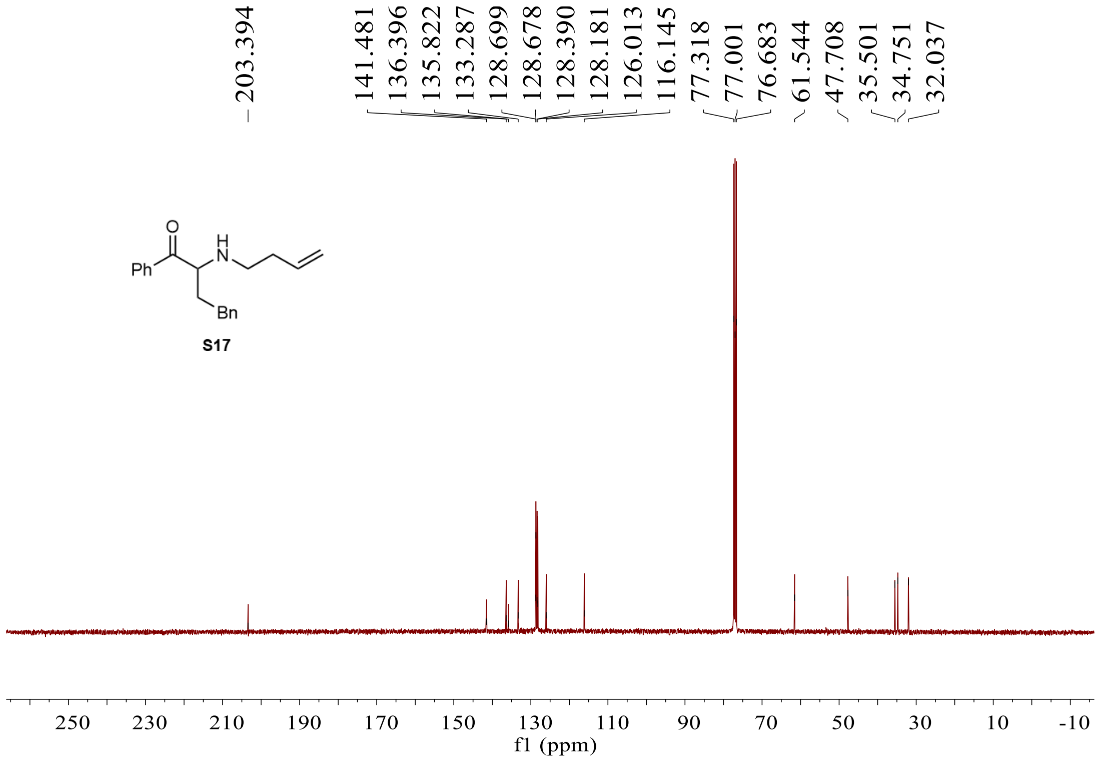

203.394
141.481
136.396
135.822
133.287
128.699
128.678
128.390
128.181
126.013
116.145
77.318
77.001
76.683
61.544
47.708
35.501
34.751
32.037
Ph
O
H
N
Bn
S17
250 230 210 190 170 150 130 110 90 70 50 30 10 -10
f1 (ppm)

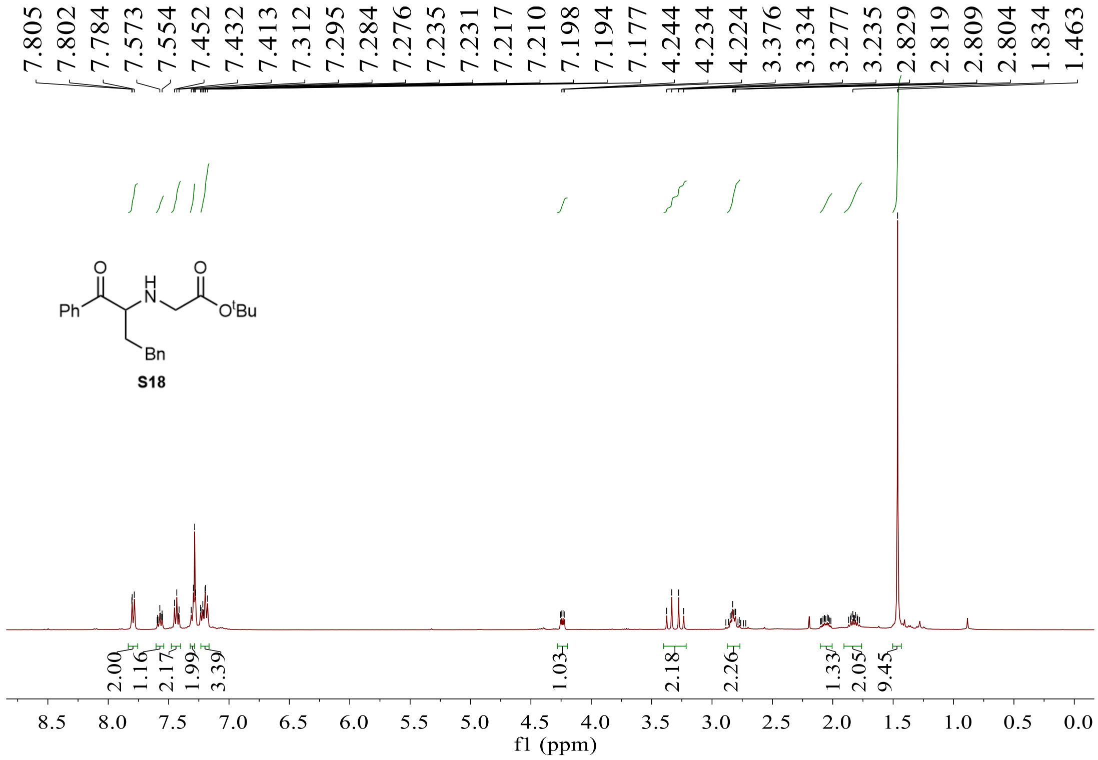

7.805
7.802
7.784
7.573
7.554
7.452
7.432
7.413
7.312
7.295
7.284
7.276
7.235
7.231
7.217
7.210
7.198
7.194
7.177
4.244
4.234
4.224
3.376
3.334
3.277
3.235
2.829
2.819
2.809
2.804
1.834
1.463
Ph
O
H
N
O
O^{t}Bu
Bn
S18
2.00
1.16
2.17
1.99
3.39
1.03
2.18
2.26
1.33
2.05
9.45
8.5 8.0 7.5 7.0 6.5 6.0 5.5 5.0 4.5 4.0 3.5 3.0 2.5 2.0 1.5 1.0 0.5 0.0
f1 (ppm)

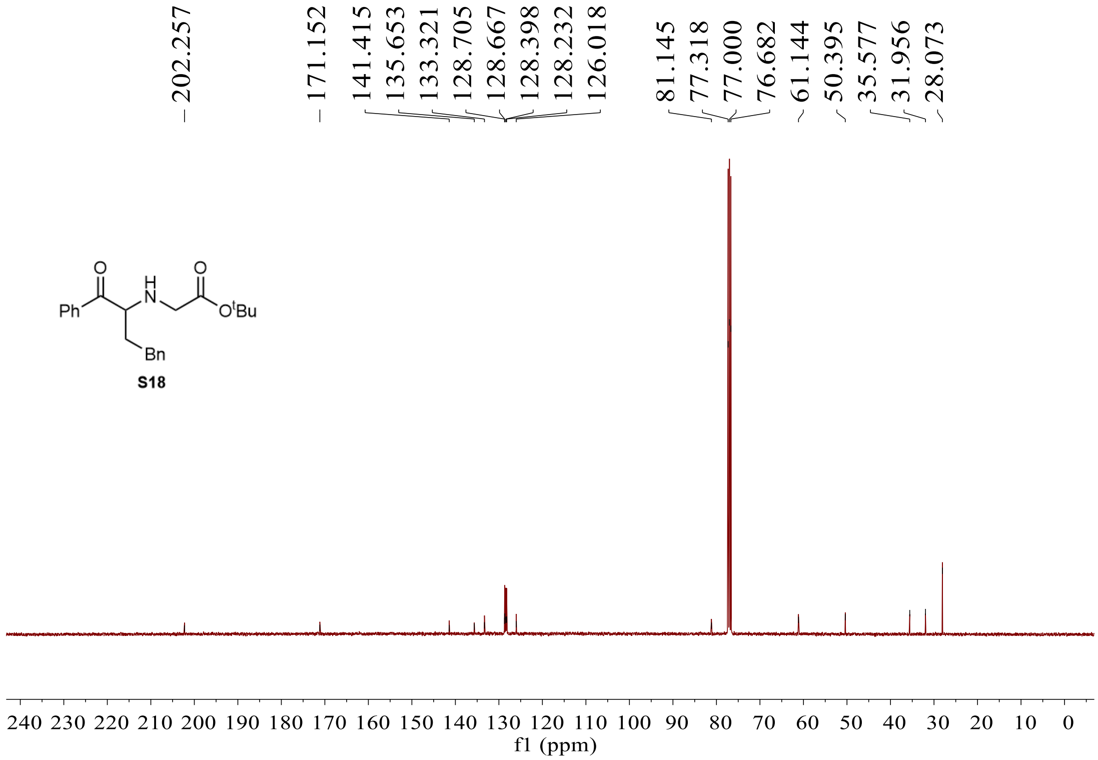

202.257
171.152
141.415
135.653
133.321
128.705
128.667
128.398
128.232
126.018
81.145
77.318
77.000
76.682
61.144
50.395
35.577
31.956
28.073
Ph
O
H
N
O
OtBu
Bn
240 230 220 210 200 190 180 170 160 150 140 130 120 110 100 90 80 70 60 50 40 30 20 10 0
f1 (ppm)

S18

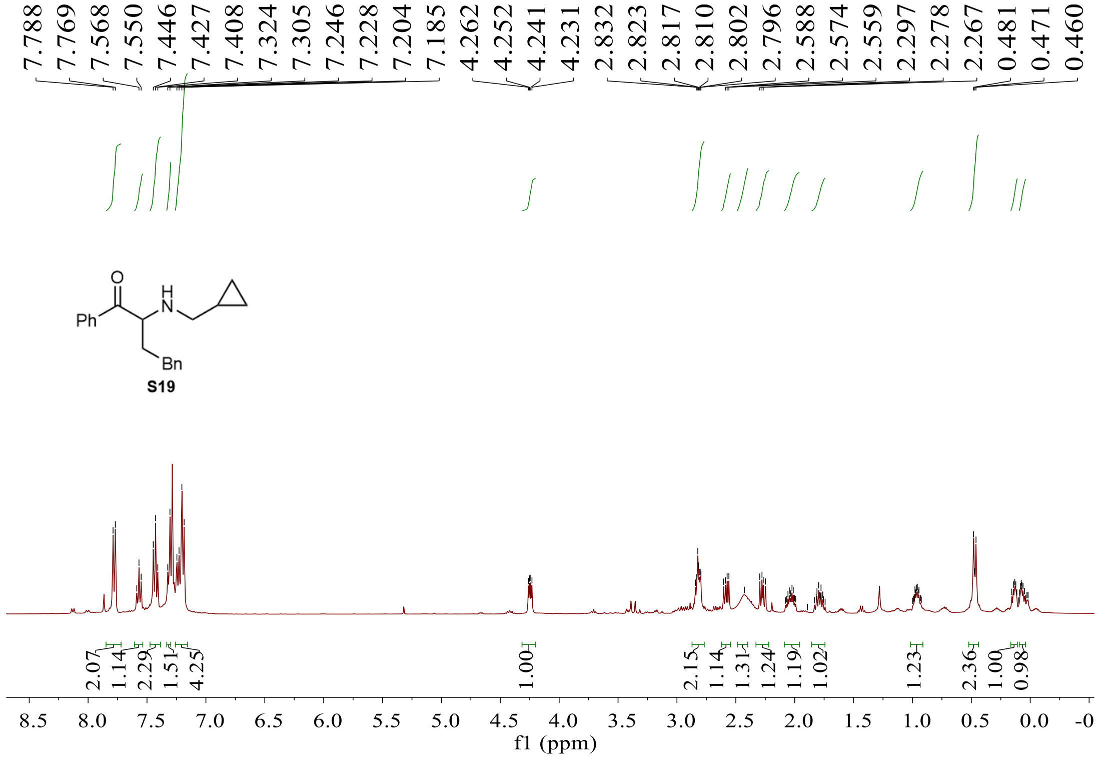

7.788
7.769
7.568
7.550
7.446
7.427
7.408
7.324
7.305
7.246
7.228
7.204
7.185
4.262
4.252
4.241
4.231
2.832
2.823
2.817
2.810
2.802
2.796
2.588
2.574
2.559
2.297
2.278
2.267
0.481
0.471
0.460
Ph
O
H
N
Bn
2.07
1.14
2.29
1.51
4.25
1.00
2.15
1.14
1.31
1.24
1.19
1.02
1.23
2.36
1.00
0.98
8.5 8.0 7.5 7.0 6.5 6.0 5.5 5.0 4.5 4.0 3.5 3.0 2.5 2.0 1.5 1.0 0.5 0.0 -0
f1 (ppm)

S19

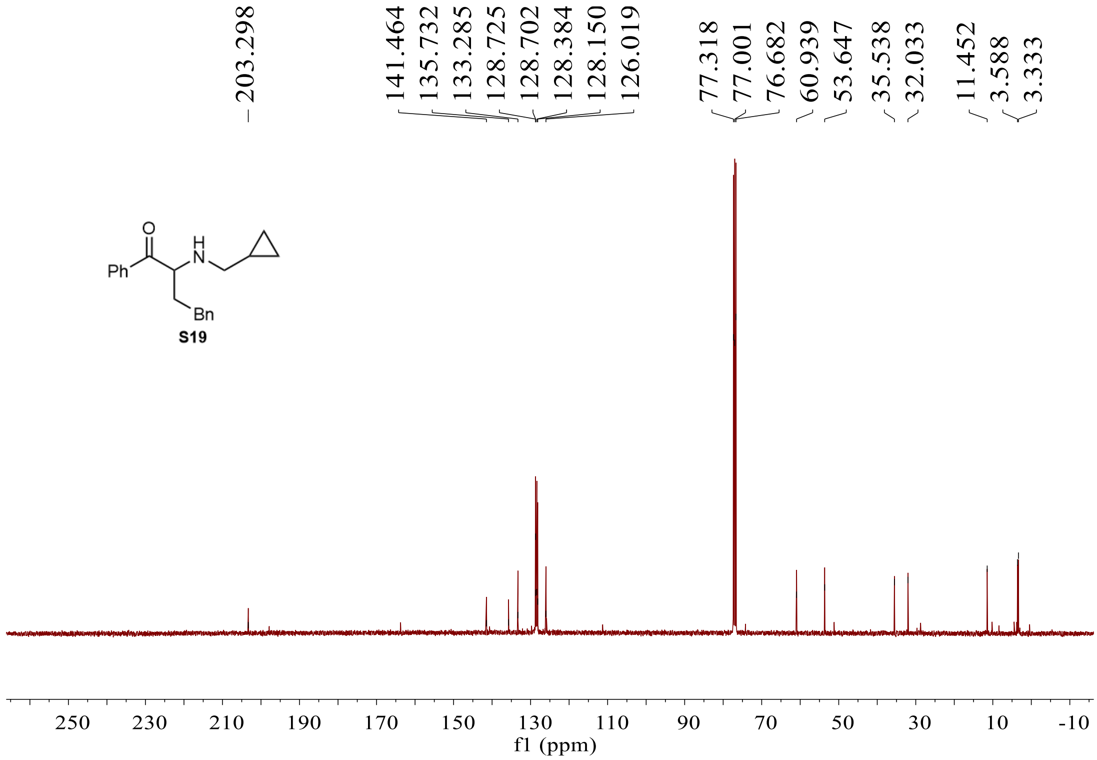
203.298
141.464
135.732
133.285
128.725
128.702
128.384
128.150
126.019
77.318
77.001
76.682
60.939
53.647
35.538
32.033
11.452
3.588
3.333
O
H
N
Ph
Bn
S19
250 230 210 190 170 150 130 110 90 70 50 30 10 -10
f1 (ppm)

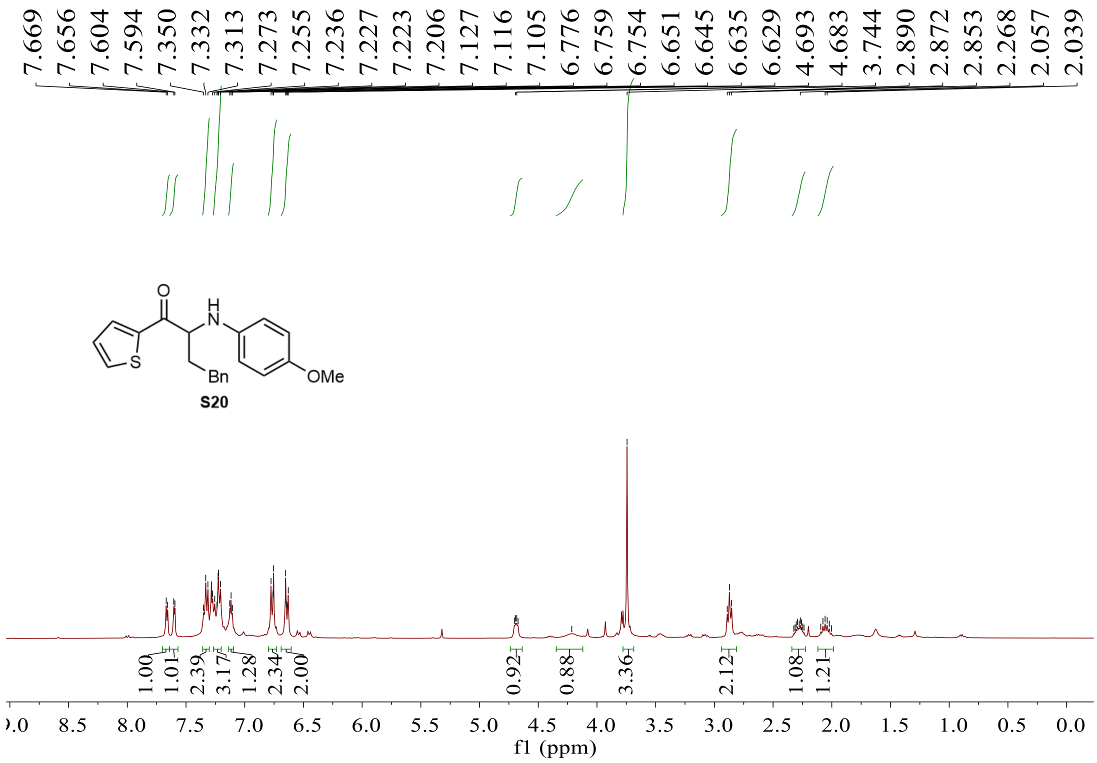
7.669
7.656
7.604
7.594
7.350
7.332
7.313
7.273
7.255
7.236
7.227
7.223
7.206
7.127
7.116
7.105
6.776
6.759
6.754
6.651
6.645
6.635
6.629
4.693
4.683
3.744
2.890
2.872
2.853
2.268
2.057
2.039
O
H
N
S
Bn
OMe
S20
1.00
1.01
2.39
3.17
1.28
2.34
2.00
0.92
0.88
3.36
2.12
1.08
1.21
8.5 8.0 7.5 7.0 6.5 6.0 5.5 5.0 4.5 4.0 3.5 3.0 2.5 2.0 1.5 1.0 0.5 0.0
f1 (ppm)

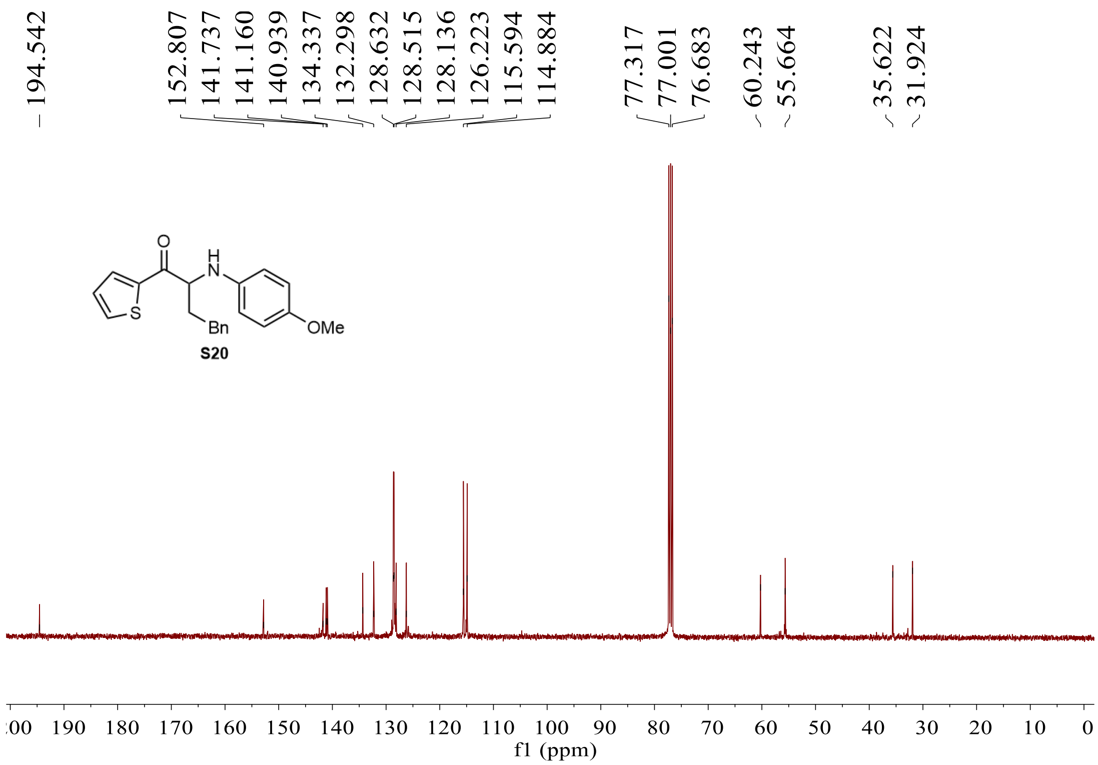
194.542
152.807
141.737
141.160
140.939
134.337
132.298
128.632
128.515
128.136
126.223
115.594
114.884
77.317
77.001
76.683
60.243
55.664
35.622
31.924
O
H
N
S
Bn
OMe
S20
f1 (ppm)

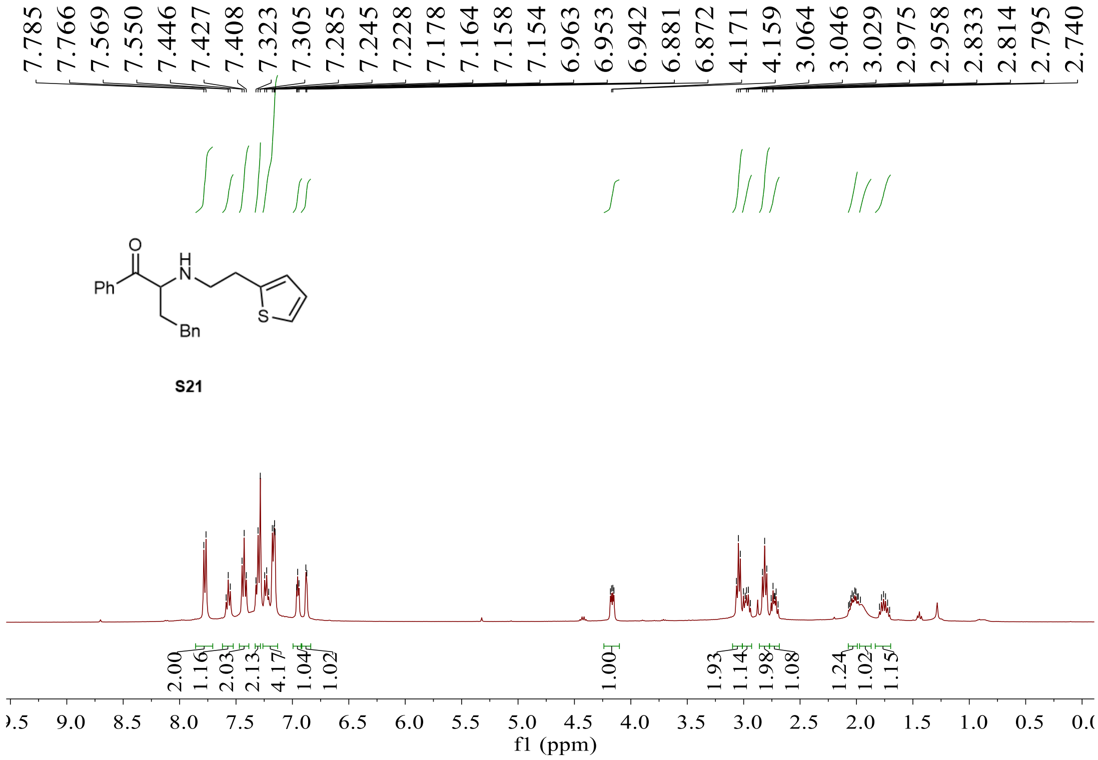
7.785
7.766
7.569
7.550
7.446
7.427
7.408
7.323
7.305
7.285
7.245
7.228
7.178
7.164
7.158
7.154
6.963
6.953
6.942
6.881
6.872
4.171
4.159
3.064
3.046
3.029
2.975
2.958
2.833
2.814
2.795
2.740
O
H
N
Ph
S
Bn
S21
2.00
1.16
2.03
2.13
4.17
1.04
1.02
1.00
1.93
1.14
1.98
1.08
1.24
1.02
1.15
f1 (ppm)

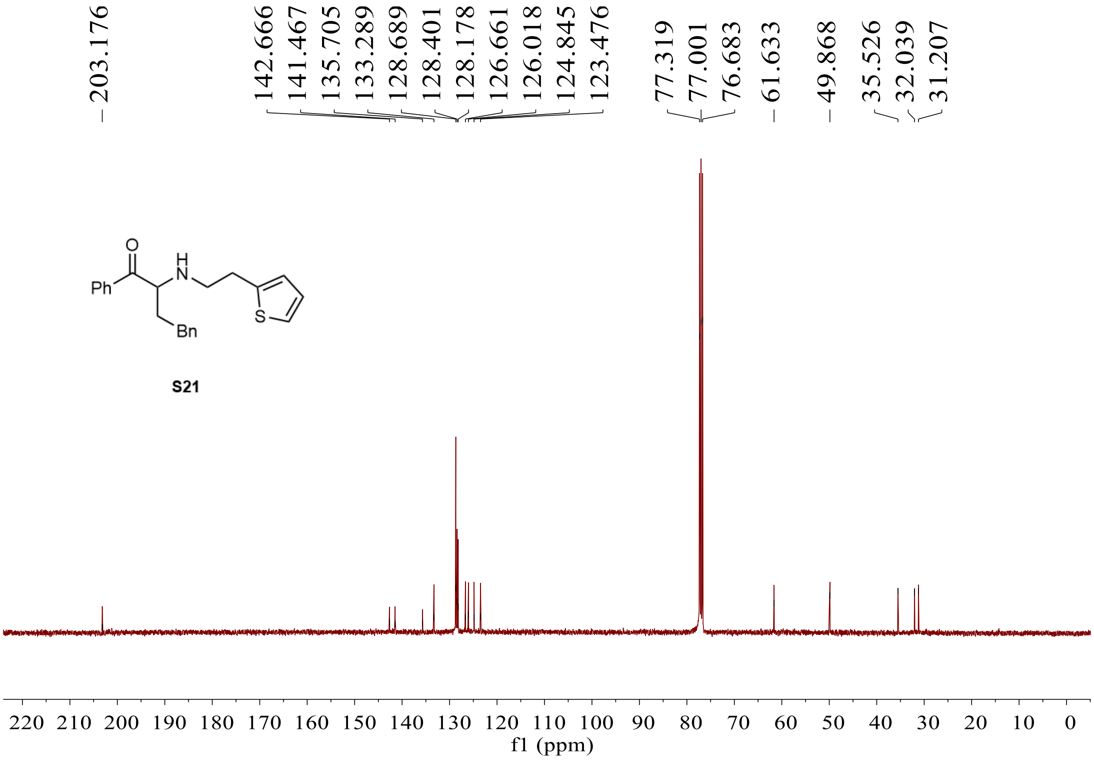

203.176
142.666
141.467
135.705
133.289
128.689
128.401
128.178
126.661
126.018
124.845
123.476
77.319
77.001
76.683
61.633
49.868
35.526
32.039
31.207
Ph
O
H
N
S
Bn
S21
220 210 200 190 180 170 160 150 140 130 120 110 100 90 80 70 60 50 40 30 20 10 0
f1 (ppm)

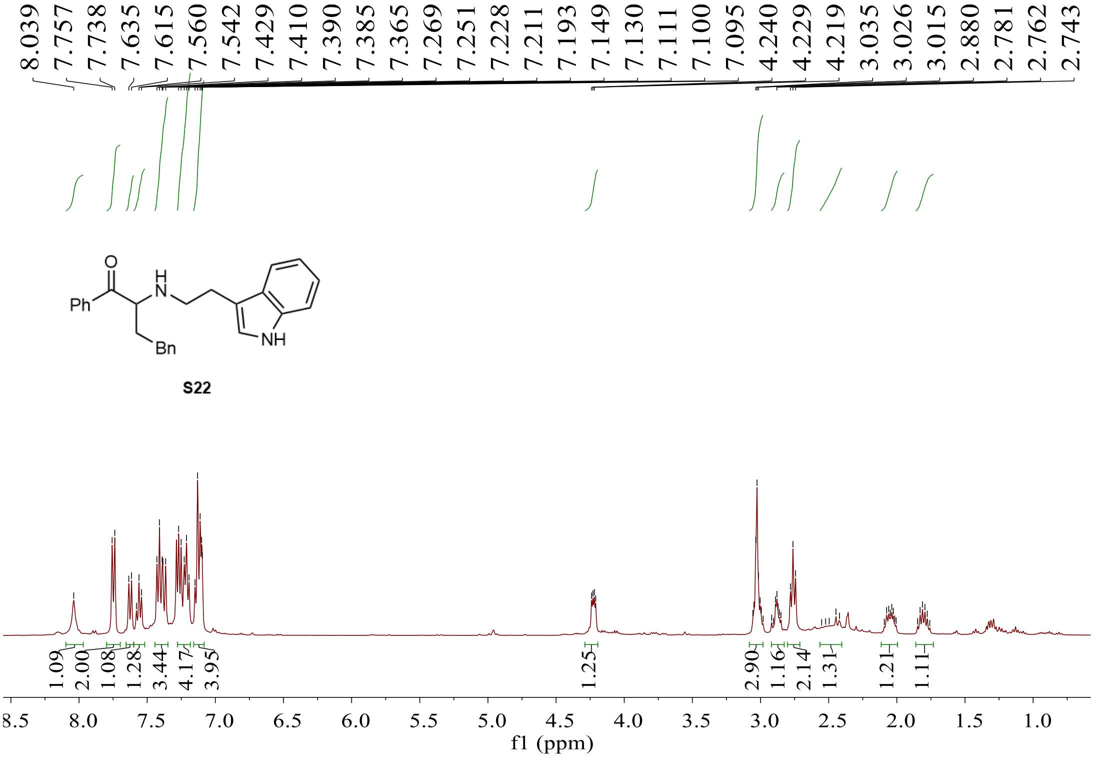

8.039
7.757
7.738
7.635
7.615
7.560
7.542
7.429
7.410
7.390
7.385
7.365
7.269
7.251
7.228
7.211
7.193
7.149
7.130
7.111
7.100
7.095
4.240
4.229
4.219
3.035
3.026
3.015
2.880
2.781
2.762
2.743
Ph
O
H
N
NH
Bn
S22
1.09
2.00
1.08
1.28
3.44
4.17
3.95
1.25
2.90
1.16
2.14
1.31
1.21
1.11
8.5 8.0 7.5 7.0 6.5 6.0 5.5 5.0 4.5 4.0 3.5 3.0 2.5 2.0 1.5 1.0
f1 (ppm)

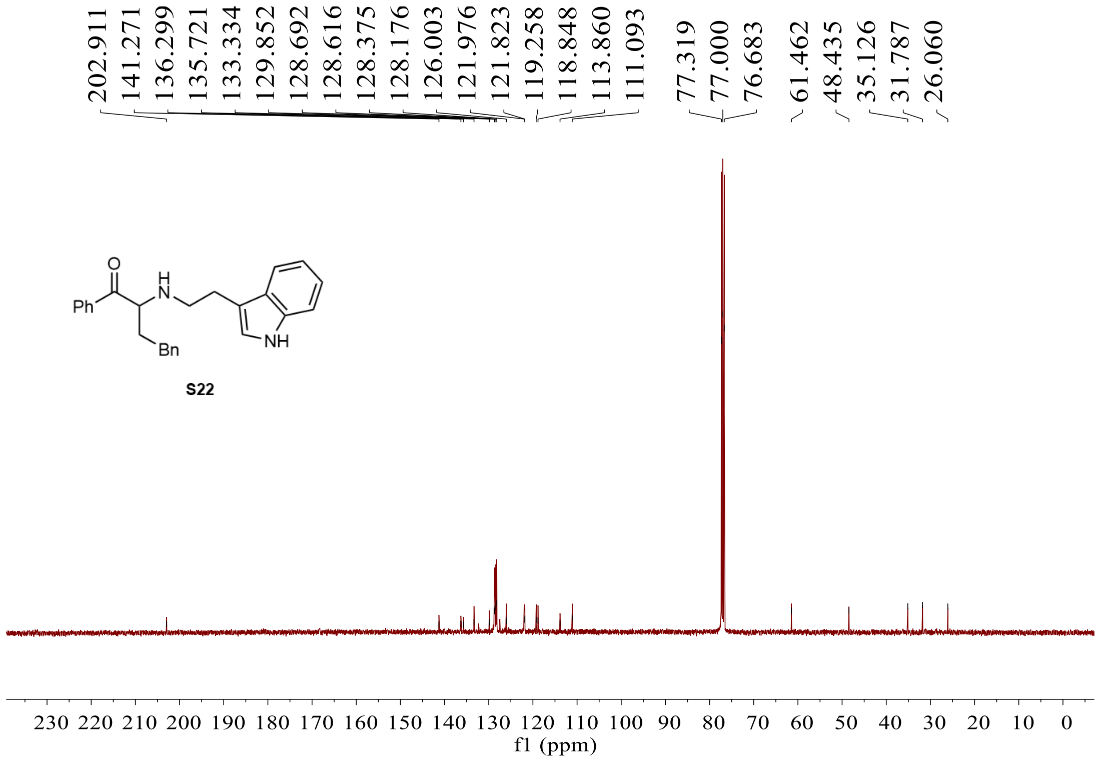
202.911
141.271
136.299
135.721
133.334
129.852
128.692
128.616
128.375
128.176
126.003
121.976
121.823
119.258
118.848
113.860
111.093
77.319
77.000
76.683
61.462
48.435
35.126
31.787
26.060
Ph
O
H
N
NH
Bn
S22
230 220 210 200 190 180 170 160 150 140 130 120 110 100 90 80 70 60 50 40 30 20 10 0
f1 (ppm)

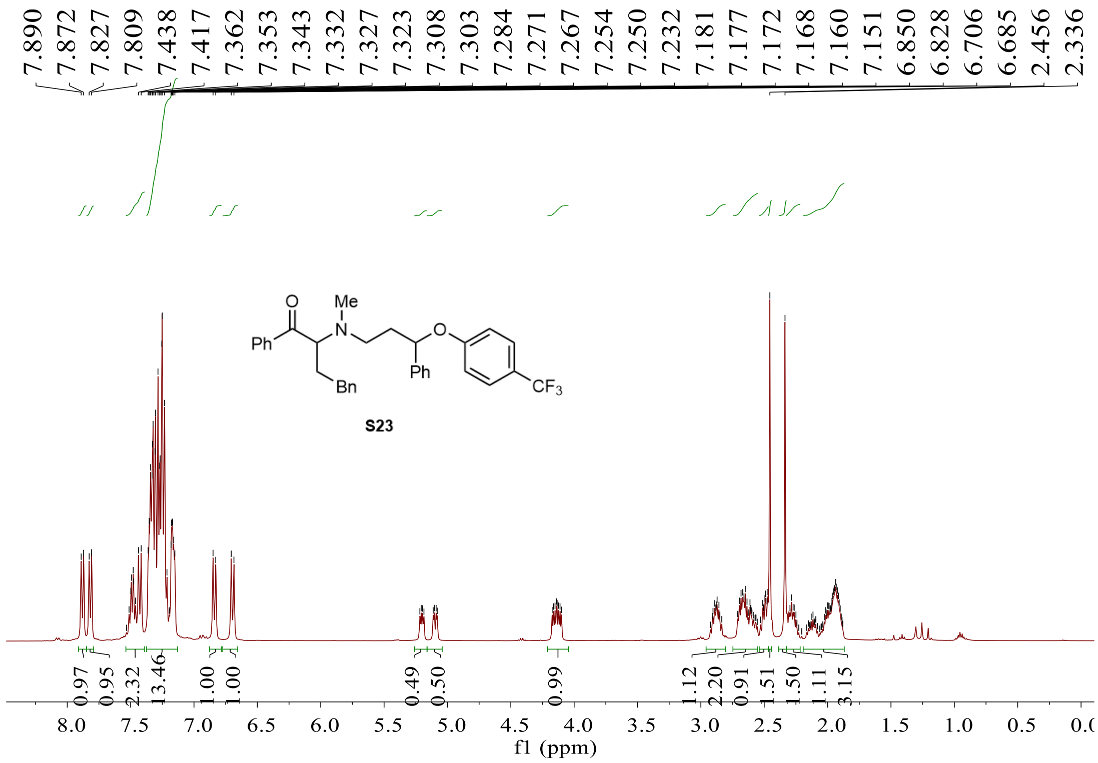
7.890
7.872
7.827
7.809
7.438
7.417
7.362
7.353
7.343
7.332
7.327
7.323
7.308
7.303
7.284
7.271
7.267
7.254
7.250
7.232
7.181
7.177
7.172
7.168
7.160
7.151
6.850
6.828
6.706
6.685
2.456
2.336
Ph
O
Me
N
O
Ph
Bn
CF3
S23
0.97
0.95
2.32
13.46
1.00
1.00
0.49
0.50
0.99
1.12
2.20
0.91
1.51
1.50
1.11
3.15
8.0 7.5 7.0 6.5 6.0 5.5 5.0 4.5 4.0 3.5 3.0 2.5 2.0 1.5 1.0 0.5 0.0
f1 (ppm)

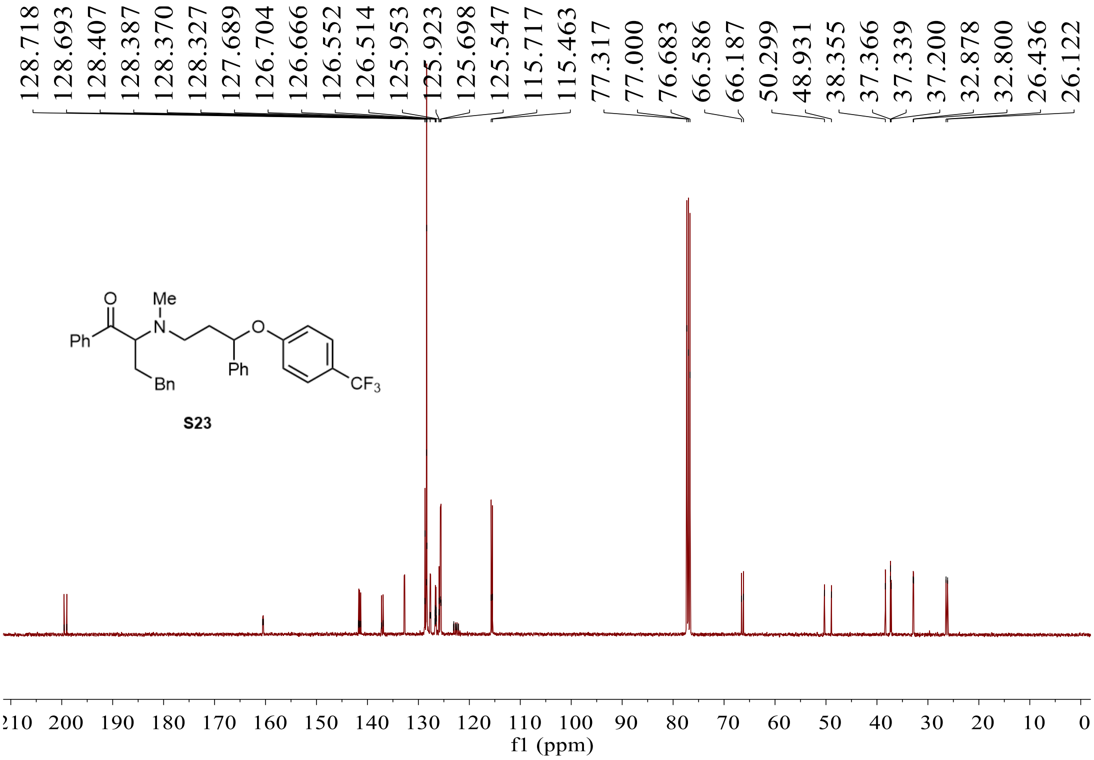
128.718
128.693
128.407
128.387
128.370
128.327
127.689
126.704
126.666
126.552
126.514
125.953
125.923
125.698
125.547
115.717
115.463
77.317
77.000
76.683
66.586
66.187
50.299
48.931
38.355
37.366
37.339
37.200
32.878
32.800
26.436
26.122
O
Me
N
O
Ph
Ph
Bn
CF3
S23
210 200 190 180 170 160 150 140 130 120 110 100 90 80 70 60 50 40 30 20 10 0
f1 (ppm)

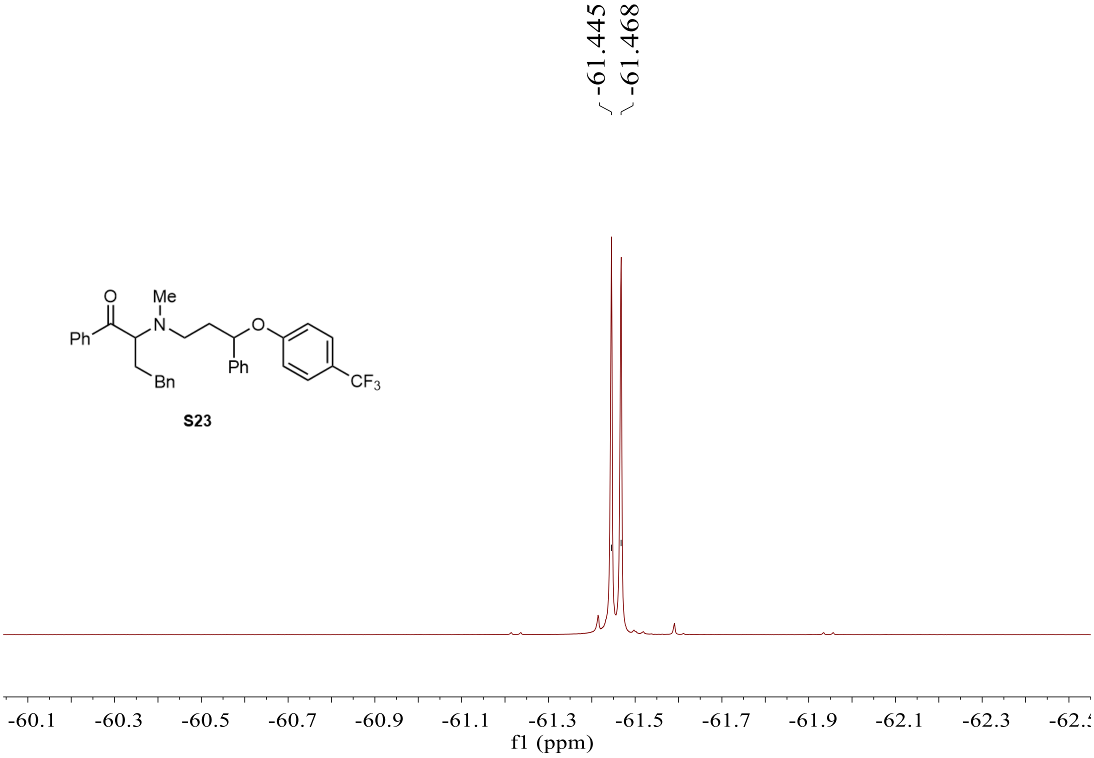
-61.445
-61.468
O
Me
N
O
Ph
Ph
Bn
CF3
S23
-60.1 -60.3 -60.5 -60.7 -60.9 -61.1 -61.3 -61.5 -61.7 -61.9 -62.1 -62.3
f1 (ppm)

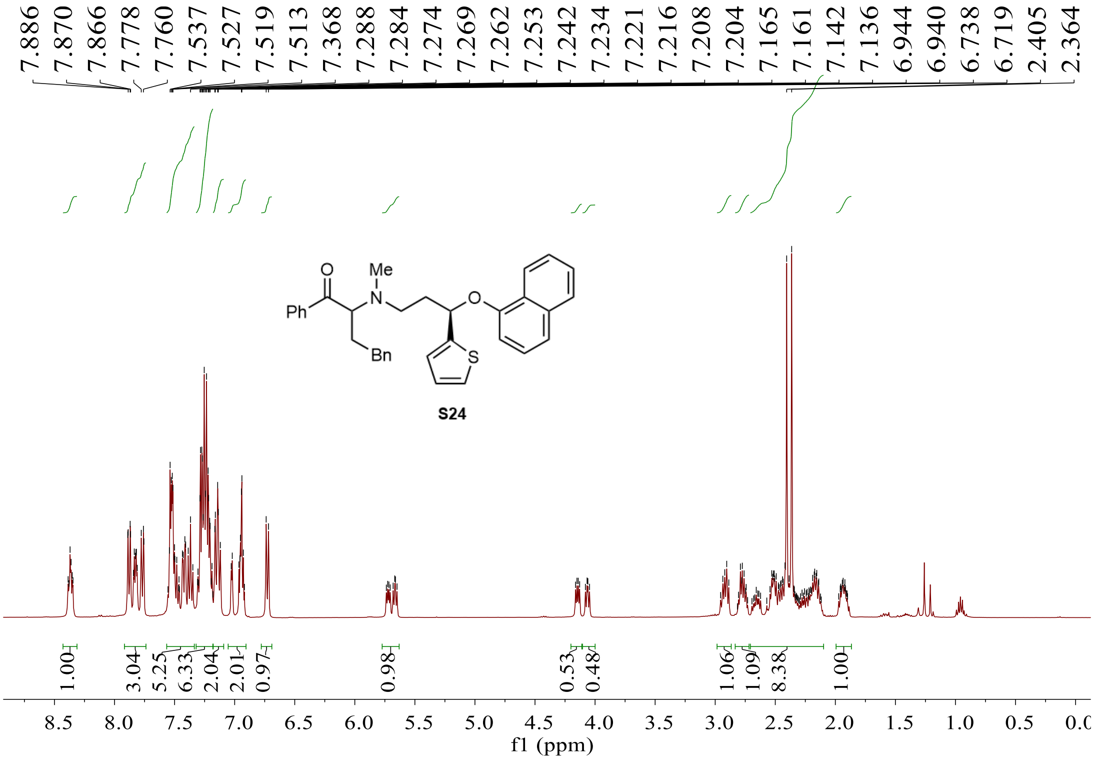
7.886 7.870 7.866 7.778 7.760 7.537 7.527 7.519 7.513 7.368 7.288 7.284 7.274 7.269 7.262 7.253 7.242 7.234 7.221 7.216 7.208 7.204 7.165 7.161 7.142 7.136 6.944 6.940 6.738 6.719 2.405 2.364
O
Me
Ph
N
O
Bn
S
S24
1.00 3.04 5.25 6.33 2.04 2.01 0.97 0.98 0.53 0.48 1.06 1.09 8.38 1.00
8.5 8.0 7.5 7.0 6.5 6.0 5.5 5.0 4.5 4.0 3.5 3.0 2.5 2.0 1.5 1.0 0.5 0.0
f1 (ppm)

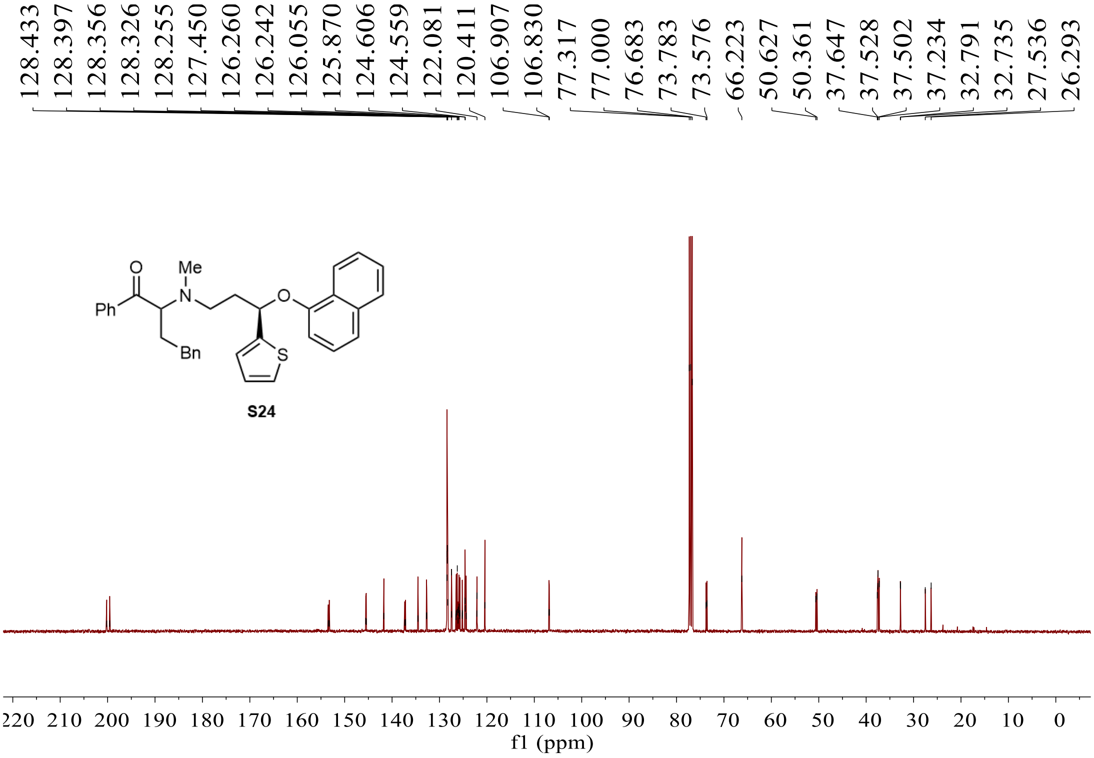
128.433 128.397 128.356 128.326 128.255 127.450 126.260 126.242 126.055 125.870 124.606 124.559 122.081 120.411 106.907 106.830 77.317 77.000 76.683 73.783 73.576 66.223 50.627 50.361 37.647 37.528 37.502 37.234 32.791 32.735 27.536 26.293
O
Me
Ph
N
O
Bn
S
S24
220 210 200 190 180 170 160 150 140 130 120 110 100 90 80 70 60 50 40 30 20 10 0
f1 (ppm)

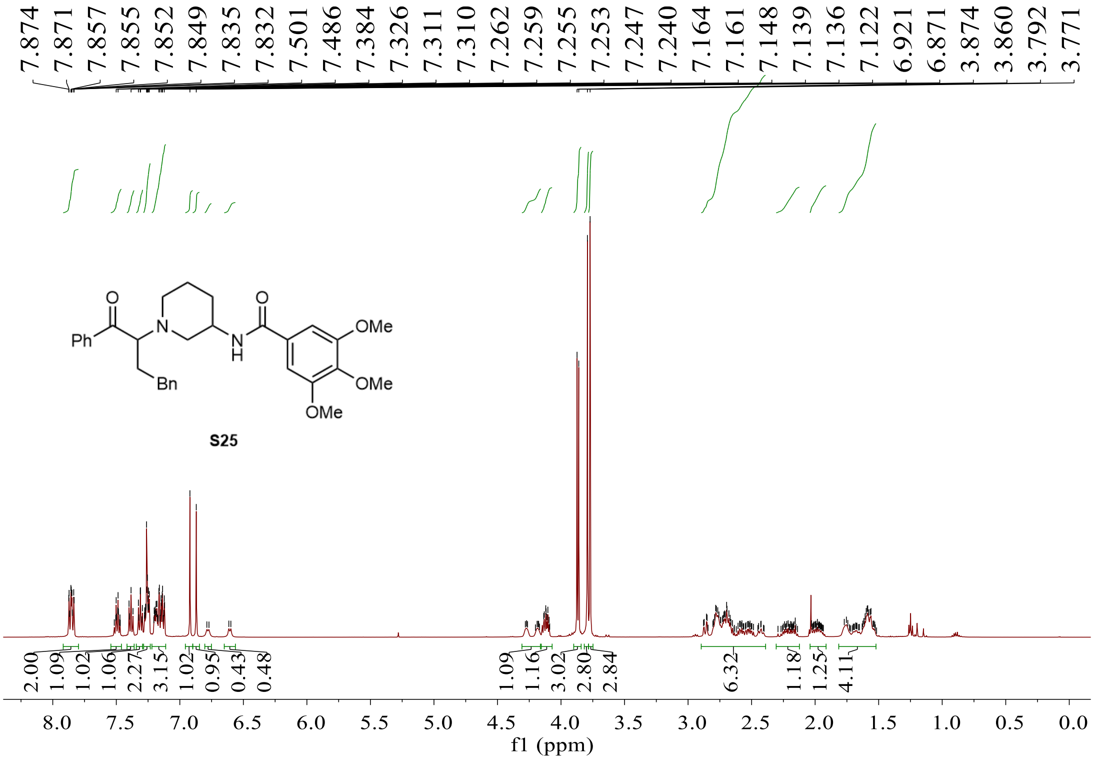
Ph
N
N
H
OMe
OMe
OMe
Bn
S25
f1 (ppm)

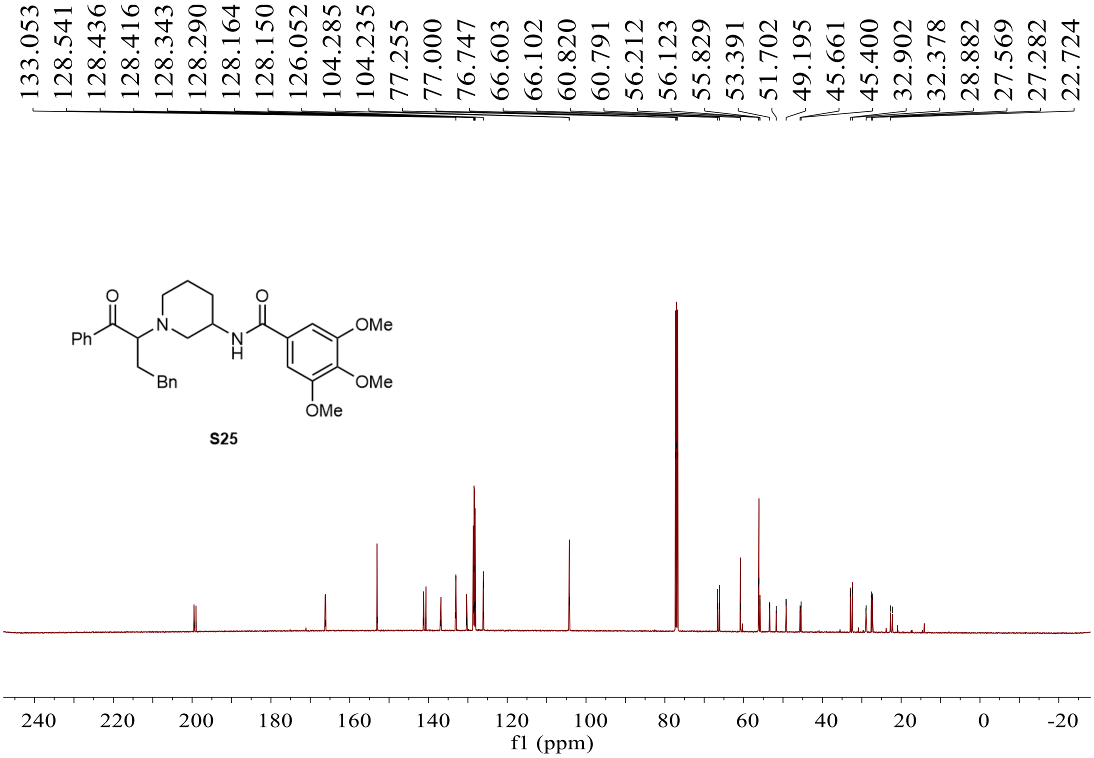
Ph
N
N
H
OMe
OMe
OMe
Bn
S25
f1 (ppm)

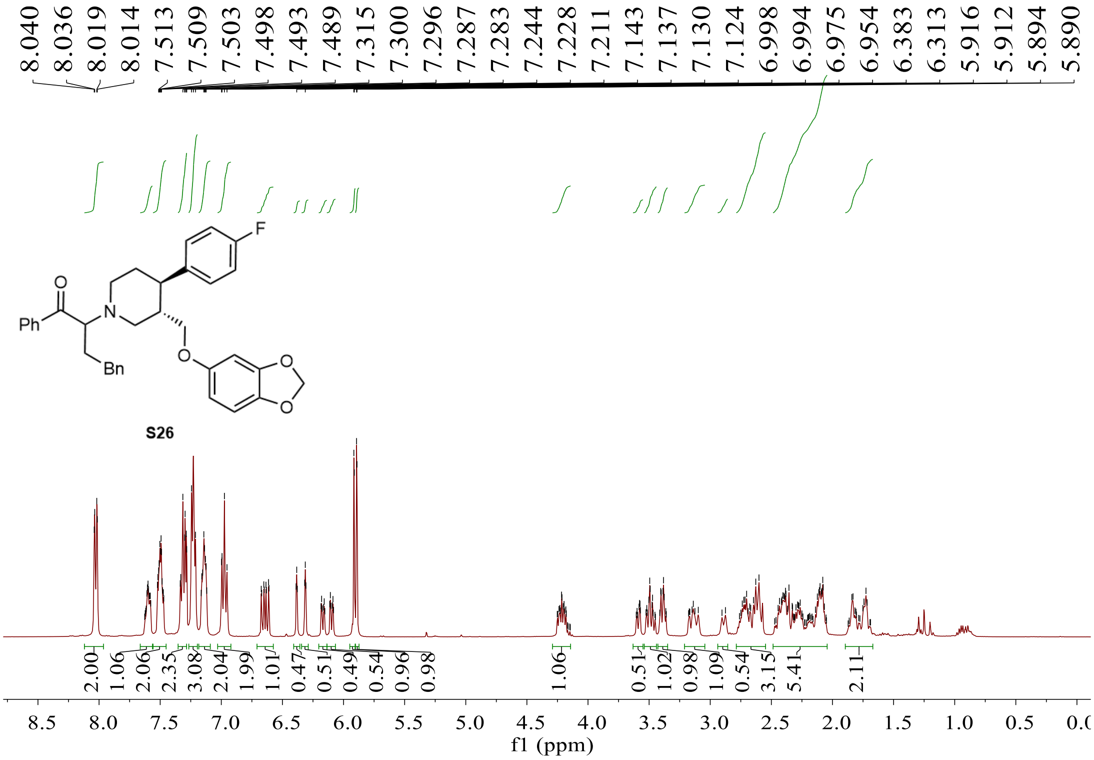
8.040 8.036 8.019 8.014 7.513 7.509 7.503 7.498 7.493 7.489 7.315 7.300 7.296 7.287 7.283 7.244 7.228 7.211 7.143 7.137 7.130 7.124 6.998 6.994 6.975 6.954 6.383 6.313 5.916 5.912 5.894 5.890
F
O
Ph
N
O
Bn
O
O
S26
2.00 1.06 2.06 2.35 3.08 2.04 1.99 1.01 0.47 0.51 0.49 0.54 0.96 0.98 1.06 0.51 1.02 0.98 1.09 0.54 3.15 5.41 2.11
8.5 8.0 7.5 7.0 6.5 6.0 5.5 5.0 4.5 4.0 3.5 3.0 2.5 2.0 1.5 1.0 0.5 0.0
f1 (ppm)

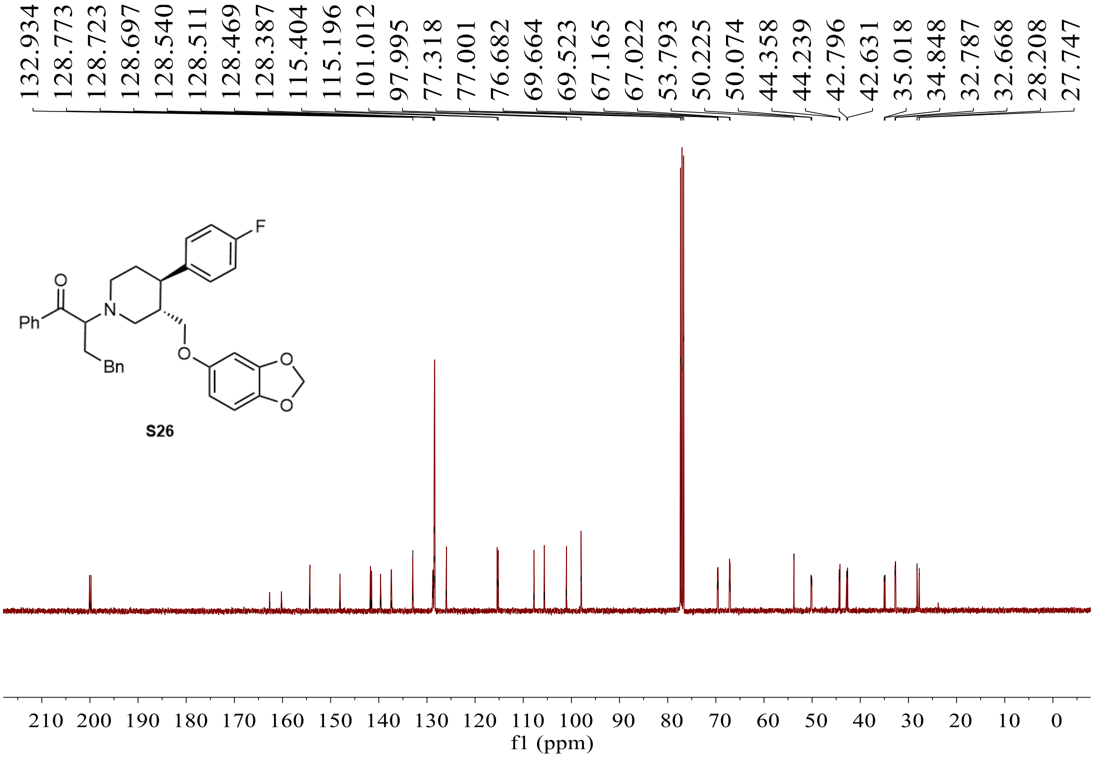
132.934 128.773 128.723 128.697 128.540 128.511 128.469 128.387 115.404 115.196 101.012 97.995 77.318 77.001 76.682 69.664 69.523 67.165 67.022 53.793 50.225 50.074 44.358 44.239 42.796 42.631 35.018 34.848 32.787 32.668 28.208 27.747
F
O
Ph
N
O
Bn
O
O
S26
210 200 190 180 170 160 150 140 130 120 110 100 90 80 70 60 50 40 30 20 10 0
f1 (ppm)

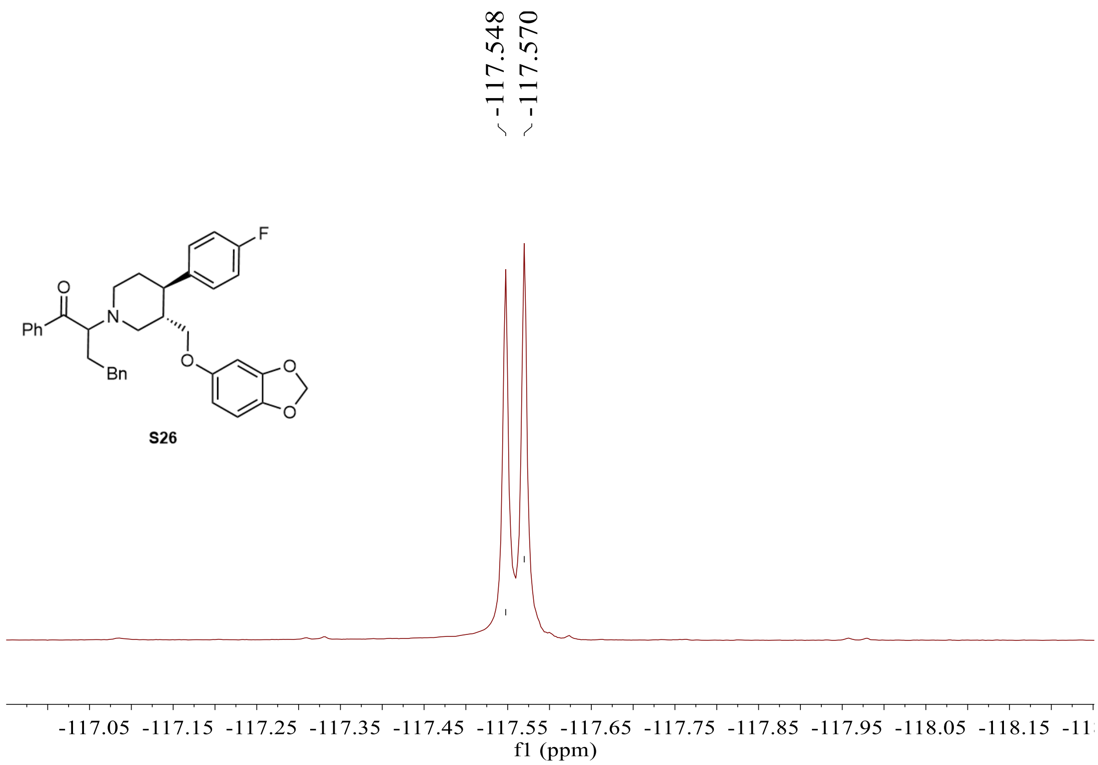

-117.548
-117.570
F
O
Ph
N
Bn
O
O
O
S26
-117.05 -117.15 -117.25 -117.35 -117.45 -117.55 -117.65 -117.75 -117.85 -117.95 -118.05 -118.15
f1 (ppm)

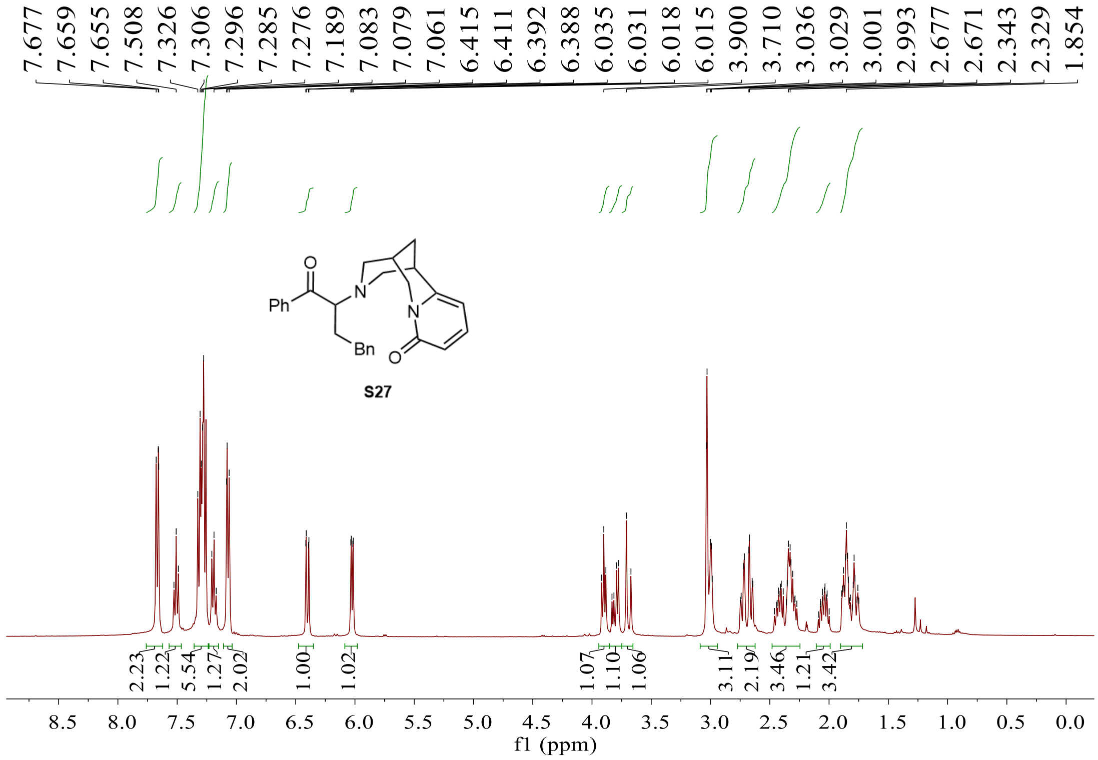

7.677 7.659 7.655 7.508 7.326 7.306 7.296 7.285 7.276 7.189 7.083 7.079 7.061 6.415 6.411 6.392 6.388 6.035 6.031 6.018 6.015 3.900 3.710 3.036 3.029 3.001 2.993 2.677 2.671 2.343 2.329 1.854
O
Ph
N
Bn
N
O
S27
2.23 1.22 5.54 1.27 2.02 1.00 1.02 1.07 1.10 1.06 3.11 2.19 3.46 1.21 3.42
8.5 8.0 7.5 7.0 6.5 6.0 5.5 5.0 4.5 4.0 3.5 3.0 2.5 2.0 1.5 1.0 0.5 0.0
f1 (ppm)

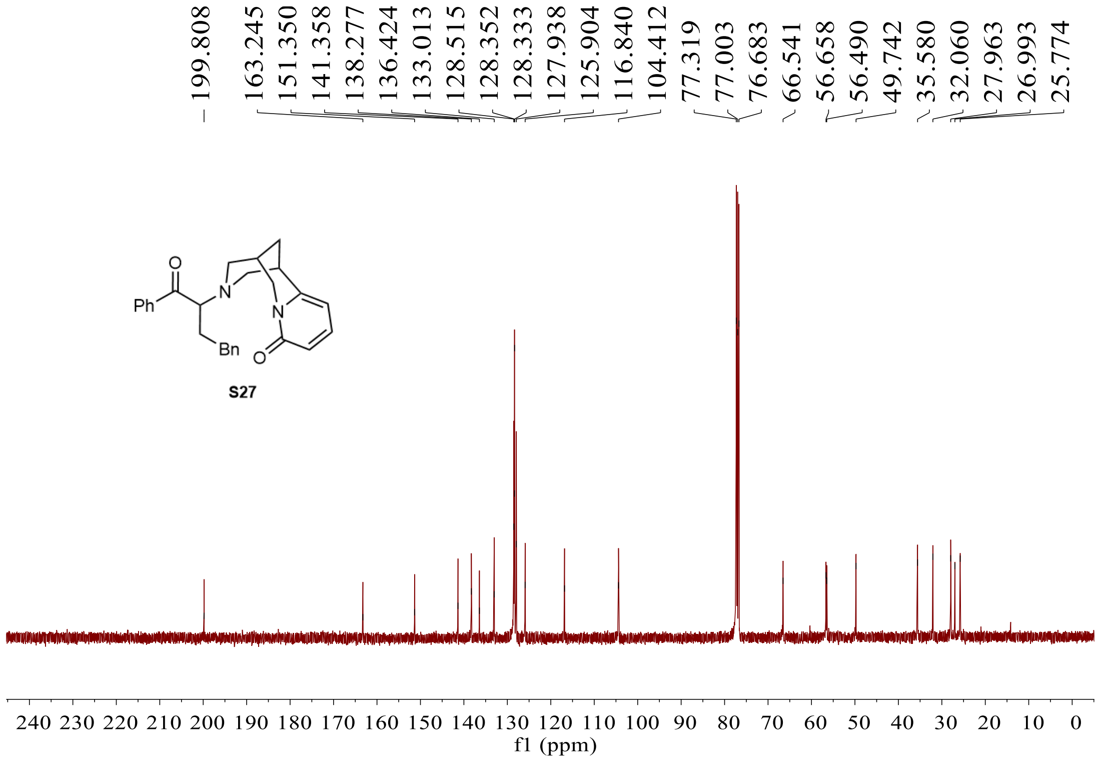

199.808
163.245
151.350
141.358
138.277
136.424
133.013
128.515
128.352
128.333
127.938
125.904
116.840
104.412
77.319
77.003
76.683
66.541
56.658
56.490
49.742
35.580
32.060
27.963
26.993
25.774
O
Ph
N
N
Bn
O
240 230 220 210 200 190 180 170 160 150 140 130 120 110 100 90 80 70 60 50 40 30 20 10 0
f1 (ppm)

S27

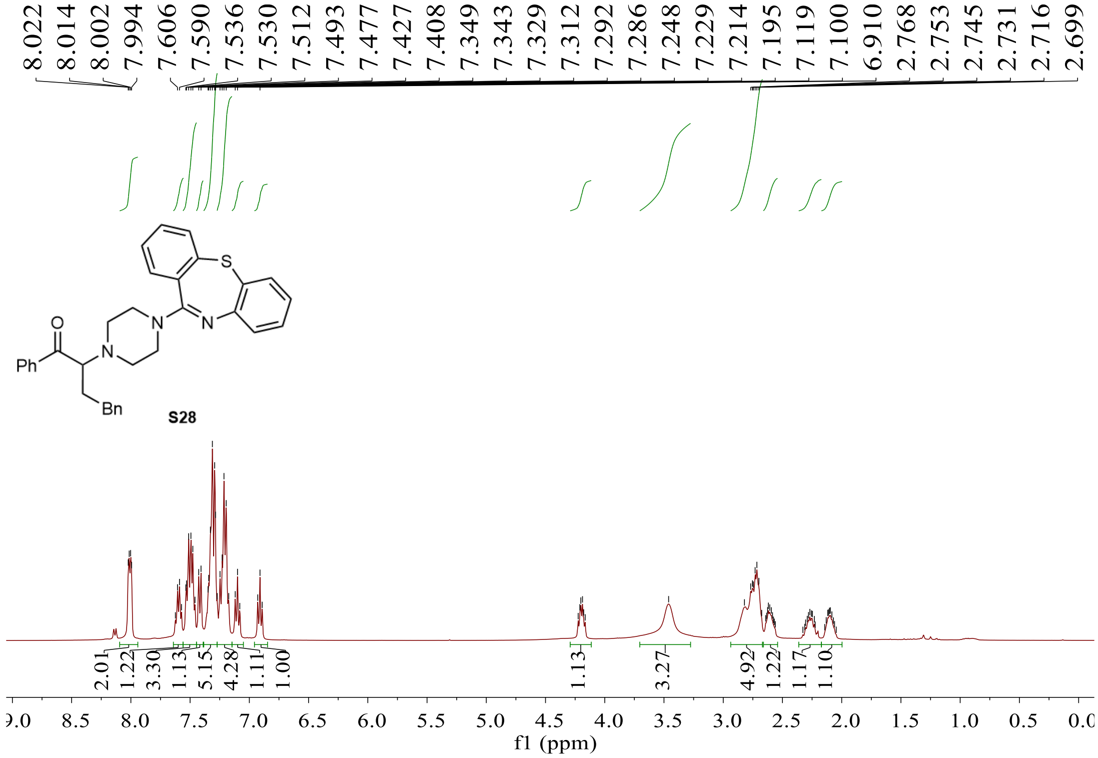

8.022
8.014
8.002
7.994
7.606
7.590
7.536
7.530
7.512
7.493
7.477
7.427
7.408
7.349
7.343
7.329
7.312
7.292
7.286
7.248
7.229
7.214
7.195
7.119
7.100
6.910
2.768
2.753
2.745
2.731
2.716
2.699
S
N
N
N
O
Ph
Bn
2.01
1.22
3.30
1.13
5.15
4.28
1.11
1.00
1.13
3.27
4.92
1.22
1.17
1.10
9.0 8.5 8.0 7.5 7.0 6.5 6.0 5.5 5.0 4.5 4.0 3.5 3.0 2.5 2.0 1.5 1.0 0.5 0.0
f1 (ppm)

S28

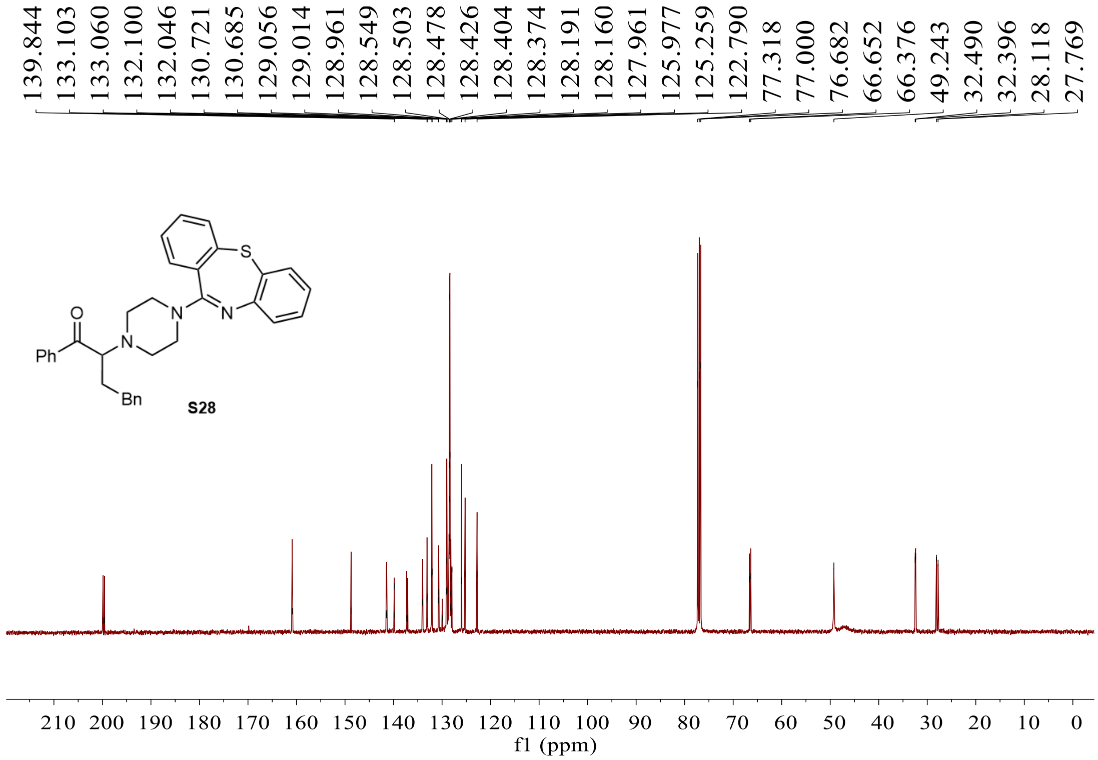
139.844
133.103
133.060
132.100
132.046
130.721
130.685
129.056
129.014
128.961
128.549
128.503
128.478
128.426
128.404
128.374
128.191
128.160
127.961
125.977
125.259
122.790
77.318
77.000
76.682
66.652
66.376
49.243
32.490
32.396
28.118
27.769
S
N
N
N
O
Ph
Bn
S28
210 200 190 180 170 160 150 140 130 120 110 100 90 80 70 60 50 40 30 20 10 0
f1 (ppm)

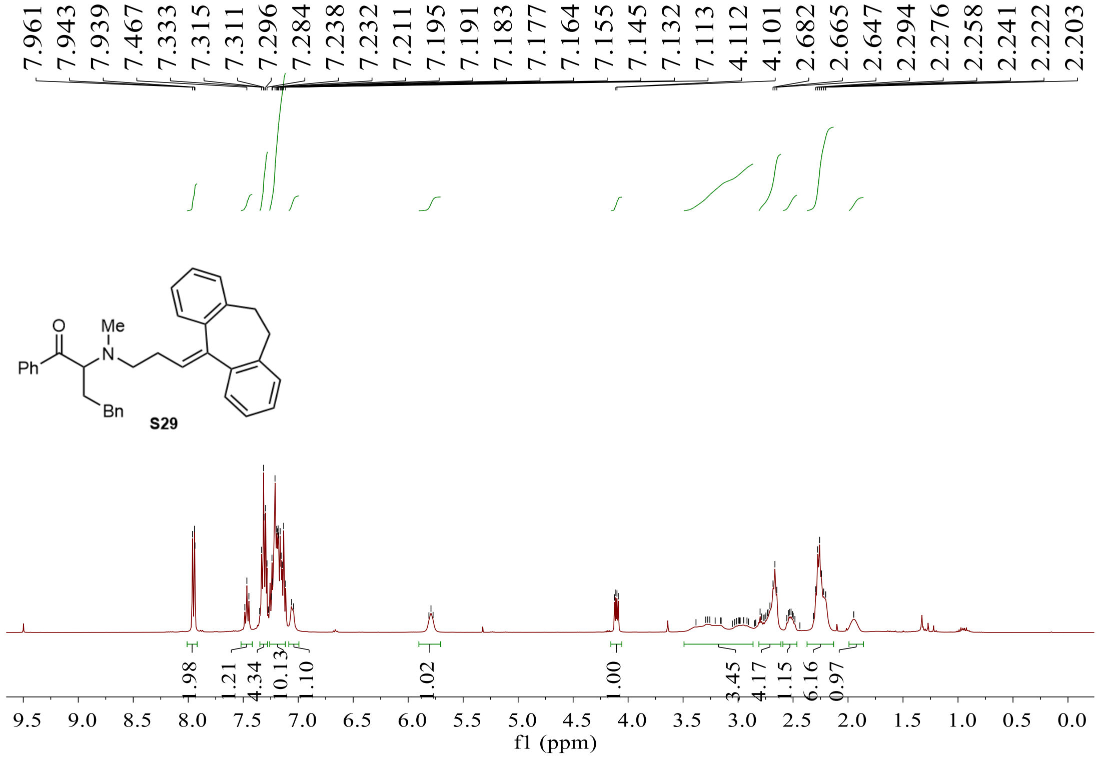
7.961
7.943
7.939
7.467
7.333
7.315
7.311
7.296
7.284
7.238
7.232
7.211
7.195
7.191
7.183
7.177
7.164
7.155
7.145
7.132
7.113
4.112
4.101
2.682
2.665
2.647
2.294
2.276
2.258
2.241
2.222
2.203
O
Me
N
Ph
Bn
S29
1.98
1.21
4.34
10.13
1.10
1.02
1.00
3.45
4.17
1.15
6.16
0.97
9.5 9.0 8.5 8.0 7.5 7.0 6.5 6.0 5.5 5.0 4.5 4.0 3.5 3.0 2.5 2.0 1.5 1.0 0.5 0.0
f1 (ppm)

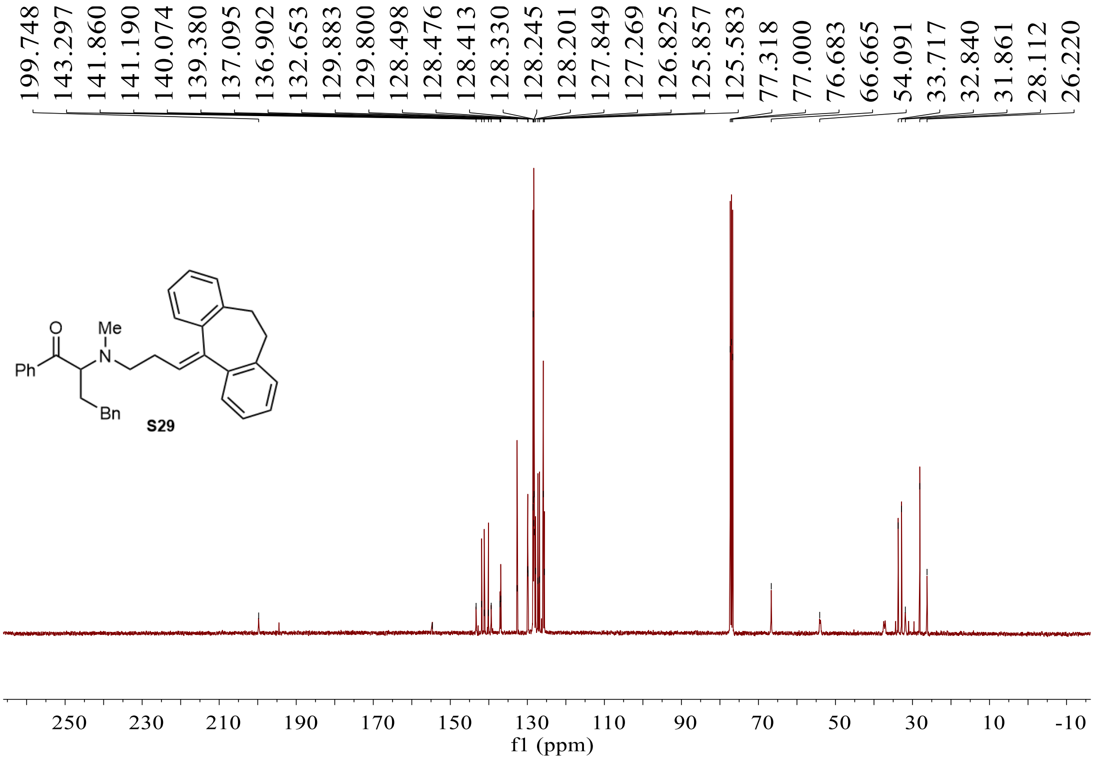
199.748 143.297 141.860 141.190 140.074 139.380 137.095 136.902 132.653 129.883 129.800 128.498 128.476 128.413 128.330 128.245 128.201 127.849 127.269 126.825 125.857 125.583 77.318 77.000 76.683 66.665 54.091 33.717 32.840 31.861 28.112 26.220
O
Me
N
Ph
Bn
S29
250 230 210 190 170 150 130 110 90 70 50 30 10 -10
f1 (ppm)

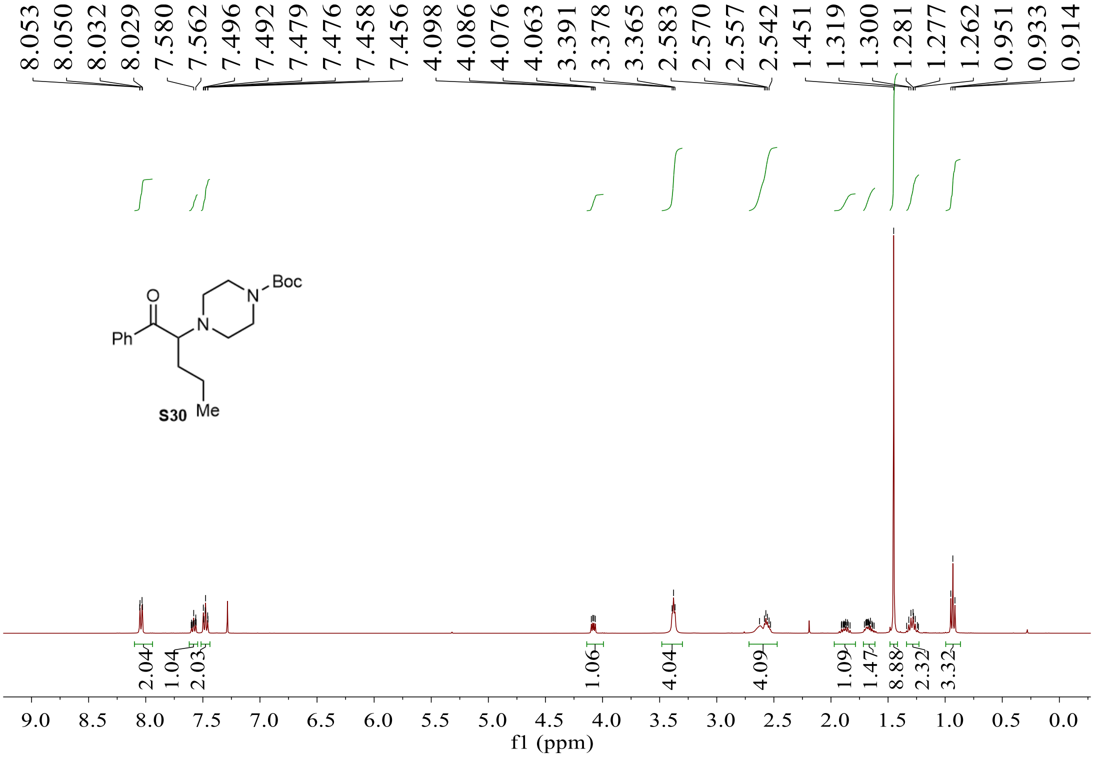
8.053 8.050 8.032 8.029 7.580 7.562 7.496 7.492 7.479 7.476 7.458 7.456 4.098 4.086 4.076 4.063 3.391 3.378 3.365 2.583 2.570 2.557 2.542 1.451 1.319 1.300 1.281 1.277 1.262 0.951 0.933 0.914
O
Boc
N
N
Ph
S30
Me
2.04 1.04 2.03 1.06 4.04 4.09 1.09 1.47 8.88 2.32 3.32
9.0 8.5 8.0 7.5 7.0 6.5 6.0 5.5 5.0 4.5 4.0 3.5 3.0 2.5 2.0 1.5 1.0 0.5 0.0
f1 (ppm)

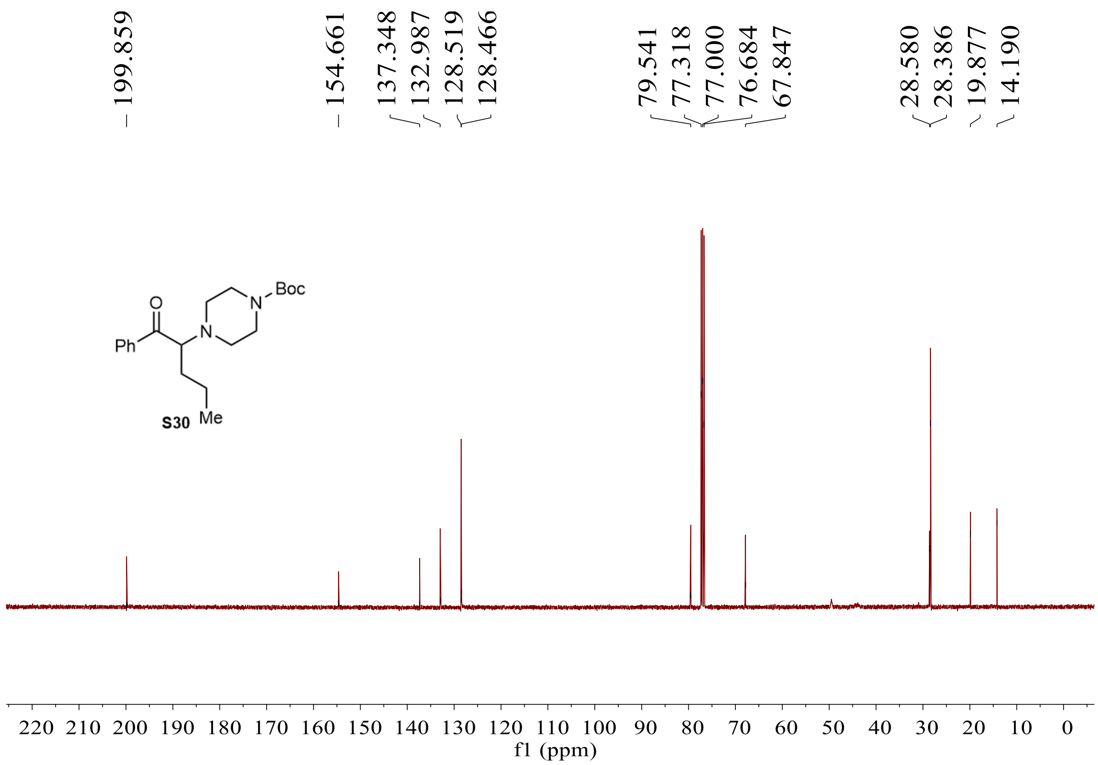
199.859
154.661
137.348
132.987
128.519
128.466
79.541
77.318
77.000
76.684
67.847
28.580
28.386
19.877
14.190
O
Ph
N
N
Boc
Me
S30
220 210 200 190 180 170 160 150 140 130 120 110 100 90 80 70 60 50 40 30 20 10 0
f1 (ppm)

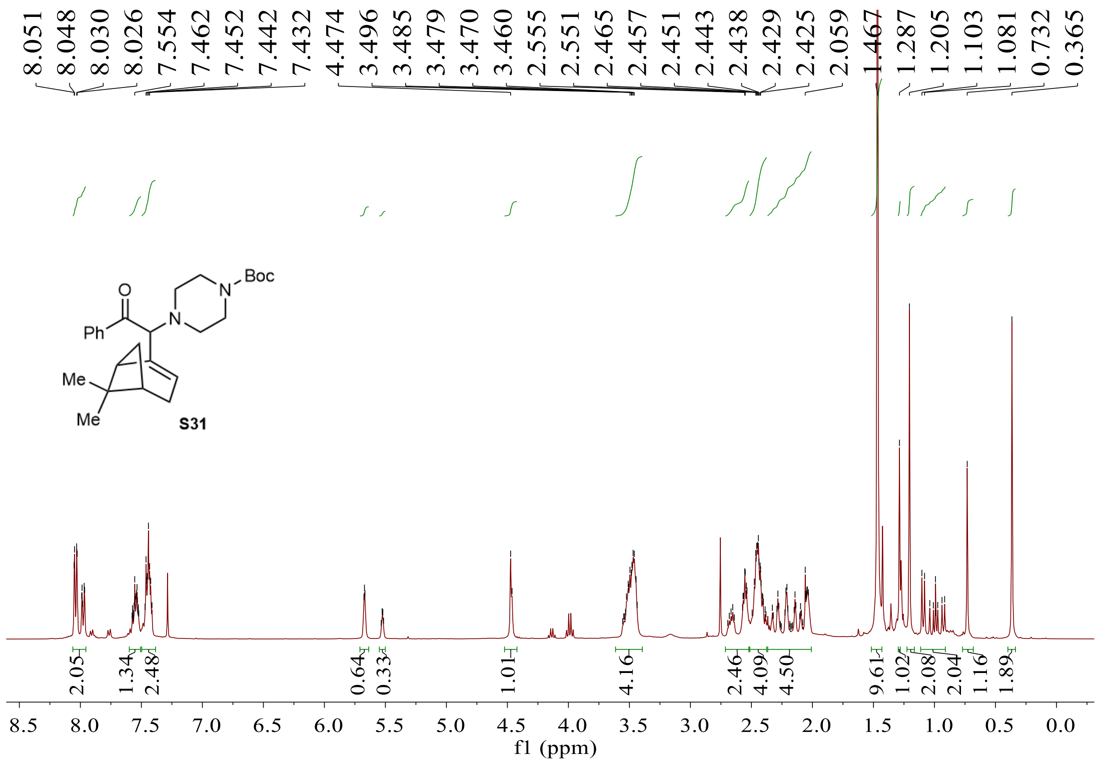
8.051
8.048
8.030
8.026
7.554
7.462
7.452
7.442
7.432
4.474
3.496
3.485
3.479
3.470
3.460
2.555
2.551
2.465
2.457
2.451
2.443
2.438
2.429
2.425
2.059
1.467
1.287
1.205
1.103
1.081
0.732
0.365
O
Ph
N
N
Boc
Me
Me
S31
2.05
1.34
2.48
0.64
0.33
1.01
4.16
2.46
4.09
4.50
9.61
1.02
2.08
2.04
1.16
1.89
8.5 8.0 7.5 7.0 6.5 6.0 5.5 5.0 4.5 4.0 3.5 3.0 2.5 2.0 1.5 1.0 0.5 0.0
f1 (ppm)

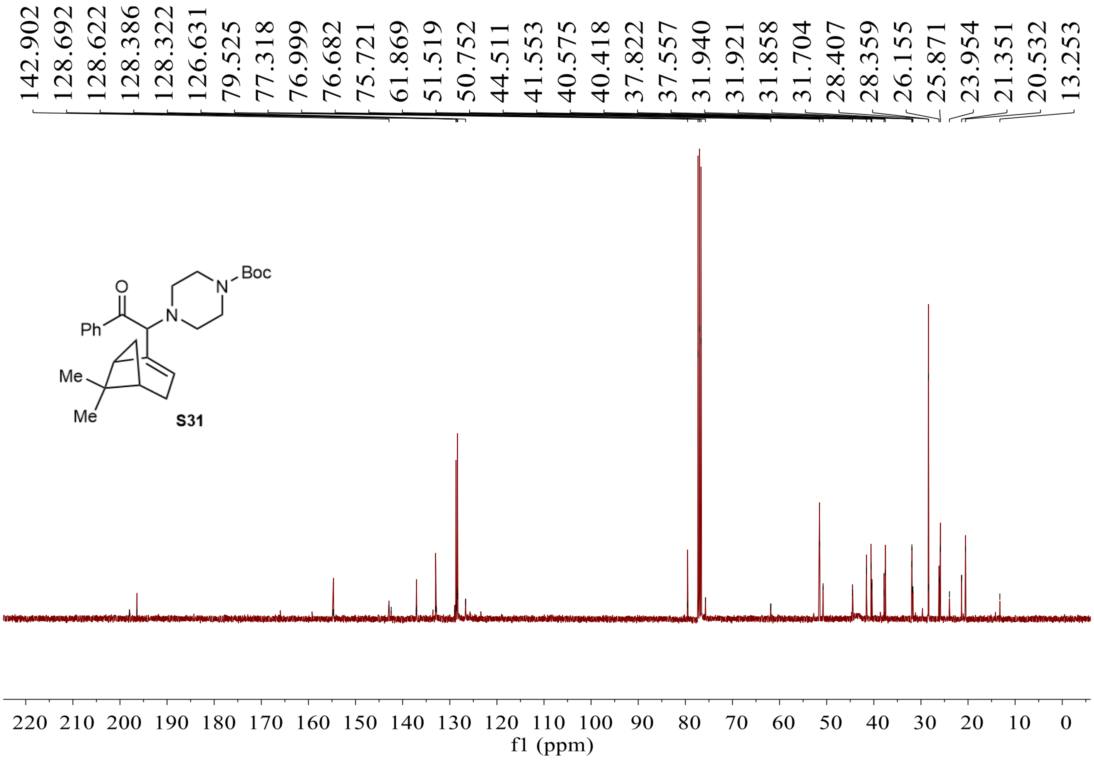
142.902
128.692
128.622
128.386
128.322
126.631
79.525
77.318
76.999
76.682
75.721
61.869
51.519
50.752
44.511
41.553
40.575
40.418
37.822
37.557
31.940
31.921
31.858
31.704
28.407
28.359
26.155
25.871
23.954
21.351
20.532
13.253
Boc
O
N
Ph
Me
Me
S31
220 210 200 190 180 170 160 150 140 130 120 110 100 90 80 70 60 50 40 30 20 10 0
f1 (ppm)

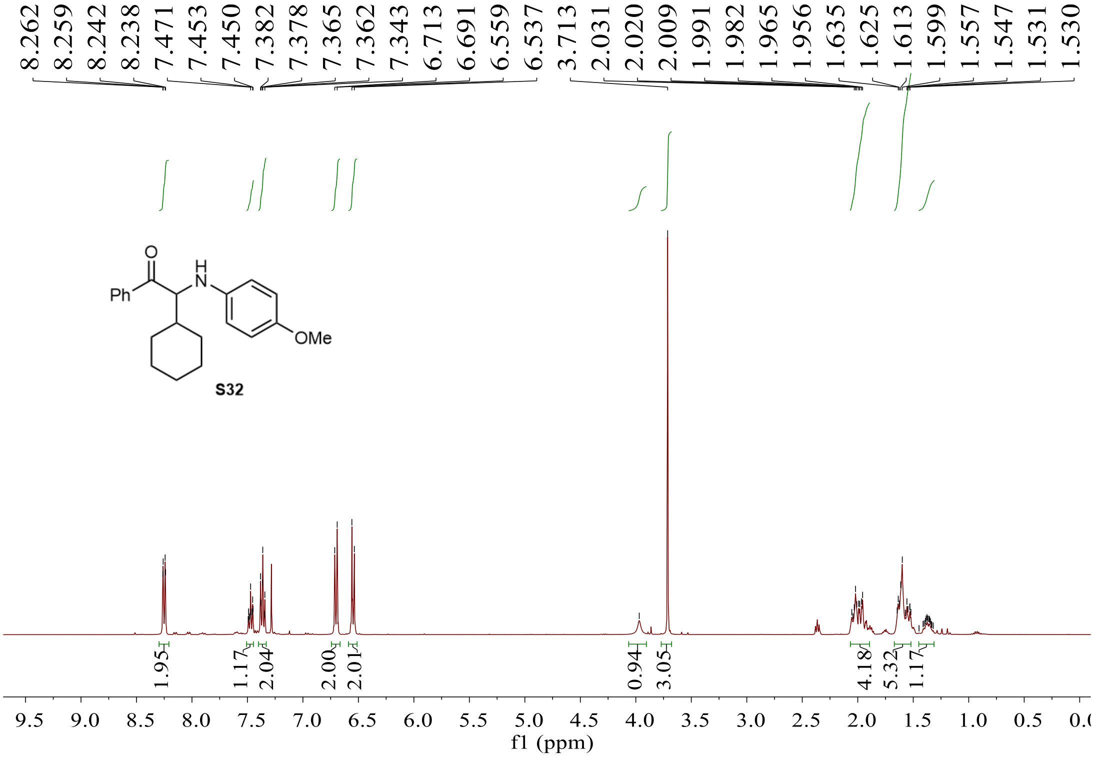
8.262
8.259
8.242
8.238
7.471
7.453
7.450
7.382
7.378
7.365
7.362
7.343
6.713
6.691
6.559
6.537
3.713
2.031
2.020
2.009
1.991
1.982
1.965
1.956
1.635
1.625
1.613
1.599
1.557
1.547
1.531
1.530
O
H
N
Ph
OMe
S32
1.95
1.17
2.04
2.00
2.01
0.94
3.05
4.18
5.32
1.17
9.5 9.0 8.5 8.0 7.5 7.0 6.5 6.0 5.5 5.0 4.5 4.0 3.5 3.0 2.5 2.0 1.5 1.0 0.5 0.0
f1 (ppm)

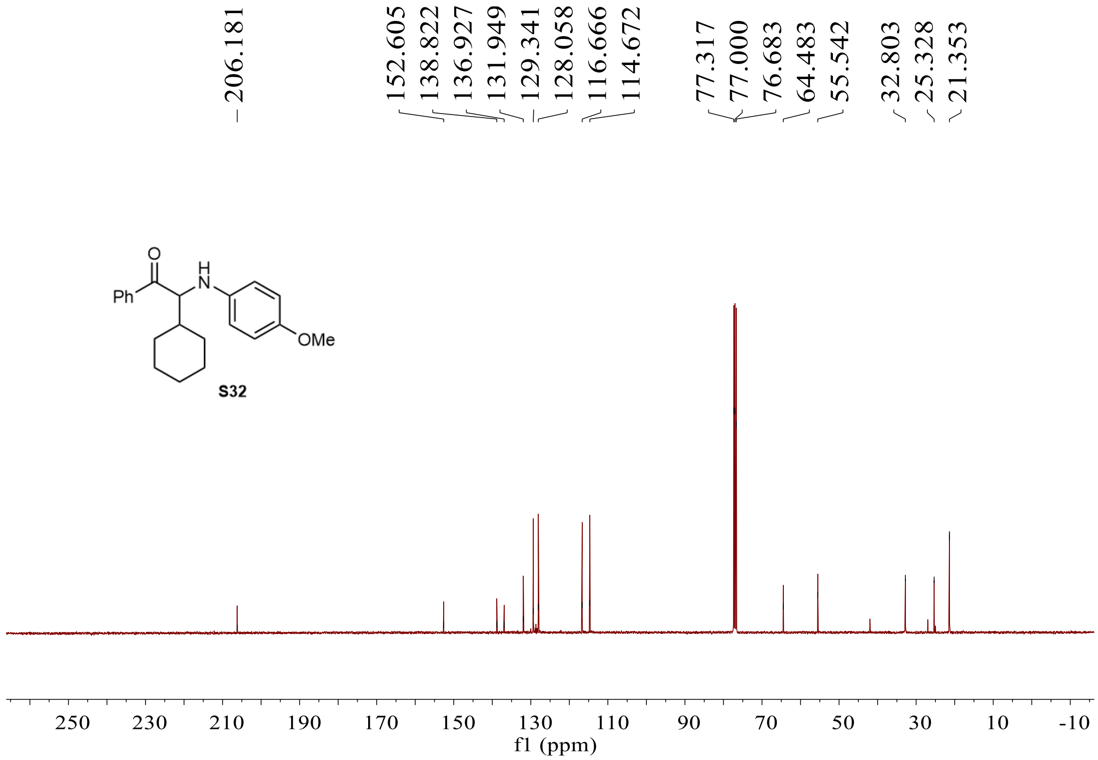

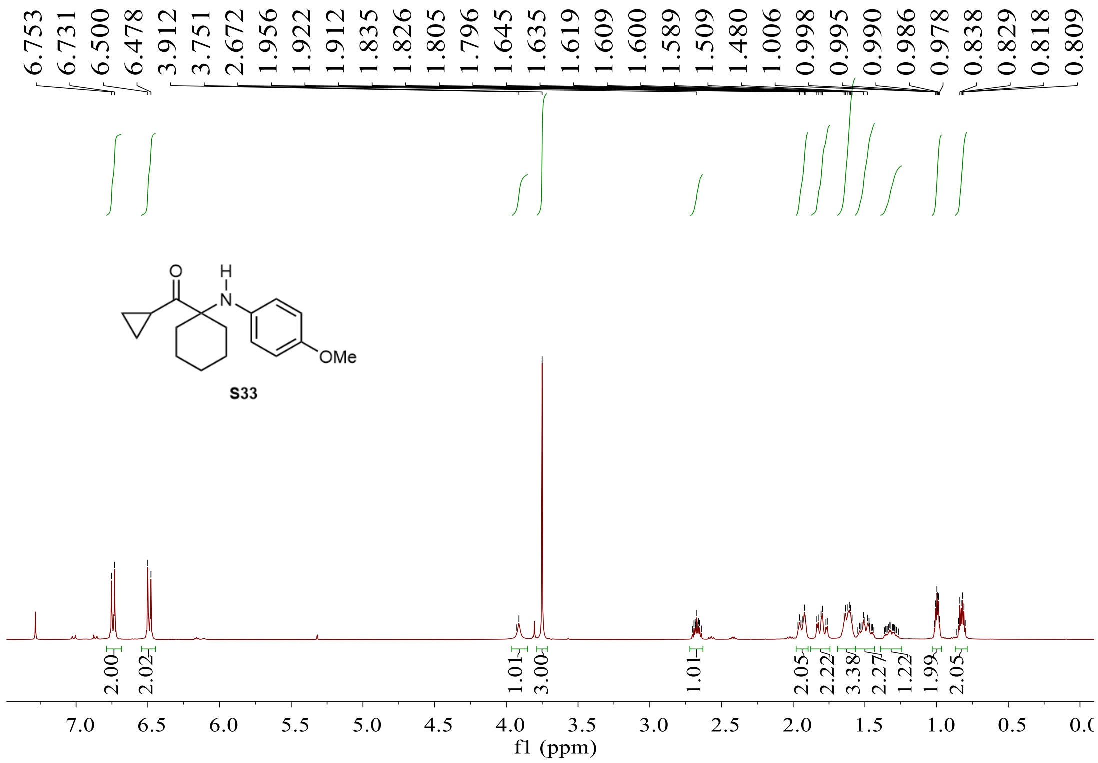

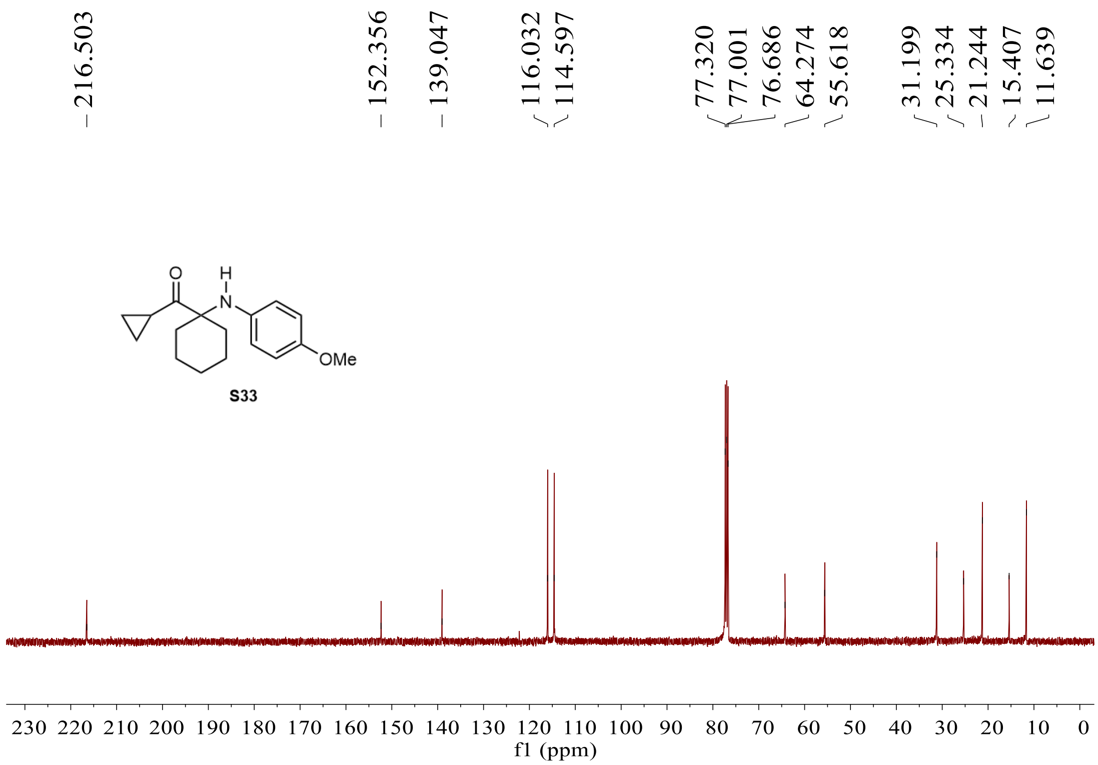
216.503
152.356
139.047
116.032
114.597
77.320
77.001
76.686
64.274
55.618
31.199
25.334
21.244
15.407
11.639
O
H
N
OMe
S33
230 220 210 200 190 180 170 160 150 140 130 120 110 100 90 80 70 60 50 40 30 20 10 0
f1 (ppm)

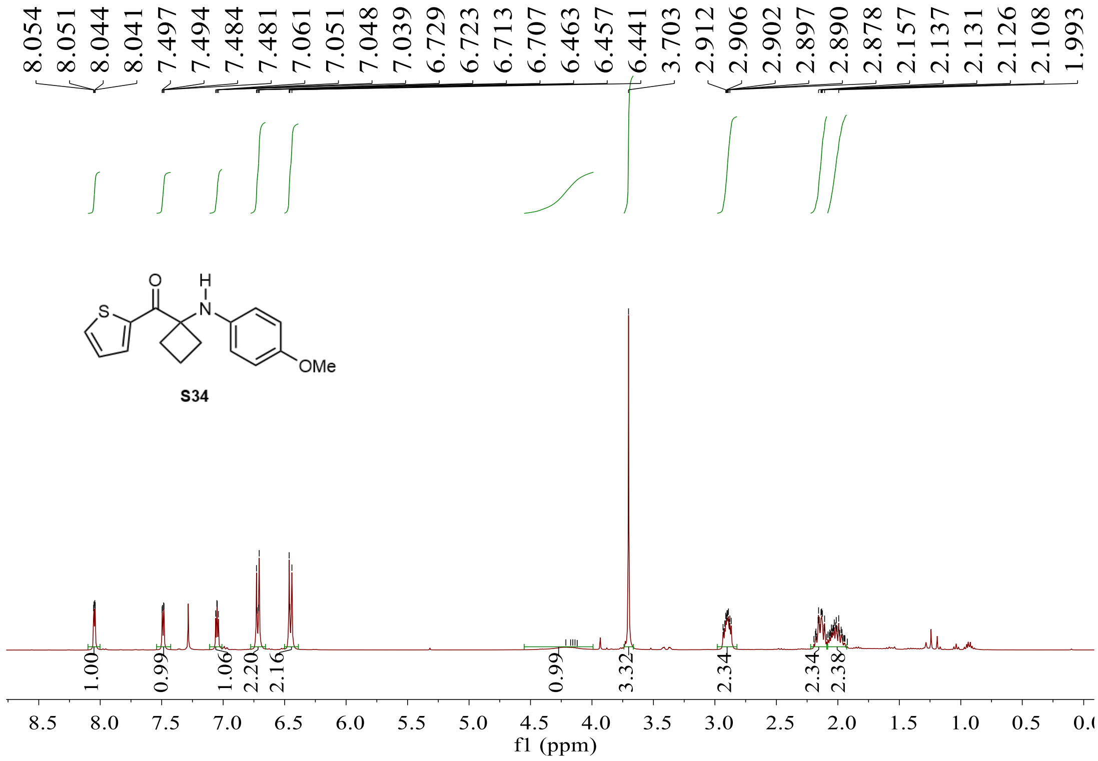
8.054
8.051
8.044
8.041
7.497
7.494
7.484
7.481
7.061
7.051
7.048
7.039
6.729
6.723
6.713
6.707
6.463
6.457
6.441
3.703
2.912
2.906
2.902
2.897
2.890
2.878
2.157
2.137
2.131
2.126
2.108
1.993
S
O
H
N
OMe
S34
1.00
0.99
1.06
2.20
2.16
0.99
3.32
2.34
2.34
2.38
8.5 8.0 7.5 7.0 6.5 6.0 5.5 5.0 4.5 4.0 3.5 3.0 2.5 2.0 1.5 1.0 0.5
f1 (ppm)

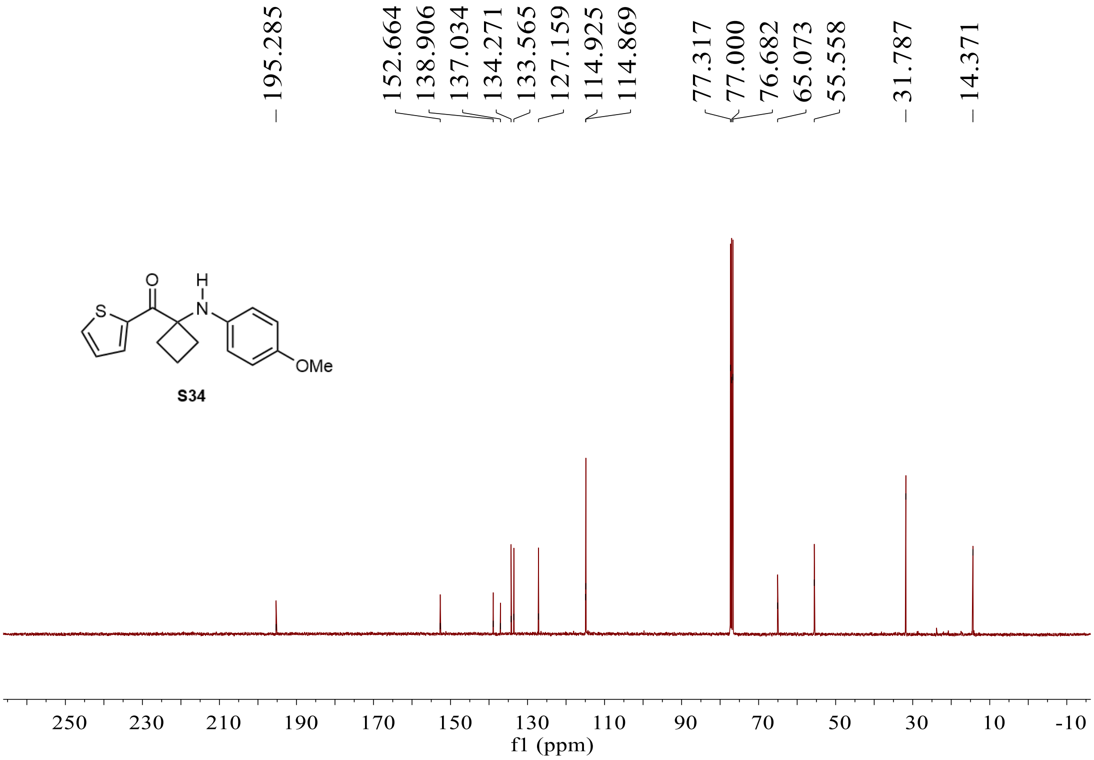
195.285
152.664
138.906
137.034
134.271
133.565
127.159
114.925
114.869
77.317
77.000
76.682
65.073
55.558
31.787
14.371
S
O
H
N
OMe
S34
250 230 210 190 170 150 130 110 90 70 50 30 10 -10
f1 (ppm)

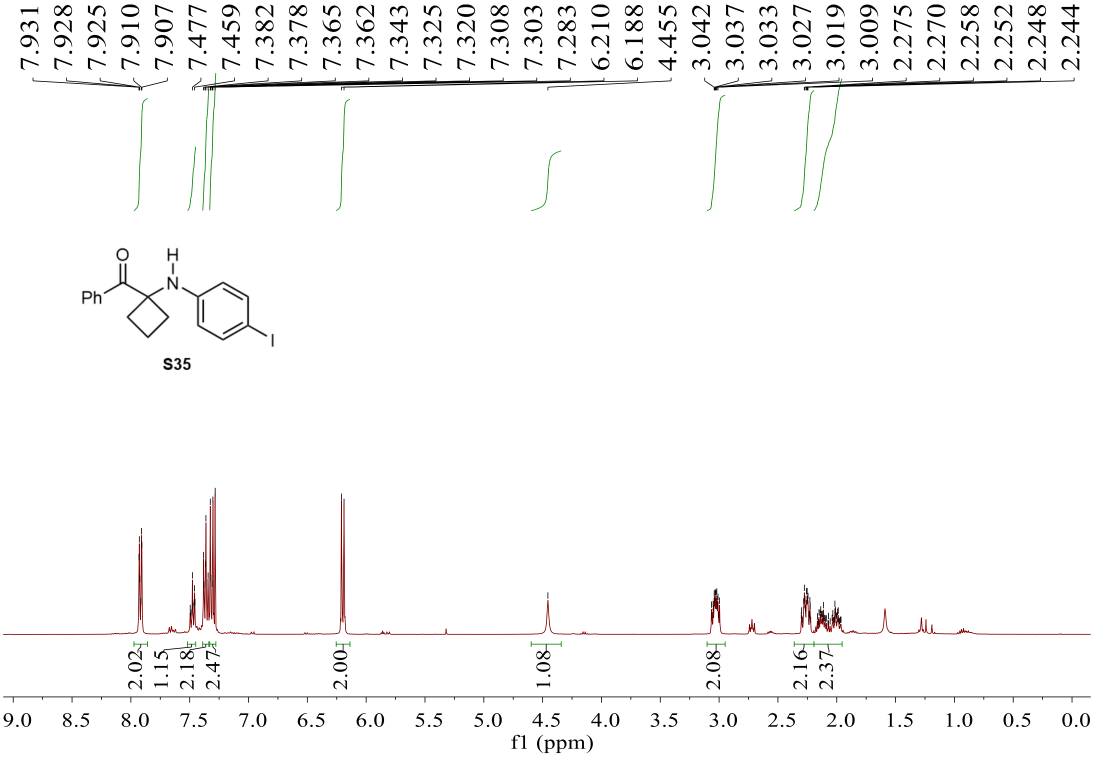
7.931
7.928
7.925
7.910
7.907
7.477
7.459
7.382
7.378
7.365
7.362
7.343
7.325
7.320
7.308
7.303
7.283
6.210
6.188
4.455
3.042
3.037
3.033
3.027
3.019
3.009
2.275
2.270
2.258
2.252
2.248
2.244
O
H
N
Ph
I
S35
2.02
1.15
2.18
2.47
2.00
1.08
2.08
2.16
2.37
9.0 8.5 8.0 7.5 7.0 6.5 6.0 5.5 5.0 4.5 4.0 3.5 3.0 2.5 2.0 1.5 1.0 0.5 0.0
f1 (ppm)

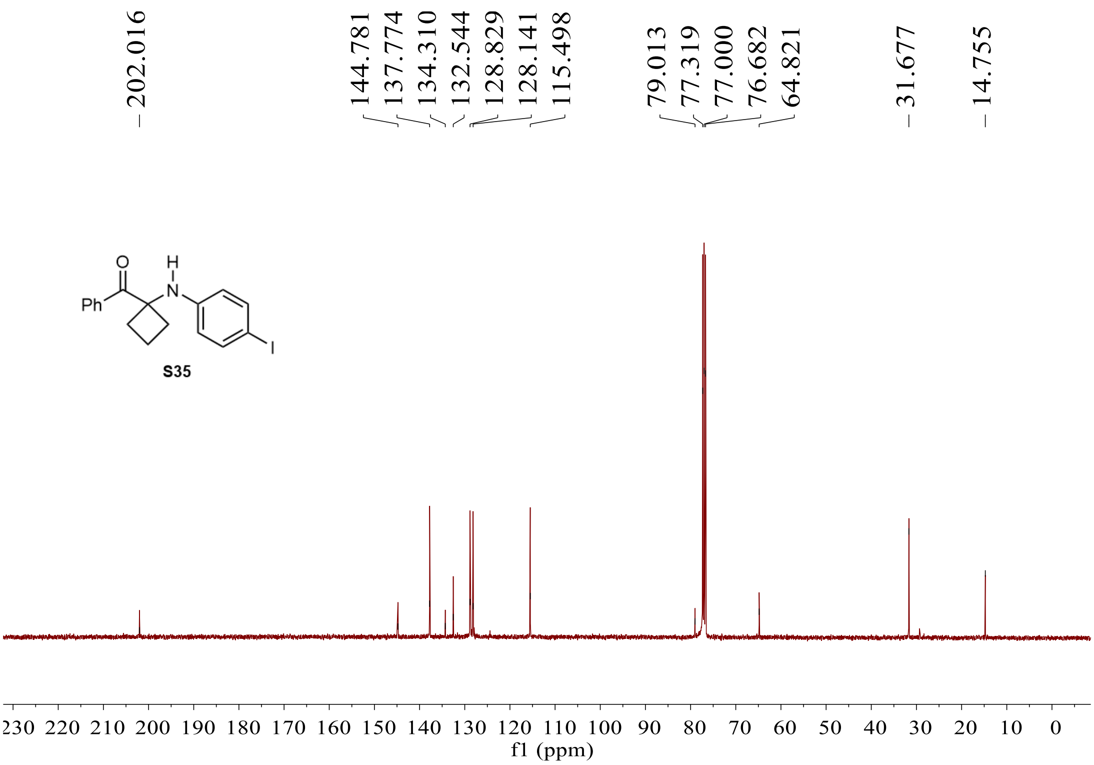
202.016
144.781
137.774
134.310
132.544
128.829
128.141
115.498
79.013
77.319
77.000
76.682
64.821
31.677
14.755
Ph
O
H
N
I
S35
f1 (ppm)

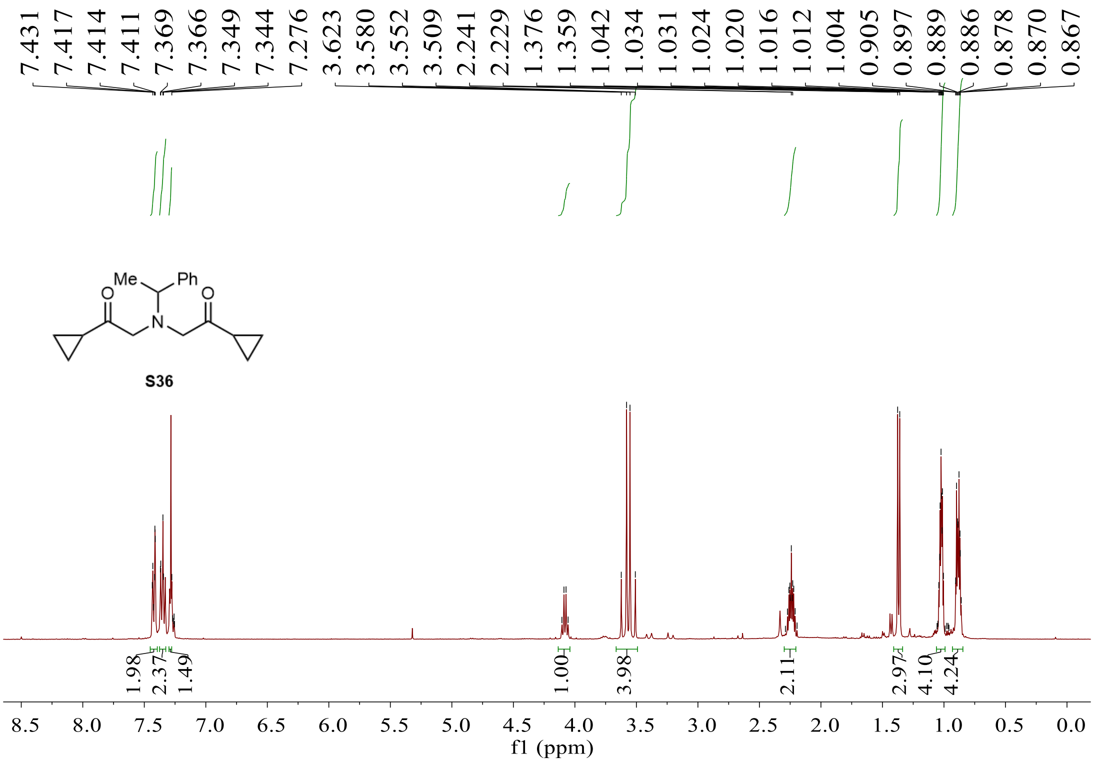
7.431
7.417
7.414
7.411
7.369
7.366
7.349
7.344
7.276
3.623
3.580
3.552
3.509
2.241
2.229
1.376
1.359
1.042
1.034
1.031
1.024
1.020
1.016
1.012
1.004
0.905
0.897
0.889
0.886
0.878
0.870
0.867
Me
Ph
O
N
O
S36
1.98
2.37
1.49
1.00
3.98
2.11
2.97
4.10
4.24
f1 (ppm)

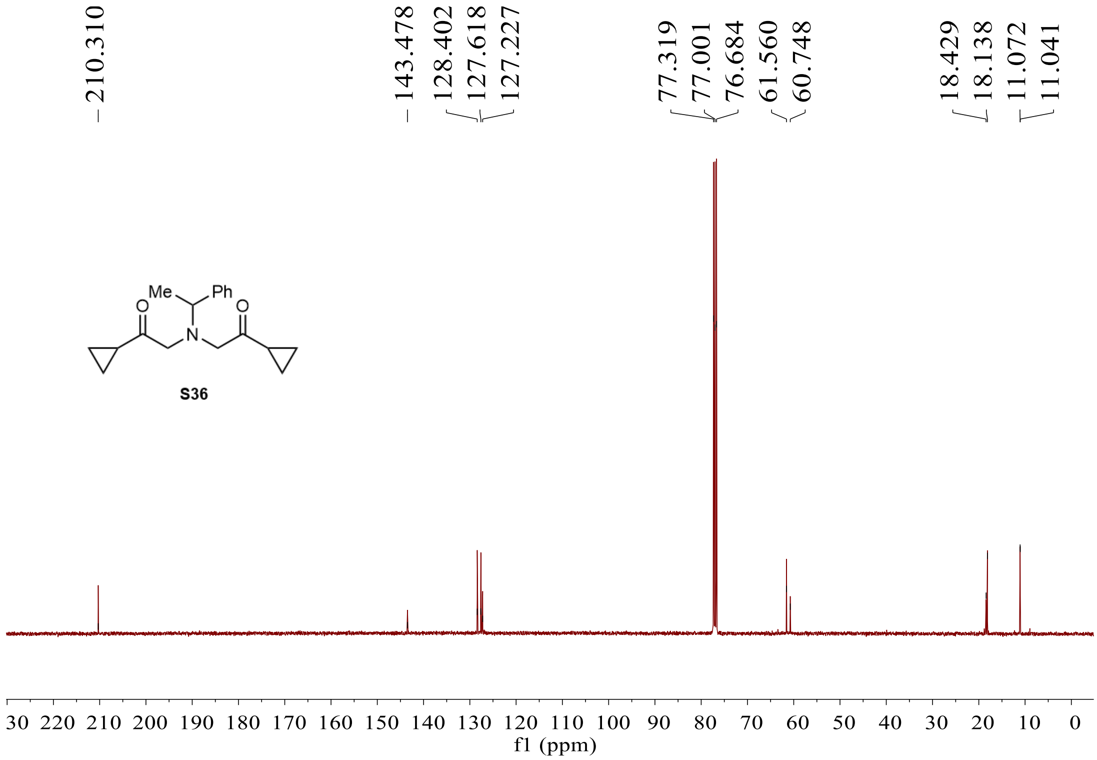

210.310
143.478
128.402
127.618
127.227
77.319
77.001
76.684
61.560
60.748
18.429
18.138
11.072
11.041
Me
Ph
O
N
f1 (ppm)

S36

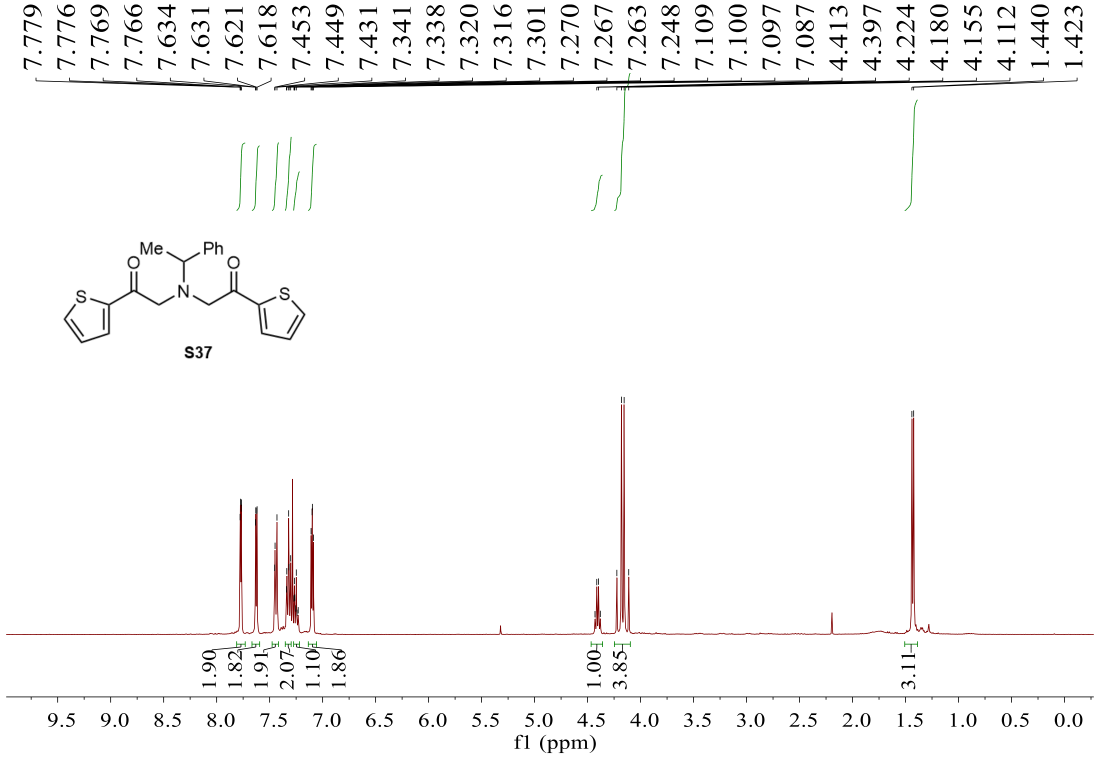

7.779
7.776
7.769
7.766
7.634
7.631
7.621
7.618
7.453
7.449
7.431
7.341
7.338
7.320
7.316
7.301
7.270
7.267
7.263
7.248
7.109
7.100
7.097
7.087
4.413
4.397
4.224
4.180
4.155
4.112
1.440
1.423
Me
Ph
O
N
S
1.90
1.82
1.91
2.07
1.10
1.86
1.00
3.85
3.11
f1 (ppm)

S37

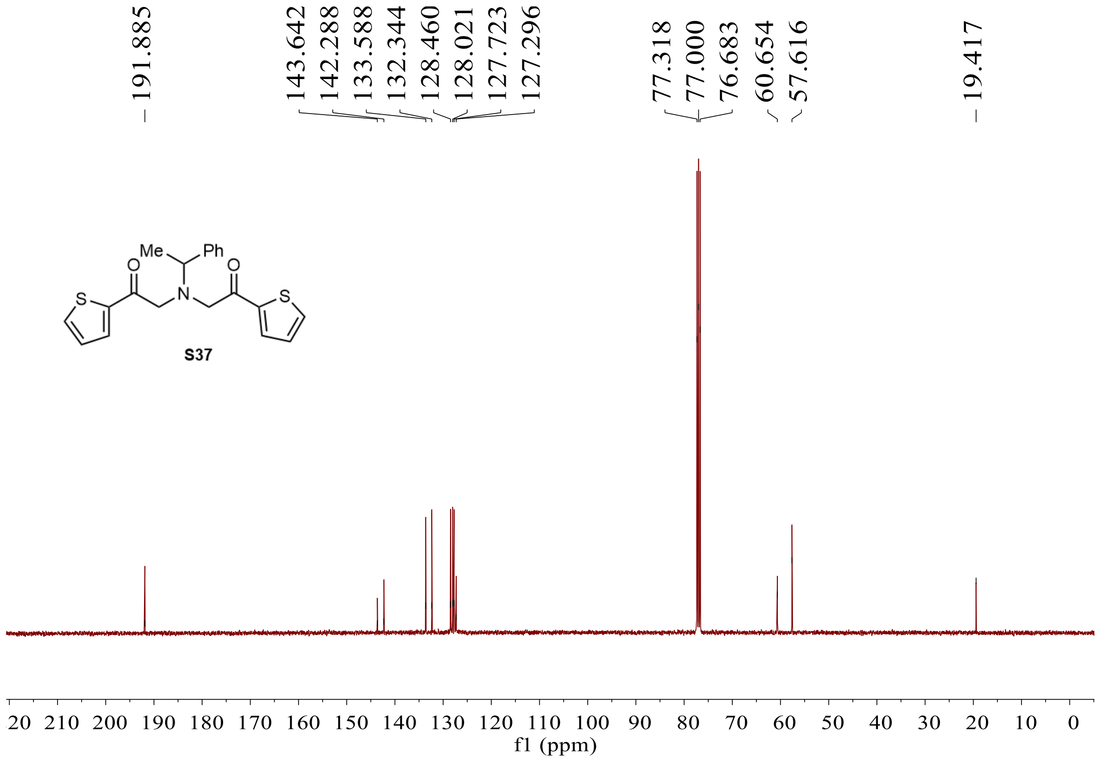
191.885
143.642
142.288
133.588
132.344
128.460
128.021
127.723
127.296
77.318
77.000
76.683
60.654
57.616
19.417
Me
Ph
O
N
S
S37
20 210 200 190 180 170 160 150 140 130 120 110 100 90 80 70 60 50 40 30 20 10 0
f1 (ppm)

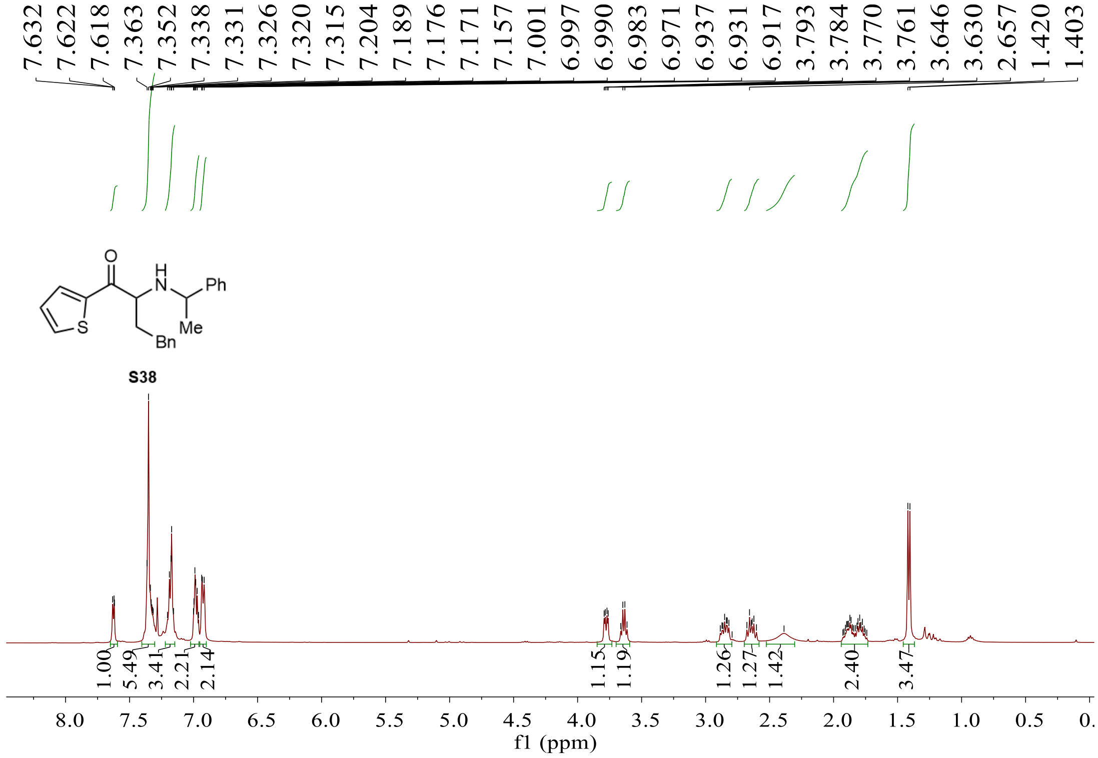
7.632
7.622
7.618
7.363
7.352
7.338
7.331
7.326
7.320
7.315
7.204
7.189
7.176
7.171
7.157
7.001
6.997
6.990
6.983
6.971
6.937
6.931
6.917
3.793
3.784
3.770
3.761
3.646
3.630
2.657
1.420
1.403
O
H
N
Ph
S
Me
Bn
S38
1.00
5.49
3.41
2.21
2.14
1.15
1.19
1.26
1.27
1.42
2.40
3.47
8.0 7.5 7.0 6.5 6.0 5.5 5.0 4.5 4.0 3.5 3.0 2.5 2.0 1.5 1.0 0.5 0.
f1 (ppm)

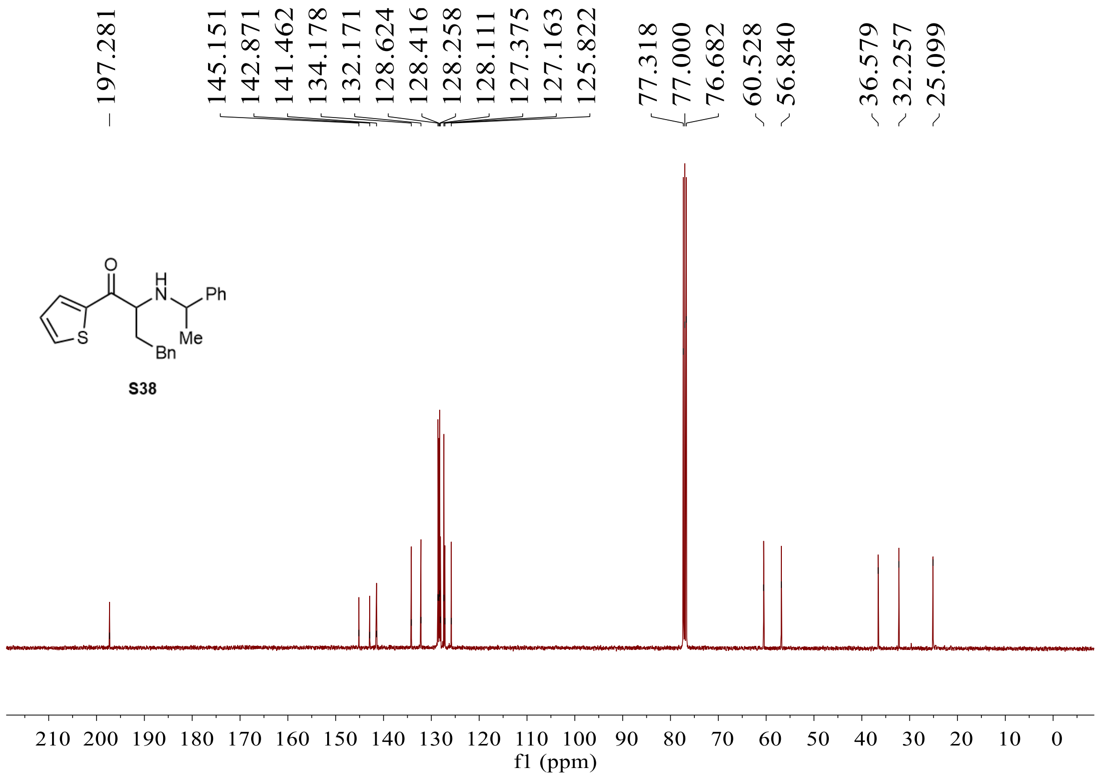
197.281
145.151
142.871
141.462
134.178
132.171
128.624
128.416
128.258
128.111
127.375
127.163
125.822
77.318
77.000
76.682
60.528
56.840
36.579
32.257
25.099
O
H
N
Ph
S
Me
Bn
S38
210 200 190 180 170 160 150 140 130 120 110 100 90 80 70 60 50 40 30 20 10 0
f1 (ppm)

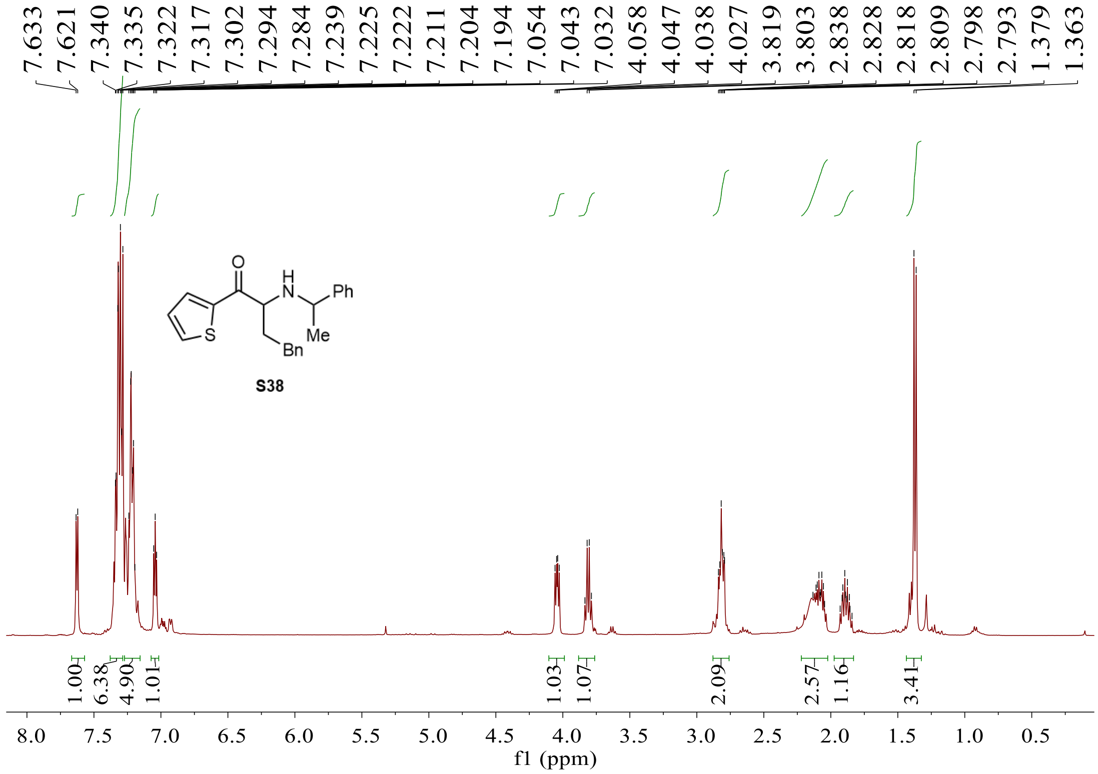
7.633
7.621
7.340
7.335
7.322
7.317
7.302
7.294
7.284
7.239
7.225
7.222
7.211
7.204
7.194
7.054
7.043
7.032
4.058
4.047
4.038
4.027
3.819
3.803
2.838
2.828
2.818
2.809
2.798
2.793
1.379
1.363
O
H
N
Ph
S
Me
Bn
S38
1.00
6.38
4.90
1.01
1.03
1.07
2.09
2.57
1.16
3.41
8.0 7.5 7.0 6.5 6.0 5.5 5.0 4.5 4.0 3.5 3.0 2.5 2.0 1.5 1.0 0.5
f1 (ppm)

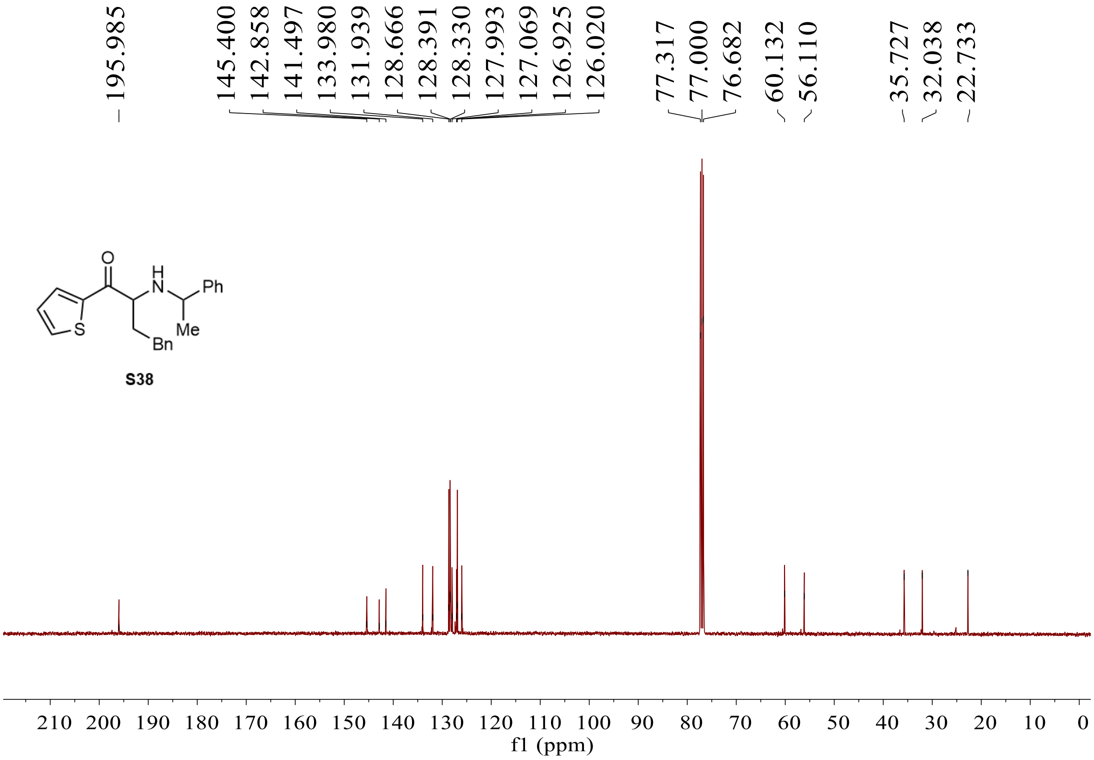

195.985
145.400 142.858 141.497 133.980 131.939 128.666 128.391 128.330 127.993 127.069 126.925 126.020
77.317 77.000 76.682 60.132 56.110
35.727 32.038 22.733
O
H
N
Ph
S
Me
Bn
S38
210 200 190 180 170 160 150 140 130 120 110 100 90 80 70 60 50 40 30 20 10 0
f1 (ppm)

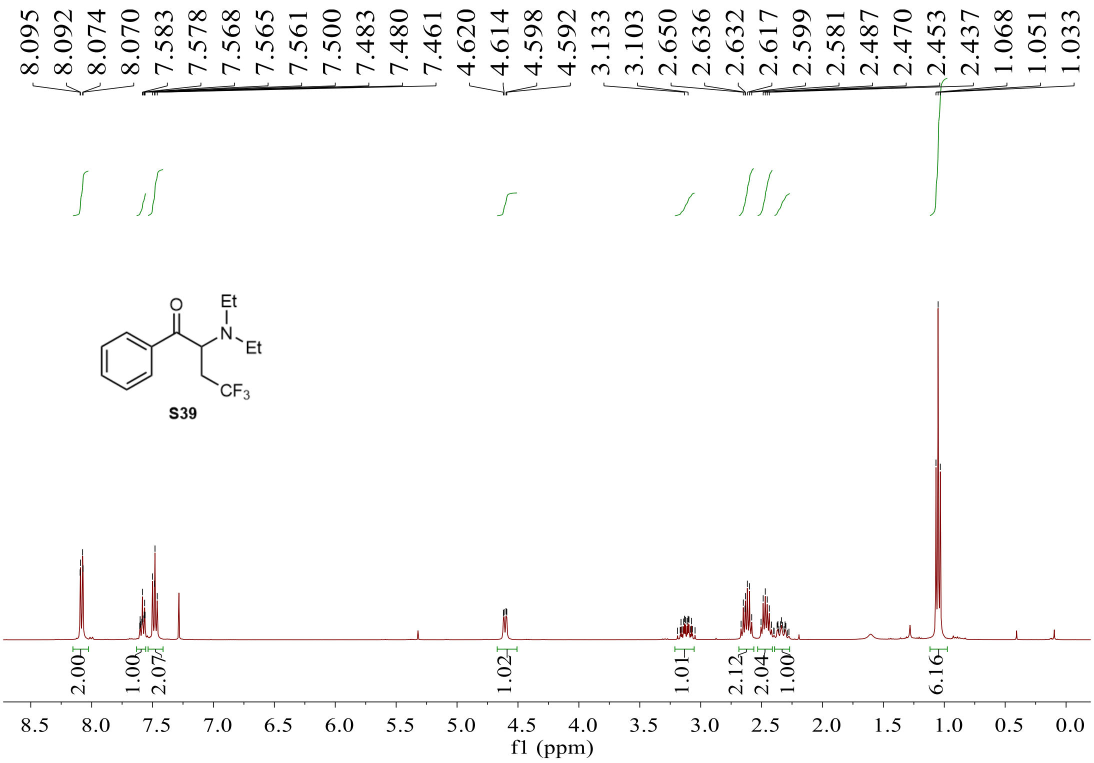

8.095 8.092 8.074 8.070 7.583 7.578 7.568 7.565 7.561 7.500 7.483 7.480 7.461 4.620 4.614 4.598 4.592 3.133 3.103 2.650 2.636 2.632 2.617 2.599 2.581 2.487 2.470 2.453 2.437 1.068 1.051 1.033
O
Et
N
Et
CF3
S39
2.00
1.00
2.07
1.02
1.01
2.12
2.04
1.00
6.16
8.5 8.0 7.5 7.0 6.5 6.0 5.5 5.0 4.5 4.0 3.5 3.0 2.5 2.0 1.5 1.0 0.5 0.0
f1 (ppm)

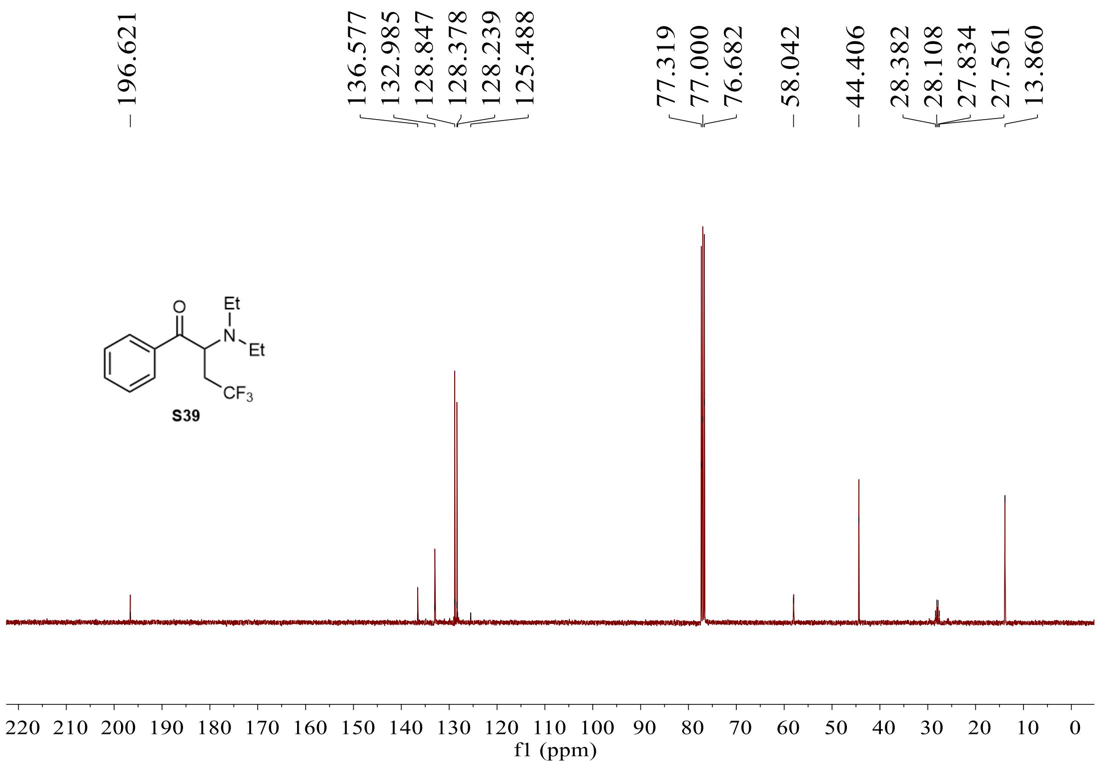
196.621
136.577
132.985
128.847
128.378
128.239
125.488
77.319
77.000
76.682
58.042
44.406
28.382
28.108
27.834
27.561
13.860
O
Et
N
Et
CF3
S39
220 210 200 190 180 170 160 150 140 130 120 110 100 90 80 70 60 50 40 30 20 10 0
f1 (ppm)

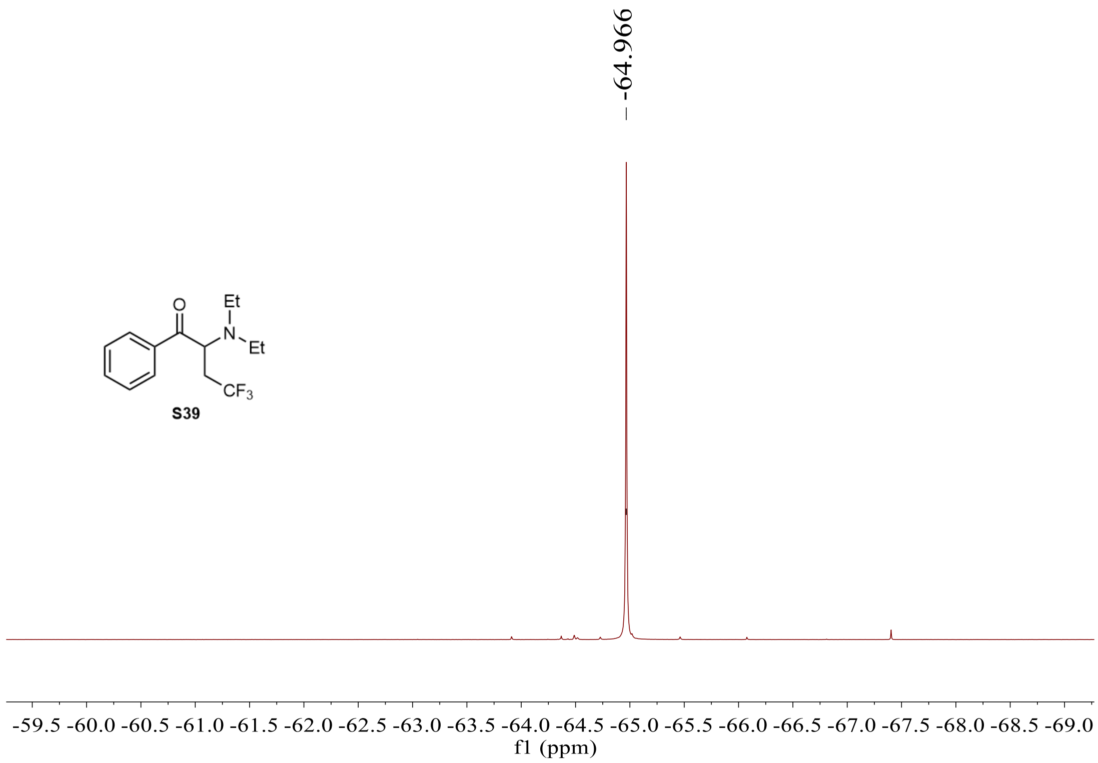
-64.966
O
Et
N
Et
CF3
S39
-59.5 -60.0 -60.5 -61.0 -61.5 -62.0 -62.5 -63.0 -63.5 -64.0 -64.5 -65.0 -65.5 -66.0 -66.5 -67.0 -67.5 -68.0 -68.5 -69.0
f1 (ppm)

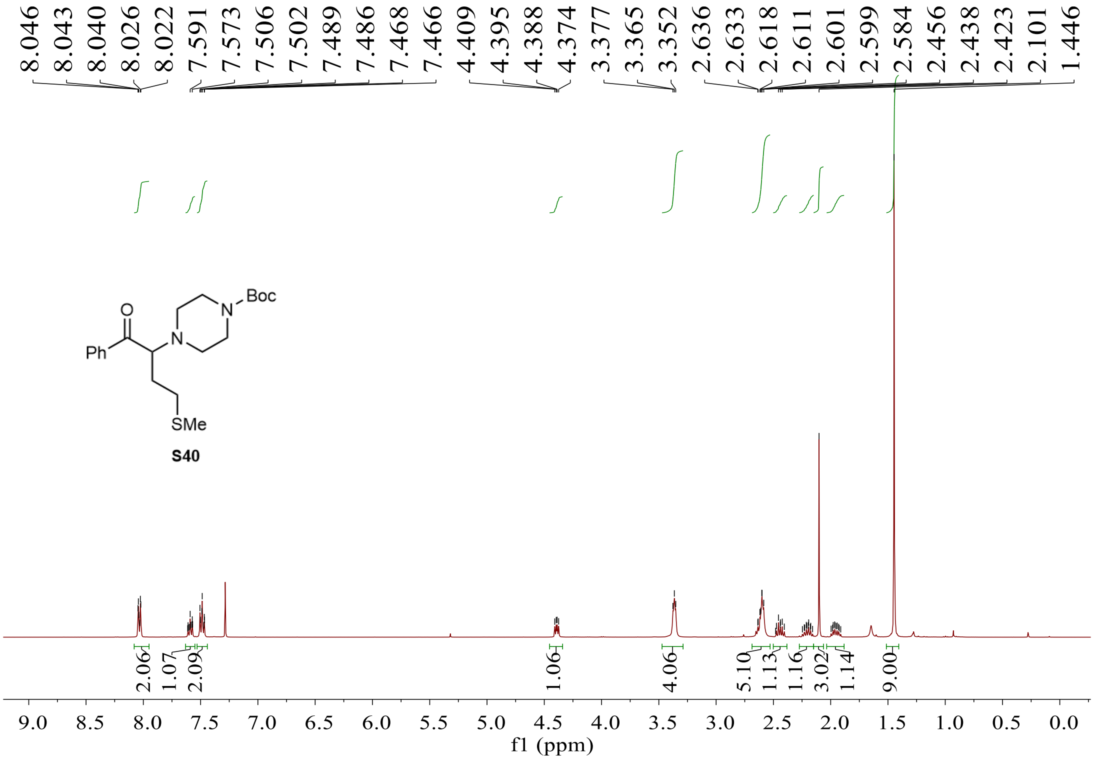
8.046
8.043
8.040
8.026
8.022
7.591
7.573
7.506
7.502
7.489
7.486
7.468
7.466
4.409
4.395
4.388
4.374
3.377
3.365
3.352
2.636
2.633
2.618
2.611
2.601
2.599
2.584
2.456
2.438
2.423
2.101
1.446
Boc
O
N
N
Ph
SMe
S40
2.06
1.07
2.09
1.06
4.06
5.10
1.13
1.16
3.02
1.14
9.00
9.0 8.5 8.0 7.5 7.0 6.5 6.0 5.5 5.0 4.5 4.0 3.5 3.0 2.5 2.0 1.5 1.0 0.5 0.0
f1 (ppm)

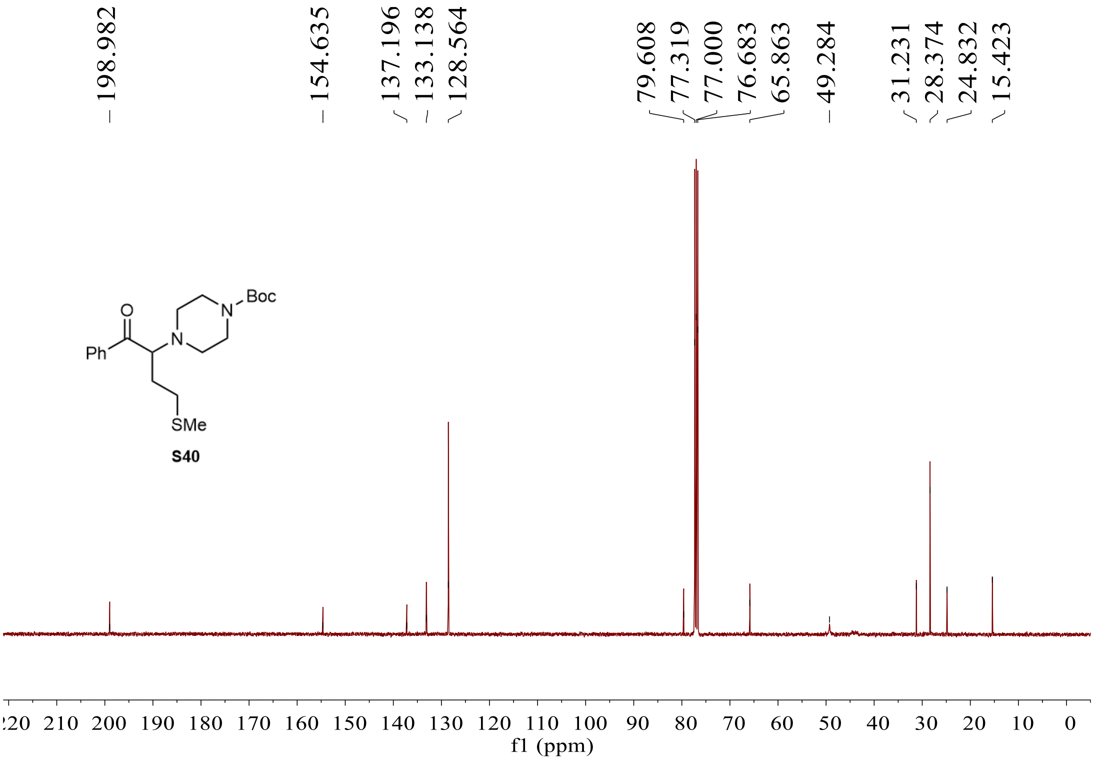
198.982
154.635
137.196
133.138
128.564
79.608
77.319
77.000
76.683
65.863
49.284
31.231
28.374
24.832
15.423
Boc
O
N
N
Ph
SMe
S40
220 210 200 190 180 170 160 150 140 130 120 110 100 90 80 70 60 50 40 30 20 10 0
f1 (ppm)

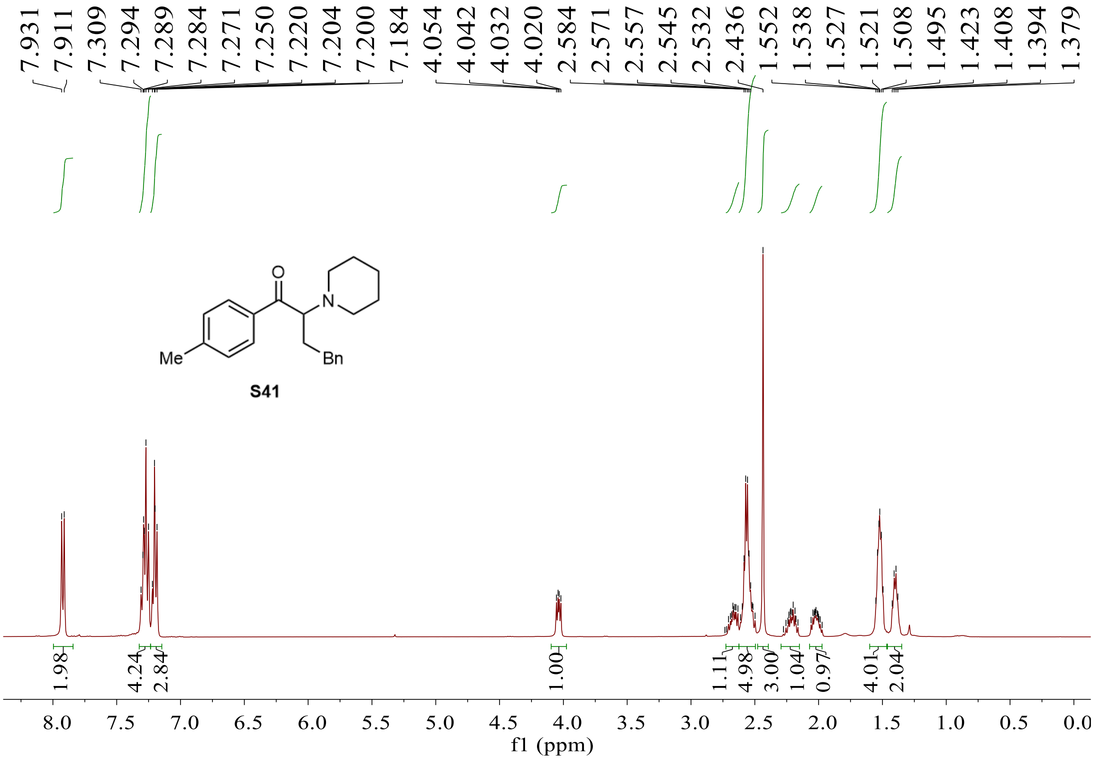
7.931
7.911
7.309
7.294
7.289
7.284
7.271
7.250
7.220
7.204
7.200
7.184
4.054
4.042
4.032
4.020
2.584
2.571
2.557
2.545
2.532
2.436
1.552
1.538
1.527
1.521
1.508
1.495
1.423
1.408
1.394
1.379
O
N
Me
Bn
1.98
4.24
2.84
1.00
1.11
4.98
3.00
1.04
0.97
4.01
2.04
8.0
7.5
7.0
6.5
6.0
5.5
5.0
4.5
4.0
3.5
3.0
2.5
2.0
1.5
1.0
0.5
0.0
f1 (ppm)

S41

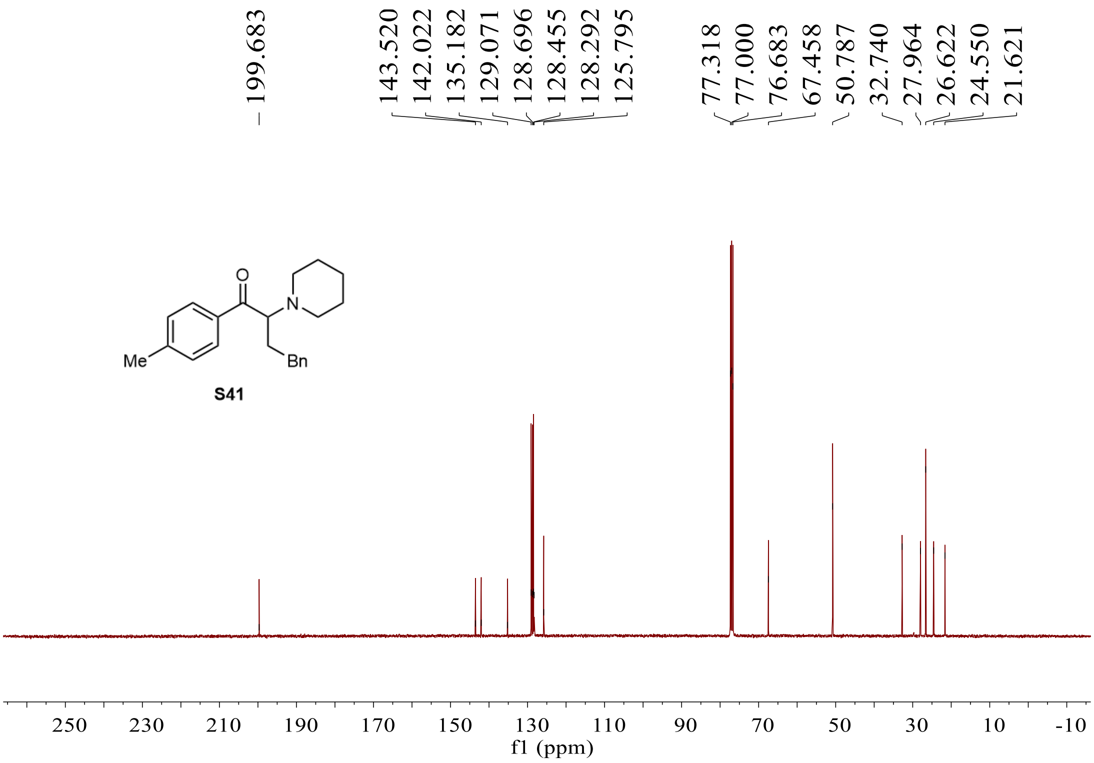
199.683
143.520
142.022
135.182
129.071
128.696
128.455
128.292
125.795
77.318
77.000
76.683
67.458
50.787
32.740
27.964
26.622
24.550
21.621
O
N
Me
Bn
250
230
210
190
170
150
130
110
90
70
50
30
10
-10
f1 (ppm)

S41

^{19}F-NMR to calculate the assay yield

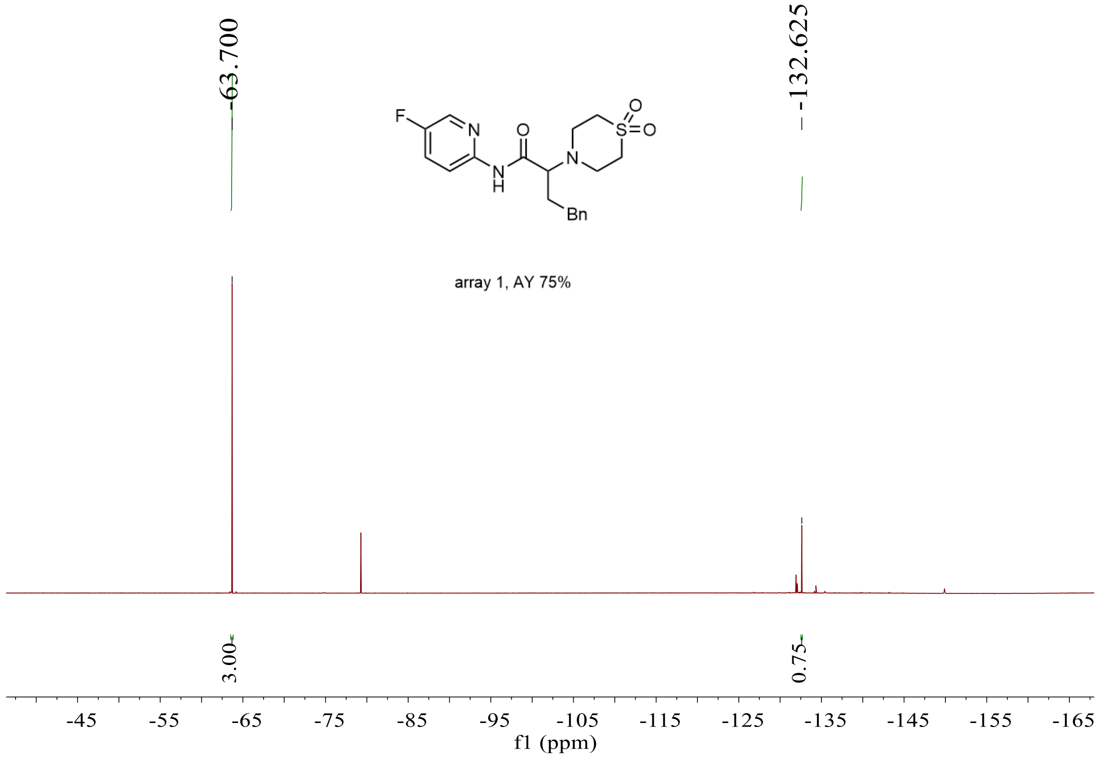

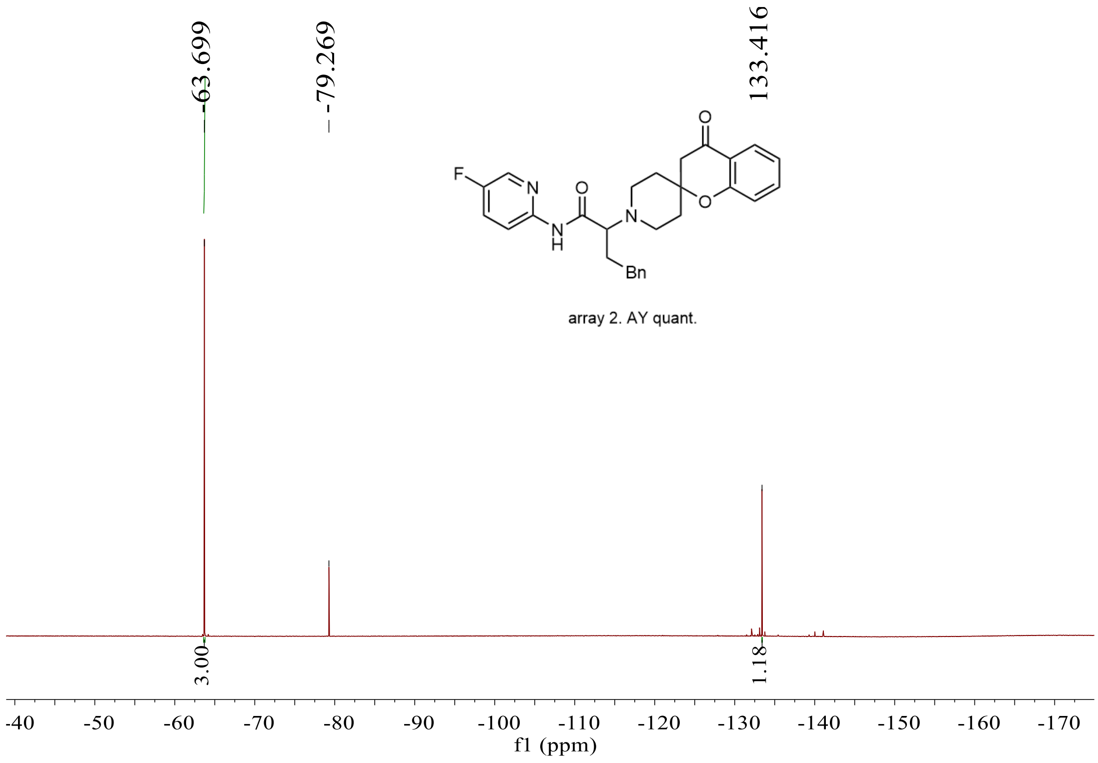

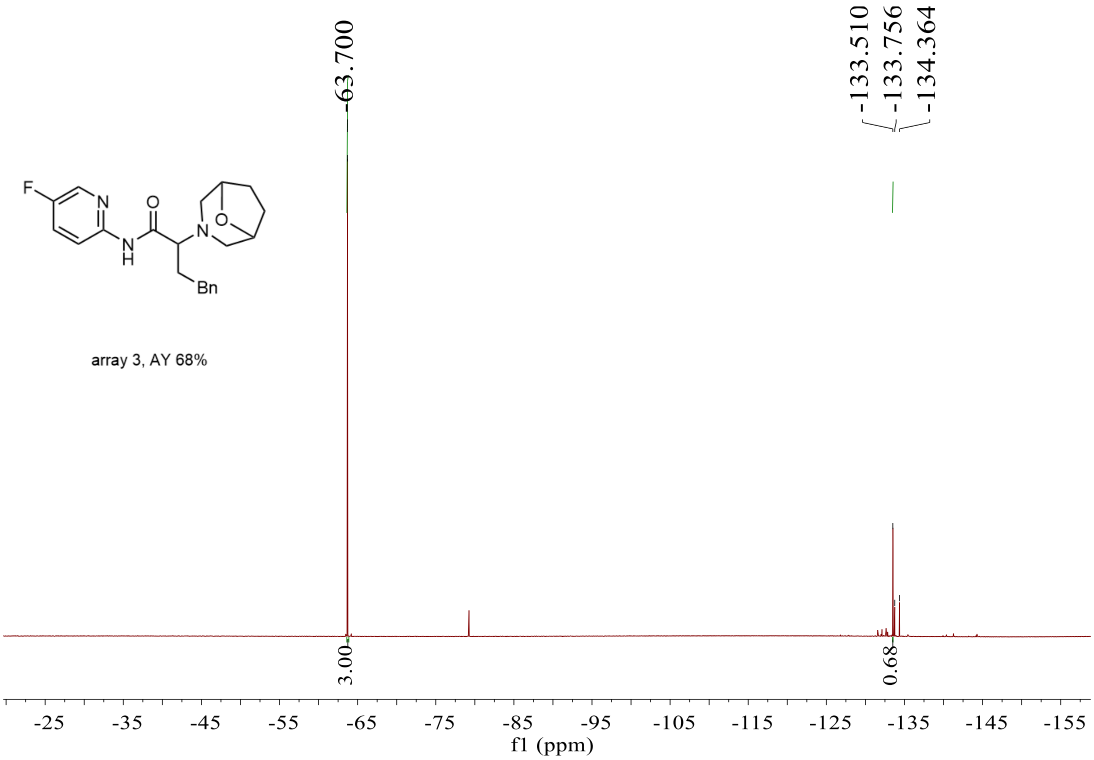

array 3, AY 68%

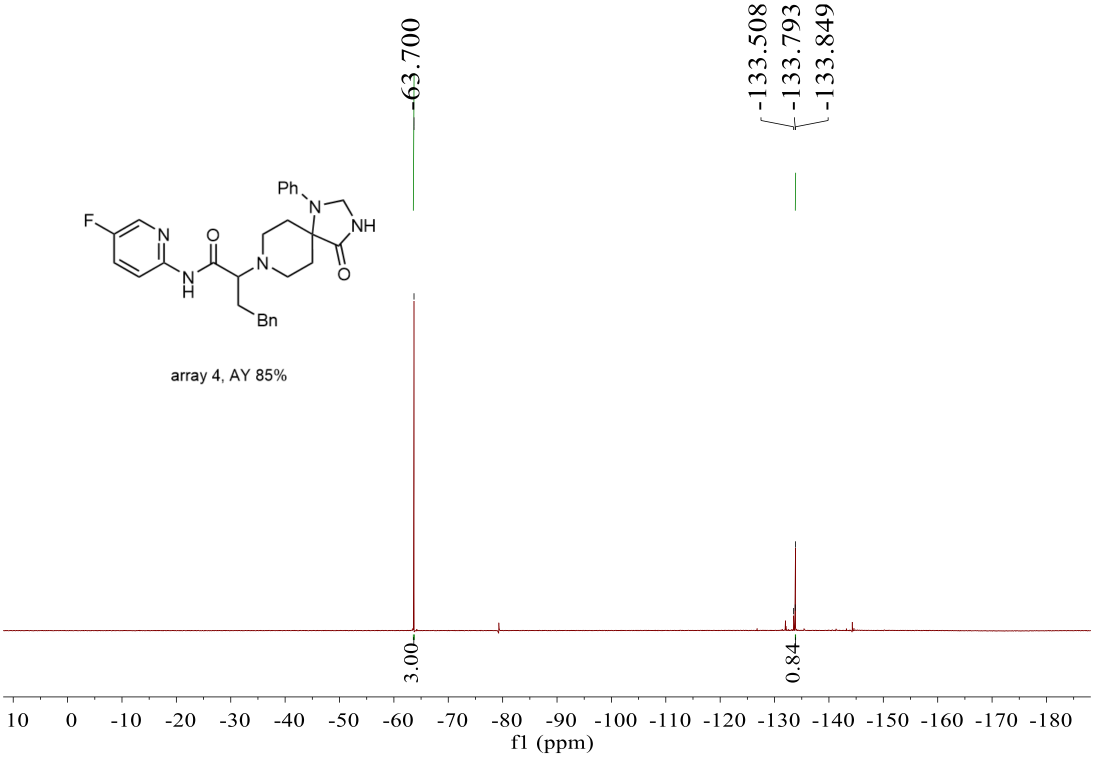

array 4, AY 85%

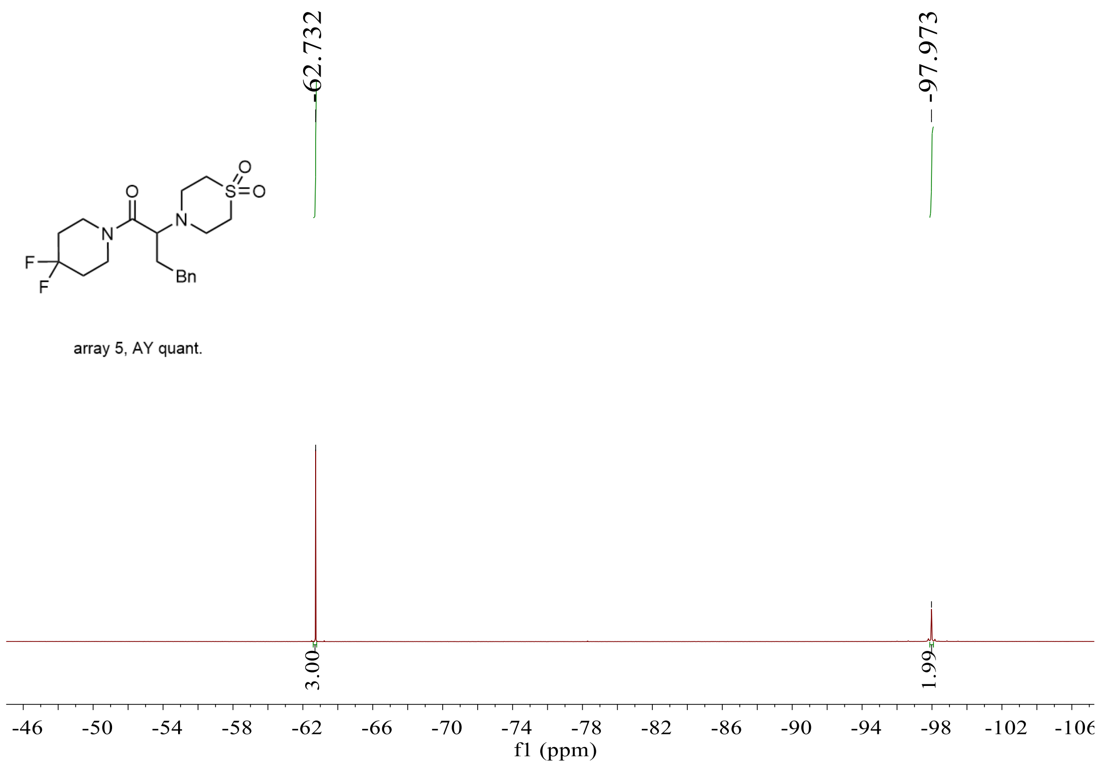
-62.732
-97.973
F
F
N
O
N
S
O
O
Bn
array 5, AY quant.
3.00
1.99
-46
-50
-54
-58
-62
-66
-70
-74
-78
-82
-86
-90
-94
-98
-102
-106
f1 (ppm)

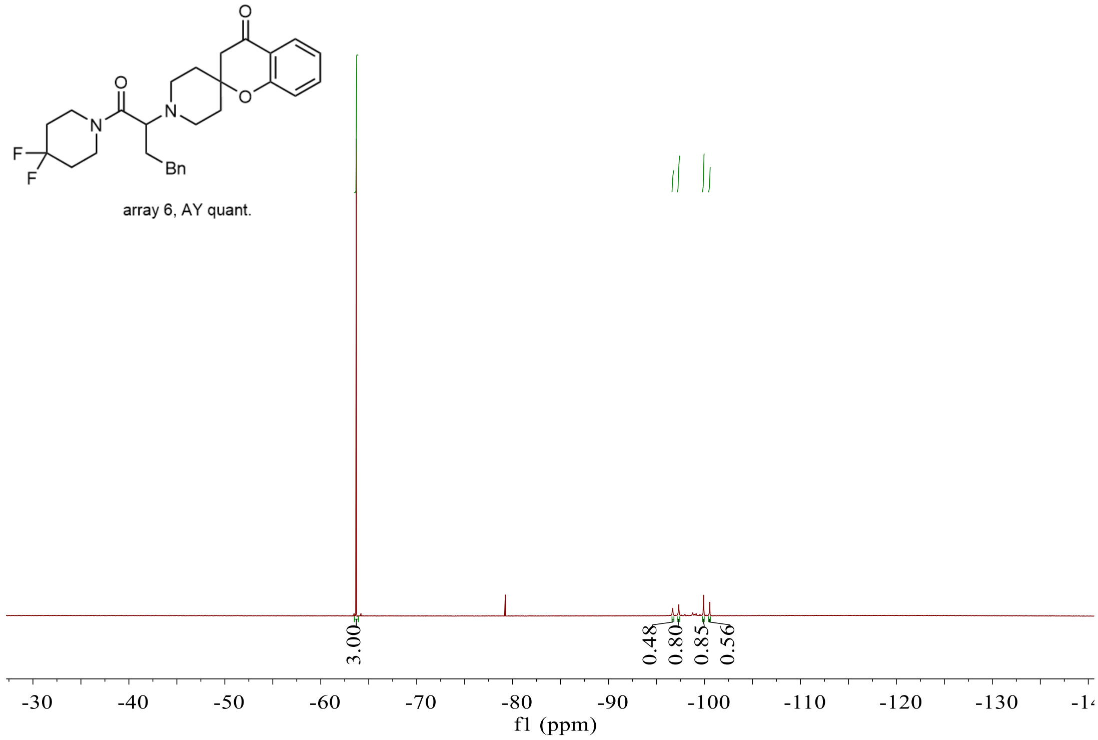
O
O
F
F
N
O
N
Bn
array 6, AY quant.
3.00
0.48
0.80
0.85
0.56
-30
-40
-50
-60
-70
-80
-90
-100
-110
-120
-130
f1 (ppm)

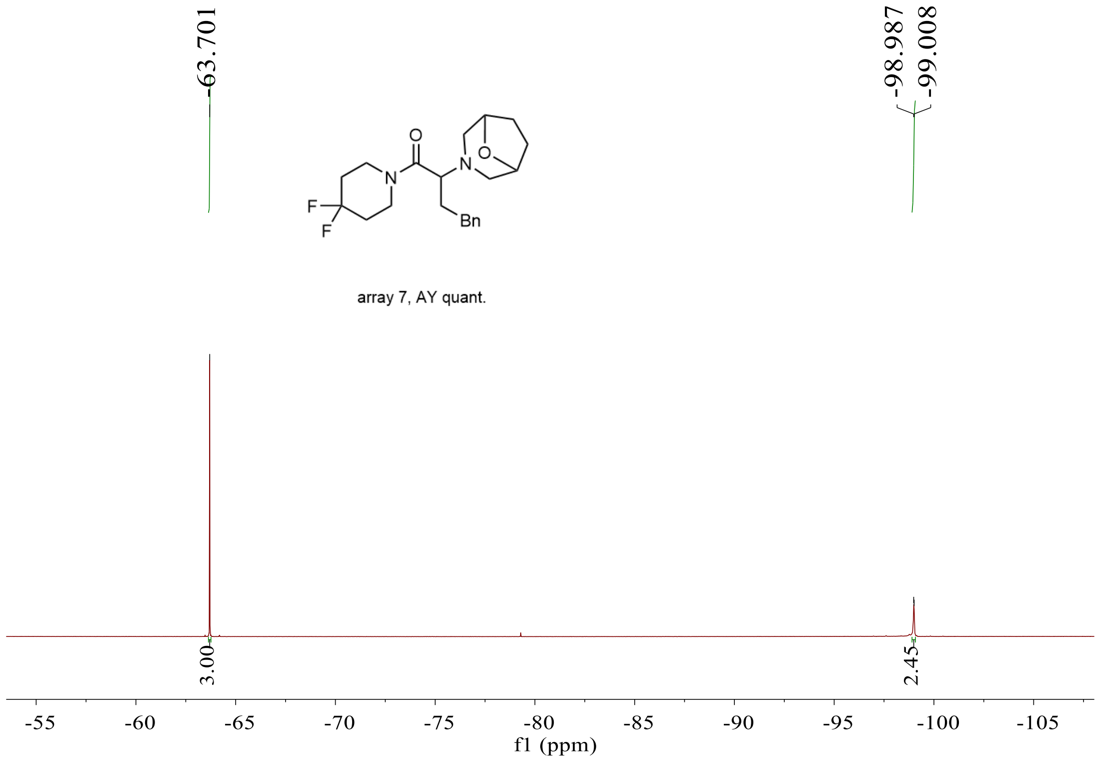

-63.701
-98.987
-99.008
O
N
N
O
F
F
Bn
array 7, AY quant.
3.00
2.45
-55 -60 -65 -70 -75 -80 -85 -90 -95 -100 -105
f1 (ppm)

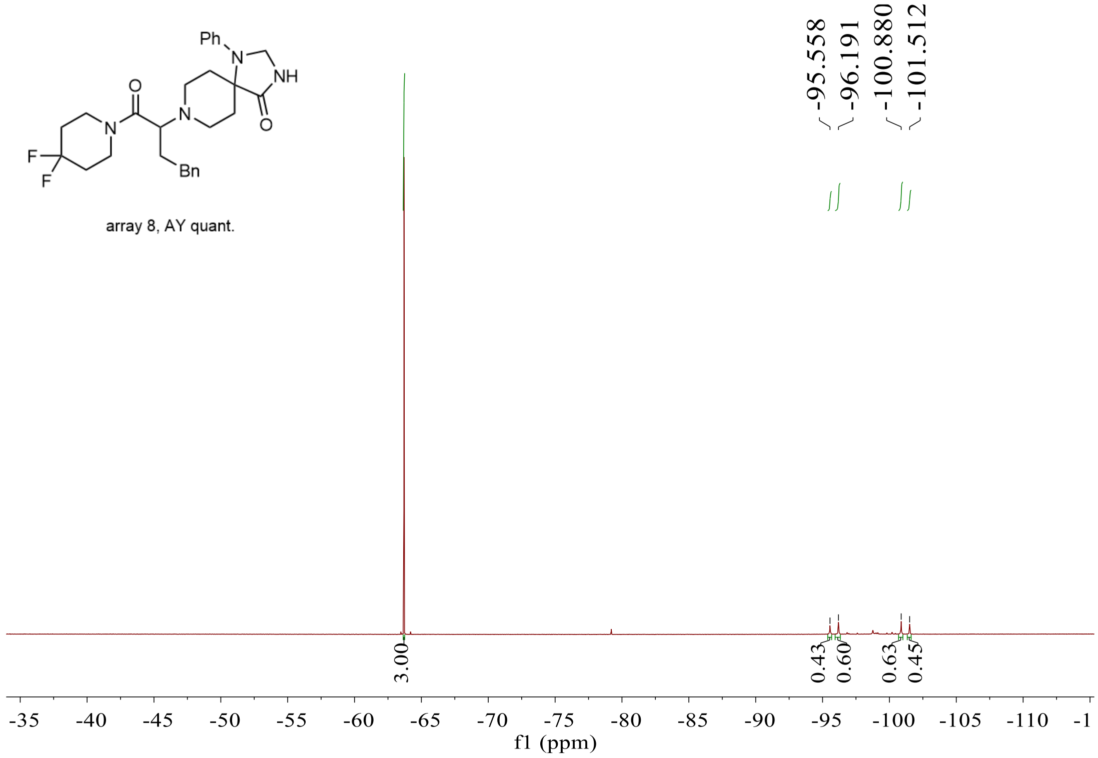

Ph
N
NH
O
N
N
O
F
F
Bn
array 8, AY quant.
-95.558
-96.191
-100.880
-101.512
3.00
0.43
0.60
0.63
0.45
-35 -40 -45 -50 -55 -60 -65 -70 -75 -80 -85 -90 -95 -100 -105 -110
f1 (ppm)

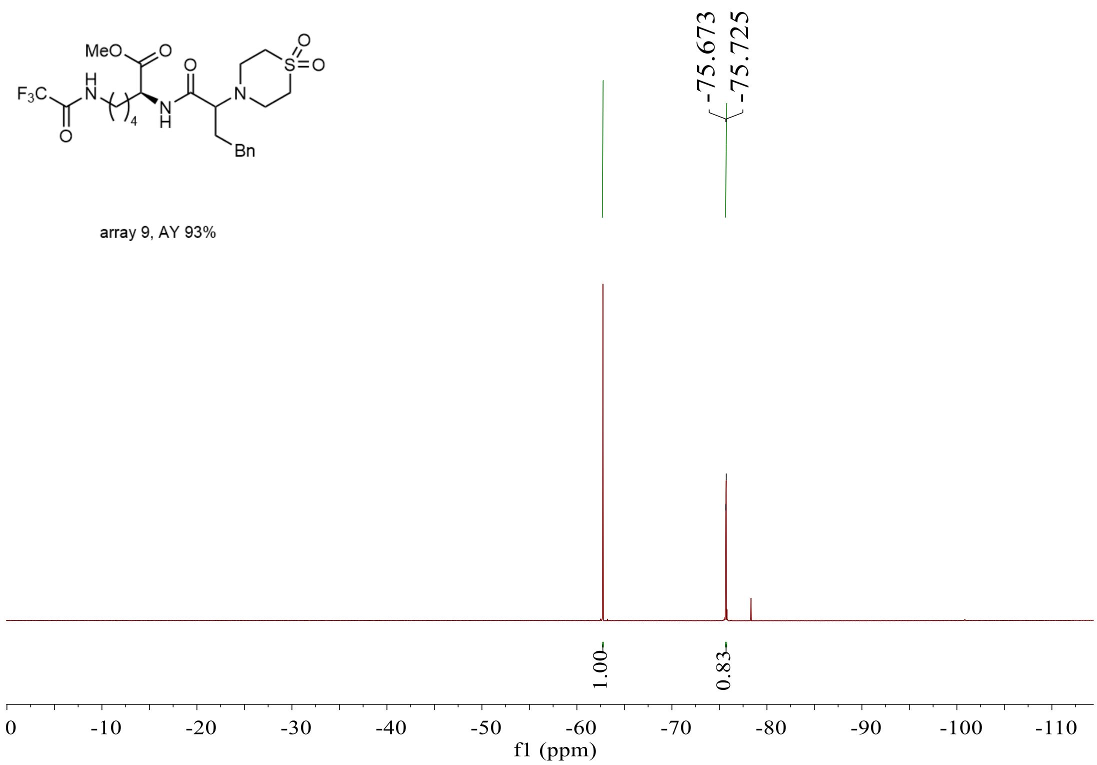

MeO
F3C
Bn
array 9, AY 93%
-75.673
-75.725
1.00
0.83
0 -10 -20 -30 -40 -50 -60 -70 -80 -90 -100 -110
f1 (ppm)

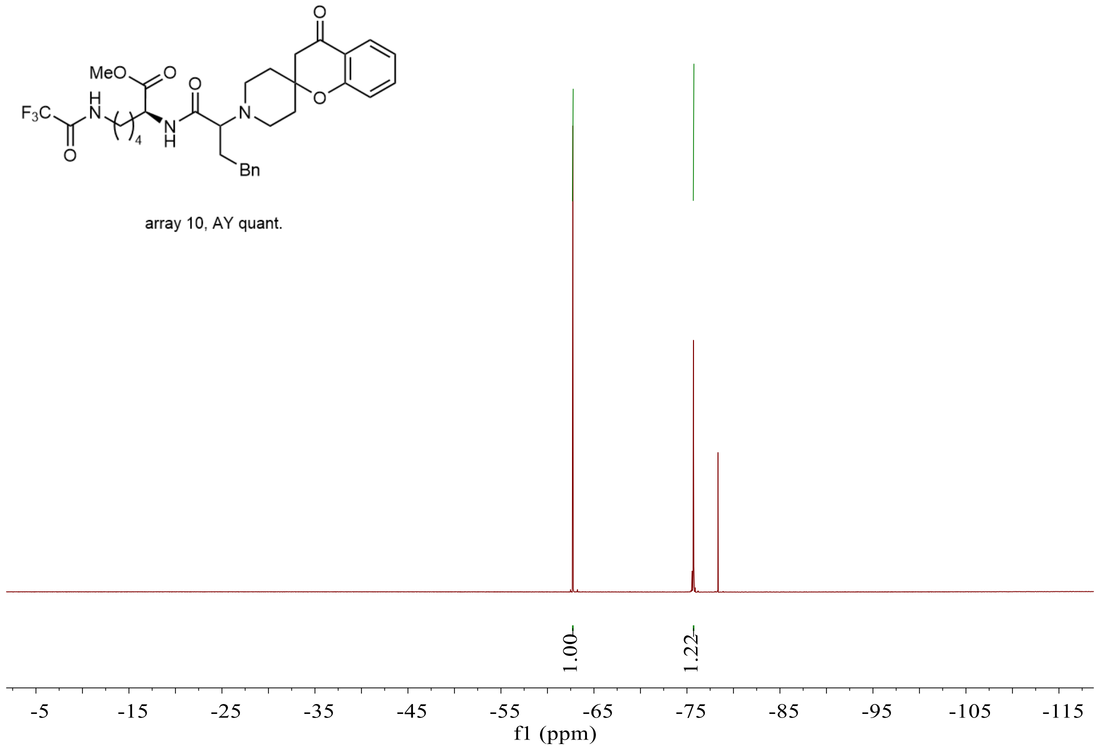

MeO
F3C
Bn
array 10, AY quant.
1.00
1.22
-5 -15 -25 -35 -45 -55 -65 -75 -85 -95 -105 -115
f1 (ppm)

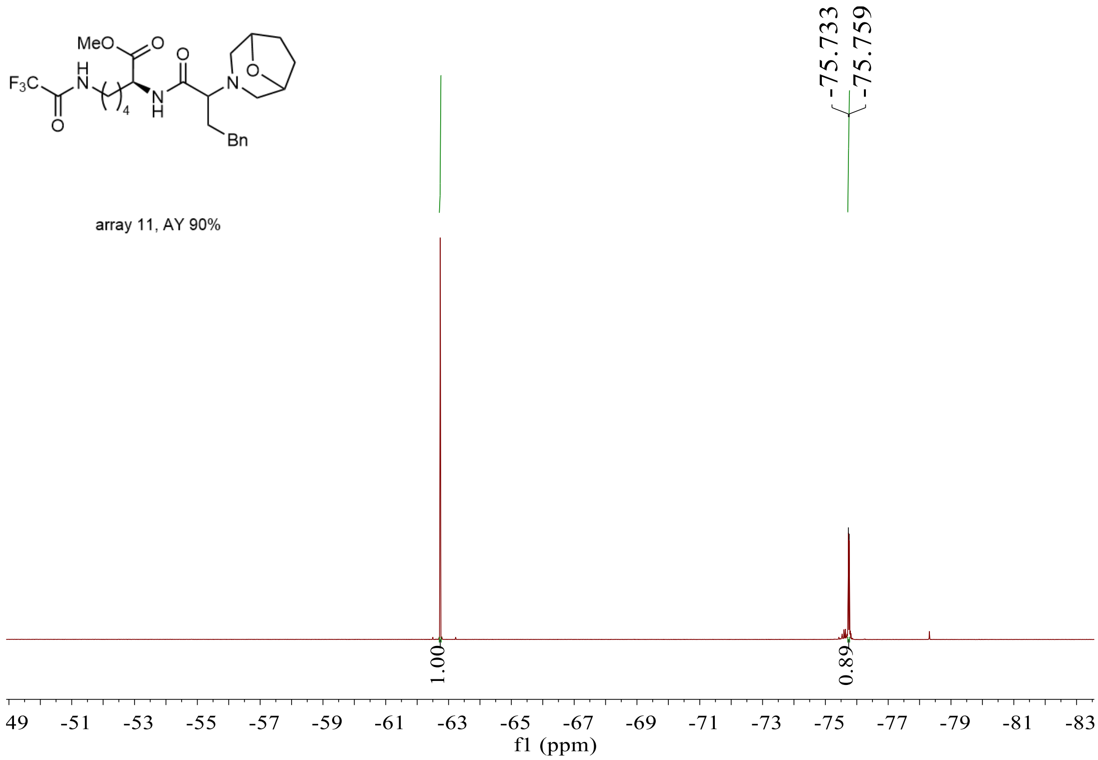

array 11, AY 90%

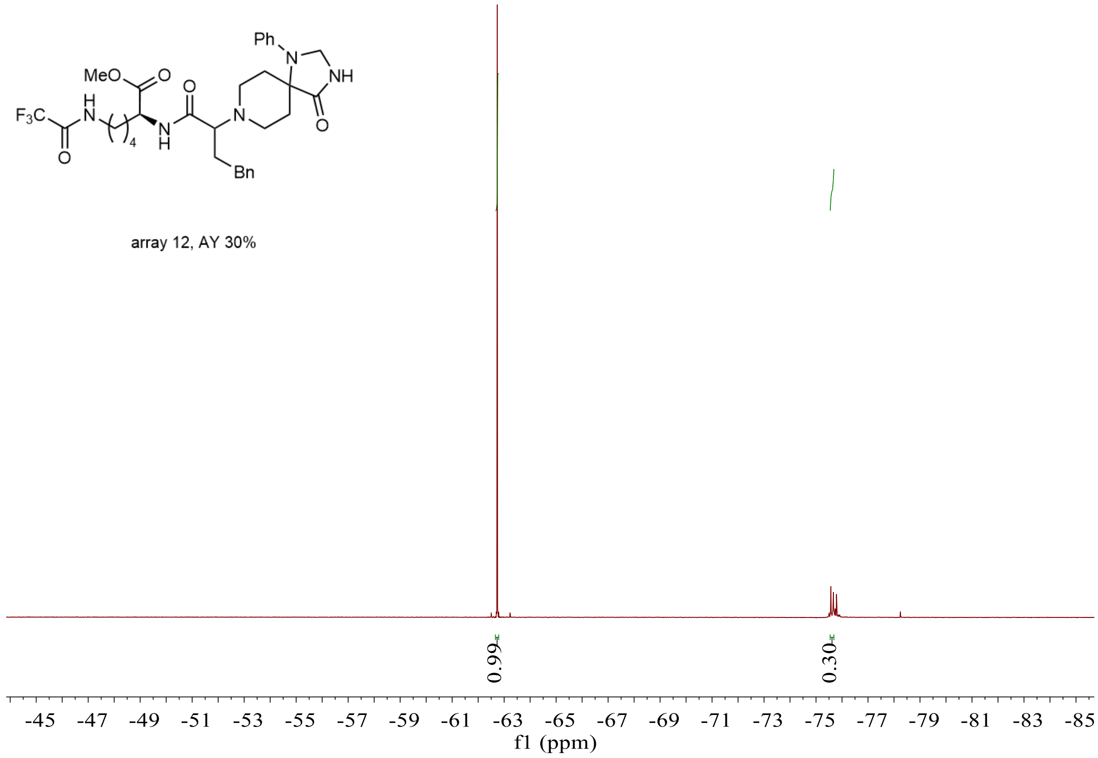

array 12, AY 30%

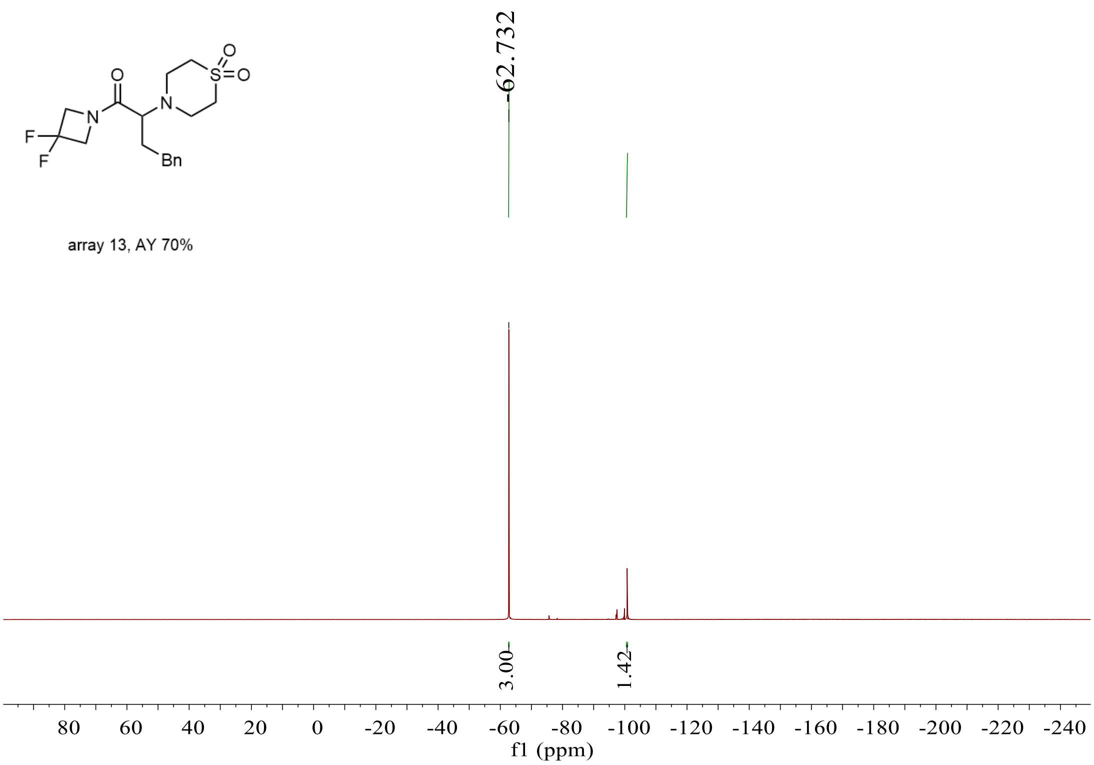

array 13, AY 70%

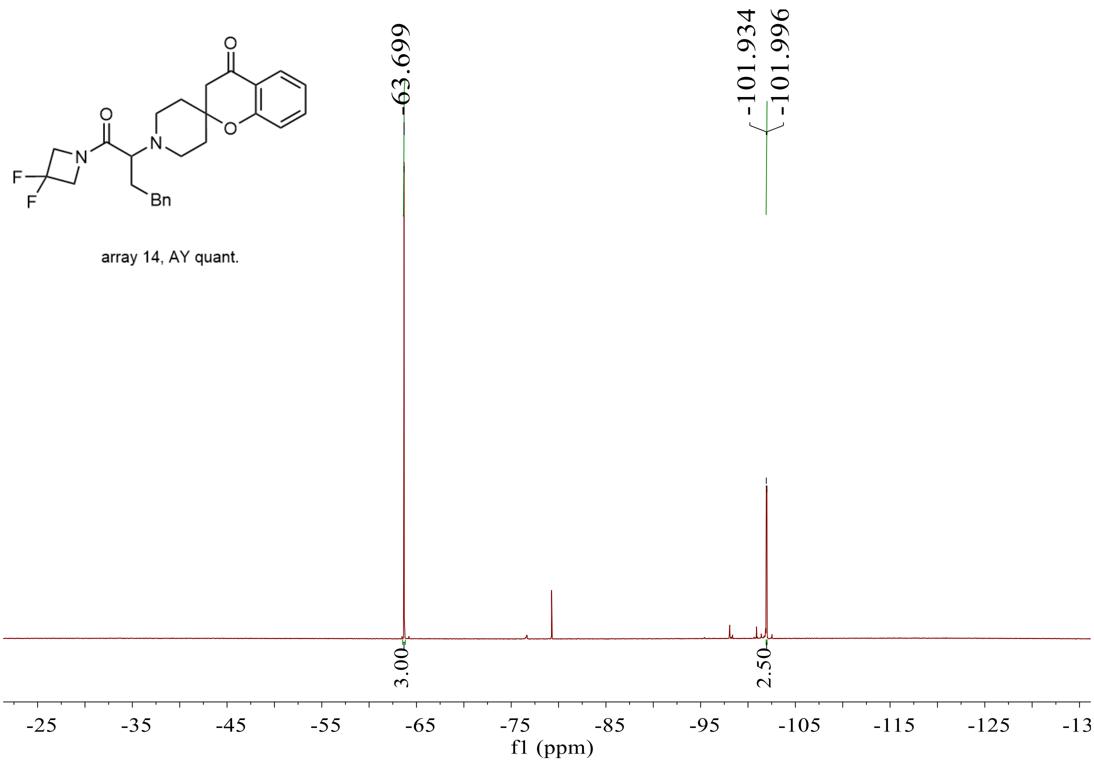

array 14, AY quant.

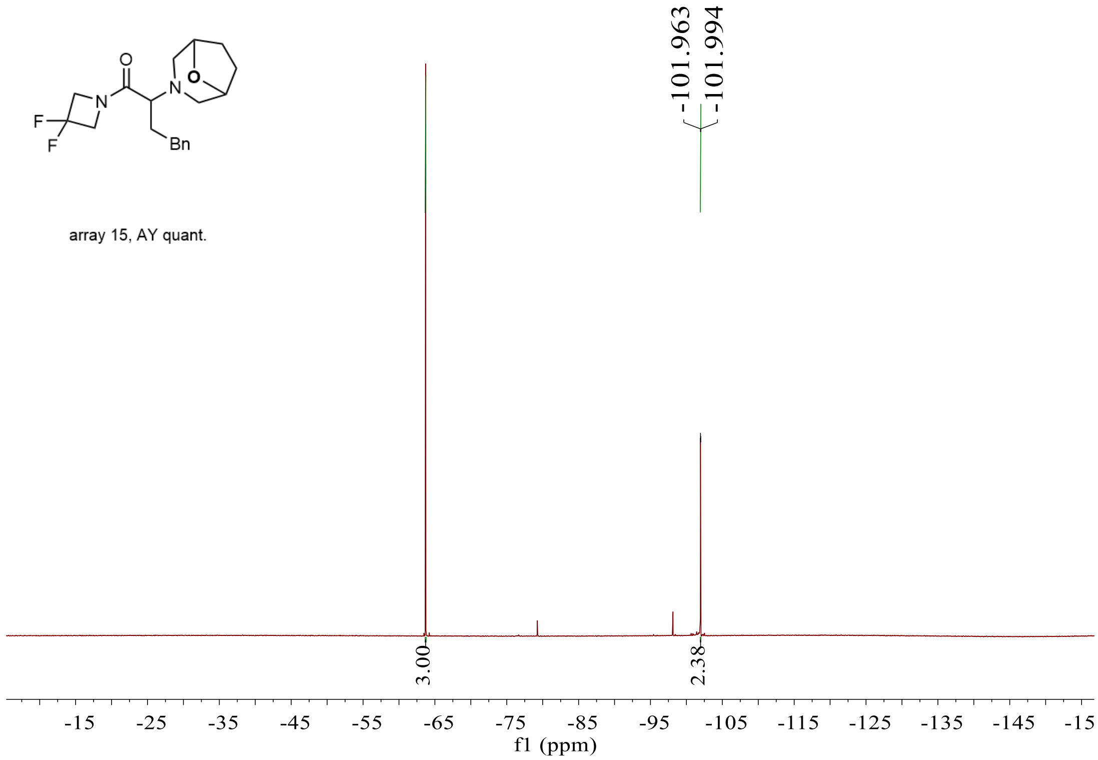

array 15, AY quant.

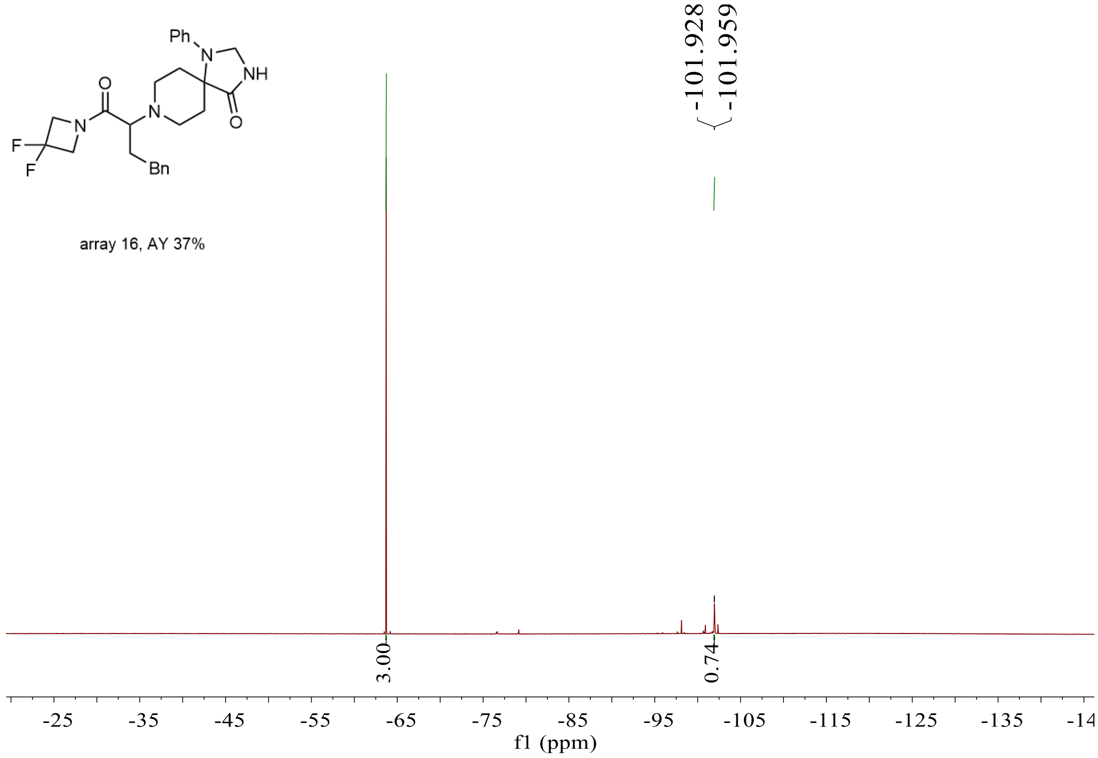

array 16, AY 37%

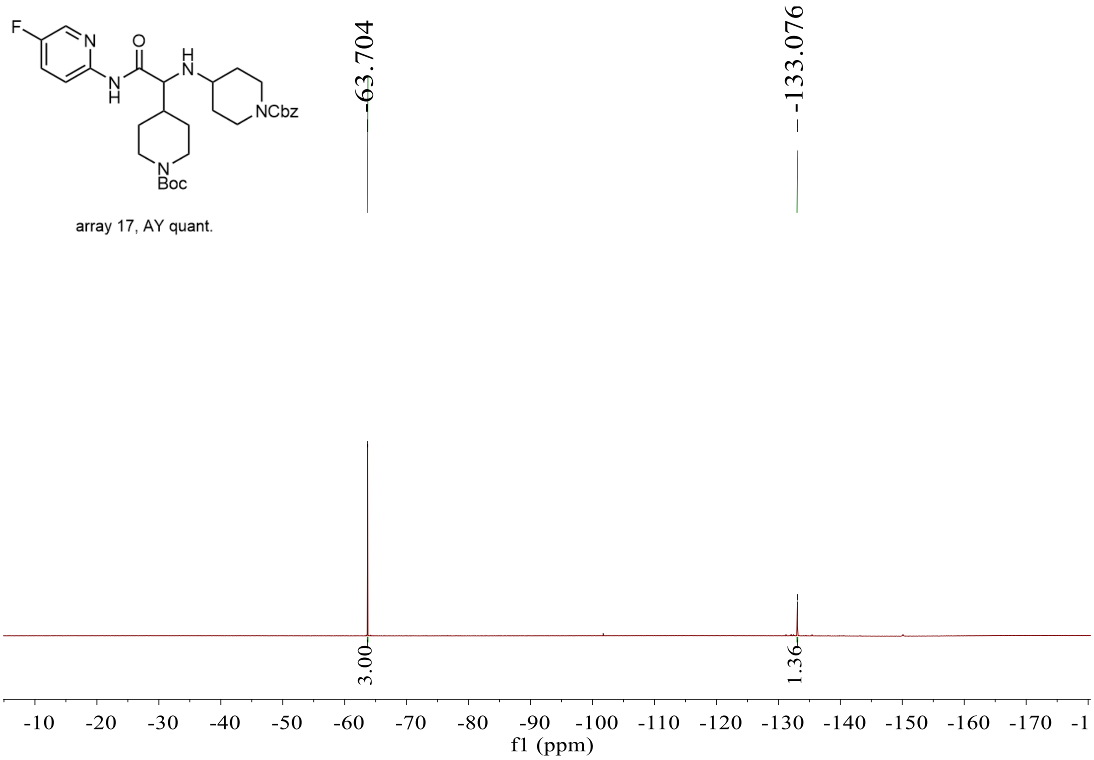

F
N
O
H
N
N
H
NCbz
N
Boc
array 17, AY quant.
-63.704
-133.076
3.00
1.36
-10 -20 -30 -40 -50 -60 -70 -80 -90 -100 -110 -120 -130 -140 -150 -160 -170 -1
f1 (ppm)

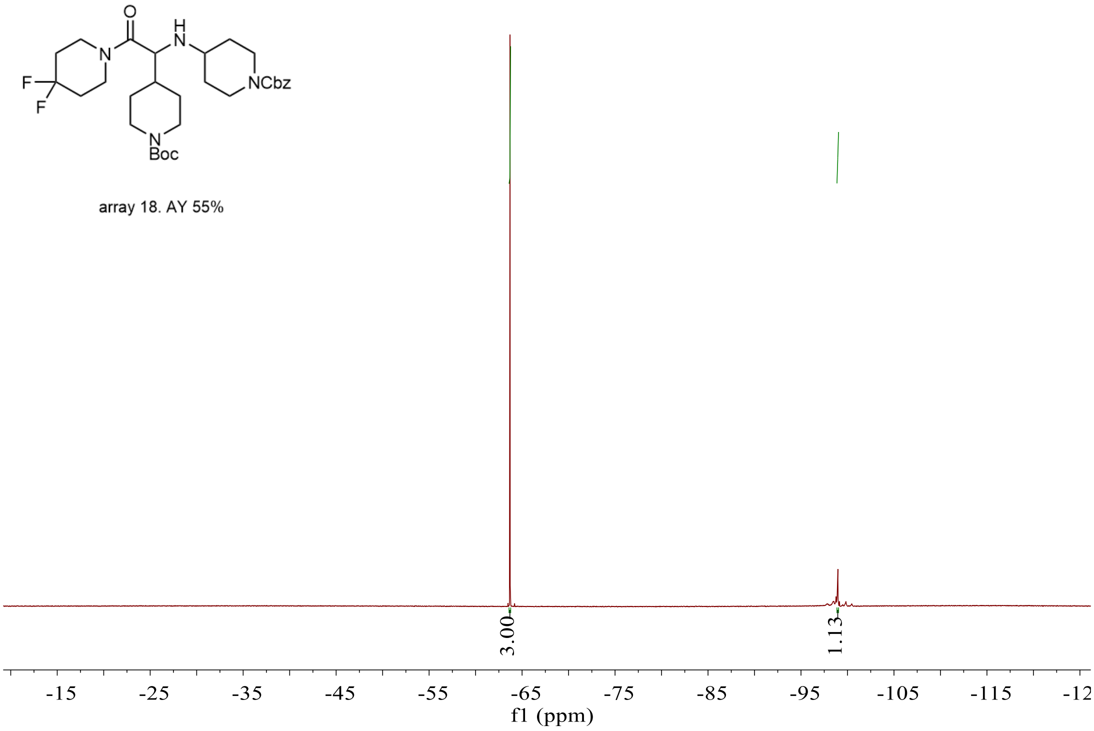

O
H
N
N
F
F
NCbz
N
Boc
array 18. AY 55%
3.00
1.13
-15 -25 -35 -45 -55 -65 -75 -85 -95 -105 -115 -12
f1 (ppm)

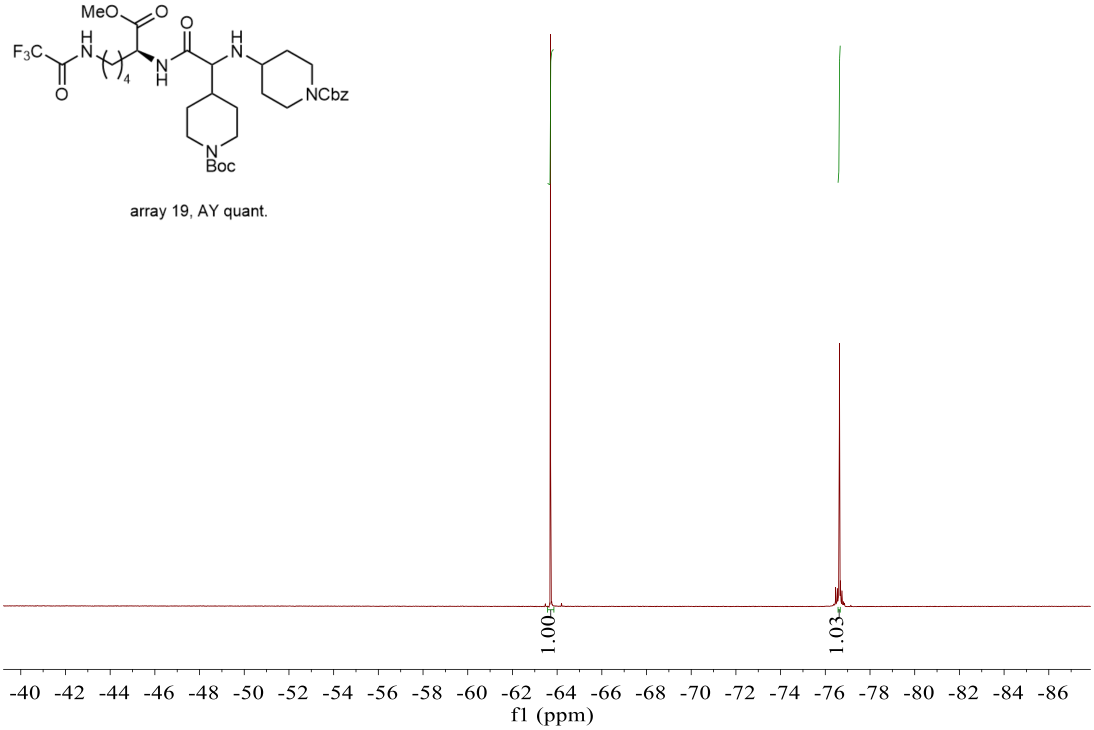

array 19, AY quant.

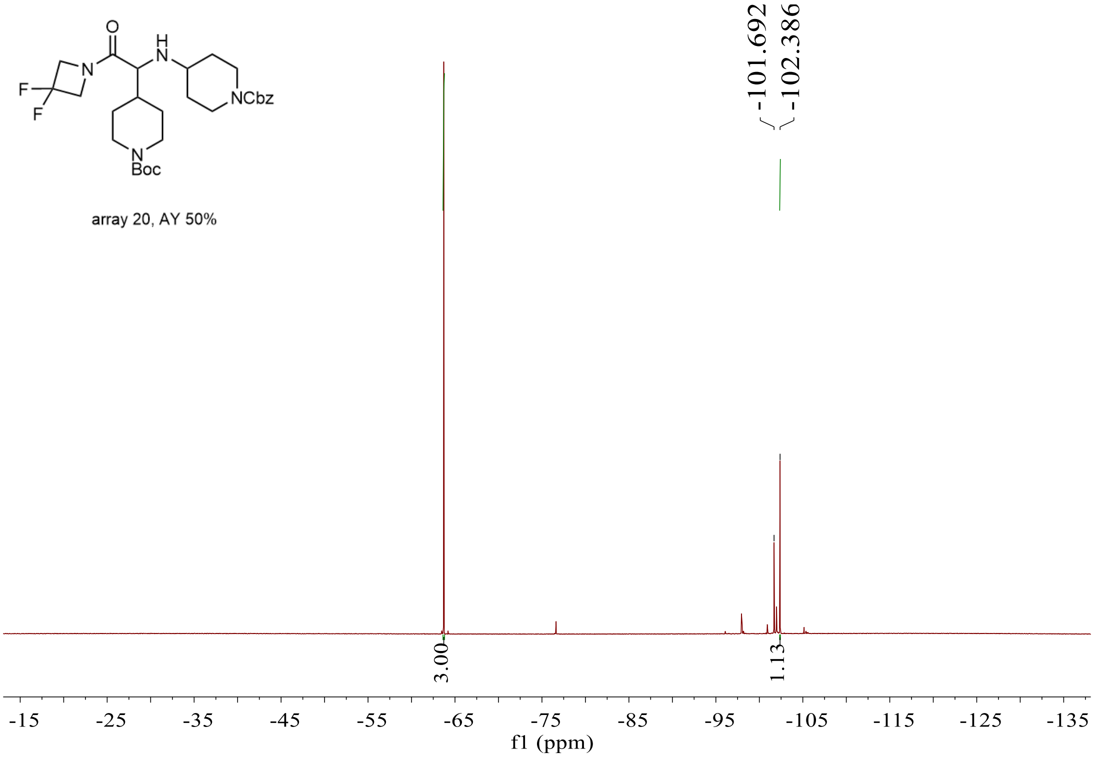

array 20, AY 50%

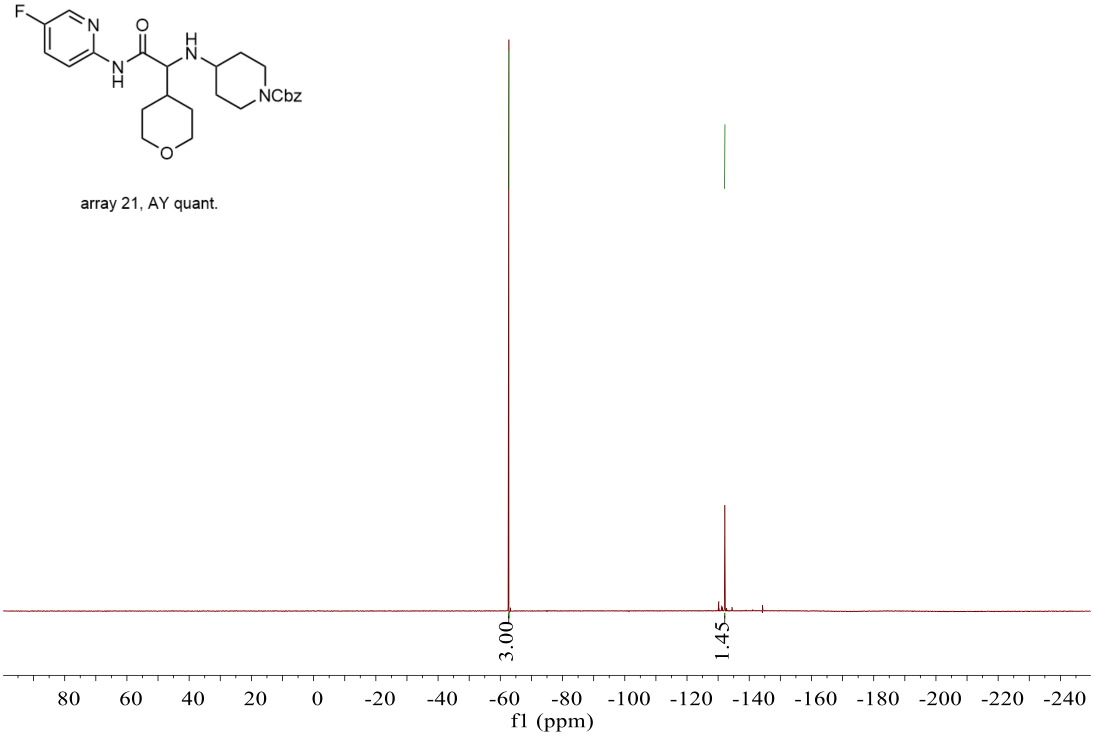

array 21, AY quant.

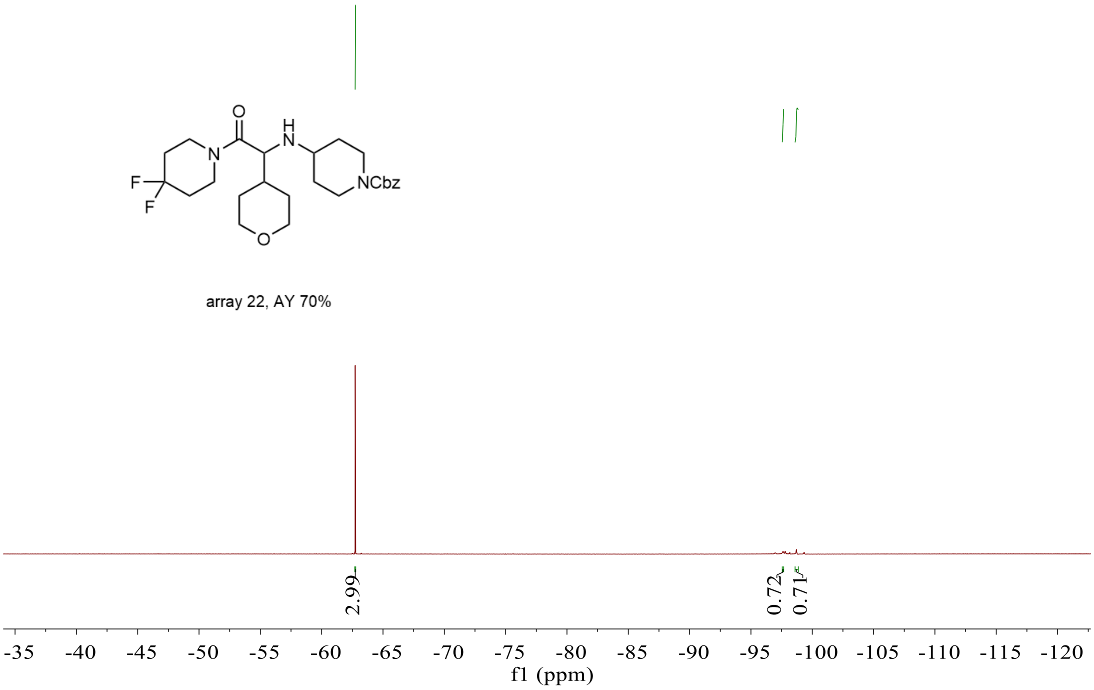

array 22, AY 70%

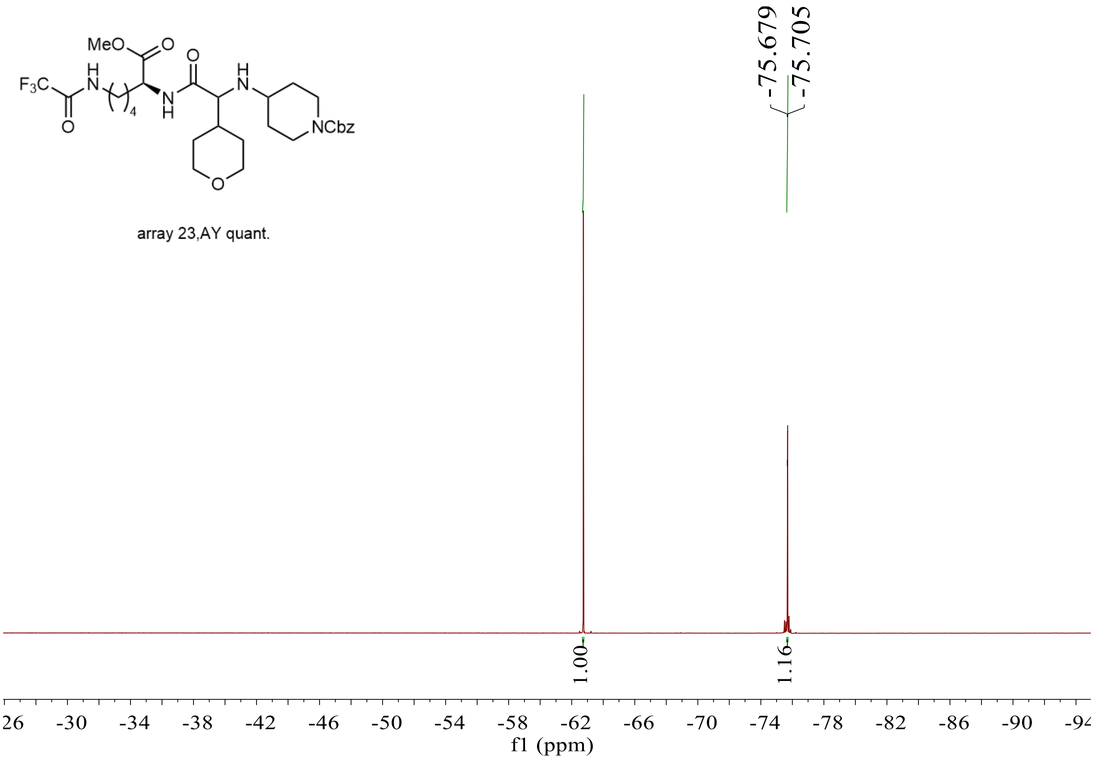
array 23,AY quant.
-75.679
-75.705
1.00
1.16
26 -30 -34 -38 -42 -46 -50 -54 -58 -62 -66 -70 -74 -78 -82 -86 -90
f1 (ppm)

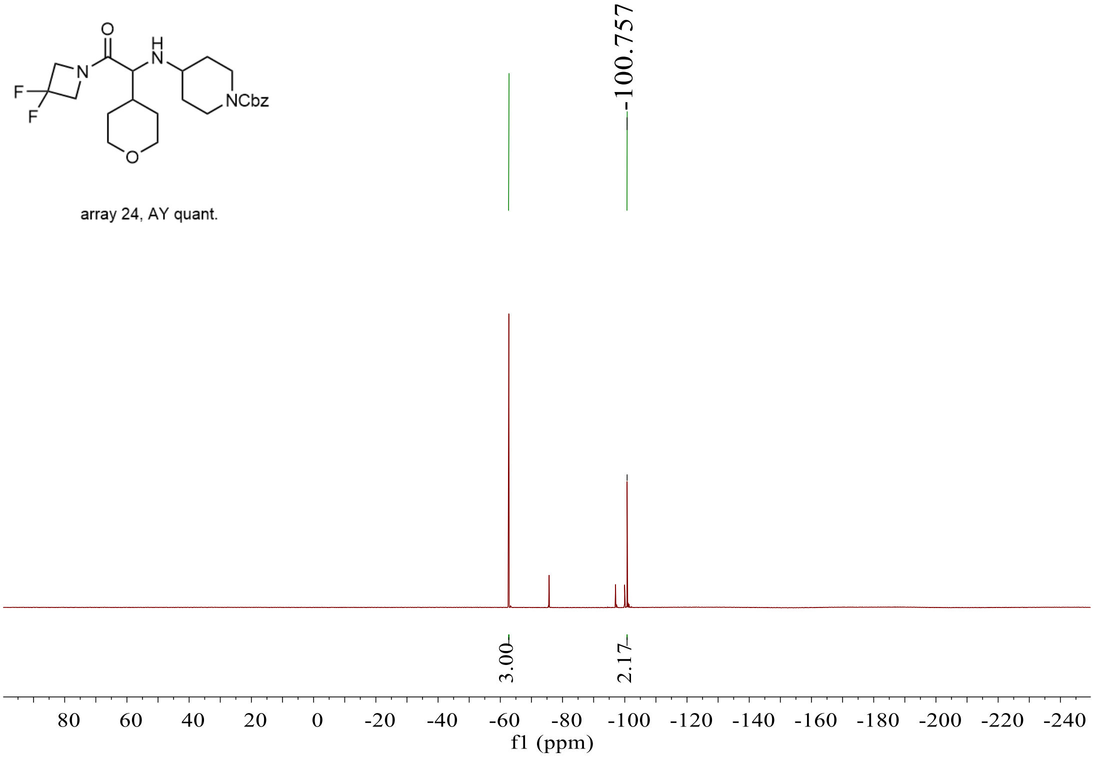
array 24, AY quant.
-100.757
3.00
2.17
80 60 40 20 0 -20 -40 -60 -80 -100 -120 -140 -160 -180 -200 -220 -240
f1 (ppm)

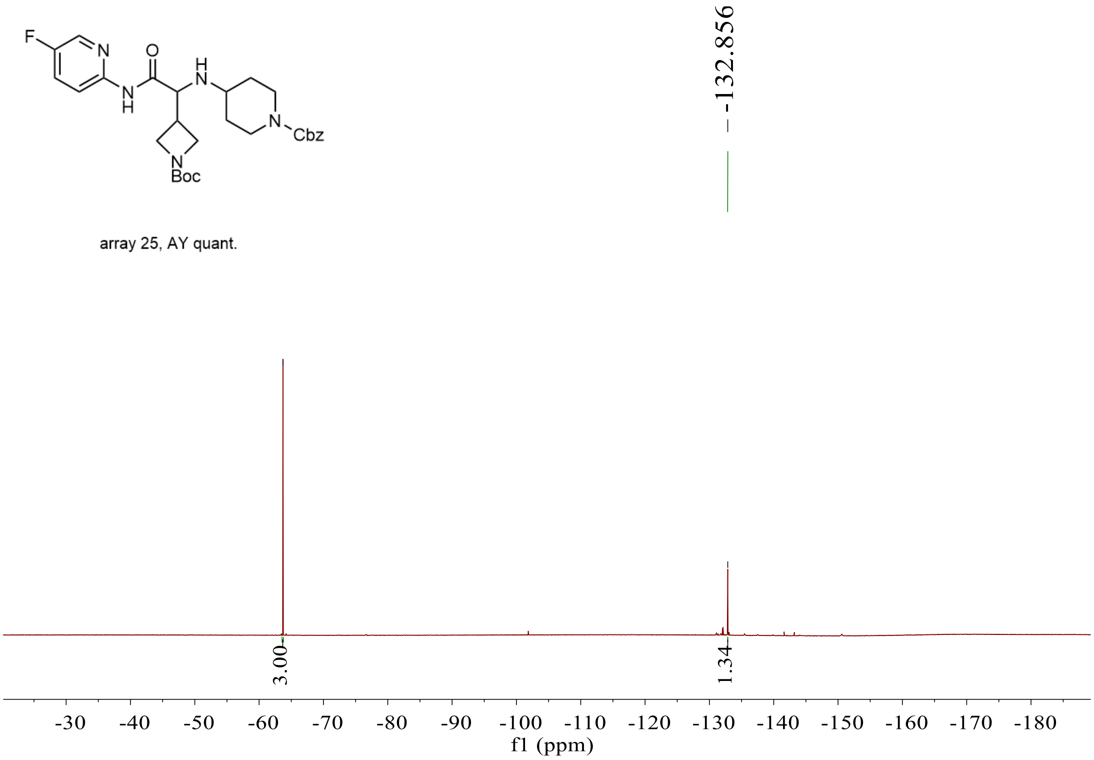

array 25, AY quant.
-132.856
3.00
1.34
-30 -40 -50 -60 -70 -80 -90 -100 -110 -120 -130 -140 -150 -160 -170 -180
f1 (ppm)

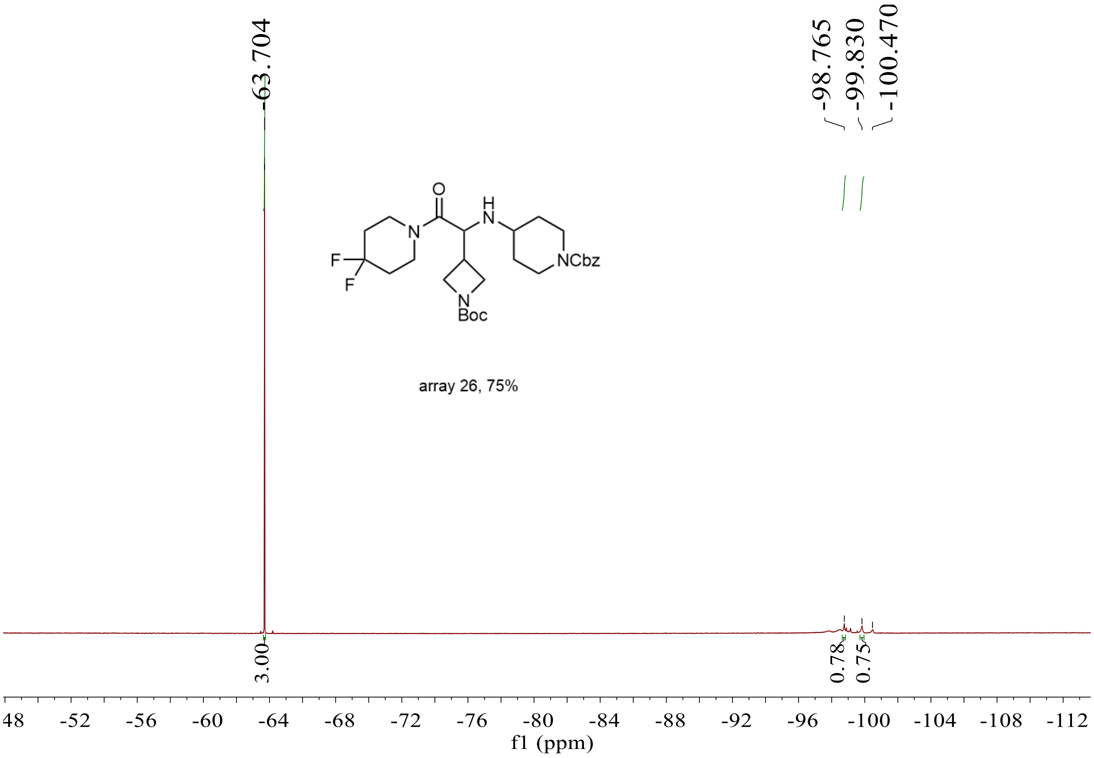

-63.704
-98.765
-99.830
-100.470
array 26, 75%
3.00
0.78
0.75
48 -52 -56 -60 -64 -68 -72 -76 -80 -84 -88 -92 -96 -100 -104 -108 -112
f1 (ppm)

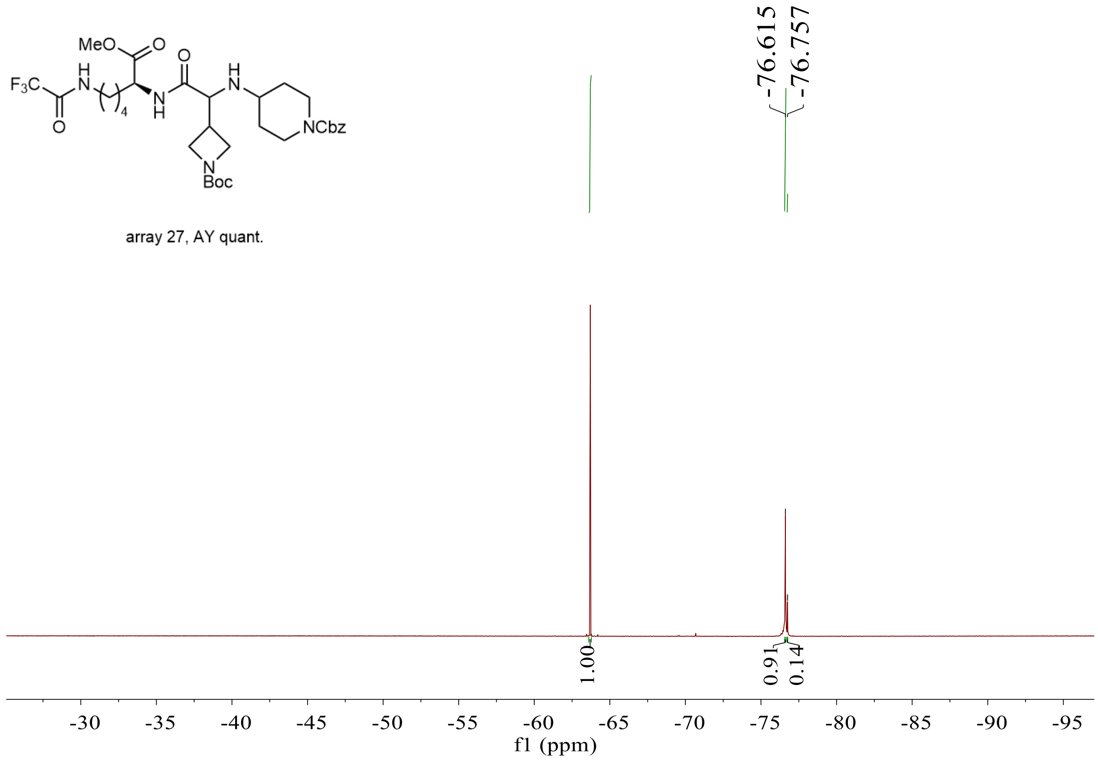
MeO
O
H
N
F3C
O
4
H
N
O
H
N
NCbz
N
Boc
array 27, AY quant.
-76.615
-76.757
1.00
0.91
0.14
-30
-35
-40
-45
-50
-55
-60
-65
-70
-75
-80
-85
-90
-95
f1 (ppm)

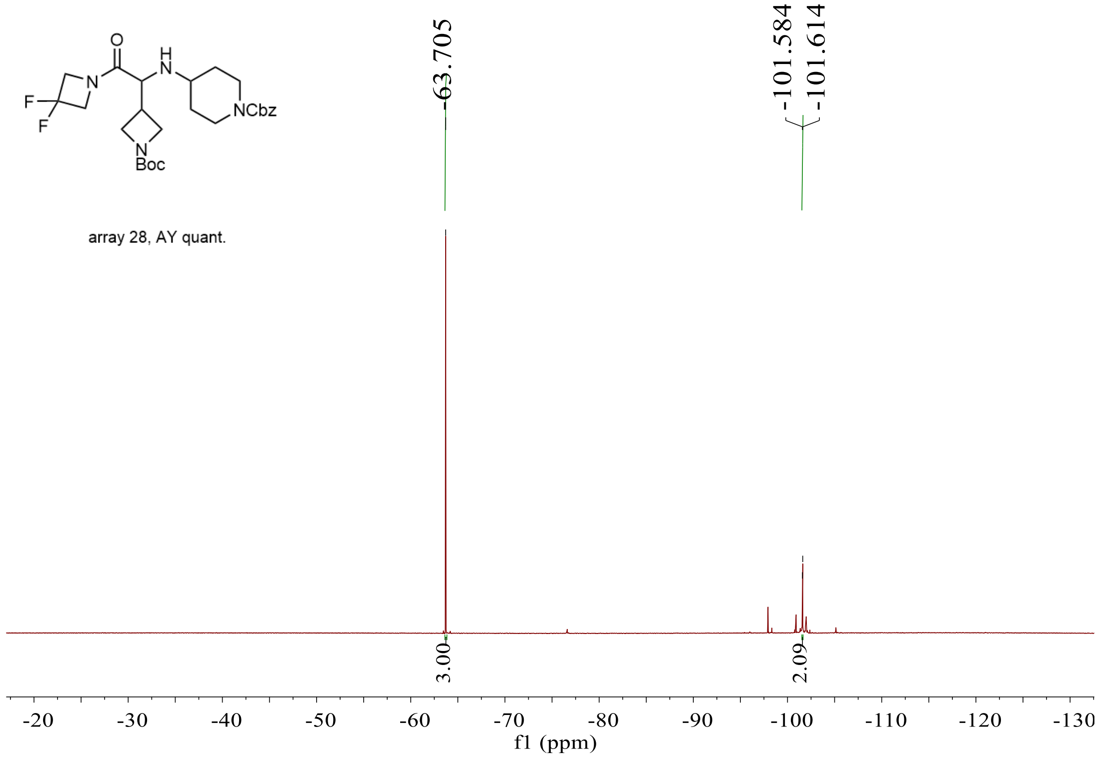
O
H
N
F
F
N
NCbz
N
Boc
array 28, AY quant.
-63.705
-101.584
-101.614
3.00
2.09
-20
-30
-40
-50
-60
-70
-80
-90
-100
-110
-120
-130
f1 (ppm)

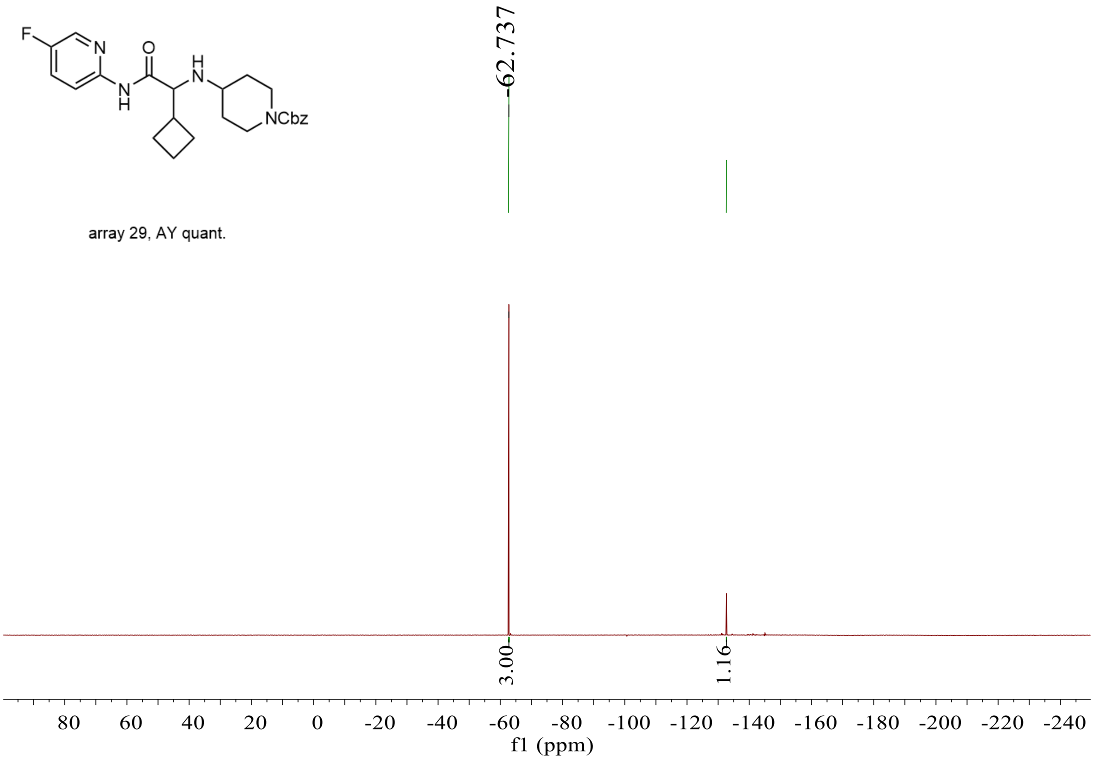

array 29, AY quant.

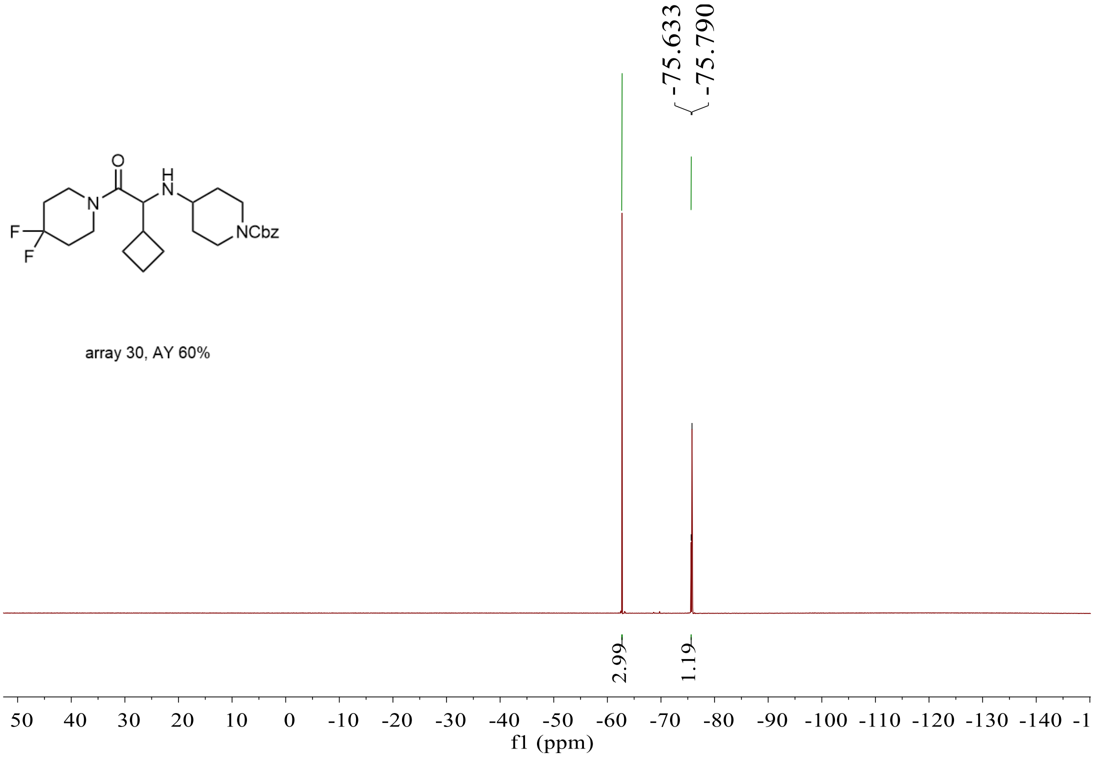

array 30, AY 60%

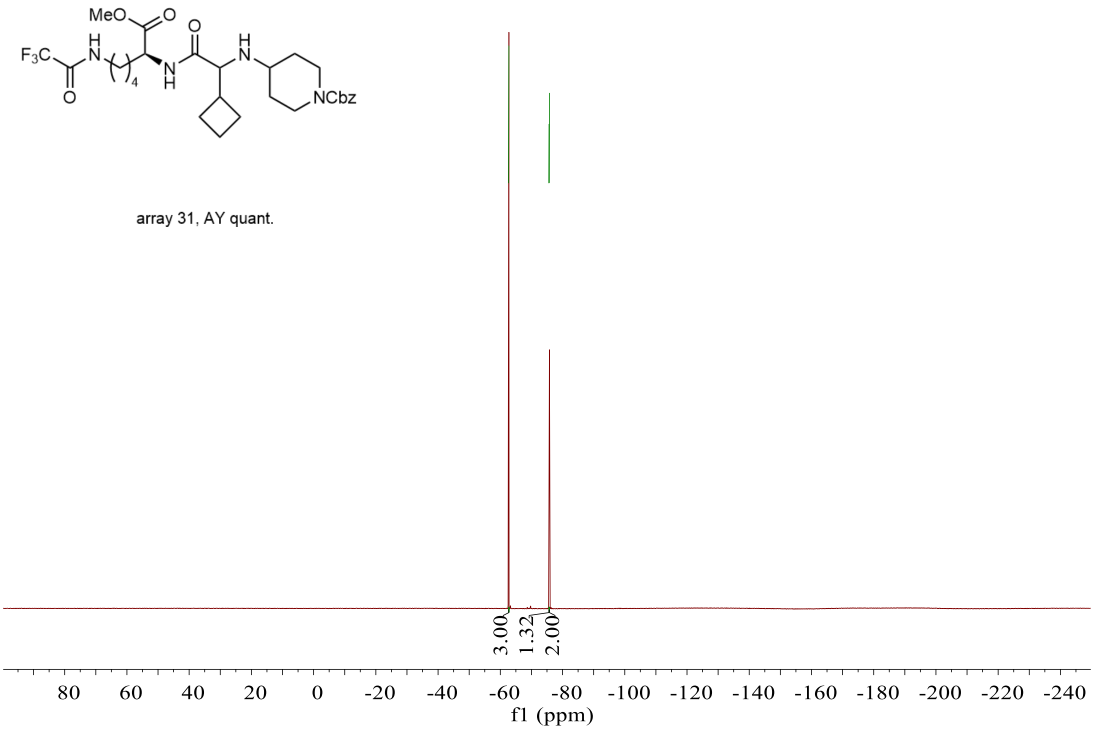
MeO
F3C
NCbz
array 31, AY quant.
3.00
1.32
2.00
80 60 40 20 0 -20 -40 -60 -80 -100 -120 -140 -160 -180 -200 -220 -240
f1 (ppm)

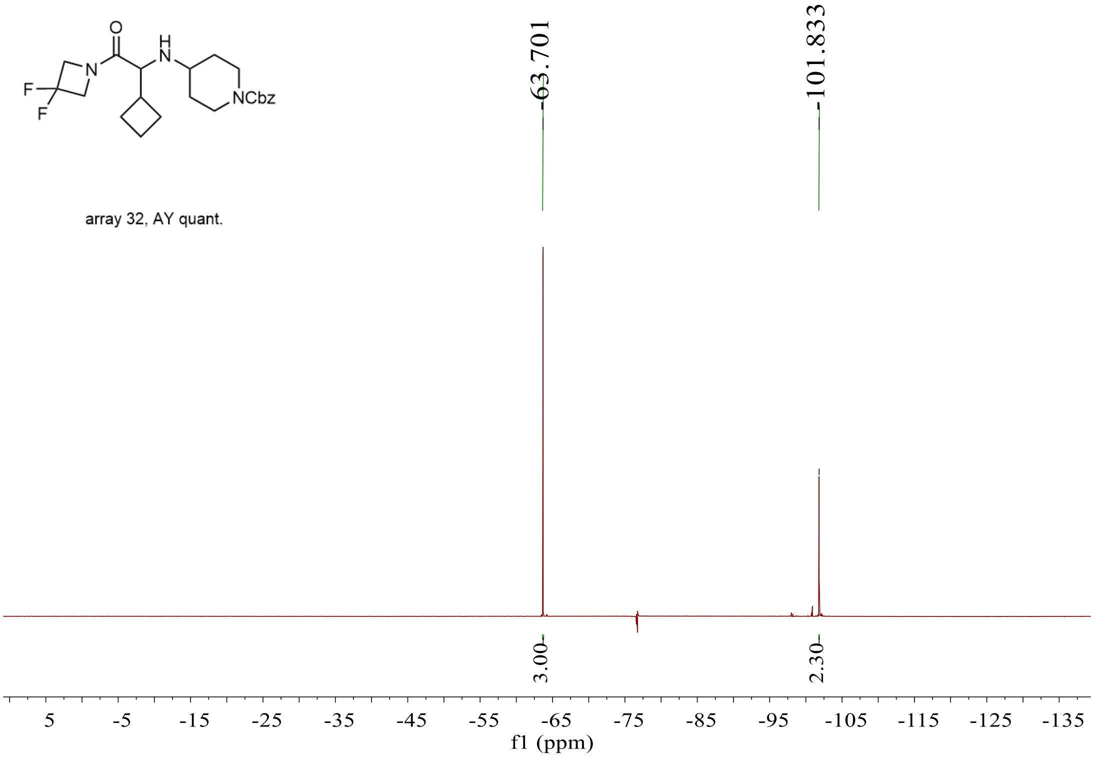
F
F
NCbz
array 32, AY quant.
-63.701
-101.833
3.00
2.30
5 -5 -15 -25 -35 -45 -55 -65 -75 -85 -95 -105 -115 -125 -135
f1 (ppm)

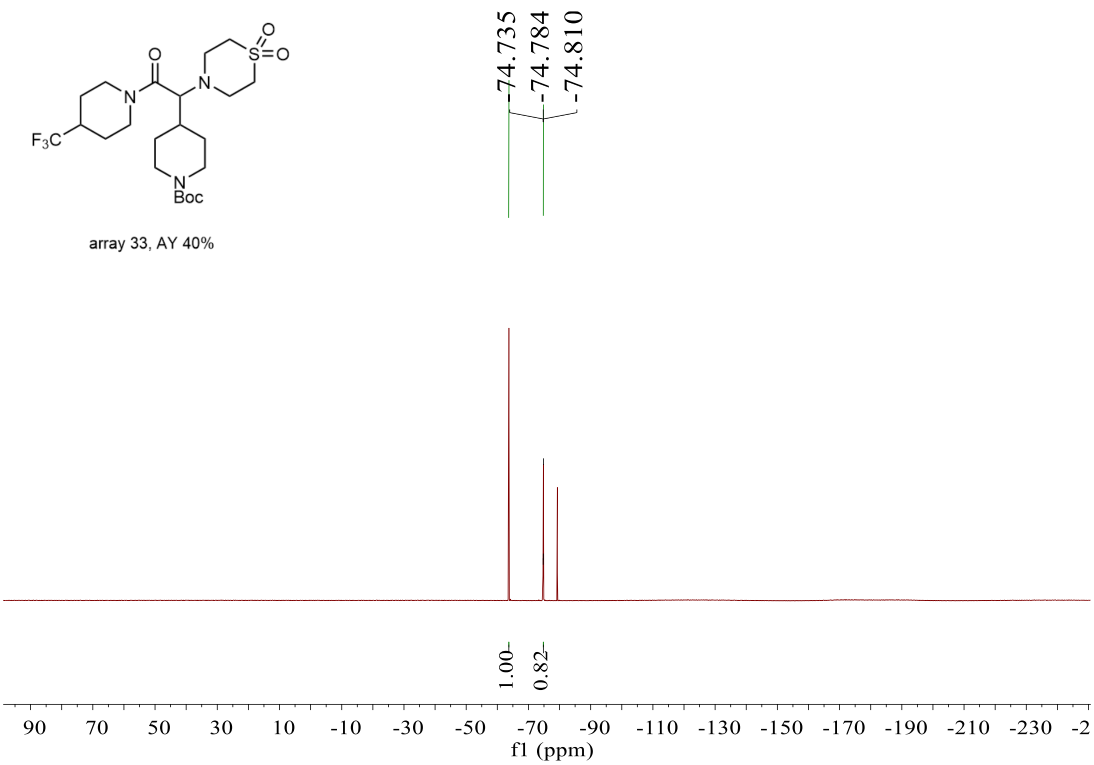

array 33, AY 40%

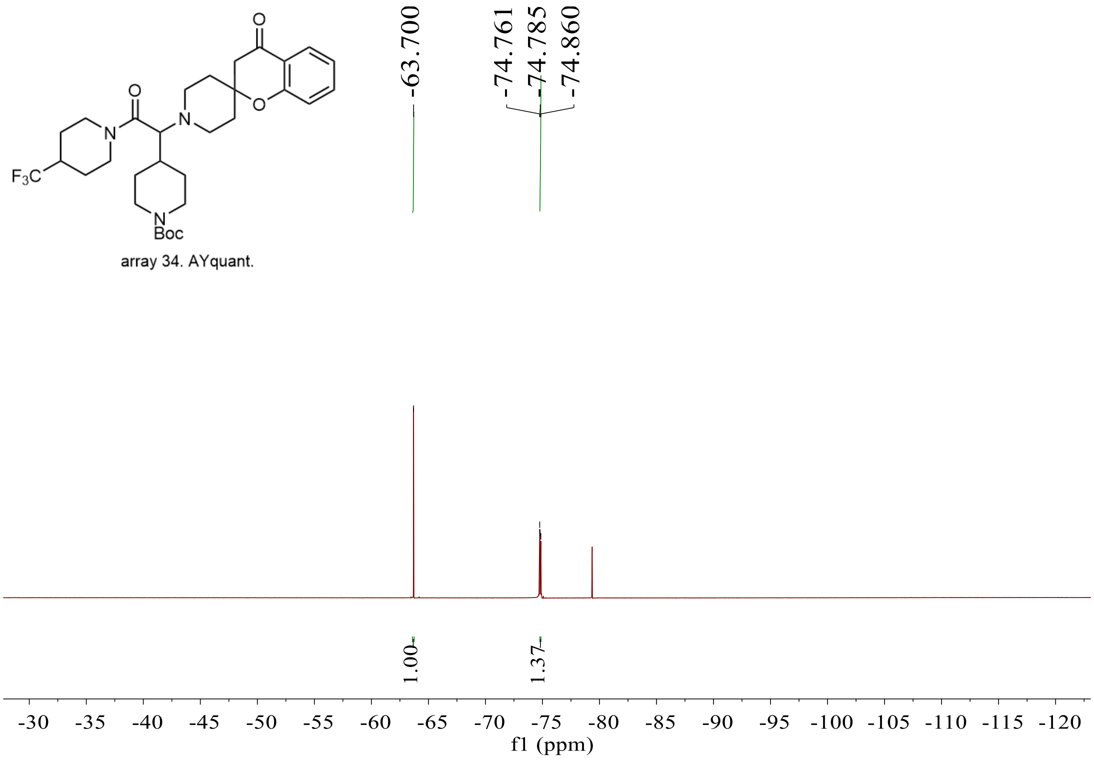

array 34. AYquant.

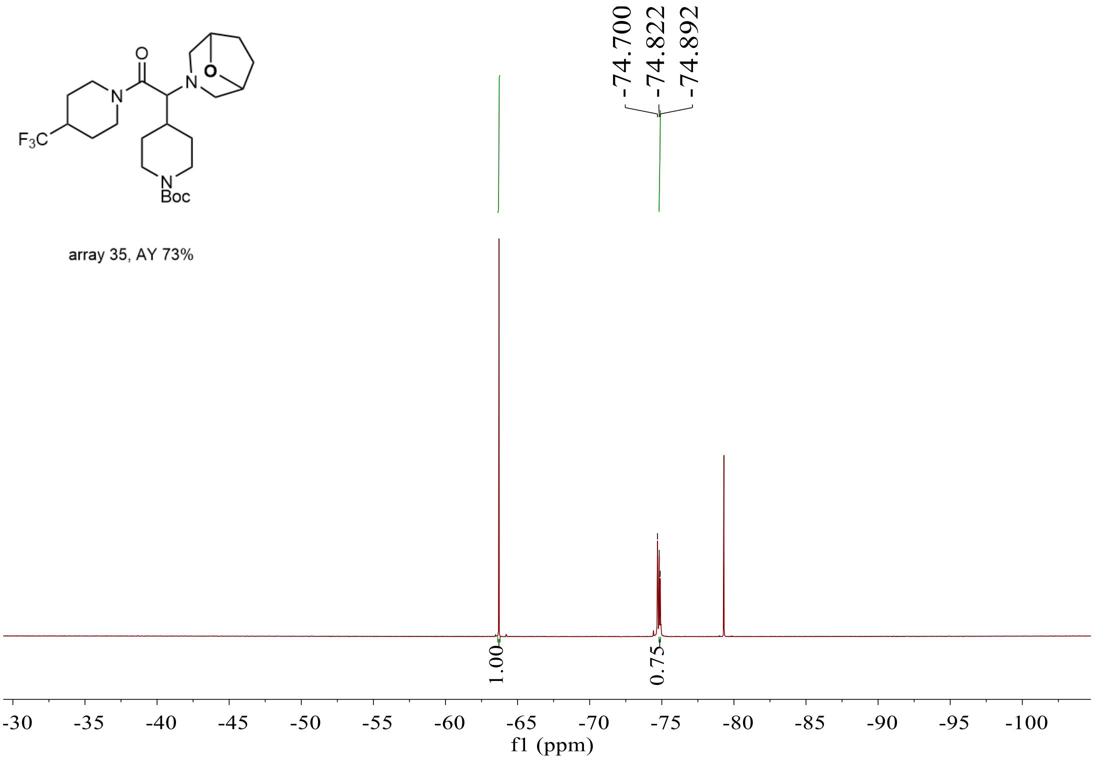

array 35, AY 73%

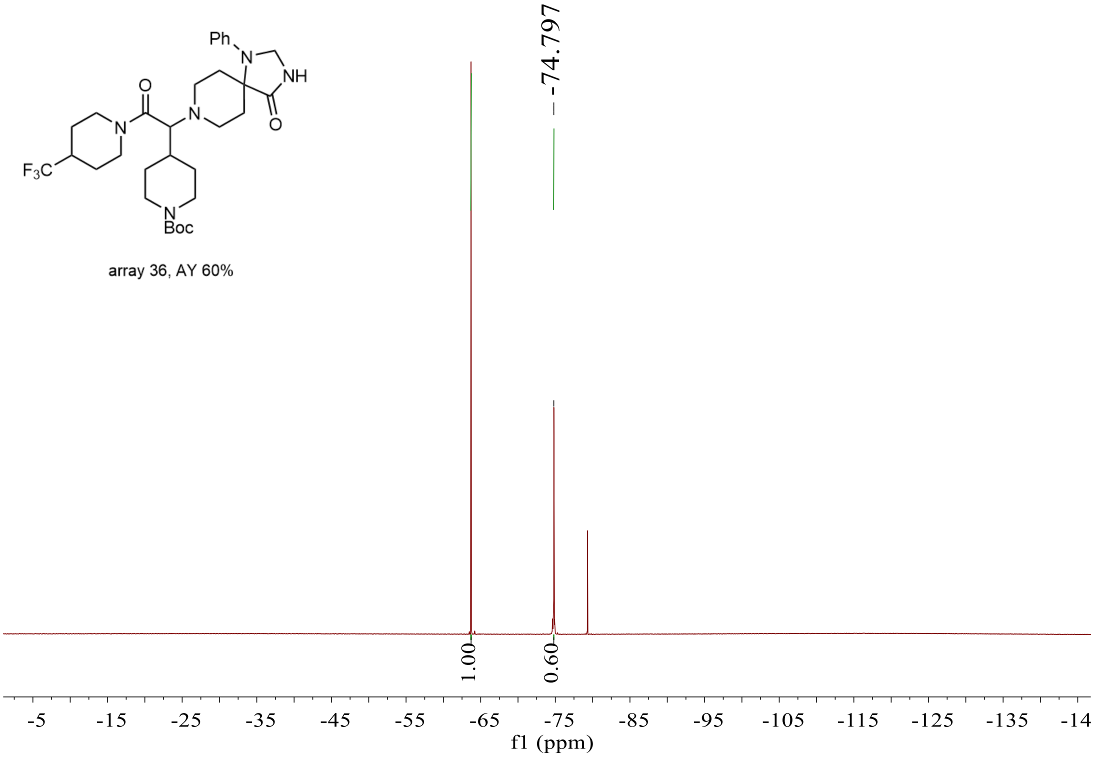

array 36, AY 60%

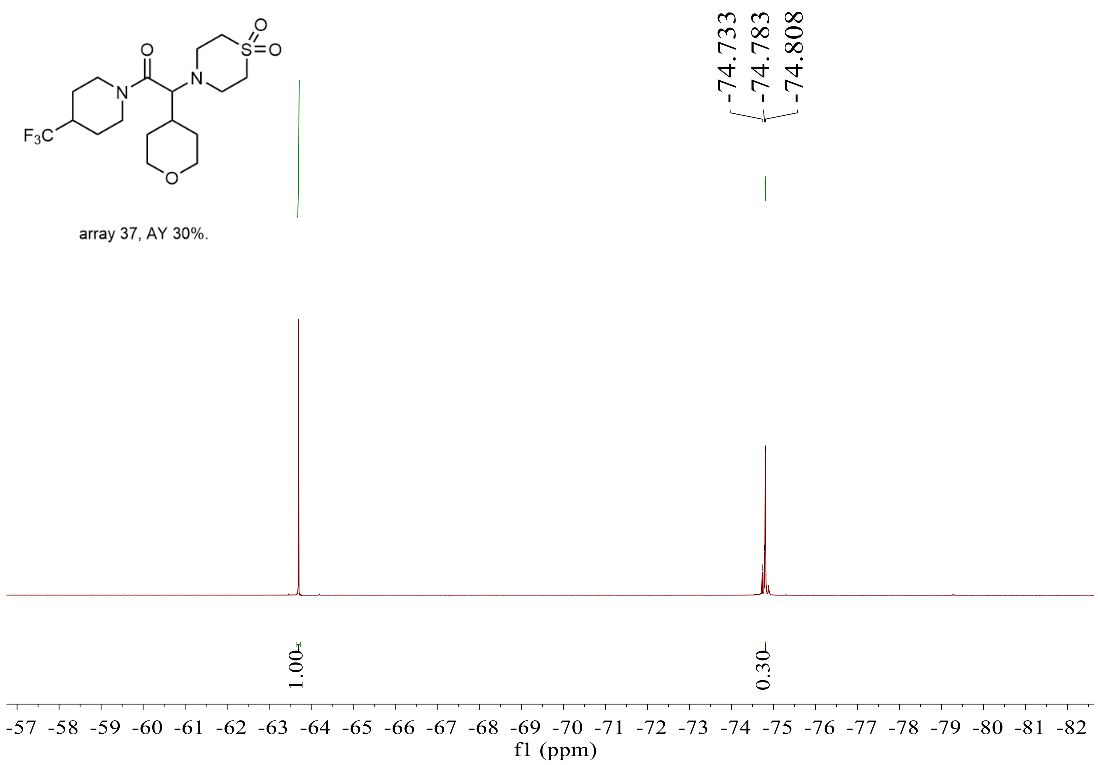
F3C
array 37, AY 30%.
-74.733
-74.783
-74.808
1.00
0.30
-57 -58 -59 -60 -61 -62 -63 -64 -65 -66 -67 -68 -69 -70 -71 -72 -73 -74 -75 -76 -77 -78 -79 -80 -81 -82
f1 (ppm)

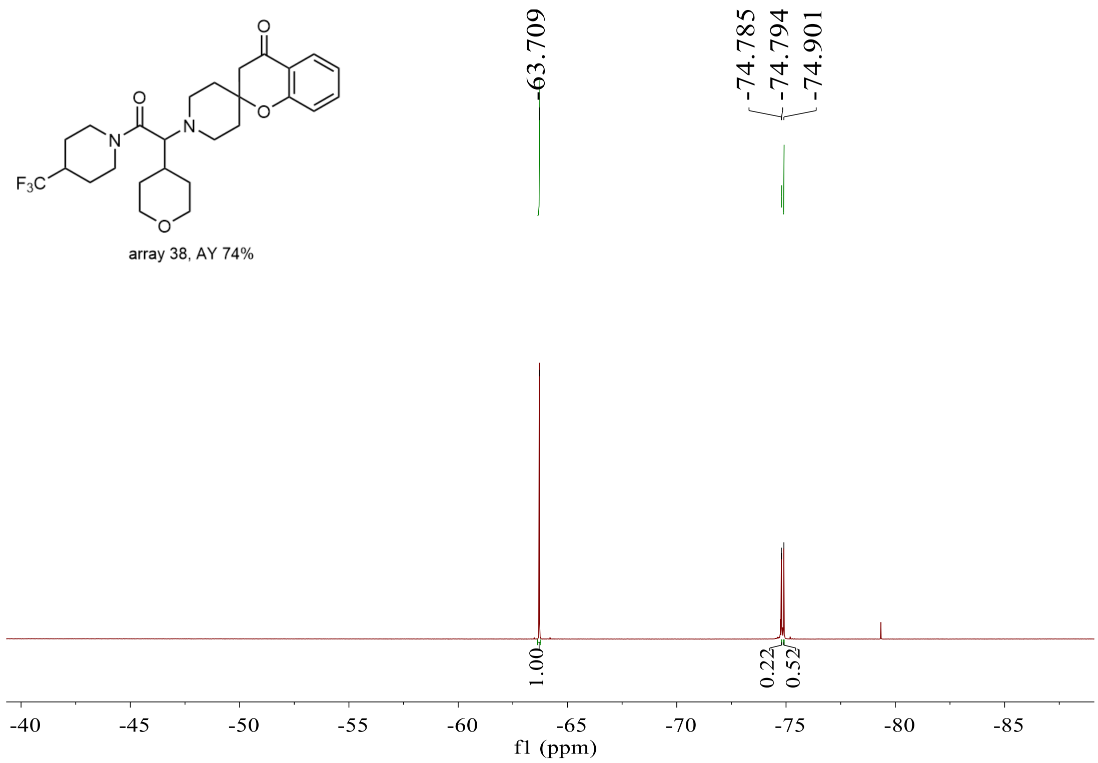
F3C
array 38, AY 74%
-63.709
-74.785
-74.794
-74.901
1.00
0.22
0.52
-40 -45 -50 -55 -60 -65 -70 -75 -80 -85
f1 (ppm)

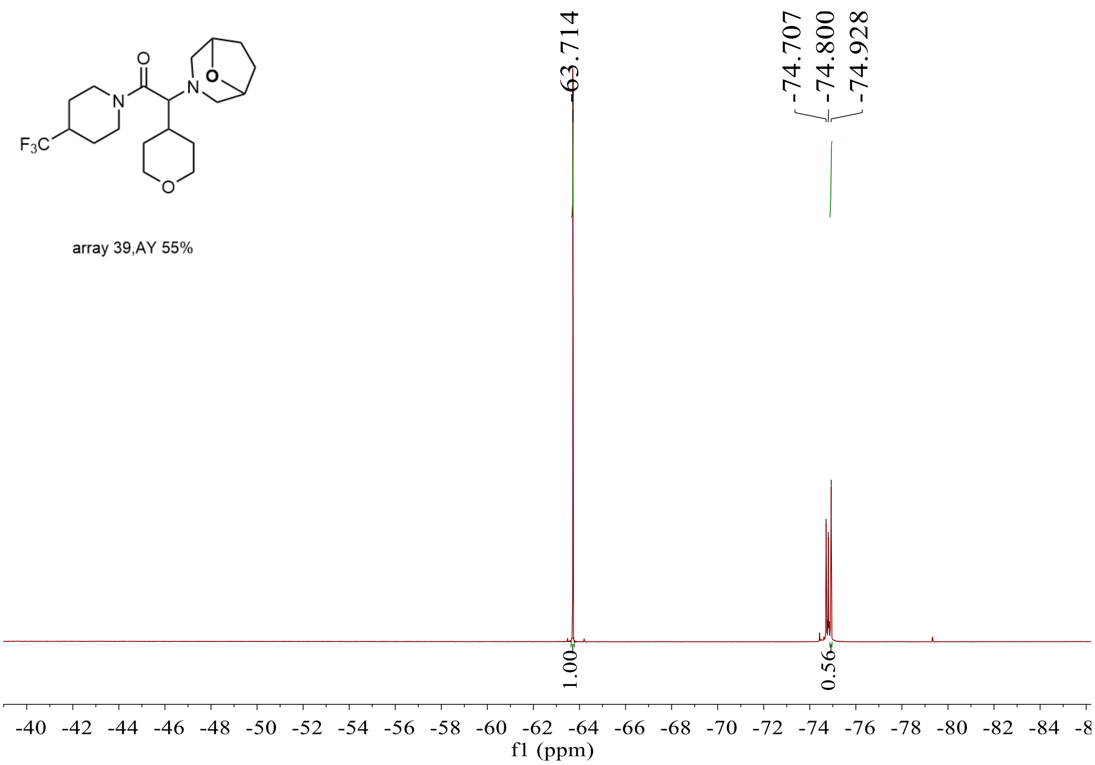
F3C
array 39,AY 55%
-63.714
-74.707
-74.800
-74.928
1.00
0.56
-40 -42 -44 -46 -48 -50 -52 -54 -56 -58 -60 -62 -64 -66 -68 -70 -72 -74 -76 -78 -80 -82 -84
f1 (ppm)

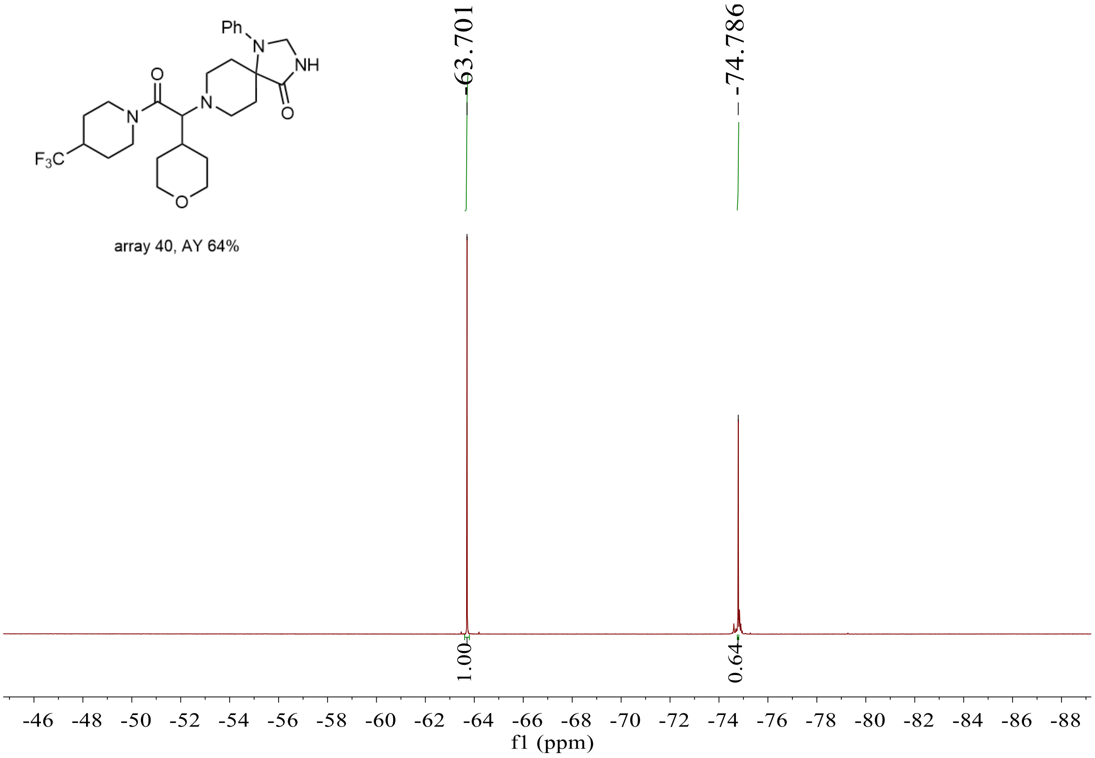
Ph
NH
F3C
array 40, AY 64%
-63.701
-74.786
1.00
0.64
-46 -48 -50 -52 -54 -56 -58 -60 -62 -64 -66 -68 -70 -72 -74 -76 -78 -80 -82 -84 -86 -88
f1 (ppm)

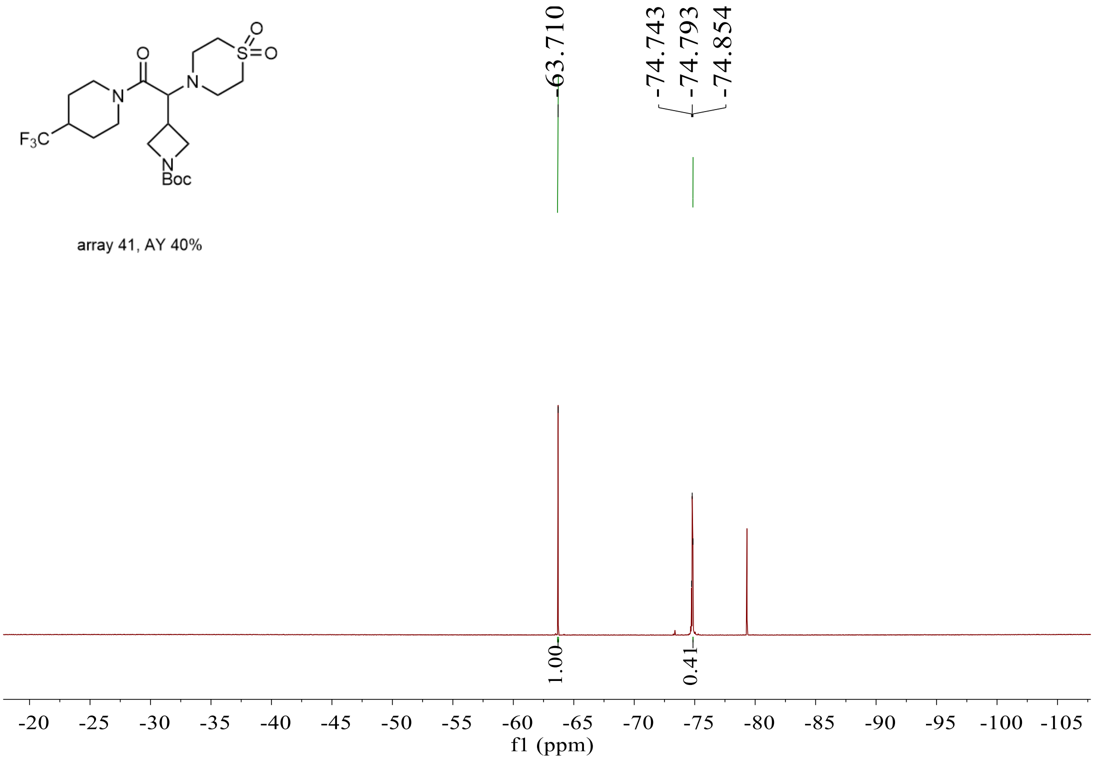

array 41, AY 40%

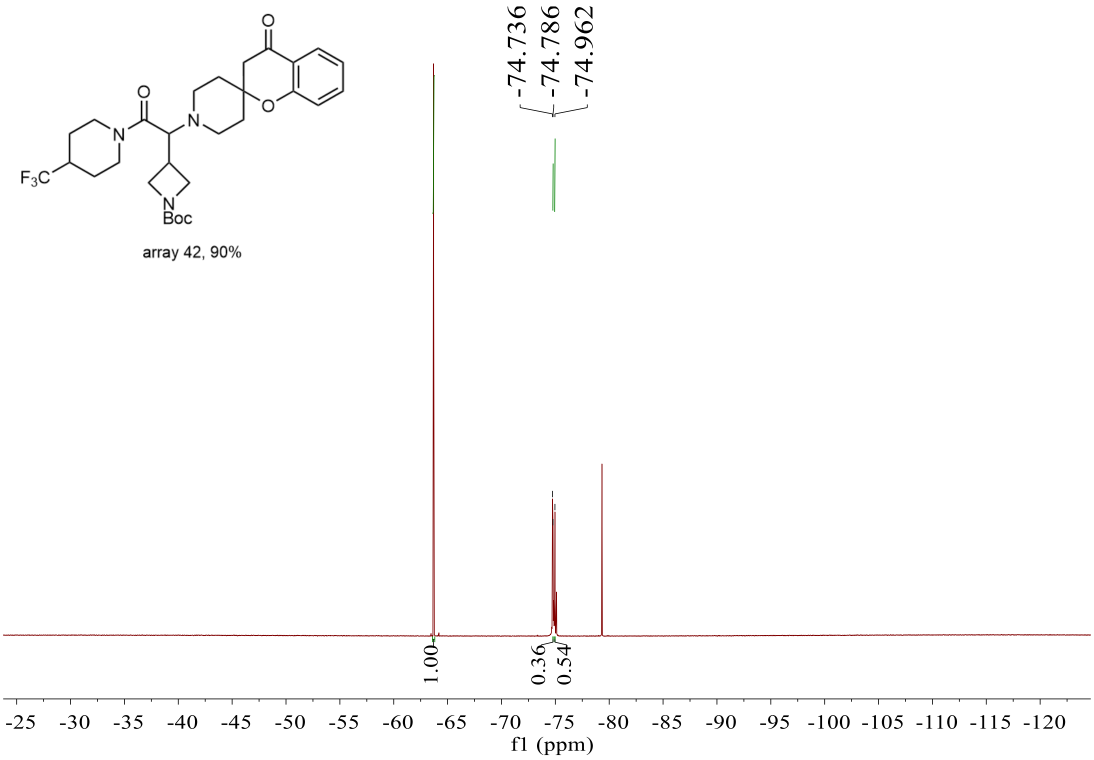

array 42, 90%

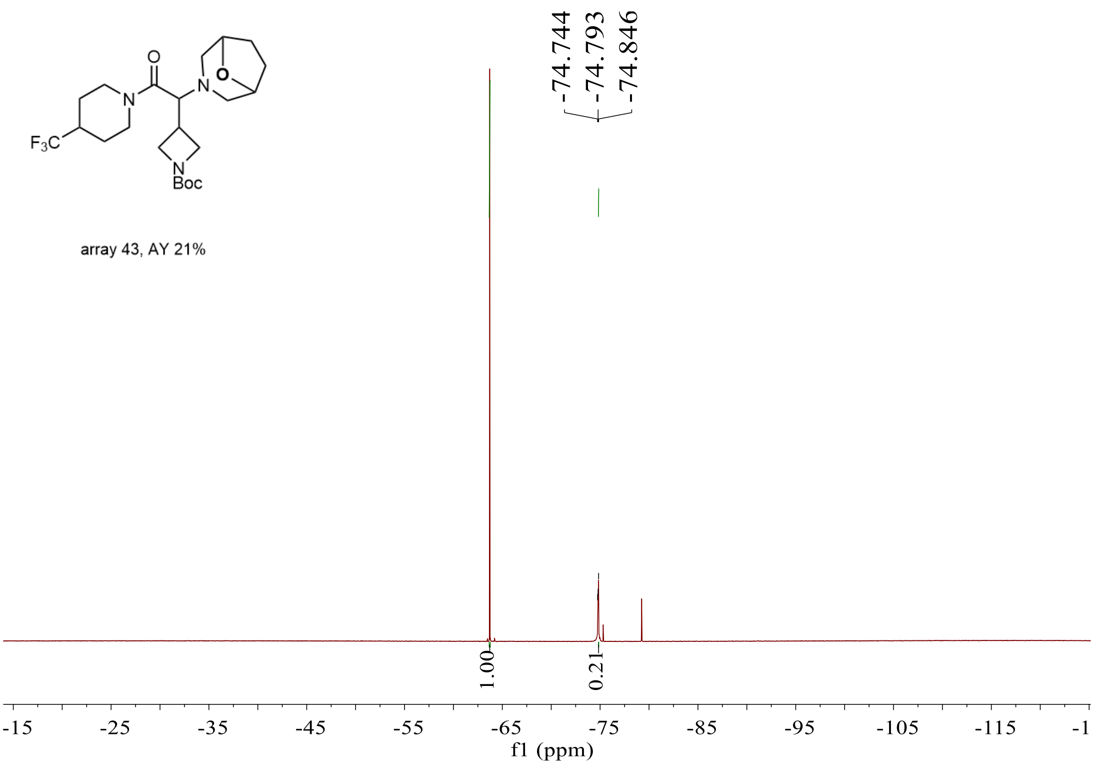
-74.744
-74.793
-74.846
F3C
O
N
N
O
N
Boc
array 43, AY 21%
1.00
0.21
-15 -25 -35 -45 -55 -65 -75 -85 -95 -105 -115 -1
f1 (ppm)

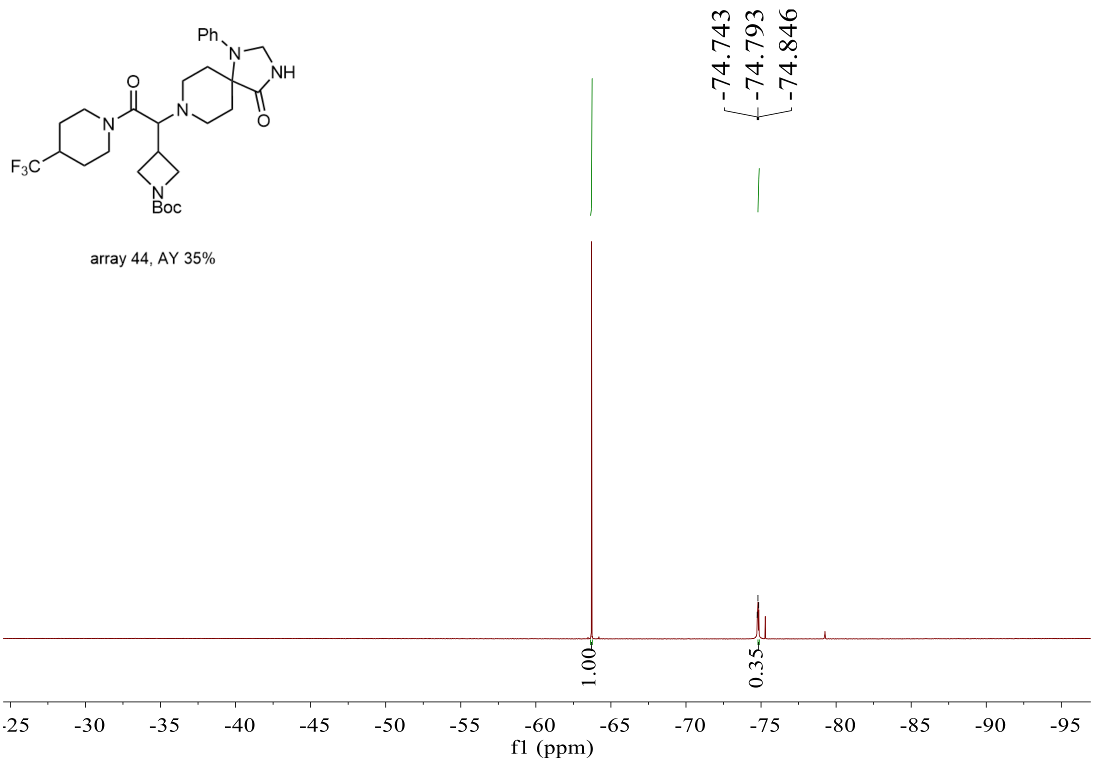
-74.743
-74.793
-74.846
Ph
N
NH
O
N
N
O
F3C
N
Boc
array 44, AY 35%
1.00
0.35
-25 -30 -35 -40 -45 -50 -55 -60 -65 -70 -75 -80 -85 -90 -95
f1 (ppm)

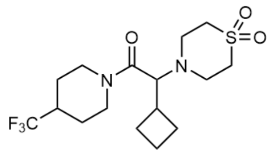

array 45, AY 60%

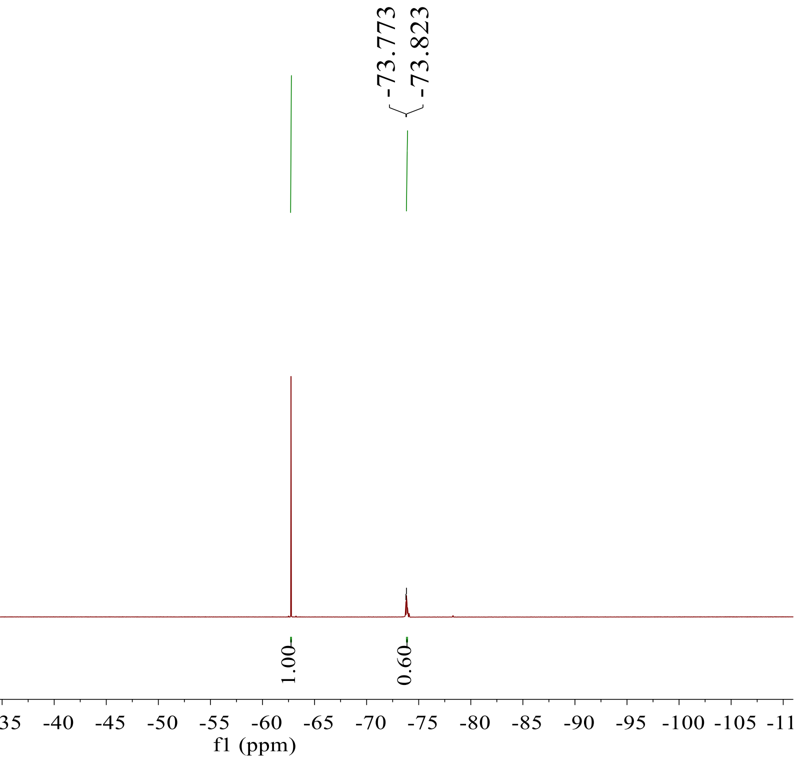

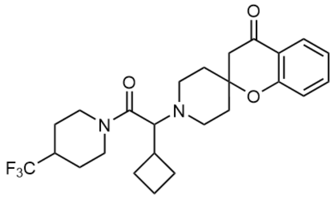

array 46, AY quant.

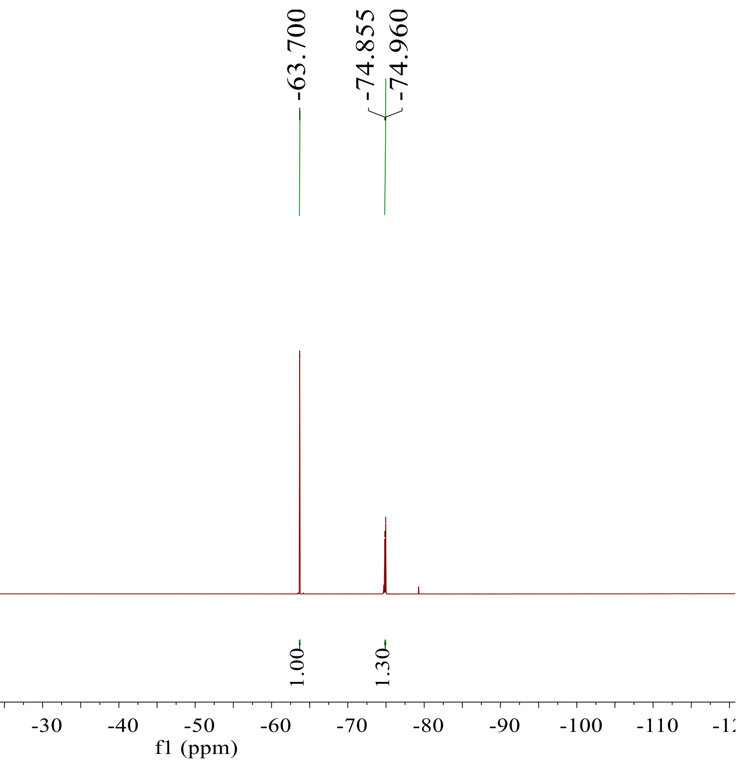

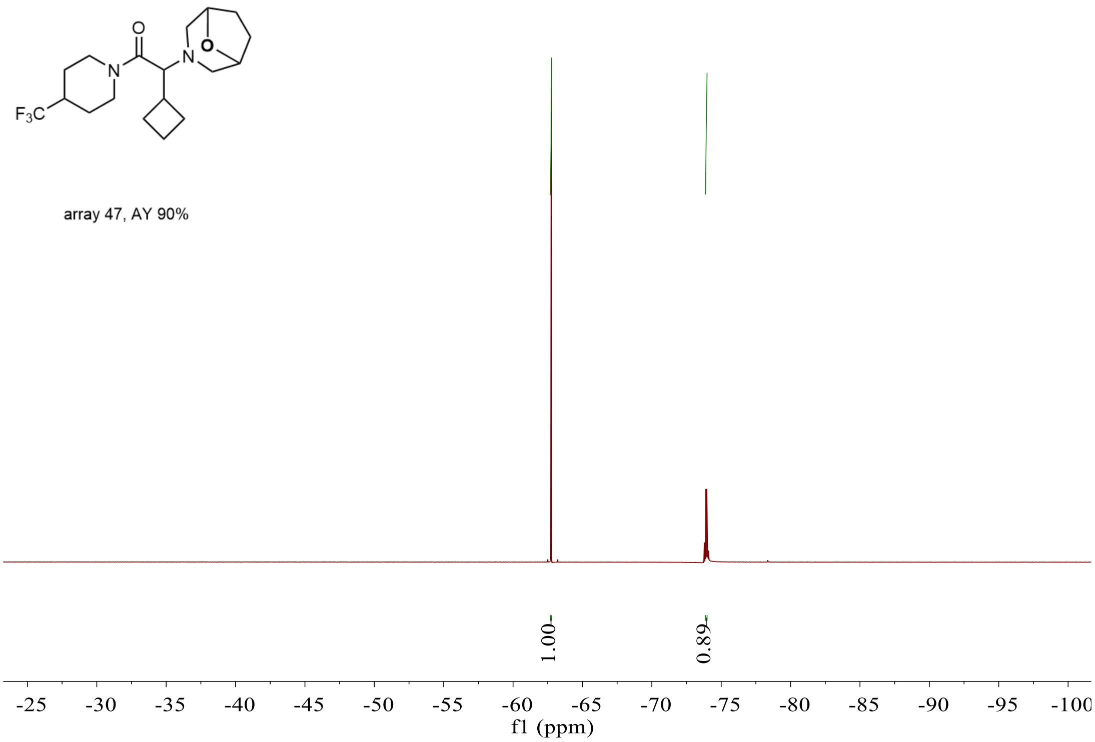

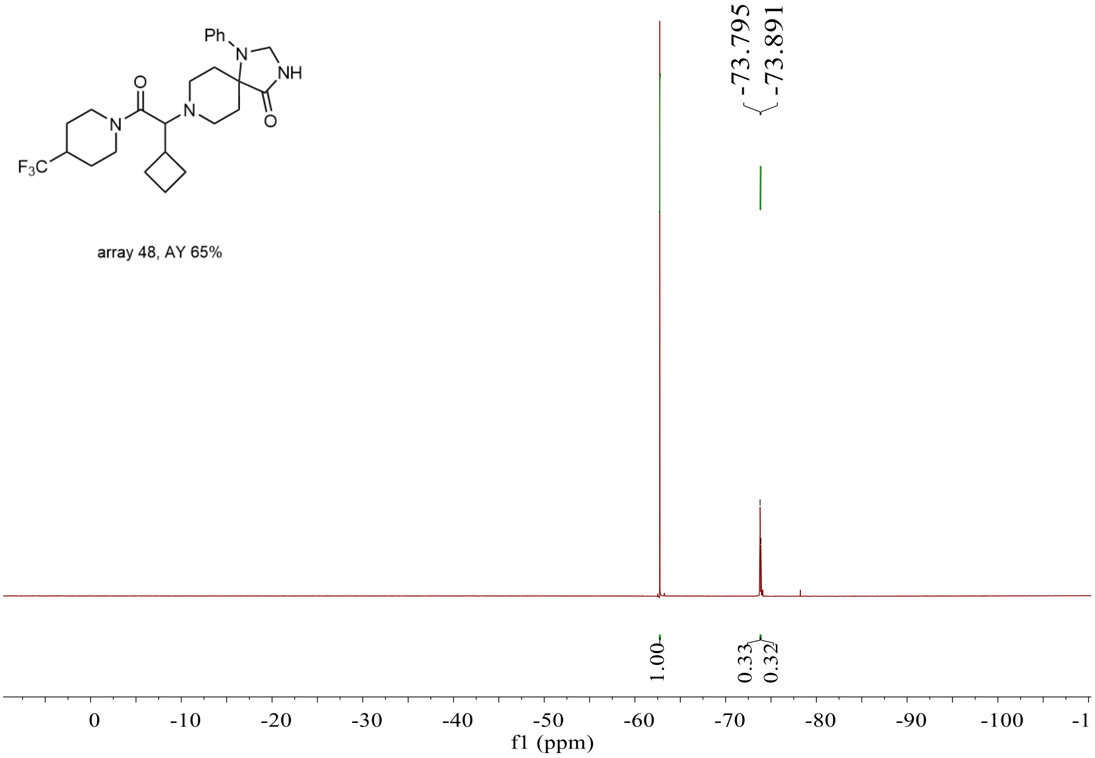

Spectra of the array library

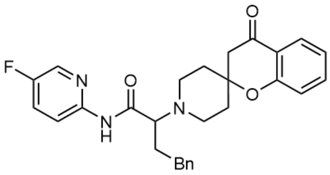

2
array 1

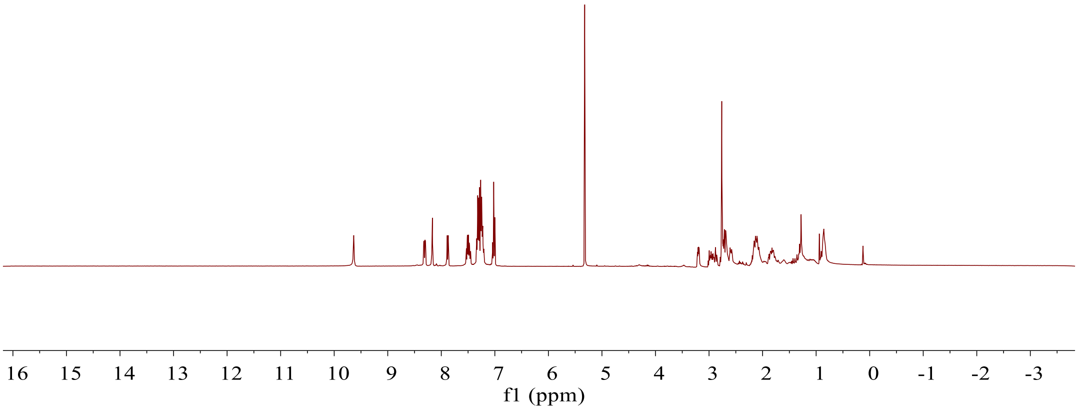

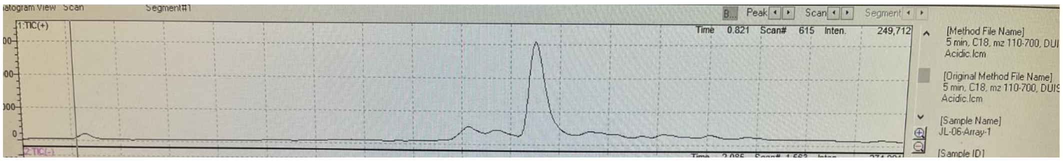

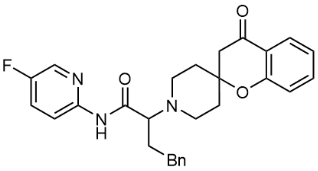

2
array 1

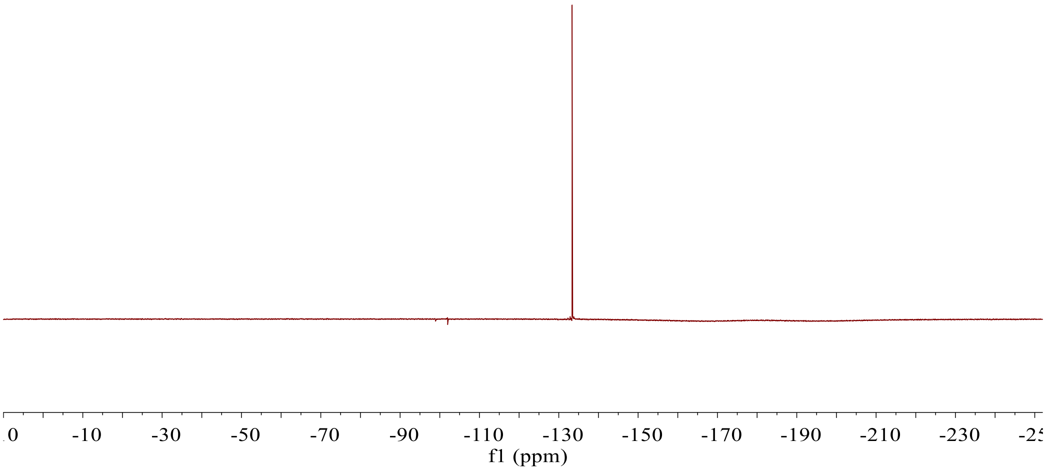

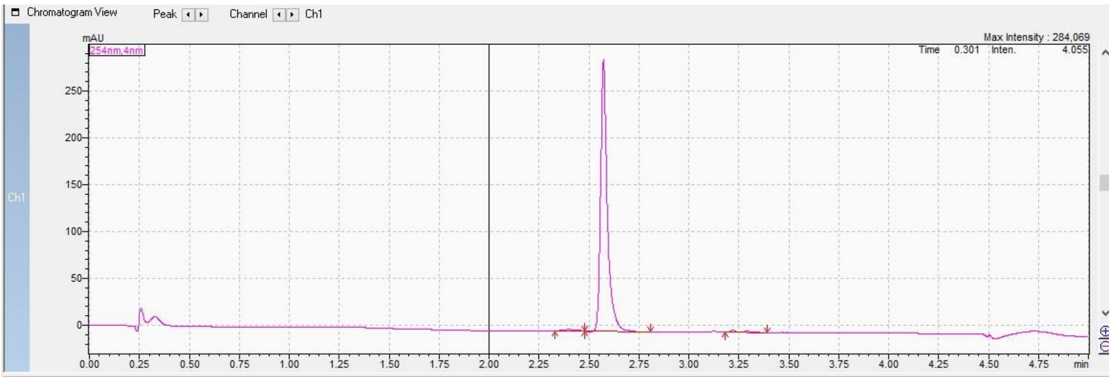

Results View - Peak Table

Peak Table | Compound | Group | Calibration Curve

Peak#	Ret. Time	Area	Height	Mark	Conc.	Unit	ID#	Name	Area%
1	2.399	9750	2491	M	0.000				1.399
2	2.573	679780	285777	M	0.000				97.520
3	3.220	7540	2434	M	0.000				1.082
Total		697069	290702		0.000				100.000

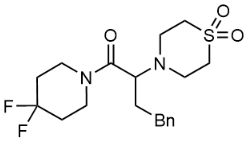

5
array 2

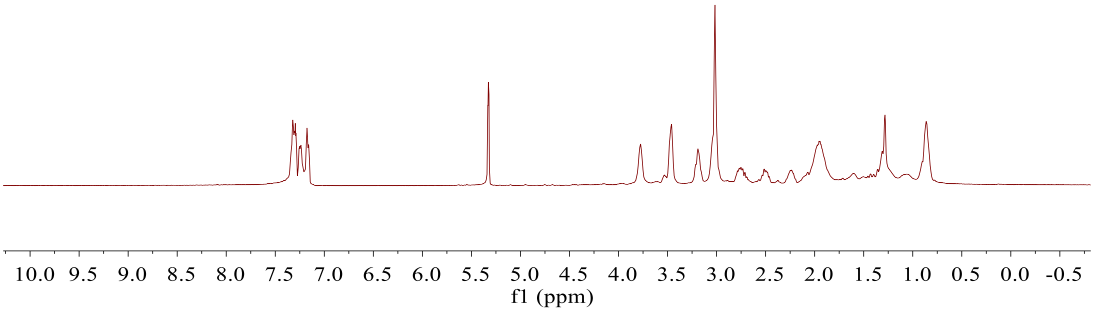

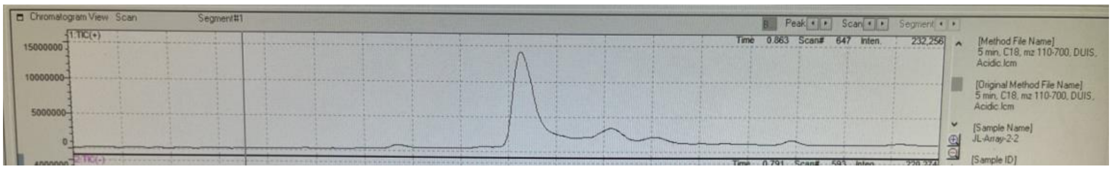

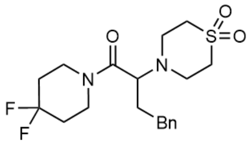

5
array 2

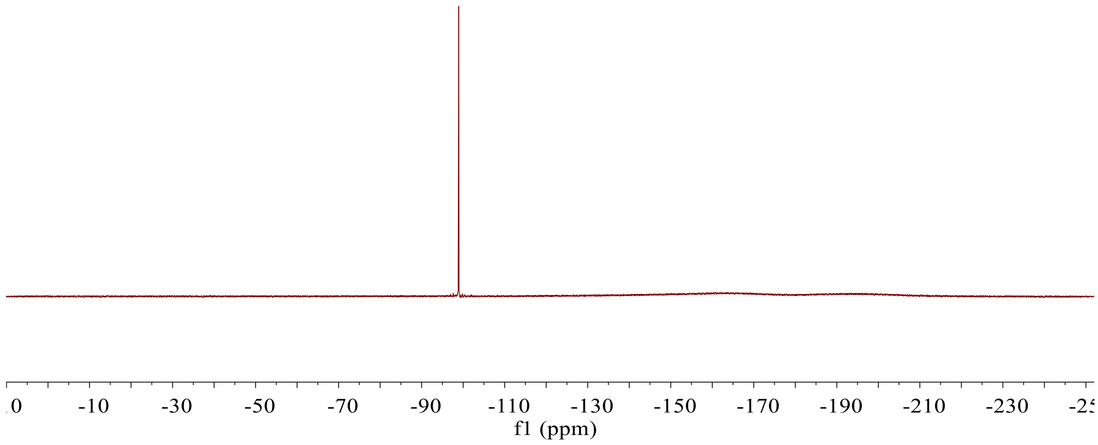

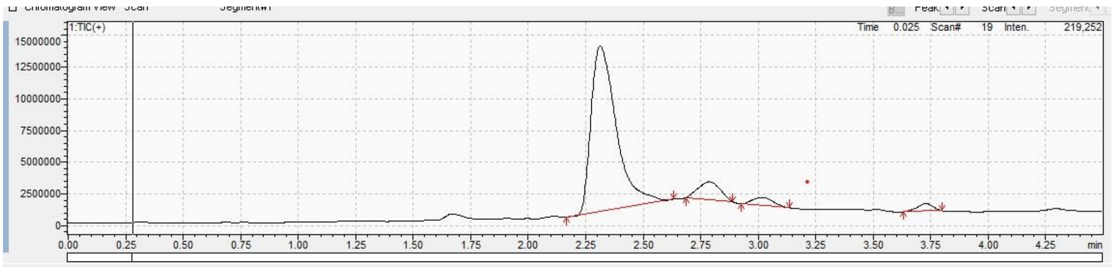

Results View - Peak Table

Spectrum | Peak Table | Compound | Group | Calibration Curve

Mass | Compound Name | PeakTable

TIC(Ev1)
TIC(Ev2)

Peak#	Ret. Time	Area	m/z	Area%
1	2.313	99257512	TIC	86.082
2	2.787	9214796	TIC	7.992
3	3.016	3971679	TIC	3.444
4	3.728	2861486	TIC	2.482
Total		115305473		100.000

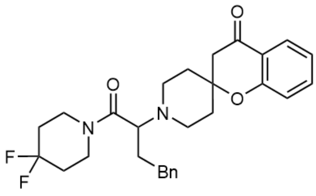

6
array 3

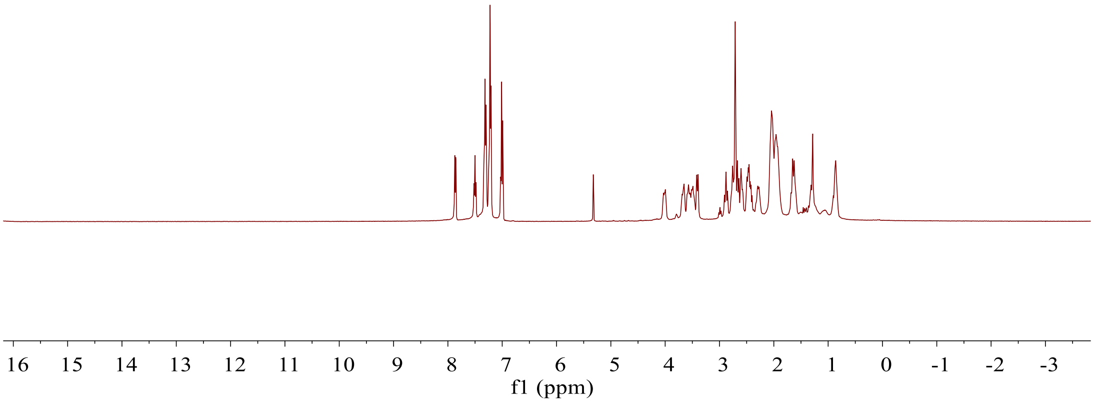

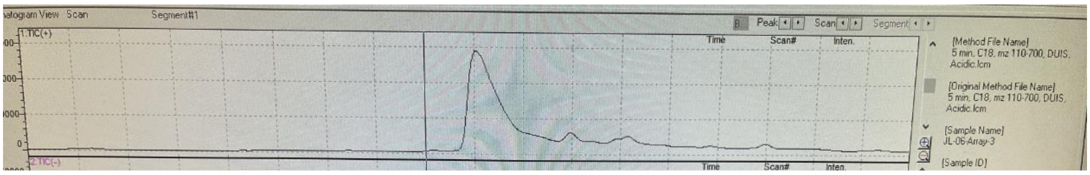

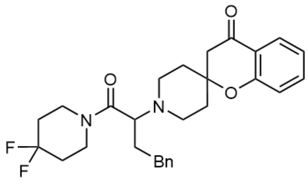

6
array 3

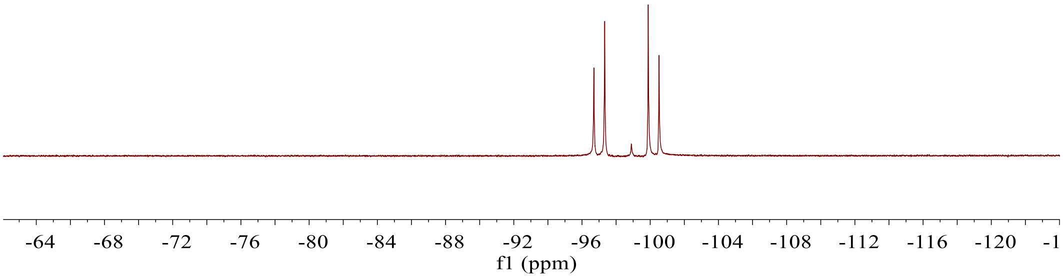

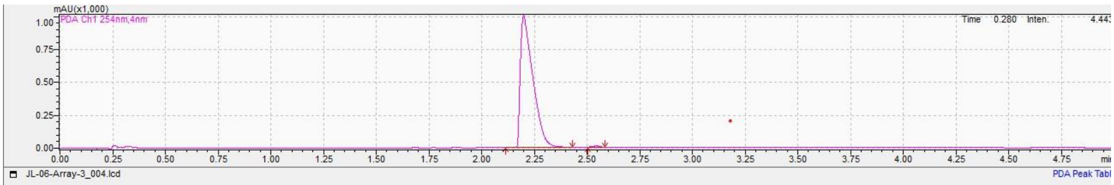

Peak#	Ret. Time	Area	Height	Mark	Conc.	Unit	ID#	Name	Area%
1	2.200	4180624	987931	M	0.000				99.367
2	2.546	26631	11096	M	0.000				0.633
Total		4207255	999027		0.000				100.000

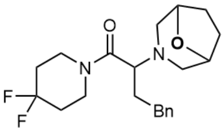

7
array 4

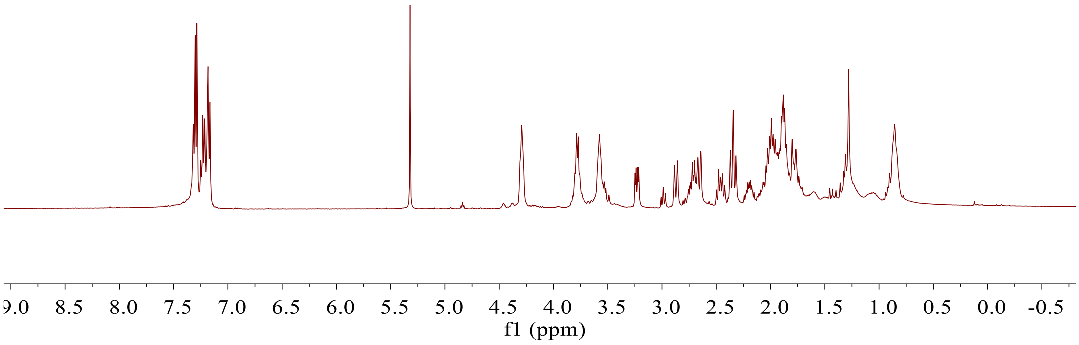

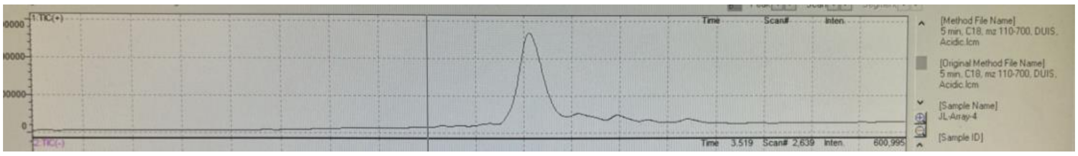

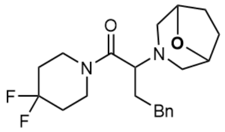

7
array 4

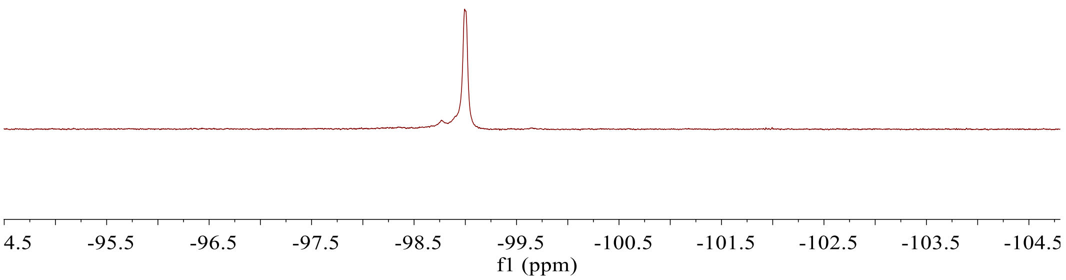

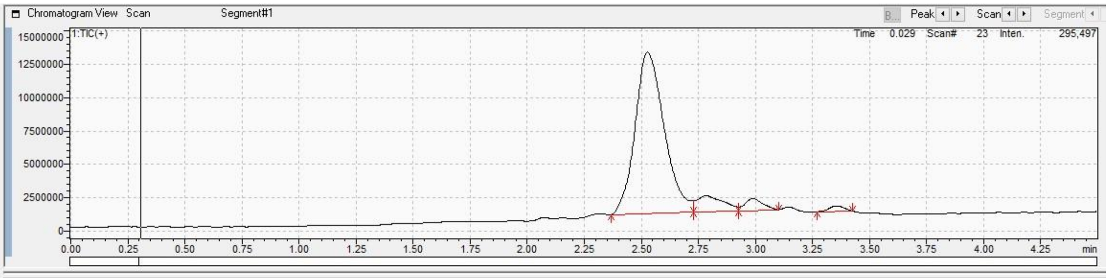

Results View - Peak Table

Spectrum | Peak Table | Compound | Group | Calibration Curve

Mass, Compound Name, PeakTable

TIC(Ev1)
TIC(Ev2)

Peak#	Ret. Time	Area	m/z	Area%
1	2.528	108032097	TIC	86.712
2	2.784	9918615	TIC	7.961
3	2.987	4889448	TIC	3.924
4	3.355	1747796	TIC	1.403
Total		124587956		100.000

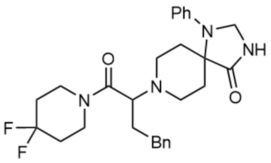

8
array 5

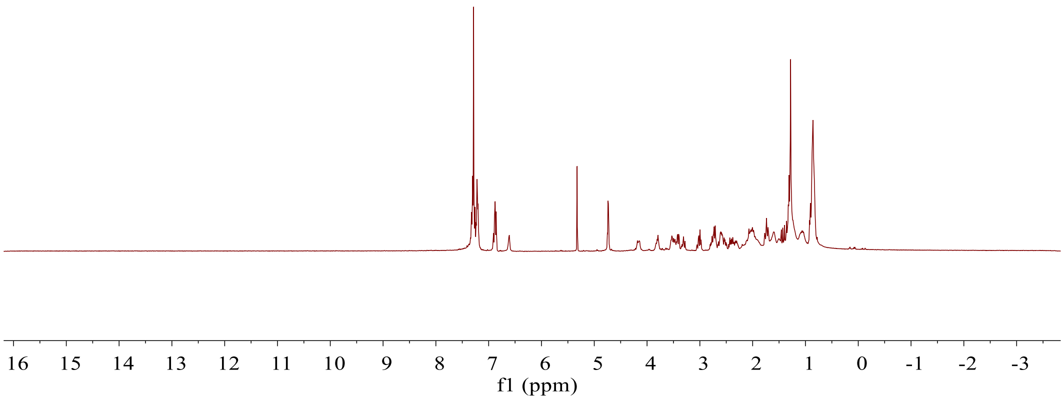

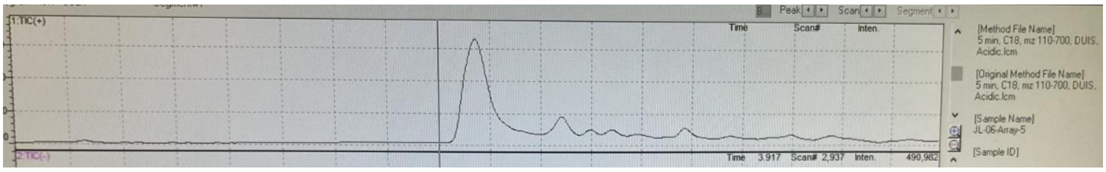

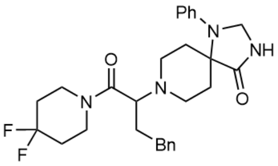

8
array 5

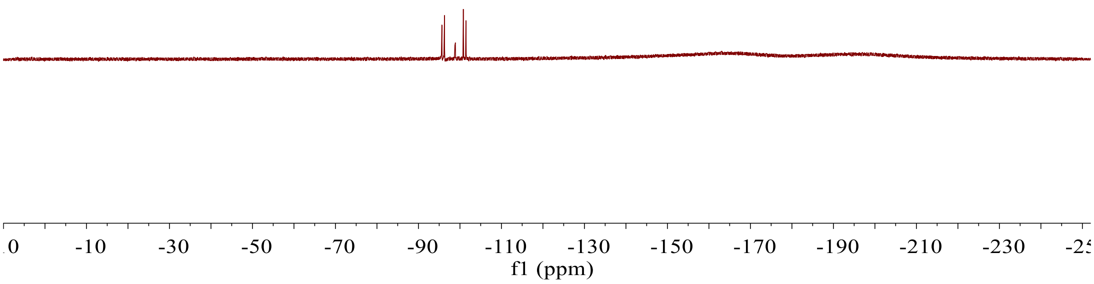

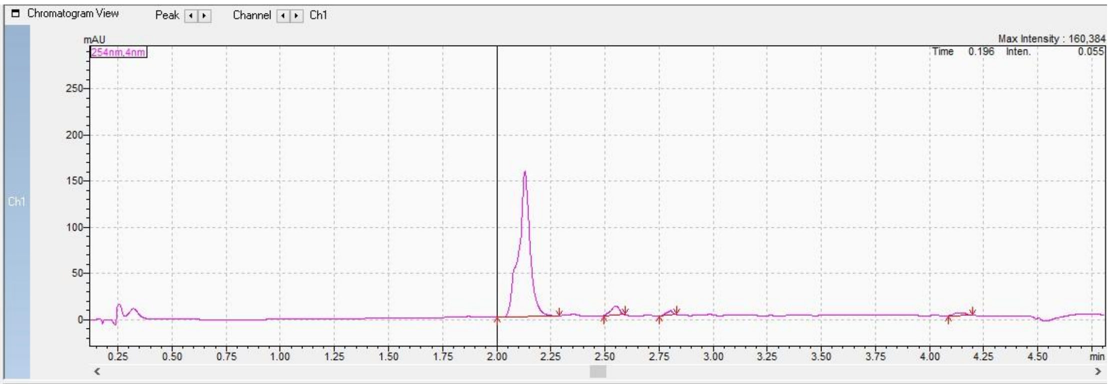

Results View - Peak Table

Peak Table | Compound | Group | Calibration Curve

Peak#	Ret. Time	Area	Height	Mark	Conc.	Unit	ID#	Name	Area%
1	2.131	573804	154231	M	0.000				92.550
2	2.550	25974	9525	M	0.000				4.189
3	2.803	9660	4514	M	0.000				1.558
4	4.150	10558	2948	M	0.000				1.703
Total		619996	171218		0.000				100.000

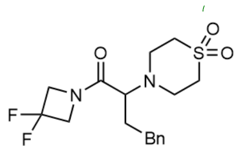

13
array 7

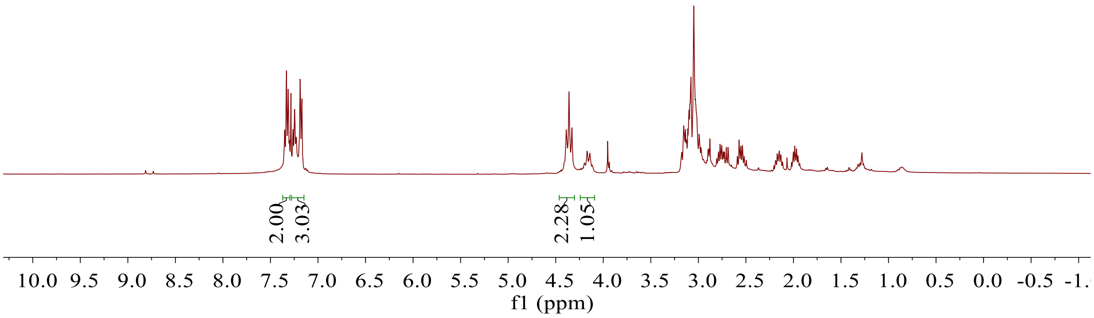

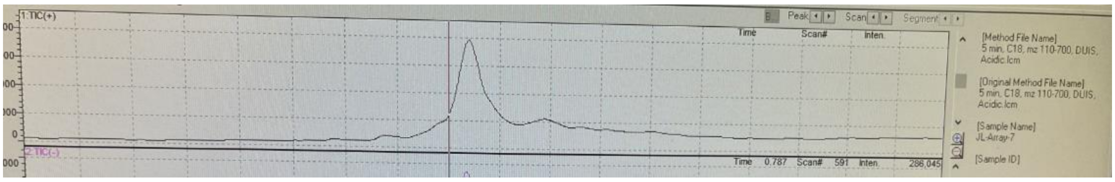

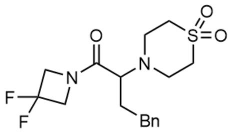

13
array 7

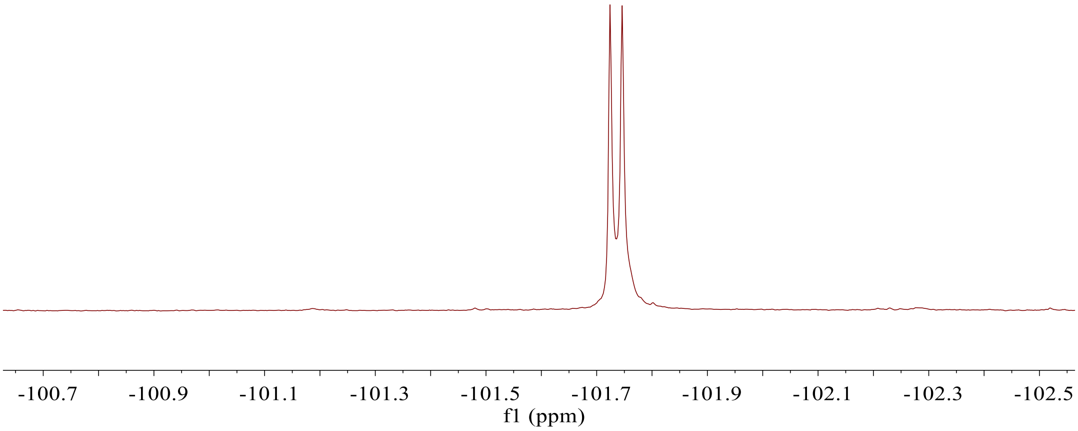

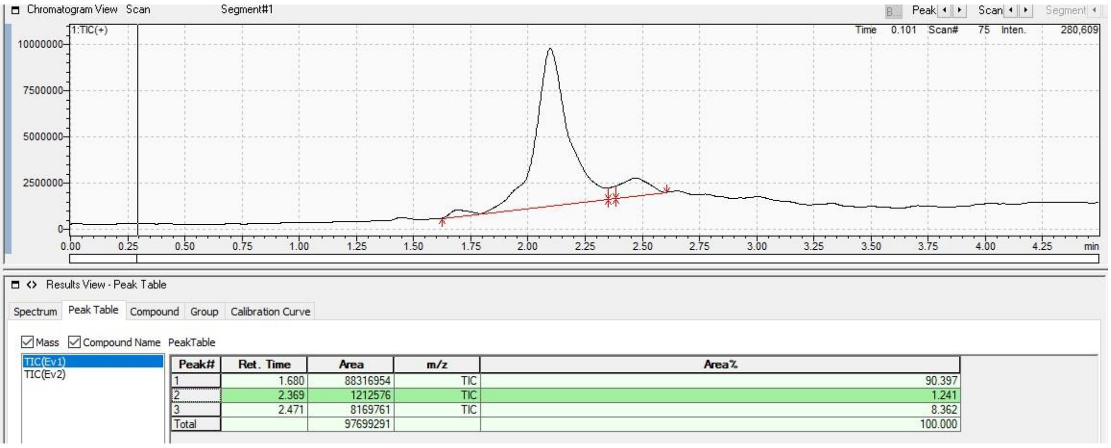

Peak#	Ret. Time	Area	m/z	Area%
1	1.680	88316954	TIC	90.397
2	2.369	1212576	TIC	1.241
3	2.471	8169761	TIC	8.362
Total		97699291		100.000

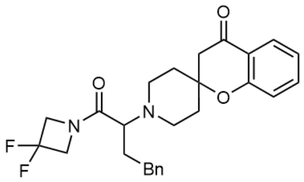

14
array 8

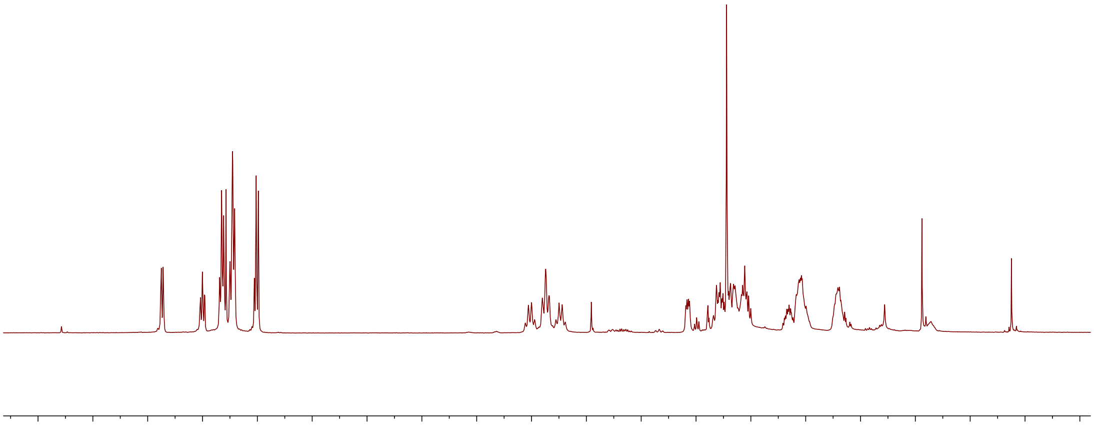

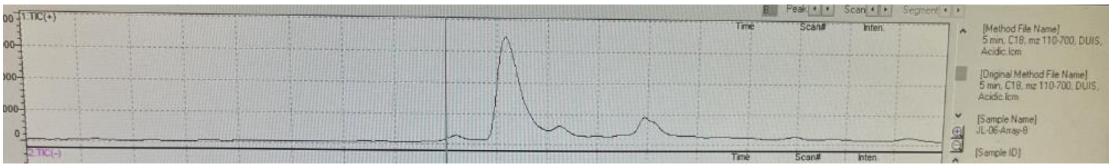

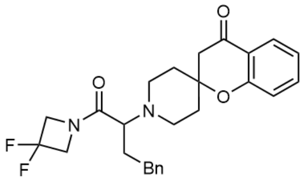

14
array 8

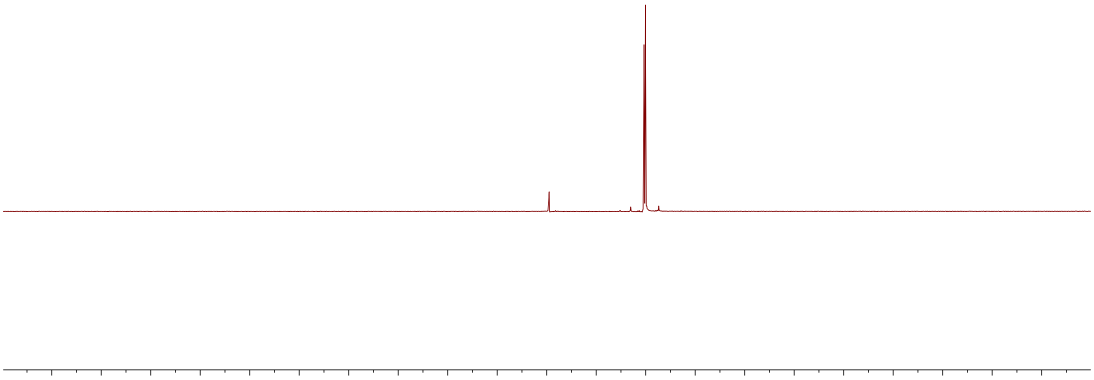

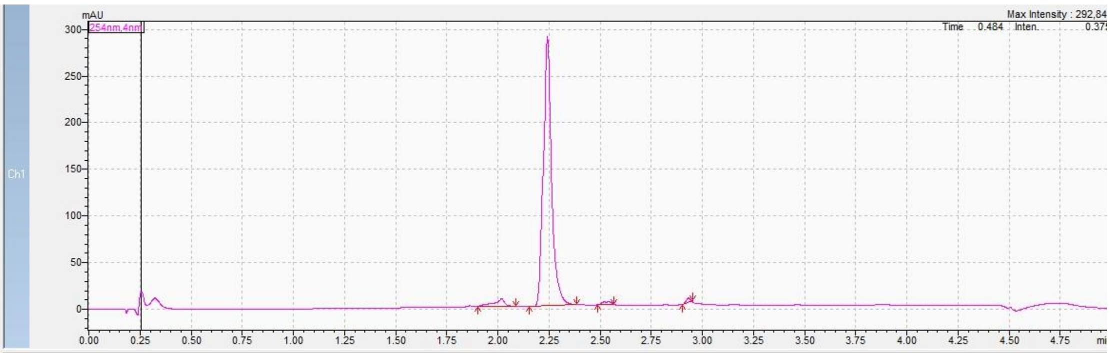

Results View - Peak Table

Peak Table | Compound | Group | Calibration Curve

Peak#	Ret. Time	Area	Height	Mark	Conc.	Unit	ID#	Name	Area%
1	2.019	28494	7822	M	0.000				3.348
2	2.243	807316	282600	M	0.000				94.867
3	2.541	8595	2746	M	0.000				1.010
4	2.933	6592	4438	M	0.000				0.775
Total		850997	297606		0.000				100.000

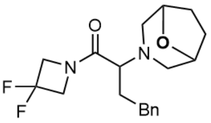

15
array 9

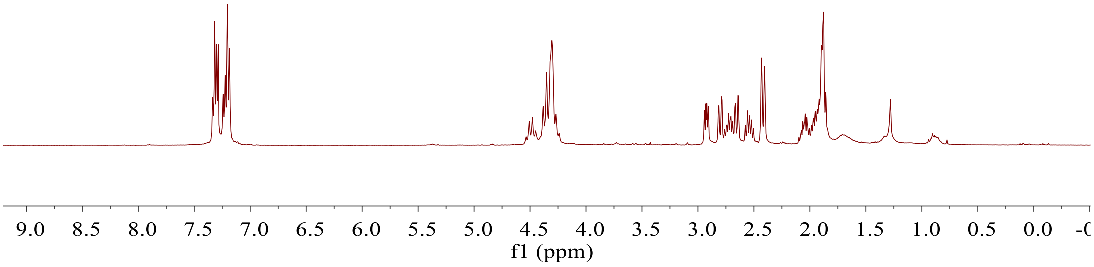

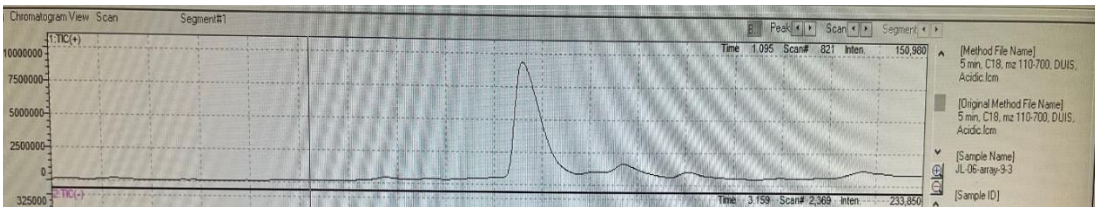

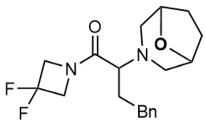

15
array 9

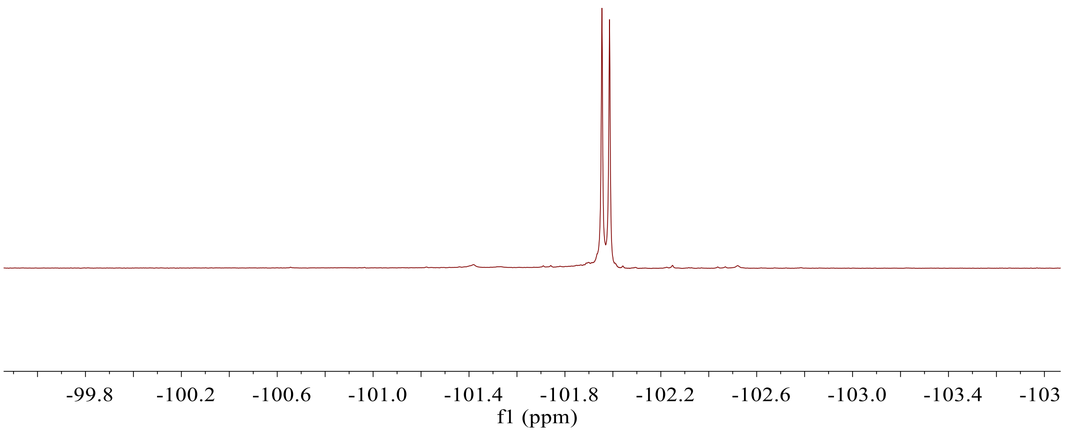

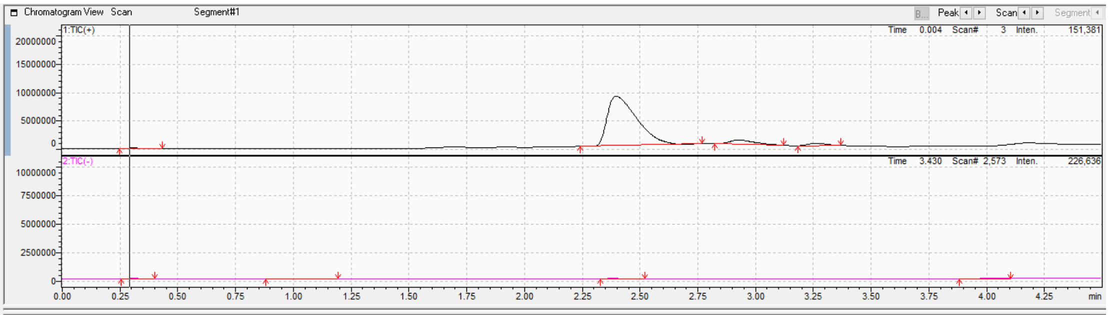

Results View - Peak Table

Spectrum | Peak Table | Compound | Group | Calibration Curve

Mass Compound Name PeakTable

TIC(Ev1)
TIC(Ev2)
1 Prod269

Peak#	Ret. Time	Area	m/z	Area%
1	0.309	518382	TIC	0.618
2	2.397	75842308	TIC	90.391
3	2.927	5364857	TIC	6.394
4	3.264	2179154	TIC	2.597
Total		83904702		100.000

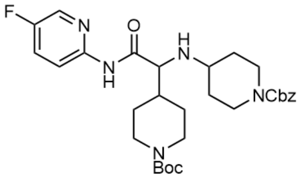

17
array 10

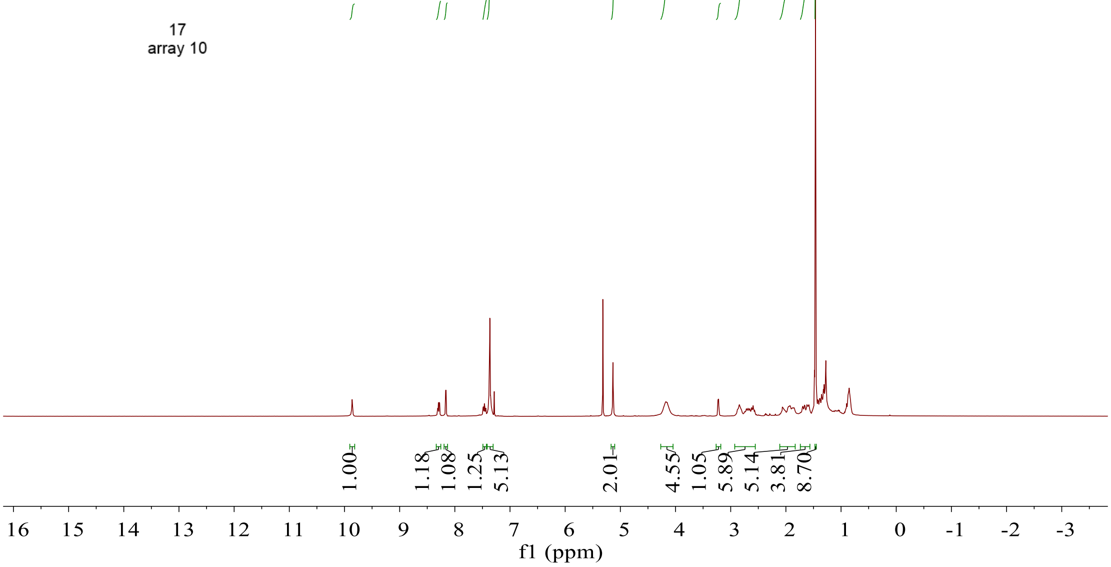

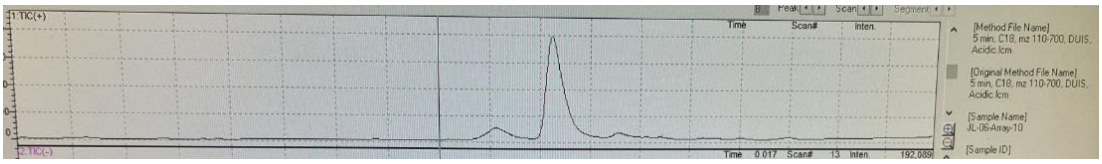

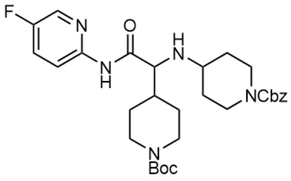

17
array 10

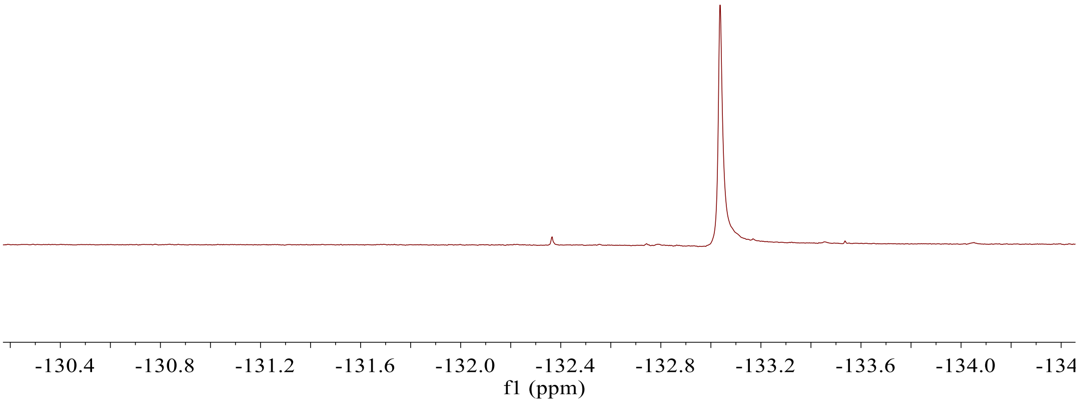

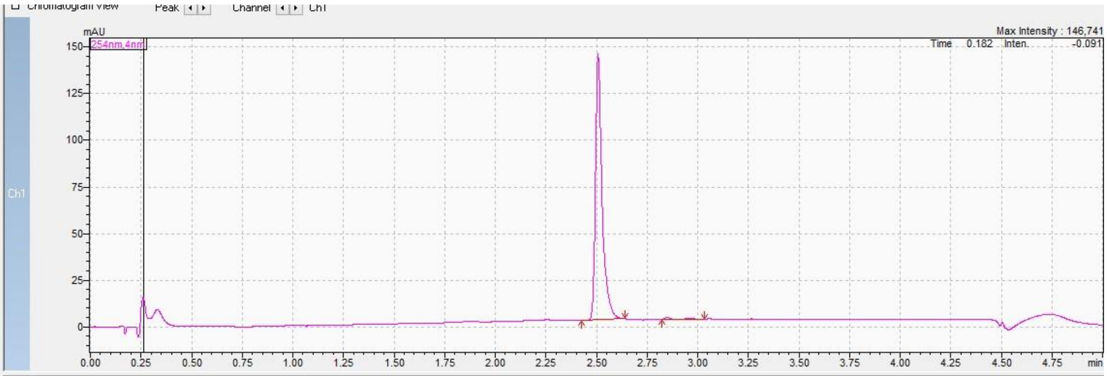

Results View - Peak Table

Peak Table | Compound | Group | Calibration Curve

Peak#	Ret. Time	Area	Height	Mark	Conc.	Unit	ID#	Name	Area%
1	2.509	326928	140484	M	0.000				98.658
2	2.848	4449	1115	M	0.000				1.342
Total		331377	141598		0.000				100.000

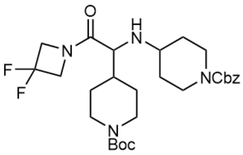

20
array 11

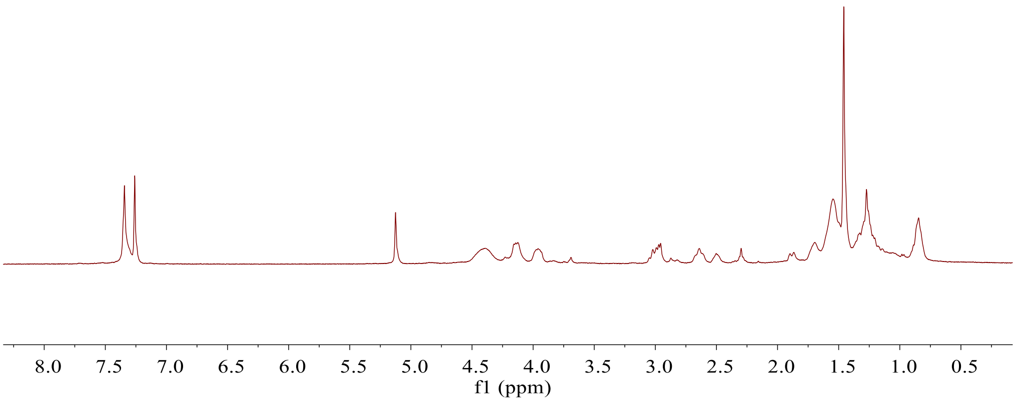

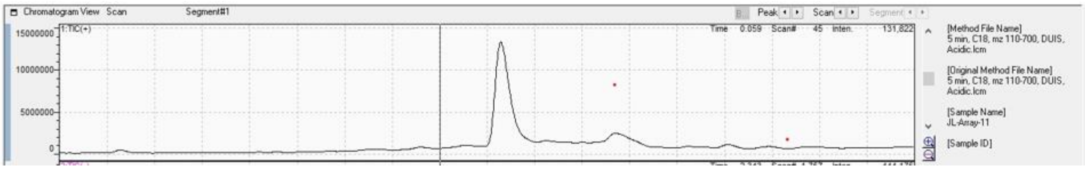

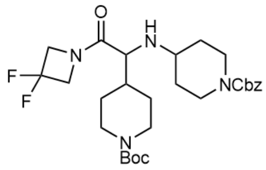

20
array 11

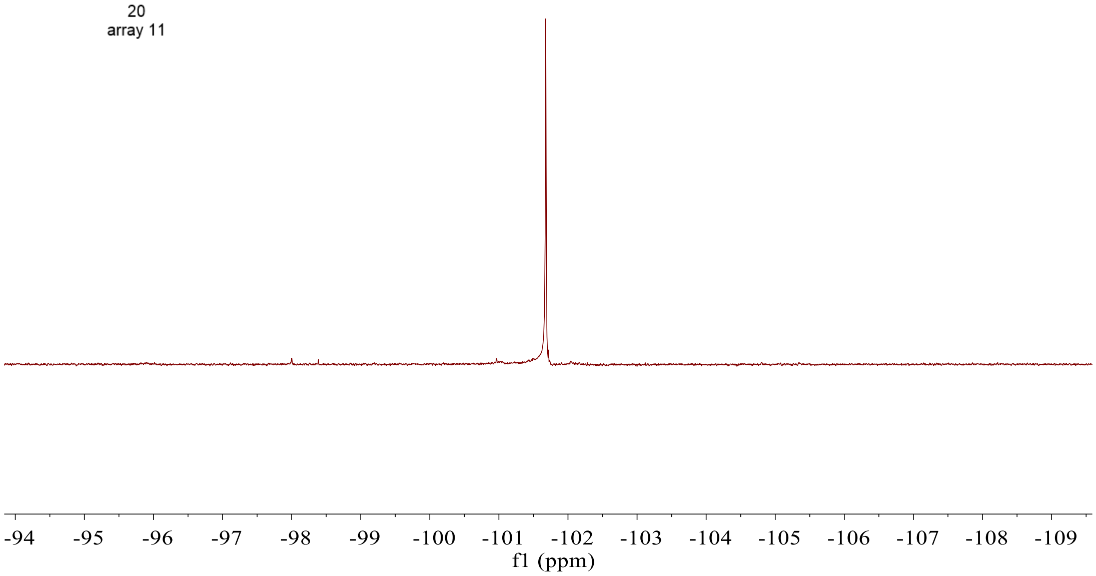

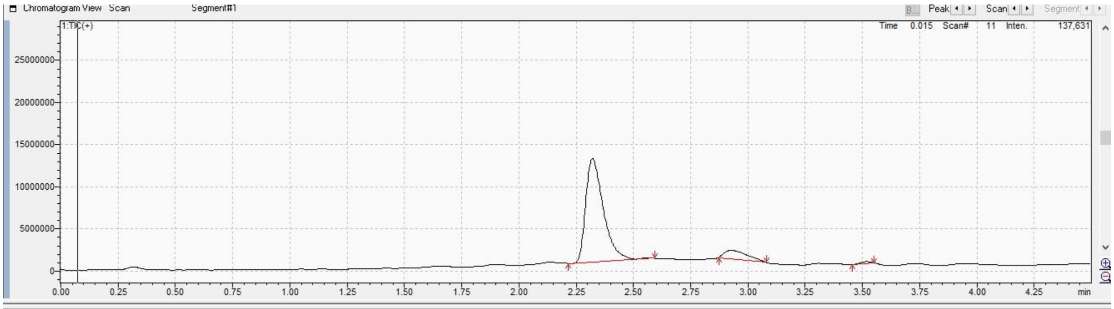

Results View - Peak Table

Spectrum | Peak Table | Compound | Group | Calibration Curve

Mass, Compound Name, PeakTable

TIC(Ev1)
TIC(Ev2)

Peak#	Ret. Time	Area	m/z	Area%
1	2.321	66001111	TIC	89.219
2	2.927	7212308	TIC	9.749
3	3.519	763020	TIC	1.031
Total		73976439		100.000

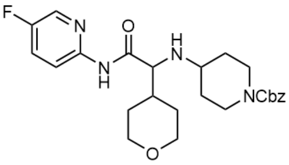

21
array 13

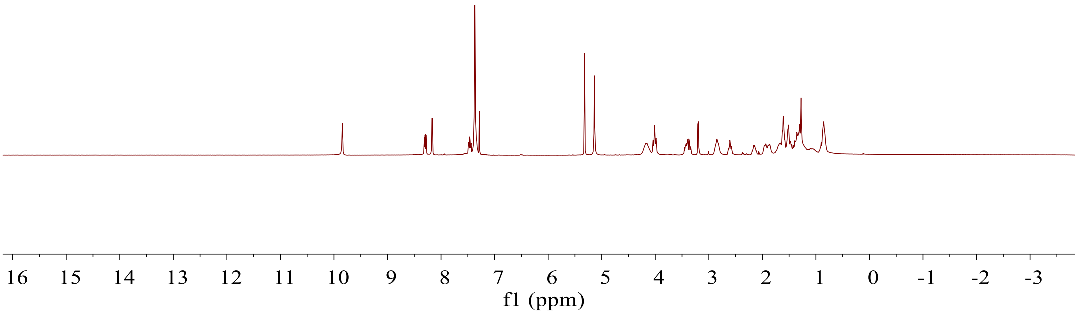

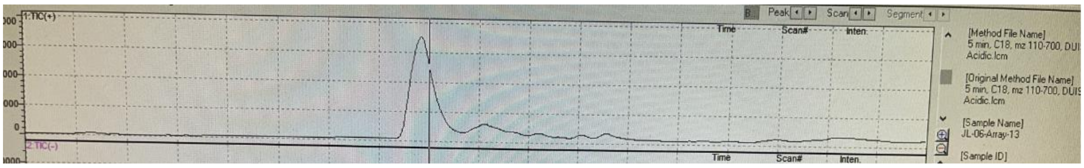

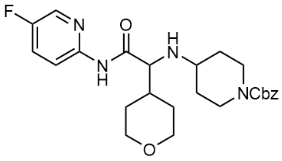

21
array 13

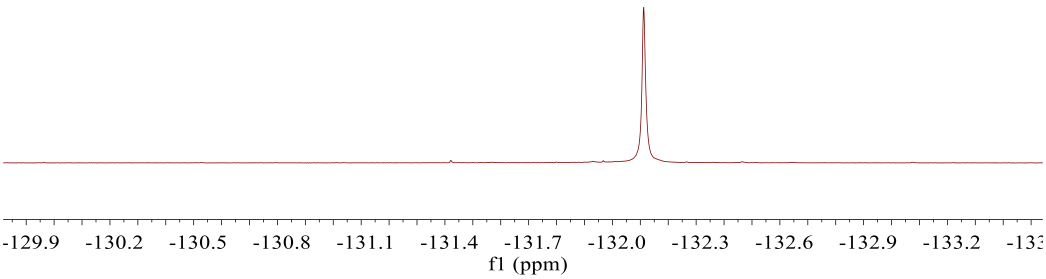

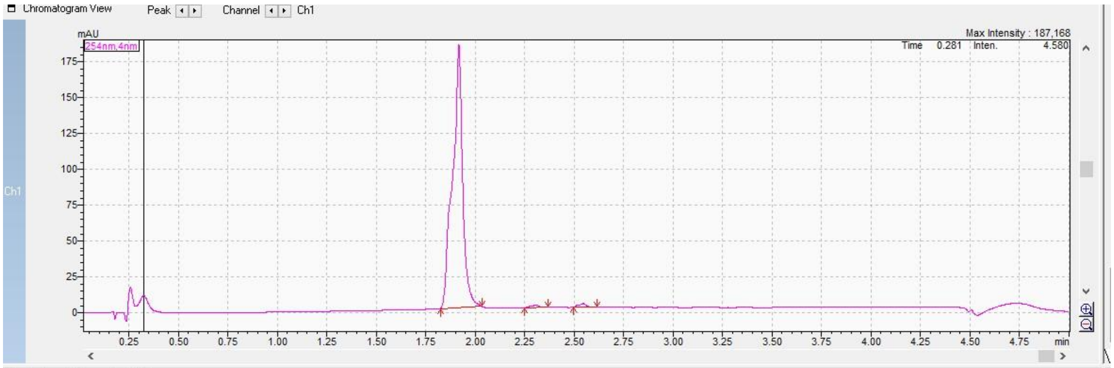

Results View - Peak Table

Peak Table | Compound | Group | Calibration Curve

Peak#	Ret. Time	Area	Height	Mark	Conc.	Unit	ID#	Name	Area%
1	1.916	648293	180922	M	0.000				98.233
2	2.307	5557	1879	M	0.000				0.842
3	2.545	6106	2235	M	0.000				0.925
Total		659955	185036		0.000				100.000

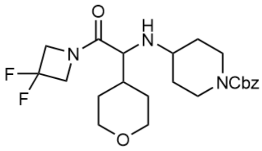

24
array 14

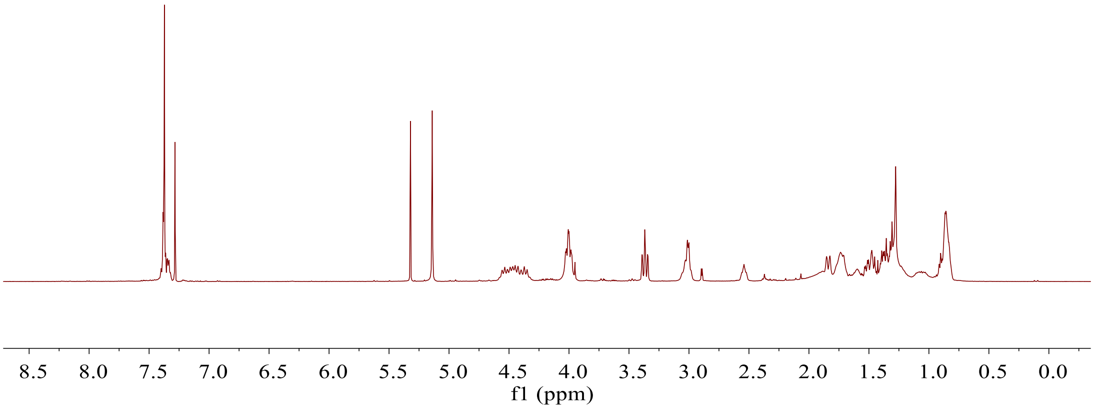

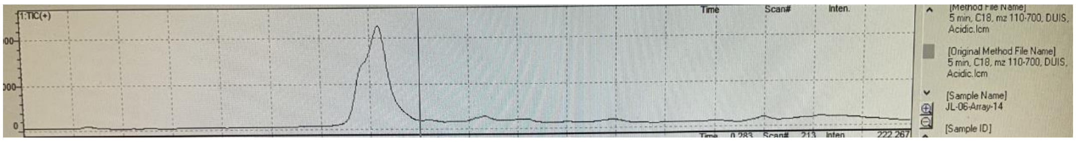

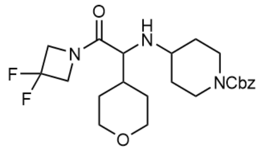

24
array 14

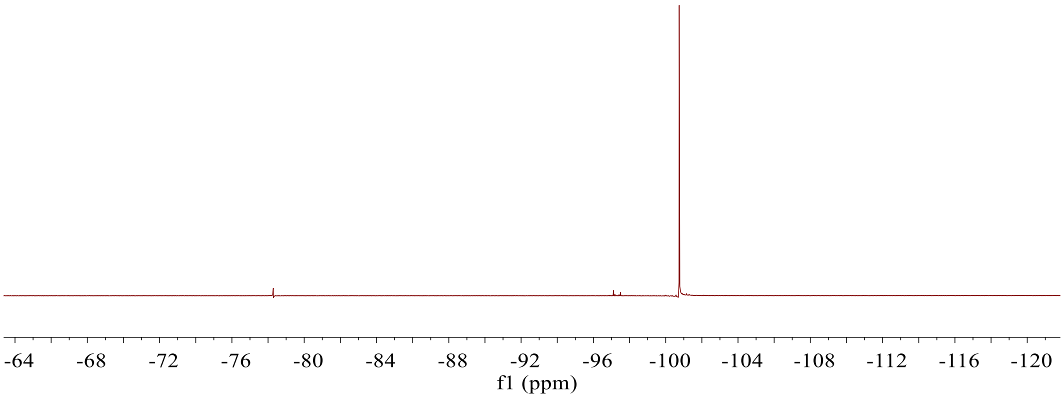

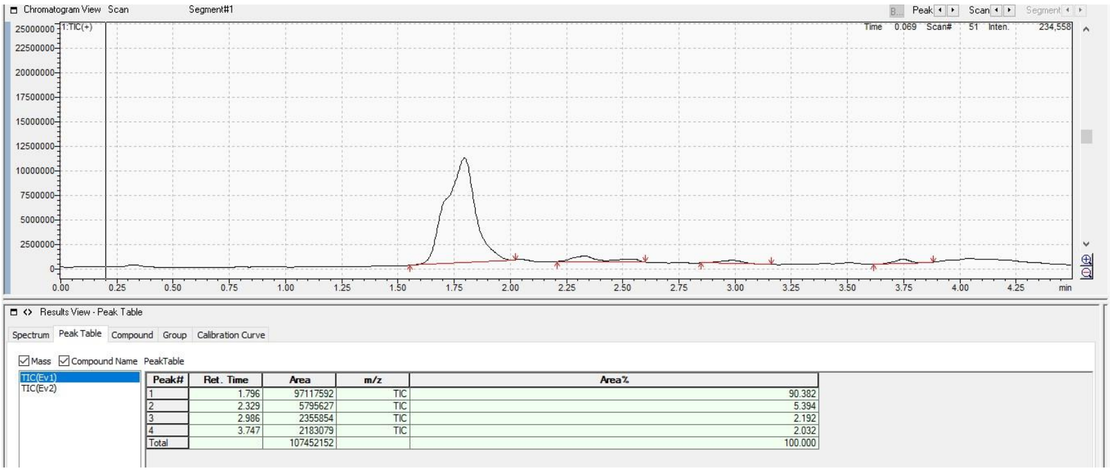

Peak#	Ret. Time	Area	m/z	Area%
1	1.796	97117592	TIC	90.382
2	2.329	5795627	TIC	5.394
3	2.986	2355854	TIC	2.192
4	3.747	2183079	TIC	2.032
Total		107452152		100.000

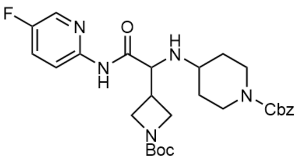

25
array 15

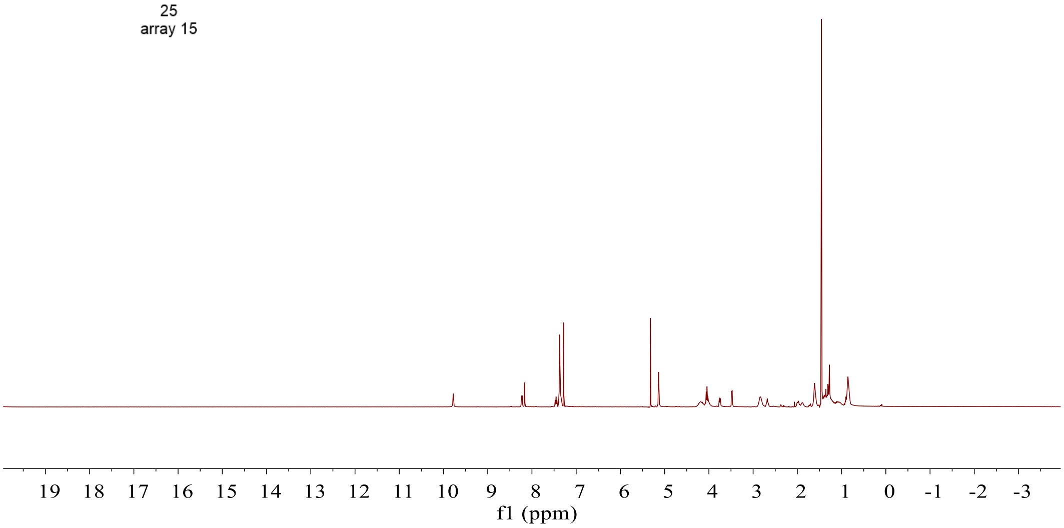

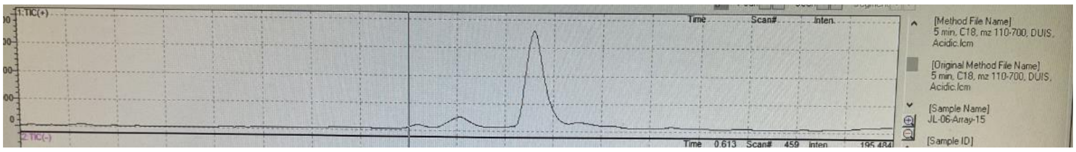

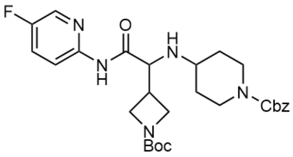

25
array 15

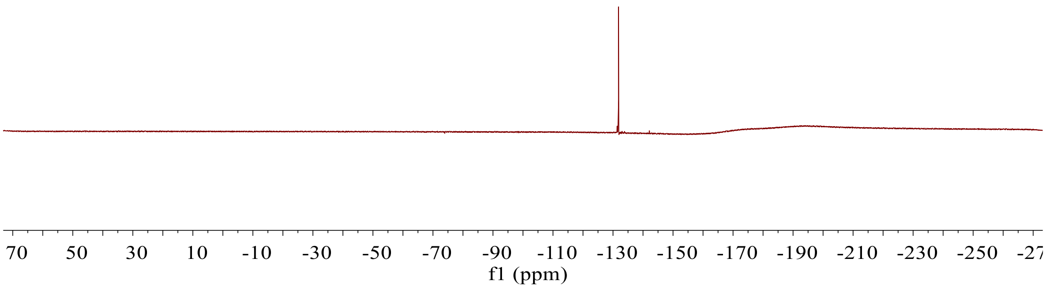

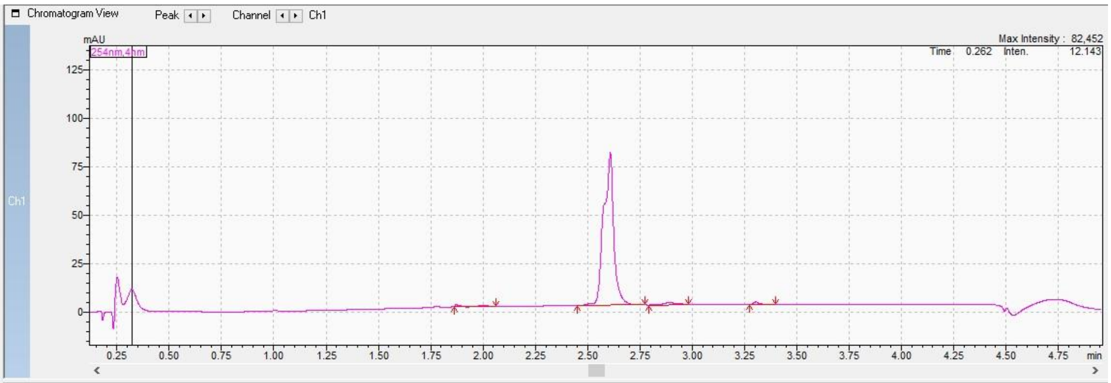

Results View - Peak Table

Peak Table | Compound | Group | Calibration Curve

Peak#	Ret. Time	Area	Height	Mark	Conc.	Unit	ID#	Name	Area%
1	1.875	2842	831	M	0.000				1.037
2	2.608	263362	74927	M	0.000				96.142
3	2.898	4442	1072	M	0.000				1.621
4	3.304	3284	1370	M	0.000				1.199
Total		273929	78200		0.000				100.000

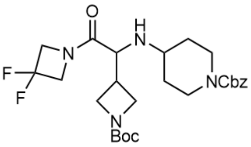

28
array 16

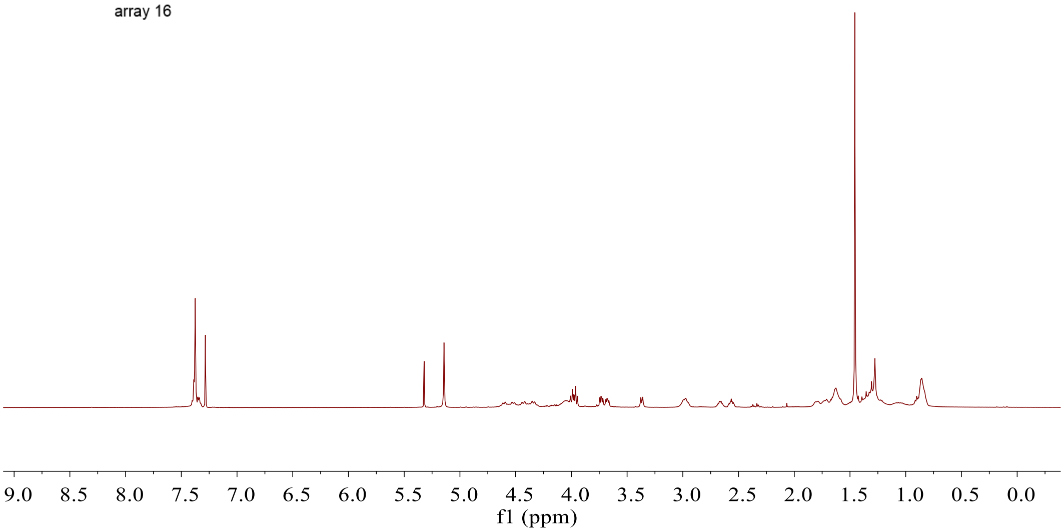

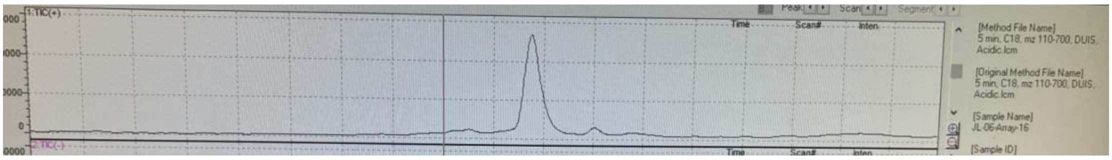

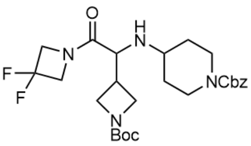

28
array 16

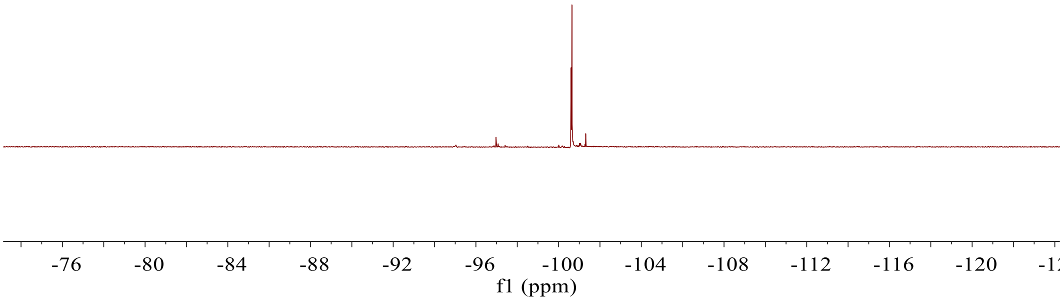

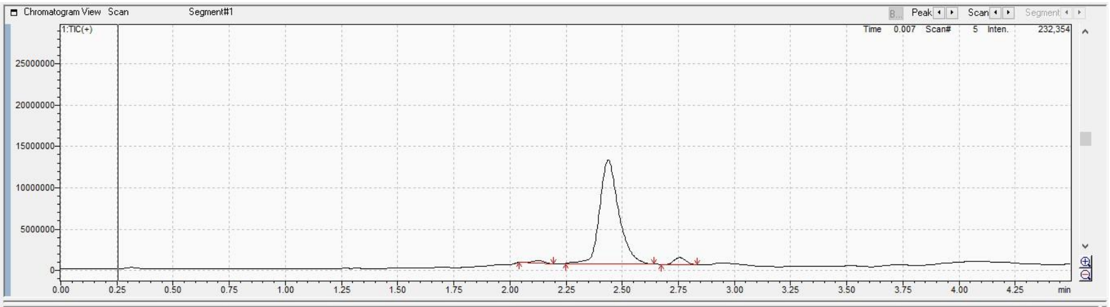

Results View - Peak Table

Spectrum | Peak Table | Compound | Group | Calibration Curve

Mass | Compound Name | PeakTable

TIC(Ev1)
TIC(Ev2)

Peak#	Ret. Time	Area	m/z	Area%
1	2.125	1218879	TIC	1.559
2	2.438	74000819	TIC	94.634
3	2.755	2977171	TIC	3.807
Total		78196869		100.000

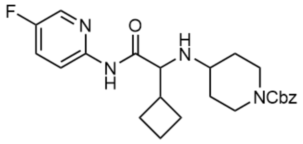

29
array 17

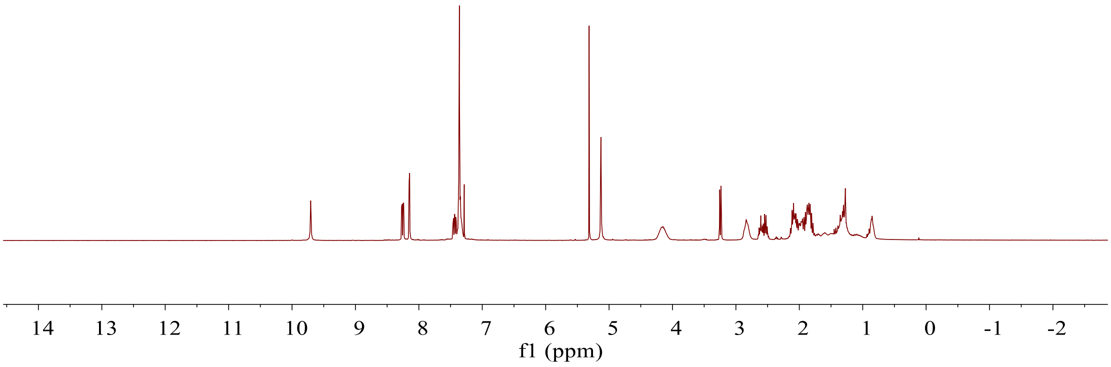

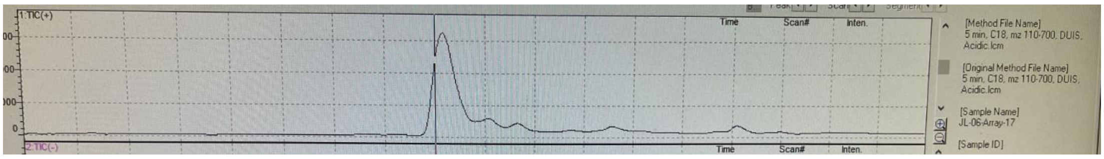

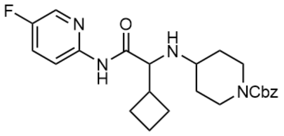

29
array 17

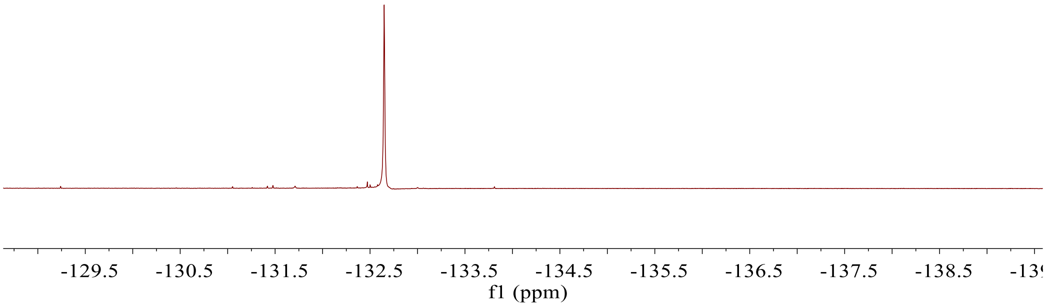

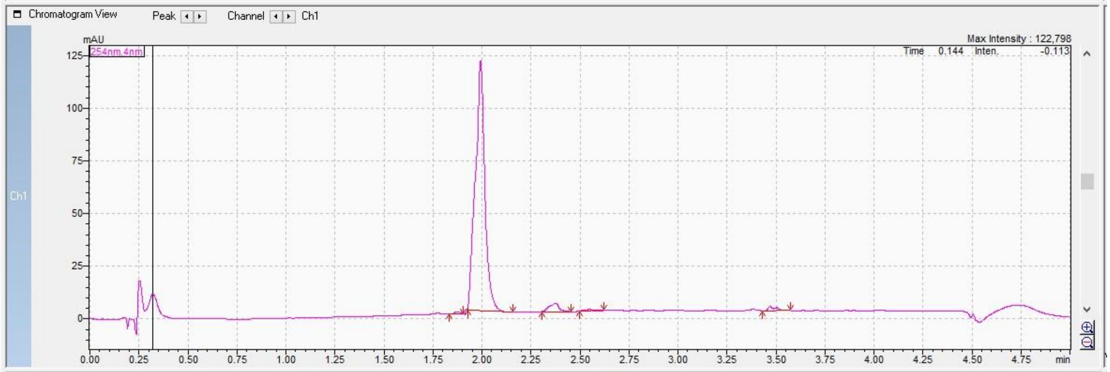

Results View - Peak Table

Peak Table | Compound | Group | Calibration Curve

Peak#	Ret. Time	Area	Height	Mark	Conc.	Unit	ID#	Name	Area%
1	1.871	1428	930	M	0.000				0.340
2	1.994	397995	115105	M	0.000				94.681
3	2.374	13781	4014	M	0.000				3.279
4	2.547	1761	788	M	0.000				0.419
5	3.471	5387	1884	M	0.000				1.282
Total		420352	122721		0.000				100.000

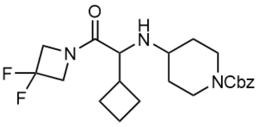

32
array 18

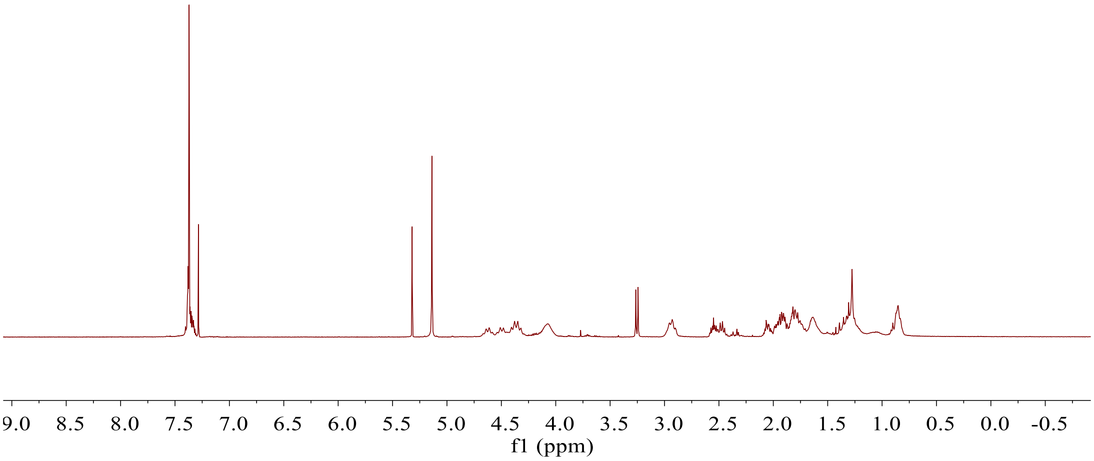

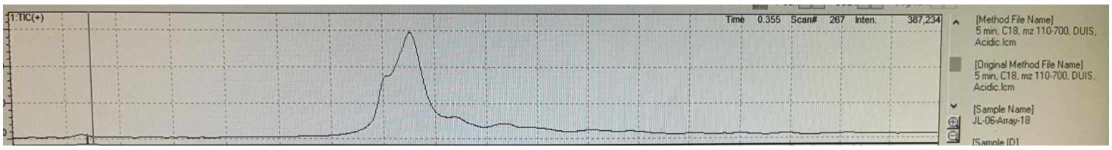

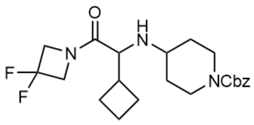
O
H
N
N
F
F
NCbz

32
array 18

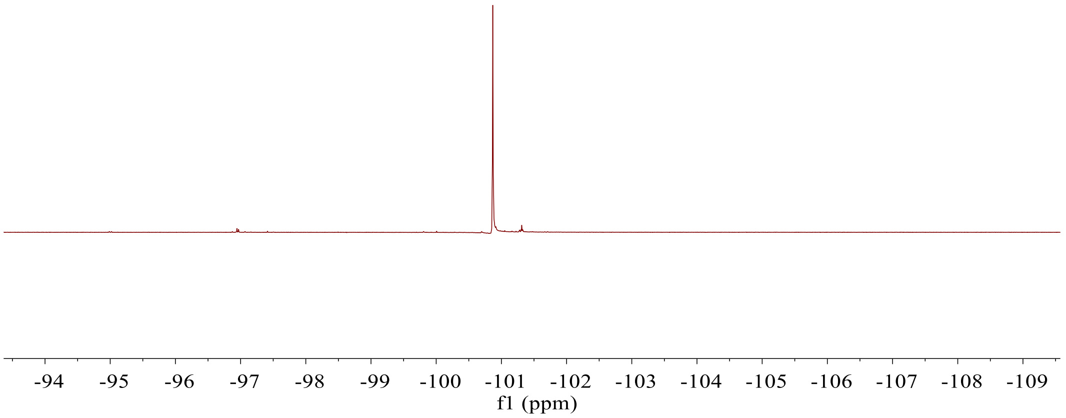
-94 -95 -96 -97 -98 -99 -100 -101 -102 -103 -104 -105 -106 -107 -108 -109
f1 (ppm)

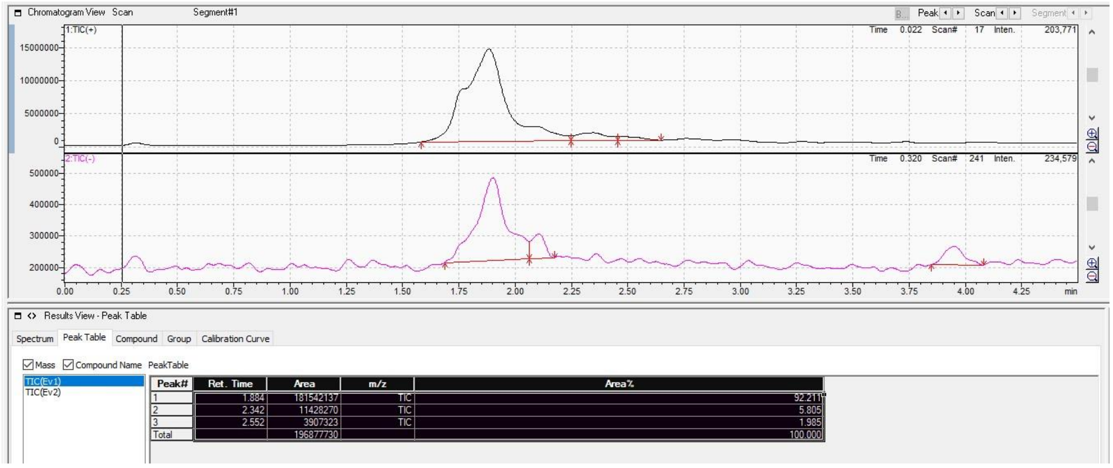
Chromatogram View Scan
Segment#1
1:TIC(+)
2:TIC(-)
Results View - Peak Table
Spectrum
Peak Table
Compound
Group
Calibration Curve
TIC(Ev1)
TIC(Ev2)
Peak# | Ret. Time | Area | m/z | Area%
1 | 1.884 | 181542137 | TIC | 92.211
2 | 2.342 | 11428270 | TIC | 5.805
3 | 2.552 | 3907323 | TIC | 1.985
Total | | 196877730 | | 100.000

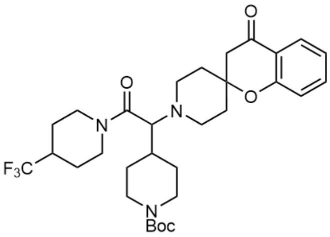

34
array 19

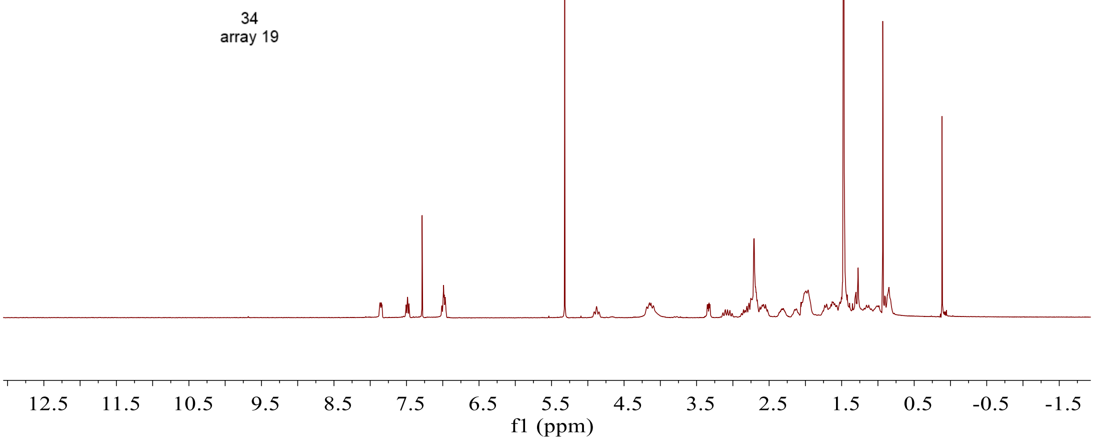

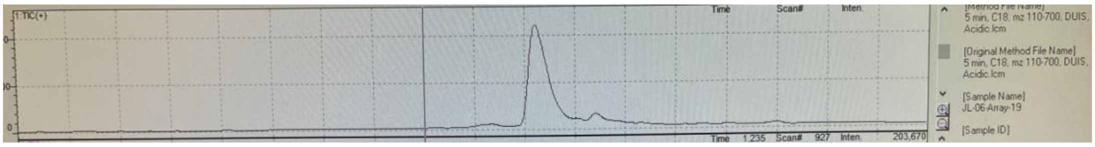

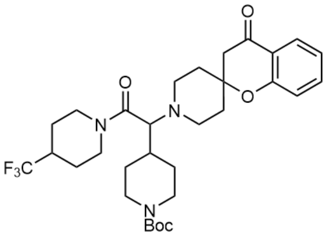

34
array 19

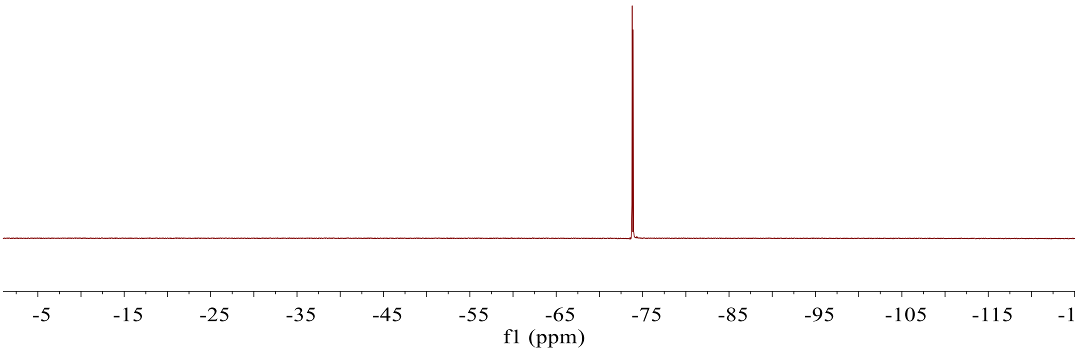

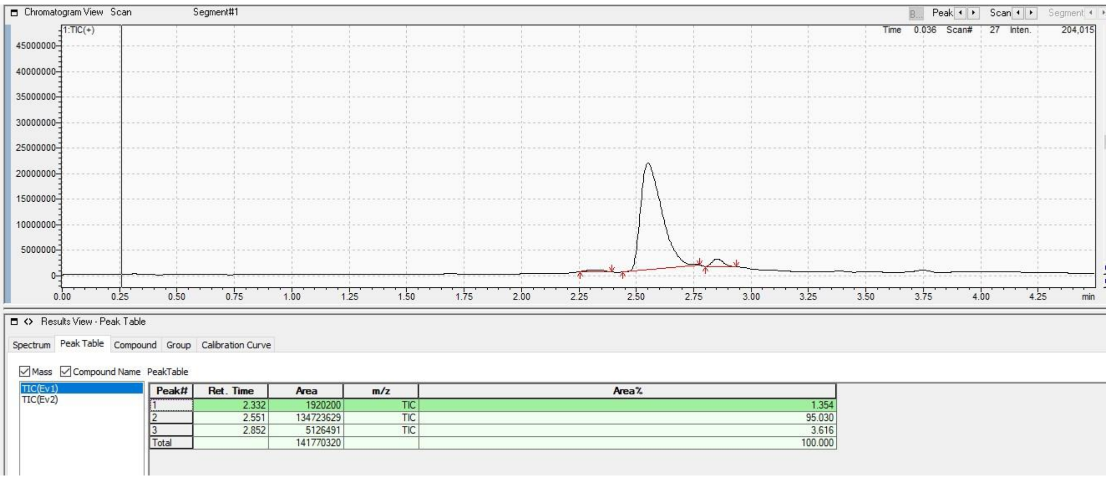

Peak#	Ret. Time	Area	m/z	Area%
1	2.332	1920200	TIC	1.354
2	2.551	134723629	TIC	95.030
3	2.852	5126491	TIC	3.616
Total		141770320		100.000

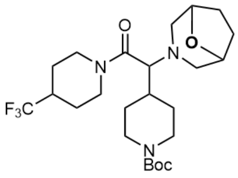

35
array 20

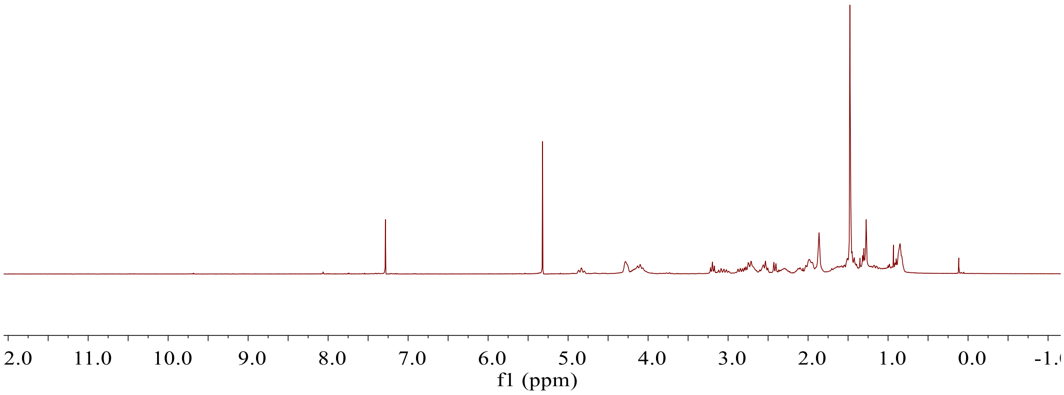

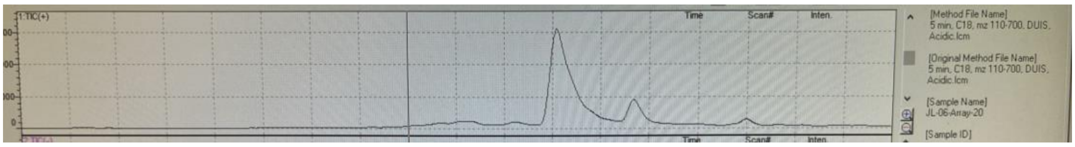

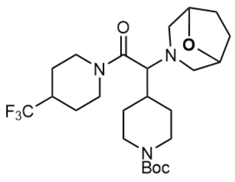

35
array 20

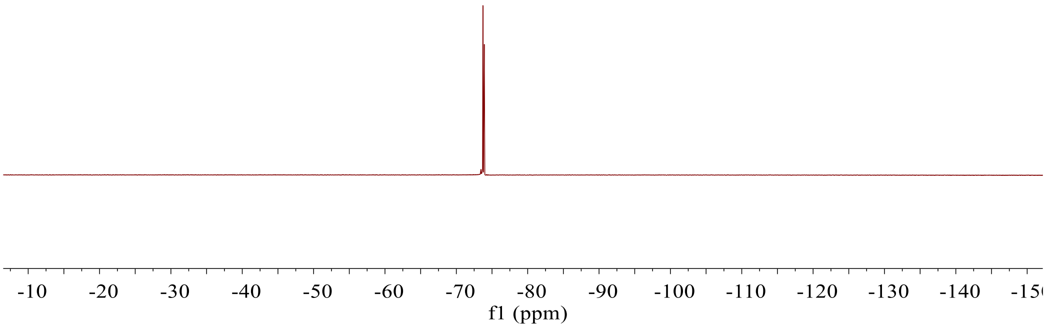

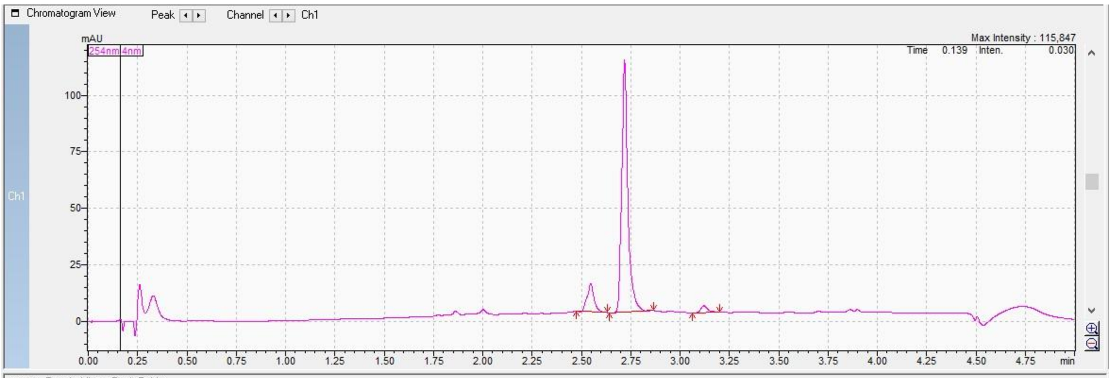

Results View - Peak Table

Peak Table | Compound | Group | Calibration Curve

Peak#	Ret. Time	Area	Height	Mark	Conc.	Unit	ID#	Name	Area%
1	2.548	29819	12327	M	0.000				10.823
2	2.719	238698	106739	M	0.000				86.632
3	3.121	7013	2819	M	0.000				2.545
Total		275530	121886		0.000				100.000

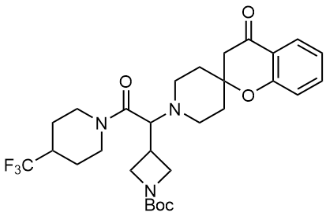

42
array 21

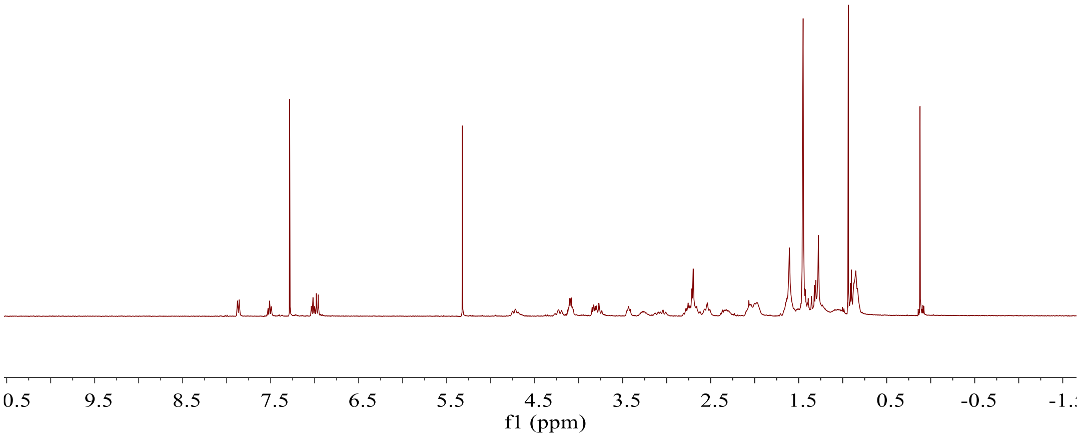

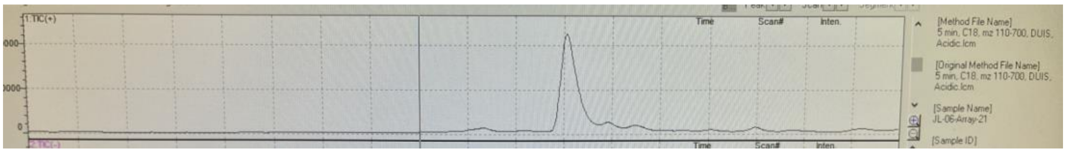

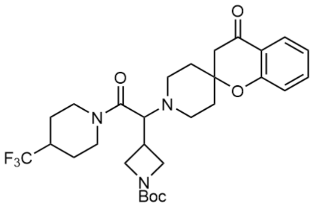

42
array 21

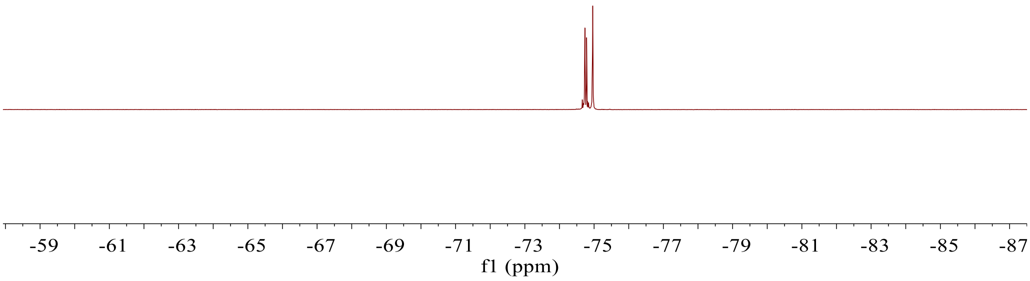

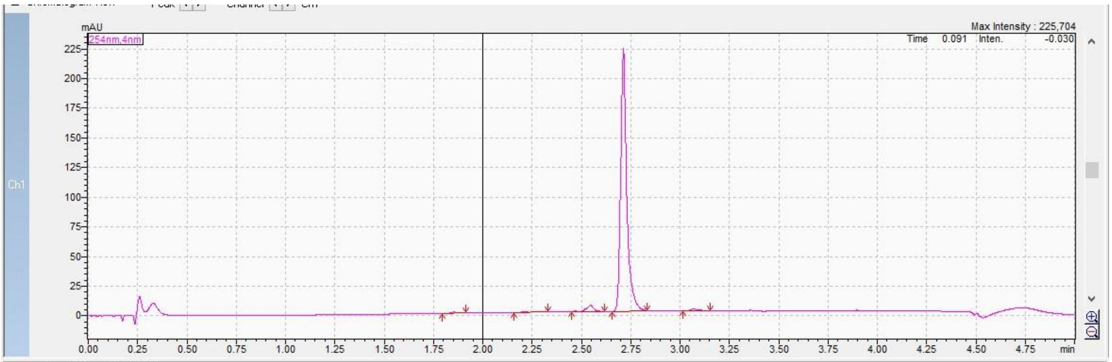

Results View - Peak Table

Peak Table | Compound | Group | Calibration Curve

Peak#	Ret. Time	Area	Height	Mark	Conc.	Unit	ID#	Name	Area%
1	1.855	1171	762	M	0.000				0.236
2	2.298	706	430	M	0.000				0.143
3	2.547	12878	5098	M	0.000				2.601
4	2.713	475288	207780	M	0.000				95.979
5	3.070	5159	1573	M	0.000				1.042
Total		495201	215643		0.000				100.000

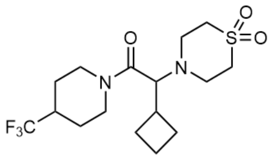

45
array 22

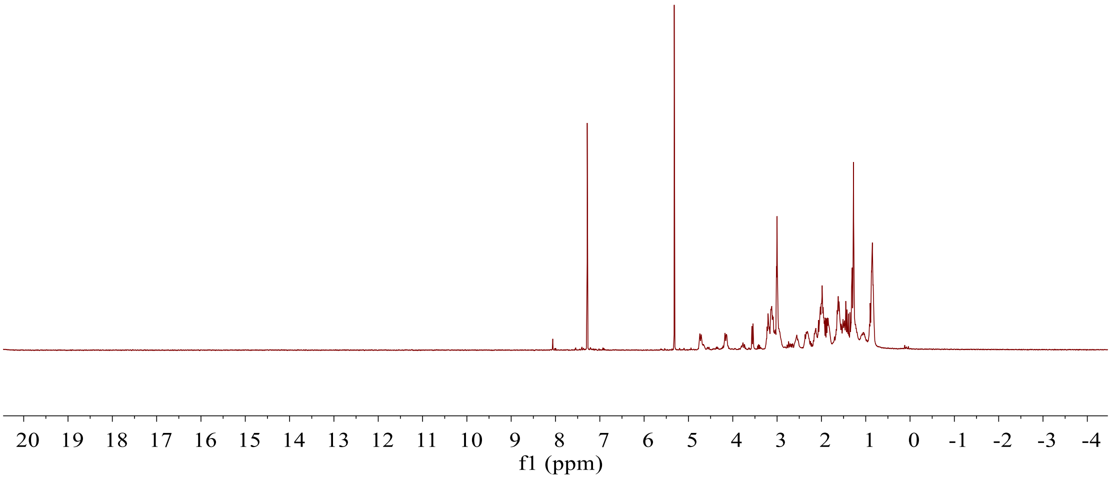

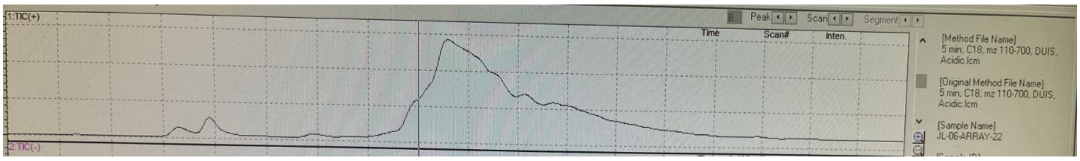

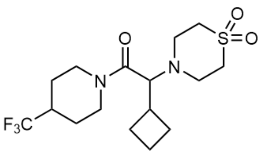

45
array 22

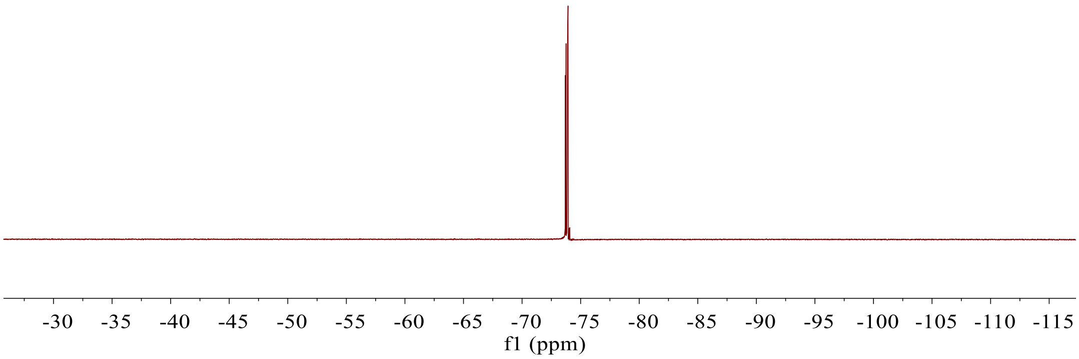

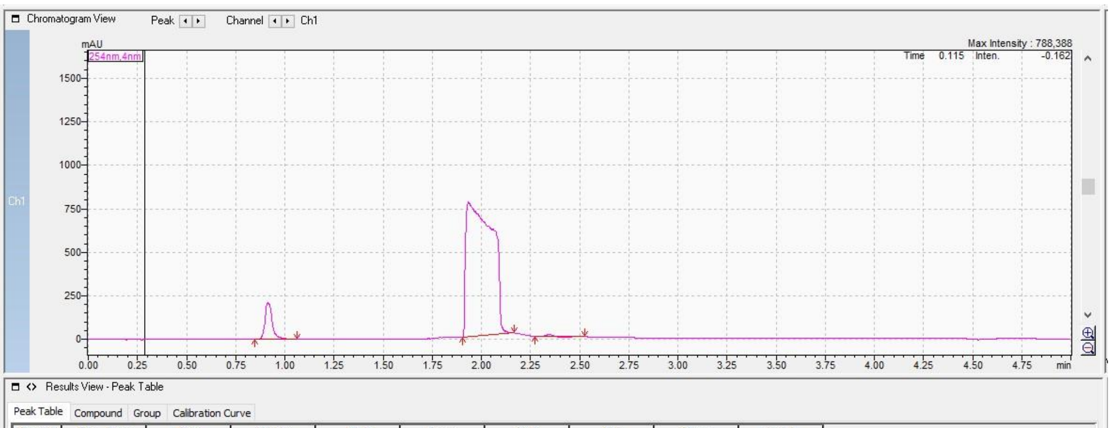

Peak#	Ret. Time	Area	Height	Mark	Conc.	Unit	ID#	Name	Area%
1	0.915	532174	206889	M	0.000				6.960
2	1.936	7111683	768193	M	0.000				93.004
3	2.345	2793	10958	M	0.000				0.037
Total		7646650	986040		0.000				100.000

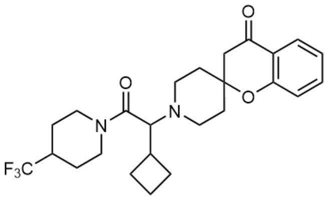

46
array 23

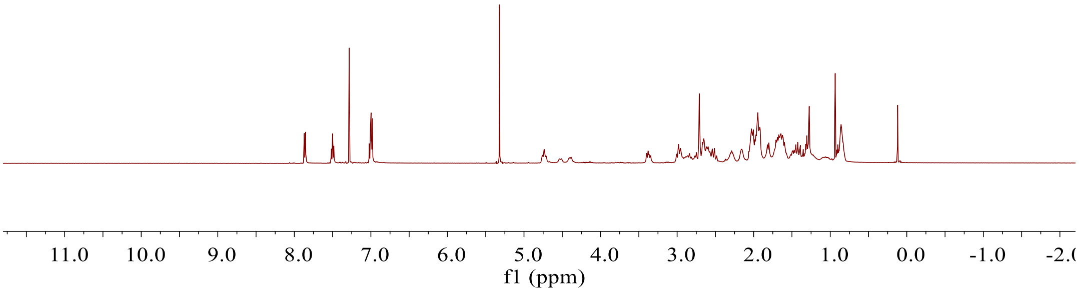

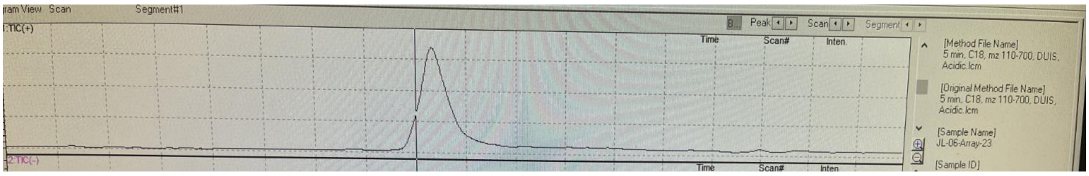

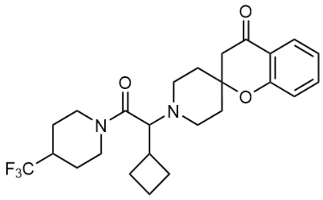

46
array 23

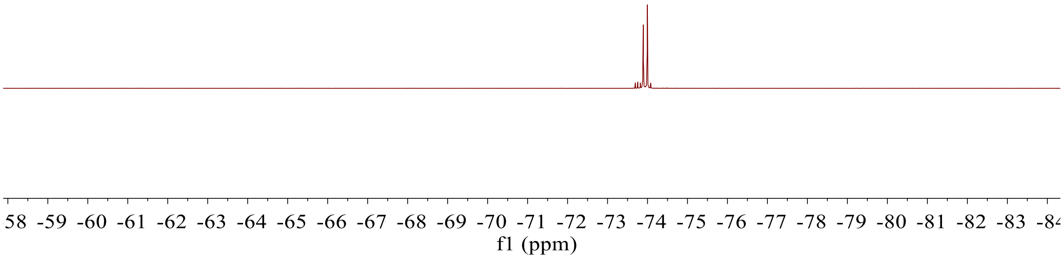

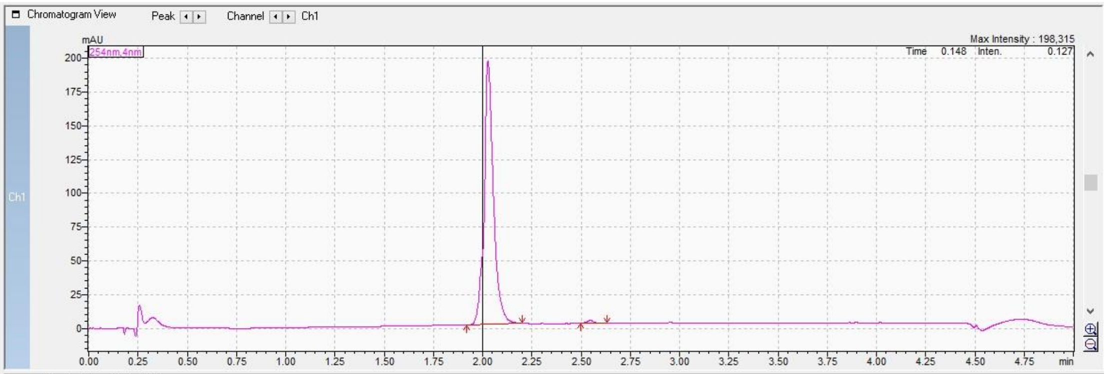

Results View - Peak Table

Peak Table | Compound | Group | Calibration Curve

Peak#	Ret. Time	Area	Height	Mark	Conc.	Unit	ID#	Name	Area%
1	2.028	641766	193471	M	0.000				99.112
2	2.548	5748	2258	M	0.000				0.888
Total		647514	195729		0.000				100.000

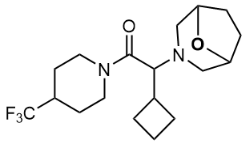

47
array 24

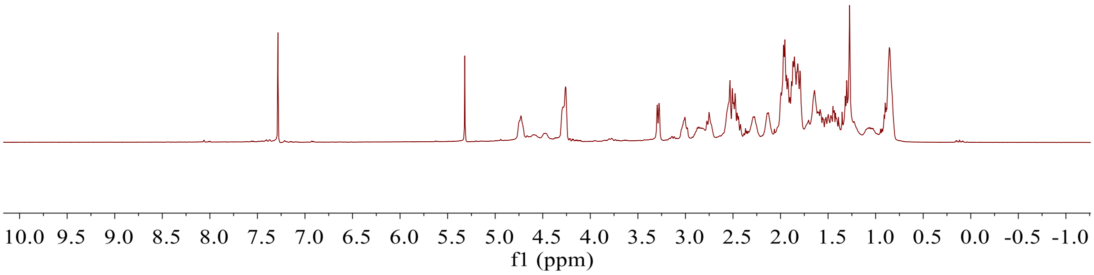

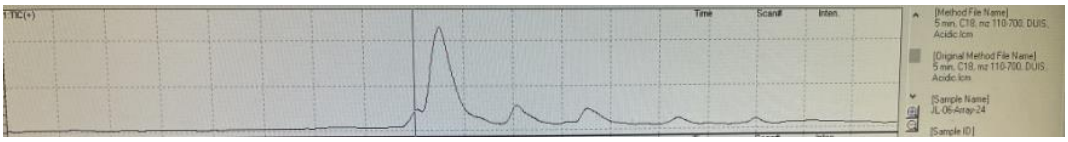

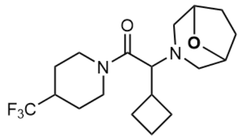

47
array 24

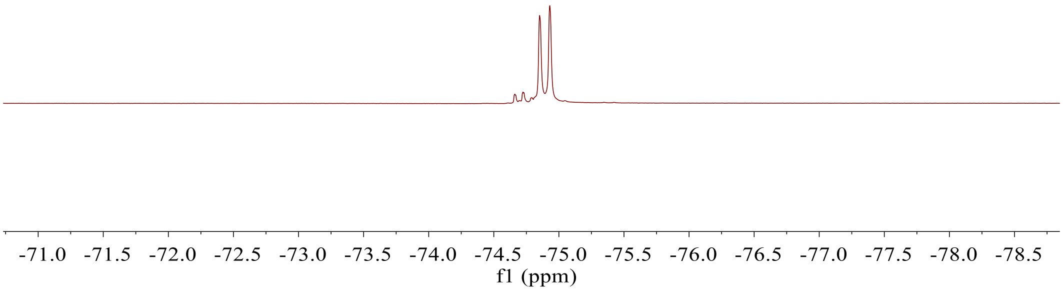

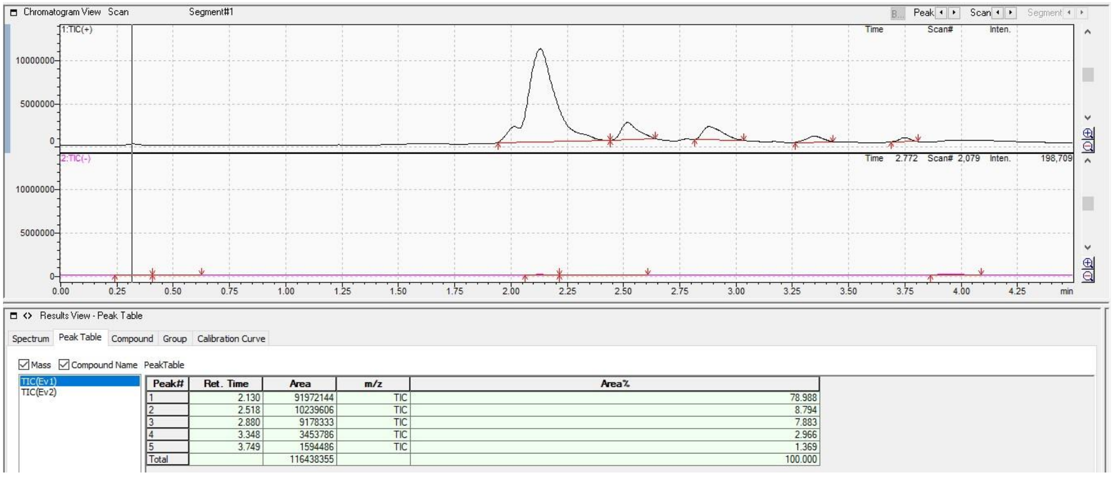

Peak#	Ret. Time	Area	m/z	Area%
1	2.130	91972144	TIC	78.988
2	2.518	10239606	TIC	8.794
3	2.880	9178333	TIC	7.883
4	3.348	3453786	TIC	2.966
5	3.749	1594486	TIC	1.369
Total		116438355		100.000
